# ﻿A revision of the “spiny solanums” of Tropical Asia (*Solanum*, the Leptostemonum Clade, Solanaceae)

**DOI:** 10.3897/phytokeys.198.79514

**Published:** 2022-06-01

**Authors:** Xavier Aubriot, Sandra Knapp

**Affiliations:** 1 Université Paris-Saclay, CNRS, AgroParisTech, Ecologie Systématique et Evolution, 91190, Gif-sur-Yvette, France The Natural History Museum London United Kingdom; 2 The Natural History Museum, Cromwell Road, London SW7 5BD, UK Université Paris-Saclay Paris France

**Keywords:** Asia, endemism, islands, Leptostemonum, spiny solanums, taxonomy, tropical forests, weeds

## Abstract

The Leptostemonum Clade, or the “spiny solanums”, is the most species-rich monophyletic clade of the large cosmopolitan genus *Solanum* (Solanaceae) and represents almost half the species diversity of the genus. Species diversity in the clade is highest in the Americas, but significant clusters of endemic taxa occur in the Eastern Hemisphere. We present here a taxonomic revision of the 51 species of spiny solanums occurring in tropical Asia (excluding the island of New Guinea, and the lowlands of Nepal and Bhutan). Three species are described as new: *Solanumkachinense* X.Aubriot & S.Knapp, **sp. nov.** from northern Myanmar, *S.peikuoense* S.S.Ying, **sp. nov.** from Taiwan, and *S.sulawesi* X.Aubriot & S.Knapp, **sp. nov.** from northern Sulawesi, Indonesia. Of the spiny solanums occurring in the region, 38 are native and 13 are introduced from the Americas or Africa, either as adventive weeds or as cultivated plants. Phylogenetic resolution amongst these taxa is still a work in progress, so we have chosen to treat these taxa in a geographical context to aid with identification and further taxon discovery. For the native species we provide complete nomenclatural details for all recognised species and their synonyms, complete descriptions, distributions including maps, common names and uses, and preliminary conservation assessments. For the introduced taxa that have been treated in detail elsewhere we provide details of types, synonyms based on tropical Asian material, general distributions, and common names for the region. We provide lecto- or neotypifications for 67 names; 63 for native and 4 for introduced taxa. All taxa are discussed and compared to similar species; keys are provided for all taxa. We illustrate all native species with herbarium and field photographs and introduced species with field photographs only. All specimens examined for this treatment are included in Suppl. materials 1–3 as searchable files.

## ﻿Introduction

*Solanum* L. is one of the ten most species-rich genera of flowering plants (Frodin 2004) and has approximately 1,300 species occurring on all temperate and tropical continents. Species of *Solanum* have flowers with fused sepals and petals that are usually 5-merous, stellate to pentagonal corollas, stamens with short filaments, and anthers opening by terminal pores. The highest diversity of both groups and species is in tropical South America, concentrated in a circle around the Amazon Basin (see Knapp 2002a), but significant diversity occurs in Africa, Asia, Australia (incl. New Guinea), and the Pacific (Bean 2004, 2016; McClelland 2012; Knapp and Vorontsova 2016; Vorontsova and Knapp 2016; Echeverría-Londoño et al. 2020). *Solanum* was one of Linnaeus’s (1753) larger genera; he included 23 species, most of them from Europe, Africa, or Asia. The last time *Solanum* was monographed in its entirety was in De Candolle’s *Prodromus* (Dunal 1852), which included 901 species (with an additional 19 recorded as incompletely known by him at the time). Until the 21^st^ century, the taxonomy of *Solanum* was largely limited to rearrangements of infrageneric taxa, species-level treatments of smaller groups within the genus, and floristic works. The large size of *Solanum* and its poorly understood infrageneric structure has meant that *Solanum* taxonomy has proceeded in a piecemeal fashion until relatively recently and the genus had acquired a reputation of being intractable. A project funded by the United States National Science Foundation’s Planetary Biodiversity Inventory (PBI) program begun in 2004 has sought to accelerate species-level taxonomic work across the genus and has resulted in a series of monographic and phylogenetic treatments (e.g., Tepe and Bohs 2011; McClelland 2012; Stern et al. 2013; Knapp 2013a; Clark et al. 2015; Särkinen et al. 2015; Wahlert et al. 2015; Aubriot et al. 2016a; Knapp and Vorontsova 2016; Vorontsova and Knapp 2016; Knapp et al. 2017, 2019a; Särkinen et al. 2018). An electronic monographic treatment of the entire genus is being made available online in the web resource Solanaceae Source (http://www.solanaceaesource.org). This treatment of the tropical Asian spiny solanums is part of that collaborative effort.

*Solanum* is divided into 12 major clades, of which the Leptostemonum Clade is the most species-rich monophyletic group, comprising 578 of the 1,244 currently recognised species of the genus (Gagnon et al. 2022). Traditionally recognised as subgenus Leptostemonum Bitter, the Leptostemonum Clade is characterised by possession of tapered anthers, stellate trichomes (see Morphology) and epidermal prickles; it has been recognised as a natural group since the time of Linnaeus (1753). While most of the diversity of the clade is in the Americas, considerable species-richness is found in Australia and in tropical Asia.

Here we treat the species of spiny solanums occurring in tropical Asia (see Table 1) excluding taxa found only in Australia and the island of New Guinea where *Solanum* species richness and diversification rates are high (ca. 60 and 170 species estimated for New Guinea and Australia, respectively including introduced taxa) with new species being described regularly (e.g., McDonnell et al. 2019). These taxa have been treated in previous monographs (Symon 1981, 1985; Bean 2004, 2016) and are largely endemic. Species that occur in both those regions and across the wider tropical Asian region, however, are included here (e.g., *S.dunalianum*, *S.schefferi*, *S.torvoideum*). Nepal and Bhutan are also not treated here; both have small portions of territory that are tropical in nature but share all of their spiny solanum species with India and China (see descriptions). Past and future floristic work (Mill 2001; http://www.floraofnepal.org/onlineflora) treats these taxa in the context of those countries as a whole. Descriptions and full synonymy are provided for all native species (details for introduced species are provided in other sources and on Solanaceae Source, www.solanaceaesource.org), and a key to all species, native and introduced, is provided.

**Table 1.** Distribution and status of the species of spiny solanums in tropical Asia. Single country endemics are in bold-face type. Species inclusion in subclades taken from Stern et al. (2011), Vorontsova et al. (2013), Aubriot et al. (2016a) and Gagnon et al. (2022). Country-level distribution is presented in Table 2.

Species	Status in tropical Asia	Distribution	Species group in Whalen (1984)	Subclade (see above)
*Solanumaculeatissimum* Jacq.	Introduced	Weedy worldwide in tropics and subtropics; native to southern South America	*S.mammosum* group	Acanthophora
*Solanumaethiopicum* L.	Introduced; cultivated	Only known from cultivation; originally from Africa	*S.anguivi* group	Anguivi grade
*Solanumarundo* Mattei	Native; possibly introduced?	India; East Africa	*S.arundo* group	Dennekense clade
*Solanumbarbisetum* Nees	Native	China, India and adjacent Myanmar	Not included	Un-named group with *S.praetermissum* and *S.wightii*
***Solanumcamranhense* Dy Phon & Hul**	**Native**	**Vietnam**	Not included	*S.camranhense* and relatives
*Solanumcapsicoides* All.	Introduced	Widespread weed in tropics and subtropics; native to southern South America	*S.mammosum* group	Acanthophora
*Solanumchrysotrichum* Schltdl.	Introduced	Weedy in tropics and subtropics; native to Mexico and Central America	*S.torvum* group	Torva
***Solanumcomitis* Dunal**	**Native**	**Indonesia (Java)**	Not included	Unknown
*Solanumcordatum* Forssk.	Native	Western India	Not included	sister to Giganteum clade
*Solanumcyanocarphium* Blume	Native	Indochina and Malay Archipelago to Philippines	Unusual species	unplaced
***Solanumdeflexicarpum* C.Y.Wu & S.C.Huang**	**Native**	**China (Yunnan)**	Not included	*S.violaceum* and relatives
*Solanumdunalianum* Gaudich.	Native	Indonesia (Sulawesi); also on New Guinea and in the Pacific	*S.dunalianum* group	Sahul-Pacific
*Solanumelaeagnifolium* Cav.	Introduced	Weedy and invasive; native to the Americas (amphitropical)	*S.ellipticum* group	Elaeagnifolium
*Solanumforskalii* Dunal	Native	Western India	Not included	Unknown
*Solanumgiganteum* Jacq.	Native	Tropical and subtropical Africa, peninsular India to Sri Lanka	*S.giganteum* group	Giganteum
***Solanumgraciliflorum* Dunal**	**Native**	**Indonesia (except Borneo)**	Unusual species (related to *S.anguivi* group)	Sahul-Pacific
***Solanumharmandii* Bonati**	**Native**	**Cambodia**	Not included	Unknown
***Solanumhovei* Dunal**	**Native**	**India**	Not included	*S.violaceum* and relatives
*Solanuminsanum* L.	Native	Tropical Africa to Indochina and the Philippines	*S.incanum* group	Eggplant
*Solanuminvolucratum* Blume	Native	Indochina and Malay Archipelago (incl. Christmas Island)	Not included	*S.expedunculatum* and relatives
*Solanumjamaicense* Mill.	Introduced	Weedy, scattered introductions; native to Caribbean and Central America	Unusual species	Micracantha
***Solanumkachinense* X.Aubriot & S.Knapp**	**Native**	**Myanmar**	Not included	Unknown
*Solanumlasiocarpum* Dunal	Native	Widespread; cultivated	*S.quitoense* group	Lasiocarpa
*Solanummacrocarpon* L.	Introduced	Cultivated; relatively widespread, but rare outside Africa	*S.incanum* group	Anguivi Grade
*Solanummammosum* L.	Introduced	Widespread weed, sometimes cultivated; native to southern South America	*S.mammosum* group	Acanthophora
*Solanummelongena* L.	Cultivated (native)	Widespread in cultivation	*S.incanum* group	Eggplant
*Solanummiyakojimense* T.Yamaz. & Takushi	Native	Japan (Ryuku Islands), offshore islands of Taiwan, Philippines	Not included	Unknown
***Solanummultiflorum* Roth**	**Native**	**India**	Not included	*S.violaceum* and relatives
*Solanumnienkui* Merr. & Chun	Native	China (Hainan Island), Vietnam	Not included	*S.camranhense* and relatives
***Solanumpeikuoense* S.S.Ying**	**Native**	**Taiwan**	Not included	Torva
***Solanumpoka* Dunal**	**Native**	**Indonesia**	Not included	Torva
*Solanumpraetermissimum* Kerr ex Barnett	Native	Southern China, Indochina to northeastern India	Not included	Un-named group with *S.barbisetum* and *S.wightii*
*Solanumprocumbens* Lour.	Native	Southern China, Indochina to Indonesia	Miscellaneous species related to the broad *S.anguivi* group (as synonymof *S.trilobatum*)	*S.expedunculatum* and relatives
*Solaumpseudosaponaceum* Blume	Native	Taiwan, Japan (Ryuku Islands), Philippines, southern China, Indochina to Indonesia [Papua New Guinea]	Not included	Torva
*Solanumpubescens* Willd.	Native	India	Uunsual species (related to *S.somalense* Franch.)	Giganteum
***Solanumputii* Kerr ex Barnett**	**Native**	**Thailand**	Not included	*S.camranhense* and relatives
*Solanumretrorsum* Elmer	Native	Indonesia, Philippines	Not included	unplaced
***Solanumrobinsonii* Bonati**	**Native**	**Vietnam**	Not included	*S.camranhense* and relatives
*Solanumrobustum* H.Wendl.	Introduced	Weedy, scattered distribution; native to southern South America	*S.erythrotrichum* group	Erythrotrichum
*Solanumschefferi* F.Muell.	Native	Indonesia, Philippines [Papua New Guinea, Solomon Islands]	Not included	*S.athenae* and relatives; Sahul-Pacific (but see text)
*Solanumsisymbriifolium* Lam.	Introduced	Widespread weed; native to southern South America	Unusual species	Sisymbriifolium
***Solanumsulawesi* X.Aubriot & S.Knapp**	**Native**	**Indonesia (Sulawesi)**	Not included	Unknown
*Solanumtorvoideum* Merr. & L.M.Perry	Native	Indonesia [Papua New Guinea]	Not included	Torva
*Solanumtorvum* Sw.	Introduced	Widespread weed; native to Caribbean and Central America	*S.torvum* group	Torva
*Solanumtrilobatum* L.	Native	India, Bangladesh, Sri Lanka, Malaysia and Thailand to Indochina	Miscellaneous species related to the broad *S.anguivi* group	*S.trilobatum* + *S.usaramense*
*Solanumvagum* Nees	Native	India, Sri Lanka	Not included	unplaced
*Solanumviarum* Dunal	Introduced	Widespread weed throughout; native to southern South America	*S.mammosum* group	Acanthophora
*Solanumviolaceum* Ortega	Native	Widespread throughout, but not south of Indonesia	*S.anguivi* group (as *S.violaceum* Jacq.)	*S.violaceum* and relatives
*Solanumvirginianum* L.	Native	Widespread from India and Sri Lanka to China; also Arabian Peninsula and (cultivated?) in northern Africa	*S.incanum* group	unplaced
***Solanumwightii* Nees**	**Native**	**India**	Unusual species (as synonym of *S.pubescens*)	Un-named group with *S.barbisetum* and *S.praetermissum*
*Solanumwrightii* Benth.	Introduced	Cultivated; native to Bolivia	*S.crinitum* group	Crinitum

## ﻿Taxonomic history and phylogeny

Solanums from Asia were among the first tropical species to be described by European botanists. Linnaeus described three of the taxa treated here (Linnaeus 1753, *S.melongena* and *S.trilobatum*; Linnaeus 1767, *S.insanum*), and another four before the beginning of the 19^th^ century. Some of these (e.g., *S.giganteum*) were described from material cultivated in European botanic gardens, as extensive botanical exploration of the region had not yet begun. João de Loureiro, a Portuguese missionary, compiled the first local flora for tropical Asia (Loureiro 1790), based on his long residence in the southern third of today’s Vietnam, then known as ‘Cochinchina’ (Merrill 1935). He used both the Linnaean sexual and binomial systems and included six species of spiny solanums, only one of which, *S.procumbens*, was newly described.

The Scottish surgeon and botanist William Roxburgh lived and worked in northern India in the late 18^th^ century. He was Superintendent of the British East India Company’s botanical garden in Calcutta (Kolkata), and began work on a monumental flora of India, but this was only published posthumously, edited by the British botanist William Carey with comments and additions by Nathaniel Wallich (Roxburgh 1824), who noted that most of the taxa thought new by Roxburgh had already been described earlier. In his notes, Wallich carefully added both distributional and morphological details for all the *Solanum* species described.

Nathaniel Wallich, Danish prisoner-of-war and later director of the Calcutta Botanic Garden, collected intensively in India and Nepal in the early part of the 19^th^ century. He also amassed herbarium specimens from other collectors in the region (De Candolle and Radcliffe-Smith 1981). In 1828 he came to London due to ill health, and spent several years working with many British and continental botanists on the “Wallich Catalogue” (A numerical list of dried specimens of plants in the East India Company’s Museum, collected under the superintendence of Dr. Wallich of the Company’s Botanic Garden at Calcutta); this remained an unpublished list of plants from the region (now fully searchable online, see http://wallich.rbge.info/). A treatment of Solanaceae from this herbarium was published by Christian G. Nees van Esenbeck, the director of the herbarium in Breslau (now Wrocław, Poland). He recognised 14 species of spiny solanums, validating one of Wallich’s names (*S.vagum*) and describing as new *S.wightii*; the work also presented comprehensive synonymy for all species (Nees van Esenbeck 1834). Few new taxa were added to the understanding of the solanaceous flora of India in the monumental “Flora of British India” (Clarke 1883).

In Indonesia, then under the control of the Dutch East India Company, C.L. Blume served as director of the botanical gardens in Bogor on the island of Java from 1823 to 1826. He published a flora (Blume 1826) based on his collections and observations in which he included nine species of spiny solanums, the majority of which were described as new taxa (*S.canescens* Blume, *S.cyanocarphium*, *S.involucratum*, *S.pseudosaponaceum*, *S.pseudoundatum* Blume). After he returned to Leiden, where he became the director of the Rijksherbarium, Blume tended to reserve new and interesting collections from southeast Asia for himself and impeded work by others working on the rich flora of the region (Stafleu 1966). This situation changed when Friedrich A.W. Miquel switched his interests from the flora of Brazil to that of southeast Asia. Miquel was offered the opportunity to study the collections of Franz Wilhelm Junghuhn, who refused to surrender the specimens collected during his time as a medical officer in Java to Blume and the Rijksherbarium, where he thought they would remain unavailable for others to study (Stafleu 1966). Eventually the directorship of the Rijksherbarium was passed to Miquel, who in the 1850s began his own flora of the region (Miquel 1857), incorporating material from a wide variety of sources. He recognised 28 species of spiny solanums for the region, including some he had not seen material of but expected to find there (e.g., *S.virginianum*, as *S.xanthocarpum* Schrad. & J.C.Wendl.). Two of these were Australian species (*S.horridum* Dunal, *S.brownii* Dunal [as *S.violaceum* R.Br.]) that he suggested might occur on the island of Timor; we have seen no evidence of these taxa there.

Michel-Félix Dunal used all these floristic works in his treatment of Solanaceae for the *Prodromus* (Dunal 1852), but often did not see the material used by earlier authors, so names for spiny solanums in the region proliferated. Of the 53 names that were validly published by Dunal for native tropical Asian spiny solanums in his different taxonomic treatments (Dunal 1813, 1814, 1852), only six are accepted here (*S.comitis*, *S.forskalii*, *S.graciliflorum*, *S.hovei*, *S.lasiocarpum* and *S.poka*). Since the major global monograph of *Solanum* done by Dunal (1852) the taxa from this region have never been treated in other than checklists or regional floras (e.g., Matthew 1983; Singh 1991; Mohanan and Henry 1994; Zhang et al. 1994; Naithani et al. 1997; Hul and Dy Phon 2014).

The early floristic works in general did not discuss relationships of the species they described, but they did always divide the solanums into those with and without spines (more correctly prickles, see Morphology). Dunal (1813, 1816) divided *Solanum* into two major groups, *Inermia* (unarmed solanums) and *Aculeata* (armed solanums), based on presence or absence of prickles. He renamed these groups as section Pachystemonum Dunal, for taxa with stout anthers and no prickles, and grad. ambig. *Leptostemonum* for taxa with tapering anthers that were never glabrous and usually possessed prickles (Dunal 1852). Bitter (1919) recognised and validated *Leptostemonum* at the subgeneric rank, and the group has been demonstrated to be well-supported as monophyletic using phylogenetic reconstruction with DNA sequence data (Bohs 2005; Levin et al. 2006; Weese and Bohs 2007; Stern et al. 2011; Särkinen et al. 2013; Gagnon et al. 2022), if prickly species such as *S.nemorense* Dunal and *S.wendlandii* Hook., now members of the *S.nemorense* group and the *S.wendlandii* group respectively, are excluded (see Stern et al. 2011; Clark et al. 2015; Gagnon et al. 2022).

Schemes for the taxonomic grouping of the many species of the Leptostemonum Clade were presented by Dunal (1852, as grad. ambig. *Leptostemonum* with 19 groups of species), Seithe (1962, 16 sections), Danert (1970, 15 sections), D’Arcy (1972, 22 sections), and Whalen (1984, 33 species groups). Seithe (1962), who used trichome morphology extensively in designing her system, only treated a few of the native species from tropical Asia but did not assign most of them to any one of her sections, instead classifying them as “Stellatipilum” (i.e., spiny solanums). She included *S.barbisetum* and *S.lasiocarpum* (as *S.ferox* L.) as members of her “Simplicipilum” along with members of what is now recognised as the Acanthophora clade (e.g., *S.aculeatissimum*), *S.giganteum* as a member of “Torvaria” (=the Torva clade), *S.pubescens* as the type species of section Anisantherum Bitter, *S.nienkui* as a member of section Graciliflorum Seithe, and *S.wightii* as a member of section Nycterium Dunal. Danert (1967, 1970), focusing on sympodial structure, essentially followed Seithe’s (1962) scheme, but did not list component taxa for his sections. D’Arcy (1972) identified or designated lectotypes for all his recognised sections; he, like Danert (1970), did not list component species. He did, however, define section Lasiocarpa (Dunal) D’Arcy, which he typified with *S.lasiocarpum.*Whalen’s (1984) scheme for Leptostemonum had 33 informally named species groups for which he listed component taxa and one additional “group” of unusual species that he could not place (36 species); this scheme has been the backbone for study of spiny solanums since (Levin et al. 2006; Stern et al. 2011). Whalen (1984) treated the tropical Asian spiny solanums in a variety of groups (see Table 1). Child and Lester (2001) presented a modified sectional classification for *Solanum* based in part on Whalen (1984), but like D’Arcy (1972) did not indicate component taxa for their groups.

Molecular phylogenetic studies identified the spiny solanums from Africa, Asia, and Australia (incl. New Guinea) as a strongly supported monophyletic group (Levin et al. 2006; Stern et al. 2011; Vorontsova et al. 2013), but few tropical Asian species were included in these analyses. Aubriot et al. (2016a) looked at relationships amongst these species of spiny solanums including 60 species from Asia and Australia. They found that the vast majority of African, Asian, and Australian species were part of a large monophyletic grouping, the Eastern Hemisphere spiny clade (referred to as the “Old World clade” in previous publications, e.g., Aubriot et al. 2016a; Echeverría-Londoño et al. 2020), and within it, they recovered a number of well-supported clades often composed of species of intermingled geographical origins. Two clades of South American origin were shown to contain five (for the Torva clade) and two (for the Lasiocarpum clade) species originating from the Asian and Australo-Pacific regions. These disjunctions are likely the result of long-distance dispersal (Dupin et al. 2016; Echeverría-Londoño et al. 2020). While all the African species were clustered in a number of clades that had been identified previously (see Vorontsova et al. 2013), most of the tropical Asian taxa were part of large polytomies that also included Australian, New-Guinean and Pacific species; only a few Asian species were found to be nested within the largely African clades (*S.deflexicarpum*, *S.hovei*, *S.insanum*, *S.melongena*, *S.multiflorum*, *S.pubescens*, *S.trilobatum*, *S.violaceum* and *S.virginianum*). The phylogenetic backbone was poorly resolved, this has meant we are still unable to clearly define major clades and undertake sound biogeographical and trait evolution analyses for the spiny solanums of tropical Asia. Future study of the evolutionary relationships of the Eastern Hemisphere spiny solanums will depend upon inclusion of many more species from the Pacific, Australia, and New Guinea, where on-going taxonomic work is adding greatly to the species complement (e.g., Bean 2016; McDonnell et al. 2019; Cámara-Leret et al. 2020; McClelland et al. 2020).

## ﻿Morphology

### ﻿Habit

**Figure 1.**
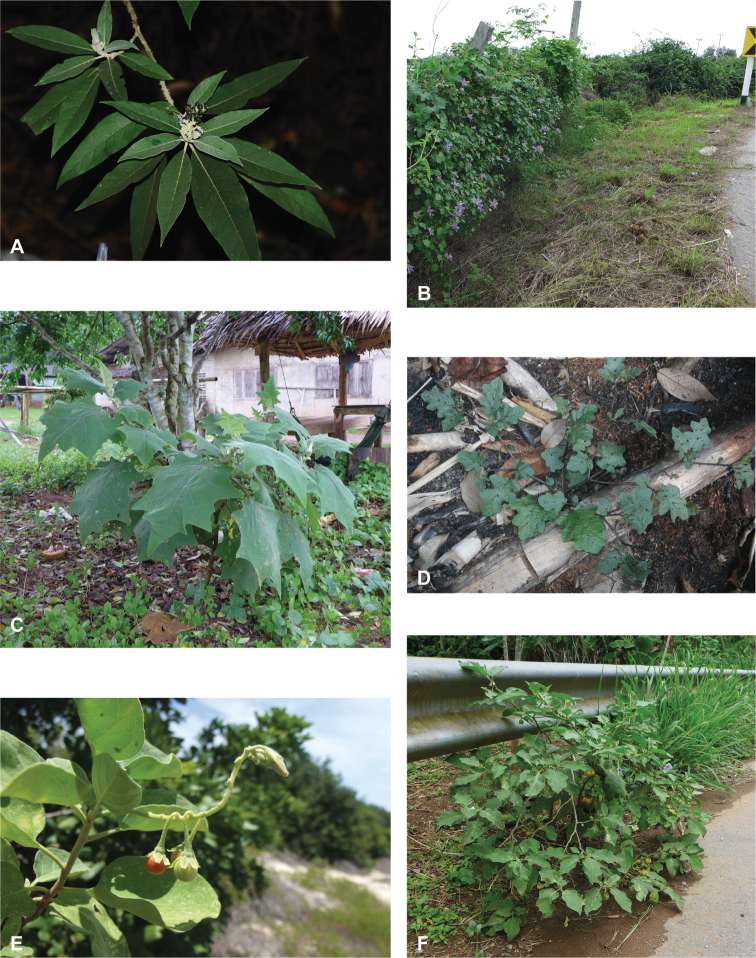
Diversity of habits and habitats for SolanumsubgenusLeptostemonum in tropical Asia **A***Solanumgiganteum*, a small tree of the tropical forest understory in India and Sri Lanka (field photograph, unvouchered, India) **B***S.trilobatum*, a scandent species in its typical habitat, along the road in mangrove area (*Meeboonya et al. RM 245*, Thailand) **C***S.lasiocarpum*, an erect shrub found in disturbed and human inhabited areas where it is often cultivated (*Meeboonya et al. RM 272*, Thailand) **D***S.cyanocarphium*, a creeper with decumbent stems in forest understory (field photograph, unvouchered, Vietnam) **E***S.camranhense*, a scandent shrub endemic to the coastal dunes of South Vietnam (field photograph, unvouchered, Vietnam) **F***S.insanum*, the wild progenitor of the brinjal eggplant usually found in degraded areas (*Meeboonya et al. RM 305*, Thailand). Photograph credits: **A** S. More **B, C, F** X. Aubriot; **D** M. Nuraliev **E** S. Hul.

Spiny solanums in Asia are mostly perennial woody plants (see Fig. 1), but some are short-lived herbaceous perennials, such as the common roadside pioneer *S.violaceum* or the introduced *S.viarum*. Although some species have been characterized as herbs, a truly annual habit is restricted to the cultivated species *S.aethiopicum* and *S.melongena*. Habit varies considerably; species can be erect, prostrate, or climbing, but this distinction is not always clear from herbarium material. Most native species are erect shrubs and usually do not exceed 1–2 m in height. Tree-like shrubs with several stems up to 6 m tall include *S.giganteum* and *S.torvoideum*, but these can reproduce at heights of less than 1 m and reach full height only occasionally. *Solanumwrightii* is cultivated as a shade tree throughout the tropics and subtropics and many other places and can reach 10 cm in diameter when mature. Prostrate or climbing species such as *S.trilobatum* or *S.procumbens* produce long arching stems densely covered in small, curved prickles; these plants rarely rise more than 30 cm above the ground without external support, but they clamber over rocks and other vegetation with the aid of curved prickles. *Solanumschefferi*, however, is a large woody liana with stems to 10 m long. The climbing habit also occurs in American groups and has clearly evolved more than once in the spiny solanums (Whalen 1984; Stern et al. 2011). Some clambering species (e.g., *S.camranhense*, *S.cyanocarphium*, *S.procumbens*, *S.trilobatum*), appear to develop as small shrubs when support is not available.

### ﻿Stems

Sympodial growth is characteristic of Solanaceae giving the stems a typical “zig-zag” appearance; details of sympodial structure have proved useful for infrageneric classification within *Solanum* (Child and Lester 1991; Knapp 2002b). Vegetative growth is initially monopodial, but with the onset of flowering, becomes sympodial. The inflorescence is developmentally terminal, and stem continuation is initiated in the axil of the leaf below each inflorescence. Each lateral shoot with alternate leaves arranged in a 1/3 phyllotaxic spiral and a terminal inflorescence is termed a sympodial unit. In some cases, when the axes of sympodial units are fused, the inflorescences appear to originate laterally from the middle of an internode; and when growth of the axes is suppressed, the leaves appear paired (geminate) at a node (Danert 1958, 1967).

Characteristics of sympodial structure in Eastern Hemisphere spiny solanums (as defined here) do not define monophyletic groups (Aubriot et al. 2016a), as is the case in other groups (e.g., tomatoes, Peralta et al. 2008). Some species have plurifoliate sympodial units with an indeterminate and variable number of leaves between each inflorescence (e.g., *S.pseudosaponaceum*, *S.schefferi*). Others have difoliate sympodial units with two leaves between each inflorescence. In those taxa with difoliate sympodial units, the leaves are either paired (geminate) and one is often smaller than the other (e.g., *S.comitis*, *S.poka*, *S.torvoideum*) or more often are variably spaced on the axes between inflorescences (e.g., *S.sulawesi*, *S.barbisetum*). Genetic control of the number of leaves per sympodial unit is known to involve the tomato self-pruning (*sp*) locus: the sympodial units of *sp* mutants are terminated early and have fewer leaves (Paran and van der Knaap 2007).

### ﻿Leaves

**Figure 2.**
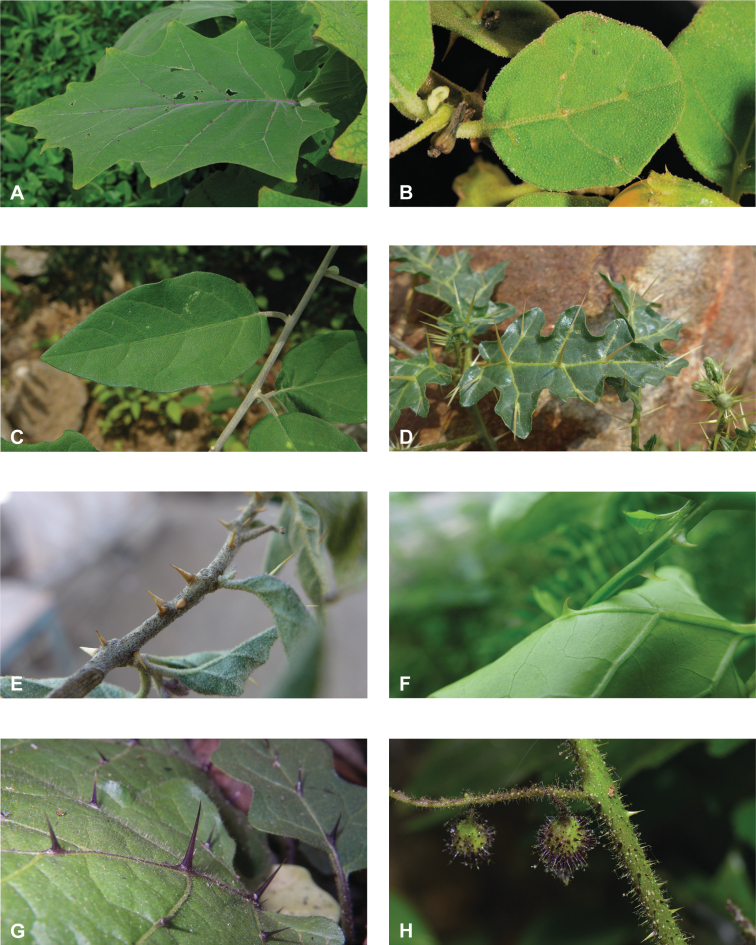
Vegetative characters for tropical Asian spiny solanums **A** large and densely hairy repand leaf of *S.lasiocarpum* (*Meeboonya et al. RM 287*, Thailand) **B** small, pubescent, very shallowly lobed leaf of *S.miyakojimense* (field photograph, unvouchered, Taiwan) **C** entire, pubescent leaf of *S.robinsonii* (*Nuraliev 3031*, Vietnam) **D** glabrescent and deeply dissected leaf of *S.virginianum* (*Sampath Kumar et al. 126968*, India) **E** conical straight prickles on a pubescent young stem of *S.hovei* (field photograph, unvouchered, India) **F** strongly hooked prickles on a glabrous young stem and on the abaxial leaf surface of *S.trilobatum* (*Meeboonya et al. RM 242*, Thailand) **G** needle-like purple prickles on the adaxial leaf surface of *S.cyanocarphium* (field photograph, unvouchered, Vietnam) **H** pubescent young stem of *S.barbisetum* armed with prickles and bristles (*Suksathan et al. PS 3832*, Thailand). Photograph credits: **A, B, D–F** X. Aubriot **C, G** M. Nuraliev **H** D. Pedersen.

Leaf morphology in the Leptostemonum Clade is very diverse (Fig. 2), not only among groups of species, but also within groups and even within individuals of a single species. The highly plastic size, shape, and lobing of the leaves are often the first characters to be noticed by herbarium taxonomists and attributing undue importance to this variability is one of the causes of excessive synonymy. Once enough specimens have been seen to appreciate the infraspecific variability, however, leaf characters do have great value for species recognition. Leaves of tropical Asian spiny solanums are simple and usually shallowly to deeply lobed. Deeply lobed (i.e., pinnatifid or pinnatisect) leaves (see Peralta et al. 2008) are found only in some individuals of *S.arundo*, *S.multiflorum*, *S.violaceum*, *S.virginianum* and in the introduced *S.sisymbriifolium.*

It is common for members of a single species to display multiple leaf morphologies. Species of dry, open habitats often have very small leaves less than three centimetres in length (e.g., *S.camranhense*, *S.cordatum*, *S.procumbens*, *S.trilobatum*), and others have large, repand leaves to 40 cm long (e.g., *S.involucratum*, *S.lasiocarpum*, the cultivated *S.macrocarpon*). These repand leaves have been interpreted as retention of juvenile leaf morphology at an adult stage (Whalen 1984). Interpretation of leaf morphology in the spiny solanums can be complicated by extreme differences in juvenile and adult leaves (Roe 1966). Juvenile, non-reproductive plants usually have larger, pricklier, more deeply lobed leaves, whereas adult reproductive plants have often smaller, less prickly, and more often entire leaves, especially at distal ends of the shoots (e.g., *S.poka*, *S.torvoideum*, *S.torvum*). Petiole length is also usually longer in leaves of juvenile plants.

Despite the variability in leaf shape within and between species, many taxa can be recognized by characteristic leaf morphology. Leaves that are almost as wide as long are found in *S.camranhense*, *S.cordatum*, *S.trilobatum* and *S.wightii* (Figs 10, 14, 74, 82), moderately to deeply lobed leaves with rounded apices are characteristic of *S.cyanocarphium* and *S.graciliflorum* (Figs 16, 27), and deeply lobed, pinnatifid or bipinnatifid leaves are found in *S.arundo*, *S.multiflorum*, *S.violaceum*, *S.virginianum*, and in the introduced *S.sisymbriifolium* (Figs 6, 45, 73, 78, 80).

Characteristics of leaf prickles and trichomes are treated under separate sections of this revision.

### ﻿Inflorescences

**Figure 3.**
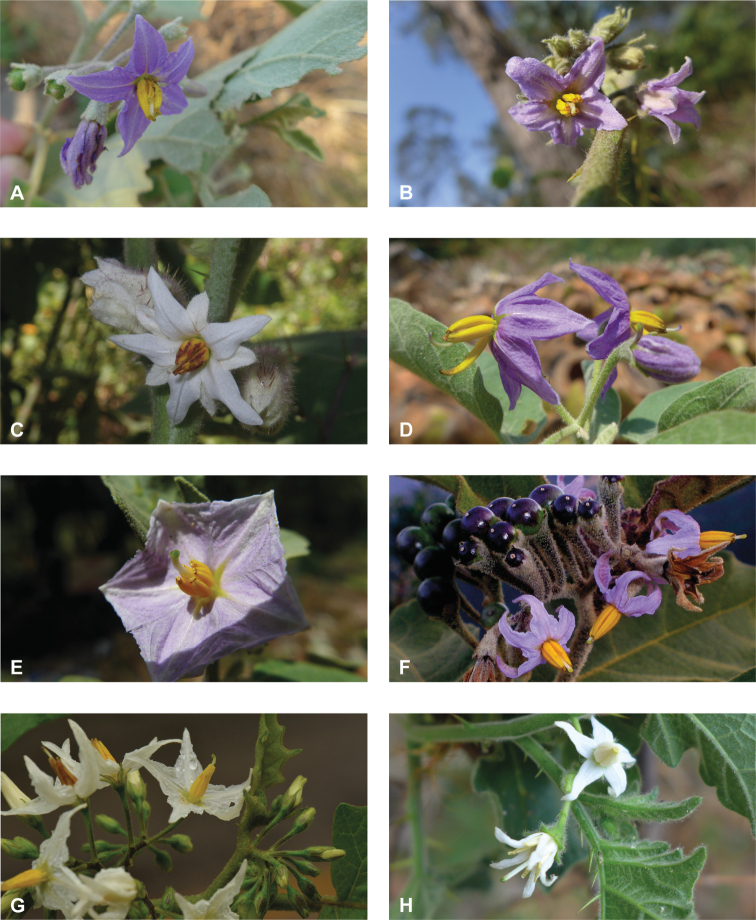
Diversity of inflorescences and flowers in tropical Asian spiny solanums **A** lax, spreading inflorescences and stellate flowers of *Solanumviolaceum* (*Sampath Kumar et al. 126945*, India) **B** condensed, recurved inflorescences and stellate flowers of *S.multiflorum* (*Sampath Kumar et al. 126950*, India) **C** inflorescences and large stellate flowers of *S.involucratum* (field photograph, unvouchered, Vietnam) **D** inflorescences and stellate flowers of *S.pubescens* with unequal stamens, one long and recurved (*Sampath Kumar et al. 126956*, India) **E** large rotate flower of *S.insanum* with abundant interpetalar corolla tissues (*Sampath Kumar et al. 126918*, India) **F** many flowered inflorescence and infructescence of *S.pseudosaponaceum* (field photograph, unvouchered, Philippines) **G** many flowered inflorescences of *S.torvum*; flowers with well-developed interpetalar corolla tissues (*Suksathan et al. PS 3815*, Thailand) **H** small stellate flowers of *S.viarum* (*Sampath Kumar et al. 126944*, India). Photograph credits: **A, B, D, E, H** X. Aubriot **C** M. Nuraliev **F** D. Tandang **G** D. Pedersen.

The *Solanum* inflorescence is a scorpioid cyme with flowers in pairs arranged along a simple axis (Whalen 1984; Knapp 2001), with older flowers and fruits at the proximal end and buds developing distally (see Fig. 3). Inflorescences can be unbranched (simple, e.g., *S.barbisetum*), forked (furcate, e.g., *S.peikuoense*) and more or less ‘Y’ shaped with a peduncle of variable length, or more complexly branched and very large (e.g., *S.giganteum*). During inflorescence growth, the inflorescence shoot-meristems develop new axillary branches before terminating in flowers. The study of tomato mutants compound inflorescence (*s*) and *anantha* (*an*) with complex branched inflorescences suggests that transcription factors alter the timing of flower initiation and thus determine the extent of branching and inflorescence size (Lippman et al. 2008).

Most species of tropical Asian spiny solanums have unbranched (e.g., *S.barbisetum*, *S.hovei*, *S.retrorsum*, *S.wightii*) or forked (e.g., *S.peikuoense*, *S.dunalianum*) inflorescences, and many species have both (e.g., *S.arundo*, *S.forskalii*, *S.vagum*). Highly branched complex inflorescences are found in several species (e.g., *S.giganteum*, *S.graciliflorum*, *S.multiflorum*, *S.torvoideum*) but these taxa can sometimes have unbranched inflorescences when young. The number of flowers per inflorescence parallels inflorescence size and varies from 1–150 flowers; this represents the total flower number over the lifetime of an inflorescence and is obtained by counting buds, flowers, fruits, and pedicel scars from any fallen flowers. In general, 1–30 flowers are open at any one time, depending on the size of the inflorescence. The peduncle (distance from the base of the inflorescence axis to the first flower) varies from almost absent (e.g., *S.comitis*, *S.miyakojimense*, *S.torvoideum*) to stout and elongate (e.g., *S.giganteum*, *S.kachinense*). In strongly andromonoecious species (e.g., *S.insanum*, *S.macrocarpon*, *S.melongena*), the hermaphroditic flower is borne very near the base of the inflorescence and a gap occurs before female-sterile (staminate) flowers arise (see Figs 3E, 22G, H, 33, 42).

### ﻿Pubescence

Trichomes are extraordinarily diverse in *Solanum*, including simple, branched, and stellate types with a broad range of morphologies; infrageneric classifications have been proposed on the basis of trichome types (Seithe 1962, 1979). Molecular phylogenetic reconstructions indicate that trichome types do not provide unambiguous definition for monophyletic groups but can be valuable taxonomic characters at the species level (Knapp 2002b; Bohs 2005).

All the species treated in this monograph possess the stellate trichomes characteristic of the Leptostemonum Clade (see Fig. 1 in Vorontsova and Knapp 2016). Minute simple trichomes (papillae) are also present, usually on the new growth; these are sometimes glandular (e.g., *S.torvum*). In the tropical Asian species stellate trichomes are either: 1) porrect, with unicellular acicular rays arranged horizontally in a single plane, and a unicellular midpoint perpendicular to the rays; or 2) multangulate, i.e., with the rays arranged in more than one plane, and sometimes with a midpoint that is longer than the numerous rays; the lepidote trichomes found in some species from Madagascar (e.g., *S.croatii* D’Arcy & Keating in Vorontsova and Knapp 2016) are not present in any of the species treated here, except the introduced *S.elaeagnifolium*. None of the species treated here possesses strictly echinoid trichomes, which are common in the members of the Brevantherum clade (Roe 1971) or in some species of the Dulcamaroid clade (Knapp 2013a). Stellate trichome types can either be stalked (borne on a multiseriate, multicellular base of varying length) or sessile, and eglandular or glandular.

Most species treated here have only porrect-stellate trichomes (e.g., *S.lasiocarpum*, *S.violaceum*). The stalk varies in length and can be up to 0.5 cm long and then the trichomes appear as long bristles with stellae at their apices (e.g., *S.barbisetum*). The midpoint is variable in length (to 2 mm long in *S.barbisetum*) and is sometimes reduced to a rounded structure (e.g., *S.poka*). In some taxa (e.g., *S.cordatum*, *S.schefferi*) the bulbous midpoint can make the trichomes appear somewhat lepidote (with the rays joined together in a plate at the centre). *Solanumcomitis* has trichomes in which the midpoint is completely absent, and unusual upwardly pointing rays. Most species have midpoints that are equal in length or slightly shorter than the rays. The introduced *S.torvum* differs from other similar, but unrelated native taxa, like *S.violaceum*, in the simple glandular trichomes found exclusively in the inflorescence. Multangulate trichomes are found in *S.giganteum* and to some extent in *S.arundo* and *S.vagum*; in *S.giganteum* these give the plants a characteristic scurfy, mealy look. They are also found on the upper leaf surfaces of *S.comitis* and stems of *S.cordatum*.

The simple trichomes of the introduced members of the Acanthophora clade (*S.aculeatissimum*, *S.capsicoides*, *S.mammosum*, and *S.viarum*) are usually interpreted as the midpoints of sessile stellate trichomes that lack rays (Nee 1979; Whalen 1984; Levin et al. 2005). A similar situation occurs in section Gonatotrichum Bitter of the Brevantherum clade (Stern et al. 2013), where transitions between these trichome types can be seen.

Trichomes are usually translucent, often orange-brown and rusty or white on dried material, and sometimes dark purple on living plants (e.g., *S.barbisetum*, *S.cyanocarphium*, *S.involucratum*, *S.praetermissum*). Young shoots of living plants are sometimes purple-tinged due to the dense purple pubescence; this coloration is often not easily visible in herbarium specimens. Although trichomes of most species retain their structure on drying, those of *S.giganteum* are weak-walled and collapse on drying and are easily brushed off from stems and leaves – they are sometimes characterised as “farinaceous” (e.g., Vorontsova and Knapp 2016); the trichomes of *S.pseudosaponaceum* are also somewhat weak, but not to the extent of those of *S.giganteum*. Trichome density is highly variable and can affect the colour of living plants and dried specimens.

Stellate trichomes are morphologically variable within an individual (even on any given organ), within a species, and among species. Trichomes on the veins of the abaxial leaf surface are often larger and often have more numerous rays than those of the lamina (e.g., *S.violaceum*). Trichomes on the leaf upper surfaces are like those on the abaxial surface but usually smaller and less dense. *Solanumcomitis* has unusual multangulate trichomes on adaxial surfaces, with porrect-stellate trichomes abaxially. As is common in *Solanum*, pubescence of new growth is denser than that of mature stems, and usually more glandular.

### ﻿Prickles

Prickles in *Solanum* are epidermal in origin and are thought to be modified multicellular stellate trichomes with layers of elongate and lignified cells (Whalen 1984). The common origin of trichomes and prickles can be observed on young stems of *S.barbisetum* where some trichomes develop longer lignified stalks that become prickles with an apical stellate trichome that is later deciduous. Often prickles can themselves bear trichomes, reflecting their epidermal nature. The development of prickles in *Solanum* has not been studied in detail in any species.

In tropical Asian spiny solanums, prickles are diverse and provide useful taxonomic characters (Fig. 2D–H). Prickles can occur on all above-ground parts of a plant except the corolla and the fruit. Density and distribution of prickles vary with the age of the plant and environmental conditions and, thus, are not particularly useful characters; many species can have branches or entire individuals with no prickles. This plasticity has led to much taxonomic confusion (e.g., *S.retrorsum*). A few species have no prickles in most specimens we have seen (e.g., *S.nienkui*, *S.pubescens*, *S.robinsonii*). Where prickles are present, they are variable in length and can be strongly curved (e.g., *S.cordatum*, *S.schefferi*), straight (e.g., *S.hovei*, *S.sulawesi*, *S.torvoideum* and introduced species of the Acanthophora clade such as *S.viarum*), both curved and straight (e.g., *S.pseudosaponaceum*, *S.violaceum*), or extremely long and thin and forming an indument of soft bristles (*S.barbisetum*). Climbing species usually have sharply curved prickles (e.g., *S.cyanocarphium*, *S.procumbens*, *S.schefferi*, *S.trilobatum*). *Solanumarundo* is distinctive in its combination of broad-based curved prickles on stems and long, straight prickles on leaves (Fig. 6).

Colour of prickles is quite variable between and within species or individuals; they can be green, white, greyish or yellowish brown in dried or living material. In some species (e.g., *S.cyanocarphium*, *S.barbisetum*, *S.insanum*, *S.involucratum*, *S.praetermissum*) some or all of the prickles are purple-tinted with the base or the entire structure light to dark purple. Mechanisms leading to different prickle and trichome colour (see above) are not known in *Solanum*.

### ﻿Pedicels

**Figure 4.**
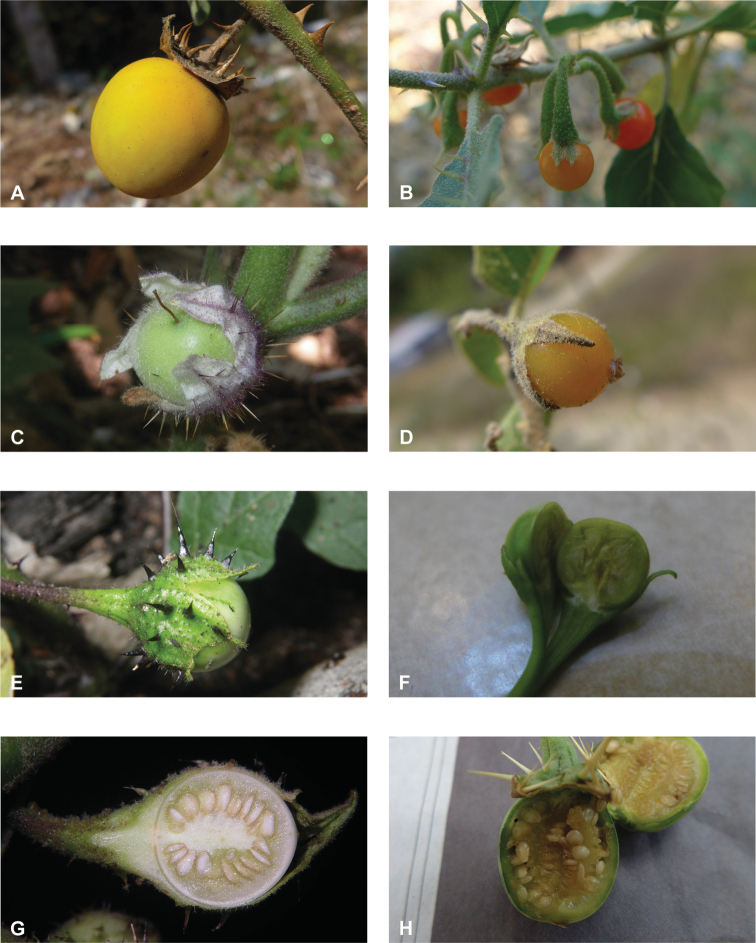
Diversity of fruits and seeds in tropical Asian spiny solanums **A** large oval fruit of *Solanuminsanum* (*Sampath Kumar et al. 126918*, India) **B** condensed infructescence and small rounded fruits of *Solanumhovei* (field photograph, unvouchered, India) **C** large rounded hairy fruit of *S.involucratum* with strongly accrescent and spiny calyx (field photograph, unvouchered, Vietnam) **D** small rounded fruit of *Solanumpubescens* with elongated calyx lobes (*Sampath Kumar et al. 126956*, India) **E** rounded fruit of *Solanumcyanocarphium* with strongly accrescent and spiny calyx (field photograph, unvouchered, Vietnam) **F** small rounded fruit of *S.trilobatum* in transverse cut (*Meeboonya et al. RM 242*, Thailand) **G** rounded fruit and accrescent calyx of *S.praetermissum* in transverse cut (field photograph, unvouchered, Vietnam) **H** oval fruit of *Solanumvirginianum* in transverse cut (field photograph, unvouchered, India). Photograph credits: **A, B, D, F, H** X. Aubriot **C, E, G** M. Nuraliev.

Pedicels are usually herbaceous but, at the fruiting stage in large-fruited species, become woody and up to 9 mm in diameter at the base. Pedicels in most species uniformly abscise less than 1 mm from the base or at the base (see Fig. 4). In contrast, in potatoes and their relatives, the abscission zone is in the middle of the pedicel (Spooner et al. 2014), and in the Dulcamaroid clade, the pedicel bases are sleeve-like (Bohs 2005; Knapp 2013a). In most species, pedicels are usually straight in bud but recurved in flower and fruit (e.g., *S.poka*, *S.torvoideum*), but in others, they are strongly curved in flower but straighten and become erect during fruit development (e.g., *S.giganteum*). Pedicel curvature in fruit is a useful taxonomic character for distinguishing *S.deflexicarpum* and *S.multiflorum* from the sympatric *S.violaceum*; the former two taxa have strongly curved pedicels in fruit whereas *S.violaceum* has straight, spreading pedicels (Figs 3A, B, 18, 45, 78). Conspicuously tapered pedicels that are much wider at the distal end are seen in *S.schefferi* (Fig. 67).

### ﻿Calyx

Calyx morphology has been useful in *Solanum* taxonomy, mostly due to differences in size and shape of the lobes in flower, but also due to developmental or size differences between the calyx in flower and fruit. In bud, the calyx lobes are fused for some to most of their length, and in fruit the calyx lobes of most species lengthen at least to some degree. In most species of *Solanum*, there is no vasculature between the fused calyx lobes, and corolla expansion can tear the calyx tissue between the lobes, sometimes creating lobes that vary in length (D’Arcy 1986). Shape of the lobe and of its apex can provide useful taxonomic characters. In the species treated here, calyx lobes vary from broadly deltate, deltate to long-triangular, and acuminate. Several species have calyx lobes that arise from a truncate tube that often tears at anthesis (e.g., *S.schefferi*, *S.torvoideum*). The long-styled flowers of the cultivated *S.macrocarpon* and *S.melongena* often have large, foliaceous calyx lobes (Figs 22G, H, 42).

In fruit, the calyx lobes of most species expand to some degree (e.g., Fig. 4C–G). We have reported the length of calyx lobes in fruit relative to mature fruit size where possible in the species descriptions. We have described as accrescent only those calyces that enclose the berry at maturity or for most of its development. In these species, the calyx tube is usually the part that expands and encloses the fruit. *Solanumbarbisetum*, *S.cyanocarphium*, *S.involucratum*, *S.praetermissum* and *S.wightii* have accrescent spiny calyces that almost completely cover the berry (Figs 8, 16, 53, 82); other species have calyx lobes that expand slightly during fruit development but are usually reflexed to expose the fruit (e.g., *S.schefferi*). The introduced *S.sisymbriifolium* has a prickly calyx that encloses the berry during development, but at fruit maturity the calyx lobes split, reflex, and expose the bright red fruit.

### ﻿Corolla

The corolla of solanums is sympetalous, usually pentamerous, and actinomorphic. Tetramery occurs in some species (e.g., *S.procumbens*) and has been considered important by earlier workers such as Bitter (1923), but this character varies within species and occasionally within individual plants. We do not consider it to have taxonomic significance among the species treated here. Species cultivated for fruit (e.g., *S.macrocarpon*, *S.melongena*) often have fasciated flowers and exhibit corolla lobe numbers higher than five; fasciation can be a distinguishing characteristic to tell *S.melongena* from its wild progenitor *S.insanum*. *Solanumpubescens* and *S.vagum* have zygomorphic corollas (see also Androecium below) where the lower two lobes are slightly enlarged relative to the upper three lobes (see Fig. 3D). As in other species of *Solanum*, corolla zygomorphy is correlated with androecial zygomorphy (see below) and has evolved multiple times (Lester et al. 1999; Anderson et al. 2007; Bohs et al. 2007).

Most species treated here have corollas that are white or various shades of purple, but in some species (e.g., *S.graciliflorum*, *S.insanum*, *S.procumbens*, *S.violaceum*) corollas of both colours are found and individuals are either white or purple-flowered (See Figs 3, 78). In many species, the corollas are variously violet-tinged or striped (see Fig. 1 of Vorontsova et al. 2013). Flower colour can be useful in distinguishing similar species, for example, two taxa with zygomorphic androecia differ in flower colour, *S.pubescens* consistently has violet corollas whereas *S.vagum* has white corollas. Corolla colour can sometimes change with flower maturity; in the introduced *S.wrightii*, flowers change from dark violet to white with age (Fig. 73G).

Corolla size varies considerably within the species treated here but can be an important character for differentiating species. For tropical Asian native species, the largest corollas are found in *S.wightii* and some cultivars of *S.melongena* (to 5 cm in diameter) and the smallest in *S.harmandii*, *S.nienkui* and *S.putii* (to 0.7–1.6 cm in diameter). Most species have corollas in the range of 1–3 cm in diameter.

Corolla shape varies from rotate-stellate to deeply stellate. Rotate-stellate corollas have short lobes relative to the corolla diameter and are lobed less than halfway to the base (e.g., *S.hovei*, *S.insanum*). Stellate corollas are more deeply lobed. We have characterised corollas with long narrow lobes and an extremely short tube (e.g., *S.nienkui*, *S.schefferi*) as deeply stellate. The corolla lobes are more or less spreading (held perpendicular to the pedicel) at anthesis in most of the species treated here, but in some the lobes are strongly reflexed at maturity (e.g., *S.graciliflorum*); this character is difficult to see on herbarium specimens.

Corollas are usually variously stellate-pubescent on their abaxial surfaces; the trichomes are generally smaller and weaker than those of vegetative parts; they also usually lack midpoints. Areas of the abaxial corolla surface exposed in bud generally have denser pubescence composed of more robust trichomes than does interpetalar tissue, which is usually more or less glabrous.

### ﻿Androecium

All species of *Solanum* have stamens with relatively small filaments basally fused to the corolla tube and anthers that are always longer than the filaments and dehiscent by apical pores. Members of the Leptostemonum Clade have free, slender, and attenuate anthers with distally directed pores, while the non-spiny solanums usually have ellipsoid anthers. Tomato anthers bearing an elongate beak are unique in non-spiny solanums and are not anatomically similar to those of spiny solanums (see Peralta et al. 2008). Amongst Asian spiny solanums, anther length broadly parallels corolla size and breeding system (see below) and can be useful for species differentiation. As the flower ages, the terminal anther pores occasionally elongate, and the anthers appear to “unzip” (D’Arcy et al. 1996; Knapp 2002b). Occasional stellate trichomes are found on anthers, especially along the abaxial connective surface.

Some of the species treated here have anthers of slightly different lengths, with two or three longer that the rest (e.g., *S.graciliflorum*, *S.nienkui*, *S.procumbens*). In these taxa the anthers are not markedly different in shape, and only subtly different in size, or occasionally the filaments differ in length. Markedly zygomorphic androecia are found in three species in the region (*S.pubescens*, *S.vagum*, *S.wightii*). *Solanumwightii* has three longer anthers that are markedly curved, especially in hermaphroditic flowers, and two short straighter anthers (see Fig. 82). In *S.pubescens* and *S.vagum* young flowers or buds have the five anthers of more or less equal lengths, but older flowers have one anther significantly longer than the rest (see descriptions, Figs 59, 76). We do not know if this represents a polymorphism in flower type or growth of the anther during anthesis as happens in *S.turneroides* Chodat (see Stern et al. 2013) and some species of *Lycianthes* (Dunal) Hassl. (Dean 2001). Both those cases involve the elongation of the filament, while in *S.pubescens* and *S.vagum*, it seems the anther itself elongates. Insufficient field observations have been recorded to determine if in these species the longest anther deposits pollen on a different part of the bee than do the shorter anthers, as demonstrated in *S.rostratum* Dunal (Bowers 1975; Vallejo-Marín et al. 2010). The differences in stamen length in these two species are not as pronounced as those documented in strongly enantiostylous solanums (e.g., *S.vespertilio* Aiton and *S.lidii* Sunding of the Canary Islands, see Anderson et al. 2007) so any effect on pollen deposition might be quite small, but may be important in facilitating outcrossing as flowers mature.

### ﻿Gynoecium

The ovary is spherical to ovoid and usually glabrous over most of its surface, but with some minute glandular hairs; stellate trichomes are usually limited to the apical region near the style junction. The size and pubescence of the ovary were considered taxonomically important characters by Bitter (1923), but we have found few taxonomically significant characters in the gynoecium except ovaries with supernumerary carpels in species cultivated for fruits (e.g., *S.aethiopicum*, *S.macrocarpon*, *S.melongena*). *Solanuminvolucratum*, *S.lasiocarpum* and *S.sulawesi* have densely pubescent ovaries with the trichomes persisting to the mature fruit stage (Figs 4C, 39, 69). Styles are broadly similar in these spiny solanums and are usually exserted beyond the anthers (except in short-styled flowers in andromonoecious species, see below), often curved, and apically somewhat dilated. *Solanumpubescens* and *S.vagum* have strongly curved styles that are held next to the longest anthers (Figs 3D, 59), but field studies are needed to determine if these species are truly enantiostylous.

Styles of many of the species treated here are of different lengths in flowers at different positions along the inflorescence rachis. We have called this condition heterostyly in the species descriptions; it is associated with the derived breeding systems dioecy and andromonoecy in *Solanum* (Symon 1970; Symon 1979; Whalen and Costich 1986; Anderson and Symon 1989). We have not seen dioecy in tropical Asian solanums, but it is common in Australian members of the Leptostemonum Clade (Symon 1970; Martine et al. 2009, 2019) and occurs in several other *Solanum* clades (Knapp et al. 1998; Anderson et al. 2015). Andromonoecy, on the other hand, is found in several species of tropical Asian solanums. These species usually have one to few basal flowers with long styles that function as hermaphrodites and varying numbers of distal flowers with short styles that function as staminate flowers (e.g., Figs 3E, 4A). Andromonoecy is described in more detail below (Sex Expression and Breeding Systems).

### ﻿Fruits

*Solanum* fruits are usually indehiscent 2-carpellate berries with axile placentation and a matrix that can be juicy, gelatinous, or spongy and dry (see Knapp 2002a for review of fruit types in Solanaceae). *Solanum* berries can be variously modified and are occasionally capsule-like and dry (Whalen 1979; Symon 1981; Knapp 2002a; Knapp et al. 2017). Fruits of *Solanum* in tropical Asia are usually not dry at maturity, except for *S.wightii*, whose berries are completely enclosed in the calyx as is found in other solanums with dry, dehiscent berries (e.g., *S.houstonii* Martyn of Mexico, see Knapp et al. 2017). Field observations of the fruits of *S.wightii* throughout their development are a priority.

Most variation in the fruit of tropical Asian *Solanum* species lies in the size, shape, colour, and thickness or toughness of the pericarp (see Fig. 4). Berry diameters range from 0.6 to 4.5 cm and individual berries contain few (ca. 7) to many (>200) seeds. Berries in species with all hermaphroditic flowers (e.g., *S.multiflorum*, *S.violaceum*) are usually uniformly green when young and ripen to become yellow, then orange, then bright red and soft with a juicy matrix and thin, shiny pericarp (Fig. 4B, F). Larger fruits in andromonoecious species are usually striped or marbled white and green when young and ripen to yellow with thick leathery pericarp (Fig. 4A, H); these fruits often have a sticky or gelatinous matrix (e.g., *S.insanum*, *S.schefferi*).

Most berries in tropical Asia solanums are spherical or more or less globose. The cultivated eggplant (aubergine) *S.melongena* has some cultivars in which berries are not spherical (Fig. 42), some plants of the introduced *S.mammosum* have teat-shaped berries with basal protuberances (Fig. 41B), and *S.schefferi* has ellipsoid fruit (fide Symon 1985). Fruit shape is probably controlled by the same genes identified in tomatoes and peppers; as in the tomatoes, in African spiny solanums, shape features are more pronounced in larger fruit (Van der Knaap and Tanksley 2003). The elongated apically pointed fruits of the tomato mutants *ovate* and *sun* resemble fruits of *S.schefferi.* Increases in locule number above two are generally found only in species cultivated for their fruits like *S.melongena*.

### ﻿Seeds

Seeds of all spiny solanums treated here are flattened-reniform, somewhat uneven in outline, and have a fine reticulate pattern of testal cell outlines on the seed surface (Lester and Durrands 1984). With a 10× lens, margins of the cells can be seen to be either pentagonal to rectangular (e.g., *S.barbisetum*) or sinuate (e.g., *S.pseudosaponaceum*). This difference in outline of testal cells can be useful in recognising species but can be difficult to see if seeds are not mature. Seed number varies from fewer than ten per berry to more than 200; seed number and fruit size are generally correlated. Most species have seeds in the range of 2–3 mm long, but the seeds of *S.vagum* are the largest at ca. 5 mm long. Seeds of the species treated here are pale yellow to dark orange-brown on herbarium specimens, and pale yellowish tan or creamy white in live plants, but *S.cordatum* and *S.forskalii* of western India have seeds that are dark brown to nearly black.

### ﻿Chromosomes

Chromosome numbers for tropical Asian species are, like all of Solanum, based on a base chromosome number of x = 12. Only ten of the native taxa treated here (*S.forskalii*, *S.insanum*, *S.lasiocarpum*, *S.melongena*, *S.multiflorum*, *S.torvoideum*, *S.trilobatum*, *S.vagum*, *S.violaceum* and *S.virginianum*) have published chromosome counts; these are all either n = 12 (haploid counts) or 2n = 24 (diploid counts). None of the species treated here, native or introduced, has been documented as polyploid (expect *S.elaeagnifolium* in South America; see Scaldaferro et al. 2012), but chromosome counts for many species are not yet available.

## ﻿Ecology and natural history

### ﻿Habitats

Like many species of spiny *Solanum*, tropical Asian taxa are primarily plants of open habitats such as forest clearings, roadsides and areas disturbed by human activities (Fig. 1). The introduced species are usually found in disturbed habitats, and some (e.g., *S.torvum*, *S.viarum*) have become major components of the weedy flora of many areas. Native species occupy a wide range of forest types, from lowland tropical rainforest (e.g., *S.poka*, *S.pseudosaponaceum*) to seasonally dry forests (e.g., *S.nienkui*) to higher elevation grasslands (e.g., *S.hovei*, *S.multiflorum*) and coastal dune systems (e.g., *S.camranhense*). *Solanumarundo*, *S.cordatum* and *S.forskalii* from western India are plants of deserts and extremely dry areas.

In some areas, the adventive species from the Americas like *S.torvum* or *S.chrysotrichum* have achieved local dominance and are completely naturalised. We do not know if they are posing a threat to the less common native taxa, but *S.torvum*, for example, is certainly the most-collected species from tropical Asia in more recent collections, perhaps indicating not only its commonness but the propensity for collection along roadsides and other disturbed areas.

### ﻿Sex expression and breeding systems

Many members of the Leptostemonum Clade exhibit andromonoecy where the first (proximal) “long-styled” flower(s) have styles that protrude beyond the anthers and go on to develop fruits, and the later (distal) “short-styled” flowers have partly developed styles and do not normally develop fruits (e.g., *S.insanum*). Andromonoecy has been extensively studied in *Solanum* (e.g., Dulberger et al. 1951; Whalen and Costich 1986; Anderson and Symon 1989; Miller and Diggle 2003) and is believed to be an adaptation to limited resources which allows the plant to restrict the number of costly fruits without decreasing pollen production. In the spiny solanums there is a continuum from weakly andromonoecious species with a low proportion of staminate flowers (i.e., many hermaphroditic flowers) to strongly andromonoecious species with a high proportion of staminate flowers and only one hermaphroditic flower that sets fruit. A statistically significant correlation has been found between the strength of andromonoecy, larger fruit size, larger ovary size, and larger size of long-styled flowers (Miller and Diggle 2003). Andromonoecy and its correlation with fruit size has formed the basis of many traditional taxonomic systems for spiny *Solanum* (e.g., Bitter 1923 in his treatment of African spiny solanums) but may instead represent a suite of correlated characters driven by breeding system and not by evolutionary relationships. Our phylogenetic studies indicate andromonoecy does not define monophyletic groups (Vorontsova et al. 2013; Aubriot et al. 2016a). Most species of tropical Asian solanums that exhibit heterostyly are only weakly andromonoecious, with several long-styled flowers in the proximal part of the inflorescence. *Solanumlasiocarpum* usually bears one to a few fruits at the base of each inflorescence; *S.melongena* and its wild progenitor *S.insanum* are strongly andromonoecious with usually only a single basal long-styled hermaphroditic flower (e.g., Figs 3E, 33, 42) as is the case also in the introduced *S.macrocarpon* (Fig. 22G, H).

## ﻿Uses

Native tropical Asian species of spiny solanums include two significant fruit crops, *S.melongena* and *S.lasiocarpum.* In addition to these, *S.aethiopicum*, the scarlet eggplant native to Africa is cultivated in some areas for its fruits and leaves (for details of this and the following species and their uses see Vorontsova and Knapp 2016). A few collections of the African Gboma eggplant *S.macrocarpon* have been recorded from Singapore and Sri Lanka, these are all over 50 years old and the species does not appear to have been adopted more widely, although Burkill (1935) suggested it was spreading in the early twentieth century. Several other species of spiny solanums are used locally, both as foods and medicines.

*Solanummelongena*, the brinjal eggplant or aubergine, is the second most commercially important fruit crop in the Solanaceae after tomato (*S.lycopersicum* L.; Daunay and Hazra 2012). The English common name refers to early varieties that were imported from Asia with small, white berries; these are still in cultivation and use in Asia but are no longer commonly found in Europe. *Solanummelongena* has been derived from its wild progenitor *S.insanum* (see Knapp et al. 2013) via artificial selection for domestication, possibly in several different areas across the region (Meyer et al. 2012, 2014; but see Page et al. 2019 for an alternative scenario). The taxonomy and phylogenetic relationships of the largely African wild relatives of the eggplant have been extensively worked on in recent years (e.g., Knapp et al. 2013; Vorontsova and Knapp 2016; Aubriot et al. 2018), with clarifications in nomenclature and species circumscription. The crop has a long history of use in tropical Asia (Daunay and Janick 2007; Wang et al. 2008; Chatterjee and van Andel 2019) and many local varieties, both named and un-named, are in current cultivation (e.g., Caguiat and Hautea 2014; Husnudin et al. 2019). Considerable cultivar diversity also exists outside Asia (e.g., Muñoz-Falcón et al. 2009; Hurtado et al. 2012; Cericola et al. 2013), and recent work in adapting agriculture for climate change (e.g., Dempewolf et al. 2014) and for pest resistance has led to considerable interest in the wild relatives of *S.melongena* for crop improvement purposes (e.g., Prohens et al. 2012, 2013; Kaushik et al. 2016; Kouassi et al. 2016; Plazas et al. 2016; Gramazio et al. 2017; Prohens et al. 2017; Taher et al. 2017; García-Fortea et al. 2019). Many of the wild relatives of *S.melongena*, however, are poorly represented in gene banks and threatened with extinction (Syfert et al. 2016).

Eggplants are valued nutritionally for their high phenolic and antioxidant content (e.g., Wu et al. 2013; Gramazio et al. 2014; Kandoliya et al. 2015; see references in Taher et al. 2017) as well as vitamins and other health-beneficial compounds (Naeem and Ugur 2020). Crosses with wild relatives are being explored to increase content of these nutritionally important compounds in cultivated eggplant (e.g., Prohens et al. 2013). Pre-breeding and breeding efforts are actively being pursed in *S.melongena* using modern molecular breeding methods, more details can be found in the chapters of Chapman (2019) and in Gramazio et al. (2019).

The wild progenitor of the cultivated eggplant, *S.insanum* (recently distinguished from the similar *S.incanum* L. of Africa to Pakistan, see Aubriot et al. 2018), is widely used medicinally across tropical Asia (see table 2 in Ranil et al. 2016). It is clearly distinguished from the cultivated *S.melongena* by local people (see Ranil et al. 2016). Among common uses are relief of chest congestion and rheumatism, but in Ayurvedic medicine *S.insanum* (often cited as *S.incanum* or as a variety of *S.melongena*, http://www.instituteofayurveda.org) is used for treatment of a wide variety of ailments, including overall body pain.

**Table 2.** Country distribution of spiny solanums in tropical Asia as delimited here (excluding the island of New Guinea, see Introduction). Introduced species are shown in brackets, single country endemics in **bold face** type.

Country	Species
Bangladesh	lasiocarpum, melongena, praetermissum, (sisymbriifolium), (torvum), trilobatum, violaceum, virginianum
Brunei Darussalam	lasiocarpum, (torvum)
Cambodia	(capsicoides), cyanocarphium, **harmandii**, insanum, involucratum, lasiocarpum, (torvum), trilobatum
China	(aculeatissimum), (aethiopicum), barbisetum, (capsicoides), (chrysotrichum), **deflexicarpum**, insanum, (jamaicense), lasiocarpum, (macrocarpon), (mammosum), melongena, nienkui, praetermissum, procumbens, pseudosaponaceum, (sisymbriifolium), (torvum), (viarum), violaceum, virginianum, (wrightii)
India	(aculeatissimum), arundo, barbisetum, (capsicoides), (chrysotrichum), cordatum, (elaeagnifolium), giganteum, forskalii, **hovei**, insanum, melongena, **multiflorum**, praetermissum, pubescens, (robustum), (sisymbriifolium), (torvum), trilobatum, vagum, (viarum), violaceum, virginianum, **wightii**, (wrightii)
Indonesia	barbisetum, (capsicoides), **comitis**, cyanocarphium, dunalianum, **graciliflorum**, insanum, involucratum, (jamaicense), lasiocarpum, melongena, **poka**, procumbens, pseudosaponaceum, retrorsum, schefferi, **sulawesi**, torvoideum, (torvum), trilobatum, violaceum, virginianum, (wrightii)
Japan	(capsicoides), melongena, miyakojimense, pseudosaponaceum, (torvum), trilobatum
Laos	(aculeatissimum), barbisetum, (capsicoides), insanum, involucratum, lasiocarpum, praetermissum, procumbens, pseudosaponaceum, (torvum), violaceum
Malaysia	(capsicoides), (chrysotrichum), cyanocarphium, insanum, involucratum, lasiocarpum, melongena, procumbens, torvoideum, (torvum), violaceum, virginianum, (wrightii)
Mauritius and Réunion	insanum, melongena, (robustum), (torvum), violaceum
Myanmar (Burma)	(aculeatissimum), barbisetum, (capsicoides), insanum, **kachinense**, lasiocarpum, (mammosum), melongena, praetermissum, (torvum), (viarum), violaceum, virginianum, (wrightii)
Philippines	cyanocarphium, insanum, (jamaicense), lasiocarpum, (mammosum), melongena, miyakojimense, pseudosaponaceum, retrorsum, schefferi, torvoideum, (torvum), trilobatum, violaceum, (wrightii)
Singapore	insanum, lasiocarpum, (macrocarpon), melongena, (torvum)
Sri Lanka	(capsicoides), (chrysotrichum), giganteum, insanum, lasiocarpum, (macrocarpon), melongena, (torvum), trilobatum, vagum, violaceum, virginianum
Taiwan	(capsicoides), insanum, lasiocarpum, melongena, miyakojimense, **peikuoense**, pseudosaponaceum, (sisymbriifolium), (torvum), violaceum
Thailand	barbisetum, (capsicoides), cyanocarphium, insanum, involucratum, lasiocarpum, (mammosum), melongena, praetermissum, procumbens, **putii**, (torvum), trilobatum, violaceum, (wrightii)
Timor-Leste	insanum, lasiocarpum, procumbens, torvoideum, violaceum
Vietnam	(aculeatissimum), **camranhense**, (capsicoides), cyanocarphium, insanum, involucratum, lasiocarpum, nienkui, praetermissum, procumbens, **robinsonii**, (torvum), trilobatum, (viarum), violaceum, (wrightii)

*Solanumlasiocarpum* of the mostly American Lasiocarpa clade is widely cultivated for its juicy fruit across the tropics of Asia; fruits are used to make sauces and condiments for curries (Whalen et al. 1981; Heiser 1986). It is not yet a commercially important crop in the region or internationally, most use is from home gardens and local plots (Lim 2012). Various plant parts are also used in medicine for a variety of ailments including toothache, respiratory problems, and venereal disease (Lim 2012); Heiser (1986) reports that *S.lasiocarpum* is used medicinally in China.

Fruits of several other spiny solanum species are also used in cooking; the berries of the introduced *S.torvum* are widely used in curries, and are marketed in Europe as pea eggplants, and we have seen one collection label indicating that the berries of *S.cyanocarphium* are eaten. *Solanumtorvum* is widely used as a graft rootstock for eggplant and has been of considerable interest for its biological and pharmacological activity (Jaiswal 2012). Many accounts of *S.torvum* in both the Ayurvedic and Chinese pharmacopeias (Zubaida et al. 2012), mention its use as a vermifuge and for relief from respiratory ailments (as is common for solanums in general). Methanolic extracts of *S.torvum* fruit exhibit considerable antibacterial activity and extracts have been shown to include phenolics that are effective in inducing cancer cell death (Zubaida et al. 2012).

The roots of several species of Asian spiny solanum are important components of the Ayurvedic preparation ‘Dashamoola’ (Daśamūla), also known as the “Ten Roots”. This complex preparation includes roots of ten plants, among them *S.violaceum* (as brhatī, usually as *S.indicum* L., but *S.torvum* and *S.insanum* also used) and *S.virginianum* (as kantakarī, Aparna et al. 2012). Daśamūla is used to relieve fevers, oedema, respiratory troubles, rheumatic arthritis, and general debility; it is widely available as an Ayurvedic remedy (https://www.netmeds.com/health-library/post/dashamoola-benefits-uses-ingredients-dosage-and-side-effects).

*Solanumtrilobatum* is used in the traditional Siddha medicinal system of southern India (Mohanan et al. 1998) to treat a variety of respiratory ailments, particularly bronchial asthma (Govindan et al. 1999, 2004). Aqueous and alcoholic extracts of whole plants were effective as antimicrobials against a wide variety of microorganisms (Latha and Kannabiran 2006), including *Staphylococcus*. Jain and Borthakur (1986) summarise many uses of *Solanum* in India with reference to previous works; care must be taken, however, with these records as vouchers are not often provided and many of the names used are no longer in use (e.g., *S.ferox*, *S.indicum*, *S.surattense* Burm.f.). Other species of tropical Asian spiny solanums have uses recorded on labels or in local floras (e.g., *S.giganteum*); details for these taxa can be found in individual species descriptions.

## ﻿Species concepts

Our goal for this treatment has been to provide circumscriptions for the members of this morphologically variable group of species, while clearly highlighting areas, taxa and populations where further in-depth research would be useful. Delimitation of species here basically follows what is known as the “morphological cluster” species concept (Mallet 1995; Knapp 2008): i.e., “assemblages of individuals with morphological features in common and separate from other such assemblages by correlated morphological discontinuities in a number of features” (Davis and Heywood 1963). Biological (Mayr 1982), phylogenetic (Cracraft 1989) and the host of other finely defined species concepts (see Mallet 1995) are almost impossible to apply in practice and are therefore of little utility in a practical sense (see Knapp 2008). It is important, however, to clearly state the criteria for the delimitation of species, rather than dogmatically follow particular ideological lines (see Luckow 1995; Davis 1997). Our decisions relied on clear morphological discontinuities to delimit species. Specific characters used for recognition are detailed with each species description and in the key. Some potential reasons for variability and intergradation are recent divergence, hybridisation, and environmental influence on morphology. In this revision we have tried to emphasise similarities between populations instead of differences, which so often reflect incomplete collecting or local variation. Although infraspecific taxa have been recognised by others within these taxa, we have not recognised subspecies or varieties, we have rather described and documented variation where present, rather than formalised such variability with a name which then encumbers the literature. We have been conservative in our approach, recognising as distinct entities those population systems (sets of specimens) that differ in several morphological characteristics. Some of the species in this area are extremely widespread and variable (e.g., *S.insanum*, *S.pseudosaponaceum*, *S.torvum*, *S.violaceum*); variation exists in certain characters, but inspection of large number of specimens reveals no apparent natural breaks in variation but rather a mixing between highly morphologically variable populations. For these widespread species the pattern of variation is such that no reliable units can be consistently extracted, nor is geography a completely reliable predictor of character states. Here variability within and between populations seems more important than the variations of the extremes other taxonomists have recognised as distinct. We describe this variation realising that others may wish to interpret it differently.

## ﻿Materials and methods

Our taxonomic treatment is based on study of herbarium specimens and the molecular phylogenetic study of the spiny solanums of tropical Asia (Aubriot et al. 2016a). Delimitation and descriptions are based on field work and examination (physical and virtual) of 12,519 herbarium specimens representing 9,211 collections (of which 4,099 collections and 6,149 specimens were from tropical Asia as treated here) from 162 herbaria: A, AD, AK, ALCB, AMD, ARIZ, AS, B, BA, BAA, BAB, BACP, BH, BHCB, BISH, BK, BKF, BLAT, BM, BO, BR, BRI, BRLU, BRY, BSD, BSHC, BSI, BSIS, C, CAL, CAS, CEPEC, CESJ, CICY, COL, CORD, CPUN, CR, CS, CTES, CUZ, DD, DS, DSM, DUKE, E, EA, ECON, EIU, ENCB, ENLC, ESA, ETH, F, FCQ, FT, FUEL, FURB, G, G-DC, GH, GOET, GZU, HA, HAL, HAO, HAST, HBG, HFSL, HIFP, HITBC, HSTM, HUEFS, HUFSJ, IAC, IBK, IBSC, ICN, INB, IND, INPA, IPA, JBSD, JPB, K, KIEL, KUFS, KUN, K-W, L, LAE, LE, LIL, LINN, LOJA, LPB, MA, MBK, MBM, MBML, MEL, MERL, MESA, MEXU, MH, MICH, MISS, MMNS, MO, MOL, MPU, MT, NA, NOU, NSW, NU, NY, OSC, OXF, P, PAL, PBL, PDA, PE, Q, QAP, QCNE, RB, RENO, RHT, RM, S, SCA, SF, SI, SING, SP, SPF, SPSF, SZ, TAN, TCF, TI, TNM, U, UBC, UBD, UC, UEC, UNM, UPCB, UPS, US, USM, UT, UTC, W, WAG, WUK, XAL, YA, Z. Some of these specimens were examined digitally through individual herbarium portals; we include only those specimens we have been able to unequivocally identify from these images or that are duplicates of collections we have personally examined.

We include detailed descriptions and nomenclature for species native to the region only; introduced taxa are included in the main text with details of distribution and synonyms based on tropical Asian material, in the keys, in Tables 1 and 2 and in the Suppl. materials 1–3. Descriptions for the eleven introduced taxa (*S.aculeatissimum*, *S.aethiopicum*, *S.capsicoides*, *S.chrysotrichum*, *S.elaeagnifolium*, *S.jamaicense*, *S.macrocarpon*, *S.mammosum*, *S.sisymbriifolium*, S. *torvum*, *S.viarum* and *S.wrightii*) are all available on Solanaceae Source (www.solanaceaesource.org) and in other monographic works cited in the text (e.g., Vorontsova and Knapp 2016; Knapp et al. 2017), along with complete nomenclatural details for these names and their synonyms; here we have only listed types and nomenclatural details for synonyms based on tropical Asian types.

Measurements were made from dried herbarium material supplemented by measurements and observations from living material. Colours of vegetative organs (e.g., leaves, prickles, trichomes) and seeds are described from dried herbarium collections (and living plants when available), and for corollas, fruits, etc., are described from living material or from herbarium label data. Specimens with latitude and longitude data on the labels were mapped directly. Some species had few or no georeferenced collections; here we retrospectively georeferenced the collections using available locality data. Maps were constructed with the points in the centres of degree squares in a 1° square grid. Conservation threat status was assessed following the IUCN Red List Categories and Criteria (IUCN 2019) using the GIS-based method of Moat (2007) as implemented in the online assessment tools in GeoCat (http://geocat.kew.org). The Extent of Occurrence (EOO) measures the range of the species, and the Area of Occupancy (AOO) represents the number of occupied points within that range based on the default grid size of 2 km^2^. We have used only the EOO in the threat assessments for widespread species; AOO is very sensitive to georeferencing bias and collecting effort.

Where specific herbaria have not been cited in protologues we have followed McNeill (2014) and designated lectotypes rather than assuming holotypes exist. We cite page numbers for all previous lectotypifications. In general, we have lectotypified names with the best preserved, or in some cases with the only, herbarium sheet we have seen; in these cases, we have not outlined our reasoning for the lectotypifications. Where there has been difficulty or where the choice may not be obvious, we detail our reasoning at the end of the species discussions. For names that have been “inadvertently” lectotypified (sensu Prado et al. 2015), we indicated what the original author cited (e.g., “type”, “holotype”) after the lectotype citation.

Type specimens with sheet numbers are cited with the herbarium acronym followed by the sheet number (e.g., SD [acc. # 6543]); barcodes are written as a continuous string in the way they are read by barcode readers (e.g., G00104280, MO-1781232). For those herbaria (e.g., A, GH, NY, US) where the barcode consists of only a number, we cite only the number string. Where herbaria have both barcodes and accession numbers, we always cite the barcode first, followed by the accession number (e.g., MO-503846, acc. # 3783069); this citation will allow users to access individual sheets where barcode numbers are not human-readable.

Specimens associated with Blume’s flora of the East Indies (Blume 1826) are difficult to pin down. We have assumed that the specimens noted as being collected by Blume in L are as noted; and have used these in typifying Blume’s names unless better material unambiguously attributable to Blume was available.

When typifying the names coined by Christian Godfrey Nees van Esenbeck in his monograph of East Indian Solanaceae (Nees van Esenbeck 1834) we have used the specimens from his own herbarium, now held in Graz (GZU; Stafleu and Cowan 1981), in preference over those in the East India (Wallich) herbarium at Kew (K). They are annotated by Nees van Esenbeck, and we are sure he saw them; the numbering of individual sheets in the Wallich herbarium can be somewhat chaotic.

Identities of all collections seen for this study are in Suppl. material 1 (Index to Numbered Collections; Suppl. material 1) and full searchable specimen details are available in Suppl. material 2 (tropical Asian specimens only) and Suppl. material 3 (all specimens examined) and in the dataset for this study deposited in the Natural History Museum Data Portal (https://doi.org/10.5519/0rqfzvgd). All paratypes for new taxa described here are cited in the text.

Citation of literature follows BPH-2 (Bridson 2004) with alterations implemented in IPNI (International Plant Names Index, http://www.ipni.org) and Harvard University Index of Botanical Publications (http://kiki.huh.harvard.edu/databases/publication_index.html). Following Knapp (2013a) we have used the square bracket convention for publications in which a species is described by one author in a publication edited or compiled by another, the traditional “in” attributions such as Dunal in DC. for those taxa described by Dunal in Candolle’s “Prodromus Systematis Naturalis Regni Vegetabilis”. This work is cited here as Prodr. [A.P. de Candolle] and the names are thus attributed only to Dunal. For “ex” attributions we cite only the publishing author, as suggested in the “Code” (Turland et al. 2018). Standard forms of author names are according to IPNI (International Plant Names Index, http://www.ipni.org).

## ﻿Taxonomic treatment

### ﻿ Solanum subgenus Leptostemonum

Taxon classificationPlantaeSolanalesSolanaceae

Bitter, the Leptostemonum Clade

664DA3A5-02CC-56AD-A07E-8BD77C63593A

[for synonymy see Whalen 1984]

#### Description.

Herbs, vines, scandent or prostrate shrubs, shrubs or small trees; armed or apparently (to almost completely) unarmed; young stems stellate-pubescent, the trichomes multangulate or more often porrect-stellate with the rays all in a single place, occasionally lepidote, sometimes glabrescent; prickles absent, sparse or dense, straight or curved; bark of older stems grey or brown, sometimes glabrescent. Sympodial units plurifoliate or difoliate, the leaves geminate or not. Leaves entire to deeply pinnately lobed, usually stellate-pubescent on both surfaces, sometimes glabrescent or the trichomes very sparse; pubescence of porrect, multangulate or lepidote stellate trichomes, these sessile or variously stalked, with or without midpoints, the midpoints if present very short and bump-like to elongate. Inflorescences lateral and internodal or opposite the leaves, unbranched to many times branched, usually pedunculate, not bracteate, variously stellate-pubescent; petioles articulated near the base. Flowers actinomorphic or zygomorphic, bisexual (hermaphroditic) or unisexual and the plants andromonoecious; calyx 5-parted (rarely 4-parted or more than 5-parted in cultivated forms of *S.melongena*), usually stellate-pubescent, sometimes glabrescent; corolla 5-parted (rarely 4-parted or more than 5-parted in cultivated forms of *S.melongena*), rotate-stellate to deeply stellate, white, lilac or deep purple, some species polymorphic for flower colour, interpetalar tissue absent to copious; stamens 5 (rarely 4 or more than 5 in cultivated forms of *S.melongena*), the filaments equal or unequal, usually glabrous; anthers equal or unequal, yellow or cream, blunt to strongly tapered, tightly connivent or spreading, dehiscing by terminal pores, the pores usually not lengthening to slits with age; ovary bicarpellate (fasciated in cultivars of *S.melongena*), globose to conical, glabrous or stellate-pubescent; style straight or curved, in andromonoecious species shorter than the anthers and held within the anther cone; stigma minutely to large-capitate or clavate, sometimes bilobed. Fruit a berry, usually globose or nearly globose (but variously shaped in *S.mammosum* and *S.melongena*), the pericarp dry, fleshy or leathery, glabrous or stellate-pubescent, the surfaces shiny or matte; fruiting calyx lobes not enlarging or often accrescent, sometimes completely enclosing the berry. Seeds flattened-reniform, the testal cells sinuate or polygonal in outline. Chromosome number: n = 12, 24, 36 (see Scaldaferro et al. 2012).

#### Distribution.

Members of the Leptostemonum Clade are found worldwide, in all habitats but the group is most diverse in the tropics (see Whalen 1984; Bohs 2005; Gagnon et al. 2022). They are often weedy plants of disturbed areas.

#### Discussion.

The Leptostemonum Clade is the largest monophyletic group in the genus *Solanum*. The clade is characterised by the possession of stellate trichomes (sometimes modified), prickles (sometimes absent) and long-attenuate, tapering anthers with distally directed pores that do not elongate to laterally dehiscing slits (as do those of other groups of *Solanum*, see Knapp et al. 2019a). Here we have chosen to treat the species occurring in tropical Asia as a group to fill a significant gap in the understanding of the species level taxonomy of spiny solanums. As for other geographically based groupings in spiny solanums, Asian members of the Leptostemonum Clade do not form a monophyletic assemblage (e.g., Aubriot et al. 2016a; Vorontsova and Knapp 2016); when the species treated have been included in phylogenetic studies, their hypothetical evolutionary relationships are provided in species discussion sections. For further discussion and previous assignments of the taxa treated here to species groups see sections on Taxonomy and Phylogeny above and Table 1.

We have cited types but not reproduced synonymy and complete descriptions of the introduced species here; these can be found in the cited monographic works and on Solanaceae Source. Several of these names are lectotypified here because they were incorrectly cited as having holotypes in previous works. We have cited all synonyms based on specimens from tropical Asia. Many of these introduced taxa are expanding in range with increased disturbance and environmental damage, so the distribution given in Table 2 is what we know now; we expect some of these to be found elsewhere in the future.

##### ﻿Artificial key to tropical Asian spiny solanum species (including non-native adventive taxa)

**Artificial key to all species**

**Table:** 

1	Young stems and/or upper leaf surfaces with simple (unbranched) trichomes only	**2**
–	Young stems and/or upper leaf surfaces glabrous or with stellate, multangulate or lepidote trichomes	**6**
2	Trees to 10 m tall; corollas > 4 cm in diameter with copious interpetalar tissue; anthers 14–16 mm long, slightly curved; mature berries hard, green and subtended by a swollen calyx. Cultivated as street trees	***Solanumwrightii***
–	Shrubs or subshrubs; corollas < 4 cm in diameter, without copious interpetalar tissue; anthers 5.5–12 mm long, straight; mature berries yellow or orange, the calyx not markedly swollen. Weeds of disturbed areas	**3**
3	Flowers pale to medium purple; anthers 8–12.5 mm long; berry globose or variously lobed and extended into a nipple. Introduced and weedy	***Solanummammosum***
–	Flowers white or greenish white; anthers 5–7.5 mm long; berry globose	**4**
4	No stellate trichomes on any part of the plant; berry bright orange; seeds winged. Introduced and weedy	***Solanumcapsicoides***
–	Stellate trichomes present on lower leaf surfaces; berry yellow; seeds not winged	**5**
5	Calyx lobes 5–6.5 mm long, long-acuminate; stems sparsely glandular; ovary stipitate-glandular. Introduced and weedy	***Solanumaculeatissimum***
–	Calyx lobes 0.8–2 mm long, deltate; stems densely and evenly glandular; ovary puberulent. Introduced and weedy	***Solanumviarum***
6	Leaf trichomes lepidote (the ray bases fused into a shield-like structure); rhizomatous shrubs. Introduced and weedy, India	***Solanumelaeagnifolium***
–	Leaf trichomes stellate or multangulate, occasionally almost completely absent; plants without rhizomes	**7**
7	Mature leaves glabrous or very sparsely pubescent above (the pubescence confined to the leaf base or along the midrib)	**8**
–	Mature leaves variously pubescent above, not appearing glabrous, the pubescence easily visible	**19**
8	Stems armed with strongly curved downwards (hooked) prickles	**9**
–	Stems armed with straight prickles or unarmed	**11**
9	Erect shrubs to trees; sympodial units plurifoliate; berries 1.8–3 cm in diameter	***Solanumarundo***
–	Scandent herbs to shrubs to trees; sympodial units difoliate, the leaves geminate; berries 0.3–1.2 cm in diameter	**10**
10	Leaves 7–11 cm long (rarely smaller); corolla 0.5–1 cm in diameter; berries 0.3–0.5 cm in diameter; seeds 6–9 per berry. Indonesia	***Solanumgraciliflorum***
–	Leaves 2.5–6.5 cm long; corolla 2–3 cm in diameter; berries 0.7–1.2 cm in diameter; seeds 16–47 per berry. India and Indochina	***Solanumtrilobatum***
11	Leaves deeply lobed, densely prickly with bright yellow or straw-colored prickles; flowers fragrant. India to China	***Solanumvirginianum***
–	Leaves entire or shallowly lobed, if prickly only sparsely so, the prickles various, but not bright yellow or straw-colored	**12**
12	Androecium zygomorphic, with one anther markedly longer than the rest. Southern India and Sri Lanka	***Solanumvagum***
–	Androecium actinomorphic, all anthers the same length	**13**
13	Stem prickles (if present) downwardly pointing (retrorse); leaves narrowly elliptic to lanceolate. Philippines, Indonesia (?Taiwan, Lanyu Island)	***Solanumretrorsum***
–	Stem prickles (if present) not strongly retrorse; leaves orbicular to elliptic	**14**
14	Scandent to erect shrub; leaves orbicular, as long as wide. Western India	***Solanumcordatum***
–	Erect herbs or shrubs; leaves ovate to elliptic, 1.5–3 times longer than wide	**15**
15	Inflorescence unbranched; berries usually large (usually >1 cm in diameter), often flattened and/or ridged. Cultivated plants, perhaps escaped from gardens	**16**
–	Inflorescence forked to many times branched; berries < 1 cm in diameter (not known in *S.kachinense*)	**17**
16	Berries 4–6 cm in diameter; flowers heterostylous and the plants strongly andromonoecious; corolla subrotate with abundant interpetalar tissue	***Solanummacrocarpon***
–	Berries 1.5–2.5 cm in diameter; flowers all perfect, only occasionally with a few distal staminate; corolla stellate	***Solanumaethiopicum***
17	Pubescence of multangulate trichomes, these scurfy and deciduous; inflorescence many times branched and erect. India, Sri Lanka	***Solanumgiganteum***
–	Pubescence of porrect-stellate trichomes, these merely sparse, not deciduous; inflorescence forked or several times branched, not markedly erect	**18**
18	Leaves drying black or dark brown; leaf base oblique and appearing more or less truncate; major veins 6–8 pairs; corolla 1.5–1.8 cm in diameter. Myanmar	***Solanumkachinense***
–	Leaves drying olive green; leaf base acute to cuneate; major veins 5–6 pairs; corolla 2–2.4 cm in diameter. Taiwan	***Solanumpeikuoense***
19	Trichomes of upper leaf surfaces multangulate (the rays not in a flat plane)	**20**
–	Trichomes of upper leaf surfaces porrect-stellate with the rays in a single flat plane	**21**
20	Inflorescences many times branched; trichomes scurfy and deciduous; upper leaf surfaces glabrate in older plants. India, Sri Lanka	***Solanumgiganteum***
–	Inflorescences unbranched; trichomes not scurfy and deciduous; upper leaf surfaces densely pubescent. Indonesia (Java)	***Solanumcomitis***
21	Inflorescences forked to many times branched	**22**
–	Inflorescences unbranched (simple)	**50**
22	Stem prickles absent (reproductive growth), the stems unarmed	**23**
–	Stem prickles present (reproductive growth), the stems weakly to strongly armed	**26**
23	Inflorescences stellate-pubescent and densely glandular pubescent, the glandular hairs unbranched; rest of plant eglandular. Introduced and naturalised, widespread	***Solanumtorvum***
–	Inflorescences variously stellate-pubescent, not glandular (unless rest of plant densely glandular pubescent)	**24**
24	Leaves shiny above; berries 1–1.3 cm in diameter. Taiwan	***Solanumpeikuoense***
–	Leaves not markedly shiny above; berries less than 1 cm in diameter	**25**
25	Androecium zygomorphic, with one anther markedly longer than the rest; corolla 2–3 cm in diameter; berries 0.8–1 cm in diameter. Southern India and Sri Lanka	***Solanumvagum***
–	Androecium actinomorphic, all anthers the same length; corolla 1–1.4 cm in diameter; berries 0.6–0.8 cm in diameter. Philippines, Indonesia (?Taiwan, Lanyu Island)	***Solanumretrorsum***
26	Stem prickles strongly curved downwards (hooked)	**27**
–	Stem prickles straight	**36**
27	Leaves 1–2 times as long as wide (ovate to elliptic ovate)	**28**
–	Leaves more than 2 times as long as wide (elliptic to narrowly elliptic)	**32**
28	Berries > 2 cm in diameter; prickles of leaves straight, usually >10 mm long. Kathiawar peninsula, Gujarat, India	***Solanumarundo***
–	Berries < 2 cm in diameter; prickles of leaves various, never 10 mm long and straight	**29**
29	Erect shrubs; berries orange when mature; fruiting pedicels strongly spreading. Widespread in the region	***Solanumviolaceum***
–	Scandent shrubs; berries red or red-orange when mature; pedicels erect or pendant, not strongly spreading	**30**
30	Corolla 2–3 cm in diameter; anthers 7–9 mm long; plants of forests and forest edges. India and Indochina to Philippines	***Solanumtrilobatum***
–	Corolla 0.9–2.4 cm in diameter; anthers 3–7 mm long; plants of dry, desert habitats. Western India	**31**
31	Stem pubescence of multangulate trichomes; petioles winged from decurrent leaf bases	***Solanumcordatum***
–	Stem pubescence of porrect-stellate trichomes; petioles not winged	***Solanumforskalii***
32	Leaf margins entire, the leaves not lobed; berries 3–5 cm long, ovoid. Indonesia and Philippines	***Solanumschefferi***
–	Leaf margins variously lobed; berries not as above	**33**
33	Corolla 0.5–1 cm in diameter; anthers unequal in size. Indonesia	***Solanumgraciliflorum***
–	Corolla > 1 cm in diameter; anthers all equal in size	**34**
34	Inflorescence stellate-pubescent and densely and uniformly glandular with small, unbranched glandular trichomes; rest of plant eglandular. Widespread and naturalised	***Solanumtorvum***
–	Inflorescence variously pubescent, never with glandular trichomes, unless rest of plant glandular	**35**
35	Inflorescence several times branched; calyx lobes long-acuminate with a distinct acumen; fruiting pedicels erect to slightly spreading; seeds > 20 per berry. Southern China and Indochina to Philippines	***Solanumpseudosaponaceum***
–	Inflorescence forked (once branched); calyx lobes deltate, apically acute; fruiting pedicels strongly spreading; seeds < 20 per berry. Widespread in the region	***Solanumviolaceum***
36	Stem and leaf trichomes multangulate, often deciduous through rubbing off. India, Sri Lanka	***Solanumgiganteum***
–	Stem and leaf trichomes porrect-stellate (all rays in a single plane), not deciduous	**37**
37	Stem prickles strongly retrorse and downward pointing. Philippines, Indonesia (?Taiwan, Lanyu Island)	***Solanumretrorsum***
–	Stem prickles not strongly retrorse	**38**
38	Pubescence of upper leaf surfaces sparse, scattered and mostly along veins, if present on lamina very sparse	**39**
–	Pubescence of upper leaf surfaces moderate to dense, easily visible to the naked eye	**40**
39	Leaves deeply lobed, the blades 3–11 cm long, 2–4 cm wide, densely prickly; corolla 2.5–3 cm in diameter; prostrate shrubs. India to China	***Solanumvirginianum***
–	Leaves shallowly lobed, the blades 11–22 cm long, 5.2–11.5 cm wide, sparsely prickly ; corolla 1.5–1.8 m in diameter; erect shrubs. Myanmar	***Solanumkachinense***
40	Leaves 1–2 times longer than wide (ovate to broadly elliptic)	**41**
–	Leaves > 2 times longer than wide (elliptic to narrowly elliptic)	**43**
41	Inflorescences many times branched; sympodial units plurifoliate. Vietnam	***Solanumharmandii***
–	Inflorescences forked (once branched); sympodial units difoliate	**42**
42	Scandent shrubs of deserts; leaf bases cordate to rounded; corolla 1.3–2.4 cm in diameter, divided nearly to the base; seeds dark brown, almost black. Western India	***Solanumforskalii***
–	Erect shrubs of many habitats; leaf bases truncate, often oblique; corolla 1.3–3 cm in diameter, divided half to two-thirds of the way to the base; seeds yellow to orange-brown. Widespread in the region	***Solanumviolaceum***
43	Leaves entire, the margins not lobed. Indonesia (Sulawesi)	***Solanumdunalianum***
–	Leaves lobed, the margins shallowly to deeply dissected	**44**
44	Fruiting pedicels curved and strongly deflexed. Western Ghats, India	***Solanummultiflorum***
–	Fruiting pedicels erect or spreading, not curved or deflexed	**45**
45	Leaves ovate; fruiting pedicels strongly spreading. Widespread in the region	***Solanumviolaceum***
–	Leaves elliptic to narrowly elliptic; fruiting pedicels erect or spreading from weight of fruit	**46**
46	Inflorescence densely and minutely glandular-pubescent, the glandular trichomes simple. Naturalised, widespread throughout, except western India	***Solanumtorvum***
–	Inflorescence variously pubescent, no inflorescence trichomes glandular	**47**
47	Corolla 2–5 cm in diameter; leaf pubescence golden or reddish gold	**48**
–	Corolla 1–2 cm in diameter; leaf pubescence not markedly rusty-golden, usually tan	**49**
48	Corolla 2–2.6 cm in diameter; style 6–9 mm long, mature berries yellow to orange yellow; leaf pubescence rusty-golden colour. Indonesia, Philippines	***Solanumtorvoideum***
–	Corolla to 5 cm in diameter; style 10–13.5 mm long; mature berries greenish yellow; leaf pubescence reddish brown. Introduced, China, India, Malaysia, Sri Lanka	***Solanumchrysotrichum***
49	Calyx lobes strongly keeled; corolla white; berries 0.8–1.5 cm in diameter. Indonesia (except Borneo)	***Solanumpoka***
–	Calyx lobes not keeled; corolla purple; berries 0.7–1 cm in diameter. China and Indochina, to Philippines and Taiwan	***Solanumpseudosaponaceum***
50	Leaves entire (occasionally the margins somewhat sinuate, but never distinctly lobed)	**51**
–	Leaves variously lobed	**60**
51	Stem prickles strongly curved	**52**
–	Stem prickles straight or absent	**53**
52	Corolla 2–3 cm in diameter; berries to 5 cm long, ovoid; climbing vines of rainforests. Indonesia, Philippines	***Solanumschefferi***
–	Corolla 0.9–1.6 cm in diameter; berries to 0.8 cm long, globose; scandent shrubs of dry habitats. Western India	***Solanumcordatum***
53	Flowers strongly zygomorphic, with one or two anthers much longer than the rest and strongly curved	**54**
–	Flowers actinomorphic or only weakly zygomorphic, all anthers of equal length or only slightly differing in size, never in shape	**56**
54	Corolla 3.5–5 cm in diameter; long anthers 2, 12–15 mm long; fruiting pedicels strongly deflexed; berries dry and held inside the accrescent calyx. Western Ghats, India	***Solanumwightii***
–	Corolla < 3 cm in diameter; long anther 1, < 10 mm long; fruiting pedicels not deflexed; berries soft and fleshy, the fruiting calyx not accrescent	**55**
55	Flowers white; leaves lanceolate to elliptic, strongly discolorous, eglandular. Southern India and Sri Lanka	***Solanumvagum***
–	Flowers purple; leaves ovate to broadly triangular, concolorous, densely glandular, sticky to the touch. Eastern India, Sri Lanka	***Solanumpubescens***
56	Trichomes of upper leaf surfaces multangulate, all rays pointing upwards; sympodial units difoliate, the leaves geminate; anthers all of equal size. Indonesia (Java)	***Solanumcomitis***
–	Trichomes of upper leaf surfaces porrect-stellate, the rays in a single plane; sympodial units plurifoliate, the leaves not geminate; anthers often slightly unequal	**57**
57	Scandent shrubs; flowers often 4-merous. China, Indochina, Indonesia	***Solanumprocumbens***
–	Erect spindly shrubs; flowers 5-merous	**58**
58	Leaf bases attenuate; corolla deeply stellate, divided nearly to the base. Hainan Island, China, southern Vietnam	***Solanumnienkui***
–	Leaf bases cuneate; corolla stellate, divided ca. halfway to the base	**59**
59	Leaves strongly discolorous, 2–3 times longer than wide; trichomes of stems and leaves with bulbous midpoints. Vietnam	***Solanumrobinsonii***
–	Leaves only slightly discolorous, less than 2 times longer than wide; trichomes of stems and leaves without bulbous midpoints. Thailand	***Solanumputii***
60	Stem prickles absent or extremely sparse (ca. 1–2 per 10 cm or more of stem)	**61**
–	Stem prickles always present, occasionally very dense	**64**
61	Berries smaller than 1 cm in diameter, several per inflorescence	**62**
–	Berries larger than 1 cm in diameter, usually only one or two per inflorescence	**63**
62	Leaves ovate to suborbicular; corolla 0.8–1 cm in diameter; plants of seashore dunes. Vietnam	***Solanumcamranhense***
–	Leaves elliptic to narrowly elliptic; corolla 1–1.4 cm in diameter; plants of forests. Philippines, Indonesia (?Taiwan, Lanyu Island)	***Solanumretrorsum***
63	Corolla 0.8–1 cm in diameter, white; berries bright red, often somewhat flattened or ribbed. Cultivated (scarlet eggplant)	***Solanumaethiopicum***
–	Corolla 2.5–5 cm in diameter, purple or occasionally white; berries white, yellow or purple, variously shaped. Cultivated (brinjal eggplant)	***Solanummelongena***
64	Stem prickles strongly curved (hooked)	**65**
–	Stem prickles straight	**77**
65	Leaves decurrent onto the stem, rhombic or broadly ellipsoid	**66**
–	Leaves distinctly petiolate, variously shaped	**67**
66	Mature berries densely pubescent; stems strongly winged from decurrent leaf bases; flowers greenish white. Introduced, India, Réunion (France)	***Solanumrobustum***
–	Mature berries glabrous and shiny; stems not winged from the leaf bases; flowers white. Introduced, widespread in the region but scattered	***Solanumjamaicense***
67	Erect shrubs	**68**
–	Prostrate or scrambling shrubs (can be as tall as 2 m)	**70**
68	Inflorescence densely glandular-pubescent (in addition to stellate-pubescent), the glandular trichomes simple and minute; rest of plant eglandular. Naturalised throughout the region	***Solanumtorvum***
–	Inflorescence stellate-pubescent, not glandular-pubescent, unless rest of plant with glandular trichomes	**69**
69	Berries 2.5–3.5 cm in diameter, densely pubescent at maturity, the trichomes with elongate midpoints; leaves repand; fruiting pedicels bending from weight of fruit, not markedly deflexed. Cultivated throughout the region	***Solanumlasiocarpum***
–	Berries 1–1.2 cm in diameter, glabrous; leaves not repand; fruiting pedicels strongly deflexed. Yunnan, China (Myanmar?)	***Solanumdeflexicarpum***
70	Leaves elliptic to narrowly elliptic	**71**
–	Leaves ovate to broadly ovate or suborbicular	**73**
71	Calyx accrescent in fruit, covering half or more of the berry, densely prickly; anthers of equal size. Indochina, Indonesia, Malaysia, Philippines	***Solanumcyanocarphium***
–	Calyx not accrescent in fruit, if enlarged not covering half or more of the berry, not markedly prickly; anthers of slightly different sizes	**72**
72	Leaves 7–11 cm long (rarely smaller); corolla 0.5–1 cm in diameter; berries 0.3–0.5 cm in diameter; seeds 6–9 per berry. Indonesia	***Solanumgraciliflorum***
–	Leaves 2–6.5 cm long; corolla 1–1.5 cm in diameter; berries 0.6–1 cm in diameter; seeds to 25 per berry. China, Indochina, Indonesia, Timor Leste	***Solanumprocumbens***
73	Flowers zygomorphic, with two anthers longer than the rest; berries dry, held inside an accrescent calyx on straight, strongly deflexed pedicels. India	***Solanumwightii***
–	Flowers actinomorphic; berries fleshy, the fruiting calyx not accrescent; fruiting pedicels not as above	**74**
74	Peduncle absent to 0.15 mm long, the flowers borne at the very base of the inflorescence; fruiting pedicels strongly recurved, woody; ripe berries orange. Ryuku Islands (Japan) to Philippines	***Solanummiyakojimense***
–	Peduncle to 2 cm long, always present; fruiting pedicels erect or pendant; mature berries red	**75**
75	Corolla 0.8–1 cm in diameter; plants of dunes along seashore. Vietnam	***Solanumcamranhense***
–	Corolla > 1 cm in diameter; habitat not as above	**76**
76	New growth densely stellate-pubescent, the trichomes with 6–10 rays; sympodial units difoliate, the leaves not geminate. Western India	***Solanumforskalii***
–	New growth sparsely stellate-pubescent, the trichomes with 2–5 rays; sympodial units difoliate, the leaves geminate. Widespread, India to Philippines	***Solanumtrilobatum***
77	Mature berries densely pubescent, the trichomes with long midpoints; leaves usually large and somewhat repand	**78**
–	Mature berries glabrous or with only a few stellate trichomes at the apex; leaves various, not repand	**80**
78	Leaf bases attenuate; leaves widest near the middle; upper leaf surface glabrate and densely prickly. Indonesia (Sulawesi)	***Solanumsulawesi***
–	Leaf bases truncate; leaves widest in the lower third; upper leaf surfaces pubescent, prickly or not	**79**
79	Berries completely enclosed in an accrescent, prickly calyx; corolla 1–1.2 cm in diameter; anthers 5–6 mm long. Indochina, Malay Archipelago, Christmas Island	***Solanuminvolucratum***
–	Berries not enclosed in an accrescent calyx; corolla 2.5–3.5 cm in diameter; anthers 6–8.5 mm long. Cultivated throughout the region	***Solanumlasiocarpum***
80	Inflorescences with distal staminate flowers and a single to a few basal hermaphroditic flowers, the plants strongly andromonoecious; berries usually > 2 cm in diameter	**81**
–	Inflorescences with all flowers hermaphroditic (perfect), if staminate flowers occur these are few and at the very tip of the inflorescence of otherwise hermaphroditic flowers; berries usually < 1 cm in diameter	**83**
81	Prostrate shrubs with densely prickly stems and leaves; leaves glabrescent; flowers fragrant. India to Malay Archipelago	***Solanumvirginianum***
–	Herbs or shrubs, moderately to sparsely prickly; leaves not glabrescent; flowers not fragrant	**82**
82	Inflorescences usually with only a single hermaphroditic flower at the base; staminate flowers 1–2; berries usually large, variously shaped. Cultivated (see description, can be very difficult to distinguish from *S.insanum*)	***Solanummelongena***
–	Inflorescences with more than one hermaphroditic flower at the base; staminate flowers > 2; berries 1.5–3 cm in diameter, globose. Weedy throughout	***Solanuminsanum***
83	Calyx accrescent in fruit, covering more than half of the berry, densely prickly	**84**
–	Calyx not accrescent in fruit, if enlarged not covering more than half of the berry, moderately to sparsely prickly	**87**
84	Leaves deeply divided to pinnatifid; corollas 2–3 cm in diameter; accrescent calyx peeling back at fruit maturity to reveal the red berry. Introduced and adventive, China and India	***Solanumsisymbriifolium***
–	Leaves shallowly lobed or entire, not pinnatifid or bipinnatifid; corollas usually less than 2 cm in diameter; accrescent calyx remaining around the berry at maturity. Native plants	**85**
85	Scrambling vine or shrub; leaves elliptic to narrowly elliptic; leaf bases attenuate; berries red when mature. Indochina, Indonesia, Malaysia, Philippines	***Solanumcyanocarphium***
–	Erect shrubs; leaves broadly elliptic to ovate; leaf bases truncate; berries whitish green when mature	**86**
86	Young growth densely prickly and bristly, the bristles long-stalked and some topped with stellate rays; inflorescences 3–10 cm long; corolla 1.8–2.2 cm in diameter. Indonesia, China to Indochina	***Solanumbarbisetum***
–	Young growth sparsely prickly, not bristly; inflorescences 1.5–3 cm long; corolla 0.7–1.5 cm in diameter. India to China and Indochina	***Solanumpraetermissum***
87	Flowers with the anthers slightly unequal, often 4-merous; sympodial units plurifoliate, the leaves not geminate. China, Indochina, Indonesia, Timor Leste	***Solanumprocumbens***
–	Flowers with anthers of equal size, 5-merous; sympodial units difoliate, the leaves geminate or not	**88**
88	Fruiting pedicels strongly reflexed; trichomes of stems and leaves with elongate midpoints to 2 mm long. Western Ghats, southern India	***Solanummultiflorum***
–	Fruiting pedicels erect or spreading; trichomes of stems and leaves with midpoints not markedly longer than the rays	**89**
89	Calyx lobes long-acuminate, strongly keeled; seeds > 100 per berry. Indonesia (except Borneo)	***Solanumpoka***
–	Calyx lobes deltate, not strongly keeled; seeds 10–20 per berry	**90**
90	Leaf bases cuneate to attenuate; upper leaf surfaces moderately to sparsely stellate-pubescent; fruiting pedicels strongly deflexed. Western Ghats, southern India	***Solanumhovei***
–	Leaf bases acute or truncate; upper leaf surfaces densely stellate-pubescent; fruiting pedicels spreading. Widespread throughout	***Solanumviolaceum***

##### ﻿Synoptic character list for tropical Asian spiny solanums

This synoptical character list can be used as a multi-entry key for identification. We have only listed diagnostic characters here, for example, entire and pinnatifid leaves are listed but not lobed leaves in general. For distributional information please see Table 2. The list is intended to be used as a tool via a process of elimination; any character can be selected and in combination with other characters, a smaller selection of species can be obtained, for which the descriptions will be useful for coming to a final identification.

- Plants found in cultivation (agricultural settings or home gardens): aethiopicum, macrocarpon, mammosum, melongena
- Scrambling or prostrate plants: cordatum, cyanocarphium, forskalii, miyakojimense, procumbens, schefferi, trilobatum, wightii
- Plants unarmed (no obvious prickles anywhere on plant): aethiopicum, camranhense, comitis, cordatum, deflexicarpum, dunalianum, giganteum, lasiocarpum, macrocarpon, melongena, miyakojimense, nienkui, peikuoense, pubescens, putii, retrorsum, robinsonii, torvoideum, torvum, vagum, violaceum, wrightii
- Plants drying black on herbarium specimens: giganteum, kachinense, poka, pseudosaponaceum
- Plants andromonoecious (with basal hermaphroditic and distal staminate flowers): arundo, barbisetum, insanum, lasiocarpum, macrocarpon, melongena, schefferi, virginianum, wightii
- Small trees, taller than 3 m: arundo, chrysotrichum, (comitis?), dunalianum, giganteum, peikuoense, pseudosaponaceum, torvoideum, wrightii
- Simple trichomes (never stellate) on stems: aculeatissimum, capsicoides, mammosum, viarum
- Stems white from dense pubescence of multangulate stellate trichomes: arundo, forskalii, giganteum
- Stems with strongly hooked prickles: cordatum, cyanocarphium, graciliflorum, jamaicense, procumbens, schefferi, trilobatum
- Stem prickles straight and strongly retrorse: retrorsum
- Rays of stellate trichomes fused at the base (lepidote or almost lepidote): elaeagnifolium, robinsonii
- Trichomes and/or prickles purple or purplish black in live plants (sometimes also visible on herbarium specimens): barbisetum, cyanocarphium, hovei, involucratum, lasiocarpum, praetermissum (insanum and melongena prickles purple only)
- Leaves on reproductive branches < 1.5 times longer than wide: camranhense, cordatum, forskalii, involucratum, lasiocarpum, miyakojimense, praetermissum, pubescens, sulawesi, trilobatum, virginianum, wightii
- Leaves on reproductive branches entire (without lobes): camranhense, comitis, cordatum, dunalianum, elaeagnifolium, forskalii, giganteum, miyakojimense, nienkui, poka, praetermissum, procumbens, pseudosaponaceum, pubescens, putii, retrorsum, robinsonii, schefferi, torvum, vagum
- Leaves on reproductive branches with secondary lobing (some lobing to pinnatisect): arundo, multiflorum, sisymbriifolium, violaceum, virginianum
- Leaves glabrous or glabrescent adaxially: aethiopicum, arundo, cordatum, giganteum, graciliflorum, kachinense, macrocarpon, melongena (rarely), peikuoense, retrorsum, trilobatum, vagum, virginianum
- Leaves with bright yellow, straight prickles > 1 cm long: aculeatissimum, arundo, capsicoides, sulawesi, virginianum
- Leaves with only simple trichomes adaxially: aculeatissimum, capsicoides, mammosum, viarum, wrightii
- Pubescence of adaxial leaf surfaces of multangulate trichomes with all rays pointing upwards: comitis
- Pubescence of white mealy multangulate trichomes that are easily rubbed off: giganteum, vagum
- Leaf bases decurrent onto the stem: macrocarpon, robustum
- Leaf bases cordate/truncate: camranhense, cordatum, forskalii, melongena, pubescens, wightii
- Inflorescence many times branched: dunalianum, giganteum, graciliflorum, harmandii, kachinense, multiflorum, peikuoense, pseudosaponaceum, schefferi, torvoideum
- Inflorescence glandular pubescent: aculeatissimum, pubescens, torvum, viarum
- Peduncles absent or very short (< 5 mm): cordatum, miyakojimense, sulawesi, torvoideum, violaceum, wightii
- Calyx lobes foliaceous: macrocarpon, melongena, robustum
- Calyx lobes with a distinct acumen: camranhense, peikuoense, poka, robinsonii, torvoideum, vagum, virginianum
- Corolla stellate-rotate or pentagonal (with abundant interpetalar tissue): comitis, insanum, macrocarpon, melongena, virginianum, wightii
- Corolla deeply stellate (lobed nearly to the base): barbisetum, graciliflorum, kachinense, retrorsum, schefferi, vagum
- Anthers unequal (even slightly so): nienkui, procumbens, pubescens, putii, robinsonii, vagum, wightii
- Anthers with several markedly different in size and shape, strongly curved: pubescens, vagum, wightii
- Calyx strongly accrescent in fruit, more than half covering the berry: barbisetum, cyanocarphium, involucratum, macrocarpon, praetermissum, wightii
- Immature berries marbled green and white: aculeatissimum, arundo, insanum, jamaicense, miyakojimense, viarum, virginianum
- Mature berry > 3 cm in diameter: aethiopicum, arundo, lasiocarpum, macrocarpon, mammosum, melongena, schefferi, wrightii
- Mature berries not strictly globose: hovei, macrocarpon, mammosum, melongena, miyakojimense, schefferi
- Mature berries red or orange, thin-skinned and fleshy (usually juicy): camranhense, cordatum, deflexicarpum, dunalianum, forskalii, giganteum, hovei, involucratum, jamaicense, lasiocarpum, miyakojimense, multiflorum, nienkui, peikuoense, procumbens, pseudosaponaceum, pubescens, retrorsum, robinsonii, sisymbriifolium, trilobatum, vagum, violaceum
- Mature berries bright yellow when ripe: aculeatissimum, arundo, insanum, violaceum, virginianum
- Mature berries greenish yellow or yellowish green when ripe: chrysotrichum, insanum, melongena, poka, torvoideum, torvum, torvoideum, wightii (just before dehiscence)
- Mature berries white or greenish white when ripe: barbisetum, praetermissum
- Mature berries black or purple when ripe: melongena, peikuoense, torvoideum
- Mature berries leathery: aculeatissimum, arundo, capsicoides, chrysotrichum, elaeagnifolium, insanum, macrocarpon, mammosum, melongena, viarum, virginianum, wrightii
- Mature berry dry, pseudo-capsular or the pericarp shattering: elaeagnifolium, wightii
- Mature berries densely and uniformly pubescent: involucratum, lasiocarpum, robustum, sulawesi
- Inner flesh of berry spongy and not at all juicy: capsicoides, mammosum, melongena
- Seeds > 100 per berry: cyanocarphium, insanum, involucratum, lasiocarpum, poka, pseudosaponaceum, schefferi, sulawesi, torvoideum, virginianum, wrightii
- Seeds 10 or fewer per berry: camranhense, cordatum, forskalii, graciliflorum, hovei, multiflorum, nienkui, procumbens, retrorsum, robinsonii, vagum, violaceum
- Seeds dark brown or blackish brown: cordatum, forskalii
- Seeds with a distinct wing: capsicoides

##### ﻿Species descriptions

### ﻿1. Solanum aculeatissimum

Taxon classificationPlantaeSolanalesSolanaceae

Jacq., Icon. Pl. Rar. 1: 5, t. 41. 1781.

3A463B47-B10F-5654-B5DB-D0FE57F905B3

**Figure 5.**
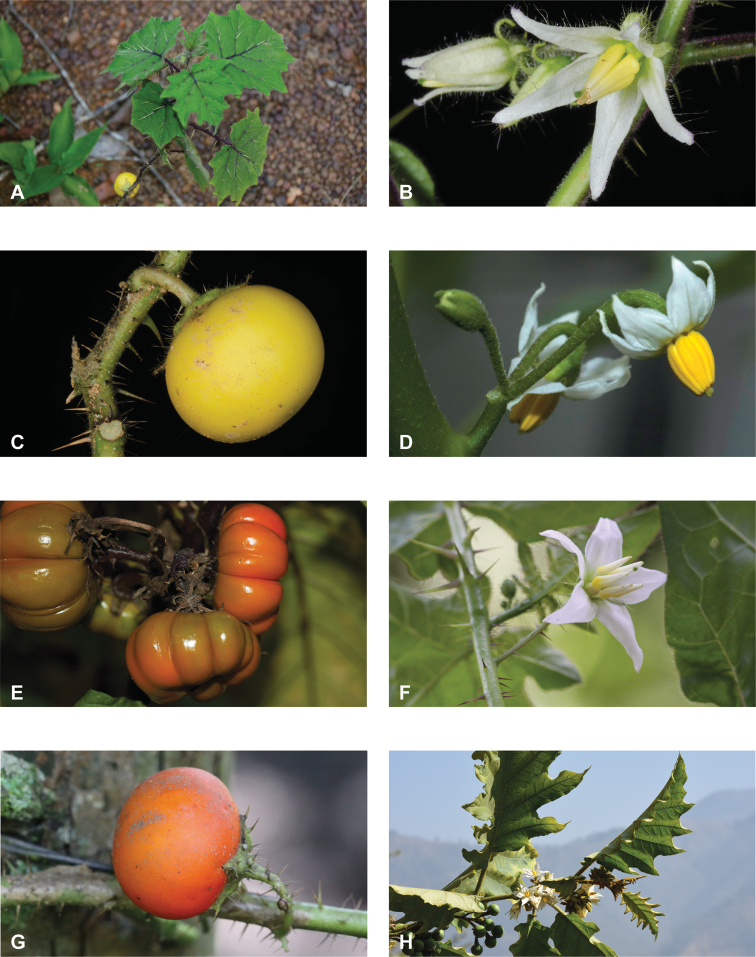
Introduced species of *Solanum*. *Solanumaculeatissimum* Jacq. **A** habit (*Bidault et al. 3627*, Gabon) **B** detail view of a flower (*Bidault et al. 3627*, Gabon) **C** detail view of a fruit (*Bidault et al. 3627*, Gabon). *Solanumaethiopicum* L. **D** detail view of a flower (field photograph, unvouchered, in cultivation at Radboud University, Nijmegen) **E** detail view of fruits (field photograph, unvouchered, in cultivation at the Max Planck Institute for Plant Breeding Research, Cologne). *Solanumcapsicoides* All. **F** detail view of a flower (*Coronado González 5457*, Nicaragua) **G** detail view of a fruit (*Coronado González 5457*, Nicaragua). *Solanumchrysotrichum* Schltdl. **H** detail of a fertile branch (field photograph, unvouchered, India). Photograph credits: **A–C** E. Bidault **D, E, H** S. Knapp **F, G** I. Coronado González.

Fig. 5A–C Only names based on tropical Asian types listed here; for complete synonymy see Nee 1979 ; Vorontsova and Knapp 2016.

Solanum khasianum C.B.Clarke, Fl. Brit. Ind. [J.D. Hooker] 4: 234. 1883. Type. India. Meghalaya [“Assam”]: Khasia Hills, *J. D. Hooker & T. Thomson [1666] 14* (lectotype, designated by Sen Gupta 1961, pg. 411: CAL; isolectotypes: GH [00077827], K [K000546077], L [L 0003639], W [acc. # 0022656]).
Solanum cavaleriei H.Lév. & Vaniot, Bull. Soc. Bot. France 55: 207. 1908. Type. China. Guizhou: “Ly-Po-Hien”, 10 Aug 1899, *J. Cavalerie 2722* (lectotype, designated here: E [E00284478]).

#### Type.

Cultivated in Vienna, Austria, of unknown origin, *Anonymous s.n.* (lectotype, designated by Vorontsova and Knapp 2016, pg. 41: W [acc. # 0022470]).

#### Description.

Vorontsova and Knapp (2016: 41–45); http://www.solanaceaesource.org/solanaceae/solanum-aculeatissimum.

#### Distribution.

*Solanumaculeatissimum* occurs only sporadically in tropical Asia (see Table 2); it is native to Brazil (Nee 1979) and adventive and widely distributed in Africa, it is less common in tropical Asia than the similar *S.viarum*.

#### Common names.

China. ka xi qie (Zhang et al. 1994). Malaysia/Singapore. tĕrong perat, tĕrong asam hutan, tĕrong puyoh, tĕrong bĕlanda, tĕrong pusat, tĕrong piat (Burkill 1935), tèrong kori, tèrong tènang (Sundanese, Burkill 1935). Vietnam. cà ung (Hul and Dy Phon 2014). Common names from Burkill (1935) may also refer to *S.viarum*.

#### Discussion.

*Solanumaculeatissimum* is a member of the Acanthophora clade (sensu Stern et al. 2011; Gagnon et al. 2022) as are several other introduced species in tropical Asia (*S.capsicoides*, *S.mammosum*, *S.viarum*) and is most similar to *S.viarum*. It can be distinguished from that species in its long-acuminate (versus deltate) calyx lobes and its minutely stipitate-glandular (versus puberulent) ovary. *Solanumviarum* is more finely and evenly glandular pubescent than is *S.aculeatissimum*.

The herbarium of the French botanist and clergyman Augustin A.H. Léveillé was acquired by the Scottish botanist George Forrest from whence it passed to the Royal Botanic Garden Edinburgh. Vorontsova and Knapp (2016) incorrectly cited a collection in E as the holotype, no herbarium was cited in the protologue. We have selected the specimen at E (E00284478) that corresponds to the description, collector, and locality (Léveillé 1908), as the lectotype for *S.cavaleriei*.

#### Specimens examined.

See Suppl. materials 1–3.

### ﻿2. Solanum aethiopicum

Taxon classificationPlantaeSolanalesSolanaceae

L., Cent. Pl. II 10. 1756.

89D789A6-9A3A-55A0-800F-EC4629508DF2

Fig. 5D, E No names based on tropical Asian types have been published; for complete synonymy see Vorontsova and Knapp 2016.

#### Type.

Ethiopia. Sin. loc., *J. Burser vol. 9 no. 17* (lectotype, designated by Hepper and Jaeger 1985, pg. 391: UPS).

#### Description.

Vorontsova and Knapp (2016: 49–56); http://www.solanaceaesource.org/solanaceae/solanum-aethiopicum-0.

#### Distribution.

We have recorded *S.aethiopicum* from China and India, it is recorded from Sri Lanka (as *S.integrifolium* Poir.) by Hepper (1987); it is a plant from tropical sub-Saharan Africa (native) but is not known in the wild; widely cultivated in South America, Europe, and Asia.

#### Common names.

China. hong qie (Zhang et al. 1994).

#### Discussion.

*Solanumaethiopicum* is the scarlet eggplant or gilo. It is sporadically cultivated for its fruits (and possibly leaves) in southwestern China and adjacent India for its edible fruits. Cultivars from tropical Asia are usually glabrescent, with large, ribbed and sometimes flattened berries. It can be easily distinguished from the cultivated brinjal eggplant *S.melongena* by its smaller white flowers and red or reddish orange mature berries. It could possibly be confused with the widespread *S.violaceum* (that is somewhat similar to *S.anguivi* Lam., the wild progenitor of *S.aethiopicum*, see Vorontsova and Knapp 2016), but is more glabrescent than that species, the berries are much larger, and the fruiting pedicels are not as strongly spreading.

#### Specimens examined.

See Suppl. materials 1–3.

### ﻿3. Solanum arundo

Taxon classificationPlantaeSolanalesSolanaceae

Mattei, Boll. Reale Orto Bot. Palermo 7: 188. 1908.

40C72A1F-BEF9-5E1A-883A-80AF022B4A5F

Fig. 6

Solanum diplacanthum Dammer, Bot. Jahbr. Syst. 48: 245. 1912. Type. Tanzania. Sin. loc., *Dr. Fischer s.n.* (type: B?, destroyed, no duplicates found).
Solanum helleri Standl., Smithsonian Misc. Collect. 68, no. 5: 15. 1917. Type. Kenya. Rift Valley: Northern Frontier: Ewaso Ngiro River, Neumann’s Camp, 26 Sep 1911, *E. Heller s.n.* (holotype: US [00027596, acc. # 634351]).

#### Type.

Somalia. Banaadir: Mogadischo, 30 May 1913, *G. Paoli 137* (neotype, designated by Vorontsova and Knapp 2016, pg. 74: FT [FT003945]; isoneotype: FT [FT003946]).

#### Description.

Erect shrub to small tree, 2–6 m, densely prickly. Young stems erect, robust, densely stellate-pubescent and densely prickly, with porrect to multangulate, variously stalked trichomes, the stalks to 0.15 mm long, the rays 7–8(–15), 0.05–0.2 mm long, the midpoints reduced or absent, the prickles 5–10 mm long, 2.5–8 mm wide at the base, strongly curved, flattened, pale yellow to orange, densely stellate-pubescent in the lower 1/3; bark of older stems glabrescent to moderately stellate-pubescent, reddish brown. Sympodial units plurifoliate. Leaves simple, lobed, the blades 2–4(–7) cm long, 1.5–2.5(–3.5) cm wide, 1.5–2 times longer than wide, elliptic, concolorous, armed with 2–5 acicular, straight prickles to 12 mm long on both surfaces; adaxial surface glabrescent, with trichomes only at the base or along the midvein; abaxial surface glabrescent to sparsely stellate-pubescent with porrect, sessile or stalked trichomes, the stalks to 0.2 mm long, the rays 7–9, 0.1–0.2 mm long, the midpoints to 0.3 mm long; principal veins 3–5 pairs, further venation not visible or faint; base cuneate to rounded; margins lobed, the lobes 2–4 on each side, 0.2–0.7(–1.5) cm long, deltate to ovate, sometimes with secondary lobing, apically rounded, sometimes obtuse, the sinuses extending 1/4–2/3 of the distance to the midvein; apex broadly acute to rounded; petiole 0.05–0.5 cm long, less than 1/5 of the leaf blade length, densely stellate-pubescent, unarmed or with a few prickles. Inflorescences apparently lateral, 2–3(–4) cm long, unbranched or forked, with 2–8 flowers; 1–3 flowers open at any one time, densely stellate-pubescent, with 0–2 prickles; peduncle 0–4 mm long; pedicels 0.45–1 cm long, erect, articulated at the base, densely stellate-pubescent, unarmed; pedicel scars spaced 1–4 mm apart. Flowers 5-merous, heterostylous and the plants andromonoecious, with the lowermost 1–2 flowers long-styled and hermaphrodite, the distal flowers short-styled and staminate. Calyx 5.5–9 mm long, the lobes 3–4 mm long, deltate, apically acute to acuminate, sparsely stellate-pubescent, unarmed or with a few prickles. Corolla 2–3.2 cm in diameter, mauve to purple, stellate, lobed for ca. 4/5 of its length, the lobes 7–14 mm long, 3.5–4.5 mm wide, narrowly deltate, spreading, densely stellate-pubescent abaxially, the trichomes porrect, sessile or stalked, the stalks to 0.2 mm, the rays 6–9, 0.2–0.3 mm long, the midpoints reduced or to 0.5 mm long. Stamens equal; filament tube ca. 1.5 mm long; free portion of the filaments 0.5–1.5 mm long; anthers ca. 5.5 mm long, yellow, connivent, tapering, poricidal at the tips, the pores not lengthening to slits with age. Ovary densely stellate-pubescent in the upper 1/3; style 13–17 mm long in long-styled flowers, stout, curved, weakly stellate-pubescent in the lower 1/3, ca. 2 mm long in short-styled flowers; stigma clavate, minutely papillate. Fruit a globose berry, 1–2 per infructescence, 1.8–3 cm in diameter, the pericarp smooth, dark green with pale green and cream markings when young, yellow at maturity; fruiting pedicels 0.8–1.5 cm long, 1.5–2 mm in diameter at the base, woody, pendulous, usually unarmed but occasionally with a few prickles; fruiting calyx not markedly accrescent, but elongating to 7–12 mm long, somewhat fleshy and covering ca. 1/4 of the mature fruit (reflexed in herbarium sheets and on old fruits), unarmed or with up to 10 prickles. Seeds ca. 20–40 per berry, 2.2–3.2 mm long, 1.8–2.2 mm wide, flattened-reniform, dull yellow to orangish brown, the surfaces minutely pitted, the testal cells sinuate in outline. Chromosome number: not known.

**Figure 6.**
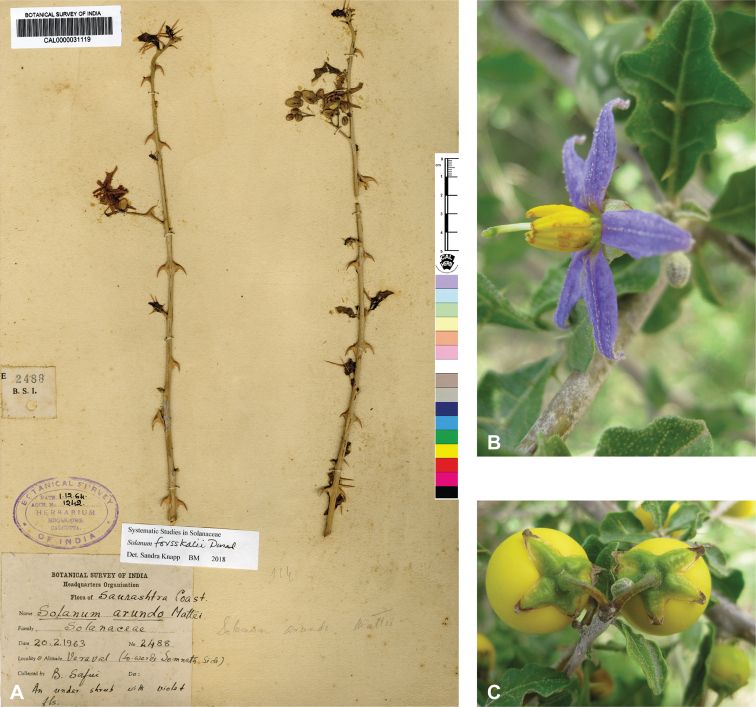
*Solanumarundo* Mattei **A** herbarium specimen collected in India in 1963 (*Safni CNH-2488*) **B** detail view of flower (*Vorontsova et al. 80*, Kenya) **C** detail view of fruit (*Vorontsova et al. 80*, Kenya). Photograph credits: **A** © The Director, Botanical Survey of India, Kolkata **B, C** M.S. Vorontsova.

#### Distribution

**Figure 7.**
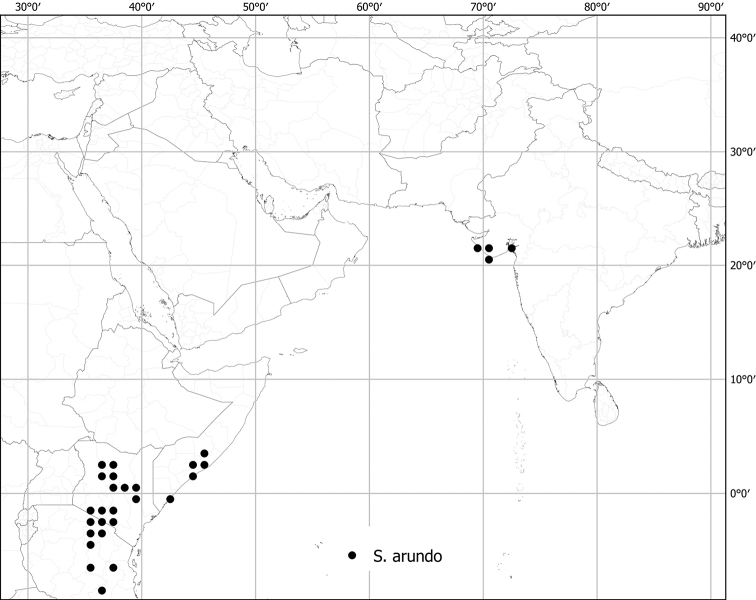
Distribution of *S.arundo*.

**(Fig. 7).***Solanumarundo* is an African species, found primarily in eastern Africa from Somalia to Tanzania, in tropical Asia it occurs only in far western India on the Kathiawar peninsula in the state of Gujarat.

#### Ecology and habitat.

*Solanumarundo* is a plant of grasslands and savannahs in Africa (Vorontsova and Knapp 2016); in India it is found in seaside vegetation (Sen Gupta 1963).

#### Common names and uses.

There have been no common names or uses recorded for *S.arundo*, but in Africa it is often used as a hedge plant, due to its ability to form thick impenetrable thickets (Vorontsova and Knapp 2016).

#### Preliminary conservation status

**(IUCN 2019).** Least Concern (LC); EOO (1,277,240 km^2^), AOO (216 km^2^). *Solanumarundo* has a broad distribution in Africa, and although it is relatively poorly represented in India, it is not of particular conservation concern based on its worldwide distribution. The distribution and occurrence of *S.arundo* on the Kathiawar peninsula need to be confirmed with modern collections.

#### Discussion.

*Solanumarundo* is a distinctive small extremely prickly tree, in Africa it is often associated with human habitation (Vorontsova and Knapp 2016). It is easy to distinguish from other spiny solanums in western India by the combination of thick, curved, and flattened prickles on the densely stellate- or multangulate-pubescent stems, and long, straight prickles on the leaves. *Solanumforskalii* is sympatric with *S.arundo* and has similar white-pubescent stems, but it is a smaller, scandent plant, with truncate to cordate rather than cuneate leaf bases, smaller corollas (1.3–2.4 versus 2–3.2 cm in diameter) and much smaller fruits (0.6–0.9 versus 1.8–3 cm in diameter). In 2018, one of us (SK) erroneously identified the specimens of *S.arundo* in CAL as *S.forskalii* (Fig. 6).

Vorontsova et al. (2013) resolved *S.arundo* as sister to the more narrowly distributed African endemic *S.dennekense* Dammer, a similar treelet with curved stem spines, white stems, and large, leathery berries.

Sen Gupta (1963) suggested that *S.arundo* had arrived in India with Arab traders in the nineteenth century, as part of the extensive Gujarati trade routes established across the Arabian Sea. Its restricted distribution on the Kathiawar peninsula certainly supports this idea. *Solanumarundo* has not been collected, to our knowledge, since the 1960s, re-evaluation of its presence and distribution in western India is necessary.

#### Specimens examined.

See Suppl. materials 1–3.

### ﻿4. Solanum barbisetum

Taxon classificationPlantaeSolanalesSolanaceae

Nees, Trans. Linn. Soc. London 17(1): 51 1834.

4CE047C0-7A92-5ACC-A923-53A82F8B75F6

Figs 2H , 8

Solanum eriophorum Dunal, Prodr. [A. P. de Candolle] 13(1): 249. 1852. Type. Myanmar. Tanintharyi: “Tavoy” [Dawei], 12 Aug 1827, *N. Wallich s.n.* [Wallich Catal. Burm. 1328] (holotype: G-DC [G00145974]).

#### Type.

Myanmar. Tanintharyi Region: “Tavoy” [Dawei], Aug 1827, *N. Gómez 9071* (lectotype, designated by Hul and Dy Phon 2014, pg. 34: K [K000759381]; isolectotypes: GZU [GZU000255503], LE).

#### Description.

Herbs to small shrubs to 1 m tall, heavily armed. Stems erect, terete, densely prickly and pubescent, the pubescence deciduous with age leaving only prickles and bristles on older stems; prickles of varying sizes to 0.5 cm long, broad-based, straight, yellowish tan in dry material, purplish black or yellowish tan in live plants, grading into long-stalked bristles and stellate trichomes; pubescence of porrect-stellate trichomes, mixture of sessile, short- and long-stalked, often recorded as purple-tinged or blackish red, the stalks multiseriate, to 3 mm long, the rays 4–6, 0.5–1 mm long, thin and brittle, the midpoints absent on stalked trichomes, the sessile trichomes with elongate midpoints to 2 mm long, always longer than the rays; new growth densely pubescent with mixture of short- and long-stalked stellate trichomes; bark of older stems greyish brown. Sympodial units difoliate, the leaves not geminate, or occasionally unifoliate in very small plants. Leaves simple, shallowly lobed, the blades (7–)12–30 cm long, (5–)10–17 cm wide, 1.5–1.7 times longer than wide, broadly elliptic to somewhat ovate, chartaceous, discolorous (paler green beneath fide *Maxwell 91-651*), sparsely armed along the midrib and major veins with prickles to 1 cm long; adaxial surface dark green, sparsely pubescent with sessile porrect-stellate trichomes, the rays 0–4, 0.1–0.5 mm long, the midpoints 2–3 mm long, delicate and thin; abaxial surface with similar sessile porrect-stellate trichomes, but these denser, especially along the veins; major veins 4–7 pairs, densely pubescent especially abaxially, in live plants often purple-tinged; base abruptly truncate, somewhat oblique; margins shallowly lobed, the lobes 4–7 on each side, occasionally somewhat secondarily lobed, to 1 cm long, more or less broadly deltate, apically acute, the sinuses less than halfway to the midrib; apex acute; petioles 3–9 cm long, half as long to almost as long as the leaf blades, prickly and sparsely pubescent, the prickles to 1 cm long, straight, grading into bristles that themselves grade into to a few long-stalked stellate trichomes, the pubescence largely of sessile stellate trichomes with 5–6 rays 0.5–1 mm long and midpoints to 2 mm, always longer than the rays. Inflorescences (1–)3–10 cm long, internodal and lateral, unbranched, with 10–40 flowers, few flowers open at any one time, densely bristly and pubescent with trichomes like those of the stems, the bristles sparser distally with early deciduous rays at the apex grading into prickles; peduncle 0.5–2 cm long, unarmed or armed with a few prickles; pedicels 0.5–0.8 cm long, 0.5–0.7 mm in diameter at the base and apex, slender and nodding at anthesis, densely bristly and stellate-pubescent like the inflorescence axes, articulated at the base; pedicel scars irregularly spaced 3–4 mm apart. Buds long-tapering and somewhat fusiform, almost completely enclosed in the foliaceous calyx. Flowers 5-merous, heterostylous and the plants weakly andromonoecious, with most flowers long-styled and only the most distal short-styled, these drop after flowering and the whip-like inflorescence axis tip persists in fruit. Calyx with the tube 2.5–5.5 mm long, deeply cupulate to somewhat urceolate, densely bristly with bristles to 5 mm long, these elongating in fruit, the lobes 5–8 mm long, 3–4 mm wide, lanceolate to broadly triangular, densely stellate-pubescent abaxially with sessile porrect-stellate trichomes with 4–6 weak rays and an elongate midpoint. Corolla 1.8–2.2 cm in diameter, white or violet (usually adaxially white and abaxially violet, but not always), deeply stellate, lobed nearly to the base, interpetalar tissue absent, the lobes 8–10 mm long, 1.5–3 mm wide, narrowly deltate, spreading to recurved at anthesis, glabrous adaxially, densely stellate-pubescent abaxially with sessile trichomes with elongate midpoints, these densest at the tips. Stamens slightly unequal, with 2 slightly longer than the rest; anthers 8.5–10 mm long, 2 slightly longer, ca. 1 mm wide, strongly tapering, yellow, glabrous, poricidal at the tips, the pores directed distally, not elongating to slits with drying; filament tube minute, glabrous; free portion of the filaments minute, glabrous, the anthers almost sessile. Ovary conical, glabrous; style (long-styled flowers) 8–10 mm long, glabrous, ca. 3 mm long in short-styled flowers; stigma capitate, the surfaces minutely papillose, bright green in live plants. Fruit a globose berry, 6–12 per infructescence, 1–1.4 cm in diameter, pale greenish white, completely enclosed in the bristly calyx tube, the pericarp thin and more or less shiny, glabrous; fruiting pedicels 0.7–0.9 cm long, ca. 1 mm in diameter at the base, 1.7–2 mm in diameter at the apex, somewhat woody, spreading to pendent; fruiting calyx greatly expanded to enclose the berry, the tube densely bristly and surrounding the berry, the lobes to 1 cm long, 0.3 cm wide, stellate-pubescent. Seeds 20–30 per berry, 2.5–5 mm long, (1.5)2–3 mm wide, flattened reniform, pale tan or yellowish brown (white in live plants), the surfaces minutely pitted, the testal cells pentagonal in shape. Chromosome number: not known.

**Figure 8.**
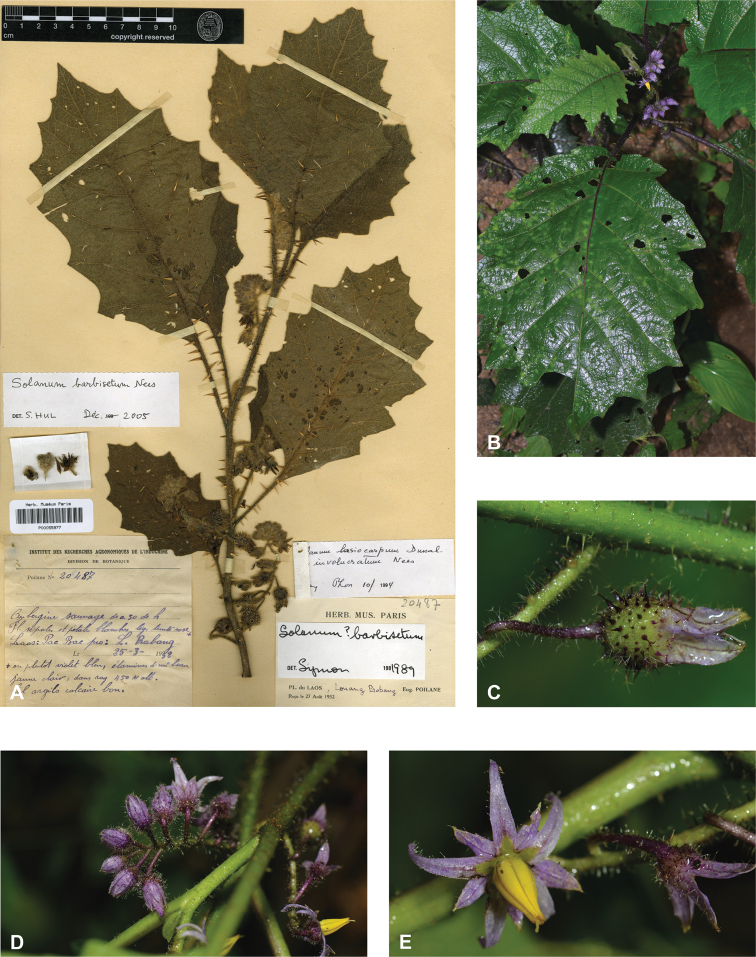
*Solanumbarbisetum* Nees **A** herbarium specimen collected in Laos in 1932 (*Poilane 20487*, P00055977) **B** habit and leaves (*Suksathan et al. PS 3832*, Thailand) **C** detail of the calyx with stellate trichomes and bristles (*Suksathan et al. PS 3832*, Thailand) **D** many flowered inflorescence (*Suksathan et al. PS 3832*, Thailand) **E** detail view of a flower (*Suksathan et al. PS 3832*, Thailand). Photograph credits: **A** CC-BY, Muséum national d’Histoire naturelle, Paris **B–E** D. Pedersen.

#### Distribution

**Figure 9.**
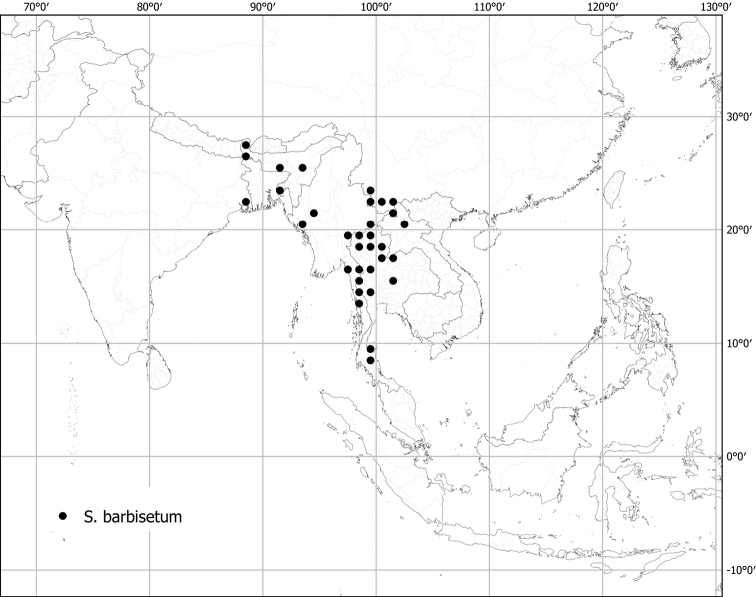
Distribution of *S.barbisetum*.

**(Fig. 9).***Solanumbarbisetum* occurs from southern China (Yunnan) and northeastern India to Laos and Thailand.

#### Ecology and habitat.

*Solanumbarbisetum* grows in open areas, in fields and along forest edges in mixed broadleaf forests; usually from 300 to 2,000 m elevation, more rarely at sea level.

#### Common names and uses.

China. ci bao qie (Zhang et al. 1994). Thailand. Nakhon Si Thammarat: ma uk pa (*Rabil Bunnag 204*); North: ma khûa chê pâ [Lao] (*Winit Wanadorn 1424*).

Fruits (ripe berries) said to be eaten in India (Jain and Borthakur 1986).

#### Preliminary conservation status

**(IUCN 2019).** Least Concern (LC); EOO (1,244,077 km^2^), AOO (248 km^2^). *Solanumbarbisetum* is widely distributed and is a plant of disturbed areas such as field and forest edges; the small AOO certainly reflects collection bias.

#### Discussion.

*Solanumbarbisetum* is a distinctive species with its elongate inflorescences, berries enclosed in an accrescent calyx and copious long-stalked stellate trichomes on all vegetative parts. It is morphologically most similar to *S.praetermissum* and to a lesser extent *S.involucratum*; it shares with both those species berries enclosed in accrescent calyces and large leaves. *Solanuminvolucratum* is not sympatric with *S.barbisetum* and is a more robust plant with pubescent berries and shorter inflorescences. *Solanumpraetermissum* is more or less sympatric with *S.barbisetum* and has been treated as a variety of *S.barbisetum* in the past (Clarke 1883, see description of *S.praetermissum*). *Solanumbarbisetum* differs from *S.praetermissum* in its bristlier pubescence, with the calyx trichomes becoming stiff and prickly as the fruit matures and its more attenuate leaf bases. The calyx pubescence of *S.barbisetum* is often purple-tinged; this is even visible in quite old herbarium sheets.

*Solanumbarbisetum* was treated under this species name for Bhutan in Mill (2001), but with the note that “Most specimens from our area labelled ‘*S.barbisetum*’ belong, or appear to belong to *S.griffithii*” (Mill 2001: 1059). The correct name for *S.griffithii* (Prain) Wu & Huang is *S.praetermissum* (see description); care should be taken with the identification of material from Bhutan for these taxa.

The phylogenetic affinities of *S.barbisetum* are unclear. In the analyses of Aubriot et al. (2016a) it is resolved as sister to the ‘S.expedunculatum and relatives’ clade (*S.expedunculatum* Symon, *S.heteracanthum* Merr. & L.M.Perry, *S.involucratum* and *S.procumbens*) but this is only supported with Bayesian inference. It might also be closely related to the morphologically similar *S.praetermissum* but that relationship is not clearly supported in molecular analyses (Vorontsova et al. 2013; Aubriot et al. 2016a).

#### Specimens examined.

See Suppl. materials 1–3.

### ﻿5. Solanum camranhense

Taxon classificationPlantaeSolanalesSolanaceae

Dy Phon & Hul, Fl. Cambodge, Laos & Vietnam 35: 16. 2014.

D811B33B-40D1-5622-8293-B5186B3489F4

Figs 1E , 10

#### Type.

Vietnam. Khánh Hòa: “Province Nha Trang, dunes littorales de Cam Ranh, My La face à la lagune de Bau Ro”, 7 Mar 1961, *Lê Công Kiêt 94* (holotype: P [P00055921]; isotype: P [P00055922]).

#### Description.

Scandent shrubs, to 1.5 m tall, unarmed or sparsely prickly. Stems more or less erect, terete, unarmed or occasionally with a few scattered tiny prickles, sparsely to densely stellate-pubescent; prickles to 1.25 mm long, to 1 mm in diameter at the base, straight or curved, acicular to deltate, orange, glabrous; pubescence of mixed sessile and very short-stalked porrect-stellate trichomes, the stalks to 0.1 mm long, the rays 6–9, 0.1–0.4 mm long, the midpoints absent or up to 0.1 mm long; new growth densely stellate-pubescent, light brownish in dry material; bark of older stems brownish grey, glabrescent. Sympodial units plurifoliate, the leaves not geminate. Leaves simple, not lobed, the blades 2–3.5 cm long, 1.5–2.5 cm wide, ca. 1–1.5 times longer than wide, broadly ovate to suborbicular, chartaceous, slightly discolorous, unarmed; adaxial surface densely stellate-pubescent, the stellate trichomes porrect, sessile to stalked, the stalks to 0.1 mm, the rays 6–8, 0.1–0.4 mm long, the midpoints to 0.1 mm; abaxial surface densely stellate-pubescent with trichomes like those of the adaxial surface; major veins 3–5 pairs, drying yellowish light-green; base truncate to subcordate; margins entire or shallowly sinuate; apex obtuse; petioles 0.4–1 cm long, 1/5–1/3 of the leaf blade length, unarmed and densely stellate-pubescent, the pubescence of sessile and short-stalked stellate-trichomes like those of the blades. Inflorescences 1.5–2 cm long, internodal and lateral, sometimes appearing terminal, unbranched, with ca. 4–7 flowers, only 1 or 2 flowers open at any one time, densely stellate-pubescent, with a mix of sessile and short-stalked porrect trichomes like those of the stems, unarmed; peduncle 0.5–2 cm long, unarmed; pedicels 5–9 mm long, ca. 0.5 mm in diameter at the base, ca. 1 mm in diameter at the apex, spreading to erect, unarmed, densely stellate-pubescent with porrect trichomes like those of the inflorescence axes, articulated at the base; pedicel scars irregularly spaced 0.5–4 mm apart. Buds elongate ellipsoid, more or less strongly exserted from the calyx before anthesis. Flowers 5-merous, apparently all perfect. Calyx with the tube ca. 3 mm long, campanulate, the lobes 2–3 mm long, ca. 1 mm wide, deltate and constricting to a short acumen, the acumen 1/4–1/3 the total lobe length, unarmed and densely stellate-pubescent abaxially with porrect-stellate trichomes like those of the pedicels. Corolla 0.8–1 cm in diameter, blue to light purple, stellate, lobed ca. 3/4 of the way to the base, the lobes 5–7 mm long, 1.5–2 mm wide, deltate, spreading at anthesis, densely stellate-pubescent abaxially on parts exposed in bud. Stamens equal; anthers 4–6 mm long, ca. 0.75 mm wide, tapering, orange, glabrous, poricidal at the tips, the pores not lengthening to slits with age; filament tube <0.5 mm long, glabrous; free portion of the filaments 0.5–1 mm long, glabrous. Ovary globose, with minute glandular hairs; style 7–8.5 mm long, slender, curved at the apex, glabrous; stigma capitate, minutely papillate. Fruit a globose berry, several per infructescence, 0.5–0.8 cm in diameter, the pericarp thin and smooth, red when mature, glabrous; fruiting pedicels 0.7–1 cm long, 0.5–1 mm in diameter at the base, 1.6–2 mm in diameter at the apex, woody, erect to spreading, straight to slightly deflexed, unarmed; fruiting calyx lobes not expanding, 1/2–2/3 the length of the mature fruit, broadly deltate, reflexed, unarmed. Seeds 8–10 per berry, 2.5–3 mm long, 2–2.5 mm wide, flattened-reniform, dull yellow, the surface minutely pitted, the testal cells pentagonal in outline. Chromosome number: not known.

**Figure 10.**
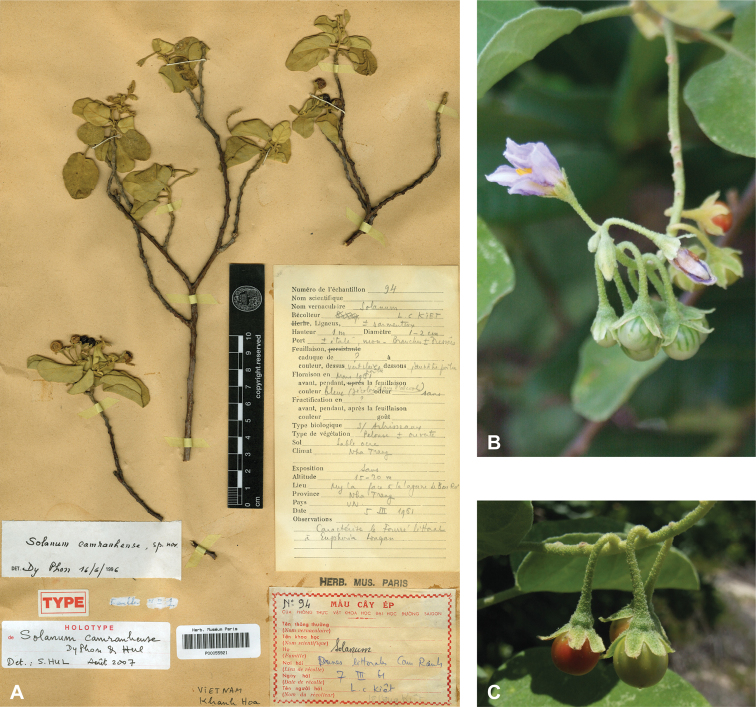
*Solanumcamranhense* Dy Phon & Hul **A** herbarium specimen (holotype) collected in Vietnam in 1961 (*Lê Công Kiêt 94*, P00055921) **B** inflorescence (field photograph, unvouchered, Vietnam) **C** detail view of the fruits (field photograph, unvouchered, Vietnam). Photograph credits: **A** CC-BY, Muséum national d’Histoire naturelle, Paris **B, C** S. Hul.

#### Distribution

**Figure 11.**
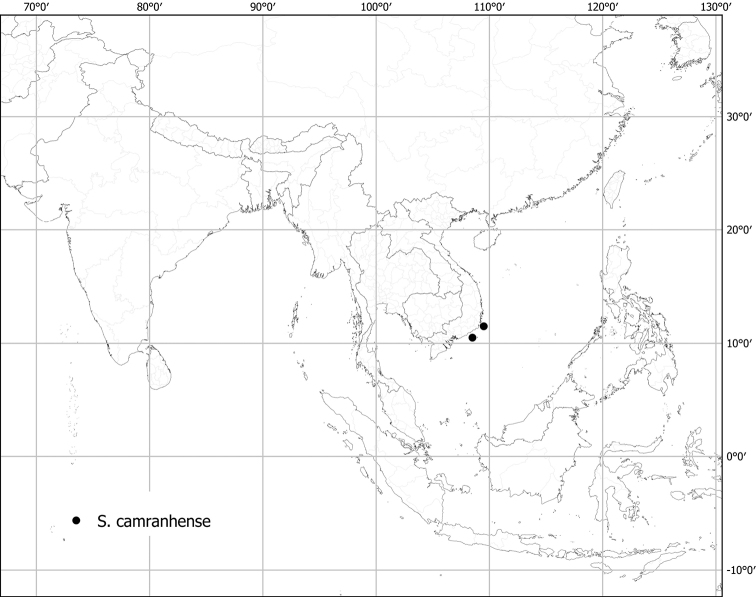
Distribution of *S.camranhense*.

**(Fig. 11).***Solanumcamranhense* is endemic to Vietnam; the few known collections are restricted to Khánh Hòa and Bình Thuận provinces of South Vietnam.

#### Ecology and habitat.

*Solanumcamranhense* has only been collected on coastal dunes of stabilized red sands; 15–20 m elevation.

#### Common names and uses.

Vietnam. Khánh Hòa: củ vè [Vietnamese] (*Chevalier 38932*).

#### Preliminary conservation status

**(IUCN 2019).** Endangered (EN [B2ab(i,ii,iii)]); EOO (555 km^2^), AOO 16 km^2^). Due to the paucity of collections, it is difficult to document the range of *Solanumcamranhense* with confidence. However, its occurrence in the fragmented and anthropogenically disturbed habitats of coastal southern Vietnam (Wikramanayake et al. 2002) suggests it is of conservation concern.

#### Discussion.

*Solanumcamranhense* is morphologically similar to the sympatric *S.robinsonii*, but differs from that species in its scandent (versus erect) habit, the denser pubescence that dries yellowish brown, shorter leaves (2–3.5 cm long versus 3–8 cm long in *S.robinsonii*), and smaller, more deeply lobed corollas (usually less than 1 cm in diameter and lobed 3/4 of the way to the base versus 1–2 cm in diameter and only lobed halfway to the base in *S.robinsonii*). Hul and Dy Phon (2014) described *S.camranhense* as unarmed, but we have seen specimens with a few scattered prickles on the scandent stems. The two species differ in habitat; *S.camranhense* is a plant of coastal dunes, while *S.robinsonii* occurs in coastal forests.

*Solanumcamranhense* is a member of the clade ‘*S.camranhense and relatives*’ of Aubriot et al. (2016a) with *S.nienkui* and *S.putii*; *S.robinsonii* has not been included in any molecular analyses to date, but we expect it to be closely related to these taxa.

#### Specimens examined.

See Suppl. materials 1–3.

### ﻿6. Solanum capsicoides

Taxon classificationPlantaeSolanalesSolanaceae

All., Auct. Syn. Meth. Stirp. Hort. Regii Taur. 64. 1773.

8B2547B1-43B5-5C25-96C3-65BE5B99053F

Fig. 5E–G Only names based on tropical Asian types listed here; for complete synonymy see Nee 1979 ; Vorontsova and Knapp 2016.

Solanum pentapetaloides Roxb. ex Hornem., Suppl. Hort. Bot. Hafn. 27. 1819. Type. Cultivated in Calcutta (?), *W. Roxburgh s.n.* (lectotype, designated by D’Arcy 1974b, pg. 842, as “type”: P-LA [P00357622]).
Solanum aculeatissimum Jacq. var. denudatum Dunal, Prodr. [A.P. de Candolle] 13(1): 244. 1852. Type. Indonesia. Java: sin. loc., *H. Zollinger 529* (lectotype, designated by Vorontsova and Knapp 2016, pg. 108: G-DC [G00145991, IDC microfiche 800-61.2080:II.6]; isolectotype: G).
Solanum bodinieri H.Lév. & Vaniot, Bull. Soc. Bot. France, 55: 206. 1908. Type. China. Hong Kong: Île Verte, 31 Jul 1895, *E.M. Bodinier s.n.* (lectotype designated here: E [E00284479]; isolectotype: A [00077790, fragment]).
Solanum xanthocarpum Schrad. & J.C.Wendl. var. geoffrayi Bonati, Fl. Indochine 4: 324. 1915. Type. Cambodia. Kampot: “Kampot”, 20 Dec 1903, *C. Geoffray 261* (lectotype, designated here: P [P00055825]; isolectotype: P [P00761034]).

#### Type.

Cultivated in Turin, Italy (Torino), *Anonymous s.n.* (holotype: TO [fide Nee 1979]).

#### Description.

Vorontsova and Knapp (2016: 108–111); http://www.solanaceaesource.org/solanaceae/solanum-capsicoides.

#### Distribution.

*Solanumcapsicoides* is widespread but scattered throughout tropical Asia (see Table 2); it is native to Southern South America and is widely adventive and cultivated for ornament.

#### Common names.

China. niu qie zi (Zhang et al. 1994).

#### Discussion.

*Solanumcapsicoides* is a member of the Acanthophora clade along with several other American species introduced to tropical Asia (e.g., *S.aculeatissimum*, *S.mammosum*, *S.viarum*). It can be distinguished from those taxa and from any native species in the region in its absence of any stellate trichomes (all trichomes are simple, but derived from stellate ones, see Levin et al. 2005) and large dark orange berries with winged seeds.

In the description of *S.bodinieri*, Léveillé (1908) did not cite a herbarium or a particular specimen. The herbarium of the French botanist and clergyman Augustin A.H. Léveillé was acquired by the Scottish botanist George Forrest from whence it passed to the Royal Botanic Garden Edinburgh. Vorontsova and Knapp (2016) incorrectly assumed the sheet in E was the holotype. We here lectotypify this name using the specimen at E (E00284479) they cited as holotype.

Bonati (1915) did not cite a specimen or herbarium in his description of var. geoffrayi. Hul and Dy Phon (2014) cited “holo-, P!”, presumably referring to one of two sheets of this gathering in P. We select as lectotype the sheet at P with the original label (P00055825) that was presumably the sheet referred to by Hul and Dy Phon (2014).

#### Specimens examined.

See Suppl. materials 1–3.

### ﻿7. Solanum chrysotrichum

Taxon classificationPlantaeSolanalesSolanaceae

Schltdl., Linnaea 19: 304. 1847.

B6741858-EB17-5FCE-A1AE-BE062D2DCB6F

Figs 5H , 22A Only names based on tropical Asian types listed here; for complete synonymy see Vorontsova and Knapp 2016.

Solanum torvum Sw. var. pleiotomum C.Y.Wu & S.C.Huang, Acta Phytotax. Sin. 16(2): 73. 1978. Type. China. Fujian: “Foojow, Tsang Tsien Shen”, 28 Mar 1937, *H. Migo s.n.* (holotype: PE).

#### Type.

Mexico. Michoacan or Jalisco[?]: near Las Trojes, 1825-31, *C.J.W. Schiede 81* (lectotype, designated by Nee 1993, pg. 45: HAL [n.v.]).

#### Description.

Vorontsova and Knapp (2016: 120–122); http://www.solanaceaesource.org/solanaceae/solanum-chrysotrichum-0.

#### Distribution.

*Solanumchrysotrichum* has been recorded from China, India, Malaysia, and Sri Lanka; it is native Mexico and Central America. Outside of its native range it has been used as a fencing treelet and is widely adventive (see Vorontsova and Knapp 2016).

#### Common names.

China. duo lie shui qie (Zhang et al. 1994).

#### Discussion.

*Solanumchrysotrichum* is a member of the Torva clade (sensu Stern et al. 2011; Aubriot et al. 2016a). It is morphologically similar to the Torva clade species native to tropical Asia (see Aubriot et al. 2016a for a discussion of this disjunction), but can be distinguished by its rusty pubescence, larger flowers and leathery green, rather than yellow, red or black berries. It is most similar to *S.torvoideum*, but the stellate trichomes of *S.chrysotrichum* are generally longer stalked than those of *S.torvoideum* and are more reddish brown rather than golden brown and the inflorescences of S. *chrysotrichum* are distinctly pedunculate, while flowers of *S.torvoideum* are borne right next to the stem.

In northern India *S.chrysotrichum* forms large stands along disturbed roadsides together with *S.erianthum* D.Don (Brevantherum clade), another widely distributed introduced species. *Solanumchrysotrichum* has often been identified as *S.hispidum* Pers.; that name is a synonym of the Andean species *S.asperolanatum* Ruiz & Pav. In the absence of voucher specimens, references to *S.hispidum* or *S.asperolanatum* in tropical Asian floristic works are difficult to assign to a species.

#### Specimens examined.

See Suppl. materials 1–3.

### ﻿8. Solanum comitis

Taxon classificationPlantaeSolanalesSolanaceae

Dunal, Prodr. [A. P. de Candolle] 13(1): 345. 1852.

DD0F77EC-0B41-5581-B91F-E9649698AC71

Fig. 12

Solanum comitum St.-Lag., Ann. Soc. Bot. Lyon 7: 135. 1880, nom. illeg. superfl. Type. Based on Solanumcomitis Dunal.

#### Type.

Indonesia. Java: Sin. loc., 1837, *Without collector* [*J.C. von Hoffmannsegg*] *s.n.* (holotype: G-DC [G00131442]; isotype: W [acc. # 1889-0135755]).

#### Description.

Habit not known, but probably shrubs or small trees, unarmed or armed with a few tiny prickles hidden by dense pubescence. Stems erect, terete, with a few tiny prickles and densely stellate-pubescent; prickles 1–3 mm long, very sparse if present, broad-based, straight, pale yellowish tan, usually absent; pubescence of mixed sessile and very short-stalked porrect-stellate trichomes, the stalks to 0.5 mm long, the rays 8–10, ca. 0.5 mm long, weak and tangled, the midpoints absent or to 0.4 mm long; new growth densely stellate-pubescent, the trichomes tangled whitish grey in dry material; bark of older stems grey (but only quite young stems seen). Sympodial units difoliate, the leaves geminate, the leaves of pair equal in size and shape or one leaf slightly smaller. Leaves simple, not lobed, the blades 5–11 cm long, 2.5–5 cm wide, ca. 2 times longer than wide, elliptic to narrowly elliptic, chartaceous, strongly discolorous, unarmed or sometimes sparsely armed along the midrib and major veins with small straight prickles; adaxial surface evenly and sparsely pubescent with erect short-stalked multangulate trichomes, the multiseriate stalks 0.1–0.5 mm long, the rays 6–8, ca. 0.4 mm long and all directly upwards (no clear midpoint), the lamina surface clearly visible; abaxial surface densely pubescent with tangled long-stalked porrect-stellate trichomes, the stalks ca. 1 mm long, the rays 8–10, to 0.6 mm long, thin and delicate; major veins ca. 5 pairs, densely pubescent especially abaxially, with a few tiny prickles abaxially; base acute-attenuate, somewhat oblique; margins entire; apex acute, the tip rounded; petioles 1–1.5 cm long, 1/8–1/4 as long as the leaf blades, sparsely prickly and densely stellate-pubescent, with 1–2 prickles to 2 mm long or more commonly prickles absent, the pubescence of sessile and short-stalked stellate-trichomes like those of the stems. Inflorescences 1–5 cm long, internodal and lateral, unbranched, with 5–15 flowers, only a few flowers open at any one time, densely pubescent with white sessile and short-stalked stellate-porrect trichomes like those of the stems, with 6–8 rays ca. 0.5 mm long and midpoints to 0.4 mm long or absent; peduncle absent and the first inflorescence branches appearing to arise directly from the stem, or to 0.3 cm long, unarmed; pedicels 1.2–1.7 cm long, ca. 1.5 mm in diameter at the base and apex, spreading to erect at anthesis, unarmed, densely stellate-pubescent like the inflorescence axes, articulated at the base; pedicel scars irregularly spaced 1.5–2 mm apart. Buds elongate ellipsoid and somewhat tapering, strongly exserted from the calyx before anthesis. Flowers 5-merous, apparently all perfect, but some distal flowers may be short-styled. Calyx with the tube ca. 3 mm long, conical, the lobes 1.5–2 mm long, 1–2 mm wide, deltate, unarmed and densely white stellate-pubescent abaxially with porrect-stellate trichomes like those of the pedicels. Corolla 2–2.6 cm in diameter, colour not recorded, shallowly stellate, lobed ca. halfway to the base, interpetalar tissue present and abundant, the lobes 6–9 mm long, 6–8 mm wide, deltate, spreading at anthesis, mostly glabrous adaxially but with a few stellate trichomes at the tips, densely stellate-pubescent abaxially with densely tangled sessile trichomes where exposed in bud, these densest at the tips, the interpetalar tissue glabrous or with a few stellate trichomes abaxially. Stamens equal or very slightly unequal, if unequal 3 longer than the other 2; anthers (longer 3) 7.5–8 mm long, ca. 1.5 mm wide, (shorter 2) 5–6 mm long, ca. 1 mm wide, all tapering, yellow, glabrous, poricidal at the tips, the pores directed distally, not elongating to slits with drying; filament tube minute, glabrous; free portion of the filaments ca. 0.5 mm long, glabrous. Ovary conical, glabrous; style 10–13 mm long, glabrous; stigma capitate, the surfaces minutely papillose. Fruit a globose berry, several per infructescence, ca. 1.2 cm in diameter, colour not known, pericarp thin and shiny, glabrous; fruiting pedicels 1.6–1.8 cm long, 0.8–1 mm in diameter at the base, 2–2.5 mm in diameter at the apex, erect and slightly woody; fruiting calyx lobes ca. 2.5 mm long, not markedly accrescent, but covering the base of the berry and not reflexed. Seeds 20–50 per berry, ca. 3 mm long, ca. 2.3 mm wide, flattened reniform, pale yellowish tan, the surfaces minutely pitted, testal cells shape not clear. Chromosome number: not known.

**Figure 12.**
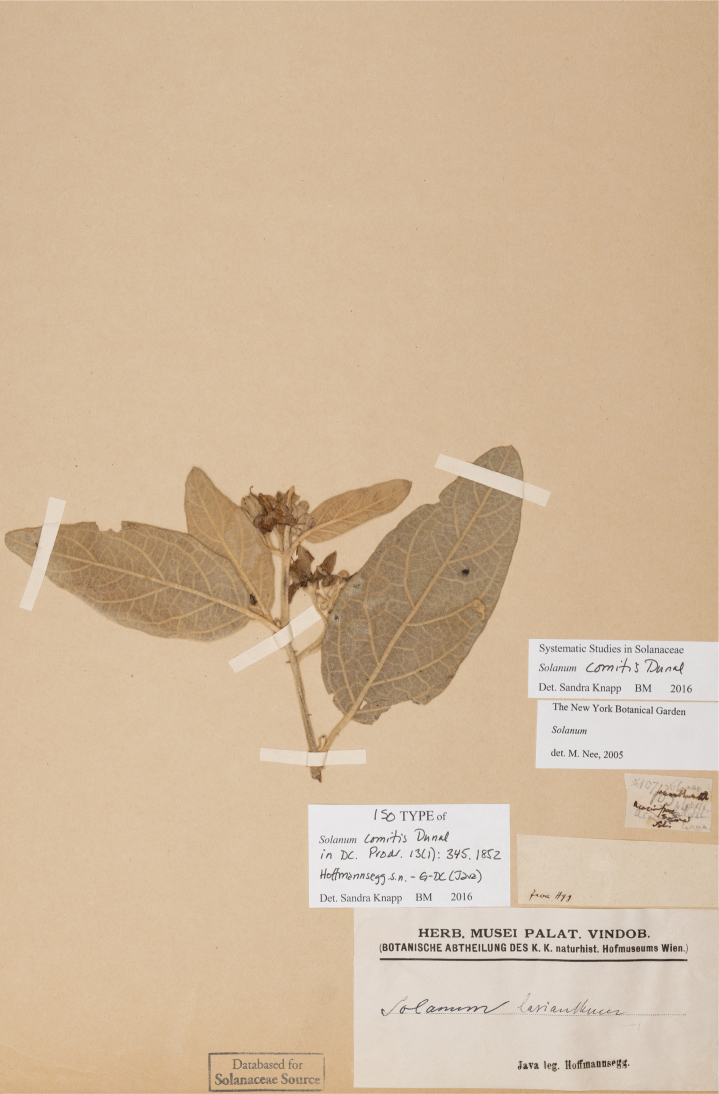
*Solanumcomitis* Dunal. Herbarium specimen (isotype) collected in Indonesia (*Without collector [J.C. von Hoffmannsegg] s.n.*, W [acc. # 1889-0135755]). Photograph credit: CC BY, Naturhistorisches Museum, Wien.

**Figure 13.**
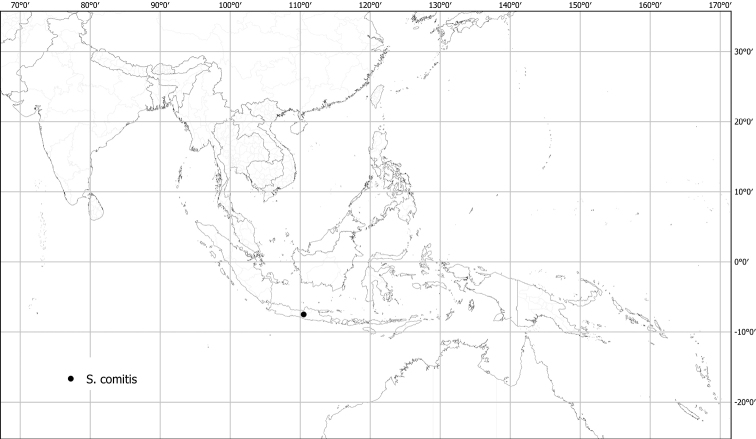
Distribution of *Solanumcomitis*.

#### Distribution

**(Fig. 13).***Solanumcomitis* is endemic to the island of Java, Indonesia. No specific localities are recorded on the only two gatherings known of this species.

#### Ecology and habitat.

No habitat information has been recorded for *S.comitis*.

#### Common names and uses.

None recorded.

#### Preliminary conservation status

**(IUCN 2019).** Data Deficient (DD); known only from two collections of uncertain specific locality. *Solanumcomitis* has not been re-collected since the early 19^th^ century, indicating it is certainly of conservation concern. Recollection of this distinctive species and discovery of any precise localities for its occurrence are priorities.

#### Discussion.

*Solanumcomitis* is a distinctive species, with dense pubescence of multangulate trichomes that dries with a whitish grey tinge. It is superficially similar to the western Australian *S.lasiophyllum* Dunal with dense whitish grey pubescence but differs from that species in its smaller flowers that are probably all hermaphroditic, smaller berries and completely different habitat (tropical versus dry and seasonal). Its relationships are not known, but in the inflorescence, flower and fruit morphology *S.comitis* resembles members of the Torva clade that occur in the Asian tropics (e.g., *S.poka*, *S.pseudosaponaceum*) and we suspect it is a member of that group. Re-collection of this species is a priority.

Although the type specimen is attributed to J.C. Graf van Hoffmansegg, he never actually collected in Java, but rather employed a friend who made natural history collections for him (van Steenis-Kruseman 1985).

#### Specimens examined.

See Suppl. materials 1–3.

### ﻿9. Solanum cordatum

Taxon classificationPlantaeSolanalesSolanaceae

Forssk., Fl. Aegypt.-Arab. 47. 1775.

2C30A932-DA58-50C9-AA5E-A6730B7CE1E8

Fig. 14

Solanum gracilipes Decne., Voy. Inde [Jacquemont] 4(Bot.): 113, t. 119. 1844. Type. India. “India Borealis Occidentalis”, *V. Jacquemont 63* (holotype: P [P00054212]).
Solanum sabeorum Deflers, Bull. Soc. Bot. France 43: 122. 1896. Type. Yemen. N side of Mount Nakhai, Bilad Fodhli, 800 m, 31 Mar 1890, *M. Deflers 488* (lectotype, designated by Vorontsova and Knapp 2016, pg. 127: P [P00051784; isolectotypes: K [K000441136], P [P00051784, P00051785, P00051786]).
Solanum darassumense Dammer, Bot. Jahrb. Syst. 38: 57. 1905. Type. Somalia. Arussi-Galla, Darassuma, 900 m, Apr 1900, *H. Ellenbeck 2024* (lectotype, designated by Vorontsova and Knapp 2010, pg. 1596: GOET).
Solanum obbiadense Chiov., Boll. Soc. Bot. Ital. 1925: 106. 1925. Type. Somalia. “Sultanate of Obbia, Biomal”, 2 May 1924, *N. Puccioni & J. Stefanini 1061 [605*] (neotype, designated by Vorontsova and Knapp 2016, pg. 127: FT [FT003066]).
Solanum nummulifolium Chiov., Boll. Soc. Bot. Ital. 1925: 107. 1925. Type. Somalia. “Sultanate of Obbia, Biomal”, 2 May 1924, *N. Puccioni & J. Stefanini 551 [605*] (neotype, designated by Vorontsova and Knapp 2016, pg. 127: FT [FT003065]).

#### Type.

Yemen. Naquil Khailan between Beui Harath and Nehm, NE of Sana’a, 2400 m, 12 May 1978, *J.R.I. Wood 2327* (neotype, designated by Wood 1984, pg. 136: K [K000441137]; isoneotype: BM [BM000942294]).

#### Description.

Scandent, sometimes erect herb to shrub, 0.3–1 m, unarmed or prickly. Young stems slender, creeping to ascendent, glabrescent, unarmed or sparsely prickly, the pubescence of multangulate, sessile trichomes visible to the naked eye as white dots, the rays 12–18, up to 0.1(–0.15) mm long, the midpoints reduced to a globular gland, the prickles, if present, 0–4 mm long, 0.1–2(–6) mm wide at the base, straight, sometimes slightly reflexed to curved, perpendicular to the stem, pale yellow to brown; bark of older stems glabrous, dark brown or dark grey to black. Sympodial units difoliate, not geminate. Leaves simple, entire, the blades 0.7–1.5(–3) cm long, 0.7–1.3(–3.5) cm wide, 1(–1.5) times longer than wide, orbicular, sometimes ovate, membranous, drying concolorous, yellowish green, glabrescent on both surfaces, with porrect, sessile, sometimes stalked trichomes, the stalks to 0.1 mm long, the rays 8–16, 0.05–0.1(–0.15) mm long, the midpoints reduced or a mere bump, unarmed on both surfaces; the primary veins not visible or 2–4 pairs, the tertiary venation not visible to the naked eye; base cordate to attenuate; apex rounded to acute; petiole 0.2–1.3 cm long, 1/3 as long to equal in length to the leaf blade, narrowly winged to almost filiform, sparsely stellate-pubescent, unarmed. Inflorescences apparently lateral, 1.5–4 cm long, unbranched, with 1(-2) flowers, 1(-2) flowers open at any one time; peduncle absent; pedicels 0.5–3 cm long, erect, filiform, protruding beyond the leaves, articulated at the base, moderately stellate-pubescent to glabrescent, unarmed; pedicel scars spaced 1–4 mm apart. Buds narrowly ellipsoid, the corolla strongly exserted from the calyx before anthesis. Flowers 4–5-merous, apparently all perfect. Calyx 2–4 mm long, the lobes 0.5–2 mm long, ca. 1 mm wide, deltate to narrowly deltate, apically obtuse to acuminate, sparsely stellate-pubescent, unarmed. Corolla 0.9–1.6 cm in diameter, mauve to purple, stellate, lobed 2/3–4/5 of the way to the base, the lobes 3–6.5 mm long, 1.2–2 mm wide, deltate, reflexed or spreading, stellate-pubescent abaxially, the trichomes porrect, sessile, the rays 8–15, up to 0.1 mm long, the midpoints shorter than the rays or reduced to bulbous base. Stamens equal; filament tube ca. 0.1 mm long; free portion of the filaments 0.5–0.7 mm long; anthers 3–5 mm long, yellow, spreading, tapering, poricidal at the tips, the pores distally directed. Ovary glabrous or with 1–2 stellate trichomes near the apex; style 0.8–0.9 cm long, filiform, curved, glabrous. Fruit a globose berry, 1(-2) per infructescence, 0.6–0.8 cm in diameter, the pericarp thin, glabrous, red at maturity; fruiting pedicels 1.5–3.5 cm long, 0.3–0.4 mm in diameter at the base, 1.5–2 mm in diameter at the apex, herbaceous, pendulous, unarmed; fruiting calyx weakly accrescent, elongating to 3–5 mm long, covering 1/4–1/3 of the mature fruit, unarmed. Seeds ca. 10–20 per berry, 1.8–3 mm long, 1.5–2.2 mm wide, flattened-reniform, dark brown to almost black, the testal cells somewhat sinuate in outline. Chromosome number: n = 12, 2n = 24 (Kumar and Subramaniam 1987, as *S.gracilipes*).

**Figure 14.**
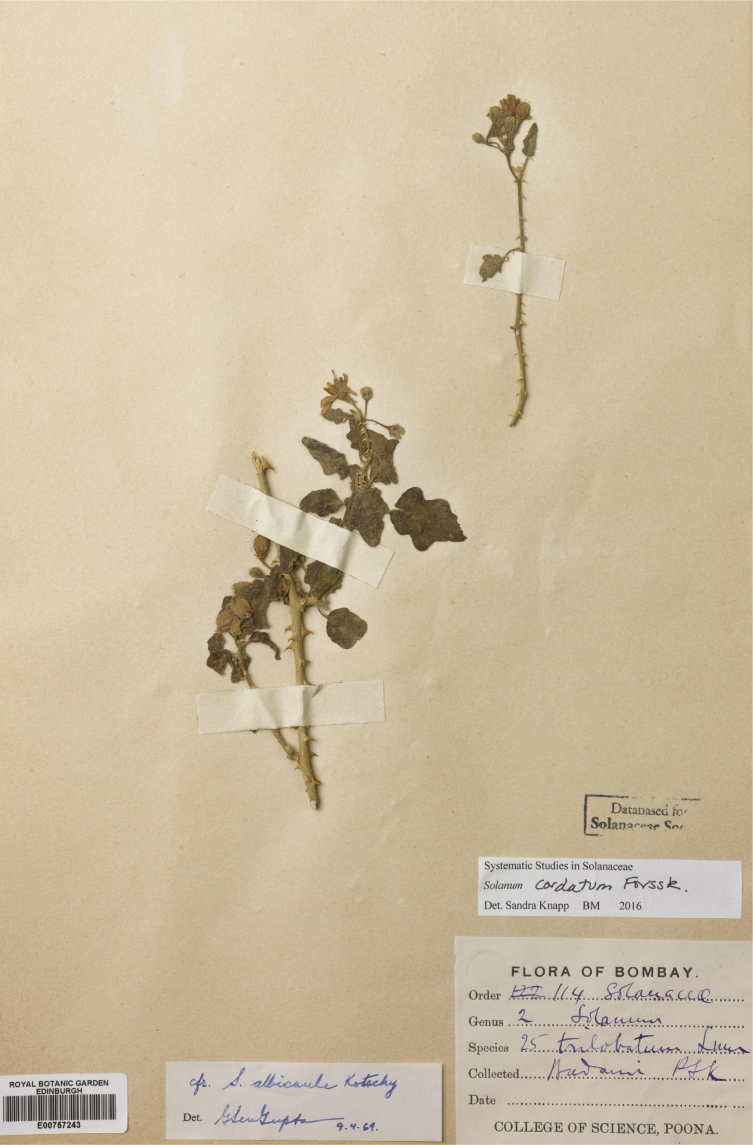
*Solanumcordatum* Forssk. Herbarium specimen collected in India (*Badami 114*, E00757243). Photograph credit: Royal Botanic Garden Edinburgh.

#### Distribution

**Figure 15.**
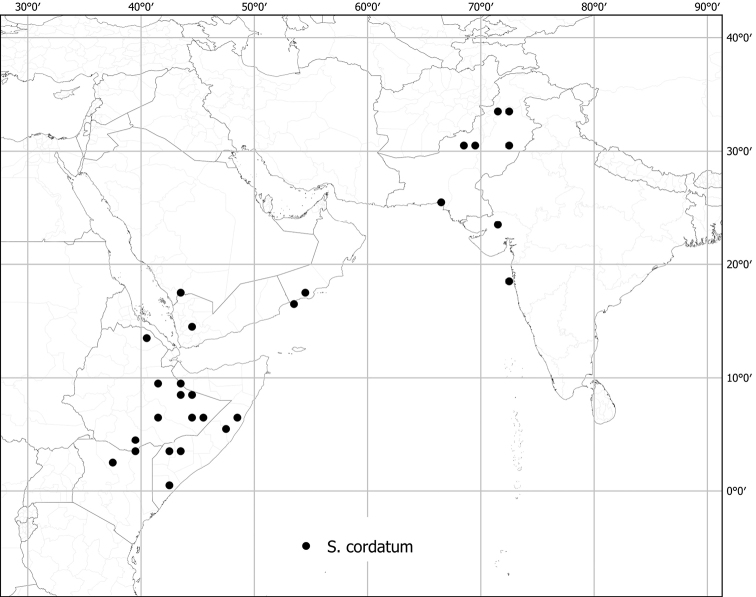
Distribution of *S.cordatum*.

**(Fig. 15).***Solanumcordatum* occurs from northeastern Africa and the Arabian Peninsula to Pakistan and western India (Punjab, Gujarat, Maharashtra). The record of *S.cordatum* from Tamil Nadu (Ramachandran and Viswanathan 2010) is based on mis-identified specimens of *S.wightii*.

#### Ecology and habitat.

*Solanumcordatum* occurs in grassland, bushland, and open woodland on silty, sandy, or stony soil; low elevations (not recorded for India, from sea level to 1,500 m fide Vorontsova and Knapp 2016).

#### Common names and uses.

None recorded from the region (see Vorontsova and Knapp 2016 for Africa).

#### Conservation status

**(Knapp 2021a).***Solanumcordatum* has been formally assessed as a species of Least Concern (LC; https://www.iucnredlist.org/species/186619213/186619254).

#### Discussion.

*Solanumcordatum* appears to be much less commonly collected in western India than is the very similar and sympatric *S.forskalii*. It differs from *S.forskalii* in its compact multangulate stem pubescence of trichomes with many very short (<0.1 mm long) rays, *S.forskalii* has porrect-stellate trichomes with fewer rays that are marginally longer (> 0.15 mm long); stems of *S.cordatum* appear white dotted from the stubby trichomes. Leaf petioles of *S.cordatum* are decurrent and narrowly winged, while leaves of *S.forskalii* are distinctly petiolate. The calyx lobes of *S.cordatum* are longer and narrower than those of *S.forskalii*, while anthers are shorter in *S.cordatum* (3–5 mm versus 4.5–7 mm).

In eastern Africa, *S.cordatum* is deciduous in the dry season, growing opportunistically during times of moisture (Vorontsova and Knapp 2016). This may be part of the reason for its relatively fewer collections in India than *S.forskalii*; this species has more robust leaves that are not deciduous. Wood (1997) suggested that *S.cordatum* was an “Indian species which extends west to Yemen but is only found in Africa in Somalia.” It is indeed found only in the Horn of Africa (Somalia, Ethiopia, and northern Kenya).

Ramachandran and Viswanathan (2010) reported *S.cordatum* from Sethukadai in the Namakkal District of Tamil Nadu, far from its known range in India. This report is based on mis-identified specimens of *S.wightii*; the description in Ramachandran and Viswanathan (2010) appears to be a mixture of elements from published descriptions of *S.cordatum* and the actual specimens of *S.wightii* they used as their new record.

#### Specimens examined.

See Suppl. materials 1–3.

### ﻿10. Solanum cyanocarphium

Taxon classificationPlantaeSolanalesSolanaceae

Blume, Bijdr. Fl. Ned. Ind. 13: 700. 1826.

7A20F66F-19CB-51E9-95D0-21DE68DD3DBF

Figs 1D , 4E , 16

Solanum sarmentosum Nees, Trans. Linn. Soc. London 17(1): 58. 1834, nom. illeg., non Solanumsarmentosum Lam., 1794. Type. Malaysia. Penang: Sin. loc., 1822, *N. Wallich s.n.* [Wallich Catal. 2628f] (lectotype, designated here: GZU [GZU000255826]; isolectotype: BM [BM000886340], K-W [K001116668]).
Solanum bullatorugosum Dunal, Prodr. [A. P. de Candolle] 13(1): 236. 1852. Type. Indonesia. Java: Sin. loc., *H. Zollinger 1018* (lectotype, designated here: G-DC [G00145849]; isolectotypes: G [G00301680], P [P00369075, P00369076]).
Solanum maingayi Kuntze, Revis. Gen. Pl. 454. 1891. Type. Malaysia. Malacca: Sin. loc., *A.C. Maingay 1158* (lectotype designated by Turner 1998, p. 40, as “holotype”: K [K001080494]; isolectotype: LAE [acc. # 229591]).
Solanum sparsiflorum Elmer, Leafl. Philipp. Bot. 5: 1838. 1913, nom. illeg., non Solanumsparsiflorum Dammer, 1912. Type. Philippines. MIMAROPA: “Puerto Princesa (Mount Pulgar), Province of Palawan, Island of Palawan”, May 1911, *A.D.E. Elmer 13157* (lectotype, designated here: GH [00077851]; isolectotypes: BISH [BISH1005088], BM [BM000778206], CAL [acc. # 316497], E [E00273861], F [acc. # 384070, v0073463F], G [G00343321], HBG [HBG511492], K [K000195917], L [L0003665], LAE [acc. # 229593], LE, MO [acc. # 706769, MO-2289036], NY [00172293], P [P00379711], W [acc. # 1913-0005906], U [U0113978], US [00027804, acc. # 873055]).
Solanum thorelii Bonati, Bull. Soc. Bot. Genève, 1913, sér. 2, 5: 310. 1914. Type. Vietnam. Tây Ninh: “Caï Cong, environs de village”, 1862, *C. Thorel 1419* (lectotype, designated here: P [P00054148]; isolectotypes: P [P00054149, P00054150]).
Solanum sakhanii Hul, Fl. Photogr. Cambodge 522. 2013. Type. Cambodia. Sihanoukville: “Sihanoukville”, 3 May 2008, *K.C. Cheng et al. CL929* (holotype: P [P00836398]; isotypes: P [P00836399, P00836400]).

#### Type.

Indonesia. Java: West Java [“in oryzetis siccis montium Seribu” Curug Seribu near Bogor; from protologue], *C.L. Blume s.n.* (lectotype, designated here: L [L0003630]).

#### Description.

Herbs to small shrubs, creeping over the ground, to 1 m tall, armed. Stems decumbent, terete, black to dark brownish, prickly and sparsely stellate-pubescent; prickles to 5 mm long, to 2.5 mm in diameter at the base, straight or curved at the tip, awl-shaped to deltate, flattened, pale yellow in dry material, tan or purplish black in live plants, glabrescent; trichomes porrect-stellate, sessile to stalked, the stalks to 0.1 mm long, the rays 4–7, 0.1–0.4 mm long, the midpoints absent or to 0.4 mm long, sometimes purplish black in live plants; new growth moderately to densely stellate-pubescent, light green in dry material; bark of older stems brownish grey, sparsely stellate-pubescent. Sympodial units plurifoliate, the leaves usually not geminate. Leaves simple, more or less deeply lobed, the blades 4.5–9 cm long, 2–5 cm wide, ca. 2 times longer than wide, elliptic to ovate, chartaceous, slightly discolorous, moderately prickly with 3–7 prickles per leaf side, the prickles to 1 cm long, to 1 mm wide at the base, straight at the tip, awl-shaped, conical, pale yellow in dried material sometimes purplish black in live plants, glabrous; adaxial surface mid-green, moderately stellate-pubescent, the stellate trichomes porrect, sessile to stalked, the stalks to 0.1 mm long, the rays 3–5, 0.1–0.4 mm long, the midpoints to 1 mm long, 2–3 times longer than the rays; abaxial surface light green, moderately stellate-pubescent with trichomes like those of the adaxial surface; major veins 4–5 pairs, drying dark; base cuneate to truncate; margins shallowly to deeply lobed, the lobes 1–4 on each side, 0.5–1 cm long, deltate to oblong, apically rounded, the sinuses extending up to halfway to the midrib; apex rounded to acute; petiole 0.7–2.2 cm long, 1/8–1/5 of the leaf blade length, moderately stellate-pubescent, armed with 1–4 prickles like those of the blades. Inflorescences 1.5–3.5 cm long, apparently lateral, unbranched, with ca. 1–4 flowers, 1–2 flowers open at any one time, sparsely to moderately stellate-pubescent with trichomes like those of the stems but with longer midpoints, unarmed; peduncle 0–8 mm long, unarmed; pedicels 0.5–2 cm long, ca. 0.5 mm in diameter at the base, ca. 1 mm in diameter at the apex, spreading to erect to somewhat nodding at anthesis, unarmed or with 1–11(–16) prickles, moderately stellate-pubescent with trichomes like those of the inflorescence axes, articulated at the base; pedicel scars spaced 0.5–6(–18) mm apart. Buds globose to oval, strongly included in the calyx lobes. Flowers 5-merous, apparently all perfect. Calyx with the tube 2–4 mm long, campanulate, the lobes 2–5 mm long, 1–1.5 mm wide, narrowly deltate, apically acute, densely prickly and stellate-pubescent abaxially with numerous prickles and trichomes like those of the pedicels but with shorter midpoints. Corolla 1–1.5 cm in diameter, white or purple, stellate, lobed ca. 1/2 of the way to the base, the lobes 3–6 mm long, 2–3 mm wide, deltate, spreading or somewhat campanulate and erect (not spreading and perpendicular to the pedicel) at anthesis, glabrous adaxially, moderately to densely stellate pubescent abaxially, the trichomes often purple-tinged. Stamens equal; anthers 3–5 mm long, ca. 0.5 mm wide, connivent, tapering, orange, glabrous, poricidal at the tips, the pores directed distally, not elongating to slits with drying; filament tube minute, glabrous; free portion of the filaments ca. 1 mm long, glabrous. Ovary conical, minutely glandular-puberulent; style ca. 5 mm long, slender, curved at the apex, glabrous; stigma capitate, the surface minutely papillate. Fruit a globose berry, 1–4 per infructescence, 1–1.6 cm in diameter, red when mature, the pericarp thin and smooth, glabrous; fruiting pedicels 2.5–4 cm long, 0.5–1 mm in diameter at the base, 1–1.5 mm in diameter at the apex, woody, deflexed and nodding, unarmed or with 1–10 prickles; fruiting calyx lobes elongating to 1.3 cm long, 1/2–3/4 the length of the mature fruit, the tips slightly reflexed, usually completely enclosing at least the lower half of the berry, with 6–17(–24) prickles, these often purplish black. Seeds 85–170 per berry, 1.75–2 mm long, 1.5–2 mm wide, flattened reniform, dull yellow, the surface minutely pitted, the testal cells sinuate in shape. Chromosome number: not known.

**Figure 16.**
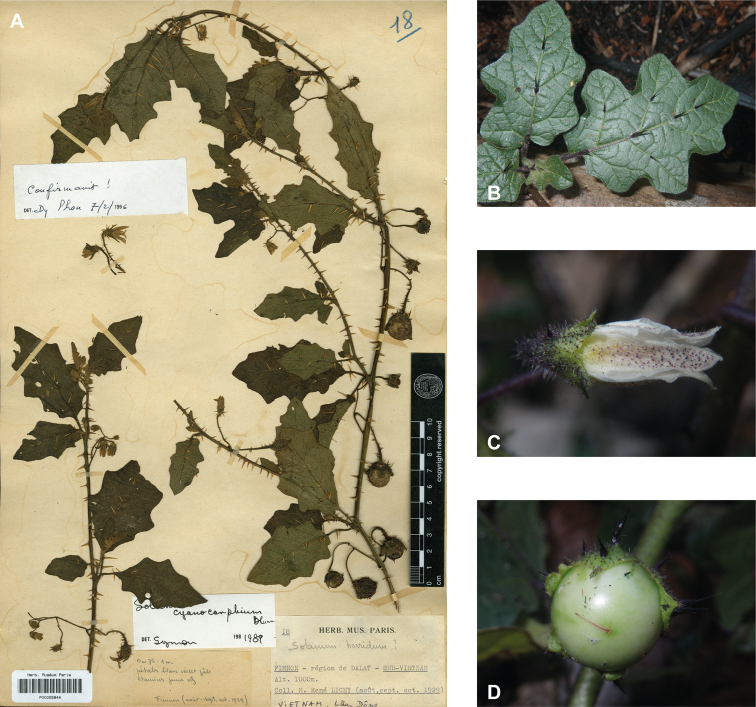
*Solanumcyanocarphium* Blume **A** herbarium specimen collected in Vietnam in 1929 (*Lichy 18*, P00055844) **B** detail of the leaves and prickles (field photograph, unvouchered, Vietnam) **C** detail view of a flower (field photograph, unvouchered, Vietnam) **D** detail view of a fruit (field photograph, unvouchered, Vietnam). Photograph credits: **A** CC-BY, Muséum national d'Histoire naturelle, Paris **B–D** M. Nuraliev.

#### Distribution

**Figure 17.**
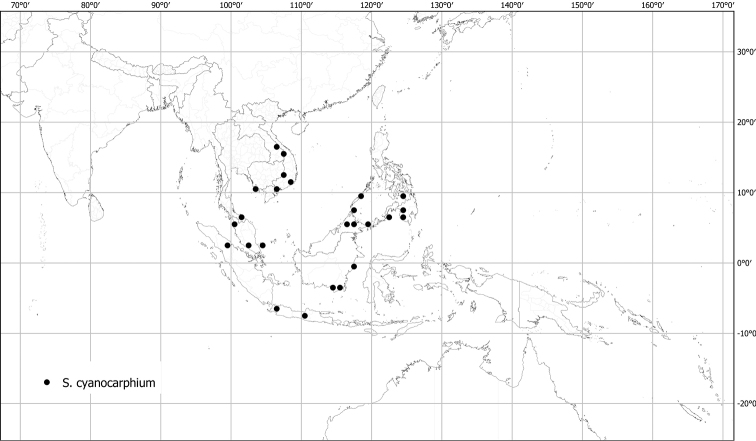
Distribution of *S.cyanocarphium*.

**(Fig. 17).***Solanumcyanocarphium* is widely distributed from southeastern Indochina to western Malay Archipelago (Borneo, Java, Sumatra and south Philippines).

#### Ecology and habitat.

*Solanumcyanocarphium* has been found growing on limestone, in riparian and secondary tropical forests as well as in open degraded vegetation; from 10 to 1,000 m elevation.

#### Common names and uses.

Cambodia. plone lane [Khmer] (*Chassagne 138*), trâp krab [Khmer] (Hul and Dy Phon 2014); Indonesia. North Sumatra: tioeng (*Toroes 1639*), teroeng oetan (*Toroes 2926*); Malaysia/Singapore. tĕrong puyoh, tĕrong tikus, tĕrong pipit (all meaning “little brinjal”, Burkill 1935); Vietnam. Dông Nai: blou xit [Mnong] (*Pierre s.n.*), cà co [Vietnamese] (*Pierre s.n.*).

*Solanumcyanocarphium* is said to have edible berries (*Cuadra A1010*) and the juice is drunk for fever (Burkill 1935). Burkill (1935) also records its use to improve the appetite of elephants.

#### Preliminary conservation status

**(IUCN 2019).** Least Concern (LC); EOO 3,563,723 km^2^ (LC), AOO 92 km^2^ (EN). Historical collections of *Solanumcyanocarphium* suggest that this species is widely distributed but absence of recent collections from large areas of the historical range suggests it may merit conservation concern because of the increasing anthropogenic alteration of natural habitat throughout lowland and coastal tropical Asia (Wikramanayake et al. 2002).

#### Discussion.

*Solanumcyanocarphium* is a weakly climbing shrub to vine that scrambles over vegetation with hooked prickles; most specimens are quite thin-stemmed and somewhat scrappy. It is superficially similar to *S.procumbens* but differs from it in the strongly accrescent and spiny fruiting calyx (not accrescent in *S.procumbens*), larger prickles on stems and leaf blades, deeply lobed leaves (leaves usually entire in *S.procumbens*) and larger and many seeded berries. The accrescent calyx in fruit is similar to that of *S.involucratum*, but that species is more robust and has pubescent, rather than glabrous berries. Hul and Dy Phon (2014) suggested that *S.cyanocarphium* was introduced to Indochina but described what we recognise as a synonym (*S.sakhanii*) as new; we consider *S.cyanocarphium* to be native across tropical Asia where it occurs.

Aubriot et al. (2016a) included *S.cyanocarphium* in a clade called ‘S.cyanocarphium + S.sakhanii’; subsequent examination shows that the two taxa are synonyms (see above), not surprising given the very short branch lengths (Aubriot et al. 2016a).

The Blume specimen in L (L0003630) we have selected as the lectotype of *S.cyanocarphium* is the only one we have found that corresponds to the protologue and is likely to have been the original material that Blume used.

Nees van Esenbeck (1834) only cited a Wallich catalogue number in the protologue of *S.sarmentosum*, but no herbarium. The Solanaceae from his own herbarium are now held at the herbarium (GZU) of the University of Graz (Stafleu and Cowan 1981) and we have selected the specimen there (GZU000255826) as the lectotype, it has flower and fruit and an annotation “S.sarmentosum” in Nees van Esenbeck’s hand.

The protologue of *S.bullatorugosum* (Dunal 1852) cites two specimens at G; we have selected the more complete sheet at G-DC (G00145849) with both flowers and fruits as the lectotype.

The collection cited in the protologue of *S.sparsiflorum* (Elmer 1913) without specification of any herbarium (*Elmer 13157*) is represented in many collections; we have chosen that at GH (0007785) as the lectotype because it is the duplicate best matching the protologue and has both flower and mature fruit. Hul and Dy Phon (2014) cited a sheet in PNH as “holo-, PNH” but this does not constitute effective lectotypification.

Hul and Dy Phon (2014) cited a holotype at P for *S.thorelii*, but no herbarium was cited in the original protologue (Bonati 1914). We select here the best preserved of the three duplicates of *Thorel 1419* held at P (P00054148) as the lectotype.

#### Specimens examined.

See Suppl. materials 1–3.

### ﻿11. Solanum deflexicarpum

Taxon classificationPlantaeSolanalesSolanaceae

C.Y.Wu & S.C.Huang, Acta Phytotax. Sin. 16(2): 73. 1978.

38FC8F48-72A3-5B27-9031-6870769AA74B

Fig. 18

Solanum indicum L. var. recurvatum C.Y.Wu & S.C.Huang, Acta Phytotax. Sin. 16(2): 73. 1978. Type. China. Yunnan: Dali, “Bohai city [transl. from the label]”, 21 Feb 1957, *Yunnan expedition 5005* (holotype: KUN [KUN184304]; isotypes: IBK [IBK00241973], IBSC [IBSC0004706], PE [PE00031389, PE02079736], SZ [SZ00254985, SZ00254997]).

#### Type.

China. Yunnan: “Xiangping Mountain, Xishou [transl. from protologue]”, 29 Aug 1947, *G. Feng 11429* (holotype: KUN [KUN484279]; isotypes: A [A00310057], PE [PE00730648], WUK [WUK0194818]).

#### Description.

Shrubs to 2 m tall, unarmed or rarely sparsely armed. Stems erect, terete, moderately stellate-pubescent; prickles, if present, to 5 mm long, to 3 mm wide at the base, broad-based, curved, pale yellowish tan, sparsely stellate-pubescent near the base; pubescence of sessile to very short-stalked porrect-stellate trichomes, the rays 5–7, 0.1–0.4 mm long, the midpoints absent or to 0.5 mm long; new growth densely stellate-pubescent, the trichomes white and tangled, soon deciduous and the stems glabrate; bark of older stems greyish brown to grey. Sympodial units difoliate, the leaves geminate, or not geminate, the leaves of a pair equal in size and shape. Leaves simple, shallowly lobed, the blades 5–11 cm long, 2.5–7.5 cm wide, ca. 2 times longer than wide, elliptic, chartaceous, discolorous, unarmed or sparsely armed along the midrib and major veins with small curved prickles; adaxial surface evenly and sparsely to moderately stellate-pubescent with white-cream sessile porrect trichomes, the rays 4–6, 0.1–0.2 mm long, the midpoints 0.5–1.3 mm long; abaxial surface with similar sessile porrect-stellate trichomes, but with some short-stalked stellate trichomes with stalks to 0.5 mm and longer rays to 0.5 mm long; major veins 3–4 pairs, densely pubescent especially abaxially; base abruptly truncate, somewhat oblique; margins shallowly lobed, the lobes 3–4 on each side, 0.3–1 cm long, broadly deltate, apically rounded, the sinuses less than halfway to the midrib; apex acute; petioles 1–2 cm long, 1/4–1/2 as long as the leaf blades, sparsely prickly and moderately pubescent, the prickles 0–3, like those of the stems, the pubescence of sessile stellate-trichomes like those of the stems. Inflorescences 1–3 cm long, internodal and lateral, unbranched, with 5–10 flowers, only 1 or 2 flowers open at any one time, pubescent with sessile stellate-porrect trichomes like those of the stems, with 4–6 rays ca. 0.2 mm long and midpoints to 0.5 mm long, unarmed or with a few small prickles to 3 mm long; peduncle 0.1–0.8 cm long, unarmed; pedicels 0.35–0.5 cm long, 0.5–1 mm in diameter at the base, ca. 1 mm in diameter at the apex, spreading and slightly nodding at anthesis, unarmed or with a few small prickles to 1 mm long, densely stellate-pubescent like the inflorescence axes, articulated at the base; pedicel scars irregularly spaced 3–4 mm apart. Buds ellipsoid and somewhat tapering, strongly exserted from the calyx before anthesis. Flowers 5-merous, apparently all perfect, but some distal flowers may be short-styled. Calyx with the tube 2–2.5 mm long, conical, the lobes 1–2 mm long, ca. 1 mm wide, deltate, unarmed or with a few small prickles to 3 mm long and densely stellate-pubescent abaxially with sessile porrect-stellate trichomes like those of the pedicels. Corolla 0.8–1 cm in diameter, white, deeply stellate, lobed 3/4 of the way to the base, interpetalar tissue present, the lobes 4–4.5 mm long, 2–2.5 mm wide, deltate, spreading at anthesis, mostly glabrous adaxially but with a few stellate trichomes at the tips, densely stellate-pubescent abaxially with densely tangled sessile trichomes where exposed in bud, these densest at the tips. Stamens equal; anthers 3–4 mm long, ca. 2 mm wide, slightly tapering, yellow, glabrous, poricidal at the tips, the pores directed distally, not elongating to slits with drying; filament tube minute, glabrous; free portion of the filaments ca. 0.5 mm long, glabrous. Ovary conical, glabrous; style 5–6 mm long, glabrous; stigma capitate, the surfaces minutely papillose, bright green in live plants. Fruit a globose berry, several to many per infructescence, 1–1.2 cm in diameter, orange-red when ripe, the pericarp thin and shiny, glabrous; fruiting pedicels 0.8–1 cm long, 1–1.2 mm in diameter at the base, ca. 2 mm in diameter at the apex, somewhat woody, sharply deflexed and nodding, unarmed or with small prickles; fruiting calyx not accrescent, the lobes often breaking off. Seeds 50–60 per berry, 2–3 mm long, 2–3 mm wide, flattened reniform, pale tan or yellowish brown, the surfaces minutely pitted, the testal cells with sinuate margins. Chromosome number: not known.

**Figure 18.**
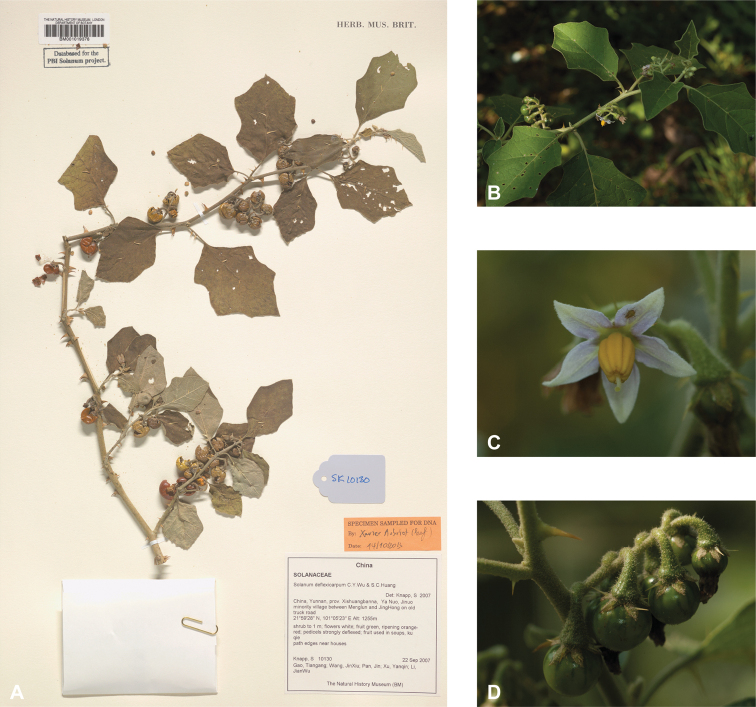
*Solanumdeflexicarpum* C.Y.Wu & S.C.Huang **A** herbarium specimen collected in China in 2007 (*Knapp et al. 10130*, BM001019378) **B** detail of a fertile branch (*Knapp et al. 10130*, China) **C** detail view of a flower (*Knapp et al. 10130*, China) **D** detail view of an infructescence (*Knapp et al. 10130*, China). Photograph credits: **A** CC-BY, © copyright The Trustees of the Natural History Museum, London **B–D** S. Knapp.

#### Distribution

**Figure 19.**
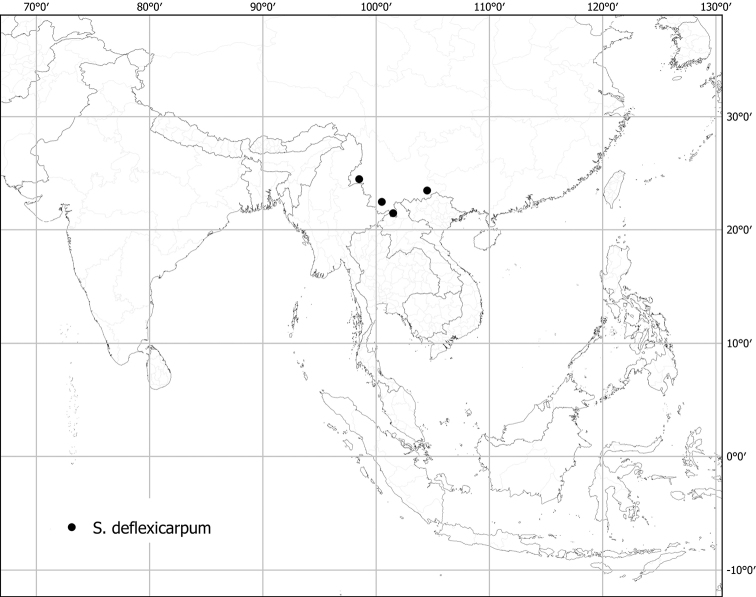
Distribution of *S.deflexicarpum*.

**(Fig. 19).***Solanumdeflexicarpum* is endemic to China (Yunnan province) and only known from few collections; potentially also present in northeastern Myanmar (*Andrew s.n.* collected in ‘Poneshee’ close to the boarder with Myanmar).

#### Ecology and habitat.

*Solanumdeflexicarpum* grows in open vegetation and roadsides in semideciduous tropical forests, from 1,255 to 1,500 m elevation.

#### Common names and uses.

China. ku ci (Zhang et al. 1994); Yunnan: ku ci, wan bing ci tian qie (pedicel curved and spiny, high altitude) [Mandarin] (*Feng 11429*).

#### Preliminary conservation status

**(IUCN 2019).** Near Threatened (NT). EOO (65,755 km^2^); AOO (16 km^2^). *Solanumdeflexicarpum* occurs over a relatively wide range, but there are very few collections; the paucity of collections suggest that the species is of some conservation concern, meriting further study.

#### Discussion.

*Solanumdeflexicarpum* is morphologically similar to the sympatric and very widespread *S.violaceum* and to the Indian endemics *S.hovei* and *S.multiflorum*; in the analyses of Aubriot et al. (2016a) it is part of a monophyletic group with those species. It differs from *S.violaceum* in its condensed inflorescences with white rather than violet flowers and strongly down-curved fruiting pedicels; the fruiting pedicels of *S.violaceum* are more spaced, straight and are strongly spreading from the axis. *Solanumdeflexiflorum* differs from *S.hovei* in its recurved fruiting pedicels (straight in *S.hovei*) and truncate, oblique (versus attenuate) leaf bases. The inflorescences of *S.multiflorum* are similarly condensed, but *S.deflexicarpum* differs from that species in its more compact trichomes, fewer flowers per inflorescence, and its distribution in southern China.

#### Specimens examined.

See Suppl. materials 1–3.

### ﻿12. Solanum dunalianum

Taxon classificationPlantaeSolanalesSolanaceae

Gaudich., Voy. Uranie 448. 1828.

49CD997C-01F9-566E-BCA9-A22F53E28CC4

Fig. 20

Solanum pulvinare Scheff., Ann. Jard. Bot. Buitenzorg 1: 39. 1876, as “*pulvinaris*”. Type. Indonesia. West Papua: Ajambori, near Doré [Dorei Bay], *J.E Teijsmann s.n.* [7854] (lectotype designated by Symon 1985, pg. 129: BO [acc. # 1324393]; isolectotype: MEL [MEL0104159]).
Solanum dunalianum Gaudich. var. lanceolatum Witasek, Repert. Spec. Nov. Regni Veg. 5: 166. 1908. Type. Papua New Guinea. East New Britain: “Vulcanes Kaia” [volcano Kaia], Sep 1905, *K. Rechinger & L. Rechinger 4821* (lectotype designated by Symon 1985, p. 129: W [acc. # 0022303]).
Solanum dunalianum Gaudich. var. puberius Bitter, Bot. Jahrb. Syst. 55: 72. 1919. Type. New Guinea. East Sepik: “Kaiser-Wilhelmsland, Hauptlager Malu” [base camp near present-day town of Ambunti], *C.L. Ledermann 10718, 12250* (syntypes; type material presumably destroyed at B, see Veldkamp et al. 1988; no duplicates found).

#### Type.

Indonesia. Malaku: Pisang Island, Moluccas Islands, [Dec 1818], *C. Gaudichaud s.n.* (lectotype designated by Bean 2004, pg. 672: P [P00369093]; isolectotype: P [P00369092]).

#### Description.

Shrub or small tree to 4 m tall, unarmed or sparsely prickly. Stems erect, unarmed or with a few scattered prickles, glabrous or very sparsely stellate-pubescent; prickles to 3.2 mm long, to 3.6 mm at the base, straight, narrowly triangular with the sides concave from a wide base, yellow-ferruginous, glabrous; trichomes porrect-stellate, sessile, the rays 6–8, ca. 0.1 mm long, the midpoints shorter to equal to the rays, white to yellow in dry material; new growth glabrous to sparsely pubescent with mixture of stellate and minute glandular trichomes, brownish to black in dry material; bark of older stems dark brownish red, glabrous. Sympodial units difoliate, the leaves geminate. Leaves simple, not lobed, the blades of major leaves 19.7–30 cm long, 9.5–16 cm wide, ca. 2 times longer than wide, ovate to elliptic, the minor leaves half as large as or the same size as the major leaves, subcoriaceous, slightly discolorous, unarmed or with a few prickles along the midrib; adaxial surface densely pubescent with a mixture of numerous minute glandular trichomes and a few porrect-stellate trichomes, the glandular hairs to ca. 0.04 mm long, the stellate trichomes with 6–8 rays, 0.1–0.2 mm long, the midpoints shorter to equal to the rays; abaxial surface moderately pubescent with similar sessile porrect-stellate and glandular trichomes; major veins 12–16 pairs, drying light brownish yellow; base short to very long attenuate, oblique; margin entire or slightly wavy; apex subacute to acute, or short acuminate; petioles 1.5–9 cm long, 1/9–1/6 of the leaf blade length, unarmed or occasionally armed with a few broad-based prickles to 3.9 mm long, to 2.5 mm in diameter at the base, straight, yellow-ferruginous, glabrous or with a few stellate trichomes like those of the blades. Inflorescence to 2 cm, apparently lateral, forked or several-branched, with ca. 50 flowers, 5–15 flowers open at any one time, glabrous to moderately stellate-pubescent on the youngest parts, with sessile porrect trichomes like those of the stems; peduncle 4–12.6 mm long, unarmed; pedicels 5.1–18.6 mm long, 0.3–0.4 mm in diameter at the base, 0.7–1.1 mm in diameter at the apex, erect, unarmed, glabrous to moderately stellate-pubescent with porrect trichomes like the inflorescence but often with longer rays, articulated at the base. Buds fusiform, exserted from the calyx before anthesis. Flowers 4–5-merous, apparently all perfect. Calyx with the tube 1.4–1.9 mm long, campanulate, the lobes 1–1.5 mm long, 1.5–1.8 mm wide, deltate, apex acuminate, the abaxial surface more or less strongly keeled along the midvein, unarmed and glabrous or sparsely stellate-pubescent abaxially with a few porrect-stellate trichomes like those of the pedicels. Corolla 1.8–2 cm in diameter, lavender to violet, stellate, lobed ca. 4/5 of the way to the base, the lobes 8.2–10.5 mm long, 2.5–2.9 mm wide, oblong, spreading at anthesis, densely stellate-pubescent abaxially on parts exposed in bud. Stamens equal; anthers 3.7–5.7 mm long, 0.8–1.3 mm wide, connivent, tapering, yellow, glabrous, poricidal at the tips, the pores directed distally, not elongating to slits with drying. Ovary conical, with a few stellate trichomes; style 6.5–9.5 mm long, filiform, curved towards the apex, glabrous; stigma capitate or slightly bilobed. Fruit a globose berry, several to many per infructescence, 0.8–1.2 cm in diameter, orange to red when mature, the pericarp thin and shiny, glabrous; fruiting pedicels 10.8–18.2 mm long, 0.7–0.9 mm in diameter at the base, ca. 1.5 mm in diameter at the apex, erect; fruiting calyx lobes slightly expending to 1.7 cm long, covering 1/4 of the berry or reflexed, glabrous. Seeds ca. 40 per berry, 1.7–2.5 mm long, 2.5–2.8 mm wide, flattened-orbicular to flattened-reniform, yellow-tan to yellow-ferruginous, the surface minutely pitted, the testal cells pentagonal to sinuate in outline. Chromosome number: not known.

**Figure 20.**
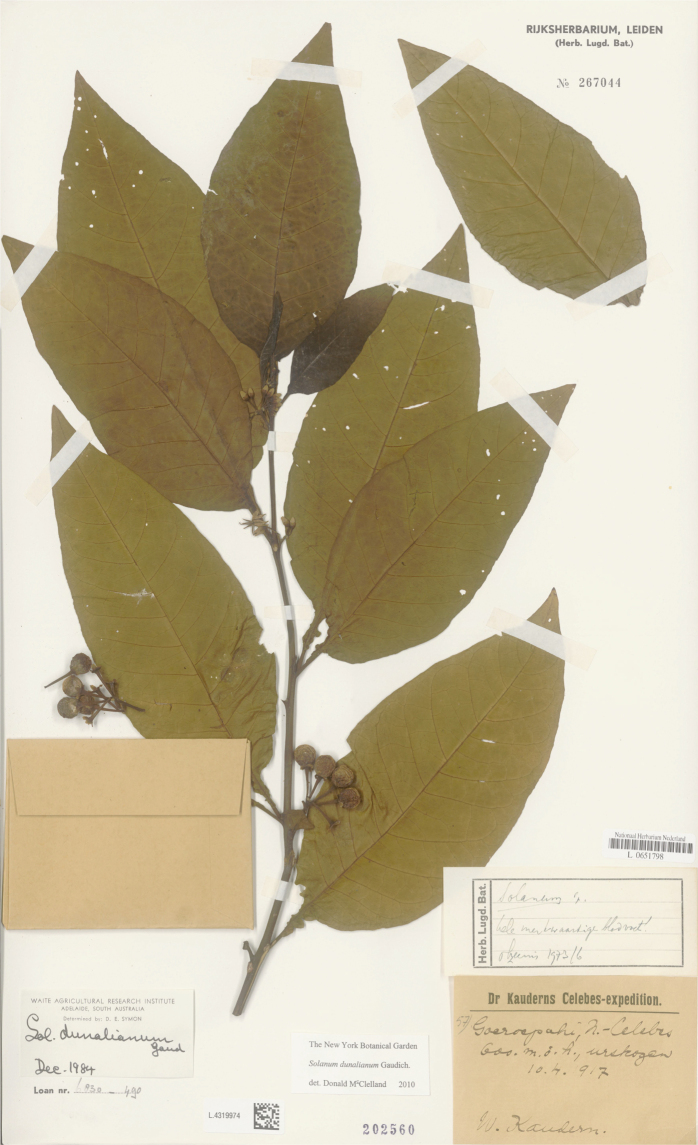
*Solanumdunalianum* Gaudich. Herbarium specimen collected in Indonesia in 1917 (*Kaudern 57*, L.4319974). Photograph credit: Naturalis Biodiversity Center.

#### Distribution

**Figure 21.**
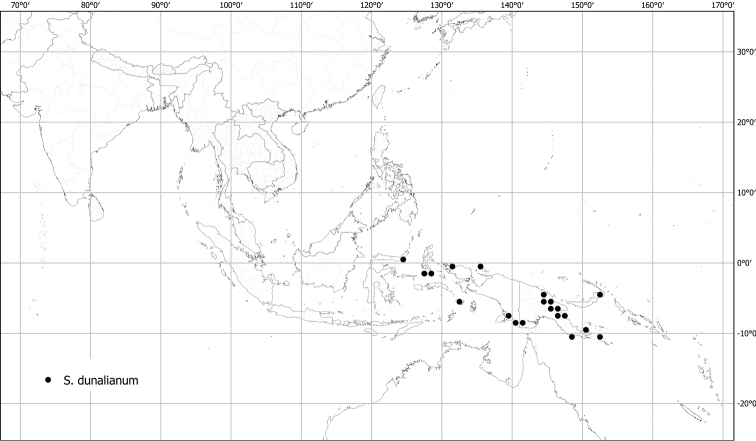
Distribution of *Solanumdunalianum*.

**(Fig. 21).***Solanumdunalianum* is found from Sulawesi east through New Guinea and south to the Cape York Peninsula in Queensland, Australia. It has also been collected in Vanuatu and Tonga (Symon 1985).

#### Ecology and habitat.

*Solanumdunalianum* typically occurs in disturbed habitats and has been found in secondary rainforest, along roads, in clearings, along streams, and gardens; from sea level to 1,200 m elevation (on New Guinea).

#### Common names and uses.

None recorded from the region treated here. Papua New Guinea. Eastern Highlands: gonovise (*Kerenga LAE 56923*); Tonga. Vava‘u: polo jongo (*Soakai 1048*).

#### Preliminary conservation status

**(IUCN 2019).** Least Concern (LC). EOO (887,043 km^2^); AOO (100 km^2^). *Solanumdunalianum* is at the northeastern edge of its range in tropical Asia, the species is common and widely distributed in New Guinea.

#### Discussion.

*Solanumdunalianum* is a species primarily of northern Australia and the island of New Guinea (McClelland 2012) that only just gets into tropical Asia. It was treated as a member of section Dunaliana Bitter by McClelland (2012) along with a number of other Pacific species such as *S.viridifolium* Dunal and *S.labyrinthinum* D.McClelland (see McClelland et al. 2020). *Solanumdunalianum* is one of the few species in this group that has been included in molecular analyses (e.g., Aubriot et al. 2016a) and it resolves as part of the ‘Sahul-Pacific clade’ and member of a group with *S.schefferi* (as *S.lianoides*) and *S.graciliflorum*. Within the area treated here, *S.dunalianum* is distinctive in its branched inflorescence with small red fruits and almost glabrous vegetative parts with very few, if any prickles. Other taxa in the area with several-branched inflorescences are usually densely and variously pubescent (e.g., *S.giganteum*, *S.graciliflorum*, *S.pseudosaponaceum*, *S.torvoideum*).

**Figure 22.**
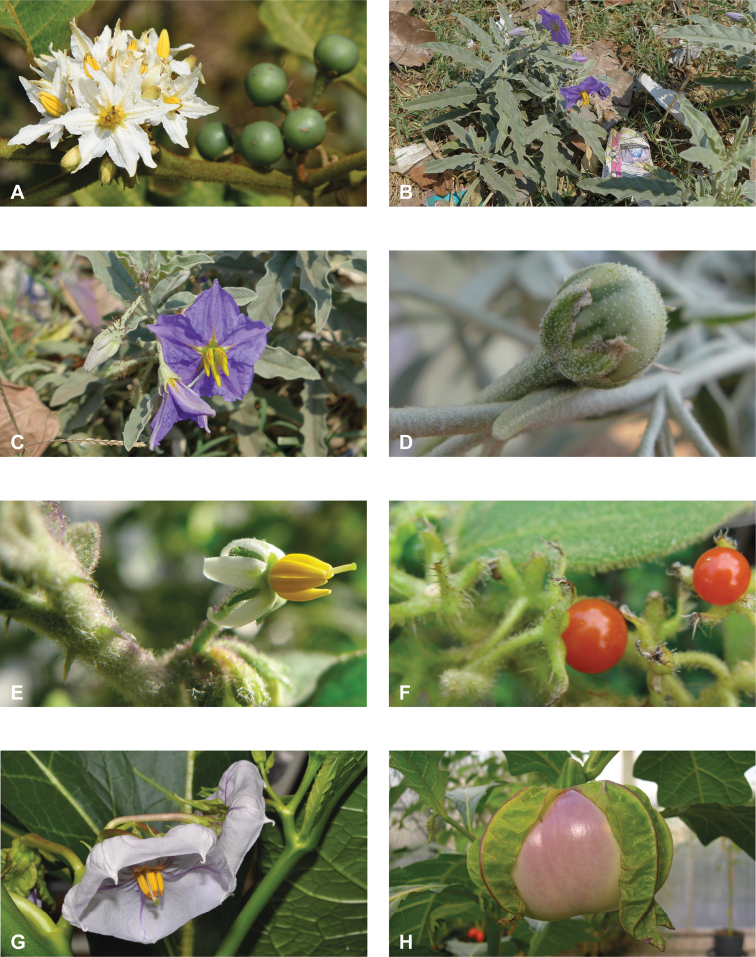
Introduced species of *Solanum.Solanumchrysotrichum* Schltdl. **A** detail of inflorescence (field photograph, unvouchered, India). *Solanumelaeagnifolium* Cav. **B** habit (*Sampath Kumar et al. 126972*, India) **C** detail view of a flower (*Sampath Kumar et al. 126972*, India) **D** detail view of a fruit (field photograph, unvouchered, India). *Solanumjamaicense* Mill. **E** detail view of a flower (*Stern 265*, Trinidad and Tobago) **F** detail view of fruits (*Stern 265*, Trinidad and Tobago). *Solanummacrocarpon* L. **G** detail view of flower (in cultivation at GAFL Avignon, unvouchered) **H** detail view of fruit (in cultivation at Radboud University, Nigmegen, unvouchered, material now at CGN, Wageningen). Photograph credits: **A** S.Knapp **B–D** X. Aubriot **E, F** S. Stern **G, H** S. Knapp.

The two collections from northern Papua New Guinea cited in the protologue of S.dunalianumvar.puberius were destroyed in Berlin, and we have found no duplicates of these, despite extensive searches. Ledermann’s travels are well-documented (Ledermann 1919; van Steenis-Kruseman 1985; Veldkamp et al. 1988), and many of the areas he visited have not been accessed since (Takeuchi and Golman 2002), so we delay neotypifying this name until such collections are available or duplicates of Ledermann’s collections are found.

#### Specimens examined.

See Suppl. materials 1–3.

### ﻿13. Solanum elaeagnifolium

Taxon classificationPlantaeSolanalesSolanaceae

Cav., Icon. 3: 22, tab. 243. 1795.

184E8145-15FC-5D5C-97DA-7D7C51C26247

Fig. 20B–D No names based on tropical Asian types have been published; for complete synonymy see Knapp et al. 2017.

#### Type.

Cultivated in Madrid from “America calidiore” [“del viaje de los espanoles alrededor del mundo, Cult. en el R. J. Bot. 1793”], *Anonymous s.n.* (lectotype, designated by Knapp 2007, pg. 198: MA [MA-476348-2]; isolectotype: MA [MA-476348-1).

#### Description.

Knapp et al. (2017: 22–46); http://www.solanaceaesource.org/solanaceae/solanum-elaeagnifolium.

#### Distribution.

*Solanumelaeagnifolium* has been rather widely collected in India (states of Karnakata, Maharashtra, Rajasthan, Tamil Nadu, and Uttar Pradesh); it is native to North and South America but widely adventive and invasive elsewhere (see Knapp et al. 2017).

#### Discussion.

Although we have only seen *S.elaeagnifolium* specimens from India and Pakistan to date, we suspect this invasive species will become more widespread with time. *Solanumelaeagnifolium* is a plant of dry habitats and can become established easily through vegetative reproduction. It is easy to distinguish from native spiny solanums in tropical Asia by its silvery pubescence of lepidote trichomes and relatively narrowly elliptic leaves. It can form large stands in disturbed areas via rhizomes.

#### Specimens examined.

See Suppl. materials 1–3.

### ﻿14. Solanum forskalii

Taxon classificationPlantaeSolanalesSolanaceae

Dunal, Hist. Nat. Solanum 237. 1813.

1BBAC478-CE81-51B4-8EB1-632CD8BCD13D

Fig. 23

Solanum villosum Forssk., Fl. Aegypt.-Arab. 47. 1775, nom. illeg. non Solanumvillosum Mill. Type. Yemen. Wadi Surdud, Feb 1763, *Herb. P. Forsskål 414* (holotype: C [C10003107]).
Solanum macilentum A.Rich., Tent. Fl. Abyss. 2: 105. 1850. Type. Ethiopia. Choho, *R. Quartin-Dillon & A. Petit s.n.* (lectotype, designated by Lester 1997, pg. 286: P [P000343696]; isolectotypes: P [P0003436967, P000343698]).
Solanum albicaule Kotschy ex Dunal, Prodr. [A. P. de Candolle] 13(1): 204. 1852. Type. Sudan. Kordofan: Nubia, Cordofan Chursi, 29 Dec 1839, *C. Kotschy 309* (lectotype, designated by Lester 1997, pg. 287: G-DC [G00145871]; isolectotypes: B, BM [BM000778322], E [E00193284], ER, GOET, K [K000414013, K000414014, K000414015], LZ, M [M0105605, M0105606], MO [acc. # 3942648], MPU, NY, P [P00344693, P00344694, P00344695], STU [STU000027], TCD [TCD0000843], W [acc. # 1889-0293762, acc. # 0000634], WAG [WAG0003359]).
Solanum heudelotii Dunal, Prodr. [A. P. de Candolle] 13(1): 205. 1852. Type. Senegal. near Gabor, 1839, *J. Heudelot 417* (*45*) (lectotype, designated by Lester 1997, pg. 287: P [P00344004]; isolectotypes: K [K000414051], MPU, P [P00344005, P00344006], UPS).
Solanum hadaq Deflers, Bull. Soc. Bot. France 43: 122. 1896. Type. Yemen. Schoukra, Bilad Fodhli, J. Areys, 30 k a’ l E.N.E. de Schughra, 150 m, 22 Mar 1890, *M. Deflers 377* (lectotype, designated by Vorontsova and Knapp 2016, pg. 159: P [P00051780]; isolectotypes: K [K000441183], P [P00051781, P00051782).
Solanum scindicum Prain, J. Asiat. Soc. Bengal, Pt. 2, Nat. Hist. 65: 542. 1896. Type. Pakistan. Sind: Scinde, *T. Cooke s.n.* (lectotype, designated by Vorontsova and Knapp 2016, pg. 159: CAL [CAL0000018701]; isolectotype: K [K000441223]).
Solanum albicaule Kotschy ex Dunal var. parvifrons Bitter, Repert. Spec. Nov. Regni Veg. Beih. 16: 102. 1923. Type. South Sudan. “Nördliches Darfur, El Fascher”, *J.D.C. Pfund 407* (syntypes: B, destroyed, Z, not found; no additional duplicates found).

#### Type.

Based on (replacement name for) *Solanumvillosum* Forssk.

#### Description.

Erect or scandent shrub, 0.5–1 m, prickly. Young stems slender, ascendent to erect, densely stellate-pubescent and prickly, the pubescence of porrect, sessile or occasionally stalked trichomes, the stalks to 0.15 mm long, the rays 6–10, 0.15–0.3(–0.5) mm long, the midpoints same length as the rays or up to 1.5 mm, occasionally rounded, the prickles 3–10 mm long, 1–3 mm wide at the base, straight, occasionally curved, flattened, strongly reflexed, pale yellow to brown, spaced 1–10 mm apart; bark of older stems densely stellate-pubescent, sometimes glabrescent, brown to grey or orange-grey. Sympodial units difoliate, not geminate. Leaves simple, entire to weakly lobed, the blades 1–4(–6) cm long, 0.5–3(–4) cm wide, 1–2 times longer than wide, ovate, membranous to chartaceous, drying concolorous to discolorous, yellow-green to grey-green or brown-green, glabrescent to moderately stellate-pubescent on both surfaces, with porrect, sessile, sometimes stalked trichomes, the stalks to 0.2 mm long, the rays 6–10, 0.2–0.4(–1) mm long, the midpoints ca. same length as the rays, sometimes to 1.5 mm long, with 0(-3) prickles on both surfaces; the primary veins 2–3 pairs, the tertiary venation not visible to the naked eye; base cordate to rounded, the lobes 2(-3) on each side, 0.2–0.4 cm long, extending to 1/4 of the distance to the midvein, broadly deltate, apically rounded to obtuse; apex rounded to obtuse; petiole 0.2–1.6(–2.5) cm long, 1/4–2/3 of the leaf blade length, densely stellate-pubescent, with 0(-5) prickles. Buds ellipsoid, the corolla strongly exserted from the calyx before anthesis. Inflorescences apparently terminal or lateral, 2–6.5 cm long, unbranched or forked, with (1–)2–20 flowers, 1–10 flowers open at any one time, densely stellate-pubescent, with 0(-5) prickles; peduncle 0.1–0.4(–1.5) cm long; pedicels 0.2–1 cm long, erect, articulated at the base, densely stellate-pubescent, with 0(-6) prickles; pedicel scars spaced 0.5–1.5 mm apart. Flowers 5-merous, apparently all perfect. Calyx 2–4.5 mm long, the lobes 0.5–2 mm long, ca. 1 mm wide, deltate, apically acuminate, moderately stellate-pubescent, with 0(-5) prickles. Corolla 1.3–2.4 cm in diameter, mauve to purple, stellate, lobed ca. 4/5 of its length, the lobes 6–11 mm long, 1.5–2 mm wide, deltate, reflexed or spreading, stellate-pubescent abaxially, the trichomes porrect, sessile or stalked, the stalks to 0.15 mm, the rays 4–10, 0.1–0.2 mm long, the midpoints shorter than the rays or to 0.5 mm long. Stamens equal; filament tube ca. 0.1 mm long; free portion of the filaments 0.7–1 mm long; anthers 4.5–7 mm long, connivent to spreading, tapering, yellow, poricidal at the tips, the pores directed distally. Ovary glabrous; style 0.9–1.2 cm long, slender, curved at the apex, glabrous. Fruit a globose berry, 1–10 per infructescence, 0.6–0.9 cm in diameter, the pericarp smooth, orange to red at maturity; fruiting pedicels 0.7–1.6 cm long, 0.3–0.4 mm in diameter at the base, 2–2.5 mm in diameter at the apex, spreading to somewhat deflexed from the weight of the fruit, with 0(-10) prickles; fruiting calyx not accrescent, covering 1/4(–1/2) of the mature fruit, with 0(-5) prickles. Seeds ca. 5–15 per berry, 2.5–4 mm long, 1.8–3 mm wide, flattened-reniform, almost black, the testal cells somewhat sinuate in outline. Chromosome number: n = 12 (Khatoon and Ali 1993), 2n = 24 (Al Wadi 2002).

**Figure 23.**
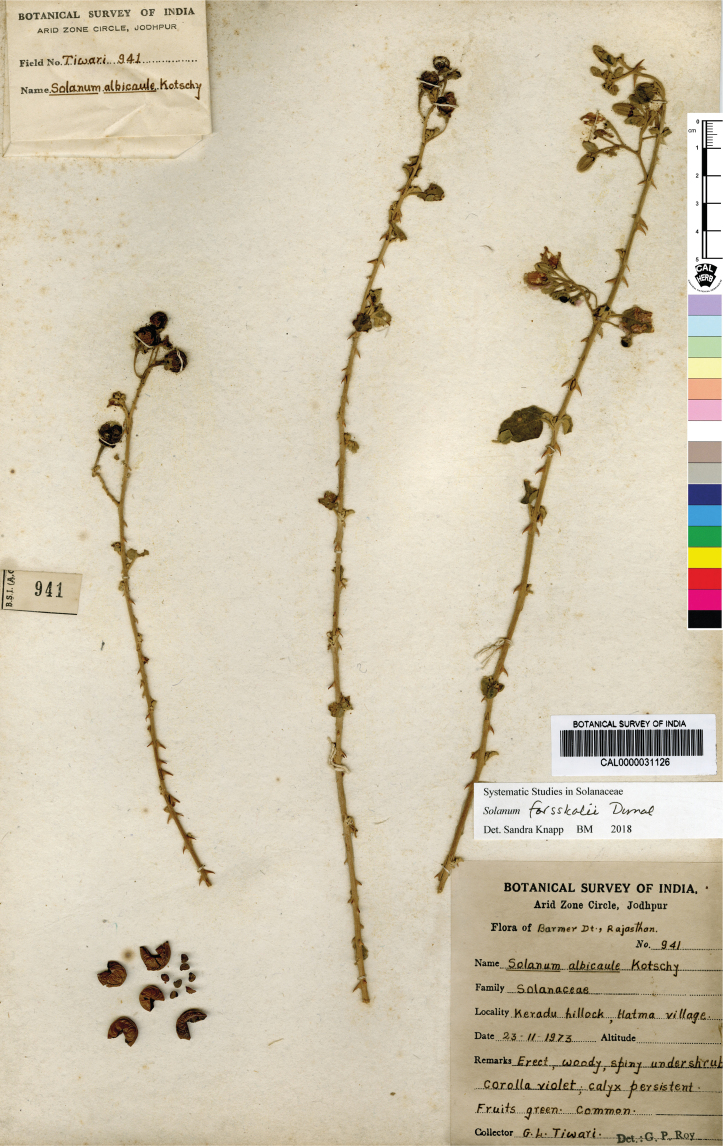
*Solanumforsskalii* Dunal. Herbarium specimen collected in India in 1973 (*Tiwari 941*, CAL0000031126). Photograph credit: © The Director, Botanical Survey of India, Kolkata.

#### Distribution

**Figure 24.**
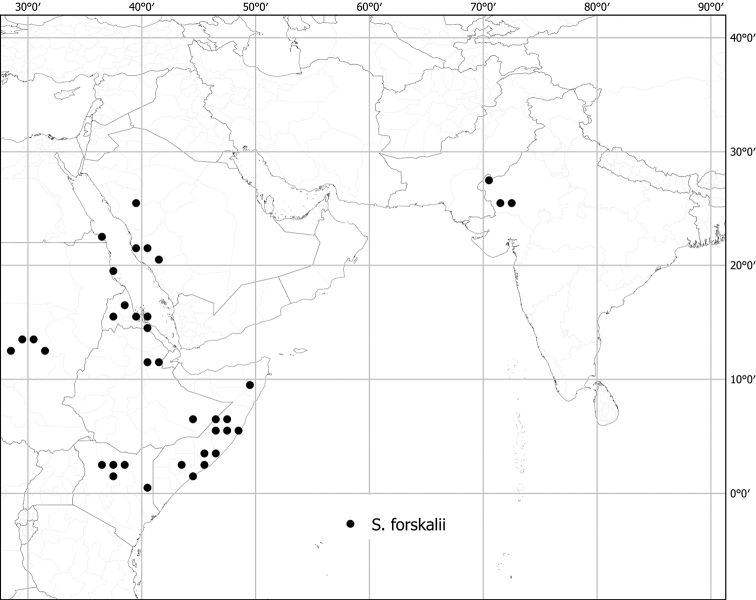
Distribution of *S.forsskalii*.

**(Fig. 24).***Solanumforskalii* is found from northeastern Africa and Arabian Peninsula to Pakistan and western India (Rajasthan and Gujarat).

#### Ecology and habitat.

*Solanumforskalii* is a desert plant growing on stony ground and rocky slopes, often on granite, elevation not recorded on herbarium sheets we have seen from India (from sea level to 2,000 m elevation fide Vorontsova and Knapp 2016)

#### Common names and uses.

India. Rajasthan: nar-kanta [Hindi] (Singh 1991, as *S.albicaule*).

#### Conservation status

**(Knapp 2021b).***Solanumforskalii* has been formally assessed as least concern (LC; https://www.iucnredlist.org/species/101526207/101526210).

#### Discussion.

Although Vorontsova and Knapp (2016) suggested collections of *S.forskalii* from India might be accidental introductions, it is reported as fairly common in the Indian state of Rajasthan (Singh 1991). It is easily recognised by its white-coloured stems, resulting from the dense pubescence of overlapping stellate trichomes with long, delicate rays, and abundant, recurved prickles.

*Solanumforskalii* can be distinguished from *S.cordatum* by its porrect trichomes with 6–10 rays over 0.15 mm long on the young stems (versus trichomes with 12–18 rays under 0.15 mm long on the young stems of *S.cordatum*), 1–20 flowers per inflorescence (versus 1–2 flowers per inflorescence in *S.cordatum*), and anthers 4.5–7 mm long (versus anthers 3–5 mm long in *S.cordatum*). The trichome rays of *S.forskalii* are much thinner and more delicate than those of *S.cordatum*, which appear stout and almost lepidote. There is considerable variability within *S.forskalii* in Africa with respect to inflorescence length, prickle size and trichome midpoint length (Vorontsova and Knapp 2016).

#### Specimens examined.

See Suppl. materials 1–3.

### ﻿15. Solanum giganteum

Taxon classificationPlantaeSolanalesSolanaceae

Jacq., Collectanea 4: 125. 1791.

9F74F65E-374D-56DE-9E37-7CE273967E54

Figs 1A , 25

Solanum niveum Vahl, Symb. Bot. 2: 41. 1791. Type. South Africa. “Cape”, sin. loc., *C. Thunberg s. n.* (holotype: UPS-THUNB [microfiche 1036, no. 5209]).
Solanum farinosum Wallich in Roxburgh, Fl. Ind. (Carey & Wallich ed.) 2: 255. 1824. Type. India. “Babobad”, *Herb. Heyne s. n.* (lectotype, designated by Vorontsova and Knapp 2016, pg. 163: K [K000658975]).
Solanum farinaceum Griffith, Itin. Pl. Khasyah Mts. 111. 1848. Type. Bhutan. Dewanagiri, 1837, *W. Griffith 148* (no specimens cited; no original material found at K).
Solanum giganteum var. tenuifolium Dunal, Prodr. [A. P. de Candolle] 13(1): 259. 1852. Type. India. Tamil Nadu: Madras State, Nilgiri Hills [“Nellighery”], Nedaubetta, 1840, *G. S. Perrottet 520* (lectotype, designated by Vorontsova and Knapp 2016, pg. 163: P [P00049799]; isolectotype: P P00049800]).
Solanum giganteum var. longifolium Dunal, Prodr. [A. P. de Candolle] 13(1): 259. 1852. Type. India. Sin. loc., *Anonymous s. n. [Wallich cat. 2610*] (lectotype, designated by Vorontsova and Knapp 2016, pg. 163: G-DC [G00145950]).
Solanum bequaertii De Wild., Repert. Spec. Nov. Regni Veg. 13: 141. 1914. Type. Democratic Republic of the Congo. Katanga: Shaba Prov., Lubumbashi [Elisabethville], 2 Mar 1912, *J. Bequaert 219* (holotype: BR [BR0000008994356]; isotype: BR [BR0000008993045]).
Solanum sordidescens Bitter, Bot. Jahrb. Syst. 57: 260. 1921. Type. Tanzania. Lindi: Kilwa-Kiwindje Distr., Tschumo, Matumbi Mts., 250 m, Jul 1903, *W. Busse III 3097* (lectotype, designated by Vorontsova and Knapp 2016, pg. 164: EA [EA000001237]; isolectotypes: BM [BM001070317], BR [BR0000006495435]).
Solanum seretii De Wild., Miss. Em. Laurent, 1: 439, tab. 122. 1907. Type. Democratic Republic of the Congo. Orientale: Kisangani, Bima to Bambili, 25 Oct 1905, *F. Seret 166* (holotype: BR [BR0000008993465]; isotype: BR [BR0000008993168]).
Solanum muansense Dammer, Bot. Jahrb. Syst. 48: 243. 1912. Type. Tanzania. Mwanza: Mwanza, May 1892, *F. L. Stuhlmann 4504* (type: B?, destroyed; no duplicates found).

#### Type.

Cultivated in Vienna, original material from South Africa, Cape of Good Hope, *N.J. Jacquin s.n.* (lectotype, designated by Vorontsova and Knapp 2016, pg. 163: W [acc. # 0000608]; isolectotype: W [acc. # 0000609]).

#### Description.

Erect shrub to small tree, to 4 m tall, armed or occasionally unarmed. Stems erect, terete, prickly, densely stellate-pubescent; prickles to 7 mm long, to 5 mm wide at the base, straight, occasionally curved, deltate, orange-brown to almost white, glabrescent to stellate-pubescent in the lower 3/4; pubescence of stalked multangulate-stellate trichomes, the stalks to 0.7 mm long, the rays 10–25, 0.05–0.2 mm long, the midpoints ca. same length as the rays; new growth densely stellate-pubescent with a pale whitish grey pubescence; bark of older stems glabrescent to densely stellate-pubescent, light brown. Sympodial units plurifoliate, the leaves usually not geminate, if geminate the leaves of a pair differing slightly in size but not in shape, in addition often with small stipule-like leaves along the stem. Leaves simple, entire, the blades 12–40 cm long (if geminate the minor blades usually ca. 3 cm long), (3–)4–13 cm wide, ca. 2.5 times longer than wide, elliptic, chartaceous, strongly discolorous, unarmed; adaxial surface drying green-brown to red-brown, glabrescent; abaxial surface drying light grey-green, moderately to densely stellate-pubescent with multangulate stalked trichomes, the stalks to 0.3 mm long, the rays 8–25, 0.1–0.2 mm long, the midpoints ca. same length as the rays; major veins 8–12 pairs, the finer venation visible on both surfaces; base cuneate; margins entire; apex acute to acuminate; petiole 1.5–5.5 cm long, 1/8–1/4 of the leaf blade length, weakly to densely stellate-pubescent with multangulate trichomes like those of the blades, unarmed. Inflorescences 5–13(–20) cm long, apparently terminal or lateral, several times branched, with 30–150 flowers, 10–30 flowers open at any one time, weakly to densely stellate-pubescent with multangulate trichomes like those of the stems, unarmed; peduncle 20–60 mm long, unarmed; pedicels 0.4–1.5 cm long, 1–1.2 mm in diameter at the base, 2–2.5 mm in diameter at the apex, erect to recurved, unarmed, moderately to densely stellate-pubescent with multangulate trichomes like those of the inflorescence axes, articulated at the base; pedicel scars unevenly spaced 1.5–10 mm apart. Buds ovoid, the corolla strongly exserted from the calyx tube before anthesis. Flowers 4–5-merous, apparently all perfect. Calyx with the tube 2.5–3 mm long, conical, the lobes 0.5–2.5(–4) mm long, 1.3–2.2 mm wide, deltate, apically obtuse to acute, unarmed, moderately to densely stellate-pubescent abaxially with multangulate trichomes like those of the rest of the plant, these often deciduous. Corolla 0.8–1.5 cm in diameter, usually mauve, sometimes white, stellate, lobed 2/3–3/4 of the way to the base, the lobes 3.5–6 mm long, 1.5–2.5 mm wide, deltate, spreading or not opening fully, moderately stellate-pubescent abaxially, the trichomes porrect, sessile or stalked, the stalks to 0.1 mm, the rays 8–15, 0.05–0.15 mm long, the midpoints ca. same length as the rays. Stamens equal; anthers 2.5–3 mm long, ca. 1 mm wide, connivent, tapering, yellow, glabrous, poricidal at the tips, the pores not elongating to slits with drying; filament tube ca. 1 mm long, glabrous; free portion of the filaments 0.4–0.8 mm long, glabrous; Ovary conical, glabrous; style 0.45–0.65 cm long, slender, straight or gently curved, glabrous; stigma small capitate. Fruit a globose berry, many per infructescence, 0.6–0.8 cm in diameter, the pericarp smooth, evenly green when young, bright red at maturity, glabrous; fruiting pedicels 0.8–1.6 cm long, 0.5–1.2 mm in diameter at the base, 1.2–2 mm in diameter at the apex, woody, erect, unarmed; fruiting calyx lobes not elongating, ca. 1/3 the length of the mature fruit, reflexed, unarmed. Seeds ca. 15–30 per berry, 2.3–2.9 mm long, 1.5–2.5 mm wide, flattened-reniform, dull yellow to orange-brown, the surface minutely pitted, testal cell margins sinuate. Chromosome number: not known.

**Figure 25.**
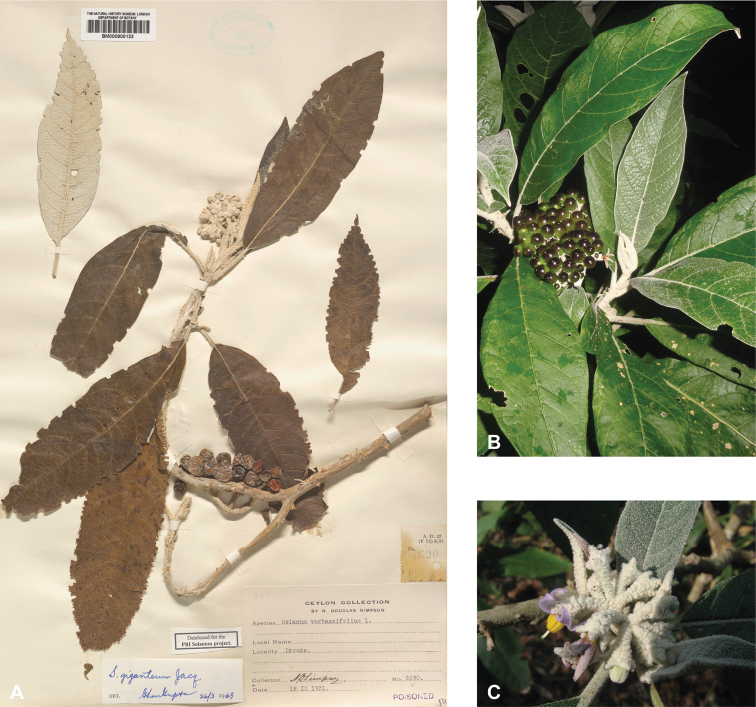
*Solanumgiganteum* Jacq. **A** herbarium specimen collected in ‘Brooks’ (Sri Lanka) in 1931 (*Simpson 8890*, BM000900133) **B** detail of leaves and infructescence (field photograph, unvouchered, India) **C** detail of an inflorescence (field photograph, unvouchered, India). Photograph credits: **A** CC-BY, © copyright The Trustees of the Natural History Museum, London **B** P. Kumar **C** A. Prashant.

**Figure 26.**
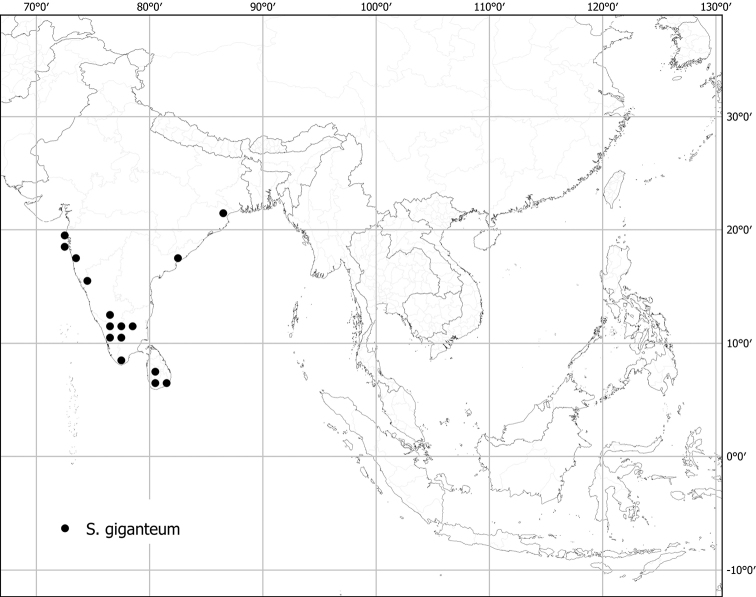
Distribution of *S.giganteum*.

#### Distribution

**(Fig. 26).***Solanumgiganteum* is a widespread species occurring from Sri Lanka and southern India and throughout eastern and southern Africa (but is absent from western Africa, see Vorontsova and Knapp 2016). It is recently being recorded as adventive in Australia (http://www.flora.sa.gov.au/) where it has not yet naturalised.

#### Ecology and habitat.

*Solanumgiganteum* is a weedy species growing at the edges of forests and re-growth in clearings, disturbed areas, sometimes in rocky places or open grasslands; from sea level to 2,100 m elevation.

#### Common names and uses.

English common names for *S.giganteum* in large parts of India are red bitter berry or healing-leaf tree; other local names in India are kurti [Marathi], paintilkkakkoti, paintilikam, peyccuntai-p-palam [Tamil] (Ved et al. 2016, envis.frlht.org); Kerala: cheruchunda (Ved et al. 2016, envis.frlht.org); Tamil Nadu: sambal kilurai [Tamil] (Matthew 1983); peyccundai, anaiccundai [Tamil] (Nair and Nayar 1987), putharichunda, sambal kilurae [Tamil] (Henry et al. 1987).

*Solanumgiganteum* is planted as a shade tree and widely used medicinally in Africa for treatment of sores and ulcers (Hutchings et al. 1996) and in folk medicine in India (Ved et al. 2016).

#### Conservation status

**(IUCN 2019).***Solanumgiganteum* has been formally assessed as LC (Least Concern) due to its wide range and weedy nature and is so listed on the IUCN Red List (Botanic Gardens Conservation International (BGCI) and IUCN SSC Global Tree Specialist Group 2020).

#### Discussion.

*Solanumgiganteum* is a common small tree in the understory of forests in India and Sri Lanka. Its large discolorous leaves accompanied by small stipule-like leaves along the stems, large bunches of bright red berries, deltate stem prickles and farinaceous white pubescence are distinctive and not shared by any other species in the region. The copious pubescence of multangulate trichomes is brittle and powdery, it easily detaches and rubs off leaves and stems, which is the origin of the specific epithet of the synonym *S.farinosum*. *Solanumvagum* of southern India and Sri Lanka is somewhat similar in overall appearance to *S.giganteum*, but has smaller, more lanceolate leaves, smaller inflorescences and less copious, non-deciduous pubescence (see description of *S.vagum*). The broad stem prickles common in young plants of *S.giganteum* are absent in *S.vagum*. Populations of *S.giganteum* in India are generally found to have smaller leaves and inflorescences than those of Africa (Vorontsova and Knapp 2016).

In analyses of phylogeny using molecular data *S.giganteum* is part of a strongly supported monophyletic group with African species *S.anomalum* Thonn., *S.schimperianum* A.Rich., *S.schleibenii* Werderm., *S.schumannianum* Dammer and *S.somalense* Franch. (Vorontsova et al. 2103). Also part of this group is the Indian species *S.pubescens* (Aubriot et al. 2016a).

*Solanumgiganteum* has a long history of cultivation, both in its native range as a shade tree and for fencing and outside of its range in European botanical gardens (see Vorontsova and Knapp 2016). Edmonds (2012) suggests it is native only to the Cape region of South Africa, but we feel this is a biased assessment due to its early introduction from there into Europe.

#### Specimens examined.

See Suppl. materials 1–3.

### ﻿16. Solanum graciliflorum

Taxon classificationPlantaeSolanalesSolanaceae

Dunal, Encycl. [J. Lamarck & al.] Suppl. 3: 763. 1814.

B91622AD-7E19-56FC-B0DD-02E94CFD2D0A

Fig. 27

Solanum athroanthum Dunal, Prodr. [A. P. de Candolle] 13(1): 208. 1852. Type. Indonesia. Java: [Prov. Banjinwanyne] “in sylvis prope Sukaradja” [Sukaraja], 1846, *H. Zollinger 2907* (holotype: G-DC [G00145833]; isotypes: BM [BM000778325], G [G00301684, G003043306], MPU [MPU012648], P [P00368939, P00368940, P00368941]).

#### Type.

Based on an unpublished illustration of Leschenault collection kept in the Node-Véran collection in Montpellier (lectotype, designated by Aubriot et al. 2016b, pg. 100: Sol. Tab. 47 [MPU028534]).

**Figure 27.**
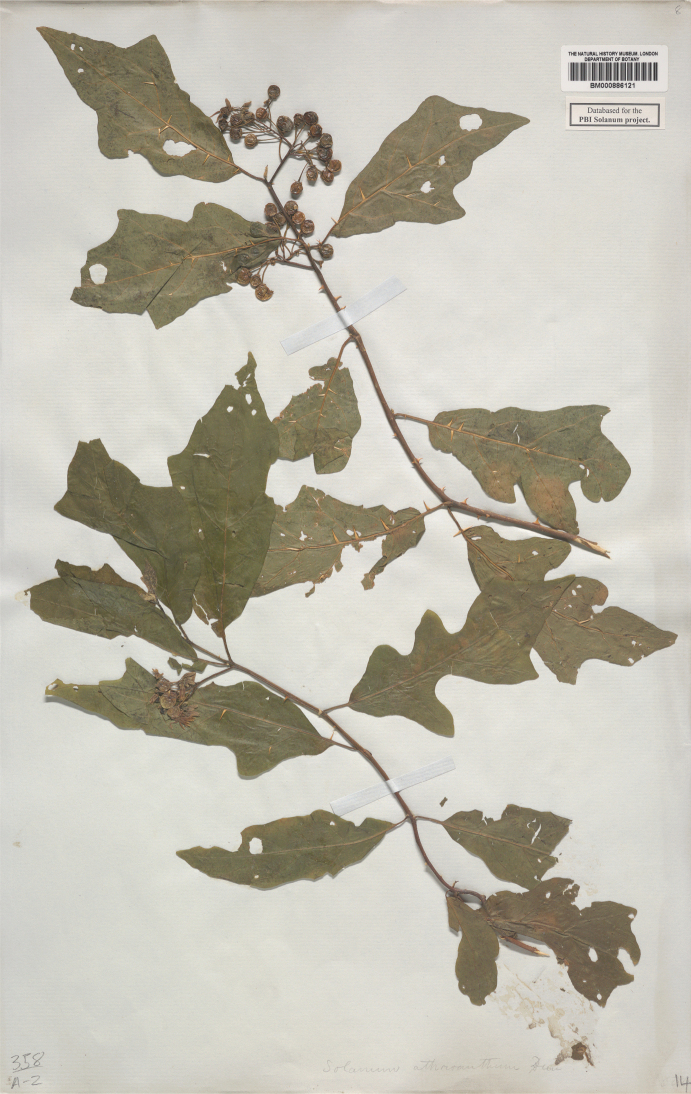
*Solanumgraciliflorum* Dunal Herbarium specimen (epitype) collected in Indonesia (*Horsfield s.n.*, BM000886121). Photograph credit: CC-BY, © copyright The Trustees of the Natural History Museum, London.

#### Description.

Scandent shrub to 2 m tall, armed. Stems apparently erect to decumbent, prickly and very sparsely stellate-pubescent; prickles to 7 mm long, to 8 mm wide at the base, curved, deltate, laterally flattened, pale yellow, glabrous; trichomes porrect-stellate, sessile to subsessile, the rays (4–)5–8, 0.1–0.25 mm long, the midpoints to 0.15 mm long; new growth sparsely stellate-pubescent; bark of older stems dark brownish grey, glabrescent. Sympodial units difoliate, the leaves geminate, usually similar in size. Leaves simple, more or less deeply lobed, the blades (4.5–)7–11 cm long, (1.5–)3–5 cm wide, ca. 2 times longer than wide, elliptic to ovate, chartaceous, slightly discolorous, usually prickly with 1–10(–12) prickles per leaf side, mostly along the midvein, to 9 mm long, to 2 mm wide at the base, straight or slightly curved at the tip, awl-shaped, conical, pale yellow, glabrous; adaxial and abaxial surfaces sparsely to very sparsely stellate-pubescent with sessile to subsessile porrect-stellate trichomes, the rays 6–8, 0.1–0.25 mm long, the midpoint to 0.25 mm long, usually as long as the rays; major veins 3–4 pairs drying dark; base attenuate to truncate; margins shallowly to deeply lobed, the lobes 1–3 on each side, 0.5–2.5 cm long, broadly deltate, apically rounded, the sinuses extending up to 2/3 to the midrib; apex rounded to acute; petiole 0.5–1.8 cm long, 1/10–1/6 of the leaf blade length, unarmed or prickly with 1–2 prickles like those of the blades, sparsely stellate-pubescent with porrect, subsessile trichomes denser at the very base. Inflorescences 2–4 cm long, leaf-opposed or apparently lateral and borne between leaf pairs, unbranched to up to 6 times branched, with 15–50+ flowers, many flowers open at any one time, sparsely to very sparsely stellate-pubescent with trichomes like those of the stems, unarmed; peduncle 1–2(–2.5) cm long, with 0–1 prickles like those of the leaves and stems; pedicels 4–7 mm long, ca. 0.5 mm in diameter at the base, ca. 1 mm in diameter at the apex, erect, unarmed and very sparsely stellate-pubescent with trichomes like those of the stems, articulated at the base; pedicel scars spaced 1–5 mm apart. Buds ellipsoid, strongly exserted from the calyx before anthesis. Flowers 5-merous, apparently all perfect. Calyx with the tube 1–1.5 mm long, campanulate, the lobes 0.25–0.75 mm long, 0.25–0.75 mm wide, deltate, apically acute, unarmed and pubescent abaxially with sessile porrect-stellate trichomes like those of the stems. Corolla 0.5–1 cm in diameter, white to pale lilac, stellate, lobed nearly to the base, the lobes 4–5 mm long, ca. 1 mm wide, narrowly deltate to linear, reflexed at anthesis, glabrous adaxially, densely stellate-pubescent abaxially with sessile porrect trichomes where exposed in bud. Stamens slightly unequal; anthers unequal, three of the five 4.5–5 mm long and two 3–4 mm long, all 0.5–0.75 mm wide, somewhat connivent, tapering, yellow, glabrous, poricidal at the tips, the pores not elongating to slits with drying; filament tube <0.5 mm long, glabrous; free portion of the filaments almost equal, 0.5–1.25 mm long, glabrous. Ovary conical, minutely glandular-puberulent; style ca. 5.5 mm long, slender, curved at the apex, glabrous; stigma capitate, the surfaces minutely papillate. Fruit a globose berry, several to many per infructescence, 0.3–0.5 cm in diameter, red when mature, the pericarp shiny, glabrous; fruiting pedicels 0.8–1.2 cm long, ca. 0.5 mm in diameter at the base, tapering to a slightly enlarged apex, 0.75–1 mm in diameter at the apex, somewhat woody, spreading, unarmed; fruiting calyx lobes slightly expanding to 1.5 mm long, ca. 1/5 the length of the mature fruit, deltate to lanceolate, unarmed. Seeds 6–9 per berry, 3.5–4 mm long, 3–3.5 mm wide, flattened-reniform, orange-brown, the surface minutely pitted, the testal cells pentagonal in outline. Chromosome number: not known.

**Figure 28.**
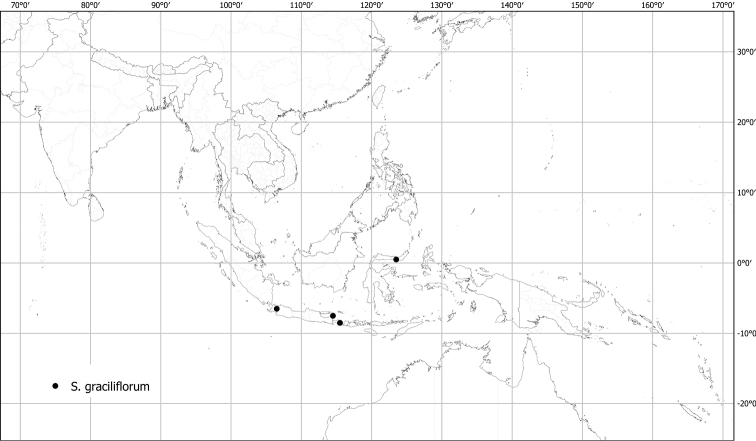
Distribution of *S.graciliflorum*.

#### Distribution

**(Fig. 28).***Solanumgraciliflorum* is known from only a few collections from the islands of Java, Bali, Sulawesi and Ambon (Indonesia).

#### Ecology and habitat.

*Solanumgraciliflorum* is a plant of tropical rainforest understory; elevation not recorded.

#### Common names and uses.

None recorded.

#### Preliminary conservation status

**(IUCN 2019).** Near Threatened (NT). EOO (131,936 km^2^); AOO (16 km^2^). *Solanumgraciliflorum* is known only from seven collections, several of which are of uncertain localities. It has not been re-collected since the first half of the 20^th^ century, indicating it is certainly of conservation concern, thus we suggest a threat status of Near Threatened to reflect this. Additional collection of this species and exploration of the type locality are priorities.

#### Discussion.

*Solanumgraciliflorum* is morphologically similar to the sympatric *S.cyanocarphium* in being a weak, scrambling plant with hooked prickles, but differs from it in its branched inflorescences, smaller flowers and fruits and calyx that is not accrescent in fruit. The inflorescence of *S.cyanocarphium* is unbranched and the calyx strongly accrescent and covers the berry in fruit.

Aubriot et al. (2016b) superfluously lectotypified *S.athroanthum* with the specimen in G-DC (G00145833) but Dunal (1852) clearly cited only this specimen in the protologue, thus making it the holotype.

#### Specimens examined.

See Suppl. materials 1–3.

### ﻿17. Solanum harmandii

Taxon classificationPlantaeSolanalesSolanaceae

Bonati, Bull. Soc. Bot. Genève, sér. 2, 3: 310. 1914.

1695A8F1-995D-59EF-991E-C48F2837B2F6

Fig. 29

#### Type.

Cambodia. Sin. loc., *F.J. Harmand s.n.* (lectotype, designated here: P [P00055939]).

#### Description.

Shrub, size unknown, armed. Stems erect, terete, prickly and densely stellate-pubescent; prickles to 1 cm long, to 0.5 cm wide at the base, straight, deltate, laterally flattened, orange brownish, sparsely to moderately stellate-pubescent in the lower half; trichomes porrect-stellate, mixture of sessile and stalked, the stalks to 0.1 mm long, the rays 5–8, 0.1–0.4 mm long, the midpoints absent or up to 0.1 mm long, with bulbous bases; new growth densely pubescent, light brownish; bark of older stems greyish, moderately stellate-pubescent. Sympodial units plurifoliate, the leaves not geminate. Leaves simple, shallowly lobed, the blades 6–16.5 cm long, 3–10 cm wide, ca. 1.5 times longer than wide, elliptic to ovate, chartaceous, discolorous, unarmed on both surfaces, or occasionally with a few prickles along the midvein; adaxial surface green, moderately to densely stellate-pubescent, the stellate trichomes porrect, sessile to stalked, the stalks to 0.2 mm long, the rays 5–8, 0.1–0.5 mm long, the midpoints to 0.25 mm long; abaxial surface densely whitish stellate-pubescent with trichomes like those of the adaxial surface; major veins 5–7 pairs drying light-green; base shortly attenuate to truncate; margins shallowly lobed, the lobes 3–4 on each side, 0.5–1.5 cm long, broadly deltate, apically rounded, the sinuses less than halfway to the midrib; apex acute; petiole 1–2.5 cm long, 1/10–1/5 of the leaf blade length, unarmed or prickly with 1–3 prickles, densely stellate-pubescent. Inflorescences 2.5–4 cm long, apparently lateral, forked or 2 times branched, with ca. 8–9 flowers, 2 flowers open at any one time, densely whitish stellate-pubescent, trichomes like those of the stems but with longer stalks, unarmed; peduncle ca. 0.5 cm long, unarmed; pedicels 0.25–0.4 cm long, ca. 1 mm in diameter at the base, ca. 2 mm in diameter at the apex, erect, unarmed, densely whitish stellate-pubescent like the inflorescence axes, articulated at the base; pedicel scars spaced 1.5–3 mm apart. Flowers 5-merous, apparently all perfect. Calyx with the tube ca. 2 mm long, campanulate, the lobes 1–2.5 mm long, 1.5–2 mm wide, deltate with an elongate acute apex, unarmed and densely whitish stellate-pubescent with trichomes like those of the pedicels. Corolla 0.7–1.2 cm in diameter, colour unknown, stellate, lobed 1/2–2/3 of the way to the base, the lobes 5–7 mm long, 2.5–4 mm wide, deltate, spreading at anthesis, glabrous adaxially, densely stellate-pubescent abaxially on parts exposed in bud. Stamens equal; anthers ca. 8 mm long, ca. 1 mm wide, glabrous, not tightly connivent, tapering, orange, glabrous, poricidal at the tips, the pores not elongating to slits with drying; filament tube <0.5 mm long, glabrous; free portion of the filaments ca. 0.5 mm long, glabrous. Ovary conical, with simple glandular hairs in the upper 1/4; style ca. 3.5 mm long, slender, curved at the apex, glabrous; stigma capitate, minutely papillate. Fruits and seeds unknown. Chromosome number: not known.

#### Distribution

**(Fig. 30).***Solanumharmandii* is endemic to Cambodia, but without specific locality.

**Figure 29.**
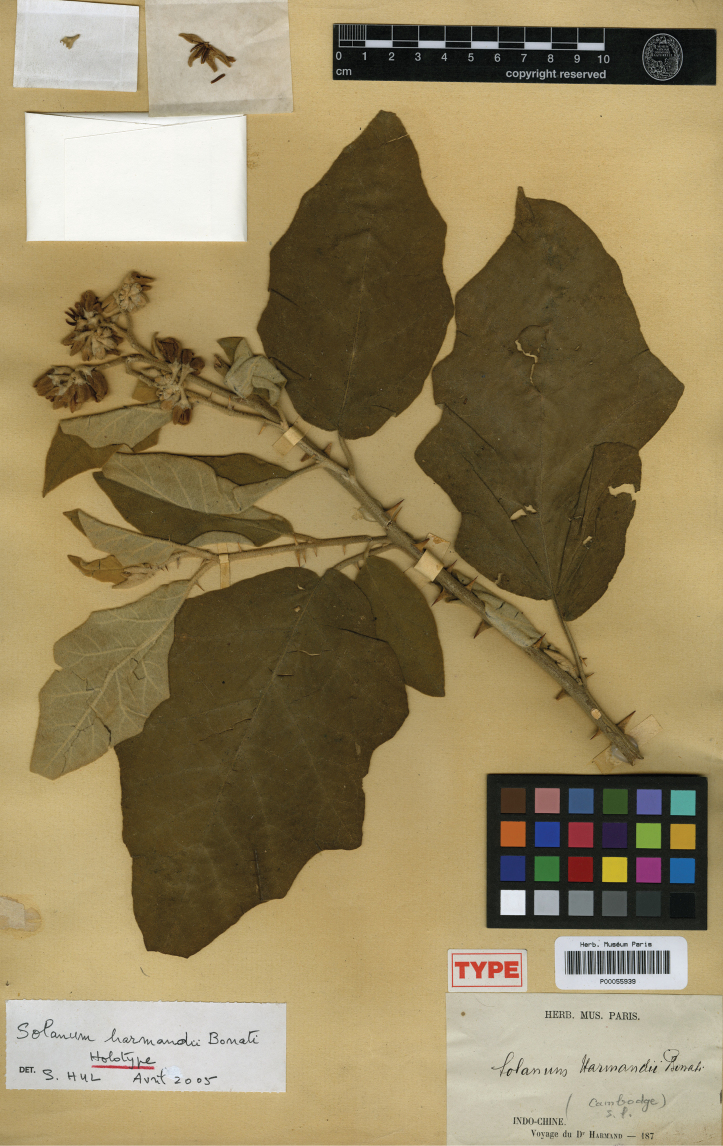
*Solanumharmandii* Bonati Herbarium specimen (lectotype) collected in Cambodia (*Harmand s.n.*, P00055939). Photograph credit: CC-BY, Muséum national d’Histoire naturelle, Paris.

**Figure 30.**
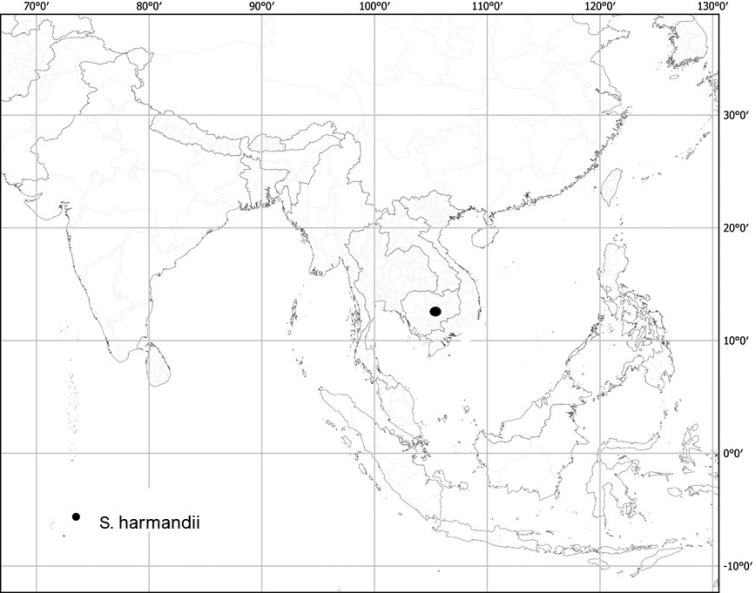
Distribution of *S.harmandii*.

#### Ecology and habitat.

*Solanumharmandii* is known only from the type collection, on which no habitat information was recorded.

#### Common names and uses.

None recorded.

#### Preliminary conservation status

**(IUCN 2019).** Data Deficient (DD). Known only from the type collection, probably collected between 1875 and 1877 (see below). No locality or habitat information was recorded which makes the recollection of this plant a priority.

#### Discussion.

*Solanumharmandii* is a stout shrub that is morphologically very different from all the other spiny solanums treated here. The growth form is reminiscent of members of the Brevantherum clade (sensu Gagnon et al. 2022), but it possesses copious prickles and in flower morphology it is a typical spiny solanum with tapering anthers opening by distally directed pores. *Solanumgiganteum* of the Indian subcontinent has similar overall morphology, with many-branched inflorescences, but differs from *S.harmandii* in its scurfy white pubescence of multangulate trichomes and entire leaves that are usually almost glabrous adaxially.

Hul and Dy Phon (2014) cited “holo-P” in their treatment of *S.harmandii*, but Bonati (1914) cited no herbarium in any part of the publication of this species. Although he was known to have worked in Paris, this does not constitute effective lectotypification. We here designate the single specimen in P (P00055939) as the lectotype. The type specimen was collected during a French expedition led by Dr. Jules Harmand, then medical officer in the region previously known as Indochina, between 1875 and 1877 (Arousseau 1922); detailed itineraries are not available for his collecting trips.

#### Specimens examined.

See Suppl. materials 1–3.

### ﻿18. Solanum hovei

Taxon classificationPlantaeSolanalesSolanaceae

Dunal, Prodr. [A. P. de Candolle] 13(1): 311. 1852.

76B282B2-5833-5C63-8F8A-36E68D81EB9A

Figs 2E , 4B , 31

Solanum jacquemontii Dunal, Prodr. [A. P. de Candolle] 13(1): 287. 1852. Type. India. Sin. loc., *V.V. Jacquemont 563* [593 in protologue] (lectotype, designated here: MPU [MPU858235]; isolectotype: P [P00049910]).

#### Type.

India. Gujarat: Ahmedabad district, Dolca [Dholka] near Sabermatty, *A.P. Hove s.n.* (holotype: BM [BM000900293]).

#### Description.

Subshrubs to shrubs, to 1.5 m tall, armed. Stems erect, terete, prickly, sparsely stellate-pubescent; prickles to 7 mm long, to 3.5 mm in diameter at the base, straight to slightly recurved, broad-based, laterally compressed, light orange to brown, glabrous; pubescence of mixed sessile and short-stalked porrect-stellate trichomes, the stalks ca. 0.3 mm long, the rays 4–8, ca. 0.3–0.4 mm long, the midpoint up to 0.3 mm long; new growth sparsely to densely stellate-pubescent; bark of older stems grey to pale greenish brown, glabrescent. Sympodial units difoliate, the leaves usually geminate. Leaves simple, shallowly lobed, the blades 5–21 cm long, 2–9 cm wide, ca. 2–3 times longer than wide, rhombic to subelliptic, chartaceous, discolorous, armed along the central and occasionally the lateral veins, the prickles to 1 cm long, to 2 mm at the base, straight, flattened, often tinged with purple at the base in live plants; adaxial surface dark green, moderately to sparsely stellate-pubescent, the trichomes porrect, thick-stalked, the stalks to 0.2 mm, the rays 3–8, 0.1–0.4 mm long, the midpoint to 1 mm; abaxial surface light green, moderately to densely stellate-pubescent, trichomes a mixture of porrect and multangulate, sessile or short-stalked, the stalks to 0.4 mm, the midpoint and lateral rays relatively equal in length, ca. 0.3–0.4 mm; major veins 4–6 pairs; base cuneate to short attenuate, somewhat oblique; margins entire to shallowly lobed, the lobes 2–3 on each side, 0.1–0.5 cm long, triangular, broadly acute to obtuse, the sinuses less than 1/4 of the way to the midrib; apex narrowly acute; petiole 0.7–2.5 cm, ca. 1/10–1/5 of the leaf blade length, moderately to densely stellate-pubescent, prickly or sometimes unarmed, the prickles similar to those on the blades. Inflorescences 0.4–4 cm long, extra-axillary, unbranched, with ca. 4–10 flowers, 1–3 flowers open at any one time, moderately to densely stellate-pubescent, glabrescent, with a mix of sessile and short-stalked stellate-porrect trichomes like those of the stems, unarmed; peduncle ca. 0–1.3 cm long, unarmed; pedicels 5–9 mm long, ca. 0.5 mm in diameter at the base, ca. 1 mm in diameter at the apex, spreading at anthesis, unarmed or prickly with a few straight and broad-based prickles, sparsely to moderately pubescent with stellate-porrect trichomes like those of the stems, articulated at the base; pedicel scars spaced 0.5–8 mm apart. Buds ellipsoid to ovoid, exserted from the calyx before anthesis. Flowers 5-merous, apparently all perfect. Calyx with the tube 0.7–2.5 mm long, conical, the lobes 0.5–1.6 mm long, 1.3–2 mm wide, broadly deltate, unarmed or very sparsely prickly with straight to slightly recurved prickles, moderately to densely stellate-pubescent abaxially with porrect-stellate trichomes like those of the pedicels, sometimes purple-tinged on living plants. Corolla 1.2–1.5 cm in diameter, light blue or purple, rotate-stellate, lobed ca. halfway to the base, interpetalar tissue somewhat present, the lobes 4–5.5 mm long, 3–4 mm wide, ovate, spreading at anthesis, sparsely stellate-pubescent along the midvein and on the lower half of lobe adaxially, the hairs sessile, multangulate, moderately to densely stellate-pubescent abaxially, the hairs subsessile, porrect-stellate and multangulate. Stamens equal; anthers 4–5.5 mm long, 0.6–1.2 mm wide, orange or orangish yellow, connivent, glabrous, poricidal at the tips, the pores directed distally, not elongating to slits with drying; filament tube minute, glabrous; free portion of the filaments 0.3–0.4 mm long, glabrous. Ovary oblong-conical, moderately stellate-pubescent towards the apex; style 6–9 mm long, sparsely to moderately stellate-pubescent on proximal 2/3–3/4, the hairs sessile, porrect-stellate or multangulate; stigma subcapitate, the surfaces minutely papillose. Fruit a subglobose berry, 1–5 per infructescence, 0.6–1 m in diameter, orange when ripe, the pericarp thin and shiny, glabrous; fruiting pedicels 1–1.5 cm long, ca. 0.6 mm in diameter at the base, ca. 2 mm in diameter at the apex, strongly deflexed; fruiting calyx not markedly accrescent, the lobes 2–3 mm long, 1–1.5 mm wide, often breaking off in dry material. Seeds 10–20 per berry, 2.8–4.1 mm long, 2.2–3 mm wide, flattened reniform, yellowish brown, the surfaces minutely pitted, the testal cells with slightly sinuate margins. Chromosome number: not known.

**Figure 31.**
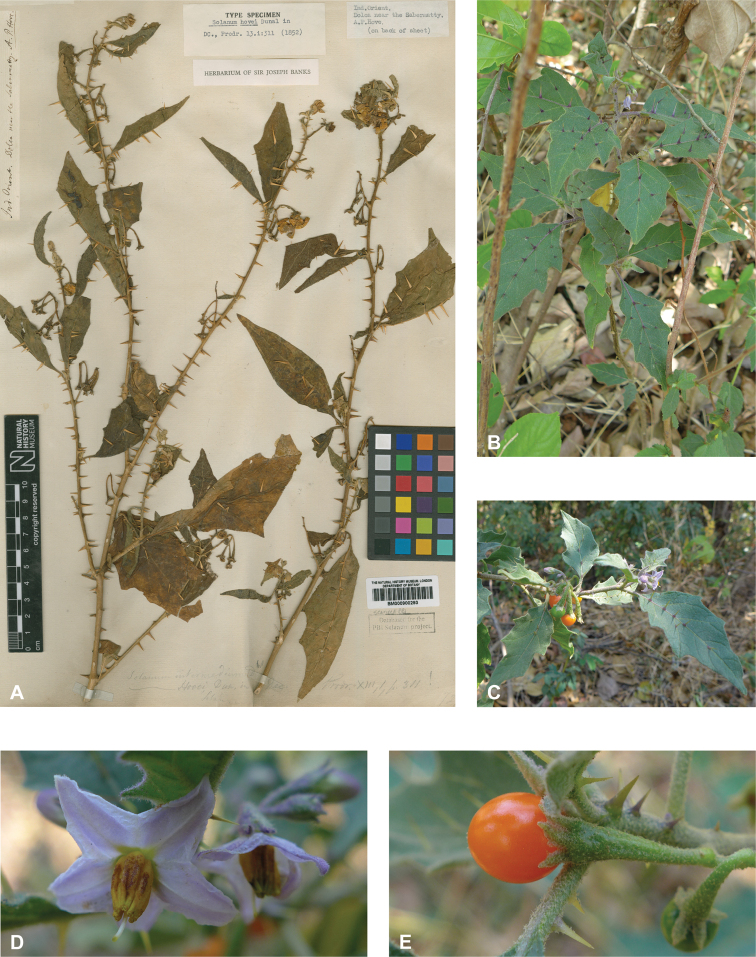
*Solanumhovei* Dunal **A** herbarium specimen (holotype) collected in India (*Hove s.n.*, BM000900293) **B** habit and leaves (field photograph, unvouchered, India) **C** detail of a fertile branch (field photograph, unvouchered, India) **D** detail view of a flower (field photograph, unvouchered, India) **E** detail view of a fruit (field photograph, unvouchered, India). Photograph credits: **A** CC-BY, © copyright The Trustees of the Natural History Museum, London **B–E** X. Aubriot.

#### Distribution

**Figure 32.**
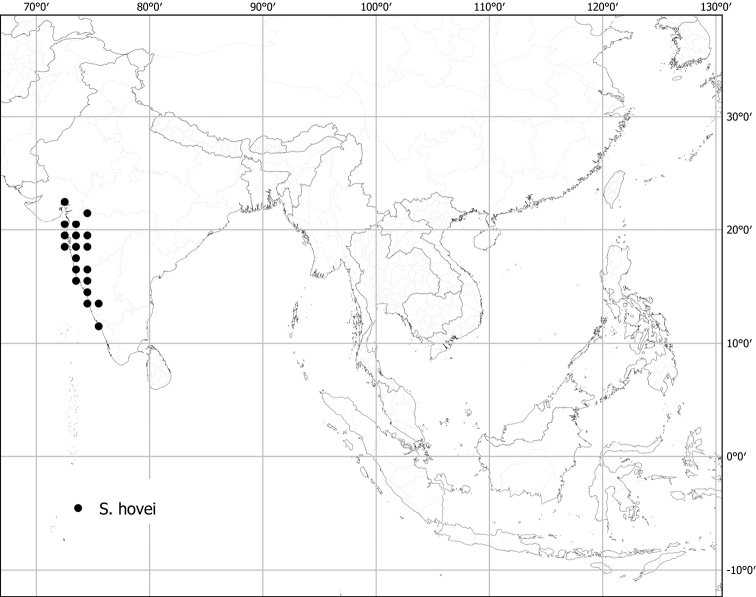
Distribution of *S.hovei*.

**(Fig. 32).***Solanumhovei* is endemic to India and occurs primarily along the Western Ghats range in the states of Goa, Gujarat, Karnataka and Maharashtra with most of the collections of this species having been made in the surroundings of Mumbai and Pune.

#### Ecology and habitat.

*Solanumhovei* grows in open areas on plateaus and along roadsides and forest outskirts from 150 to 1,200 m elevation.

#### Common names and uses.

India. Goa: mothirigani (Naithani et al. 1997).

#### Preliminary conservation status

**(IUCN 2019).** Least Concern (LC). EOO (173,911 km^2^, LC); AOO (564 km^2^, VU). *Solanumhovei* is a relatively common and widely distributed species in the Western Ghats biodiversity hotspot.

#### Discussion.

*Solanumhovei* is a member of a group of species identified in the molecular analyses of Aubriot et al. (2016a) as the ‘S.violaceum group’. It is morphologically similar to the sympatric and widespread *S.violaceum* but differs from it in having leaves with more sparsely pubescent adaxial surfaces and cuneate (rather than acute or truncate) bases. The pedicels of *S.hovei* are strongly deflexed in fruit, while those of *S.violaceum* are spreading. *Solanummultiflorum* of western India also has strongly deflexed pedicels, but they are shorter, and the leaves are densely pubescent with longer trichomes.

Sen Gupta (1964) suggested *S.hovei* was a rare plant in the Western Ghats, but the number of recent collections in local herbaria suggest otherwise. It is probable that *S.hovei* was largely confused with *S.violaceum* in herbaria (usually identified as *S.indicum*).

The protologue of *S.jacquemontii* (Dunal 1852) cites duplicates of “*Jacquemont 593*” in both P and MPU. The specimen we have selected as the lectotype has an annotation with this name in Dunal’s hand and is numbered “*563*”, we are treating this as an error to be corrected in the protologue.

#### Specimens examined.

See Suppl. materials 1–3.

### ﻿19. Solanum insanum

Taxon classificationPlantaeSolanalesSolanaceae

L., Mant. 1: 46. 1767.

78F3F677-984C-5580-ACA0-BD1D3A95FA7C

**Figure 33.**
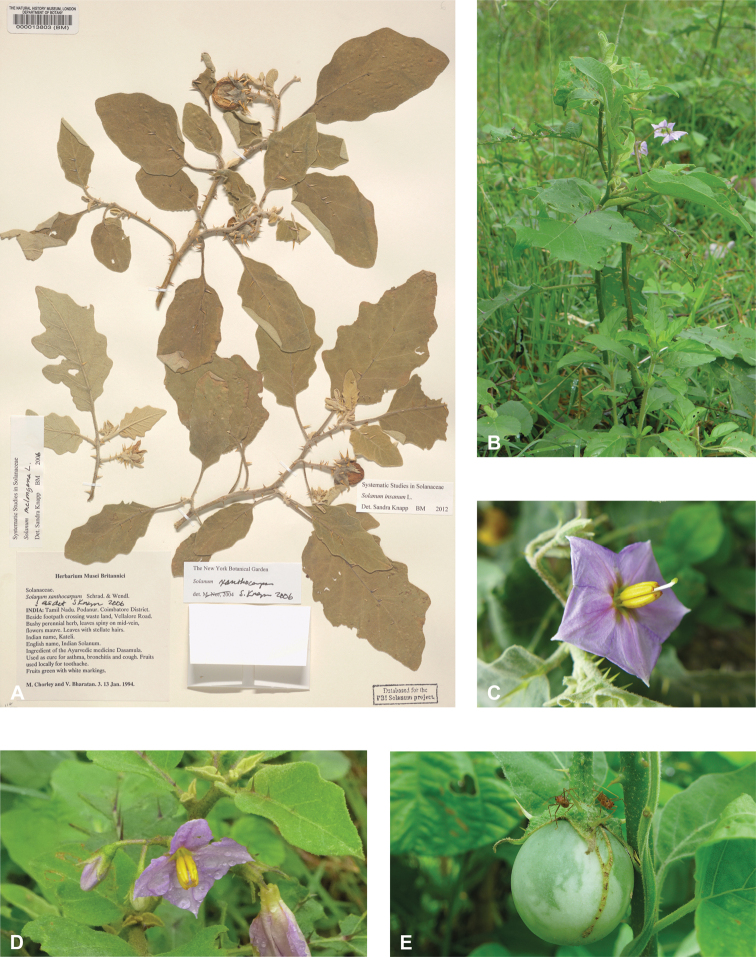
*Solanuminsanum* L. **A** herbarium specimen collected in India in 1994 (*Chorley & Bharatan 3*, BM000013803) **B** habit (*Meeboonya et al. RM 257*) **C** detail view of a hermaphroditic flower (*Meeboonya et al. RM 251*, Thailand) **D** detail view of an inflorescence with an opened staminate flower (*Meeboonya et al. RM 257*, Thailand) **E** detail view of an immature fruit (*Meeboonya et al. RM 277*, Thailand). Photograph credits: **A** CC-BY, © copyright The Trustees of the Natural History Museum, London **B–E** X. Aubriot.

Figs 1F , 3E , 4A , 33

Solanum undatum Lam., Tabl. Encycl. 2: 22. 1794. Type. Mauritius. Sin. loc., “Insula Franciae”, *J. Martin s.n.* (lectotype, designated by Vorontsova and Knapp 2016, pg. 197: P-LA (P00357695, Morton neg. 8392]).
Solanum trongum Poir., Encycl. (Lamarck) 4: 308. 1797. Type. Indonesia. Malaku: “Amboina” [Ambon Island] (no specimens cited; lectotype, designated by Merrill 1917, pg. 463, as “type”: “Trongum agreste spinosum”, Rumphius, Herb. Ambion. 5: 240, t. 86, f.1. 1747).
Solanum gula Buch.-Ham., Trans. Linn. Soc. London 14(2): 267. 1824. Type. India. Karnakata: Kowdhalli “Caudhully [protologue – Mysore]”, Oct 1800, *J. Buchanan-Hamilton s.n.* (lectotype, designated here: BM [BM000900233]).
Solanum himalense Dunal, Prodr. [A. P. de Candolle] 13(1): 300. 1852. Type. India. “Sivala & sub Himala” [protologue: In Himalae et Sivalae montibus altit. 4600 ped., Edgeworth, pl. exs. Ind. n. 116], *M.P. Edgeworth 116* (holotype: MPU [MPU981115]).
Solanum indicum Nees var. pubescens Dunal, Prodr. [A. P. de Candolle] 13(1): 310. 1852. Type. [India]. Sin. loc., *N. Wallich s.n.* [Wallich Catal. 2626g] (lectotype, designated here: G-DC [G00130396]; isolectotype: K-W [K001116650 pro parte, R-hand two stems]).
Solanum melanocarpum Dunal, Prodr. [A. P. de Candolle] 13(1): 355. 1852, nom. illeg. superfl. Type. Based on Solanuminsanum L. (cited in synonymy; “Nomen insanum mutavi, quia adeo non insanum videtur ut potius fructus edules credibilius sunt, quemadmodum nomina insanum et melongean, saepissime fuerunt confusa” [I changed the name insanum because it does not seem harmful, the fruits are probably edible, and insanum and melongena were very often getting confused]).
Solanum melanocarpum Dunal var. atropurpureum Dunal, Prodr. [A. P. de Candolle] 13(1): 356. 1852. Type. Indonesia. Java: sin. loc., *H. Zollinger 698* (lectotype, designated here: G-DC [G00131525]; isolectotypes: G [G00301647], G-DC [G00131526, as *Zollinger 698b*], P [P00379566, P00379567]).
Solanum undatum Lam. var. aurantiacum Dunal, Prodr. [A. P. de Candolle] 13(1): 359. 1852. Type. Cultivated, sin. loc., *Anonymous s.n.* (lectotype, designated by Vorontsova and Knapp 2016, pg. 198: G-DC [G00131519]).
Solanum undatum Lam. var. violaceum Dunal, Prodr. [A. P. de Candolle] 13(1): 359. 1852. Type. Mauritius. Sin. loc., *L. Bouton s.n.* (lectotype, designated by Vorontsova and Knapp 2016, pg. 198: G-DC [G00131506]; isolectotype: G [G00131504]).
Solanum trongum Poir. var. divaricatum Dunal, Prodr. [A. P. de Candolle] 13(1): 361. 1852. Type. Sri Lanka. “Trinquemalay”, *A.A. Reynaud s.n.* (lectotype, designated here: P [P00049887]).
Solanum trongum Poir. var. rumphii Dunal, Prodr. [A. P. de Candolle] 13(1): 361. 1852, nom. illeg. Type. Described using same material as S.trongum Poir. (should be var. trongum)
Solanum trongum Poir. var. sinuatopinnatifidum Dunal, Prodr. [A. P. de Candolle] 13(1): 361. 1852, nom. illeg. Type. Based on Solanumtrongum Poir. [sensu Blume 1826] (should be var. trongum)
Solanum trongum Poir. var. tongdongense Dunal, Prodr. [A. P. de Candolle] 13(1): 361. 1852. Type. Myanmar. “Tong dong”, 7 Jan 1827, *N. Wallich s.n.* [Wallich Catal. Burm. 135] (lectotype, designated here: G-DC [G00131531]; isolectotype: G-DC [G00131371]).
Solanum album Lour. var. gaudichaudii Dunal, Prodr. [A. P. de Candolle] 13(1): 361. 1852. Type. Vietnam. Sin. loc., 1839, “Cochinchina”, *C. Gaudichaud s.n.* (holotype: G-DC [G00131563]).
Solanum cyanocarphium Blume var. obtusangulum Dunal, Prodr. [A. P. de Candolle] 13(1): 362. 1852. Type. Indonesia. Java: Sin. loc., 1837, *J.C. von Hoffmannsegg 119* (lectotype, designated here: G-DC [G00131561]).
Solanum cumingii Dunal, Prodr. [A. P. de Candolle] 13(1): 363. 1852. Type. Philippines. Sin. loc., *H. Cuming 443* (lectotype, designated here: G [G00076245]; isolectotypes: BM [BM000778205], E [E00190694], G [G00076246, G00076247], K [K000195960, K000195961], L [L0003628], LE, ME [MEL2444029], P [P00578602, P00578613], W [acc. # 0000622, acc. # 1889-0084423]).
Solanum immane Hance ex Walp., Ann. Bot. Syst. 3(1): 165. 1852. Type. China. Hongkong: “Hong Kong”, [*Herb.] H.F. Hance 860* (no specimens or herbaria cited; lectotype, designated here: BM [BM000942450]).
Solanum melongena L. var. insanum Prain, Bengal Pl. 746. 1903. Type. Based on Solanuminsanum L.
Solanum mirikense C.R.Mukhop., J. Indian Soc. Bot. 72(1-2): 185. 1993. Type. India. West Bengal: Darjeeling, Mirik, 1700 m, 5 Oct 1985, *C.R. Mukhopadhyay 428A* (holotype: CAL [CAL0000018719]).
Solanum melongena L. var. cumingii (Dunal) J.Samuels, Proc. XV EUCARPIA Mtg. 258. 2013. Type. Based on Solanumcumingii Dunal
Solanum melongena L. subsp. insanum Banfi, Galasso & Bartolucci, Nat. Hist. Sci. 15(1): 19. 2017. Type. Based on Solanuminsanum L.

#### Type.

India. Gujarat: Surat, *Anonymous s.n.* (lectotype, designated by Hepper and Jaeger 1985, pg. 389: LINN [acc. # 248.29]).

#### Description.

Erect shrub, to 1 m tall, armed. Stems erect, terete, prickly, moderately stellate-pubescent to glabrescent; prickles to 8 mm long, to 5 mm wide at the base, straight, flattened, yellow-orange, glabrous; pubescence of stalked porrect-stellate trichomes, the stalks to 0.2 mm long, the rays 6–12, 0.2–0.4 mm long, the midpoints ca. same length as the rays or elongated to 1 mm; new growth densely stellate-pubescent, light brownish in dry material; bark of older stems grey to brown, glabrescent. Sympodial units difoliate, the leaves not geminate. Leaves simple, moderately lobed, the blades 2.5–12 cm long, 1.3–8 cm wide, ca. 1.5 times longer than wide, ovate, chartaceous, concolorous to weakly discolorous, armed with 2–20 prickles on both surfaces, these yellow-tan or purple-tinged; adaxial and abaxial surfaces yellow-green, moderately pubescent, with porrect-stellate trichomes, sessile or stalked, the stalks to 0.2 mm, the rays 5–8, 0.2–0.7(–1) mm long, the midpoints ca. same length as the rays; major veins 3–5 pairs; base truncate, sometimes obtuse; margins lobed, the lobes 2–3 on each side, 0.5–1.2 cm long, broad-deltate, apically rounded, the sinuses less than halfway to the midrib; apex rounded to acute; petiole 0.7–3 cm long, 1/4–1/3 of the leaf blade length, unarmed or prickly with 1–5 prickles, moderately stellate-pubescent to glabrescent, the pubescence with stellate-porrect trichomes like those of the stems. Inflorescences 2.5–3.5 cm long, apparently terminal or lateral, unbranched, with 1–3 flowers, 1 flower open at any one time, moderately to densely stellate-pubescent with porrect trichomes like those of the stems, unarmed; peduncle 0–13 mm long, unarmed or with a few prickles; pedicels 0.8–2.5 cm long, ca. 1 mm in diameter at the base, ca. 2 mm in diameter at the apex, erect, unarmed or with a few scattered prickles, densely stellate-pubescent with porrect trichomes like those of the inflorescence axes, articulated at the base; pedicel scars spaced 1–2 mm apart. Buds ovoid, poorly exserted from the calyx before anthesis. Flowers 5(-6)-merous, heterostylous and the plants andromonoecious, with the lowermost flower(s) long-styled and hermaphrodite, the distal flowers short-styled and staminate. Calyx with the tube 4–5 mm long, campanulate, the lobes 4–6 mm long, 2–2.5 mm wide, deltate, apically acute, unarmed or with 1–15 prickles, densely stellate-pubescent with porrect trichomes like those of the pedicels. Corolla 1.8–2.5 cm in diameter, mauve or white, almost rotate with abundant interpetalar tissue, lobed ca. 1/4 of the way to the base, the lobes ca. 7 mm long, ca. 10 mm wide, broadly deltate, spreading at anthesis, glabrous to sparsely stellate-pubescent adaxially with porrect-trichomes mostly on the midvein and the tips, densely stellate-pubescent abaxially with variously stalked porrect trichomes. Stamens equal; anthers 4–5 mm long, ca. 1 mm wide, tapering, yellow, glabrous, poricidal at the tips, the pores directed distally, not elongating to slits with drying; filament tube ca. 1.5 mm long, glabrous; free portion of the filaments 1–2 mm long, glabrous. Ovary conical to ovoid, stellate-pubescent and glandular in the upper 1/4; style 2–3 mm in short-styled flowers, 5–7 mm long in long-styled flowers, moderately stellate-pubescent in the lower 1/2; stigma capitate or occasionally somewhat bilobed, the surfaces minutely papillose. Fruit a globose berry, 1 per infructescence, 1.5–3 cm in diameter, dark green with pale green and cream markings when young, yellow at maturity, the pericarp smooth, glabrous; fruiting pedicels 1.5–2.2 cm long, 1.5–3 mm in diameter at the base, 4–5 mm in diameter at the apex, unarmed or with a few stout prickles, woody, pendulous; fruiting calyx lobes elongating to 9–15 mm long, 1/4–1/3 the length of the mature fruit, reflexed, with 2–30 prickles. Seeds ca. 50–150 per berry, 2.4–2.8 mm long, 1.8–2.2 mm wide, flattened-reniform, orange-brown, the surface minutely pitted, the testal cells with sinuate margins. Chromosome number: n = 12 (Meyer et al. 2012, as *S.undatum*).

#### Distribution

**Figure 34.**
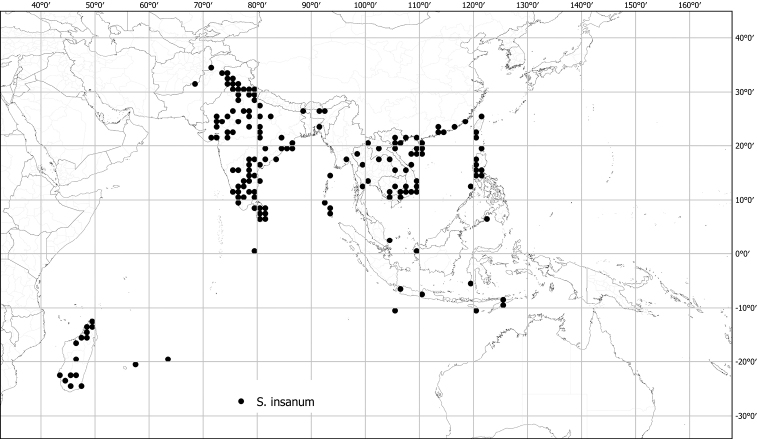
Distribution of *S.insanum*.

**(Fig. 34).***Solanuminsanum* is widely distributed in India and southeastern Asia (east to the Philippines) and is also found as far west as Madagascar and Mauritius (possibly introduced there from Asia for its medicinal properties).

#### Ecology and habitat.

*Solanuminsanum* is a plant of degraded scrubland and secondary vegetation and occurs from sea level to ca. 500 m elevation.

#### Common names and uses.

Cambodia. trâp rôm nhong (Hul and Dy Phon 2014), trôp (*Collard 56*), trôp som nhon (*Collard 32*), China. ye qie (Zhang et al. 1994); Hainan: ngou ke tzi (*Lei 392*). India. Andhra Pradesh: challa mulaga (*Foulkes 83*); Chattisgarh: anpa, jangli bhata (*Kumar CNH-15565*); Rajasthan: bhutkataiya [Hindi] (Singh 1991, as *S.incanum*); Tamil Nadu: thalamoolagah, nullamoolaga (*Wight 1573 Ab*); Uttar Pradesh: bari bhatkatanja (*Bell 863*), jangli bengan (*Sarin NC-5079*). Indonesia. Borneo: terong nasi (*Ambriansyah 687*); Java: terong-glate (*Hoffmansegg 119*). Laos. mok kua kim (*Spire 914*), mok khma lê (*Pottier 263*). Malaysia/Singapore. tĕrong (with various qualifiers, Burkill 1935). Malaysia. Sabah: soguntung [Rungus] (*Jones 152*). Singapore. terong nyonya (*Corner SFN-37775*). Sri Lanka. Uva: ela batu [Sinhala] (*Hepper & Silva 4719*). Vietnam. heng khôm (*Spire 1081*), cà co [Annamite] (*Pierre 706*), la plonh [Moi] (*Poilane 85*), blon blok (*Schmid 1608*).

#### Preliminary conservation status

**(IUCN 2019).** Least Concern (LC). EOO (10,914,652 km^2^, LC); AOO (1,052 km^2^, VU). *Solanuminsanum* is a widespread weedy species of disturbed areas and agricultural margins. It was not considered of conservation concern in an assessment of eggplant wild relatives (Syfert et al. 2016).

#### Discussion.

*Solanuminsanum* is the wild progenitor of the cultivated eggplant (aubergine; *S.melongena*) and readily crosses with that species (Davidar et al. 2015; Mutegi et al. 2015), thus complicating its recognition in the past (see references and discussion in Knapp et al. 2013; Ranil et al. 2016). We have chosen to recognise the two as distinct species rather than as infraspecific taxa (e.g., Lester and Hasan 1991; Daunay and Hazra 2012) because cultivated and wild plants are on distinct evolutionary trajectories. Wild plants are subject to natural selection, while cultivated plants are commensal with human beings. We recognise that this can lead to difficulties in identifying individual specimens, particularly those from old collections where details of provenance are not clear.

Distinguishing *S.insanum* from the cultivated *S.melongena* can be difficult in the absence of fruits. Keys in previous publications (Knapp et al. 2013; Ranil et al. 2016) can be used for identification, but in general specimens we assign to *S.insanum* are pricklier, with smaller flowers and fruit, and more ruderal than those we assign to *S.melongena*. Local cultivars of *S.melongena* in tropical Asia can be especially difficult to distinguish from *S.insanum*.

*Solanumincanum* L. is another species with which *S.insanum* is frequently confused, and the name *S.incanum* has frequently been used in the past literature for all “wild eggplants” (see Knapp et al. 2013 for the history of nomenclature of the wild eggplant relatives both in Asia and Africa). *Solanumincanum* occurs from across northern Africa and the Middle East to Pakistan; we have seen no specimens identifiable as *S.incanum* from the area treated here. The many specimens labelled “Punjab” from early collections in “British India” have all proved to be from Pakistan, not from adjacent India, but *S.incanum* potentially occurs near border areas. *Solanumincanum* is a more densely pubescent plant than is *S.insanum*, with long-stalked trichomes that dry a distinctive yellow colour (see Vorontsova and Knapp 2016 for a description) and plants are generally pricklier, although this character is extremely variable within these taxa; it generally grows in much drier habitats than does *S.insanum*. Crossability of these two taxa is not known, confusion over the identity of accessions used in crossing studies (e.g., Daunay and Hazra 2012) means that this remains to be tested.

Mill (2001: 1050) suggested *S.mirikense*, treated as a synonym of *S.insanum* here, was synonymous with *S.mauritianum* Scop., an introduced member of the Brevantherum clade. He had not seen the type specimen in CAL, which clearly places it as a synonym of *S.insanum*.

The sole element cited in Poiret’s (1797) description of *S.trongum* is the treatment and illustration of “Trongum agreste spinosum” in Rumphius (1747), and Merrill (1917) effectively lectotypified the name with this illustration by stating “type”. The illustration is of a very spiny plant, which Rumphius (1747) stated was wild and described as “ingrata et spinosa herba” (ungrateful and spiny herb).

The name *S.gula* has been placed in the synonymy of various spiny solanums over the years (R.N. Lester, m.s.). In the protologue Buchanan-Hamilton (1824) states “I found it first in my journey to Mysore, where it is called Gula, and in 1806 I gave specimens, a drawing and a description of it to Sir J.E. Smith”. No specimens are extant in the Smith herbarium at the Linnean Society of London, but the drawing held there (see https://linnean-online.org/168572/) is clearly of *S.insanum* and has been annotated as such in James Edward Smith’s hand. A specimen at BM (BM000900233) is labelled “Solanumgula B” by Buchanan-Hamilton and also has an annotation of “insanum L” in Smith’s hand. This is certainly the specimen mentioned in the protologue and is here designated as the lectotype of *S.gula*.

In the protologue of S.indicumvar.pubescensDunal (1852) cited two Wallich catalogue numbers, “2629 a, ex parte” and “2626 g”, and stated that he had seen specimens in G-DC and “herb. Wall.”. The catalogue number 2629 is a mixture of various species (including *S.pubescens*), so we have chosen the G-DC sheet of Wallich cat. 2626g (G00130396) as the lectotype because it is unambiguous, it matches the protologue and it was clearly seen by Dunal. The specimen labelled “Wallich cat. 2626G” at Kew in the Wallich Herbarium (K00116650) is a mixture of two taxa; the left-hand stem corresponds to *S.multiflorum* and the two right hand stems to *S.insanum*, only these two right-handstems are considered isolectotype material here.

Two of the four infraspecific taxa described by Dunal (1852) under *S.trongum* were described with no specimens cited, the only references are to previously published works and the descriptions are copied from those. Var. sinuato-pinnatifidum cites only “Blum. bijdr. 700, *karondong*, nomen Indicum”; this is a reference to Blume’s (1826: 702, an error in citation of page) *S.trongum* Poir., where the name karondong is stated, but no material or localities cited. We consider this homotypic with *S.trongum* and do not recognise Blume’s treatment of *S.trongum* as a distinct species. We consider var. rumphii to be illegitimate because Dunal (1852) cited the same type as that of *S.trongum*; Dunal appears to be using this as the “typical” variety. Both of the other varieties had specimens cited. Two Wallich catalogue numbers (burm. 135, 2628E) were cited in the protologue of S.trongumvar.tongdongense, both of which were seen in G-DC (“v.s. h. DC”). We have selected one of the duplicates of the best preserved of these, Wallich cat. iter. Burm. *135*, at G-DC as the lectotype. The protologue of var. divaricatum cites two collections (*Perottet s.n.* from “Pondicherry” [India], *Reynaud s.n.* from “Ceylon”) both seen in Paris. Both collections are *S.insanum*; we have selected the Reynaud specimen from Sri Lanka (P00049887) as the lectotype because it has both flowers and fruits.

Two collections from Java, *Hoffmansegg 119* in G-DC and *Roemer s.n.* in G (”herb. Boiss.”) were cited in the protologue of S.cyanocarphiumvar.obtusangulum; we have selected the first of these as the lectotype of the name because the number makes it unambiguous. Although Java collections are attributed to him (as here), Hoffmansegg never visited Java (van Steenis-Kruseman 1985); plants in his herbarium were collected for him there by a friend.

Of the many duplicates of *Cuming 443*, the sole collection cited in the protologue of *S.cumingi*, we have selected the one in G cited by Dunal (1852, as ”herb. Boiss.”) that bears an annotation label in hand as the lectoytpe (G00076245).

The protologue of *S.immane* cites no specimens or herbaria, but the name (and probably the description) is taken from Henry Fletcher Hance’s unpublished manuscript describing Chinese plants (Walpers 1852). Walpers worked in Berlin, and any specimens he saw are likely to have been destroyed. Of the specimens we have seen that could correspond to original material or duplicates of the Hance collection used to describe *S.immane*, only one specimen at BM (*Herb. Hance 860*, BM000942450) matches both the protologue description and the collection locality; we have selected this as the lectotype for the name. Other duplicates of *Hance 860* in P are either from different localities (i.e., P00049897, from “prope Cantonem”) or represent different species (e.g., P00055662 = *S.violaceum*).

#### Specimens examined.

See Suppl. materials 1–3.

### ﻿20. Solanum involucratum

Taxon classificationPlantaeSolanalesSolanaceae

Blume, Bijdr. Fl. Ned. Ind. 13: 701. 1826.

0601CCD9-B842-573E-BDB0-910152170F7F

**Figure 35.**
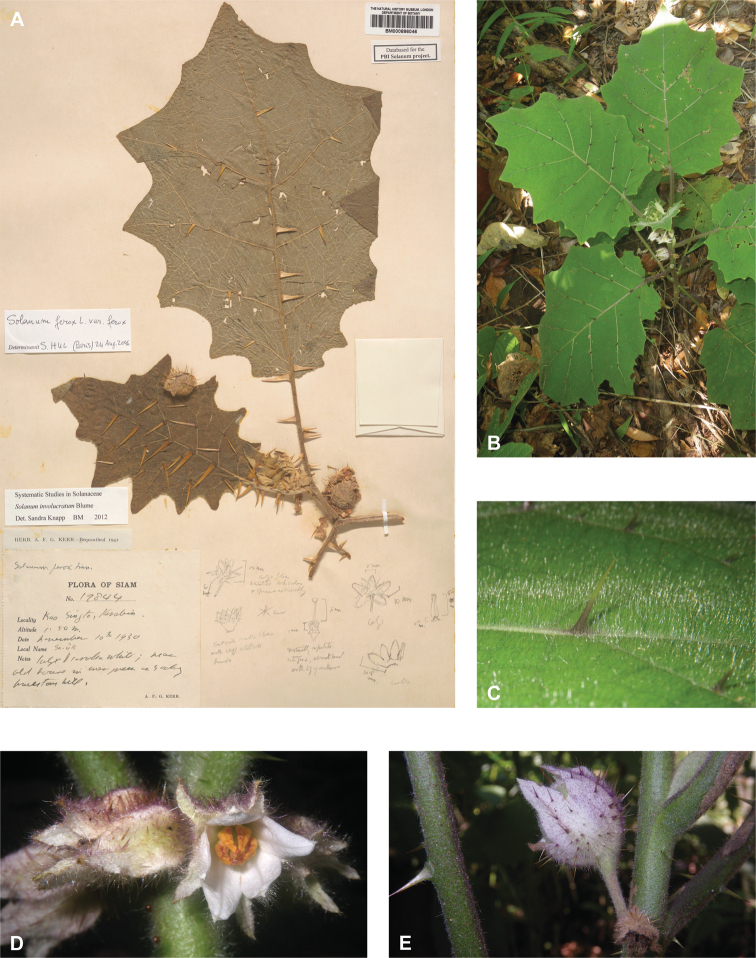
*Solanuminvolucratum* Blume **A** herbarium specimen collected in Thailand in 1930 (*Kerr 19844*, BM000886046) **B** Habit and leaves (field photograph, unvouchered, Vietnam) **C** detail of the trichomes and prickles on the adaxial surface of a blade (field photograph, unvouchered, Vietnam) **D** detail view of an inflorescence and a flower (field photograph, unvouchered, Vietnam) **E** detail view of an accrescent fruiting calyx (field photograph, unvouchered, Vietnam). Photograph credits: **A** CC-BY, © copyright The Trustees of the Natural History Museum, London **B–E** M. Nuraliev.

Figs 2G , 3C , 4C , 35

Solanum ferox L. var. involucratum (Blume) Miq., Fl. Ned. Ind. 2: 647. 1857. Type. Based on Solanuminvolucratum Blume.

#### Type.

Indonesia. Java: Sin. loc., *C.L. Blume s.n.* (lectotype, designated here: L [L0003633]).

#### Description.

Shrub to 2 m tall, strongly armed. Stems erect, terete, densely prickly and pubescent; prickles to 8 mm long, to 3 mm at the base, straight, awl-shaped to deltate, pale yellow, glabrous; trichomes porrect-stellate, mixture of subsessile and stalked, the stalks to 0.75 mm long, the rays 4–6, 0.4–1.5 mm long, the midpoints shorter than the rays or up to 2.25 mm long, sometimes purple-tinged on living plants; new growth densely pubescent with mixture of subsessile and long-stalked stellate trichomes; bark of older stems brownish, glabrescent. Sympodial units difoliate, the leaves not geminate. Leaves simple, shallowly to deeply lobed, the blades 12–30 cm long, 9.5–19 cm wide, ca. 1–1.5 times longer than wide, broadly elliptic to ovate, chartaceous, discolorous, armed with ca. 20–80 prickles per leaf side, prickles to 20 mm long, to 6 mm wide at the base, straight, awl-shaped, conical, pale yellow, on dried material, sometimes purple-tinged on living plants, glabrous; adaxial surface moderately pubescent, with sessile porrect-stellate trichomes, the rays 3–6, 0.1–0.25 mm long, the midpoints to 1.75 mm; abaxial surface densely stellate-pubescent with trichomes like those of the adaxial surface but stalked, the stalks to 0.4 mm; major veins 5–6 pairs drying light green; base attenuate; margins shallowly to deeply lobed, the lobes 5–6 on each side, 1–1.2 cm long, 3–5 cm wide, broadly deltate, apically acute, the sinuses less than halfway to the midrib; apex acute; petiole 3.5–17.5 cm long, 1/3–4/5 of the leaf blade length, prickly with 5–17 prickles like those of the blade, densely stellate-pubescent with porrect trichomes like those of the stem. Inflorescences apparently lateral, 3–8 cm long, unbranched, with 3–10 flowers, 1 to many flowers open at any one time, densely stellate pubescent with stellate-porrect trichomes like those of the stems but often tinted with purple, unarmed; peduncle 1–4 mm long, unarmed; pedicels 3–10 mm long, 1–2 mm in diameter at the base, ca. 1.5 mm in diameter at the apex, spreading, unarmed or sparsely prickly with a few prickles, densely stellate-pubescent with purple porrect-trichomes like the inflorescence axes, articulated at the base; pedicel scars spaced 0.5–1 mm apart. Buds ovoid, included in the calyx lobes until just before anthesis. Flowers 5-merous, apparently all perfect. Calyx with the tube 3.5–4 mm long, cup-shaped and slightly inflated at the base and appearing saccate, the lobes 4–6 mm long, 4–4.5 mm wide, deltate to broadly deltate, apically acute, armed with numerous prickles and densely stellate pubescent with purple-tinted porrect-trichomes like those of the pedicels. Corolla 1–1.2 cm in diameter, white, stellate, lobed ca. 1/2–2/3 of the way to the base, interpetalar tissue present, the lobes 4–6 mm long, 2–3 mm wide, deltate, spreading at anthesis, glabrous adaxially, densely stellate-pubescent abaxially on parts exposed in bud with trichomes like those of the calyx but white. Stamens equal; anthers 5–6 mm long, 1.5–2 mm wide, connivent, tapering, glabrous, poricidal at the tips, the pores directed distally, not elongating to slits with drying; filament tube ca. 0.5 mm long, glabrous; free portion of the filaments ca. 0.25 mm long, glabrous. Ovary conical, densely covered with long simple hairs; style ca. 6 mm long, slender, curved at the apex, glabrous; stigma capitate, the surfaces minutely papillose. Fruit a globose berry, 3–10 per infructescence, 1–2 cm in diameter, orange when mature, the pericarp thick, very densely stellate-pubescent with porrect-trichomes like those of the adaxial surface of the leaves; fruiting pedicels 0.8–2 cm long, ca. 1 mm in diameter at the base, 2.5–2.8 mm in diameter at the apex, woody, erect to spreading, armed with 3–25 prickles; fruiting calyx lobes expanding to 2 cm long, completely enclosing the fruit at maturity, armed with ca. 10–20 prickles per lobe, the lobes white with purplish venation and trichomes. Seeds >100 per berry, 2–3 mm long, 1.5–2 mm wide, flattened-reniform, dull yellow, the surface minutely pitted, the testal cells sinuate in outline. Chromosome number: not known.

**Figure 36.**
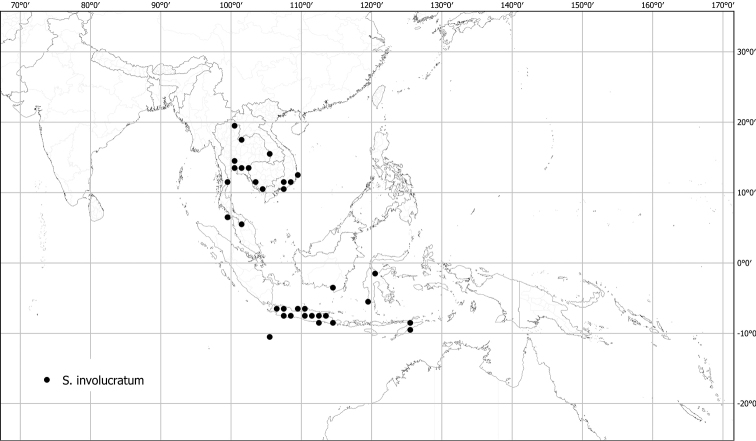
Distribution of *S.involucratum*.

#### Distribution

**(Fig. 36).***Solanuminvolucratum* is widely distributed in Indochina and Malay Archipelago (Indonesia, Malaysia, and Christmas Island).

#### Ecology and habitat.

*Solanuminvolucratum* is a plant of tropical evergreen or deciduous rainforest, it grows on limestone or on clay soil, also in secondary forests; from 50 to 1,400 m elevation.

#### Common names and uses.

Laos. hma:k sè:n, khüa khünx, hma:k khüa hlè:z, khüa hna:m (Hul and Dy Phon 2014). Thailand. Prachinburi: sa-uk [Thai] (*Kerr 19844*); Prachuap Khiri Khan: ma uk muak [Thai] (*Kerr 10780*); Vietnam. ca duoc nui [Vietnamese] (*Fleury 29910*); cà du’o’c nui, cây cà rai, cà ung, co chu [Munong] (Hul and Dy Phon 2014).

#### Preliminary conservation status

**(IUCN 2019).** Least Concern (LC). EOO (1,868,808 km^2^, LC); AOO (216 km^2^, EN). *Solanuminvolucratum* is found over a very broad range in the archipelago, the AOO measurement certainly reflects collecting bias.

#### Discussion.

*Solanuminvolucratum* is a distinctive species, with pubescent berries entirely enclosed within a spiny accrescent calyx. In flower the corolla lobes are shorter than the calyx, so the flowers appear to be entirely within the calyx at anthesis. In flower the calyx tube appears to be somewhat inflated, but it is appressed against the berry in fruit. The bristly, prickly accrescent calyx in fruit is similar to that of *S.barbisetum*, *S.cyanocarphium* and *S.praetermissum*, but all of those species have glabrous fruits, while those of *S.involucratum* are densely pubescent with stiff, stellate trichomes. The inflorescences of *S.cyanocarphium* are few-flowered like those of *S.involucratum*, but not robust. Inflorescences of *S.barbisetum* and *S.praetermissum* are elongate and multi-flowered.

On the island of Sulawesi *S.involucratum* is sympatric with the very similar *S.sulawesi.* It differs from that species in its accrescent calyx in fruit, larger mature berries and more densely pubescent stems and leaves. Both *S.involucratum* and *S.lasiocarpum* have leaves with petioles that are longer relative to leaf size than *S.sulawesi*, but these three species can be difficult to distinguish.

*Solanuminvolucratum* was considered by Miquel (1857) to be a variety of *S.lasiocarpum* (as S.feroxvar.involucratum). The two species are not closely related; *S.involucratum* is a member of a clade comprising *S.procumbens*, *S.expedunculatum* Symon and *S.leptacanthum* Merr. & L.M.Perry (the latter two species from New Guinea), itself apparently related, but with poor support, to *S.barbisetum* and *S.praetermissum* (Aubriot et al. 2016a). *Solanumlasiocarpum* on the other hand has been shown in numerous studies to be a member of the otherwise American Lasiocarpa clade (e.g., Bruneau et al. 1995; Bohs 2004; Stern et al. 2011).

Heiser (1996, 2001) followed Miquel (1857) in using the name *S.ferox* L., nom utique rej. (see Knapp 2011) for *S.involucratum*, recognising it at the varietal level, while Whalen et al. (1981) regarded it as ambiguous. Care should be taken with specimens annotated as *S.ferox* in collections, they could be either *S.lasiocarpum* (more common in our experience, see discussion under that species) or *S.involucratum*.

Hul and Dy Phon (2014) incorrectly cited a specimen at L as “holo – L!” for *S.involucratum*. This is not a holotype (see McNeill 2014) and we have selected as lectotype a specimen at L (L0003633) that matches the protologue and appears to have been original material; this is probably the same sheet referred to as holotype by Hul and Dy Phon (2014).

#### Specimens examined.

See Suppl. materials 1–3.

### ﻿21. Solanum jamaicense

Taxon classificationPlantaeSolanalesSolanaceae

Mill., Gard. Dict., ed. 8 Solanum 17. 1768.

914C7256-CD79-503B-B52F-BF738FEEA795

Fig. 22E, F No names based on tropical Asian types have been published; for complete synonymy see D’Arcy 1974.

#### Type.

Jamaica: “Solanum bacciferum caule et foliis tomentis incanis spiosis, flor luteo, fructu croceo, minore Sl. Cat: 107” [as Herb. Sloane 107 in D’Arcy 1974a, a correctable error], *W. Houstoun s.n.* (lectotype, designated by D’Arcy 1974a, pg. 865, as “type”: BM [BM000815972]).

#### Description.

D’Arcy (1974a: 685–686); Nee (1993: 77–78); http://www.solanaceaesource.org/solanaceae/solanum-jamaicense.

#### Distribution.

*Solanumjamaicense* has been recorded from Indonesia (islands of Borneo, Java, Sulawesi, and Sumatra) and the Philippines in tropical Asia, the single collection we have seen from China was cultivated; it is native to the Caribbean, Central and South America, where it occurs in forests and forest margins. It is also known from the state of Florida (United States of America).

#### Discussion.

*Solanumjamaicense* is apparently only sporadically adventive in rainforest areas; most collections are from around logging camps. It is not easily confused with any other from the region. Its sharply hooked prickles, attenuate, decurrent leaves, and tiny flowers and fruits are distinctive.

*Solanumjamaicense* has been identified as a potential problematic weed in the state of Florida (Diaz et al. 2008) where it is locally invasive in the hammock habitat in the central part of the state where it was first recorded in 1930. It has not yet, however, become more widely distributed there.

D’Arcy (1974a) inadvertently lectotypified *S.jamaicense* by citing the specimen collected by William Houstoun at BM (BM000815972) labelled with the polynomial used in the protologue that came from Philip Miller’s herbarium. Although Miller (1768) did not cite Houstoun explicitly in the protologue of *S.jamaicense*, he had access to Houstoun’s collections before 1768, so this specimen qualifies as original material. He did cite Houstoun in association with this polynomial in earlier editions of the “Gardener’s dictionary” (J. Wajer, pers. comm.). An annotation at the base of the twig in Daniel Solander’s hand “Solanum Jamiacense Mill dict” indicates the sheet was part of Miller’s herbarium used for the dictionary and had been filed as *S.jamaicense* prior to the herbarium coming to BM. We consider the use of “Herb. Sloane” rather than “Sl[oane]. Cat[alogue]:” by D’Arcy (1974a), and subsequently followed by others (Nee 1993), to be a correctable error; therefore, a second step lectotypification is not needed.

#### Specimens examined.

See Suppl. materials 1–3.

### ﻿22. Solanum kachinense

Taxon classificationPlantaeSolanalesSolanaceae

X.Aubriot & S.Knapp sp. nov.

E09232C9-1283-55FD-873C-59488CC57213

urn:lsid:ipni.org:names:77298803-1

Fig. 37

#### Diagnosis.

Like *Solanumtorvum* Sw., but differing in the eglandular pubescence of the inflorescence, more highly branched inflorescences, smaller flowers and almost glabrous mature foliage.

#### Type.

Myanmar. Kachin: Putao District, “North Triangle (Hkinlum)”, 29 Jul 1953, *F. Kingdon-Ward 21211* (holotype: BM [BM000900353]; isotype: A).

#### Description.

Shrubs of unknown height, armed. Stems erect, terete, drying black, prickly and sparsely stellate-pubescent; prickles broad-based, to 1 cm long, 0.8 cm wide at the base, straight, straw-colored; pubescence of mixed sessile and very short-stalked porrect-stellate trichomes, the stalks to 0.1 mm, the rays 6–8, ca. 0.5 mm long, the midpoints to 0.2 mm long, always shorter than the rays; new growth densely stellate-pubescent, mixed sessile and short-stalked porrect-stellate like those of the stems, the trichomes golden, soon deciduous and the stems glabrate; bark of older stems brown or dark brown. Sympodial units difoliate, the leaves geminate, the leaves of a pair only differing in size. Leaves simple, shallowly lobed, the blades 16–22 cm long, 8–11.5 cm wide, ca. 1.5 times longer than wide, elliptic, widest at the middle or just below, chartaceous, discolorous, armed or unarmed; adaxial surface glabrous to sparsely stellate-pubescent along the veins with a few scattered sessile porrect-stellate trichomes, the rays 5–6, ca. 0.5 mm long, the midpoints equal to the rays; abaxial surface similarly but more densely pubescent with mixed sessile and short-stalked porrect-stellate trichomes, the stalks if present less than 0.2 mm long, the trichomes still only along the veins; major veins 6–8 pairs, drying black, very sparsely stellate-pubescent especially abaxially, sometimes armed, the prickles if present 1–3 per face, to 1.5 cm long; base truncate, strongly oblique with the basiscopic side 1–2 cm further along the petiole; margins shallowly lobed, the lobes broadly deltate with acute tips, the sinuses less than halfway to the midrib; apex acute; petioles 1.8–3 cm long, ca. 1/10 as long as the leaf blades, unarmed and glabrous to stellate-pubescent with a few golden porrect-stellate trichomes like those of the stems, drying blackish brown. Inflorescences 3–6 cm long, internodal and lateral, forked to 5-times branched, with 50–60 flowers, many flowers open at any one time, glabrous but sparsely pubescent with porrect-stellate trichomes at the distal ends; peduncle 1.5–4 cm long, glabrate; pedicels 0.5–0.7 cm long, ca. 1 mm in diameter at the base, ca. 1 mm in diameter at the apex, nodding at anthesis, sparsely stellate-pubescent with golden porrect-stellate trichomes like the inflorescence axes, articulated at the base; pedicel closely spaced ca. 1 mm apart or slightly overlapping. Buds elongate and tapering, strongly exserted from the calyx before anthesis. Flowers 5-merous, apparently all perfect. Calyx with the tube 1.5–2 mm long, conical, scarious and irregularly ripping into lobes at anthesis, the lobes 2–3.5 mm long (including the tip), ca. 2 mm wide, deltate with a subulate tip 1.5–2 mm long, sparsely stellate-pubescent like the pedicels, the subulate tip glabrous. Corolla 1.5–1.8 cm in diameter, “purple sometimes white” (fide *Kingdon Ward 21211*), stellate, lobed nearly to the base or 3/4 of the way to the base, with little interpetalar tissue, the lobes 6.5–8 mm long, 2.5–4 mm wide, spreading or perhaps somewhat reflexed, glabrous adaxially or with a few stellate trichomes along the petal midvein, densely stellate-pubescent abaxially, the trichomes porrect-stellate with 4–8 weak, tangled rays. Stamens equal; anthers 5–5.5 mm long, ca. 1 mm wide, tapering, yellow, poricidal at the tips, the pores directed distally, not elongating to slits with drying; filament tube minute, glabrous; free portion of the filaments ca. 1 mm long, glabrous. Ovary conical, glabrous; style 6–8 mm long, glabrous; stigma clavate, the surfaces minutely papillose. Fruit and seeds not known. Chromosome number: not known.

**Figure 37.**
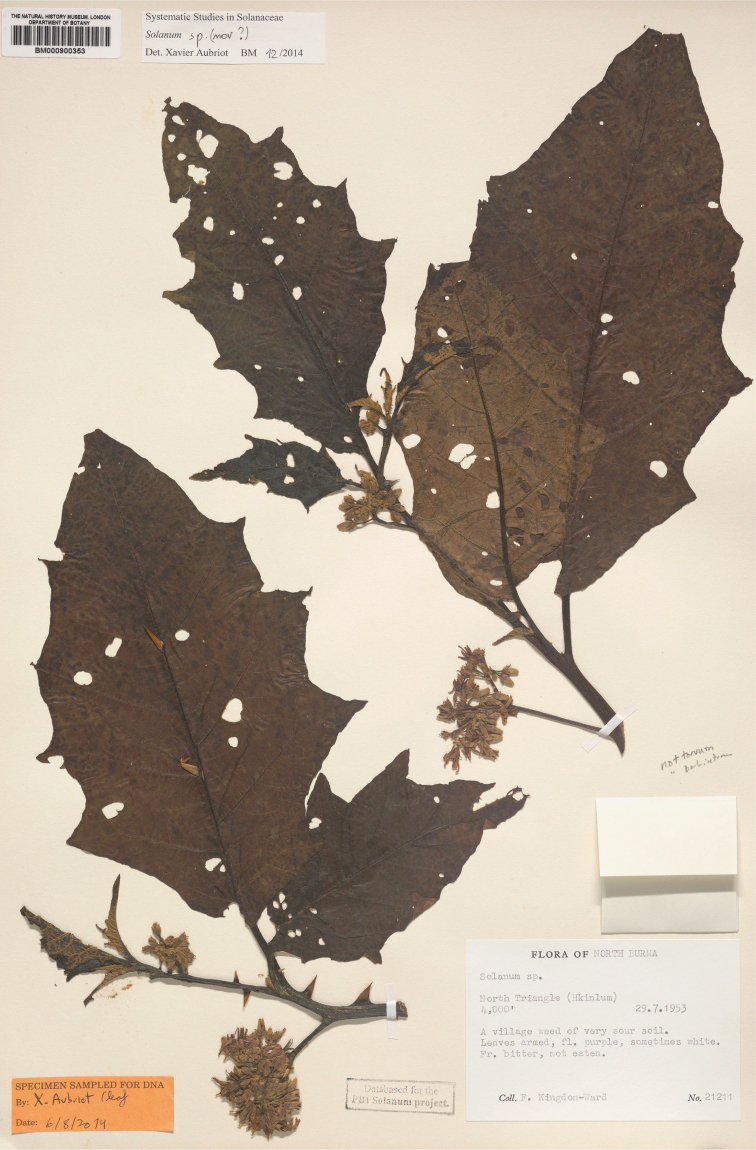
*Solanumkachinense* X.Aubriot & S.Knapp – Herbarium specimen (holotype) collected in Myanmar in 1953 (*Kingdon-Ward 21211*, BM000900353). Photograph credit: CC-BY, © copyright The Trustees of the Natural History Museum, London.

#### Etymology.

*Solanumkachinense* is named in honour of the province of Myanmar where it occurs, and for the Kachin peoples of the region – who generously assisted the Kingdon-Wards while they were collecting in the region.

#### Distribution

**Figure 38.**
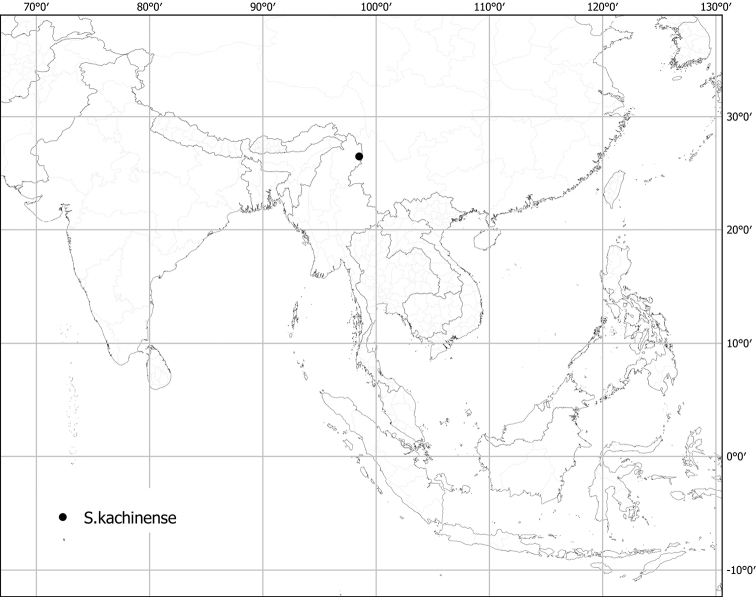
Distribution of *S.kachinense*.

**(Fig. 38).***Solanumkachinense* is known only from the type collection, made in northern Myanmar in the drainage of the Mali Kha, one of the eastern tributaries of the Irrawaddy River whose waters are fed by the Himalayan glaciers of Tibet.

#### Ecology and habitat.

The type specimen indicates *S.kachinense* is a “village weed of very sour soil” indicating it grows, as do many solanums, in disturbed or open areas; the village of Hkinlum is at approximately 1,200 m elevation in a region where tropical and temperate elements of the flora mix; Kingdon-Ward (1956) characterised the vegetation of Hkinlum as “moist warm temperate evergreen forest. Not subtropical…”

#### Common names and uses.

Myanmar. “fruits bitter, not eaten” (*Kingdon-Ward 21211*).

#### Preliminary conservation status

**(IUCN 2019).** Data Deficient (DD). Known only from a single collection, data on the distribution and status of *S.kachinense* is insufficiently known to speculate about its conservation status.

#### Discussion.

Despite being currently known to us from a single collection, we describe *S.kachinense* here because it is so distinct, and that future botanists will be encouraged to find more collections because the entity is known to exist. Very often botanists pass by spiny solanums as uninteresting, in part due to the ubiquity of many introduced species such as *S.torvum*.

Morphologically, *S.kachinense* is similar to members of the Torva clade (sensu Stern et al. 2011; Aubriot et al. 2016a) with difoliate sympodia and lateral or leaf-opposed inflorescences with many flowers and fruits. It differs from all other Torva clade species in the region (*S.chrysotrichum*, *S.comitis*, *S.poka*, *S.peikuoense*, *S.pseudosaponaceum*, *S.torvoideum* and *S.torvum*) in its glabrate leaves and stems with long (to 1 cm) very broad-based prickles, especially on stems It has larger flowers than the almost sympatric *S.pseudosaponaceum* (1–1.8 cm in diameter versus 1–1.5 mm). The glandular simple trichomes present on the inflorescence axes of *S.torvum* (see description in Vorontsova and Knapp 2016 and on Solanaceae Source) are absent in *S.kachinense*.

The glabrate upper leaf surfaces are similar to those of *S.giganteum* but the copious scurfy white pubescence of inflorescences, leaf undersides and stems on that species is distinctive; even in more glabrate individuals of *S.giganteum* the pubescence is of multangulate trichomes, not of porrect-stellate trichomes like those of *S.kachinense*.

*Solanumkachinense* was collected on Frank and Jean Kingdon-Ward’s last trip to the drainages of the upper Irrawaddy in Myanmar to collect in the high mountains bordering China. Hkinlum was their base for their forays into the higher mountains collecting rhododendrons, lilies and alpines for the horticultural trade in England. This plant was not mentioned in Kingdon-Ward (1956); it was collected in the hot, wet season between the Kingdon-Ward’s forays into the mountains, spring for flowers and autumn for seeds when they were perhaps a bit fed up with being stuck in a hot, wet village.

#### Specimens examined.

See Suppl. materials 1–3.

### ﻿23. Solanum lasiocarpum

Taxon classificationPlantaeSolanalesSolanaceae

Dunal, Hist. Nat. Solanum 222. 1813.

3295FE27-C2A2-5326-B977-030BFC4DCEBE

Figs 1C , 2A , 39

Solanum indicum L., Sp. Pl. 187. 1753. nom. utique rej. Type. Sri Lanka. Sin. loc., “Habitat in India utraque”, *Anonymous s.n.* (lectotype, designated by Hepper 1978, pg. 555: Herb. Hermann 3: 16, No. 94 [BM000594658]).
Solanum hirsutum Roxb. ex Wall., Fl. Ind. (Carey & Wallich ed.) 2: 253. 1824, nom. illeg., not Solanumhirsutum Dunal, 1813. Type. probably based on Solanumlasiocarpum Dunal.
Solanum quadriloculare Spreng., Syst. Veg., ed. 16 [Sprengel] 4(2, Cur. Post.): 72. 1827. Type. based on SolanumSolanumhirsutum Roxb. ex Wall.
Solanum zeylanicum Blanco, Fl. Filip. [F.M. Blanco] 136. 1837, nom. illeg., non Solanumzeylanicum Scop., 1786. Type. Philippines (no specimens or illustrations cited; no specimens extant). Philippines. Luzon, Benguet province, May 1914, *E.D. Merrill [Species Blancoanae] 465* (neotype, designated here: A [00230113]; isoneotypes: CAL [acc. # 316470], K [K000195921], P [P00369171]).
Solanum lasiocarpum Dunal var. velutinum Dunal, Prodr. [A. P. de Candolle] 13(1): 253. 1852. Type. Philippines. Sin. loc., 1841, *H. Cuming 690* (lectotype, designated here: P [P00379526]; isolectotypes: BM [BM000886159], K [K000195954, K000195955], G [G00301654], P [P00379525]).
Solanum ferox L. var. lasiocarpum (Dunal) Miq., Fl. Ned. Ind. 2: 647. 1857. Type. Based on Solanumlasiocarpum Dunal.
Solanum lasiocarpum Dunal var. domesticum Heiser, Solanaceae: Biol. & Syst. (ed. D’Arcy) 413. 1986. Type. Cultivated in the Indiana University greenhouses from seeds of “fruits purchased in market in Bangkok, Thailand, Sept. 1, 1980” [collection date mistakenly as “September 21, 1980” in the protologue], 30 Jun 1981, *C.B. Heiser 8008* (holotype: IND [IND-1000065]).

#### Type.

India. Sin. loc. (lectotype, designated by Whalen et al. 1981, pg. 100: [illustration] Rheede von tot Draakestein, Hort. Malab. 2: tab. 35, “*Ana-Schunda*”. 1680).

#### Description.

Shrub to 2 m tall, often heavily armed. Stems erect to spreading, terete, unarmed or sparsely to moderately prickly and stellate-pubescent; prickles to 0.5–1 cm long, broad-based, deltate to narrowly deltate, laterally compressed, very variable in size on a single stem; trichomes porrect-stellate, mixture of sessile and variously stalked, the stalks multiseriate, to 2.5 mm long, the rays 5–8, 0.4–1.5 mm long, the midpoints variable in length, shorter or longer than the rays; new growth densely pubescent; bark of older stems brownish, moderately to sparsely prickly and stellate-pubescent. Sympodial units difoliate, the leaves geminate, the leaves of a pair equal in size and shape or the minor leaf slightly smaller. Leaves simple, shallowly lobed, the blades 20–35 cm long, 15–30 cm wide, ca. 1–1.5 times longer than wide, broadly ovate to ovate, chartaceous, discolorous, armed with prickles similar to those of the stem, these scattered along the midrib and principal lateral veins, often dark-purplished tinged; adaxial surface felty, densely stellate pubescent with porrect sessile trichomes, the rays 2–5, 0.1–0.5 mm long, the midpoints to 2.5 mm long; abaxial surface felty, densely stellate pubescent and farinaceous in appearance, with porrect trichomes like those of the adaxial surface but with longer stalks, the stalks to 2 mm long; principal veins 5–7 pairs, often drying yellowish abaxially; base truncate or obtuse; margins shallowly to moderately lobed, the lobes 3–6 on each side, occasionally somewhat dentate, 1.6–4 cm long, 3–5 cm wide, deltate to broadly deltate, acute or rounded at the tips, the sinuses less than halfway to the midrib; apex acute; petioles 3–14 cm, 1/5–1/2 the length of the blades, densely stellate-pubescent, unarmed or with a few to many prickles like those of the stems, the pubescence with a mixture of stipitate and sessile porrect-trichomes like those of the stem. Inflorescences 0.4–0.9 cm, extra-axillary, often very close to a leaf pair, unbranched, with 6–16 flowers, only one or two open at a time, densely stellate-pubescent, frequently prickly, the prickles like those of the stems; peduncle 0.1–0.4 cm, unarmed or with a few prickles; pedicels 4–9 mm long, 1–1.5 mm in diameter at the base, 1.5–2 mm in diameter at the apex, spreading or somewhat reflexed at anthesis, densely stellate-pubescent like the inflorescence axes, unarmed or with a few prickles, articulated at the base; pedicel scars 0.25–1.25 mm apart. Buds ovoid, the corolla enclosed within the calyx lobes and tube until just before anthesis. Flowers 5-merous, heterostylous and the plants andromonoecious, with the lowermost flower long-styled and hermaphrodite, the distal flowers short-styled and staminate. Calyx with the tube 2.5–4.5 mm long, broadly campanulate, the lobes 3–5 mm long, 2.5–5 mm wide, deltate to broadly deltate, apically acute, unarmed and densely stellate-pubescent with porrect-stellate trichomes like those of the pedicels but the midpoints usually somewhat longer. Corolla 2.5–3.5 cm in diameter, white, stellate, lobed 1/2–3/4 of the way to the base, interpetalar tissue absent, the lobes 6–9 long, 3–6 mm wide, broadly ovate, spreading at anthesis, the tips somewhat reflexed, glabrous adaxially, densely stellate-pubescent abaxially with porrect-stellate trichomes where exposed in bud. Stamens equal or slightly unequal; filament tube minute; free portion of the filaments 0.1–0.2 mm long, glabrous; anthers 6–8.5 mm long, 1.5–2.2 mm wide, strongly tapering, connivant but the tips somewhat spreading, yellow, glabrous, poricidal at the tips, the pores directed distally, not elongating to slits with drying. Ovary conical, pubescent, the hairs appearing simple but with poorly developed and very short rays at the base; style of long-styled flowers 5–10 mm long, glabrous, 1–2 mm long in short-styled flowers; stigma capitate, the surface minutely papillose. Fruits a globose berry, 1–5 per inflorescence, 2.5–3.5 cm in diameter, orange when ripe, the pericarp thin, densely stellate pubescent with sessile porrect-trichomes, the rays 5–15, 0.1–0.4 mm long, the midpoints always longer than the rays, 1.5–4 mm long; fruiting pedicels 1–1.5 cm long, ca. 2.5 mm in diameter at the base, ca. 4 mm in diameter at the apex, woody, spreading or deflexed from weight of the berry; fruiting calyx not markedly accrescent, the lobes only slightly lengthening in fruit and often breaking off, usually unarmed. Seeds 100–200+, 2.2–3.5 mm long, 1.75–2.5 mm wide, flattened reniform, tan, the surfaces minutely pitted, the testal cells with sinuate margins. Chromosome number: n = 12 (Whalen et al. 1981; Madhavaian 196814, as *Solanumferox* L.).

**Figure 39.**
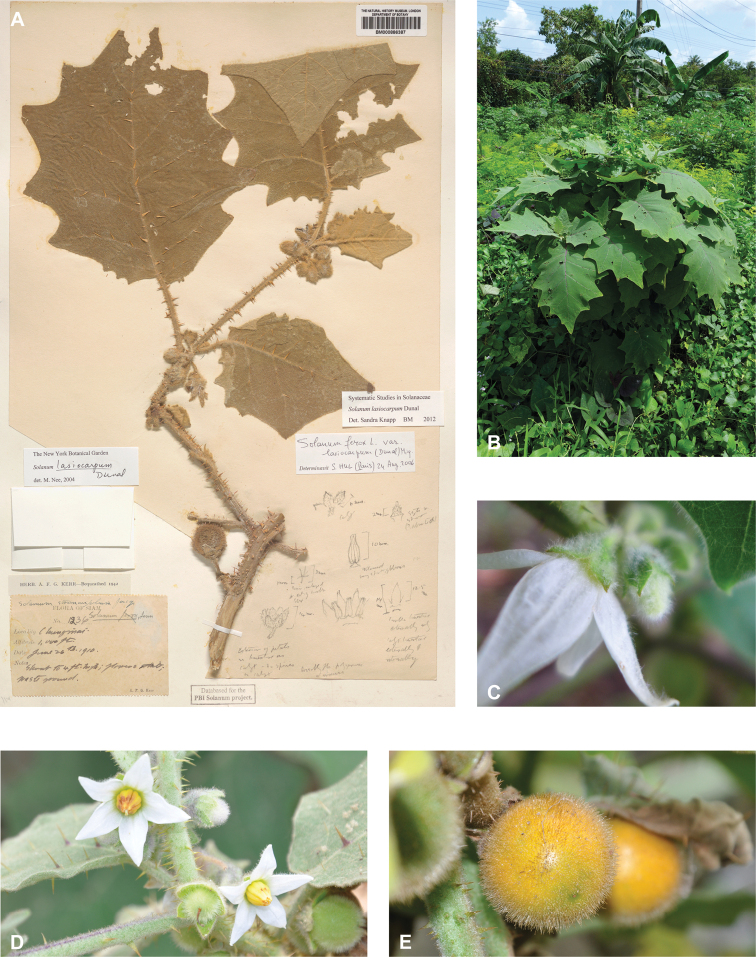
*Solanumlasiocarpum* Dunal **A** herbarium specimen collected in Thailand in 1910 (*Kerr 1236*, BM000886387) **B** habit (*Meeboonya et al. RM 287*, Thailand) **C** detail view of a calyx and a floral bud (*Meeboonya et al. RM 272*, Thailand) **D** detail view of an inflorescence with immature fruits (*Wang et al. 2063*, China) **E** detail view of a mature fruit (*Wang et al. 2063*, China). Photograph credits: **A** CC-BY, © copyright The Trustees of the Natural History Museum, London **B, C** X. Aubriot **D, E** S. Knapp.

#### Distribution

**Figure 40.**
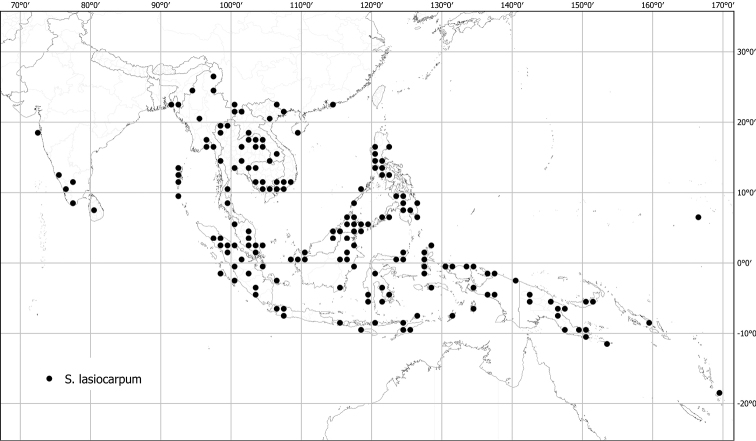
Distribution of *S.lasiocarpum.*

**(Fig. 40).***Solanumlasiocarpum* is a commonly cultivated plant and occurs from tropical India east through Indochina, extreme southern China, Malaysia and Indonesia to the Philippines and the island of New Guinea.

#### Ecology and habitat.

*Solanumlasiocarpum* grows in forest openings, disturbed sites and second growth thickets, from sea level to 1,000 m elevation. Widely cultivated in the region for its fruit.

#### Common names and uses.

Unless otherwise indicated, common names are from Whalen et al. (1981: 103); languages when recorded are in square brackets. Brunei Darussalam: tarang asai, tarung bawi (*Bernstein 133*); China: mo ke shue (*Tsang 16102*); India: ram-begun [Bengali]; Kerala vellathu-vazhuthana (Mohanan and Henry 1994, as. *S.ferox*). Indonesia. Maluku: tomate hutan (*Nooteboom 5837*), trong baguri (*Robinson 286*); Sumatra: lattoeeng (many collections), terong hutan (*Lörzing 15014*), sontoman (*si Toroes 2848*); Laos: chou pout din (*Vidal 5438*), gim ông (*Vidal 5438*); Malaysia (general): tar-ong-asam [Malay]; Sabah: tarong sulok [Kadayan] (*Abdul 2854*), terong pipit or terong pasai (*Cuadra A-2160*), tokung [Sungei] (*Cuadra A-1169*); Malaysia/Singapore: tĕrong asam (Burkill 1935); Philippines: basula [Ibang], dabatung [Sulu], tagtum [Panay-Bisaya], tarong-ayam [Bikol], tarong-tarong [Samar-Bisaya, Tagalog], talong gubat [Tagalog] (Blanco 1837: 136); Sri Lanka: mala-batu [Sinhalese]; Taiwan: da hugno teg io (*Henry 358*);Timor Leste: kaubasu (*Wiriadianata 428*); Thailand: ma ûk muak (Burkill 1935), ma uek (Heiser 1986), makhua-puu, ma-puu, mauek, yang-khui-dee, bakuek (*Widmer 106*); Vietnam: cây ca [Annamite] (*Poilane s.n.*), xo plo xoc [Moi] (*Poilane 184*). More orthographic and local variants of these names occur throughout the region (see Suppl. material 1 and dataset on the NHM Data Portal, https://doi.org/10.5519/0rqfzvgd).

*Solanumlasiocarpum* is widely cultivated across its range for its juicy fruits that are used as a base for juices and for cooking, especially in curries (Heiser 1986). The seeds are burned as used for relief of toothache and in Bangladesh *S.lasiocarpum* is used as a remedy for coughs, asthma, fever, vomiting, and gonorrhoea (Lim 2012).

#### Preliminary conservation status

**(IUCN 2019).** Least Concern (LC). EOO (6,950,615 km^2^, LC); AOO (1,032 km^2^, VU). As a cultivated plant, the true wild distribution of *S.lasiocarpum* is difficult to assess. As a weedy ruderal species, however, we suggest it is not of immediate conservation concern.

#### Discussion.

*Solanumlasiocarpum*, together with *S.repandum* G.Forst. of the Pacific islands (see Whalen et al. 1981), is one of two non-American members of the otherwise Neotropical Lasiocarpa clade (Stern et al. 2011) and shares with those species large repand leaves and pubescent berries. Heiser (1987) postulated that *S.lasiocarpum* was an introduction of the American species *S.candidum* Lindl. by Spanish mariners. Whalen et al. (1981) suggested that *S.lasiocarpum* was closely related to and were not “at ease retaining them” as distinct, but did pending further study; they suggested that the distribution of *S.lasiocarpum* was a puzzling historical problem and was likely not due to recent human introduction. The taxa are indeed morphologically very similar with felty, repand leaves and pubescent fruits, but recent molecular analyses have shown that *S.lasiocarpum* is in fact more closely related to the Pacific species *S.repandum* G.Forst. (Bruneau et al. 1995; Bohs 2004; Aubriot et al. 2016a) and that the morphological similarities are more likely due to convergence.

Cultivated forms of *S.lasiocarpum* in general lack or have very few prickles, but this character is highly variable. The fruits of *S.lasiocarpum* are bright orange and have juicy sweet-sour flesh, they are not as large as the fruits of the more commonly cultivated South American species of the Lasiocarpa clade *S.quitoense* Lam. and *S.sessiflorum* Dunal, nor of those of the Pacific *S.repandum* (see Whalen et al. 1981). *Solanumlasiocarpum* is not as strongly andromonoecious as are species such as *S.insanum* or *S.melongena*, and often several of the lowermost flowers are hermaphroditic and develop berries. In other species of the Lasiocarpa clade the expression of andromonoecy is quite labile (Miller and Diggle 2003; Diggle and Miller 2004), we suspect this is the case for *S.lasiocarpum* as well.

In many herbaria specimens of *S.lasiocarpum* and of *S.involucratum* are annotated as *Solanumferox* L., nom utique rej. This confusion is the result of the typification of another suppressed name, *S.indicum* L. (discussed in detail in Knapp 2011). Miquel (1857) recognised *S.involucratum* and *S.lasiocarpum* as varieties of the same taxon (as *S.ferox*). Heiser (1996, 2001) used the name *S.ferox* for *S.involucratum*, while Whalen et al. (1981) regarded it as ambiguous. Thus, care should be taken with specimens annotated as *S.ferox* in collections, they could be either *S.lasiocarpum* (more common in our experience) or *S.involucratum*. The two species are not closely related; *S.involucratum* is a member of a clade comprised of *S.procumbens*, *S.expedunculatum* Symon and *S.leptacanthum* Merr. & L.M.Perry (the latter two species from New Guinea), itself apparently related to *S.barbisetum* and *S.praetermissum* (Aubriot et al. 2016a). *Solanumlasiocarpum* on the other hand has been shown in numerous studies to be a member of the otherwise American Lasiocarpa clade (e.g., Bruneau et al. 1995; Bohs 2004; Stern et al. 2011).

Whalen et al. (1981) did not treat several of the taxa we recognise as synonyms of *S.lasiocarpum* here, necessitating typification of these names. No herbarium or specimens were cited in Blanco’s (1837) “Flora de Filipinas”, and no herbarium is known to exist (Merrill 1918a, 1923). Merrill (1918a) provided a series of “illustrative specimens” in order to help fix applications of the Blanco’s names, mostly from his series “Merrill: Species Blancoanae”. He felt that just because a work was difficult, it should not be ignored, stating “We can no longer look on the work or this or that author, no matter how incomplete or imperfect, as unworthy of consideration, nor can we accept Hooker’s dictum, regarding species proposed by such authors as Blanco, that it was undesirable to devote time to their identification” (Merrill 1918a: 6). We have selected the duplicate of Merrill’s “illustrative specimen” of *S.zeylanicum*, *Merrill Species Blancoanae 465* at the Arnold Arboretum (A barcode 00230113) as the neotype for this name.

In describing S.lasiocarpumvar.velutinumDunal (1852) cited two collections, one from Macau (*Callery s.n.* in P) and the other (*Cuming 690*) from the Philippines. We have selected the best-preserved duplicate of the most widely distributed of these (*Cuming 690*, P00379526) as the lectotype.

#### Specimens examined.

See Suppl. materials 1–3.

### ﻿24. Solanum macrocarpon

Taxon classificationPlantaeSolanalesSolanaceae

L., Mant. Pl. Altera 205. 1771.

51571210-C869-5987-9522-1041BD7A3A72

Fig. 22G, H Names based on tropical Asian types only; for complete synonymy see Vorontsova and Knapp 2016.

Solanum dimorphum Matsum., Bot. Mag. (Tokyo) 15: 56. 1901. Type. Cultivated in Tokyo, Japan “cult. Hort. Bot. Tokyo” (possibly from material collected in Taiwan but origin uncertain) (no specimens cited; no original material found).

#### Type.

Cultivated in Uppsala, Sweden, *Hort. Uppsala s.n.* (lectotype, designated by Hepper and Jaeger 1985, pg. 391: LINN [acc. # 248.11]).

#### Distribution.

*Solanummacrocarpon* is widely distributed across Africa and has been introduced in cultivation in other parts of the world possibly in association with the trans-Atlantic slave trade (e.g., Brazil, the Caribbean, Central America). In tropical Asia *S.macrocarpon* has been collected in Sri Lanka, Singapore, and China, but may be more widely cultivated (see below). *Solanummacrocarpon* is not known outside cultivation.

#### Common names.

Gboma eggplant is the English common name in widest use for this species; for common names in Africa see Vorontsova and Knapp (2016).

#### Description.

Vorontsova and Knapp (2016: 225–229); http://www.solanaceaesource.org/solanaceae/solanum-macrocarpon.

#### Discussion.

*Solanummacrocarpon* is a cultivated plant, used for its leaves and fruits in Africa (Vorontsova and Knapp 2016); its wild progenitor is *S.dasyphyllum* Schumach. & Thonn. of sub-Saharan Africa. It is easily distinguished from other cultivated eggplants in tropical Asia by its large, glabrous or very sparsely pubescent leaves with attenuate bases decurrent onto the stem, its large flowers with copious interpetalar tissue, and its foliaceous calyx lobes.

We have seen only a few specimens of *S.macrocarpon* L, although Burkill (1935) reports its use in the Malay Peninsula as “spreading throughout the Dutch Indies” and used as a pot-herb (leaves eaten). Accessions of *S.macrocarpon* are held in the collections of the World Vegetable Center (WorldVeg or AVDRC) in the Philippines (Taher et al. 2017) but it is not widely cultivated nor has it escaped or naturalised.

#### Specimens examined.

See Suppl. materials 1–3.

### ﻿25. Solanum mammosum

Taxon classificationPlantaeSolanalesSolanaceae

L., Sp. Pl. 1: 187. 1753.

252DA974-701E-584B-9382-09A209505306

**Figure 41.**
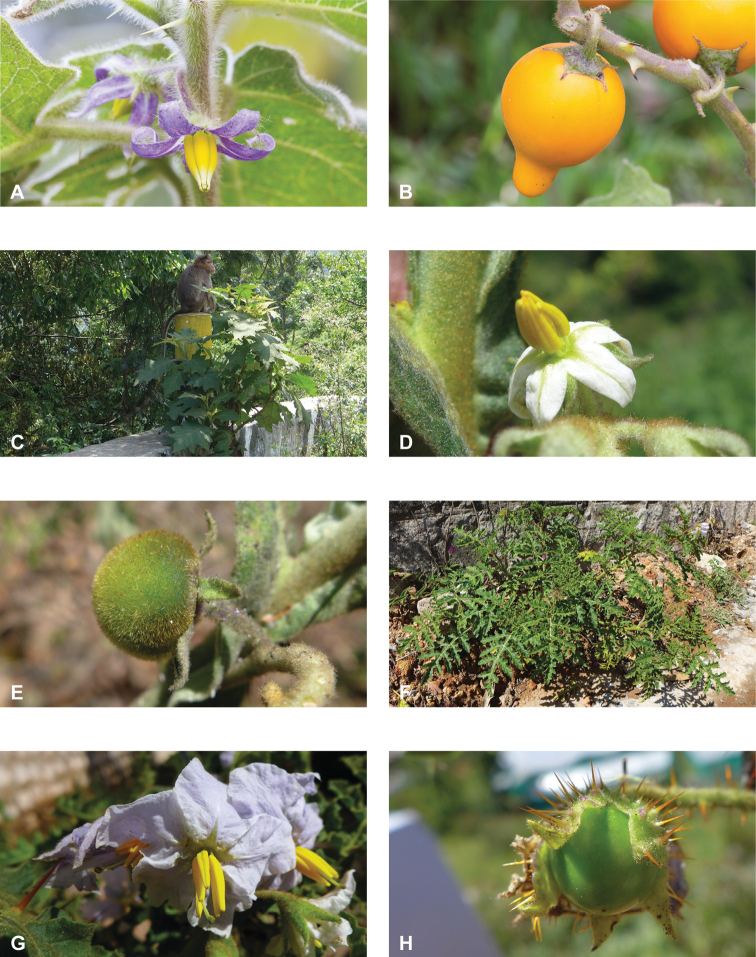
Introduced species of *Solanum*. *Solanummammosum* L. **A** detail view of a flower (*Stevens 35067*, Nicaragua) **B** detail view of a fruit (*Stevens 35067*, Nicaragua). *Solanumrobustum* H.L.Wendl. **C** habit (*Sampath Kumar et al. 126920*, India) **D** detail view of a short-styled flower (*Sampath Kumar et al. 126920*, India) **E** detail view of a fruit (*Sampath Kumar et al. 126920*, India). *Solanumsisymbriifolium* Lam. **F** habit (*Sampath Kumar et al. 126922*, India) **G** detail view of a flower (*Sampath Kumar et al. 126922*, India) **H** detail view of a fruit (*Sampath Kumar et al. 126922*, India). Photograph credits: **A, B** O.M. Montiel **C–H** X. Aubriot.

Fig. 41A, B No names based on tropical Asian types have been published; for complete synonymy see Nee 1976; Vorontsova and Knapp 2016).

#### Type.

Barbados. “Solanum barbadense spinosum, foliis villosis, fructu aureo rotundiore, Pyri parvi inversiforma et magnitude” (lectotype, designated by Knapp and Jarvis 1990, pg. 343: [Illustration] Plukenet, Phytographia, pars tertia, tab. 226, fig. 1. 1692). Typotype material: Sloane vol. 98: 59 (BM-SL).

#### Distribution.

*Solanummammosum* has been recorded from China, Myanmar, the Philippines, Thailand, and Vietnam, but is almost certainly more widespread in the region; it is native to southern South America and is widely cultivated for ornament.

#### Common names.

China. ru qie (Zhang et al. 1994). Malaysia/Singapore. tĕrong bĕlanda, tĕrong asam (Burkill 1935). Vietnam. cà du, blanh (Hul and Dy Phon 2014)

#### Description.

Vorontsova and Knapp (2016: 235–239); http://www.solanaceaesource.org/solanaceae/solanum-mammosum-1.

#### Discussion.

*Solanummammosum* is widely cultivated for its unusual teat-shaped fruit that is often used in flower arrangements. It is a member of the Acanthophora clade along with *S.aculeatissimum*, *S.capsicoides*, and *S.viarum*. *Solanummammosum* differs from those species in its larger, purple flowers, densely pilose leaves and stems, and large fruits. *Solanumcapsicoides* has similarly large berries, but these are darker orange and the seeds are strongly winged; seeds of *S.mammosum* lack wings. Hul and Dy Phon (2014) record it as naturalised in Vietnam, where the fruits are apparently eaten by villagers once cooked, which is a bit surprising, given that they are used as insecticides in other parts of the world (Nee 1979).

#### Specimens examined.

See Suppl. materials 1–3.

### ﻿26. Solanum melongena

Taxon classificationPlantaeSolanalesSolanaceae

L., Sp. Pl. 1: 186. 1753.

02C8330D-A79A-5075-9444-56D4D1794644

Fig. 42

Solanum mexianum Hill, Veg. Syst. 9: 39, pl. 39, fig. 1. 1765. Type. Cultivated “The Rugged Nightshade” (no specimens cited; lectotype, designated here: [illustration] Hill, The Vegetable System 9: Tab 39, f. 1. 1765).
Melongena ovata Mill., Gard. Dict., ed. 8, no. 1. 1768. Type. “Melongena (Ovata) caule inermi herbaceo, foliis oblong-ovatis tomentosis integris, fructu ovato [Melongena fructu oblongo violaceo Tourn. Inst. 151]” (no specimens cited; possibly described from live plants; neotype, designated here: BM [BM000942564]).
Melongena teres Mill., Gard. Dict., ed. 8, no. 2. 1768, as “*Tereta*”. Type. “Melongena (*Tereta*) caule inermi herbaceo, foliis oblong-ovatis tomentose, fructu terete [Melongena fructu tereti violaceo Tourn. Inst. 151]” (no specimens cited, no material found; probably described from live plants).
Melongena incurva Mill., Gard. Dict., ed. 8, no. 3. 1768. Type. “Melongena (*Incurva*) caule inermi herbaceo, foliis oblongis sinuatis tomentosis, fructu incurvo [Melongena fructu incurvo Tourn. Inst. 152]” (no specimens cited, no material found; probably described from live plants).
Melongena spinosa Mill., Gard. Dict., ed. 8, no. 4. 1768. Type. “Melongena (Spinosa) spinosa, foliis sinuatis-lacinitis, fructu tereti, caule herbaceo [Solanum pomiferum fructu spinoso J.B. 3. 619]” (no specimens cited, no material found; lectotype, designated here: [illustration] “Solanum pomiferum fructu spinoso” Bauhin, Historia plantarum 3: 619. 1651).
Solanum zeylanicum Scop., Delic. Fl. Faun. Insubr. 1: 1. 1786. Type. Cultivated in Italy [Pavia] from seeds sent by D. Marsilius (no specimens cited, described from live plants; lectotype, designated here: [illustration] Scopoli, Deliciae Flora et Fauna Insubricae 1: tab. 1. 1786).
Solanum album Lour., Fl. Cochinch. 129. 1790. Type. Indonesia. Malaku Islands: Amboina [Ambon Island] (no specimens cited; based on illustration; lectotype, designated here [cited as holotype by Hul and Dy Phon 2014, pg. 30]: [illustration] “Trongum agreste spinosum” in Rumphius, Herb. Amboin. 5: 241, tab. 86, fig. 1. 1747).
Solanum oviferum Nocca, Ann. Bot. (Usteri) 6: 61. 1793., nom. illeg. superfl. Type. Based on Solanummelongena L. (cited in synonymy)
Solanum oviferum Salisb., Prodr. Stirp. Chap. Allerton 134. 1796, nom. illeg. superfl. Type. Based on Solanummelongena L. (cited in synonymy)
Solanum esculentum Dunal, Hist. Nat. Solanum 208, tab. 3. 1813, nom. illeg. superfl. Type. Based on Solanummelongena L. (cited in synonymy with the comment [Dunal 1852] “nomen Melongena no accepi, quia sub hoc nomine, species plures confusae suerunt”).
Solanum ovigerum Dunal, Hist. Nat. Solanum 210. 1813, nom. illeg. superfl. Type. Based on Solanummelongena L. (cited in synonymy)
Solanum pressum Dunal, Hist. Nat. Solanum 217. 1813. Type. Based on “Trongum prâ rubrum” of Rumphius (no specimens cited; lectotype, designated here: [illustration] Rumphius, Herbarium Amboinense 5: tab. 86, f. 2. 1747).
Solanum melanoxylon Link, Enum. Hort. Berol. Alt. 1: 188. 1821. Type. Cultivated in Berlin, Germany (no specimens cited; no original material found, likely destroyed).
Solanum longum Roxb. ex Wall., Fl. Ind. (Carey & Wallich ed.) 2: 248. 1824. Type. India. West Bengal: Cultivated “HBC” [Hort. Bot. Calcutta], 1821, *Without collector s.n. [Wallich cat. 2628D*] (lectotype, designated here: K-W [K001116666]).
Solanum ovigerum Dunal var. insanum Blume, Bijdr. Fl. Ned. Ind. 13: 698. 1826. Type. Indonesia. “in hortis frequenter cultum/Terong Pangang” (no specimens cited; no original material found).
Solanum pseudo-undatum Blume, Bijdr. Fl. Ned. Ind. 13: 699. 1826. Type. Indonesia. Java. “in locis cultis/nomen Terong Rangu” [ex protologue] Sin. loc., *C.L. Blume s.n.* (lectotype, designated here: L [L0003642]).
Solanum pseudo-undatum Blume var. albiflorum Blume, Bijdr. Fl. Ned. Ind. 13: 699. 1826. Type. Indonesia. “in hortis colitur/Terong lelles” [ex protologue] (no specimens cited; no original material found).
Solanum pseudo-undatum Blume var. atropurpurascens Blume, Bijdr. Fl. Ned. Ind. 13: 699. 1826. Type. Indonesia. “cum praecidentibus/Terong Kupa” [ex protologue] (no specimens cited; no original material found).
Solanum pseudo-undatum Blume var. leucocarpon Blume, Bijdr. Fl. Ned. Ind. 13: 700. 1826. Type. Indonesia. “montis Salak” [ex protologue] (no specimens cited; no original material found).
Solanum edule Schumach. & Thonn., Beskr. Guin. Pl. 125 [145]. 1827. Type. “Guinea”. Sin. loc., cultivated, *P. Thonning 141* (lectotype, designated here: C [C10004584]).
Solanum melongena L. var. esculentum (Dunal) Walp., Repert. Bot. Syst. (Walpers) 3: 81. 1844. Type. Based on Solanumesculentum Dunal.
Solanum aethiopicum L. var. violaceum Dunal, Prodr. [A. P. de Candolle] 13(1): 351. 1852. Type. Based on Solanumaethiopicum sensu Lour., non L.
Solanum album Lour. var. richardii Dunal, Prodr. [A. P. de Candolle] 13(1): 362. 1852. Type. Réunion. “Bisom. croix dans les lieux habités”, 1837, *A. Richard 186* (holotype: P [00352546]).
Solanum album Lour. var. rumphii Dunal, Prodr. [A. P. de Candolle] 13(1): 361. 1852, nom. illeg. Type. Based on Solanumalbum Lour. (as “Trongum agreste”).
Solanum edule Thonn. var. multifidum Dunal, Prodr. [A. P. de Candolle] 13(1): 357. 1852. Type. Cultivated “in hort. Tonelle Audiberti cultum” [Audibert nursery in Tonelle, near Tarascon, France], sin. dat., *Without collector s.n.* (holotype: AV [n.v.]).
Solanum serpentinum Desf. ex Dunal, Prodr. [A. P. de Candolle] 13(1): 358. 1852. Type. Cultivated in Montpellier, France, 17–26 Aug 1838, *R.L. Desfontaines s.n.* (holotype: MPU [MPU854428]).
Solanum esculentum Dunal var. aculeatum Dunal, Prodr. [A. P. de Candolle] 13(1): 355. 1852. Type. Indonesia. Java: Sin. loc., sin. dat., *H. Zollinger 702* (holotype: G-DC [G00131553]; isotypes: BM [BM000778111], K [K000788269]).
Solanum esculentum Dunal var. inerme Dunal, Prodr. [A. P. de Candolle] 13(1): 355. 1852. Type. Brazil. Bahia: sin. loc., sin dat., *J.S. Blanchet 368* (lectotype, designated here: G [G00357998]).
Solanum esculentum Dunal var. subinerme Dunal, Prodr. [A. P. de Candolle] 13(1): 355. 1852. Type. Brazil. Bahia: sin. loc., 1831, *J.S. Blanchet 236* (lectotype, designated here: G-DC [G00131527]).
Solanum lagenarium Dunal, Prodr. [A. P. de Candolle] 13(1): 368. 1852. Type. Cultivated “hort. Tonelle cultum” [Audibert nursery in Tonelle, near Tarascon, France], 1824, *Anon. s.n.* (holotype: AV [n.v.]).
Solanum ovigerum Dunal [unranked] *sinuatorepandum* Dunal, Prodr. [A. P. de Candolle] 13(1): 358. 1852. Type. Cuba. La Habana: “La Havane” [Havana], 1826, *de la Ossa s.n.* (lectotype, designated here: G-DC [G00131521]).
Solanum ovigerum Dunal [unranked] *subrepandum* Dunal, Prodr. [A. P. de Candolle] 13(1): 358. 1852. Type. Cultivated in Geneva, Switzerland “in tepidario DC.”, sin. dat., *Anon. s.n.* (lectotype, designated here: G-DC [G00131523]).
Solanum ovigerum Dunal var. violaceum Dunal, Prodr. [A. P. de Candolle] 13(1): 358. 1852, as Solanumovigerum [unranked] *subrepandum*var. violaceum. Type. No specimens or locality cited; reference to Tournefort (“Tourn. inst. 152”).
Solanum ovigerum Dunal var. oblongo-cylindricum Dunal, Prodr. [A. P. de Candolle] 13(1): 358. 1852, as Solanumovigerum [unranked] *subrepandum*var. oblongo-cylindricum. Type. Based on Solanumovigerumvar.insanum Blume.
Solanum trongum Poir. var. sinuatopinnatifidum Dunal, Prodr. [A. P. de Candolle] 13(1): 361. 1852. Type. Based on Solanumtrongum Poir. sensu Blume [Java. “in agestribus/karnadong”, *C. Blume s.n.* (no specimens cited, based on S.trongum Poir. sensu Blume)].
Solanum requienii Dunal, Prodr. [A. P. de Candolle] 13(1): 363. 1852., as ‘Requieni’. Type. Cultivated in France “hort. Tonelle ex Audibert fratr.”, 1822, *Anon. s.n.* (holotype: AV [n.v.]).
Solanum sativum Dunal, Prodr. [A. P. de Candolle] 13(1): 360. 1852, nom. illeg. superfl. Type. Based on Solanumpseudo-undatum Blume (cited in synonymy “Nomen pseudo-undatum mutavi, quonian semigraecum, semilatinem”)
Solanum sativum Dunal var. albiflorum (Blume) Dunal, Prodr. [A. P. de Candolle] 13(1): 360. 1852. Type. Based on Solanumpseudo-undatumBlumevar.albiflorum Blume.
Solanum sativum Dunal var. atropurpurascens (Blume) Dunal, Prodr. [A. P. de Candolle] 13(1): 360. 1852. Type. Based on Solanumpseudo-undatumBlumevar.atropurpurascens Blume.
Solanum sativum Dunal var. leucocarpon (Blume) Dunal, Prodr. [A. P. de Candolle] 13(1): 361. 1852. Type. Based on Solanumpseudo-undatumBlumevar.leucocarpon Blume.
Solanum melongena L. var. giganteum Alef., Landw. Fl. 136. 1866, as “*gigantea*”. Type. Cultivated (no specimens cited; no original material found).
Solanum melongena L. var. leucoum Alef., Landw. Fl. 136. 1866. Type. Cultivated (no specimens cited; no original material found).
Solanum melongena L. var. stenoides Alef., Landw. Fl. 136. 1866. Type. Cultivated (no specimens cited; no original material found).
Solanum melongena L. var. stenoleucum Alef., Landw. Fl. 136. 1866, as “*stenoleuca*”. Type. Cultivated (no specimens cited; no original material found).
Solanum melongenum St.-Lag., Ann. Soc. Bot. Lyon 7: 135. 1880, nom. illeg. superfl. Type. Based on Solanummelongena L. (cited in synonymy).
Melongena esculenta (Dunal) Grecescu, Consp. Fl. Romaniei 423. 1898. Type. Based on Solanumesculentum Dunal, nom. illeg.
Solanum melongena L. var. inerme (Dunal) Hiern, Cat. Afr. Pl. (Hiern) 1(3): 748. 1898. Type. Based on Solanumesculentumvar.inerme Dunal
Solanum melongena L. var. viride Dikii, Trudy Prikl. Bot. 88: 104. 1984, as “*viridis*”. Type. Russia. Cultivated at Maikop Experiment station of VIR (Maikop, Krasnodar Krai, Republic of Adygea), originally from Asia Minor. 4 Jul 1971, *L. Studentsova s.n. [VIR cat. # 249*] (holotype: WIR [n.v.]).
Solanum melongena L. subsp. agreste Dikii, Trudy Prikl. Bot. Genet. Selek. 88: 105. 1984, as “agrestis”. Type. Russia. Cultivated at Maikop Experiment station of VIR (Maikop, Krasnodar Krai, Republic of Adygea), originally from India, 7 Aug 1975, *N. Frantskevich s.n. [VIR cat. # 713*] (holotype: WIR [n.v.]).
Solanum melongena L. var. angustum Dikii, Trudy Prikl. Bot. Genet. Selek. 88: 105. 1984, as “*angusta*”. Type. Russia. Cultivated at Maikop Experiment station of VIR (Maikop, Krasnodar Krai, Republic of Adygea), originally from India “c.v. R.R.I.”, 7 Aug 1975, *N. Frantskevich s.n. [VIR cat. # 2333*] (holotype: WIR [n.v.]).
Solanum melongena L. var. cylindricum (Filov) Dikii, Trudy Prikl. Bot. Genet. Selek. 88: 105. 1984, as “*cylindrica*”. Type. Russia. Cultivated at Maikop Experiment station of VIR (Maikop, Krasnodar Krai, Republic of Adygea), originally from India, 7 Aug 1975, *N. Frantskevich s.n. [VIR cat. # 116*] (holotype: WIR [n.v.]).
Solanum melongena L. var. racemiflorum Dikii, Trudy Prikl. Bot. Genet. Selek. 88: 105. 1984, as “*racemiflora*”. Type. Russia. Cultivated at Maikop Experiment station of VIR (Maikop, Krasnodar Krai, Republic of Adygea), originally from India “c.v Purple Cluster”, 27 Sep 1958, *V. Barkovskaya s.n. [VIR cat. # 2515*] (holotype: WIR [n.v.]).
Solanum melongena L. var. racemosum (Filov) Dikii, Trudy Prikl. Bot. Genet. Selek. 88: 105. 1984, as “*racemosa*”. Type. Russia. Cultivated at Maikop Experiment station of VIR (Maikop, Krasnodar Krai, Republic of Adygea), originally from India, 4 Jul 1971, *L. Studentsova s.n. [VIR cat. # 709*] (holotype: WIR [n.v.]).
Solanum melongena L. var. globosi Dikii, Trudy Prikl. Bot. Genet. Selek. 88: 106. 1984. Type. Russia. Cultivated at Maikop Experiment station of VIR (Maikop, Krasnodar Krai, Republic of Adygea), originally from Vietnam, 14 Jul 1971, *L. Studentsova s.n. [VIR cat. # 2582*] (holotype: WIR [n.v.]).

#### Type.

Cultivated in Uppsala, Sweden, *Anonymous s.n.* (lectotype, designated by Schönbeck-Temesy 1972, pg. 70: LINN [acc. # 248.28]).

#### Description.

Erect annual herb, 0.2–0.5 m, unarmed or more rarely prickly. Stems erect, terete, unarmed or occasionally prickly, moderately stellate-pubescent to glabrescent; prickles to 3 mm long, less than 0.5 mm wide at the base, straight, acicular, yellow-orange to dark brown, glabrous; pubescence a mixture of minute simple hairs and porrect-stellate trichomes, the stellate trichomes sessile to short-stalked, the stalks to 0.2 mm long, the rays 8–15, 0.3–0.7 mm long, the midpoints ca. same length as the rays or elongated to 1 mm; new growth moderately to densely stellate-pubescent, whitish green; bark of older stems glabrescent, green-brown to dark brown. Sympodial units difoliate, not geminate. Leaves simple, moderately lobed, the blades 10–23 cm long, 9–15 cm wide, 1.5–2 times longer than wide, ovate, chartaceous, drying discolorous, unarmed; adaxial surface yellow-green to red-brown, sparsely to moderately stellate-pubescent, occasionally glabrescent; abaxial surface glabrescent to moderately stellate-pubescent usually more pubescent than the adaxial surface, the trichomes porrect, sessile to short-stalked, the stalks to 0.2 mm long, the rays 5–8, 0.3–1 mm long, the midpoints ca. same length as the rays; major veins 4–7 pairs; base cordate to obtuse; margins lobed, the lobes 2(-3) on each side, 0.5–2 cm long, deltate, apically rounded, the sinuses extending 1/4–1/3 to the midrib; apex acute; petiole 1–10 cm long, 1/4–1/3(–2/3) of the leaf blade length, moderately stellate-pubescent to glabrescent, unarmed or prickly with 1–2 prickles. Inflorescences 6–15 cm long, apparently terminal or lateral, unbranched, with 1–8 flowers, 1–3 flowers open at any one time, moderately stellate-pubescent to glabrescent, with a mix of sessile and short-stalked stellate-porrect trichomes like those of the stems, unarmed; peduncle 0–80 mm long, unarmed; pedicels 0.8–3.5 cm long, 1–1.8 mm in diameter at the base, 1–2 mm in diameter at the apex, erect to pendent, unarmed or more rarely prickly with up to 5 prickles, moderately stellate-pubescent to glabrescent with porrect-stellate trichomes like those of the stems, articulated at the base; pedicel scars spaced 3–5 mm apart. Buds ovoid, the corolla ca. halfway exerted from the calyx before anthesis. Flowers 4–8-merous, heterostylous and the plants andromonoecious, with the lowermost flower long-styled and hermaphrodite, the distal flowers short-styled, staminate and usually somewhat smaller. Calyx with the tube 3–8 mm long in long-styled flowers, urceolate to deeply cup-shaped, 2–3 mm long in short-styled flowers, cup-shaped to elongate cup-shaped, the lobes in long-styled flowers 5–17 mm long 3–5 mm wide, in short-styled flowers 3.5–5 mm long, 2–2.5 mm wide, deltate to narrowly deltate, apically acute to long-acuminate, unarmed or more rarely prickly with up to 30 prickles, moderately stellate-pubescent, with porrect-stellate trichomes like those of the pedicels. Corolla 2.5–5 cm in diameter in long-styled flowers, 2.4–4 cm in diameter in short-styled flowers, white to mauve or purple, stellate, lobed 1/4–1/2 of the way to the base, the lobes 10–15 mm long, 8–13 mm wide in long-styled flowers, 5–8 mm long and 8–13 mm wide in short-styled flowers, broad-deltate, spreading at anthesis, not opening fully in long-styled flowers, sparsely stellate-pubescent abaxially, the trichomes porrect, sessile or stalked, the stalks to 0.2 mm, the rays 4–8, 0.2–0.7 mm long, the midpoints ca. same length as the rays. Stamens equal; anthers 5.5–7.5 mm long in long-styled flowers, 5.5–6 mm long in short-styled flowers, ca. 2 mm wide, connivent, tapering, yellow, glabrous, poricidal at the tips, the pores not elongating to slits with drying; filament tube 2–3 mm long, glabrous; free portion of the filaments 1.2–3 mm long, glabrous. Ovary conical, stellate-pubescent in upper part near the style base; style in long-styled flowers ca. 9 mm long, broad and straight, moderately stellate-pubescent in the lower half to 3/4, in short-styled flowers 2–3 mm long, pubescent at the base; stigma globose capitate to somewhat bilobed, bright green in live plants, the surface minutely papillate. Fruit a globose to ovoid, ellipsoid, or oblong to variously curved berry (many cultivars with a wide variety of fruit shapes), 1 (rarely > 1) per infructescence, 3–20 cm long, 3–7 cm wide, green, sometimes mottled or striped, white, pink, mauve, purple, or black when young, usually white or maroon at maturity, the pericarp smooth, glabrous; fruiting pedicels 2.5–8 cm long, 2–4 mm in diameter at the base, 5–10 mm in diameter at the apex, unarmed or sparsely prickly, woody, pendent and deflexed from the weight of the berry; fruiting calyx lobes elongating to 12–50 mm long, 1/4–1/3 the length of the mature fruit, often cup-shaped around the fruit in some cultivars, unarmed or sparsely prickly. Seeds usually aborted in cultivars. Chromosome number: n = 12 (Doganlar et al. 2002; Barchi et al. 2019; Chapman 2019; Gramazio et al. 2019; Wei et al. 2020).

**Figure 42.**
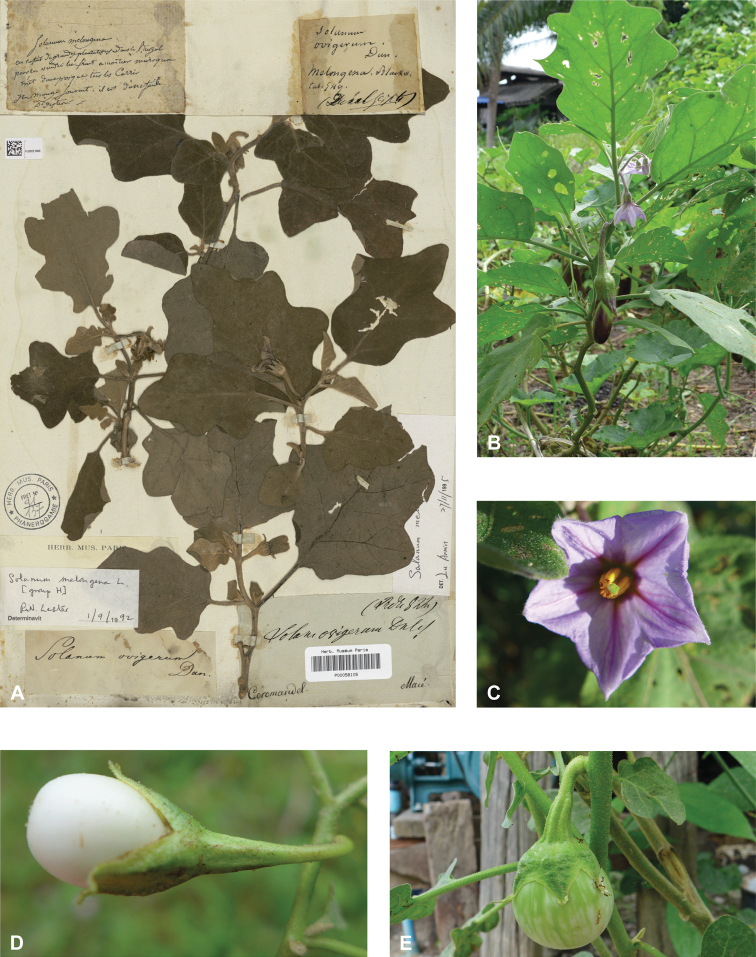
*Solanummelongena* L. **A** herbarium specimen collected in India (*Macé s.n.*, P00058105) **B** habit (field photograph, unvouchered, Thailand) **C** detail view of a hermaphroditic flower (*Meeboonya et al. RM 294*, Thailand) **D** detail view of an oblong and white fruit (*Meeboonya et al. RM 250*, Thailand) **E** detail view of a round green fruit marbled with white (*Meeboonya et al. RM 248*, Thailand). Photograph credits: **A** CC-BY, Muséum national d’Histoire naturelle, Paris **B–E** X. Aubriot.

#### Distribution.

*Solanummelongena* is cultivated worldwide in tropical and subtropical areas (in the temperate zone under glass); the greatest diversity of landraces and cultivars is found in Asia (India, China and southeast Asia), with secondary centres in the Middle East and around the Mediterranean. The origin of *Solanummelongena* is in Asia, but the exact place of domestication in not clear (see Meyer et al. 2012; references in Knapp et al. 2013). We have not provided a distribution map as herbarium specimens do not accurately reflect the range of the species in cultivation.

#### Ecology and habitat.

*Solanummelongena* is only known from cultivation (see below).

#### Common names and uses.

China. qie (Zhang et al. 1994), qie zi (Lester and Hawkes 2001). India. brinjal [Hindi] (Lester and Hawkes 2001); Bihar: baigan, bhanta (Varma 1981); Karnataka: advibadani, doorla (Singh 1988); Tamil Nadu: kathiri (Tamil, Matthew 1983); kaththiri ([Tamil] Henry et al. 1987). Japan. nasu (Lester and Hawkes 2001). Indonesia. Java: enchung, térong (Burkill 1935); Sumatra: tiung, chung (Burkill 1935). Malaysia/Singapore. tĕrong (with many qualifiers indicating varieties, Burkill 1935). *Solanummelongena* is a worldwide food crop; after tomato (*S.lycopersicum* L.) it is the most widely cultivated fruit crop in the Solanaceae (Daunay and Janick 2007; Knapp et al. 2019b). Recent work to improve eggplants in the face of climate change has seen increased interest in wild relatives and crosses with wild species across the spiny solanums (e.g., García-Fortea et al. 2019). See section on Uses (pp. 18–20) for further details. Discussion of the many aspects of eggplant biology can be found in Chapman (2019).

#### Preliminary conservation status

**(IUCN 2019).***Solanummelongena* is known primarily from cultivation, and as such we have not assigned it a threat status using the IUCN criteria.

#### Discussion.

*Solanummelongena* is known to cross easily with its wild progenitor *S.insanum* (Davidar et al. 2015; Mutegi et al. 2015) and distinguishing the two taxa can be difficult especially in older specimens or in feral populations. We have chosen to recognise the two as distinct species rather than as infraspecific taxa (e.g., Lester and Hasan 1991; Daunay and Hazra 2012) because cultivated and wild plants are on distinct evolutionary trajectories. Wild plants are subject to natural selection, while cultivated plants are commensal with human beings. We recognise that this can lead to difficulties in identifying individual specimens, particularly those from old collections where details of provenance are not clear.

Distinguishing the cultivated *S.melongena* from wild *S.insanum* can be particularly difficult in the absence of fruits. Keys in previous publications (Knapp et al. 2013; Ranil et al. 2016) can be used for identification, but in general specimens we assign to *S.insanum* are pricklier, with smaller flowers and fruit, and more ruderal than those we assign to *S.melongena*. Flowers of *S.melongena* are often fasciated (with more than 5 parts), but usually are not in local cultivars. Local cultivars of *S.melongena* in tropical Asia can be especially difficult to distinguish from *S.insanum*.

The only original material we have found for Sir John Hill’s *Solanummexianum* is his illustration (Hill 1765) that clearly represents *S.melongena*; we lectotypify the name with this illustration.

Miller (1768) recognised the pre-Linnaean genus *Melongena*, in which he segregated those species treated as *Solanum* by Linnaeus (1753) as *S.melongena*. His species were distinguished based mainly on fruit size and shape; he considered colour variants to all belong to his *Melongenaovata*. He did not cite any specimens in his treatment, he cited instead pre-Linnaean polynomials mostly from Tournefort (1700), none of which are illustrated in that work. Original material is thus apparently lacking for these names. From Miller’s (1768) discussion of *Melongena* he had obviously cultivated all of the taxa he recognised at the Chelsea Physic Garden. A single specimen of *S.melongena* from the Miller herbarium has been found in BM, labelled on the verso side with “Hort. Chelsea, Miller” and “Melongenaovata” in Solander’s handwriting, indicating it was so filed in Miller’s herbarium (J. Wajer, person. comm.). We designate this here as the neotype for *Melongenaovata*, as it is not clear it is original material; all of these species of *Melongena* are certainly described from living material, as were most of the plants in the “Gardener’s dictionary” (Miller 1768). We have not designated neotypes for *Melongenateres* and *M.incurva*; they should perhaps be sought in the Tournefort herbarium in Paris. *Melongenaspinosa* was described (Miller 1768) with reference to Bauhin’s (1651) “Historia plantarum” (“Solanum pomiferum fructu spinoso J.B. 3. 619”). In that work there is an illustration labelled “Solanum pomiferum fructu spinoso” that is clearly *S.melongena*, with two fruits, one an ovoid shape with a prickly calyx and the other a different elongate shape with a non-prickly calyx. We designate this illustration as the lectotype for *M.spinosa*, it is the only original material we have found.

In describing *S.zeylanicum*Scopoli (1786) provided an extremely detailed description of the plant and an excellent illustration both clearly referrable to *S.melongena*, although to a prickly relatively small-fruited variety. Scopoli (1786) stated that the fruit pulp was white (“albida”) and the illustration shows large, pendent fruits.

In describing *S.album*, Loureiro (1790) referred only to “Trongum agreste. Album. Rumph. Amb. l. 8. c. 48. P. 241” and refers to its use “Virtus radices Ondontologica. Bacca edulis”. In Rumphius’s “Herbarium Amboinense” (Rumphius 1747) “Trongum agrestealbum/Trongum pra” comprises two white-flowered taxa, one with green fruits marbled with white and the other with red fruits, but the use in dentistry cited by Loureiro (1790) is part of the description of the previous taxon, “Trongum agreste/Trongum udan” (Rumphius 1747: 240). Hul and Dy Phon (2014) cited as “holotype” of *S.album* the illustration of “Trongum agreste spinosum” (Rumphius 1747: tab. 86, f. 1), a taxon described as part of “Trongum agreste”, not “Trongum agrestealbum”. No botanical materials have survived from Loureiro’s stay in Indochina (Merrill 1935), so a holotype is out of the question; we have designated as the neotype the illustration cited by Loureiro (1790) that was clearly available to him at the time he described *S.album* (see Turland et al. 2018, Art. 8.1). In his interpretation of the “Herbarium Amboinense”, Merrill (1917) cited a specimen that “almost certainly represents “*Solanumagreste*album” Rumph.” (*Robinson 286*, US acc. # 654604). This is a specimen of *S.lasiocarpum* Dunal and does not correspond with the illustrations in Rumphius (1747).

Dunal (1813) based his *S.pressum* entirely on Rumphius’ (1747) “Trongum prâ rubrum”, citing no other material and taking his description entirely from that of Rumphius. He took his epithet from the suggestion that “Trongum agrestealbum” was translatable to Latin as “Trongum pressum” (Rumphius 1747). We have selected the one element cited by Dunal (1813) as the lectotype of *S.pressum*; the illustration shows an unarmed plant with solitary flowers and berries. Rumphius’ (1747) use of the word “rubrum” may indicate the berries were red, thus perhaps making this name a synonym of *S.aethiopicum* L., but this is not mentioned in the description.

In the description of *S.longum*Roxburgh (1824) states that he distinguishes this from *S.melongena* by its elongate fruit form, in all other respects said to be exactly like the brinjal eggplant. Roxburgh (1814) first used the name *Solanumlongum* in his “Hortus Bengalensis”, which is a list of the plants growing in the Calcutta Botanical Garden; we have not found any unambiguous original material, nor any specimens with fruits as those in the description, the description may have come from live plants. We have selected as a neotype for *S.longum* specimen in the Wallich herbarium at Kew of a plant cultivated in the Calcutta Botanical Garden in 1821 (Wallich cat. 2628D) that has an immature fruit that looks like it might be of the elongate form (K001116666).

Unravelling the identities of the various taxa referrable to *S.melongena* described by Blume (1826) is complicated by the lack of specimens of these cultivated plants. We here typify only those names for which we have found unambiguous original material; from Blume’s descriptions the identity of the plants in question is clear. In describing S.ovigerumvar.insanum, he is apparently coining the varietal epithet as new, not citing the Linnaean name *S.insanum*. The citation of this as occurring in cultivation and having oblong, cylindrical fruit makes it a clear synonym of *S.melongena*, but no original material has been found. *Solanumpseudo-undatum* was also described from cultivated plants; here we designate a sheet in L collected by Blume and labelled as “Solanumpseudoundatum” as the lectotype (L 0003642). The three varieties of *S.pseudo-undatum*, var. albiflorum, var. atropurpurascens and var. leucocarpon, are distinguished purely on flower or fruit colour and common name (see above); we have found no original material for any of these.

Schumacher and Thonning (Schumacher 1827) described *S.edule* from plants cultivated in west Africa. Of the two collections labelled as *S.edule* in Thonning’s herbarium at Copenhagen, the sheet collected by Thonning himself (C10004584) is here selected as the lectotype, the other sheet, collected by P.E. Isert (C10004585) is less well preserved. Vorontsova and Knapp (2016) incorrectly cited the Thonning collection as “holotype”.

The protologue of S.esculentumvar.inerme (Dunal 1852) cites two Brazilian collections, one of which (*Salzmann 392* in G-DC, G00131550) was questioned as belonging to *S.melongena* by Dunal himself. We have therefore selected the other collection, *Blanchet 368* (G00357998) that bears a label in Dunal’s hand as the lectotype. Var. subinerme similarly was based on two collections; we have selected *Blanchet 236* (G00131527) as the lectotype because it has a label in Dunal’s hand saying “esculentum β 1842” and the other syntype, *Sieber 308* from Trinidad (represented by two sheets G00357997, G00131564) is not annotated by Dunal.

The infraspecific names Dunal (1852) coined under his *S.ovigerum* are presented in a manner different to most of the other infraspecific names in this work. The main infraspecific divisions, *subrepandum* and *sinuatorepandum*, are indicated with capital letters and are thus unranked; greek letters in this work indicate varietal rank. The varieties listed under [unranked] *subrepandum* are a mixture of polynomials and references to pre-Linnaean works, we do not typify these here. Dunal (1852) cited several collections in the protologue of [unranked] *sinuatorepandum*, *Wallich cat. 2628d*, *de la Ossa s.n.* and *Gaudichaud 475*. We have selected *de la Ossa s.n.* (G00131521) in G-DC as the lectotype, the Wallich specimen in G-DC is of poor quality and the Gaudichaud collection in Paris is a plant of *S.aethiopicum*. The protologue of [unranked] *subrepandum* mentions only “v.s. in h. DC”, making the identity of original material difficult. We have selected a specimen in G-DC that has a label in Dunal’s hand stating “A. folia subrepanda” as the lectotype for this name (G00131523).

The varietal names coined for the various fruit-shape and colour forms of *S.melongena* in Friedrich Alefeld’s “Landwirthschaftliche flora” (Alefeld 1866), a compendium of names for all plants cultivated in Germany, are most likely to have been based on living plants, the descriptions are minimal and no localities are cited. We have not typified these; they are more properly considered as cultivar names. In his work on the cultivated flora of the Soviet Union Filov (1958) coined many names, but many of these had neither description nor diagnosis and so are not validly published. The Russian taxonomist S.P. Dikii (1984) apparently thought Alefeld’s names were not validly published and re-published them all at the same rank (see Names not validly published).

#### Specimens examined.

See Suppl. materials 1–3.

### ﻿27. Solanum miyakojimense

Taxon classificationPlantaeSolanalesSolanaceae

T.Yamaz. & Takushi, J. Jap. Bot. 66: 46. 1991.

33954992-406A-5114-B888-68C2BFBFA169

Figs 2B , 43

#### Type.

Japan. Ryuku Islands: Cultivated in Nago, Okinawa Island [originally collected in Miyako Island [Miyakojima], Agarihetona], 20 Dec 1989, *A. Takushi [6400] s.n.* (holotype: TI [TI00085080]; isotypes: TI [TI00085081, TI00085082]).

#### Description.

Woody herb or subshrub, the branches to 1 m long, spreading and prostrate, armed or unarmed. Stems terete, unarmed or sparsely prickly and moderately to densely eglandular pubescent, the prickles, if present, 0.1–0.35 cm long, straight or very slightly curved, somewhat broad-based, the pubescence of sessile or short-stalked porrect-stellate trichomes, the stalks if present ca. 0.1 mm, the rays 5–8, 0.2–0.4 mm long, the midpoints absent or ca. 0.1 mm long, occasionally to 0.5 mm long; new growth densely (moderately in cultivation) pubescent with sessile or short-stalked porrect-stellate trichomes like those of the stems; bark of older stems pale tan-brown. Sympodial units difoliate, the leaves geminate or not geminate. Leaves simple or very shallowly lobed, the blades 1.2–4 cm long, 0.9–3 cm wide, 1.1–1.3 times longer than wide, broadly ovate to nearly orbicular, widest in the lower half, somewhat thick and fleshy, concolorous or slightly discolorous, unarmed or sparsely armed with a few straight prickles to 0.2 cm long; adaxial surfaces densely pubescent with the lamina still somewhat visible, the pubescence of sessile and short-stalked porrect-stellate trichomes, the stalks 0.1–0.3(0.5) cm long, the rays 5–8, ca. 0.5 mm long, the midpoints absent or 0.3–0.5 mm long, the prickles few or absent; abaxial surfaces densely pubescent with the lamina completely obscured, the pubescence of sessile and short-stalked porrect-stellate trichomes, the stalks usually longer than those of the adaxial surface trichomes, 0.2–0.5 mm long, the rays 6–10, 0.5–0.6 mm long, the midpoints ca. 0.5 mm long; principal veins 2–3 pairs, only visible on the adaxial surfaces; base acute; margins entire or very shallowly lobed, the lobes rounded at the tips, the sinuses reaching less than 1/3 of the way to the midrib, rounded; apex obtuse and rounded; petioles 0.3–0.9 cm long, densely stellate-pubescent like the stems and leaves. Inflorescences internodal, 0.1–0.3(–1.5) cm long, occasionally longer but always with a flower emerging from the very base, unbranched and usually looking like a tiny spur on the stem, the 1–2(4) flowers often seeming to emerge directly from stem, 1–2 flowers open at any one time, ; densely stellate-pubescent with sessile and short-stalked porrect-stellate trichomes, the stalks if present ca. 0.1 mm, the rays 5–8, 0.2–0.4 mm long, the midpoints absent or ca. 0.1 mm long, occasionally to 0.5 mm long; peduncle absent to 1.5 mm long; pedicels ca. 0.9 cm long, ca. 0.8 mm in diameter at the base, ca. 1 mm in diameter at the apex, abruptly narrowing to calyx base, densely to moderately pubescent with sessile and short-stalked porrect-stellate trichomes like the stems, articulated at the base; pedicel scars tightly packed at tip of minute inflorescence, with the lowermost one near the stem. Buds ellipsoid, the corolla ca. halfway included in the calyx tube until just before anthesis. Flowers 5-merous, apparently all perfect. Calyx tube 2.5–3.5 mm long, cup-shaped, the lobes mere enations on the rim to 1 mm long, broadly deltate, tearing during development, densely to moderately pubescent with sessile and short-stalked porrect-stellate trichomes, the stalks if present ca. 0.1 mm, the rays 5–8, 0.2–0.4 mm long, the midpoints absent or ca. 0.1 mm long, occasionally to 0.5 mm long, the sinuses scarious, the tips rounded. Corolla 1–1.2 cm in diameter, white, stellate, lobed ca. 3/4 of the way to the base, the lobes 4.5–5 mm long, ca. 1.5 mm wide, spreading at anthesis, glabrous adaxially, densely pubescent where exposed in bud with tangled sessile and short-stalked porrect-stellate trichomes, the stalks ca. 0.1 mm long, the rays 6–8, ca. 0.2 mm long, the midpoints absent. Stamens equal; filament tube minute; free portion of the filaments 0.25–0.3 mm long, glabrous; anthers 4–4.5 mm long, ca. 1.5 mm wide, strongly tapering, yellow, poricidal at the tips, the pores directed distally. Ovary conical, glabrous; style 5.5–6 mm long, slightly curved, white, sparsely pubescent in the lower half with weak sessile porrect-stellate trichomes; stigma minutely bilobed, the surfaces minutely pubescent. Fruit a globose to somewhat elongate berry, 1–1.5 cm in diameter, orange (with “green longitudinal bands” fide *Takushi s.n.*) when mature, the pericarp glabrous, shiny, thin and somewhat translucent; fruiting pedicels 1.2–1.5 cm long, ca. 1.5 mm in diameter at the base, ca. 2.5 mm in diameter at the apex, thickened and woody, curved downwards, glabrescent to sparsely stellate-pubescent; fruiting calyx slightly accrescent, woody, the tube 3–3.5 mm long, the lobes 2–2.5 mm long, splitting irregularly with incrassate margins, appressed to the base of the fruit. Seeds 20–60 per berry, 2–2.5 mm long, ca. 2 mm wide, flattened reniform, straw-yellow, the surfaces minutely pitted, the testal cells sinuate in outline. Chromosome number: not known.

**Figure 43.**
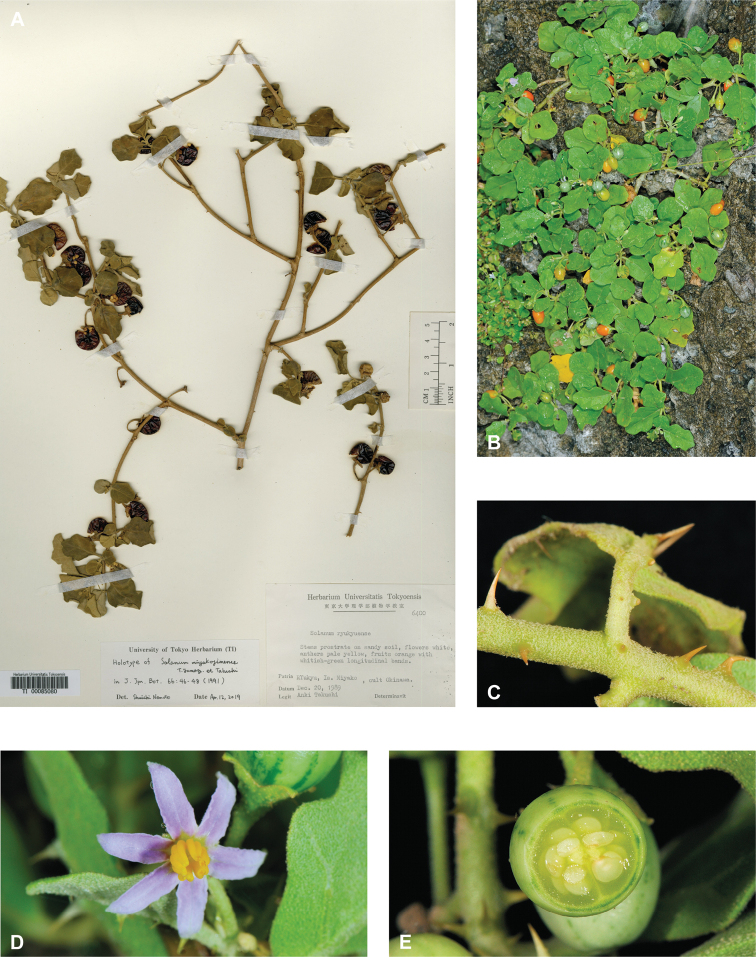
*Solanummiyakojimense* T.Yamaz. & Takushi **A** herbarium specimen (holotype) collected in Japan in 1989 (*Takushi [6400] s.n.*, TI00085080) **B** habit (*Hsu 16234*, Taiwan) **C** detail view of the prickles and the pubescence of a stem (*Hsu 16234*, Taiwan) **D** detail view of a flower (*Hsu 16234*, Taiwan) **E** detail view of a fruit with seeds inside (*Hsu 16234*, Taiwan). Photograph credits: **A** TI **B–E** M.-I. Weng.

#### Distribution

**Figure 44.**
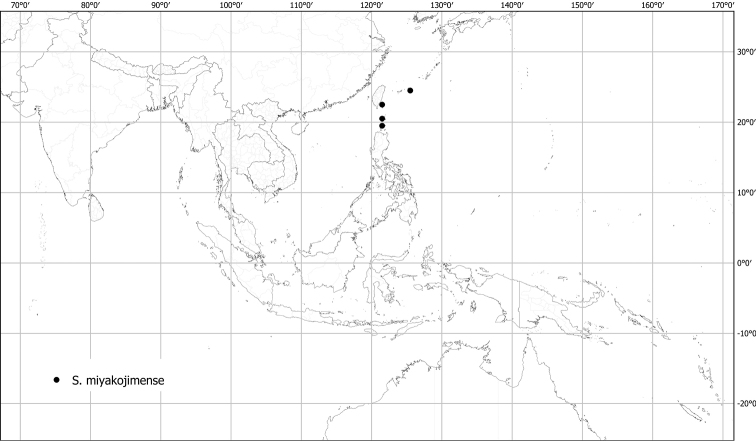
Distribution of *S.miyakojimense*.

**(Fig. 44).***Solanummiyakojimense* occurs from the southernmost Ryuku Islands of Miyako and Irabu (Okinawa prefecture) to the island of Ponso no Tao (Lan Yu or Orchid Island) off the southeastern coast of Taiwan to the islands of the Batanes group in the Philippines.

#### Ecology and habitat.

*Solanummiyakojimense* occurs in rocky coastal areas, often on coral bluffs (Hsu et al. 2007) near sea level; often occurring in sea spray.

#### Common names and uses.

None recorded.

#### Preliminary conservation status

**(IUCN 2019).** Endangered (EN [B1a,biii,iv]). EOO (641 km^2^, VU); AOO (40 km^2^, EN). *Solanummiyakojimense* is known from only a few wild collections scattered along the chain of islands across a large geographical distance, and although this makes the extent of occurrence larger than an endangered status might warrant, we consider the threats to habitats and the widely distant populations warrant this preliminary assessment.

#### Discussion.

In describing *S.miyakojimense*Yamazaki and Takushi (1991) suggested it was endemic to the Ryuku Islands; we have since found it to be more widely distributed, but still only along the island arc that runs south from Miyakojima Island to the Batanes Islands in the Philippines. *Solanummiyakojimense* is superficially similar to both *S.procumbens* and *S.violaceum*. It differs from *S.procumbens*, which occurs from China east to Thailand and the islands of Timor and Flores (Indonesia), in its seaside habitat; it can also be distinguished in its straight or only slightly curved stem prickles (versus strongly hooked), tiny inflorescences with only a few flowers with no or a very tiny peduncle (versus a longer thin peduncle), smaller flowers with broadly triangular corolla lobes (versus flowers with long-triangular corolla lobes), shorter pedicels in fruit and seeds with sinuate (versus pentagonal) cell walls. Berries of *S.procumbens* are bright red and globose at maturity, while those of *S.miyakojimense* are orange or orange-red and usually somewhat ellipsoid (Hsu et al. 2007).

The inflorescences of *S.miyakojimense* are shorter and fewer-flowered than those of the widespread *S.violaceum*, and the fruiting pedicels are conspicuously curved (not markedly spreading and straight as in *S.violaceum*) at fruit maturity. In *S.miyakojimense* the flowers are usually borne at the very base of the rhachis where it joins with the stem such that there is no clear peduncle; *S.violaceum* has a clearly pedunculate inflorescence. The leaves of *S.violaceum* are more markedly discolorous than those of *S.miyakojimense*. *Solanumviolaceum* is a relatively robust shrub that can reach 2 m tall, while *S.miyakojimense* is a prostrate plant. *Solanumviolaceum* is found in the northwestern part of the main island of Taiwan but has not been collected on Lan Yu (Orchid) island off the southeast coast. The only collections of *S.violaceum* from the Philippines are from cultivated plants.

#### Specimens examined.

See Suppl. materials 1–3.

### ﻿28. Solanum multiflorum

Taxon classificationPlantaeSolanalesSolanaceae

Roth, Syst. Veg., ed. 15 bis [Roemer & Schultes] 4: 669. 1819.

3460FB5B-23FA-5E6C-852C-CA5EB0CABAB5

Figs 3B , 45

Solanum agreste Roth in Roem. & Schult., Syst. Veg., ed. 15 bis [Roemer & Schultes] 4: 669. 1819. Type. India. “H. in India orientali. B. Heyne” 29 Jun 1816, *B. Heyne s.n.* (neotype, designated by Turner 2021, pg. 417: K-W [K00116650, shoot mounted in lower left only]).
Solanum multiflorum Roth, Nov. Sp. Pl. 129. 1821, nom. illeg. non Solanummultiflorum Roth in Roem. & Schultes, 1819. Type. Based on same material as S.multiflorum Roth ex Roem. & Schult.
Solanum agreste Roth, Nov. Pl. Sp. 130. 1821, nom. illeg. non S.agreste Roth in Roem. & Schult., 1819. Type. Based on same material and homotypic with S.agreste Roth in Roem. & Schult. (isonym).
Solanum himalense Dunal var. soumbe Dunal, Prodr. [A. P. de Candolle] 13(1): 300. 1852. Type. India. Tamil Nadu: “Nilgiris, Mts. de Nellygerry” [Nilgiri mountains], *J.B.L. Leschenault de la Tour 163* (lectotype, designated here: P [P00055586]; isolectotypes: P [P00055587, P00055588]).
Solanum erosum Van Heurck & Müll.Arg., Observ. Bot. (Van Heurck) 83. 1870. Type. India. Tamil Nadu: Nilgiris, “in montibus Nilagiri”, *R.F. Hohenacker 1074* (lectotype, designated here: BR [AWH10071571]; isolectotypes: BM [BM000778310, BM000778311], E [E00196408], G [G00442714], K [K000014858], L [L, L], LE [LE00016194], P [P00055723, P00055724, P00055725, P00055726], W [acc. # 0003339).
Solanum indicum L. var. multiflorum (Roth) C.B.Clarke, Fl. Brit. India [J. D. Hooker] 4: 235. 1883. Type. Based on Solanummultiflorum Roth
Solanum indicum L. subsp. erosum (Van Heurck & Müll.Arg.) Bitter, Repert. Spec. Nov. Regni Veg. Beih. 16: 11. 1923. Type. Based on Solanumerosum Van Heurck & Müll.Arg.
Solanum anguivi Lam. var. multiflorum (Roth) Vajr., Fl. Tamil Nadu Ind., Ser I: Analysis 2: 115. 1987, as “*multiflora*”. Type. Based on Solanummultiflorum Roth
Solanum violaceum Ortega subsp. multiflorum (C.B.Clarke) K.M.Matthew, Kew Bull. 46: 545. 1991. Type. Based on Solanummultiflorum Roth

#### Type.

India. [“H. in India orientali. B. Heyne” protologue] “Peninsula Ind. orientalis” [1836], *herb. R. Wight prop. 1575* (neotype, designated by Turner 2021, pg. 418: K-W [K000759413]; probable isoneotypes: CAL, E [E00757306, E00617617, E00617618, E00617619, E00617621), NY [NY00825634], P [P00049971, P00055603, P03933610]).

#### Description.

Erect shrub, 2–3 m, armed, much branched. Stems erect, robust, terete, densely stellate-pubescent and prickly, the trichomes porrect-stellate, sessile or short-stalked, the stalks to 0.8 mm long, the rays 6–8, 0.1–0.5 mm long, the midpoints to 2 mm long, the prickles to 10 mm long, to 6 mm wide, straight to slightly curved, flattened, orange-brown, glabrous or bearing a few stellate trichomes; new growth densely stellate-pubescent with trichomes like those of the stems; bark of older stems somewhat glabrescent, grey-brown. Sympodial units difoliate, the leaves geminate. Leaves simple, more or less deeply lobed, the blades 5–20 cm long, 3.5–16 cm wide, ca. 1.5 times longer than wide, ovate, chartaceous, discolorous, unarmed or prickly with up to 20 straight prickles to 10 mm long on primary and secondary veins on both sides of the leaf; adaxial surface densely to sparsely stellate-pubescent, with sessile to stalked porrect-stellate trichomes, the stalks to 0.8 mm, the rays 6–8, 0.2–0.8 mm, the midpoints 0.7–2 mm long; abaxial surface densely stellate-pubescent with sessile and short-stalked trichomes like those of the adaxial surface; major veins 3–5 pairs; base cuneate to truncate, often oblique; margins shallowly to deeply lobed, the lobes 3(4) on each side, 1.5–5 cm long, broad-deltate to obovate, often with well-developed secondary lobes, apically acute to rounded, the sinuses extending 1/3–4/5 to the midrib; apex acute; petioles 1–4 cm, 1/6–1/3 of the leaf length, unarmed or with a few straight prickles, densely stellate-pubescent with porrect sessile and short-stalked trichomes like those of the stems. Inflorescences 3–7 cm long, apparently lateral, unbranched or forked, more rarely several times branched, with 10–20 flowers, 3–6 flowers open at any one time, densely stellate-pubescent with stellate-porrect trichomes like those of the stems, unarmed or with a few straight prickles; peduncle 1–5 mm long, usually unarmed; pedicels 0.5–1.5 cm long, stout, ca. 1 mm in diameter at the base, ca. 1.5 mm in diameter at the apex, strongly recurved at anthesis, unarmed, densely stellate-pubescent like the inflorescence axes, articulated at the base; pedicel scars spaced 1–2 mm apart. Buds globose to ovoid, strongly exserted from the calyx before anthesis. Flowers 5-merous, apparently all perfect. Calyx with the tube 2–6 mm long, obconical to cup-shaped, the lobes 2–6 mm long, 1.5–2 mm wide, long-deltate, apically long-acuminate, unarmed or with a few straight prickles, densely stellate-pubescent with porrect-stellate trichomes like those of the pedicels. Corolla 1.3–1.5 cm in diameter, violet to purple, stellate, lobed ca. 2/3 of the way to the base, interpetalar tissue somewhat present, the lobes 4.5–6 mm long, 2.5–3 mm, deltate to ovate, spreading at anthesis, glabrous adaxially but with a few minute stellate trichomes at the tips and on the midribs, densely stellate-pubescent abaxially, the trichomes porrect, a mixture of sessile and stalked, the stalks up to 0.1 mm, the rays 6–8, 0.1–0.3 mm, the midpoints to 0.8 mm long, longer towards lobe apices. Stamens equal; anthers 3.5–4 mm, ca. 1.5 mm wide, tapering, drying brown to red-brown, sometimes with pronounced papillae on the dorsal surface, poricidal at the tips, the pores not elongating to slits with drying; filament tube minute; free portion of the filaments 0.5–1 mm, glabrous. Ovary conical, densely stellate-pubescent in the upper 3/4–1/2; style 5.5–7 mm long, stellate-pubescent for almost all of its length; stigma clavate, the surfaces minutely papillose. Fruit a globose berry, many per infructescence, 0.6–0.9 cm in diameter, orange-red to dark brown when ripe, the pericarp thin, smooth and shiny, with stellate trichomes sparsely covering the fruit pericarp persisting into maturity; fruiting pedicels 1–1.3 cm long, 0.7–1.5 mm wide at the base, 1.7–2.5 mm in diameter at the apex, woody, thick, strongly recurved downwards, unarmed or prickly with up to 5 filiform straight prickles; fruiting calyx moderately accrescent, elongating to (3.5–)5–11 mm long, covering up to 1/3 of the mature berry, usually not reflexed, unarmed or prickly with up to 10 straight prickles. Seeds ca. 10–15 per berry, 2.5–3.7 mm long, 2.1–3 mm wide, flattened-reniform, orange-brown, the surface minutely pitted, the testal cells pentagonal to slightly sinuate in outline. Chromosome number: n = 12, 2n = 24 (Madhavadian 1968; Kirti and Rao 1983; both as Solanumindicumvar.multiflorum Clarke).

**Figure 45.**
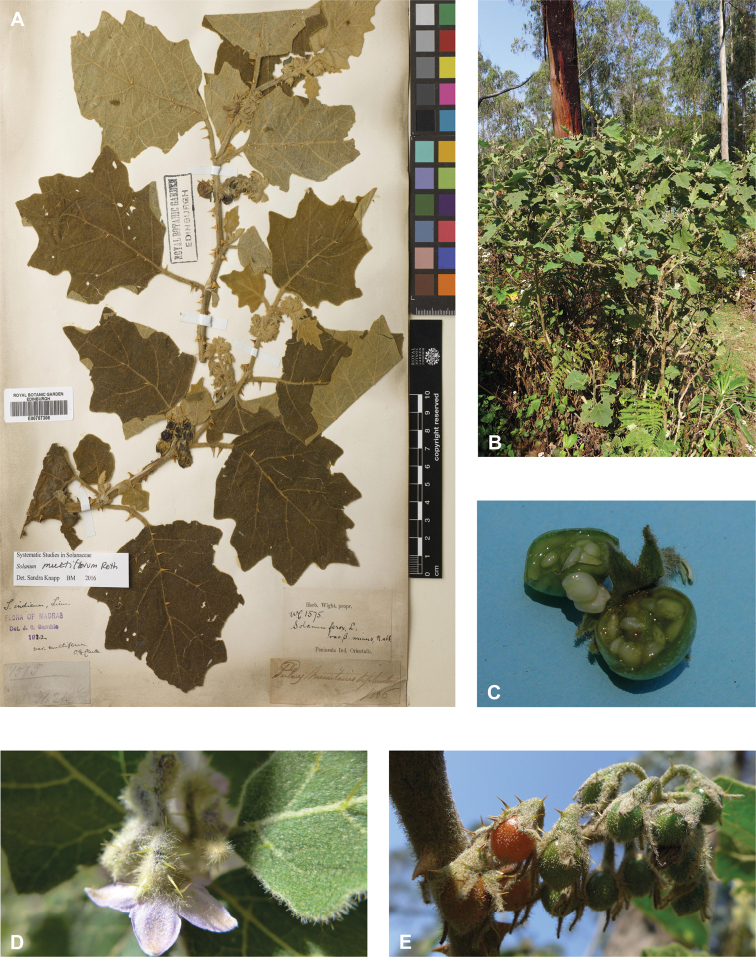
*Solanummultiflorum* Roth **A** herbarium specimen collected in India in 1835 (*Wight 1575*, E00757306) **B** habit (*Sampath Kumar et al. 126950*, India) **C** detail view of a fruit with seeds (*Sampath Kumar et al. 126958*, India) **D** detail view of a calyx and floral buds (*Sampath Kumar et al. 126937*, India) **E** detail view of an infructescence (*Sampath Kumar et al. 126950*, India). Photograph credits: **A** Royal Botanic Garden Edinburgh **B–E** X. Aubriot.

#### Distribution

**Figure 46.**
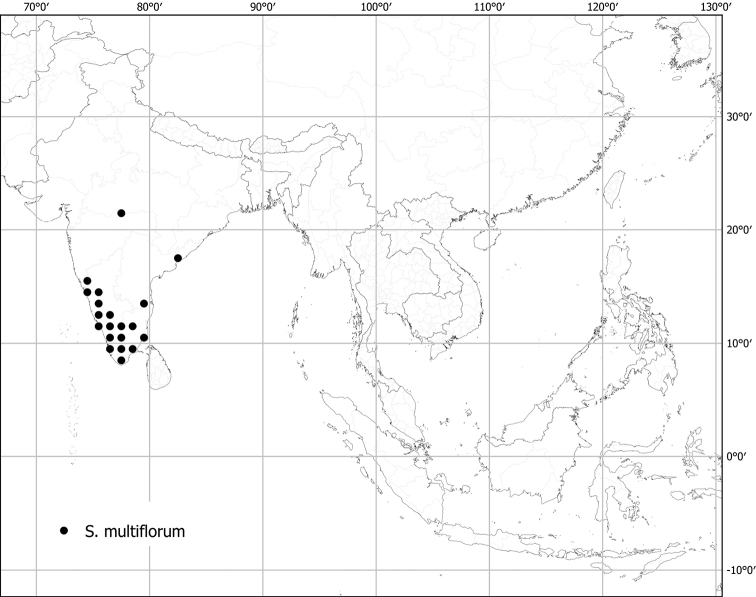
Distribution of *S.multiflorum*.

**(Fig. 46).***Solanummultiflorum* is endemic to the western part of Tamil Nadu Province in southern India, occurring in the Nilgiri, Coimbatore, Didingul and Madurai districts.

#### Ecology and habitat.

*Solanummultiflorum* is a plant of deciduous or evergreen broadleaf forests and grows in forest edges and roadsides; from 1,700 to 2,300 m elevation.

#### Common names and uses.

India. Karnataka: kahi sunde (*Hosagoudar 96871*); Tamil Nadu: soumbé (*Leschenault de la Tour 163*). Some of the names attributed to *S.indicum* in floras may refer to this species.

#### Preliminary conservation status

**(IUCN 2019).** Least Concern (LC). EOO (669,122 km^2^, LC); AOO (324 km^2^, EN). *Solanummultiflorum* is a species of disturbed habitats in southern India, with many recent collections. It is not of particular conservation concern.

#### Discussion.

*Solanummultiflorum* was treated as a variety of “*Solanumindicum*” (now a suppressed name, see Suppressed Names) in various Indian floras, in part due to issues with the application of Roth’s name. It is morphologically similar and related to *S.violaceum* (the name now used for the suppressed *S.indicum*), both taxa are members of *S.violaceum* group of Aubriot et al. (2016a), along with *S.hovei* and *S.deflexicarpum*. *Solanummultiflorum* is sympatric with *S.violaceum* and can be distinguished from it in its usually branched inflorescences with congested pedicels, long, soft pubescence and its short fruiting pedicels that are strongly deflexed rather than widely spreading. The leaves of *S.multiflorum* are usually more deeply lobed than those of *S.violaceum*, but *S.violaceum* is extremely variable and this does not always hold. *Solanummultiflorum* shares the strongly deflexed fruiting pedicels with *S.deflexicarpum* of China but differs from that species in its more copious pubescence, more deeply lobed leaves and usually purple (rather than white) flowers.

The species names proposed by Albrecht Wilhelm Roth were published in 1819 by Roemer and Schultes (1819), two years before they were published by Roth himself (Roth 1821). Descriptions in Roemer and Schultes (1819) are abbreviated from Roth’s longer ones published in 1821; no new material was apparently seen (see Turner 2021). Material at Edinburgh from Robert Wight’s personal herbarium (here as probable isoneotypes) is in better condition than the sheet (K000759413) in the Wallich hebarium designated as the neotype. The numbering of Wight’s herbarium was done after the fact specimens bearing the same number may or may not be duplicates of the neotype.

Solanumhimalensevar.soumbe was described based on the collection *Leschenault de la Tour 163* seen by Dunal (1852) in Paris. From the three specimens with this number at P we have selected the specimen (P00055586) with an annotation in Dunal’s hand as the lectotype.

The collection *Hohenacker 1074* was cited from two herbaria (“hb. Van Heurck et hb. DC”) in the protologue of *S.erosum* (Van Heurck 1870). The Van Heurck herbarium, previously held in Antwerp at AWH, has now been acquired by BR, but sheets are still barcoded with the AWH herbarium code. We have selected the duplicate of *Hohenacker 1074* held in BR (AWH10071571) as the lectotype for *S.erosum*; this collection is widely distributed.

#### Specimens examined.

See Suppl. materials 1–3.

### ﻿29. Solanum nienkui

Taxon classificationPlantaeSolanalesSolanaceae

Merr. & Chun, Sunyatsenia 2: 318. 1935.

51446CD3-08A7-5B36-B814-62D562D2DCE0

Fig. 47

#### Type.

China. Hainan: Sam Ah, 1932, *N.K. Chun & C.L. Tso 43319* (holotype: NY [00172269]; isotype: A [00077825]).

#### Description.

Erect shrub, to 4 m, unarmed or less often prickly. Stems erect, terete, unarmed or armed with small prickles, sparsely to densely stellate-pubescent, glabrescent; prickles to 3 mm long, to 3 mm wide at the base, curved, deltate, laterally flattened, pale yellow, glabrous; pubescence of mixed sessile and short-stalked porrect-stellate trichomes, the stalks to 0.25 mm long, the rays 4–8, 0.1–0.4 mm long, the midpoints up to 0.8 mm long; new growth densely stellate-pubescent, light brownish to brown in dry material; bark of older stems dark brownish, glabrous. Sympodial units plurifoliate, the leaves not geminate. Leaves simple, not lobed or less often shallowly lobed, the blades 3.5–10.5 cm long, 2.5–4 cm wide, ca. 1.5–2.5 times longer than wide, elliptic to broadly ovate, chartaceous, discolorous, unarmed or sometimes armed with small prickles, the prickles 1–9(–17) per leaf side, mostly inserted on the midvein, like those of the stems; adaxial surface moderately to densely stellate-pubescent, the stellate trichomes porrect, sessile or stalked, the stalks to 0.25 mm long, the rays 3–8, 0.1–0.5 mm long, the midpoints to 0.5 mm long, usually shorter than the rays; abaxial surface densely stellate-pubescent with trichomes like those of the adaxial surface; major veins 4–7 pairs, drying light green; base attenuate to short-attenuate; margins entire, sinuate, sometimes shallowly lobed when young, the lobes 2–3 on each side, 0.3–1 cm long, deltate, apically acute, the sinuses less than halfway to the midrib; apex acute; petioles 1.5–2.5 cm long, 1/7–1/3 of the leaf blade length, unarmed or prickly with 1–7 prickles like those of the stems, densely stellate-pubescent with sessile porrect trichomes like those of the stems. Inflorescences 2.5–7.5 cm long, internodal and lateral or more or less leaf-opposed, unbranched, with ca. 5–13 flowers, 1–3 flowers open at any one time, densely stellate-pubescent, with porrect stellate-trichomes like those of the stems, unarmed; peduncle 0.5–3 cm long, unarmed or armed with 1–3 prickles; pedicels 2.5–10 mm long, ca. 0.5 mm in diameter at the base, 0.75–1 mm in diameter at the apex, recurved, unarmed, densely stellate-pubescent with porrect stellate-trichomes like those of the axes, articulated at the base; pedicel scars spaced 0.1–0.6 mm apart. Buds ellipsoid, tapering, more or less strongly exserted from the calyx before anthesis. Flowers 5-merous, apparently all perfect. Calyx with the tube 1.2–1.5 mm long, campanulate, the lobes 0.5–2.5 mm long, 0.5–1 mm wide, deltate, acute at the apex, unarmed and densely stellate-pubescent with porrect stellate-trichomes like those of the pedicels. Corolla 0.8–1.3 cm in diameter, white to purplish blue, stellate, lobed ca. 4/5 of the way to the base, the lobes 5.5–8 mm long, 1–2.5 mm wide, deltate, spreading at anthesis, densely stellate-pubescent abaxially on parts exposed in bud. Stamens slightly unequal; anthers unequal, two to three of the five 4.5–5.5 mm long and two to three 3.5–4 mm long, all ca. 0.75 mm wide, tapering, connivent, yellow, glabrous, poricidal at the tips, the pores not lengthening to slits with age; filament tube <0.5 mm long, glabrous; free portions of the filaments all equal, ca. 0.1 mm long, glabrous. Ovary conical, minutely glandular-puberulent; style 5.5–6 mm long, slender, curved at the apex, glabrous; stigma capitate, minutely papillate. Fruit a globose berry, 1–4 per infructescence, 0.5–1 cm in diameter, the pericarp smooth, red when mature, glabrous; fruiting pedicels 0.7–1.2 cm long, ca. 0.5 mm in diameter at the base, ca. 2 mm in diameter at the apex, woody, spreading, unarmed; fruiting calyx lobes expanding to 5 mm long, 2/5–3/5 the length of the mature fruit, broadly to narrowly deltate, spreading to perhaps somewhat reflexed, unarmed, ending with a long acumen. Seeds 10–32 per berry, 2.5–3 mm long, ca. 2 mm wide, flattened-reniform, dull yellow, the surface minutely pitted, the testal cells sinuate in outline. Chromosome number: not known.

**Figure 47.**
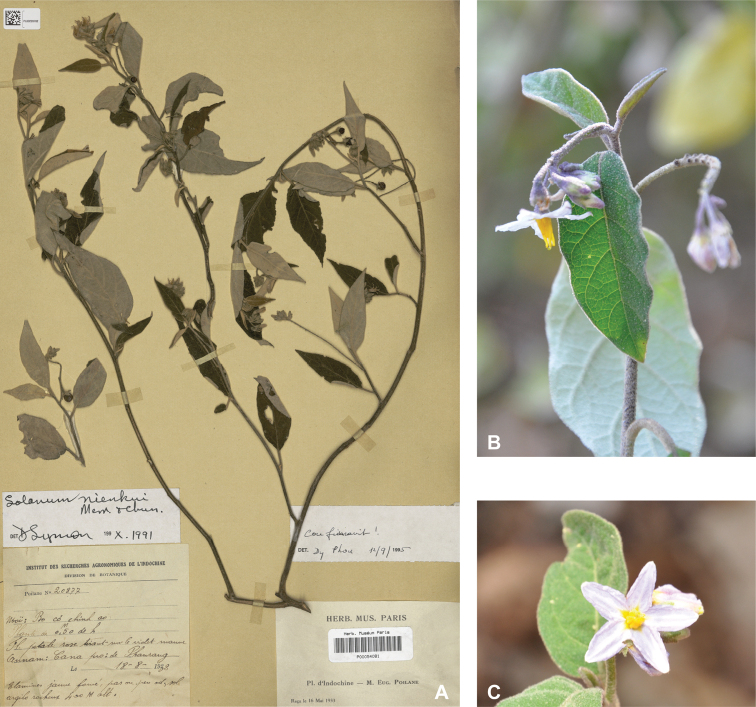
*Solanumnienkui* Merr. & Chun **A** herbarium specimen collected in Vietnam in 1932 (*Poilane 20877*, P00054081) **B** inflorescence and young stem (*Wang et al. 2073*, China) **C** detail view of a flower (*Wang et al. 2073*, China). Photograph credits: **A** CC-BY, Muséum national d’Histoire naturelle, Paris **B, C** S. Hul.

#### Distribution

**Figure 48.**
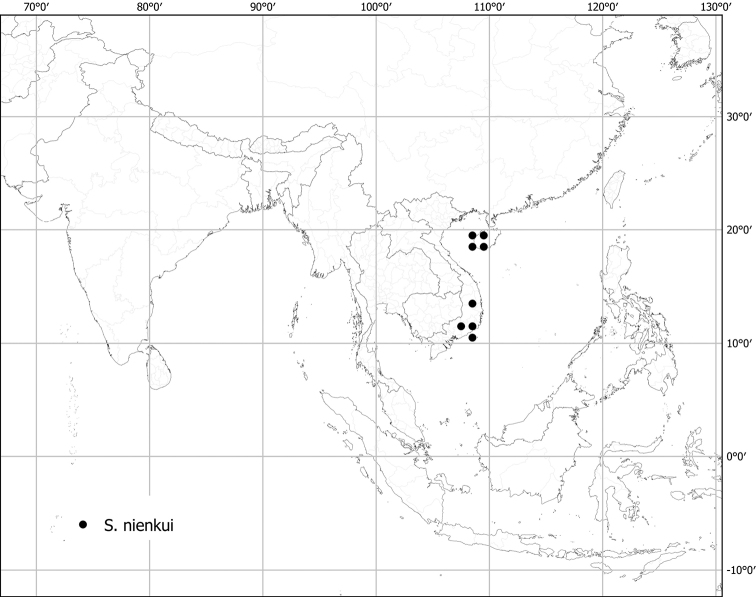
Distribution of *S.nienkui*.

**(Fig. 48).***Solanumnienkui* is found on the island of Hainan (South China) and in southern Vietnam.

#### Ecology and habitat.

*Solanumnienkui* is found in dry, deciduous forests, growing in more or less open areas on clay or sand, from 100 to 1,000 m elevation.

#### Common names and uses.

China. shu ci qie (Zhang et al. 1994); Vietnam. Gia Lai: trong nörvah [Jarai language] (*Dournes s.n.*); Ninh Thuân: bô kö chinh ao [Mnong language] (*Poilane 8933, 20877*), dan a xâm [Mnong language] (*Poilane 9887*), dan chinh ao [Mnong language] (*Poilane 9082*).

#### Preliminary conservation status

**(IUCN 2019).** Near Threatened (LC). EOO (64,999 km^2^, LC); AOO (72 km^2^, EN). *Solanumnienkui* is a plant of dry forests, often considered less valuable than wetter habitats and thus more often subject to anthropogenic change. Given the relatively small areas in which the species occurs and the potential habitat alteration, we have assigned a preliminary status indicating some threat.

#### Discussion.

*Solanumnienkui* is a weak shrub with deeply stellate flowers and a slightly zygomorphic androecium. It is morphologically similar to *S.robinsonii* and *S.putii*, and in the molecular analyses of Aubriot et al. (2016a) resolved as sister to *S.putii* in a group with *S.camranhense* that itself was sister to *S.cyanocarphium*. It differs from *S.robinsonii* (also found in Vietnam) in its wider, less discolorous leaves, deeply stellate, smaller corolla, and somewhat zygomorphic androecium. *Solanumcamranhense* has similar shaped leaves to those of *S.nienkui* but is a low creeping plant rather than an upright, spindly shrub.

#### Specimens examined.

See Suppl. materials 1–3.

### ﻿30. Solanum peikuoense

Taxon classificationPlantaeSolanalesSolanaceae

S.S.Ying in X.Aubriot & S.Knapp sp. nov.

A703A4F2-DAAB-5520-AA04-A3F6C8B5DD2C

urn:lsid:ipni.org:names:77298804-1

Fig. 49

#### Diagnosis.

Like *S.pseudosaponaceum*, but with the upper surfaces of leaves glabrate and shiny, inflorescences forked but not many times branched, larger flowers corolla (2–2.4 cm diam. versus 1–1.5 cm diam.) and generally fewer seeds per berry (18–40 versus 25->100).

#### Type.

Taiwan. Southern Taiwan: Pingtung County, Wutai Hsiang, 13 Oct 1993, *Y-R. Lin et al. 134* (holotype: HAST [29822]; isotype: BM [BM000846380], E [E00320675], MO [MO-3105792, acc. # 04669018]).

#### Description.

Erect shrub to small tree, to 4 m, unarmed, less often armed with a few prickles. Stems erect, terete, unarmed or with a few scattered prickles, sometimes dark purple (*Ku 1752*), sparsely stellate-pubescent; prickles, if present, 0.1–0.3 cm long, straight, deltate, orange-yellow, glabrous; pubescence of mixed sessile and variously stalked porrect-stellate trichomes, the stalks to 0.1 mm long, the rays 6–8, 0.1–0.5 mm long, the midpoints absent or up to 0.1 mm long; new growth densely stellate-pubescent, the trichomes like those of the stems; bark of older stems glabrescent, reddish brown and shiny. Sympodial units plurifoliate, the leaves not geminate. Leaves simple, very shallowly lobed, the blades 11–18 cm long, 5.2–10 cm wide, ca. 1.5–2 times longer than wide, elliptic to somewhat ovate, widest at the middle, chartaceous, strongly discolorous, unarmed or more rarely armed along the principal veins with tiny awl-shaped prickles like those of the stems; adaxial surface dark green, almost completely glabrous to sparsely stellate-pubescent along the midrib and veins but with a few sessile porrect-stellate trichomes on the midrib and along the principal veins, the rays 6–8, 0.3–0.5 mm long, the midpoints ca. 0.4 mm long, approximately equal to the rays; abaxial surface similarly but more densely pubescent with sessile porrect-stellate trichomes along the veins; major veins 5–7 pairs sparsely pubescent especially abaxially, drying pale yellow abaxially; base acute to attenuate, usually somewhat oblique; margins shallowly lobed, the lobes 5–7 on each side, 0.5–1 cm long, deltate and apically acute, the sinuses extending up to ca. 1/4 of the distance to the midvein; apex acute to acuminate; petiole 1.5–3.5 cm long, 1/9–1/5 of the leaf blade length, unarmed or with a few awl-shaped prickles, and very sparsely pubescent with porrect-stellate trichomes like those of the stems. Inflorescences 1.5–3.5 cm long, internodal and lateral, forked, with 10–16 flowers, some of these borne below the branching point, only a few flowers open at any one time, sparsely pubescent with porrect-stellate trichomes like those of the stems; peduncle ca. 1.3 cm long; pedicels 1–1.2 cm long, ca. 1 mm in diameter at the base, ca. 1.2 mm in diameter at the apex, spreading and perhaps slightly nodding at anthesis, sparsely stellate-pubescent with porrect-stellate trichomes like the inflorescence axes, articulated at the base; pedicel scars irregularly spaced (1)4–5 mm apart. Buds pointed-ovoid, the corolla exserted from the calyx lobe acumens before anthesis. Flowers 5-merous, apparently all perfect. Calyx with the tube ca. 2 mm long, cup-shaped, tearing to form irregular lobes, the lobes ca. 1 mm long with an elongate acumen to 3 mm long, ca. 1 mm wide, sparsely stellate-pubescent to glabrescent. Corolla 2–2.4 cm in diameter, white or purple (*Ku 1752*), stellate, lobed ca. 2/3 of the way to the base, the lobes 8–10 mm long, 3–4 mm wide, spreading at anthesis, glabrous adaxially, glabrous or sparsely stellate-pubescent along the petal midribs abaxially. Stamens equal; anthers 5–7 mm long, ca. 1.5 mm wide, tapering, yellow, poricidal at the tips, the pores directed distally, not elongating to slits with drying; filament tube minute, glabrous and papery; free portion of the filaments ca. 0.5 mm long, glabrous. Ovary conical, glabrous; style ca. 10 mm long, straight, glabrous; stigma minutely capitate, the surfaces minutely papillose. Fruit a globose berry, 2–6 per infructescence, 1–1.3 cm in diameter, the pericarp smooth, red or black (*Lin et al. 134*), glabrous; fruiting pedicels 1–2.3 cm long, 1–1.2 mm in diameter at the base, ca. 2 mm in diameter at the apex, woody, erect or spreading, unarmed; fruiting calyx lobes not markedly expanding, 1/3 the length of the mature fruit, spreading, broadly deltate with a filiform acumen to 3 mm long, this usually breaking off. Seeds 18–40 per berry, 3–3.5 mm long, 2–2.7 mm wide, flattened-reniform, dull yellow, the surfaces minutely pitted, the testal cells with sinuate margins. Chromosome number: not known.

**Figure 49.**
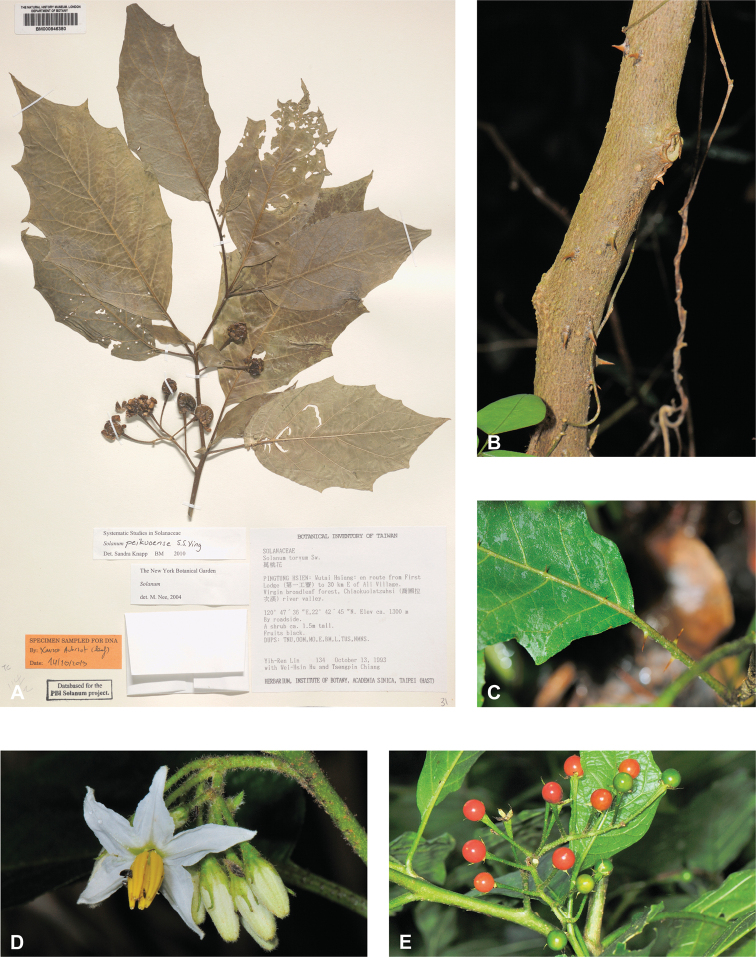
*Solanumpeikuoense* S.S.Ying **A** herbarium specimen collected in Taiwan in 1993 (*Lin et al. 134*, BM000846380) **B** old stem and prickles (field photograph, unvouchered, Taiwan) **C** detail view of leaf base and petiole (field photograph, unvouchered, Taiwan) **D** detail view of inflorescence and open flower (field photograph, unvouchered, Taiwan) **E** detail view an infructescence (field photograph, unvouchered, Taiwan). Photograph credits: **A** CC-BY, © copyright The Trustees of the Natural History Museum, London **B–E** M.-I. Weng.

#### Distribution

**Figure 50.**
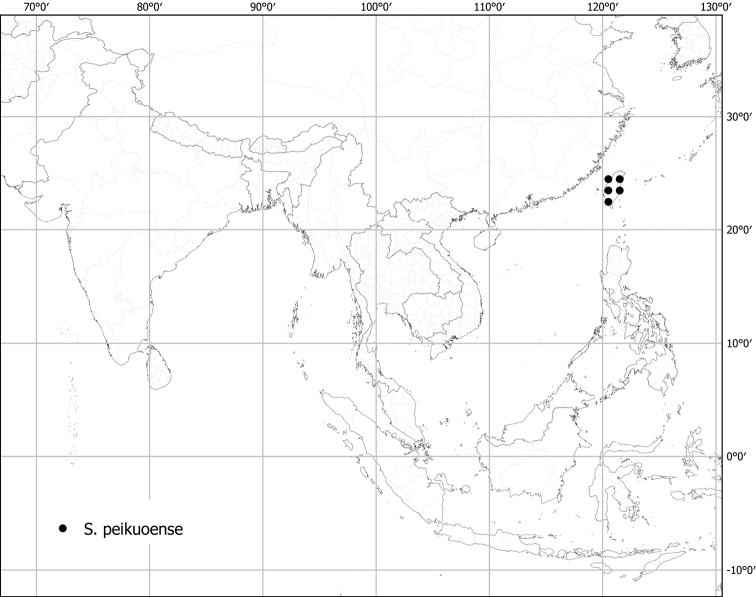
Distribution of *S.peikuoense*.

**(Fig. 50).***Solanumpeikuoense* is endemic to the island of Taiwan, most collections are from the southern part of the island.

#### Ecology and habitat.

*Solanumpeikuoense* grows in broadleaf, coniferous or mixed broadleaf-coniferous forests, often along roadsides or in open areas, from 1,200 to 2,000 m elevation.

#### Common names and uses.

None recorded

#### Preliminary conservation assessment

**(IUCN 2019).** Vulnerable (VU [B1,2a,bi,ii,]. EOO (10,013 km^2^, VU); AOO (44 km^2^, EN). *Solanumpeikuoense* is a single island endemic with a small extent of occurrence. It is found in at least one protected area (Dawushan National Park), so is likely to be protected in the future.

#### Discussion.

*Solanumpeikuoense* is a distinctive plant, and unlikely to be confused with any other species in the region. It is a member of the Torva clade (see Aubriot et al. 2016a) and is morphologically similar to other southeast Asian members of the group such as *S.poka*, *S.pseudosaponaceum* and *S.torvoideum* (and the introduced *S.torvum*). Its shiny, almost glabrous mature leaves and stems distinguish it from any of these species, all of which are densely pubescent. It can be distinguished from the introduced *S.torvum* in the shiny leaves with acute to acuminate (rather than acute to truncate) apices, and its red or black berries (versus dirty green-yellow in *S.torvum*); *S.peikuoense* also lacks the characteristic simple glandular trichomes found in the inflorescence axes of *S.torvum*. Some collections (e.g., *Ku 1752*) mention dark purple stems and purple flowers; other gatherings record the flowers as white.

Although a single gathering (*Ying s.n.*) in a single herbarium (NTUF) was cited in Ying (1997:14), the word type or holotype, or its equivalent was not used. This is contrary to Art. 40.6 of the “Code” (Turland et al 2018) and renders “*Solanumpeikuoensis* S.S.Ying” not validly published. We validate this name here and cite as type material a gathering for the same area that is widely distributed and has both flowers and fruits.

#### Paratypes.

[also see Suppl. materials 1–3] **Taiwan. Chiayi Hsien**: Chiayi, Arisan, Kagi, common around Funkiko, 25 Jan 1918, *Wilson 9657* (A). **Hualien Hsien**: Tungnengkao, Panpien, 20 Apr 1927, *Saito 6965* (A). **Kaohsiung Hsien**: Teng-chu, 22 Jan 1972, *Hsu 13689* (A); Tengchih, 17 Nov 1992, *Huang et al. 15850* (A); Taoyuan Hsiang, along Southern Cross-Island Hwy, between Lanya and Likuan, 27 Jun 2000, *Lin 405* (CAS, HAST); Taoyuan Hsiang, near road mileage sign 13 km on Meilan Forest Road, 10 May 1994, *Liu et al. 427* (HAST); Taoyuan Hsiang, Shihshan Forest Road 9.7 km, 21 May 1997, *Peng 16833* (HAST); Taoyuan Hsiang, Tengchih, 28 Jun 2000, *Wang 4317* (HAST); Kaohsiung Hsien, Taoyuan Hsiang, Tengchih, 29 Jun 2000, *Wang 4376* (HAST, PE); Taoyaun Hsiang, Tengchih, 24 Jul 2004, *Wang et al. 11628* (HAST). **Nantou Hsien**: Hsinyi Hsiang, Shenmu village, 31^st^ forest compartment, 19 Apr 1984, *Peng 6576* (HAST); Jenai Hsiang, 9-10K, 22 Apr 2000, *Wang 5514* (HAST). **Pingtung Hsien**: Wutain Hsiang, Hsiaogueihu forest road, 3 May 2003, *Ku 1752* (HAST). **Taichung Hsien**: Hoping Hsiang, Chia-yang to Tsui-luan, 7 May 1997, *Lin et al. 938* (HAST); Hoping Hsiang, Tahsuehshan Forest Road, 30 Sep 2006, *Lu 12541* (HAST); Tainchung Hsien, Hoping Hsiang, Tahhuehshan 200 forest road 30K, 28 Feb 2001, *Wang & Li 4720* (HAST, PE); Hoping Hsiang, on Hsuehshan Forest Road, at road mileage sign 28.2 km, 5 May 1999, *Wu 1273* (A, HAST); Hoping Hsiang, Pei-ko-ta-shan, 1 Aug 1996, *Ying s.n.* (HAST). **Taitung Hsien**: Haituan Hsiang, Hungshih Forest Road, near road marker 9.5 km, 1 Mar 2000, *Liu 1369* (HAST); Peinan Hsiang, LiChia Forest Road, between road marker 19 km and 22 km, 25 Jul 2008, *Lu 16536* (HAST); Yenping Hsiang, Yenping Forest Road, 24 Dec 2000, *Yang s.n.* (HAST); Chin-shui-ying, 18 Sep 1998, *Yang 23750* (HAST).

### ﻿31. Solanum poka

Taxon classificationPlantaeSolanalesSolanaceae

Dunal, Encycl. [J. Lamarck & al.] Suppl. 3: 768. 1814.

CB048977-3231-53DC-97E4-5C5C4B882C99

Fig. 51

Solanum torvum Sw. var. scabrescens Miq., Fl. Ned. Ind. 2: 648. 1857. Type. Indonesia. Sumatra: Sin. loc., *F.W. Junghuhn s.n.* (lectotype, designated here: L [L.0403917]; isolectotype: U [U0113979]).
Solanum torvum Sw. var. polyacanthum Miq., Fl. Ned. Ind. 2: 648. 1857, as “*polyacantha*”. Type. Indonesia. Java: sin. loc., *T. Horsfield s.n.* (lectotype, designated here: BM [BM000886307]).

#### Type.

Based on an unpublished illustration of a Jean-Baptiste Leschenault de la Tour collection from Java kept in the Node-Véran collection in Montpellier (lectotype, designated by Aubriot et al. 2016b, pg. 105: [illustration] Sol. Tab. 55 [MPU028527]).

#### Description.

Shrubs to 3 m, armed. Stems erect, terete, usually densely prickly distally, moderately pubescent, glabrescent; prickles to 3.5 mm long, to 2.5 mm wide at the base, straight, awl-shaped to deltate, conical, pale yellow, glabrescent; pubescence of mixed sessile and variously stalked porrect-stellate trichomes, the stalks to 0.2 mm long, the rays (4–)5–8, 0.1–0.25 mm long, the midpoints reduced to mere bumps; new growth moderately stellate-pubescent, black to dark brownish in dry material; bark of older stems brownish grey, sparsely stellate-pubescent. Sympodial units difoliate, the leaves geminate. Leaves simple, entire to deeply lobed, the blades 11–24 cm long, 4–13 cm wide, ca. 1.5–3 times longer than wide, elliptic to broadly ovate, chartaceous, slightly discolorous, unarmed or pricky with 1–6 prickles per leaf side, the prickles to 6 mm long, to 1.5 mm wide at the base, straight or slightly curved at the tip, awl-shaped, conical, pale yellow, glabrous; adaxial surface moderately stellate-pubescent with porrect, sessile and less often variously stalked trichomes, the stalks to 0.1 mm long, the rays 4–8, 0.1–0.4 mm long, the midpoints to 0.25 mm long; abaxial surface moderately stellate-pubescent with trichomes like those of the adaxial surface, but more often stalked; major veins 6–8 pairs drying yellow; base short-attenuate to truncate; margins entire or shallowly to deeply lobed, the lobes 1–5 on each side, 0.5–5 cm long, deltate, rounded to apically acute, the sinuses extending up to 2/3 of the distance to the midvein; apex acute; petiole 1.5–4 cm long, 1/10–1/5 of the leaf blade length, densely stellate-pubescent with porrect, sessile trichomes like those of the blades, unarmed or prickly with 1–5 prickles like those of the stems. Inflorescences 2–5 cm long, apparently lateral or leaf opposed, unbranched to up to 2 times branched, with ca. 5–20 flowers, 2–6 flowers open at any one time, moderately to densely stellate-pubescent with porrect-stellate trichomes like those of the stems, unarmed; peduncle 0.5–1.5 cm long, unarmed or with very few prickles; pedicels 0.5–1.2 cm long, ca. 1 mm in diameter at the base, ca. 1.5 mm in diameter at the apex, erect, unarmed, densely stellate-pubescent with porrect-stellate trichomes like those of the inflorescence axes, articulated at the base; pedicel scars spaced 2–4 mm apart. Flowers 5-merous, apparently all perfect. Calyx with the tube 1–3 mm long, conical, the lobes 3–5 mm long, ca. 1.5 mm wide, the lower part deltate and abruptly constricting to an elongate acumen, the acumen 3/4 of the total lobe length, the abaxial surface more or less strongly keeled along the midvein, unarmed, densely stellate-pubescent on the midvein with porrect-stellate trichomes like those of the pedicels. Corolla 1–2 cm in diameter, white, stellate, lobed ca. 1/2–2/3 of the way to the base, the lobes 5–8 mm long, 2–3.5 mm wide, deltate, spreading at anthesis, densely stellate-pubescent abaxially on parts exposed in bud. Stamens equal; anthers 5–6.5 mm long, ca. 0.75 mm wide, tapering, yellow, connivent, glabrous, poricidal at the tips, the pores not lengthening to slits with age; filament tube <0.5 mm long, glabrous; free portion of the filaments 0.75–1.5 mm long, glabrous. Ovary conical, minutely glandular-puberulent; style 0.6–1 cm long, slender, curved at the apex, with a few scattered hairs at the tip; stigma capitate, minutely papillate, stellate-pubescent. Fruit a globose berry, 8–18 per infructescence, 0.8–1.5 cm in diameter, the pericarp smooth, bluish green when young, turning to dark greyish yellow, glabrous; fruiting pedicels 1.2–2.5 cm long, ca. 1–1.5 mm in diameter at the base, 2–3 mm in diameter at the apex, woody, erect, unarmed; fruiting calyx lobes not expanding. Seeds 100–200 per berry, 1.75–2 mm long, 1.5–1.75 mm wide, flattened reniform, pale yellowish, the surface minutely pitted, the testal cells sinuate in outline. Chromosome number: not known.

**Figure 51.**
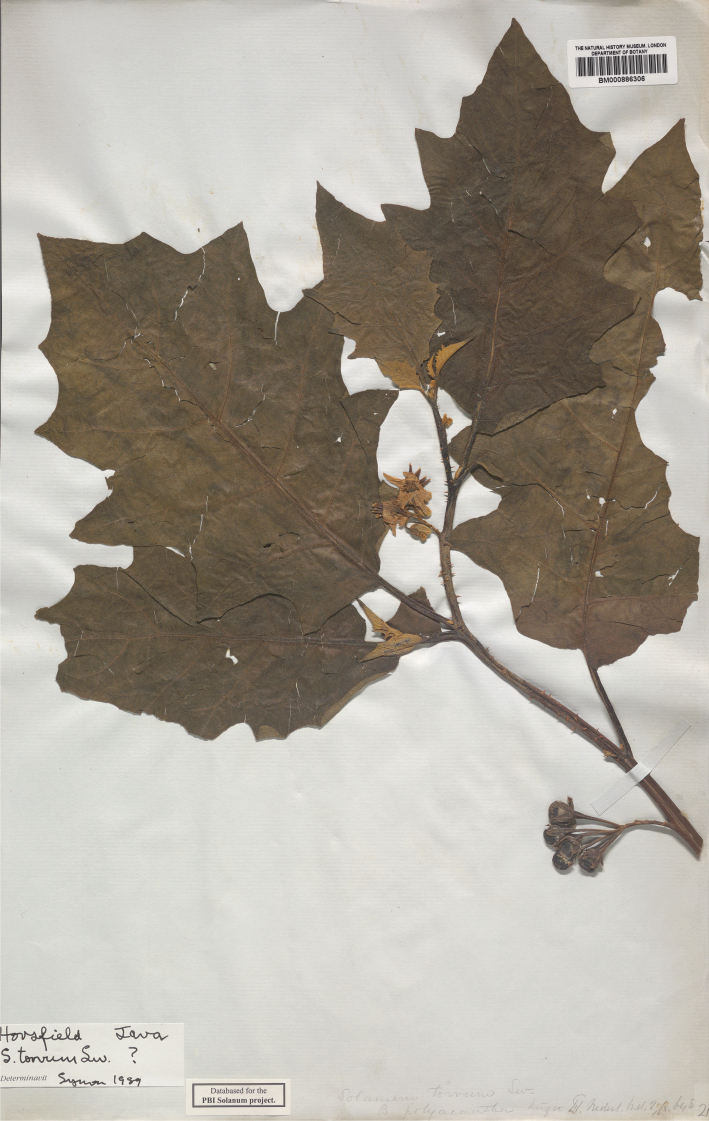
*Solanumpoka* Dunal – Herbarium specimen (epitype) collected in Indonesia (*Horsfield s.n.*, BM000886306). Photograph credit: CC-BY, © copyright The Trustees of the Natural History Museum, London.

**Figure 52.**
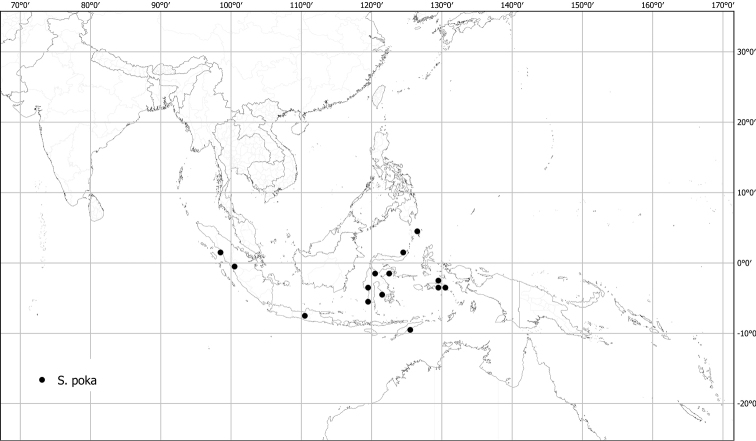
Distribution of *S.poka*.

#### Distribution

**(Fig. 52).***Solanumpoka* is widely distributed in the Malay Archipelago, from western Sumatra to the Maluku Islands and across Sulawesi, northwards to the Talaud islands.

#### Ecology and habitat.

*Solanumpoka* is a plant of evergreen broadleaf forests, growing in open woodland, forest edges, degraded vegetation, usually on limestone or volcanic rocks; from sea level to 1,600 m elevation.

#### Common names and uses.

Indonesia. Java: pooka (*Horsfield 786*), daun poka (Dunal 1814); North Sulawesi: poki poki (*De La Savinierre 343bis*), sangkirumanuwawi (*Lam 2772*).

#### Preliminary conservation status

**(IUCN 2019).** Least Concern (LC). EOO (1,202,780 km^2^, LC); AOO (68 km^2^, EN). Although the EOO measurement indicates a status of least concern, the few collections coupled with the profound transformation in lowland Indonesian habitats where *S.poka* is found (Margono et al. 2014) suggest that the species is a priority for recollection and reassessment.

#### Discussion.

*Solanumpoka* is a member of the Torva clade (Aubriot et al. 2016a) and is similar to other members of the group occurring in southeast Asia such as *S.pseudosaponaceum* and *S.torvoideum*. Morphologically, *S.poka* most closely resembles *S.pseudosaponaceum*, a widespread species ranging from Taiwan and southern China to Indonesia but differs in having denser indumentum on the adaxial leaf surface, more numerous straight prickles on the upper stems, fewer, larger flowers with elongate strongly keeled calyx lobes, and much larger fruits. Flowers of *S.pseudosaponaceum* are lilac or purplish-white while those of *S.poka* are always described on labels as white. *Solanumpoka* can be distinguished from the introduced *S.torvum* in its strongly keeled calyx lobes with elongate acumens (rather than deltate lobes), red (rather than green) berries, and in the lack of simple glandular trichomes in the inflorescence.

Aubriot et al. (2016b) recognised S.torvumvar.scabrescens as a synonym in their resurrection of the name *S.poka* but erroneously referred to the Leiden duplicate (L.0403917) of the *Junghuhn s.n.* collection cited in the protologue as “holotype”; Miquel (1857) cited no herbaria in the protologue. They did not include Miquel’s var. polyacanthum in the synonymy of *S.poka*, but the minimal description with reference to pricklier stems and leaves approaching *S.giganteum*, however, suggest this varietal name refers to the taxon we recognise as *S.poka*. The protologue states no specific locality, merely “β. Java (Horsfield)”. We have therefore selected the specimen of *S.poka* from Java collected by Thomas Horsfield that best matches the protologue at BM (BM000886307) as the lectotype for S.torvumvar.polycanthum.

#### Specimens examined.

See Suppl. materials 1–3.

### ﻿32. Solanum praetermissum

Taxon classificationPlantaeSolanalesSolanaceae

Kerr ex Barnett, Kew Bull. 16: 485. 1963.

76B4BAE8-CE24-5BEF-BE41-CE0D7364F3ED

Figs 4G , 53

Solanum barbisetum Nees var. griffithii Prain, J. Asiat. Soc. Beng. 65(2): 541. 1896. Type. India. [Arunachal Pradesh?]: “Upper Assam”, 1841, *F. Jenkins 253* (lectotype, designated by Hul and Dy Phon 2014, pg. 36: K [K000449043]; isolectotype: K [K000449042]).
Solanum griffithii (Prain) C.Y.Wu & S.C.Huang, Acta Phytotax. Sin. 16(2): 75. 1978, non S.griffithii (C.B.Clarke) Kuntze, 1891. Type. Later homonym, based on SolanumbarbisetumNeesvar.griffithii Prain.
Solanum neogriffithii V.V.Hop, J. Biol. Vietnam 27(3): 13. 2005. Type. Based on SolanumbarbisetumNeesvar.griffithii Prain.
Solanum membranisepalum Li Bing Zhang & Ngan T.Lu, Phytotaxa 186(4): 239. 2014. Type. Based on SolanumbarbisetumNeesvar.griffithii Prain.
Solanum brevipedunculatum Li Bing Zhang & Yi F.Duan, Phytotaxa 170(4): 280. 2014, non S.brevipedunculatum Rusby, 1907. Type. Based on SolanumbarbisetumNeesvar.griffithii Prain.

#### Type.

Thailand. Tak: Raheng [Rahaeng], *E. Smith s.n.* (holotype: K [K000614048]; isotypes: BK [BK229395], BM [BM000886099]).

#### Description.

Herbs to small shrubs, to 0.5 m tall, armed. Stems erect, terete, moderately to sparsely prickly and densely stellate-pubescent; prickles to 4 mm long, to 2 mm at the base, straight, narrowly deltate, pale yellow, glabrous; trichomes porrect-stellate, mixture of sessile and stalked, the stalks to 0.25 mm long, the rays 4–8, 0.1–0.4 mm long, the midpoints up to 0.1 mm long; new growth densely stellate-pubescent, whitish green in dry material; bark of older stems light brownish, moderately to densely stellate pubescent. Sympodial units difoliate, the leaves geminate, the leaves of a pair differing somewhat in size but not in shape. Leaves simple, entire to shallowly lobed, the blades 9–17 cm long, 6.5–13 cm wide, ca. 1–1.5 times longer than wide, ovate to broadly ovate, chartaceous, discolorous, moderately prickly with 4–16(–24) prickles per leaf side, usually longer than those of the stems, up to 7 mm long, often purplish black in living plants; adaxial surface dark green, moderately to densely stellate-pubescent, the stellate trichomes porrect, sessile or stalked, the stalks to 0.1 mm long, the rays 4–7, 0.1–0.4 mm long, the midpoints up to 0.5 mm long; abaxial surface light green, densely stellate-pubescent with trichomes like those of the adaxial surface but more long-stalked; major veins 4–5 pairs drying whitish brown; base attenuate to truncate; margins sinuate to shallowly lobed, the lobes 3–4 on each side, 0.5–2 cm long, broadly deltate to obovate, apically rounded to acute, the sinuses extending up to 1/3 of the distance to the midvein; apex acute; petiole 2.5–7 cm long, 1/4–1/3 of the leaf blade length, densely stellate-pubescent with porrect sessile trichomes like those of the stems, unarmed or with 1–8 prickles like those of the blades. Inflorescences 1.5–3 cm long, apparently lateral, unbranched, with ca. 5–11 flowers, 1–2 flowers open at any one time; axes densely stellate-pubescent with trichomes like those of the stems, but sometimes purplish tinged in living plants, unarmed or with prickles like those of the stems but smaller, often purplish black in live plants; peduncle 0.2–1 cm long, with 0–4 prickles; pedicels 0.4–1 cm long, ca. 0.75 mm in diameter at the base, ca. 1.5 mm in diameter at the apex, erect to recurved, unarmed or with 1–2 small prickles, densely stellate-pubescent with trichomes like those of the inflorescence axes, articulated at the base; pedicel scars spaced 1–3 mm apart. Buds oval to ellipsoid, more or less exserted from the calyx before anthesis. Flowers 5-merous, apparently all perfect. Calyx with the tube 2–3 mm long, conical, the lobes 2–3.5 mm long, 0.75–1.5 mm wide, narrowly deltate, apically acute, unarmed and densely stellate-pubescent abaxially with trichomes like those of the pedicels. Corolla 0.7–1.5 cm in diameter, white, sometimes pale lilac, stellate, lobed ca. 2/3 of the way to the base, the lobes 4–5.5 mm long, 1.5–3 mm wide, narrowly deltate to deltate, spreading at anthesis, glabrous adaxially, densely stellate pubescent abaxially on parts exposed in bud. Stamens equal; anthers 4–5 mm long, ca. 1 mm wide, not markedly connivent, tapering, yellow, glabrous, poricidal at the tips, the pores directed distally, not elongating to slits with drying; filament tube <1 mm long; free portion of the filaments 0.5–1 mm long, glabrous. Ovary conical, minutely glandular-puberulent at the top; style ca. 6 mm long, slender, slightly recurved at the apex, glabrous; stigma capitate, minutely papillate. Fruit a globose berry, 4–8 per infructescence, 0.8–1.2 cm in diameter, whitish cream at maturity, the pericarp smooth, glabrous; fruiting pedicels 0.9–1.2 cm long, ca. 1 mm in diameter at the base, 2–3.5 mm in diameter at the apex, woody, erect to recurved, unarmed or with 1–10 prickles; fruiting calyx accrescent, completely covering the fruit at maturity, the tube enclosing the ripe berry, prickly and stellate-pubescent, the lobes expanding to 1 cm long, with up to 20 prickles per lobe, these often purplish black in living plants. Seeds 20–50 per berry, 2–3 mm long, 1.5–2 mm wide, flattened-reniform, light brownish, the surface minutely pitted, the testal cells somewhat sinuate in outline. Chromosome number: not known.

**Figure 53.**
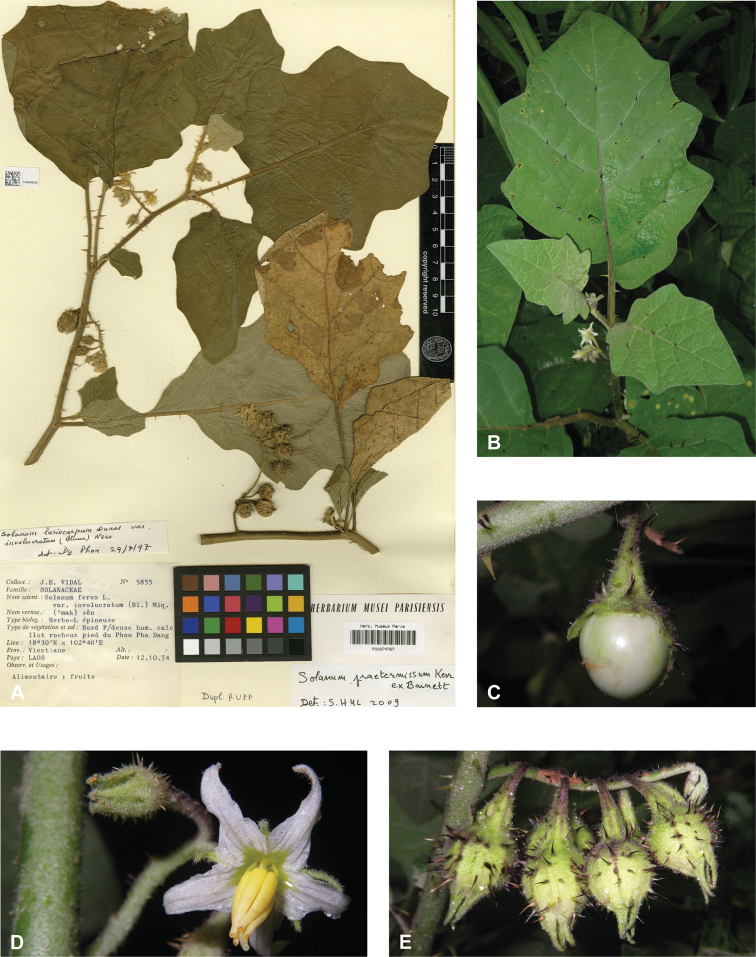
*Solanumpraetermissimum* Kerr ex Barnett **A** herbarium specimen collected in Laos in 1974 (*Vidal 5855*, P00074767) **B** habit (field photograph, unvouchered, Vietnam) **C** detail view of a fruit with the calyx removed (field photograph, unvouchered, Vietnam) **D** detail view a flower (field photograph, unvouchered, Vietnam) **E** detail view an infructescence (field photograph, unvouchered, Vietnam). Photograph credits: **A** CC-BY, Muséum national d’Histoire naturelle, Paris **B–E** M. Nuraliev.

#### Distribution

**Figure 54.**
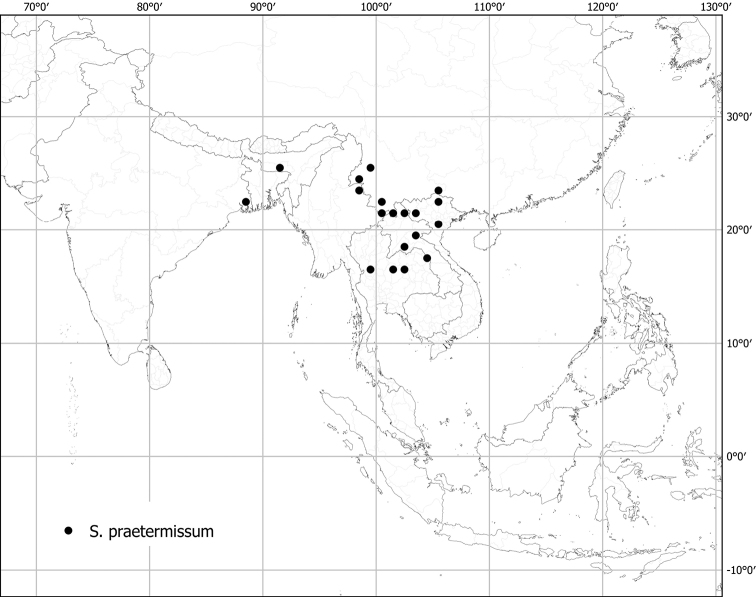
Distribution of *S.praetermissum*.

**(Fig. 54).***Solanumpraetermissum* occurs from northeastern India to South China and Vietnam.

#### Ecology and habitat.

*Solanumpraetermissum* is a plant of evergreen broadleaf forests or semi-deciduous forests, growing in forest understory or in clearings of secondary woodlands, or on rocky banks; from 300 to 400 m elevation.

#### Common names and uses.

Vietnam. co chu (Muong, Hul and Dy Phon 2014).

#### Preliminary conservation status

**(IUCN 2019).** Least Concern (LC). EOO (1,191,003 km^2^, LC); AOO (68 km^2^, EN). *Solanumpraetermissum* is widely distributed, but where we have seen it in the field, is in small populations, perhaps indicating further assessment is necessary.

#### Discussion.

*Solanumpraetermissum* is most similar to *S.barbisetum*, with which it is sympatric and has previously been confused. It was first described as a variety of the latter (as var. griffithii) and once it was recognised as distinct several attempts were made to assign a name at the specific level (see synonymy); none of these authors apparently realised the name *S.praetermissum* was already published and thus had priority. Both species are small shrubs and have small, whitish cream berries enclosed in prickly accrescent calyces. *Solanumpraetermissum* can be distinguished from *S.barbisetum* in its pubescence of largely sessile stellate trichomes (versus long-stalked stellate trichomes), lack of bristles on the inflorescence (versus densely bristly inflorescences and calyces, especially in fruit), generally shorter inflorescence axes (1.5–3 cm versus 3–10 cm long, although some young inflorescences can be shorter), and smaller flowers (0.7–1.5 cm versus 1.8–2.2 cm in diameter).

Mill (2001) treated *S.praetermissum* for Bhutan under the synonym *S.griffithii*, with the suggestion that they were doubtfully distinct. He cited no specimens for Bhutan for either species.

Aubriot et al. (2016a) resolved *S.praetermissum* as sister to the southern Indian endemic *S.wightii*, but with very low support; in the same study, *S.barbisetum* was resolved as sister to the ‘S.expedunculatum and relatives’ lineage albeit with poor support. *Solanumwightii* also has a strongly accrescent calyx but is otherwise morphologically very different from *S.praetermissum* (see description below).

#### Specimens examined.

See Suppl. materials 1–3.

### ﻿33. Solanum procumbens

Taxon classificationPlantaeSolanalesSolanaceae

Lour., Fl. Cochinch. 132. 1790.

35DE2AB0-E5B3-556D-B228-6075A6001137

Fig. 55

Solanum hainanense Hance, J. Bot. 6: 331. 1868. Type. China. Hainan: “At vias prope Kieng chau fú, metropolis ins: Hainan”, Nov 1866, *Sampson & H.F. Hance s.n.* [13816] (lectotype, designated here: BM [BM000942492]; isolectotype: K [K000759385]).
Solanum scopulorum Kerr ex Barnett, Kew Bull. 16: 486. 1963. Type. Thailand. Prachuap Khiri Khan: Hua Hin, 11 Nov 1928, *A.F.G. Kerr 16210* (holotype: K [K000922039]; isotypes BK [BK257532], BM [BM000886111]).

#### Type.

Vietnam. Thua Thiên-Huê: Huê and vicinity, Jan 1923, *R.W. Squires 27* (neotype, designated by Hul and Dy Phon 2014, pg. 20: P [P00054100]; isoneotypes: BM [BM000886103], E [E00224906], K [K000195689]).

#### Description.

Scandent or creeping herbs or shrubs, to 2(–3) m tall, armed. Stems prostate or erect, terete, prickly and pubescent; prickles up to 1 cm long, to 1 cm at the base, more or less regularly spaced, strongly hooked but sometimes straight, flattened and deltate, pale yellow, glabrous; pubescence of sessile porrect-stellate trichomes, the rays 6–8, 0.1–0.3 mm long, the midpoints absent or <0.1 mm long; new growth sparsely to densely stellate-pubescent, green to light brownish in dry material; bark of older stems brownish grey, glabrous. Sympodial units apparently plurifoliate, the leaves not geminate. Leaves simple, entire to moderately lobed, the blades 2–6.5 cm long, 1–3.5 cm wide, ca. 1.5–2.5 times longer than wide, elliptic to ovate, sometimes obovate, chartaceous, discolorous, unarmed or with up to 5 prickles per face, these slightly smaller and thinner than the ones on the stems; adaxial surface green to dark green, evenly and sparsely pubescent with erect sessile porrect-stellate trichomes, the rays 6–8, 0.1–0.3 mm long, the midpoints to 0.1 mm; abaxial surface densely stellate pubescent with trichomes like those of the adaxial surface but with stalks up to 0.1 mm long and longer rays; major veins ca. 3 pairs drying light brownish; base attenuate; margins entire, shallowly to moderately lobed, the lobes 1–3 on each side, 0.4–1 cm long, oblong to deltate, apically rounded, the sinuses extending up to 1/3–1/2 of the distance to the midvein; apex acute to rounded; petioles 0.5–1 cm long, 1/10–1/4 of the leaf blade length, unarmed or sparsely prickly with a few curved prickles, densely stellate-pubescent, the pubescence of sessile porrect-stellate trichomes like those of the blades. Inflorescences 1.5–4 cm long, lateral or occasionally leaf-opposed, unbranched, with 4–8 flowers, 1–3 flowers open at any one time, unarmed or sparsely prickly, densely pubescent with sessile stellate-porrect trichomes like those of the stems; peduncle 0.1–1.4 cm long; pedicels 0.5–2 cm long, ca. 0.5 mm in diameter at the base and 0.5–0.75 mm in diameter at top, erect or somewhat recurved at anthesis, densely stellate-pubescent like the inflorescence axes, articulated at the base; pedicel scars irregularly spaced 0.1–2 mm apart. Buds ovoid, exserted from the calyx before anthesis. Flowers 4(–5)-merous, apparently all perfect. Calyx with the tube 1–1.5 mm long, campanulate, the lobes 1–1.5 mm long, ca. 1 mm wide, deltate to broadly deltate, apically acute to acuminate, unarmed, densely stellate-pubescent with porrect-stellate trichomes like those of the pedicels. Corolla 1–1.5 cm in diameter, white or pale purple to deep blue, stellate, lobed ca. 3/4 of the way to the base, the lobes 5–7 mm long, 1–3 mm wide, long-triangular, spreading or reflexed at anthesis, mostly glabrous adaxially but with a few stellate trichomes on the middle vein, densely stellate pubescent abaxially on parts exposed in bud. Stamens equal or slightly unequal with 2 slightly longer than the rest; anthers 5–5.5 mm long, 1–1.5 mm wide, not connivent to somewhat spreading, all tapering, dull yellow, glabrous, poricidal at the tips, the pores not elongating to slits with drying; filament tube minute, glabrous; free portion of the filaments ca. 0.5 mm long, glabrous. Ovary conical to globular, minutely glandular-puberulent at the top; style 6–8 mm long, with few stellate trichomes scattered at the base; stigma clavate, the surfaces minutely papillose. Fruit a globose berry, several per infructescence, 0.6–1 cm in diameter, red at maturity, pericarp thin, glabrous; fruiting pedicels 1.3–1.8 cm long, ca. 1 mm in diameter at the base, 1.5–1.8 mm in diameter at the apex, erect or slightly recurved, somewhat woody, unarmed; fruiting calyx lobes ca. 2 mm long, not markedly accrescent, but covering 1/4 of the berry and not reflexed, unarmed. Seeds 5–25 per berry, 2–3 mm long, 2–3 mm wide, flattened reniform to rounded, brownish orange, the surfaces minutely pitted, the testal cells pentagonal in outline. Chromosome number: not known.

**Figure 55.**
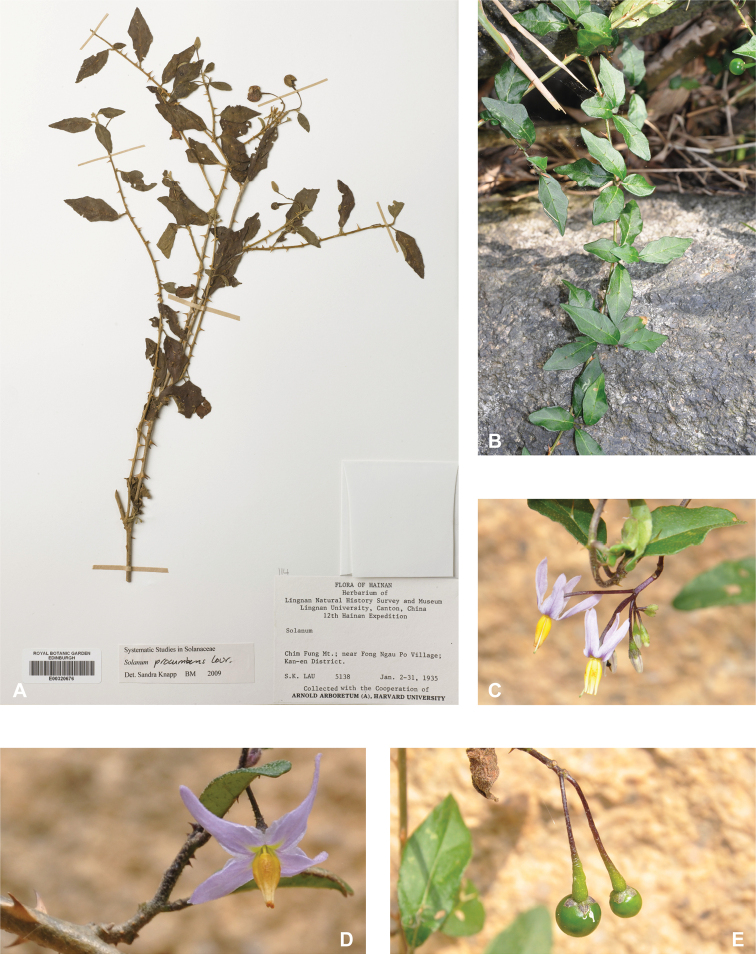
*Solanumprocumbens* Lour. **A** herbarium specimen collected in China in 1935 (*Lau 5138*, E00320676) **B** habit (*Wang et al. 2075*, China) **C** inflorescence (*Wang et al. 2075*, China) **D** detailed view of a flower and prickles (*Wang et al. 2075*, China) **E** infructescence (*Wang et al. 2075*, China). Photograph credits: **A** Royal Botanic Garden Edinburgh **B–E** S. Knapp.

#### Distribution

**Figure 56.**
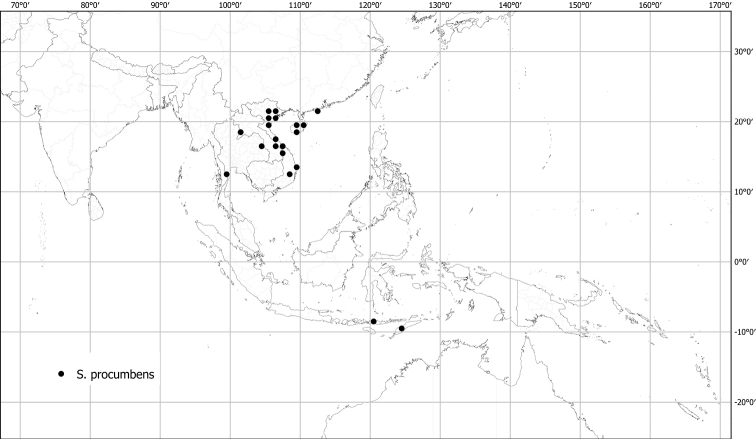
Distribution of *S.procumbens*.

**(Fig. 56).***Solanumprocumbens* is widely distributed from China and Indochina to Indonesia (Flores and Timor) and Timor Leste.

#### Ecology and habitat.

*Solanumprocumbens* is a plant of open places in many forest types, often scrambling over other vegetation and on rocks, from sea level to 650(1,500) m elevation.

#### Common names and uses.

China. hai nan qie (Zhang et al. 1994). Vietnam. cà gai, (cây) cur’o’ng, trông ca dap (Hul and Dy Phon 2014)

#### Preliminary conservation status

**(IUCN 2019).** Least Concern (LC). EOO (1,575,888 km^2^, LC); AOO (164 km^2^, EN). *Solanumprocumbens* is widely distributed and occurs in many habitat types, including extremely anthropogenically disturbed ones.

#### Discussion.

*Solanumprocumbens* is similar to *S.trilobatum* and *S.camranhense* in its habit as a small scrambling shrub, but in molecular analyses of Aubriot et al. (2016a) resolves as closely related to *S.involucratum*, a much more robust and morphologically very different plant. *Solanumprocumbens* can be distinguished from *S.camranhense* in its usually 4-parted corollas, slender fruiting pedicels, and more lanceolate leaves with less truncate bases.

No specimens were cited in the protologue (Hance 1868), so the lectotype we have selected for *S.hainanense* (BM00942492) is the better preserved of the two duplicates we have found and bears the Hance exsiccata number 13816.

#### Specimens examined.

See Suppl. materials 1–3.

### ﻿34. Solanum pseudosaponaceum

Taxon classificationPlantaeSolanalesSolanaceae

Blume, Bijdr. Fl. Ned. Ind. 13: 702. 1826.

17512BE4-2204-5781-B1A4-58DE8B1653AC

Figs 3F , 57

Solanum inaequilaterale Merr., Philipp. J. Sci. 1, Suppl. 236. 1906. Type. Philippines. CAR: Luzon, Benguet Province, Suyse to Panai, Oct 1905, *E.D. Merrill 4807* (lectotype, designated here: US [00027616, acc. # 710478]; isolectotypes: K [K000195913], NY [00172282]).
Solanum torvum Sw. var. lasiostylum Y.C.Liu & C.H.Ou, Quart. J. Chinese Forest. 7(4): 151. 1974. Type. Taiwan. Sin. loc., “In southern parts at low altitudes”, 1 Apr 1971, *Y.C. Liu & C.H. Ou 540* (holotype: TCF).
Solanum lasiostylum (Y.C.Liu & C.H.Ou) Tawada, J. Phytogeogr. Taxon. 27(1): 36. 1979. Type. Based on SolanumtorvumSw.var.lasiostylum Y.C.Liu & C.H.Ou.

#### Type.

Indonesia. Java: Sin. loc., *C.L. Blume s.n.* (lectotype, designated here: GZU [GZU000255455]; isolectotypes: L [L0003669, L0003670]).

#### Description.

Erect shrub to small tree to 8 m tall, armed. Stems erect, terete, prickly, usually densely stellate-pubescent; prickles to 5 mm long, to 5 mm wide at the base, straight to slightly curved, deltate, laterally flattened, pale yellow, glabrescent; pubescence of mixed sessile and variously stalked porrect-stellate trichomes, the stalks to 0.25 mm long, the rays 5–8, 0.25–0.5 mm long, the midpoints absent or up to 0.25 mm long; new growth densely stellate-pubescent with a whitish pubescence, bark black; bark of older stems brownish grey to black, glabrescent. Sympodial units difoliate to plurifoliate, the leaves not geminate. Leaves simple, entire to deeply lobed, the blades 8–17 cm long, 3.5–10.5 cm wide, ca. 1.5–3 times longer than wide, elliptic to ovate, chartaceous, strongly discolorous, unarmed or prickly with 1–6 prickles per leaf side, to 6 mm long, to 1.5 mm wide at the base, straight, awl-shaped, conical, pale yellow, glabrous; adaxial surface dark green, usually sparsely stellate-pubescent to glabrescent, sometimes moderately stellate-pubescent (e.g., *Elmer 12879*), the stellate trichomes porrect, sessile or stalked, the stalks to 0.25 mm long, the rays 4–9, 0.25–0.75 mm long, the midpoints to 0.5 mm long; abaxial surface light yellowish green, densely stellate-pubescent with trichomes like those of the adaxial surface, but more often stalked; major veins 5–7 pairs drying yellow; base short-attenuate to truncate; margins entire or shallowly to deeply lobed, the lobes 1–5 on each side, 0.5–4.5 cm long, deltate, rounded to apically acute, the sinuses extending up to 2/3 of the distance to the midvein; apex acute; petiole 1.5–4 cm long, 1/5–1/3 of the leaf blade length, moderately to densely stellate-pubescent with porrect, sessile trichomes like those of the blades, unarmed or prickly with 1–3 prickles like those of the stems. Inflorescences 3–5.5 cm long, apparently lateral, forked to 3 times branched, with ca. 10–40 flowers, 1–8 flowers open at any one time, densely stellate-pubescent with porrect trichomes like those of the stems, unarmed; peduncle 0.5–1.5 cm long, unarmed; pedicels 0.7–0.8 cm long, ca. 0.75 mm in diameter at the base, ca. 0.75 mm in diameter at the apex, erect, unarmed, densely stellate-pubescent with porrect trichomes like those of the inflorescence axes, articulated at the base; pedicel scars spaced 0.5–2 mm apart. Buds ovoid to ellipsoid, strongly exserted from the calyx before anthesis. Flowers 5-merous, apparently all perfect. Calyx with the tube 0.5–0.75 mm long, campanulate, the lobes 1–1.5 mm long, 0.5–0.75 mm wide, deltate, apically acute to acuminate, unarmed and densely stellate-pubescent abaxially with trichomes like those of the pedicels. Corolla 1–1.5 cm in diameter, purple, stellate, lobed ca. 1/2–3/4 of the way to the base, the lobes 4–7 mm long, 2–4 mm wide, deltate, spreading at anthesis, adaxially glabrous but with a few stellate trichomes at the lobe tips, densely stellate-pubescent abaxially on parts exposed in bud. Stamens equal; anthers ca. 5 mm long, ca. 1 mm wide, tapering, yellow, connivent, glabrous, poricidal at the tips, the pores not lengthening to slits with age; filament tube ca. 1 mm long, glabrous; free portion of the filaments 0.5 mm long, glabrous. Ovary globose, with minute glandular hairs and few stellate hairs in the lower half; style 7–8 mm long, slender, curved at the apex, densely stellate pubescent in the lower half; stigma capitate, minutely papillate. Fruit a globose berry, several to many per infructescence, 0.7–1 cm in diameter, the pericarp smooth, thin, red when mature, glabrous; fruiting pedicels 1–1.5 cm long, 1 mm in diameter at the base, ca. 1.5 mm in diameter at the apex, woody, erect, unarmed; fruiting calyx lobes expanding to 2.5 mm long, 1/3 the length of the mature fruit, broadly deltate, reflexed, unarmed. Seeds 25- –more than 100 per berry, 2–3 mm long, 1.5–2 mm wide, flattened-reniform, dull yellow, the surface minutely pitted, the testal cells sinuate in outline. Chromosome number: not known.

**Figure 57.**
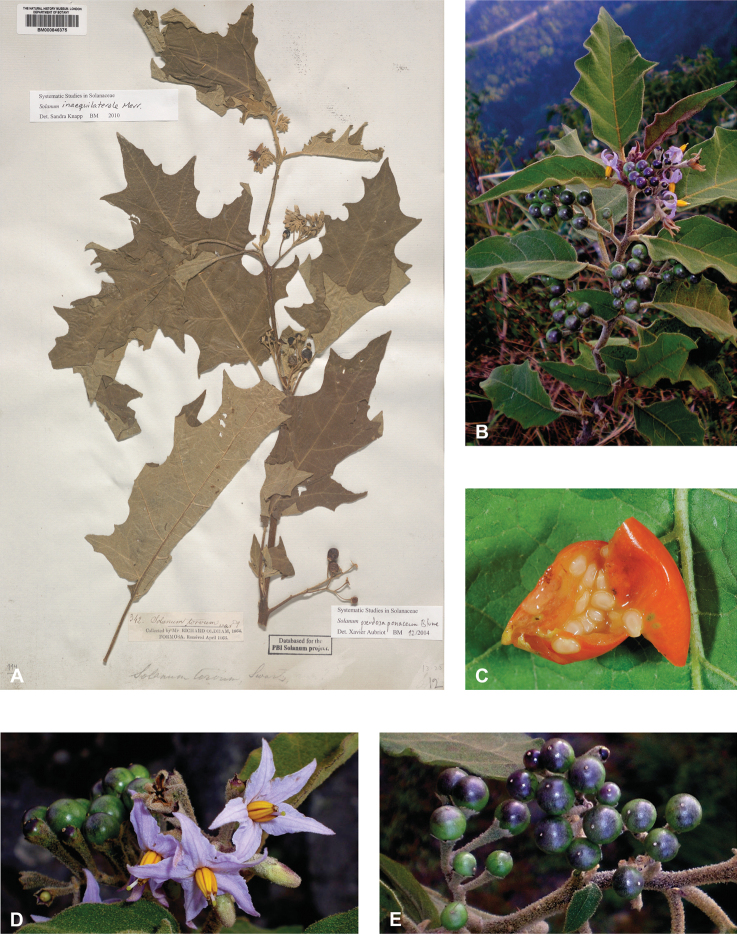
*Solanumpseudosaponaceum* Blume **A** herbarium specimen collected in Taiwan in 1864 (*Oldham 342*, BM000846375) **B** fertile stem (field photograph, unvouchered, Philippines) **C** open fruit with seeds inside (field photograph, unvouchered, Taiwan) **D** inflorescence (field photograph, unvouchered, Philippines) **E** infructescence (field photograph, unvouchered, Philippines). Photograph credits: **A** CC-BY, © copyright The Trustees of the Natural History Museum, London **B, D, E** D. Tandang **C** M.-I. Weng.

#### Distribution

**Figure 58.**
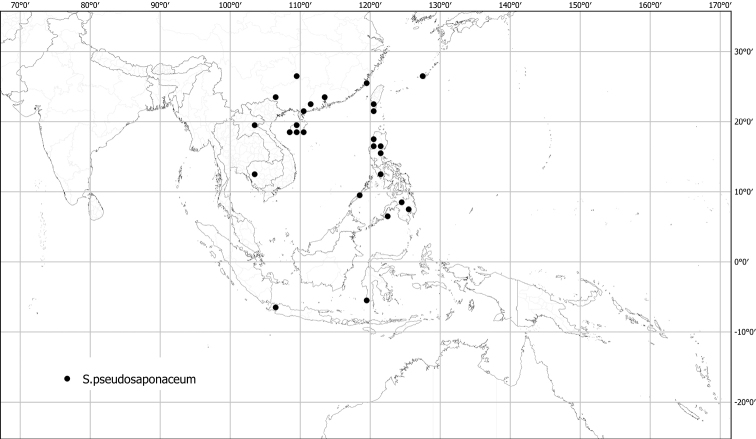
Distribution of *S.pseudosaponaceum*.

**(Fig. 58).***Solanumpseudosaponaceum* is widely distributed from South China (Fujian, Guangdong, Hainan provinces), Taiwan and Ryukyu Islands of southern Japan to the Philippines, Indonesia (Sulawesi, Java) and Indochina (Cambodia, Laos).

#### Ecology and habitat.

*Solanumpseudosaponaceum* is found in pristine or secondary rainforests as well as in open wasteland, usually growing on sand or limestone; from 20 to 2,450 m elevation.

#### Common names and uses.

China. shan qie (Zhang et al. 1994); Guangxi: tain tin ke tsz shue (*Tsang 23902*); Hainan: dithoulat (Lois, *Lau 157*), shan ke (*Tsang 874*). Indonesia. Java: tokokka, magay (Blume 1826). Philippines: qumit (Ifugao, *Conklin 217*). Taiwan: mao zhu wan tao hua (‘twigs are fluffy, many flowered, pink like peach flowers’)

#### Preliminary conservation status

**(IUCN 2019).** Least Concern (LC). EOO (2,387,194 km^2^, LC); AOO (160 km^2^, EN). *Solanumpseudosaponaceum* is widely distributed across tropical Asia, and where it occurs apparently grows in large populations at forest edges and other disturbed habitats.

#### Discussion.

This species was treated as *S.macaoense* Dunal in the “Flora of China” (Zhang et al. 1994), however, the type specimen of *S.macaoense* (*Callery 28/29*, P00055495) is clearly conspecific with *S.torvum*, making *S.pseudosaponaceum* the oldest and correct name for this taxon. *Solanumpseudosaponaceum* is one of the few Torva clade species occurring outside of the Americas (Aubriot et al. 2016a). It shares with the American members of the clade a shrubby habit, usually branched inflorescences, and hermaphroditic flowers. In common with *S.poka*, *S.peikuoense* and *S.torvoideum*, it has red berries; members of this clade native to the Americas have green to yellowish green mature fruits (e.g., *S.chrysotrichum*, *S.torvum*). For characters useful in distinguishing *S.pseudosaponaceum* from other members of the Torvum clade see descriptions of *S.poka* and *S.torvum*.

The specimen (*Martin 1383*) treated as *S.asperolanatum* Ruiz & Pav. in Hul and Dy Phon (2014) is a juvenile plant of *S.pseudosaponaceum*; in the absence of any inflorescences, it is difficult to place, but the pubescence and prickles accord better with it being identified as *S.pseudosaponaceum* than *S.chrysotrichum* (another determination on the sheet at P). *Solanumpseudosaponaceum* is only known from a few collections in Indochina. *Solanumasperolanatum* is an Andean endemic and does not occur in tropical Asia.

The disjunct distribution of the Torva clade is most likely due to long-distance dispersal (Dupin et al. 2016; Echeverría-Londoño et al. 2020), but from a single event, all species included in phylogenetic analyses (Aubriot et al. 2016a) are each other’s closest relatives.

The specimen in Nees van Esenbeck’s herbarium (Stafleu and Cowan 1981) at GZU (GZU0005524455) is annotated “Blume leg.” and has an original label with the common name “Tokkoka” cited in the protologue. We have selected this as the lectotype for *S.pseudosaponaceum* because it is clearly from Blume’s herbarium and has additional information from the protologue on it. The two collections in L here recognised as isolectotypes bear minimal labelling, and none of them has any locality or common name information; one of them (L0003669) has been previously annotated as “lectotype” by an unknown user or by L herbarium staff.

In the protologue of *Solanuminaequilaterale* (Merrill 1906) two collections were cited, *Merrill 4807* and *Elmer 6204*, the latter with the comment “appears to be a form of this species”. We have selected the best preserved of the duplicates we have seen of *Merrill 4807* (US [00027616, acc. # 710478]) as the lectotype for this name, as Merrill unequivocally accepted this collection as his new species.

#### Specimens examined.

See Suppl. materials 1–3.

### ﻿35. Solanum pubescens

Taxon classificationPlantaeSolanalesSolanaceae

Willd., Phytographia 1: 5. 1794.

0881120A-7B69-5CA2-81E1-1E88DEAD3844

Figs 3D , 4D , 59

Solanum calycinum Nees, Trans. Linn. Soc. London 17(1): 60. 1834. Type. India. Sin. loc., “Herb. Madr. 237”, *Anonymous s.n.* [Wallich Catal. Suppl. n. 237] (lectotype, designated here: GZU [GZU000255511])
Solanum neesianum D.Dietr., Syn. Pl. (D. Dietrich) 1: 697. 1839, nom. illeg., non Solanumneesianum Wall. ex Nees, 1834. Type. Based on Solanumcalycinum Nees
Solanum esenbeckii Steud., Nomencl. Bot. ed. 2, 2: 602. 1841, nom. illeg. superfl. Type. Based on Solanumcalycinum Nees
Solanum conanthum Dunal, Prodr. [A. P. de Candolle] 13(1): 127. 1852, nom. illeg. superfl. Type. Based on Solanumcalycinum Nees

#### Type.

Cultivated. “Habitat in Indiae hortis, arboretis solo argilloso rarius”, *Anonymous s.n.* (lectotype, designated by Hepper 1987, pg. 369, as “type”: B [B-W04313-010]).

#### Description.

Erect shrubs to 5 m tall, unarmed. Stems erect, terete, stellate-pubescent and sticky glandular; pubescence of very short-stalked multangulate trichomes mixed with sessile porrect-stellate trichomes, the multangulate trichomes with more than 10 rays, the rays 0.4–0.5 mm long, the porrect-stellate trichomes with 6–8 rays, 0.4–0.5 mm long, the midpoints to 1 mm long, all trichomes usually glandular tipped and the plants sticky; new growth densely glandular-pubescent, the trichomes tangled, soon deciduous and the stems glabrate; bark of older stems greyish white. Sympodial units plurifoliate, the leaves not geminate. Leaves simple, unlobed, the blades 2.5–12 cm long, 1.5–7 cm wide, ca. 1–1.5 times longer than wide, ovate to broadly triangular, widest in the lower third, chartaceous, more or less concolorous, unarmed, the leaves of lower stems much larger than those of distal branches; adaxial surface evenly and densely pubescent with sessile and very short-stalked porrect-stellate trichomes, the rays 4–8, to 0.5 mm long, glandular at the tips, the midpoints 2–4-celled, to 2 mm long, glandular at the tips; abaxial surface with similar porrect-stellate trichomes, but these denser especially along the veins; major veins 3–4 pairs, densely pubescent especially abaxially; base abruptly truncate to cordate, somewhat oblique; margins entire or slightly sinuate, not lobed; apex acute; petioles 1–4 cm long, 1/2 of the leaf blade length, unarmed, more densely glandular stellate-pubescent than the stems, but the trichomes of the same morphology. Inflorescences to 3 cm long, internodal and lateral, unbranched, with 5–12 flowers, only 1 or 2 flowers open at any one time, densely glandular pubescent with mixed multangulate and stellate-porrect trichomes like those of the stems; peduncle 0.3–0.5 cm long; pedicels 1.2–1.6 cm long, ca. 0.5 mm in diameter at the base, ca. 1 mm in diameter at the apex, spreading at anthesis, glandular stellate-pubescent like the inflorescence axes, articulated at the base; pedicel scars irregularly spaced 1–3 mm apart. Buds elongate ellipsoid and tapering, strongly exserted from the calyx before anthesis. Flowers 5-merous, apparently all perfect. Calyx with the tube 2–2.5 mm long, conical, the lobes 3.5–5 mm long, ca. 1.5 mm wide, long-triangular to lanceolate, apically acute, densely stellate-pubescent abaxially with mixed glandular multangulate and porrect-stellate trichomes like those of the pedicels. Corolla 2–2.2 cm in diameter, violet or deep purple, stellate, lobed 3/4 of the way to the base, minimal interpetalar tissue present, the lobes 6–7 mm long, 4–4.5 mm wide, spreading at anthesis, mostly glabrous adaxially or with a few stellate trichomes along the petal midvein, densely stellate-pubescent abaxially with densely tangled sessile trichomes where exposed in bud, these densest at the tips. Stamens markedly unequal, with 4 short and one long and curved; long anther 7.5–9 mm long, 1–1.5 mm wide, strongly curved and tapering, short anthers 5–7 mm long, 1–1.5 mm wide, straight, all anthers yellow, glabrous, poricidal at the tips, the pores directed distally, not elongating to slits with drying; filament tube minute, glabrous; free portion of the filaments ca. 0.5 mm long, glabrous. Ovary conical, glabrous; style 9–10 mm long, strongly curved inwards and held adjacent to the long anther, glabrous; stigma capitate or slightly clavate, the surfaces minutely papillose. Fruit a globose berry, several per infructescence, 1–1.6 cm in diameter, orange-red when ripe, the pericarp thin and shiny, glabrous; fruiting pedicels 2.5–3.5 cm long, ca. 1.5 mm in diameter at the base, 2–2.5 mm in diameter at the apex, somewhat woody, spreading to pendent from weight of berries; fruiting calyx not accrescent, the lobes often breaking off. Seeds 20–30 per berry, 4–5 mm long, 3.5–4 mm wide, flattened reniform, yellowish or reddish brown, the surfaces minutely pitted, the testal cells with sinuate margins. Chromosome number: 2n = 24 (Chennaveeraiah and Krishnappa 1971; Rao and Rao 1987).

**Figure 59.**
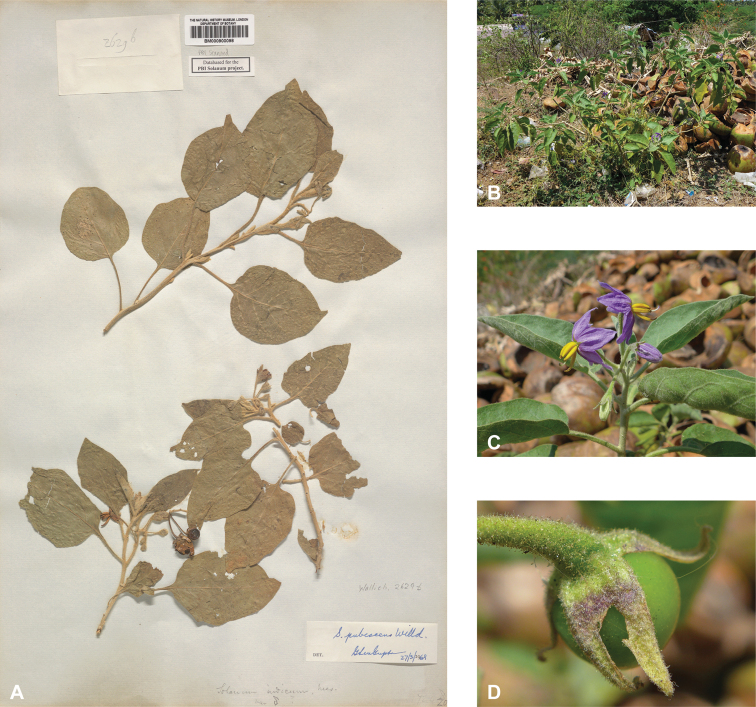
*Solanumpubescens* Willd. **A** herbarium specimen collected in India (*Wight 2629b*, BM000900098) **B** habit (*Sampath Kumar et al. 126956*, India) **C** inflorescence (*Sampath Kumar et al. 126956*, India) **D** immature fruit (*Sampath Kumar et al. 126956*, India). Photograph credits: **A** CC-BY, © copyright The Trustees of the Natural History Museum, London **B–D** X. Aubriot.

#### Distribution

**Figure 60.**
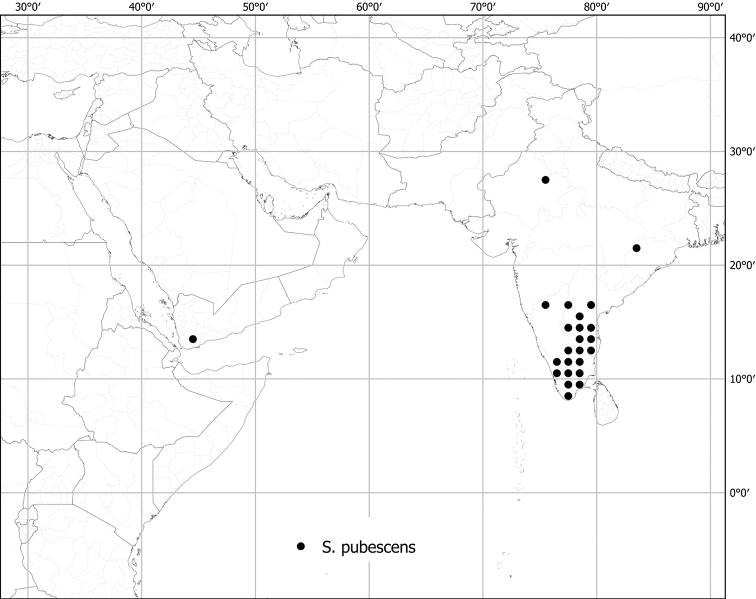
Distribution of *S.pubescens*.

**(Fig. 60).***Solanumpubescens* occurs from India to Saudi Arabia and Yemen on the Arabian Peninsula.

#### Ecology and habitat.

*Solanumpubescens* is found in a variety of dry forest types, such as thorn forest, often occurring in open areas and along roadsides, from 300 to 1,000 m elevation.

#### Common names and uses.

India. sonde, hucchu sonde, savadangi, cherichunda (Malayam), kaattu sundai kaai (Tamil), usthi kaai (Telugu) (https://www.flowersofindia.net). It is recorded as being used for bowel and joint pains (see https://www.flowersofindia.net).

#### Preliminary conservation status

**(IUCN 2019).** Least Concern (LC). EOO (996,977 km^2^, LC); AOO (272 km^2^, EN). Like many other spiny solanums in tropical Asia, *S.pubescens* is widely distributed in a variety of habitats, although the AOO suggests some concern, this is a common species.

#### Discussion.

*Solanumpubescens* is morphologically similar and probably closely related to *S.vagum*, sharing with that species zygomorphic flowers at anthesis, heteromorphic anthers and shiny berries on erect or slightly pendulous pedicels. Hepper (1987) treated material of *S.vagum* from Sri Lanka as *S.pubescens* in the “Revised Handbook to the Flora of Ceylon”. *Solanumpubescens* differs from *S.vagum* in its glandular pubescence, its narrowly elliptic leaves with attenuate bases and slightly smaller, violet (rather than white) flowers. In *S.pubescens* the lamina of young leaves is obscured by the dense covering of glandular stellate to multangulate stalked trichomes with elongate midpoints and the leaves are described as “oily to touch” (Singh 1988), while in *S.vagum* the pubescence of adaxial leaf surfaces is of very sparse sessile stellate trichomes with midpoints usually equal to the rays, and the lamina is clearly visible. The two species are sympatric in southern India.

*Solanumpubescens* and *S.vagum* share a zygomorphic androecium, with one anther distinctly longer than the rest. From herbarium sheets it appears that this difference becomes more pronounced with flower age. Post-anthesis anther expansion occurs in the unrelated *S.turneroides* Chodat (Brevantherum clade, see Stern et al. 2013) of southern South America. Buds of the African species *S.somalense* Franch. also have the anthers of more or less equal length that become different with age (Vorontsova and Knapp 2016). This phenomenon needs study with populations in the field and lab.

The Solanaceae from Nees van Esenbeck’s personal herbarium are held in Graz at GZU (Stafleu and Cowan 1981). The sheet in GZU is the only one we have found of the Wallich Herb. “Madras” gatherings cited in the protologue of *S.calycinum*. We therefore designate it (GZU000255511) as the lectotype of *S.calycinum*.

Dunal (1852) coined the replacement name *S.conanthum* for *S.calycinum* Nees citing “non Dunal” in reference to his own *S.calycinum* (= *S.macrocarpon* L. of Africa, the Gboma eggplant, see Vorontsova and Knapp 2016) that is a later homonym of Nees van Esenbeck’s name.

#### Specimens examined.

See Suppl. materials 1–3.

### ﻿36. Solanum putii

Taxon classificationPlantaeSolanalesSolanaceae

Kerr ex Barnett, Kew Bull. 16: 485. 1963.

DB6249AD-F5B8-52C9-82B8-C80B3A78670C

Fig. 61

#### Type.

Thailand. Prachuap Khiri Khan: Thap Sakae District, Hui Yang [Huai Yang], 6 Oct 1920, *Put Phraisurind 3227* (holotype: K [K000922027]; isotypes: BK [257531], BM [BM000886104]).

#### Description.

Shrubs of unknown height, unarmed. Stems erect, terete, stellate-pubescent; pubescence of sessile to very short-stalked porrect-stellate trichomes, the rays 4–8, ca. 0.2 mm long, the midpoints absent or to 0.1 mm long, much shorter than the rays; new growth densely stellate-pubescent, the trichomes white and tangled, soon deciduous and the stems glabrate; bark of older stems pale brown. Sympodial units plurifoliate, the leaves not geminate. Leaves simple, the blades 4–10 cm long, 1.5–5 cm wide, ca. 2 times longer than wide, elliptic, widest just below the middle, chartaceous, discolorous, unarmed; adaxial surface evenly and sparsely pubescent with mixed sessile and stalked porrect-stellate trichomes, the stalks to 0.5 mm long, the rays 4–8, to 0.5 mm long, the midpoints absent or to 0.5 mm long; abaxial surface moderately to densely pubescent with mixed stalked and sessile porrect-stellate trichomes, the stalks to 1.5 mm, the rays 8–10, to 0.5 mm long, the midpoints equalling the rays, the lamina clearly visible; major veins 4–5 pairs, densely pubescent especially abaxially; base abruptly truncate, usually strongly oblique; margins entire; apex acute to acuminate; petioles 1–2.5 cm long, ca. 1/4 as long as the leaf blades, unarmed and densely pubescent with weak-rayed porrect-stellate trichomes like those of the stems. Inflorescences 3–7 cm long, internodal and lateral, unbranched, with 10–20 flowers, apparently only a few flowers open at any one time, pubescent with porrect-stellate trichomes like those of the stems; peduncle 1.2–2.5 cm long; pedicels 0.9–1 cm long, ca. 1 mm in diameter at the base, ca. 1.3 mm in diameter at the apex, spreading and perhaps slightly nodding at anthesis, sparsely stellate-pubescent with porrect-stellate trichomes like the inflorescence axes, articulated at the base; pedicel scars evenly spaced 2.5–3 mm apart. Buds tapering, about halfway exserted from the calyx before anthesis. Flowers 5-merous, apparently all perfect (but isotype at BM has a flower dissection that is fasciated with 7 corolla lobes). Calyx with the tube 1.5–2 mm long, conical, the lobes 2–2.5 mm long, ca. 1 mm wide, deltate with a subulate tip to 1 mm long, sparsely stellate-pubescent with porrect-stellate trichomes like those of the pedicels. Corolla ca. 1.6 cm in diameter, white or pale lilac, stellate, lobed ca. halfway to the base, interpetalar tissue scarce, but the petal margins thin and somewhat “ruffly”, the lobes ca. 5 mm long, ca. 5 mm wide, spreading or slightly reflexed at anthesis, mostly glabrous adaxially but with a few stellate trichomes along the petal midveins, densely stellate-pubescent abaxially with densely tangled sessile trichomes where exposed in bud, these densest at the tips. Stamens equal or slightly unequal with 2 slightly longer; filament tube ca. 0.5 mm long, glabrous and papery; free portion of the filaments ca. 1 mm long, glabrous; anthers 4–4.5 mm long, ca. 0.7 mm wide, if unequal, 2 ca. 0.2 mm longer, slightly tapering, yellow, poricidal at the tips, the pores directed distally, not elongating to slits with drying. Ovary conical, glabrous but with a few trichomes at the style base; style 6–6.5 m, glabrous; stigma tiny, a mere broadening of the style apex, the surfaces minutely papillate. Fruit a globose berry, 0.5–0.6 cm in diameter (immature?), colour not known, the pericarp thin and shiny, glabrous; fruiting pedicels 1–1.5 cm long, ca. 1 mm in diameter at the base, tapering to an apex ca. 2 mm in diameter, somewhat woody, spreading; fruiting calyx not accrescent, the lobes often breaking off. Seeds ca. 20 per berry, 2–2.5 mm long, 1.5–2 mm wide, flattened reniform, reddish tan or yellowish brown, the surfaces deeply pitted, the testal cells with sinuate margins. Chromosome number: not known.

**Figure 61.**
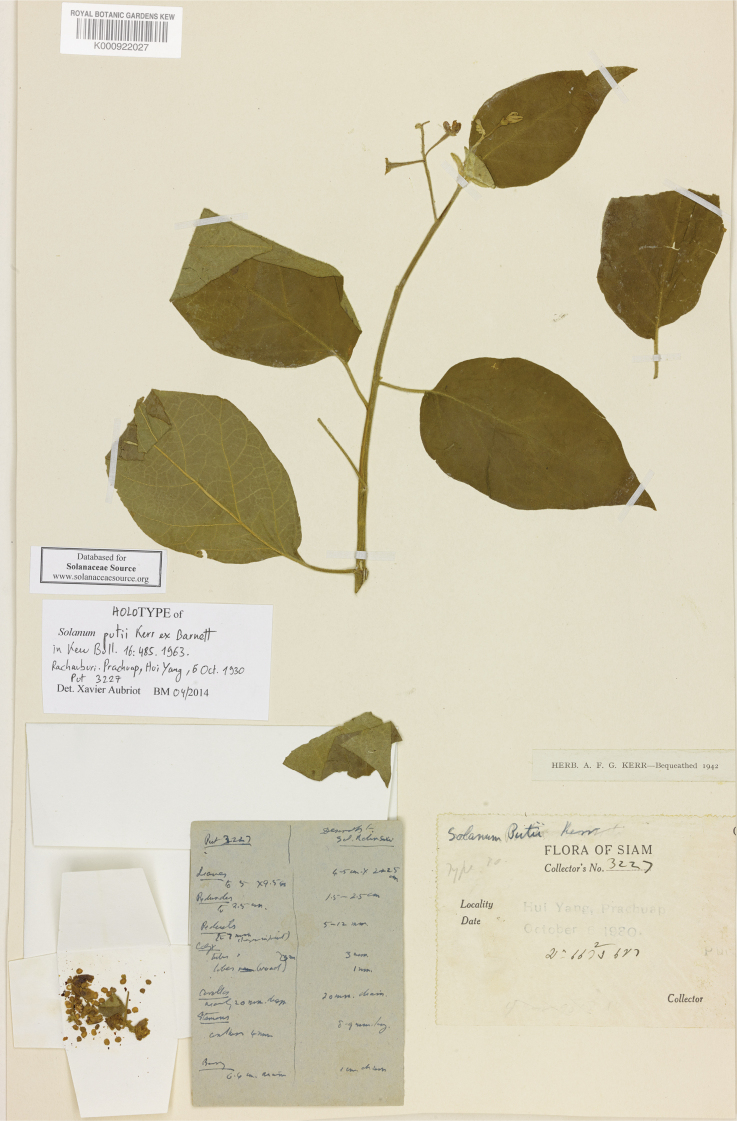
*Solanumputii* Kerr ex Barnett – Herbarium specimen (holotype) collected in Thailand in 1920 (*Put 3227*, K000922027). Photograph credit: © copyright of the Board of Trustees of the Royal Botanic Gardens, Kew.

#### Distribution

**Figure 62.**
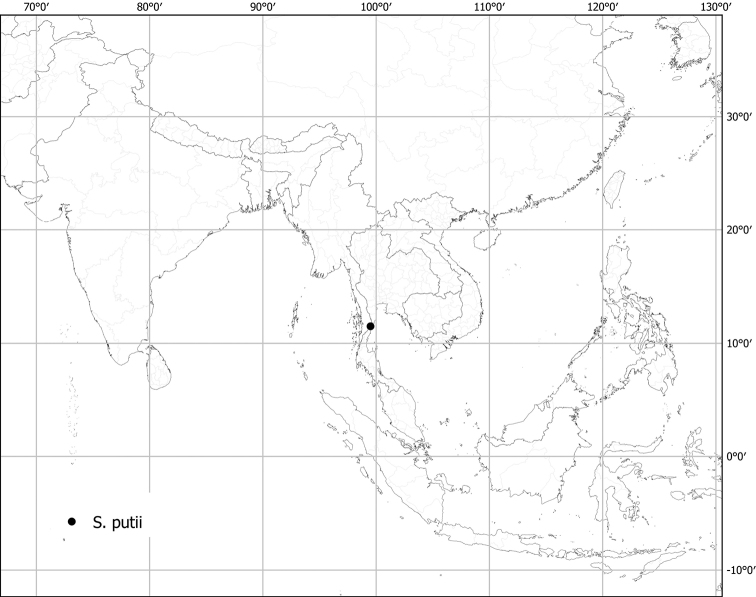
Distribution of *Solanumputii*.

**(Fig. 62).***Solanumputii* is endemic to Thailand and only known from the type collection. A single collection from Vietnam (*Nuraliev NUR-1863a*) appears to be this taxon, but we include it here with some reservation until further studies are undertaken.

#### Ecology and habitat.

No habitat notes are recorded on the type gathering of *S.putii*, but possibly occurs in dry forest.

#### Common names and uses.

None recorded.

#### Preliminary conservation status

**(IUCN 2019).** Data Deficient (DD). *Solanumputii* is only known from the type collection and little is known about this species otherwise.

#### Discussion.

*Solanumputii* is very similar and may be conspecific with *S.robinsonii*, but we hesitate to synonymise these two taxa until more material is available; the similarities were noted in the original description (notes in packet on holotype at K; Barnett 1963). The leaves of *S.putii* are more broadly elliptic, more softly pubescent, and less discolorous that those of *S.robinsonii*; they are also markedly oblique at the base, while those of *S.robinsonii* are not. The lamina beneath is clearly visible and the rays on the stellate trichomes are thinner and more delicate. A single dissected flower on the isotype sheet at BM is 7-merous and clearly fascinated, perhaps indicating this is an aberrant plant, a note by Kerr on the holotype at K states “Only flower of *Put 3227* examined was abnormal with double ovary, stamens, etc.”. The fruit of *S.putii* are smaller (ca. 0.6 cm in diameter) than those of *S.robinsonii* (ca. 1 cm in diameter).

#### Specimens examined.

See Suppl. materials 1–3.

### ﻿37. Solanum retrorsum

Taxon classificationPlantaeSolanalesSolanaceae

Elmer, Leaflets Philipp. Bot. 1: 342. 1908.

329F65D6-86E1-55D9-857D-79138D23ABC9

Fig. 63

Solanum luzoniense Merr., Philipp. J. Sci. Bot. 13: 58. 1918. Type. Philippines. Ilocos: Luzon, Central Luzon, Pangasinan Province, Mount Umingan, Aug 1910, *M. Ramos & G.E. Edaño [Bur. Sci.] 26487* (lectotype, designated here: K [K000195878]; isolectotype: US [acc # 1376054, US00027662]).
Solanum luzoniense Merr. var. glabrum Merr., Philipp. J. Sci. Bot. 13: 59. 1918. Type. Philippines. Ilocos: Luzon, Central Luzon, Pampanga Province, Calumpit, Sep 1905, *E.D. Merrill 4237* (lectotype, designated here: K [K000195959]; isolectotype: US [acc # 710167, US00027663]).

#### Type.

Philippines. CAR: Luzon, Benguet Province, Baguio, Mar 1907, *A.D.E. Elmer 8719* (lectotype, designated here: K [K000195910]; isolectotypes: A [0077850], E [E00243613]).

#### Description.

Shrubs or scrambling shrubs to 1.5 m, armed or unarmed. Stems erect, terete, prickly or unarmed, moderately stellate-pubescent; prickles if present 3–5 mm long, straight (occasionally somewhat slightly curved) and downwardly pointing (retrorse), yellowish tan; pubescence of mixed sessile and short-stalked porrect-stellate trichomes, the stalks to 0.2 mm, the rays 6–8, ca. 0.5 mm long, the midpoints equal to the rays, the trichomes persistent or deciduous and the stems glabrate; new growth moderately to densely stellate-pubescent, the trichomes whitish cream, mixed sessile and short-stalked porrect-stellate like those of the stems; bark of older stems whitish grey. Sympodial units plurifoliate, the leaves not geminate. Leaves simple, not lobed or occasionally very shallowly lobed, the blades 3–15 cm long, 1–6 cm wide, 2–3 times longer than wide, narrowly elliptic to lanceolate, widest at the middle, chartaceous, somewhat discolorous, usually unarmed; adaxial surface glabrous to moderately and evenly stellate-pubescent with mixed sessile and short-stalked porrect-stellate trichomes, the stalks to 0.2 mm, the rays 6–8, ca. 0.5 mm long, the midpoints equal to the rays; abaxial surface similarly but more densely pubescent with porrect-stellate trichomes, the lamina still visible; major veins 5–7 pairs, barely visible above, stellate pubescent especially abaxially; base attenuate, usually somewhat oblique; margins entire or rarely very shallowly lobed, the lobes 1–2 on each side, ca. 0.5 cm long, broadly deltate with rounded or acute tips, the sinuses reaching less than 1/8 of the distance to the midrib; apex acute to acuminate; petioles 0.6–2.5 cm long, ca. 1/4 as long as the leaf blades or less, unarmed and densely stellate-pubescent with mixed sessile and short-stalked porrect-stellate trichomes like those of the stems, pubescence denser than that of stems. Inflorescences 1.5–4.5 cm long, internodal and lateral, unbranched or very occasionally forked, with 10–30 flowers, only a few flowers open at any one time, sparsely to densely pubescent with porrect-stellate trichomes like those of the stems; peduncle 0.3–0.8 cm long, to 1.5 cm long on each of the inflorescence branches; pedicels 0.6–0.75 cm long, ca. 0.5 mm in diameter at the base, ca. 0.5 mm in diameter at the apex, spreading and perhaps slightly nodding at anthesis, stellate-pubescent with porrect-stellate trichomes like the inflorescence, articulated at the base; pedicel scars irregularly spaced 2–4 mm apart. Buds elongate and tapering, approximately halfway exserted from the calyx before anthesis. Flowers 5-merous, apparently all perfect. Calyx with the tube 1.5–2 mm long, conical, the lobes 2–2.5 mm long, ca. 2 mm wide, deltate to narrowly deltate, densely stellate-pubescent like the pedicels. Corolla 1–1.4 cm in diameter, white, stellate, lobed nearly to the base, the lobes 4–7 mm long, 3–4 mm wide, narrowly triangular, spreading at anthesis (perhaps slightly reflexed?), glabrous adaxially, moderately stellate-pubescent abaxially where exposed in bud. Stamens equal; filament tube minute, glabrous; free portion of the filaments ca. 0.5 mm long, glabrous; anthers 3–4.5 mm long, 1–1.2 mm wide, strongly tapering, yellow, poricidal at the tips, the pores directed distally, not elongating to slits with drying. Ovary conical, glabrous; style 6–8 mm long, glabrous or with a few stalked stellate trichomes near the base; stigma capitate, the surfaces minutely papillose. Fruit a globose berry, 0.6–0.8 cm in diameter, bright red when ripe, the pericarp thin and shiny, glabrous; fruiting pedicels 0.8–1.8 cm long, 0.7–1.1 mm in diameter at the base, 1.5–2 mm in diameter at the apex, somewhat woody, erect or somewhat spreading; fruiting calyx not accrescent, the lobes often breaking off. Seeds 6–10 per berry, 3.5–5 mm long, 1.5–3.5 mm wide, flattened reniform with incrassate margins, yellowish tan, the surfaces minutely pitted, the testal cells with sinuate margins. Chromosome number: not known.

**Figure 63.**
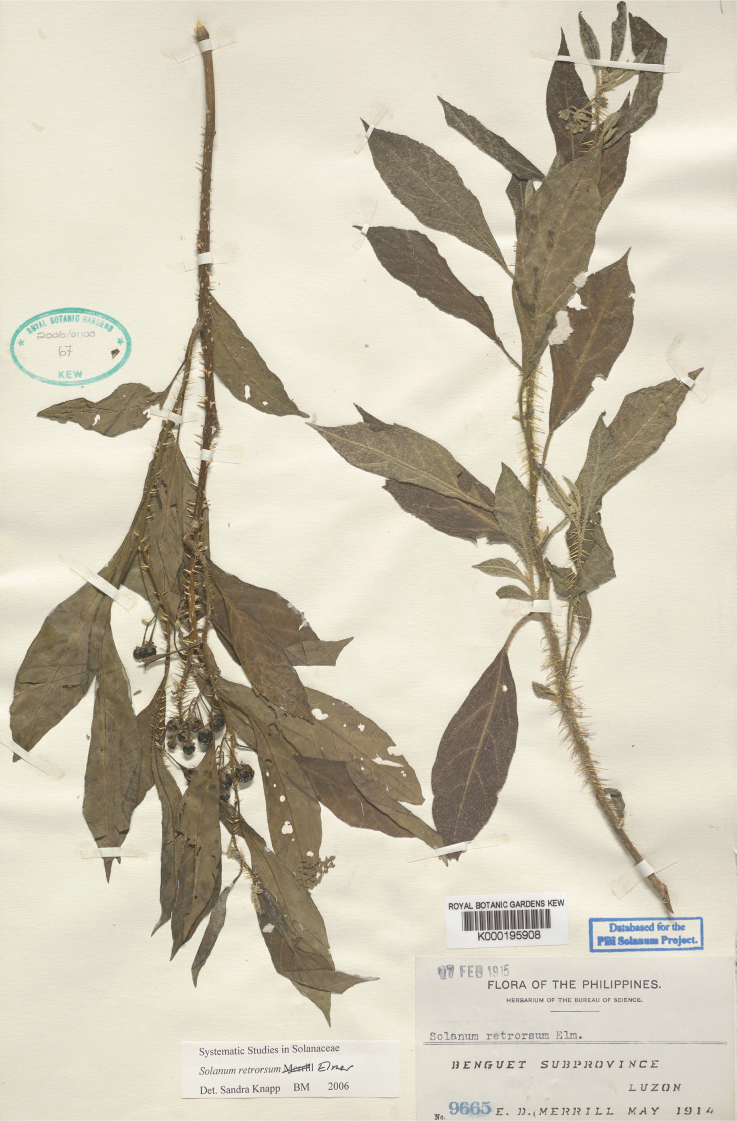
*Solanumretrorsum* Elmer – Herbarium specimen collected in the Philippines in 1914 (*Merrill 9665*, K000195908) Photograph credit: © copyright of the Board of Trustees of the Royal Botanic Gardens, Kew.

#### Distribution

**Figure 64.**
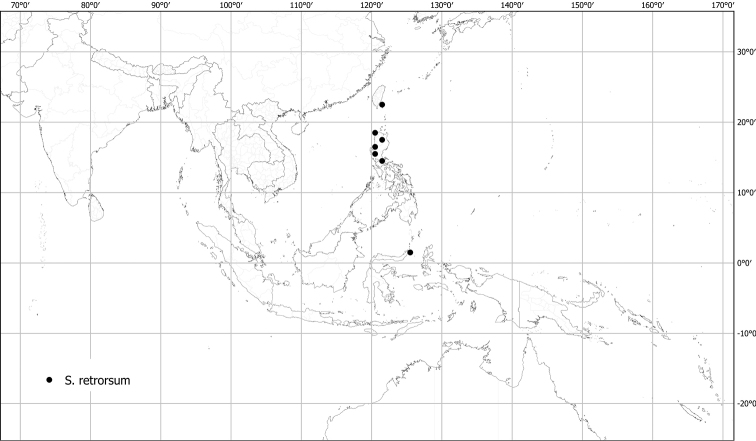
Distribution of *S.retrorsum*.

**(Fig. 64).***Solanumretrorsum* occurs from the very northern part of Sulawesi (a single collection) in Indonesia to the Philippines, where it is much more common. The single collection from Lanyu (Orchid) Island off the southeastern edge of Taiwan is a very poor specimen and is included here with some hesitation.

#### Ecology and habitat.

*Solanumretrorsum* grows in thickets, forests and forest edges in broadleaf evergreen and semideciduous woodlands; few of the collections we have seen have specific habitat information; elevations from 500 to1,600 m elevation.

#### Common names and uses.

Taiwan. lü song qie (Zhang et al. 1994, as *S.luzoniense*, but this identification is tentative).

#### Preliminary conservation status

**(IUCN 2019).** Near Threatened (LC). EOO (115,321 km^2^, LC); AOO (48 km^2^, EN). *Solanumretrorsum* is found throughout the Philippines, with populations in a variety of habitats. The range extends to Sulawesi, making it widely distributed. The status of populations outside the main range in the Philippines, however, needs further investigation and the few recent collections suggest that the species merits some conservation concern.

#### Discussion.

*Solanumretrorsum* is polymorphic for prickliness; some plants are densely prickly with the downward point prickles that give the species its name, while others lack prickles entirely. These unarmed plants have been called *S.luzoniense.* Despite this polymorphism *S.retrorsum* is distinctive, with its narrowly elliptic leaves and very small ripe berries, and not easily confused with any other species in the region.

The polymorphism in prickliness seen in *S.retrorsum* is also found in *S.ratale* D.McClelland of Fiji (see McClelland et al. 2020). That species also has unarmed and densely prickly individuals with strongly retrorse prickles mainly on the stems; the plurifoliate sympodial units, trichomes with midpoints equal to the rays and bright red berries (versus difoliate sympodia, small trichomes with elongate midpoints and black to deep purple berries in *S.ratale*), as well as their distinct distributions, clearly distinguish the two species.

The description of *S.luzoniense* in Zhang et al. (1994) was taken from a mixture of material from Taiwan and the Philippines. Material from Taiwan was a mixture of *S.miyakojimense* (*Yang 8701*) and a very scrappy specimen from Lanyu Island (*Leu et al. 2118*) that we here tentatively assign to *S.retrorsum*, but with considerable hesitation. The occurrence of *S.retrorsum* in Taiwan remains to be confirmed.

Merrill (1918b) did not specifically designate a type for his *S.luzoniense.* He cited “Bur. Sci. 24687 Ramos & Edaño”, “Bur. Sci. 17710 Otanes” and “Merrill s.n., July 1903”; we have selected the Kew duplicate of *Ramos & Edaño [Bur. Sci.] 26487* (K000196878) as the lectotype because it is well preserved and has both flower and fruit. For var. glabrum, he clearly stated that his own collection (*Merrill 4237*) was the type but did not specify the herbarium. We have selected the Kew duplicate of this collection (K000195959) as the lectotype of the varietal name because it is the best-preserved specimen of all the duplicates we have seen.

#### Specimens examined.

See Suppl. materials 1–3.

### ﻿38. Solanum robinsonii

Taxon classificationPlantaeSolanalesSolanaceae

Bonati, Bull. Soc. Bot. Genève, sér. 2, 3: 311. 1914.

F305F726-1D61-52EA-B358-A7E4F8D755F5

Figs 2C , 65

#### Type.

Vietnam. Khánh Hòa: Nha-trang and vicinity, 11–26 Mar 1911, *C.B. Robinson 1082* (lectotype, designated here: P [P00054128]; isolectotype: K [K000922030]).

#### Description.

Erect shrub, up to 1 m tall, unarmed. Stems erect, terete, sparsely to moderately stellate-pubescent, glabrescent; pubescence of mixed sessile and short-stalked porrect-stellate trichomes, the stalks to 0.08 mm long, the rays 7–8, 0.1–0.2 mm long, the midpoints to 0.03 mm long with bulbous bases; new growth densely stellate-pubescent, light brownish green; bark of older stems brownish grey, glabrous. Sympodial units plurifoliate, the leaves not geminate. Leaves simple, entire, the blades 3–8 cm long, 1–3 cm wide, ca. 2–3 times longer than wide, elliptic to ovate, chartaceous, strongly discolorous; adaxial surface moderately stellate pubescent, the trichomes porrect-stellate, sessile and short-stalked, the stalks extremely short, the rays 6–8, 0.05–0.15 mm long, the midpoints to 0.08 mm long, with bulbous bases; abaxial surface densely stellate-pubescent with sessile and short-stalked porrect-stellate trichomes, the stalks if present to 0.5 mm, the rays 5–10, 0.2–0.5 mm long, the midpoints to 0.2 mm, stout with a bulbous base, the lamina completely obscured; major veins 4–9 pairs, markedly impressed above, drying light green; base cuneate; margins entire or somewhat undulate, never lobed; apex rounded to acute; petiole 0.5–1 cm long, 1/10–1/5 of the leaf blade length, densely stellate-pubescent with trichomes like those of the stems and leaves. Inflorescences 1–3 cm long, internodal and lateral, unbranched, with ca. 5–10 flowers, only a few flowers open at any one time, densely stellate pubescent with porrect-stellate trichomes like those of the stems; peduncle 0.4–2.5 cm long; pedicels 4–9 mm long, 0.7–1 mm in diameter at the base, 1.3–1.5 mm in diameter at the apex, erect to recurved, densely stellate-pubescent with porrect stellate-trichomes like those of the axes, articulated at the base; pedicel scars spaced 0.5–2 mm apart. Buds elongate-ovoid, pointed at the tip, the corolla ca. halfway exserted from the calyx before anthesis. Flowers 5-merous, apparently all perfect. Calyx with the tube 1.4–2 mm long, campanulate, the lobes 2–3 mm long, 0.6–0.8 mm wide, narrowly deltate and narrowing to an elongate acumen, the acumen 2/3–3/4 the total lobe length, densely stellate-pubescent with porrect stellate-trichomes like those of the pedicels. Corolla 1–2 cm in diameter, blue to purple, stellate, lobed ca. 1/2–2/3 of the way to the base, the lobes 4–5 mm long, 2–2.5 mm wide, deltate, spreading at anthesis, densely stellate-pubescent abaxially on parts exposed in bud. Stamens equal or very slightly unequal with 2 longer than the rest; anthers 5–6 mm long, 0.5–0.75 mm wide, if unequal then 2 ca. 0.1 mm longer than the rest, tapering, connivent, yellow, glabrous, poricidal at the tips, the pores not lengthening to slits with age; filament tube <0.5 mm long; free portion of the filaments 0.4–0.75 mm, glabrous. Ovary conical, with minute glandular hairs at the top; style 7.5–9 mm long, slender, curved at the apex, glabrous; stigma capitate, minutely papillate. Fruit a globose berry, 1–2 per infructescence, 0.7–1.2 cm in diameter, the pericarp smooth, red when mature, glabrous; fruiting pedicels 1.2–1.6 cm long, ca. 0.5 mm in diameter at the base, 1.5–1.8 mm in diameter at the apex, woody, erect to recurved; fruiting calyx lobes slightly expanding to 4–5 mm long, 1/2–2/3 the length of the mature fruit, narrowly deltate, appressed to the berry, ending with a long acumen. Seeds ca. 10–15 per berry, 3–4 mm long, 2.5–3 mm wide, flattened-reniform, dull yellow, the surface minutely pitted, the testal cells pentagonal in outline. Chromosome number: not known.

**Figure 65.**
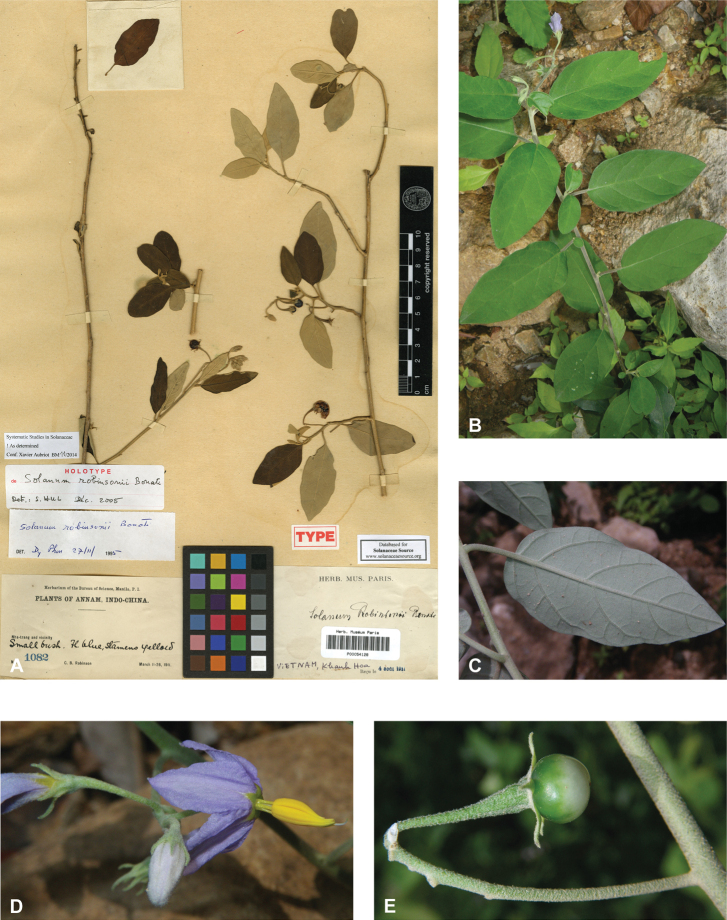
*Solanumrobinsonii* Bonati **A** herbarium specimen (holotype) collected in Vietnam in 1911 (*Robinson 1082*, P00054128) **B** habit (*Nuraliev 3031*, Vietnam) **C** detail of the abaxial surface of a leaf blade (*Nuraliev 3031*, Vietnam) **D** inflorescence and detail view of an open flower (*Nuraliev 3031*, Vietnam) **E** detail view of a fruit (*Nuraliev 3031*, Vietnam). Photograph credits: **A** CC-BY, Muséum national d’Histoire naturelle, Paris **B–E** M. Nuraliev.

#### Distribution

**Figure 66.**
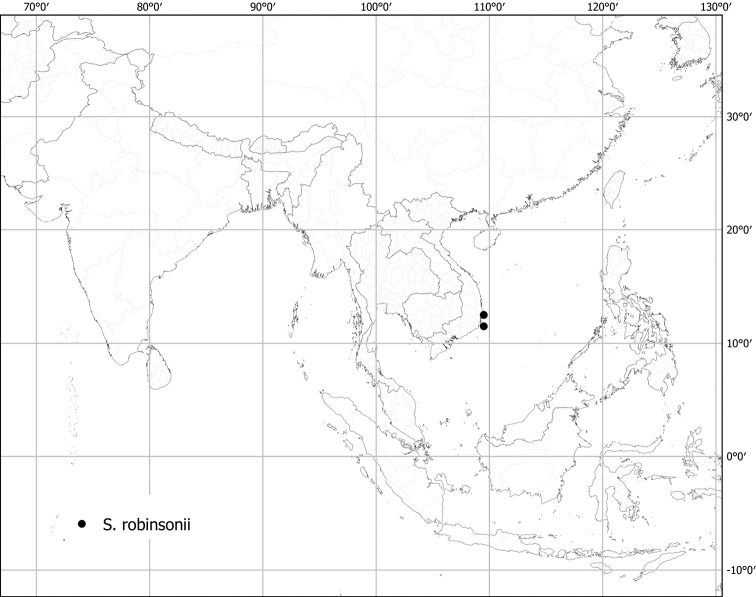
Distribution of *S.robinsonii*.

**(Fig. 66).***Solanumrobinsonii* is endemic to Vietnam; the few known collections are restricted to Khánh Hòa and Ninh Thuân provinces of southeast Vietnam.

#### Ecology and habitat.

*Solanumrobinsonii* grows on hillsides in deciduous forests, at ca. 200 m elevation.

#### Common names and uses.

None recorded.

#### Preliminary conservation status

**(IUCN 2019).** Endangered (EN [B1,2abii,iii]). EOO (610 km^2^, EN); AOO (20 km^2^, EN). *Solanumrobinsonii* has a narrow distribution and grows in coastal forests that are subject to much anthropogenic disturbance. The species is of concern due to extreme habitat alteration (Wikramanayake et al. 2002).

#### Discussion.

*Solanumrobinsonii* is superficially similar to *S.putii* of Thailand and *S.nienkui* of Hainan Island, China. The three taxa share a spindly shrubby habit, long, thin anthers and small red berries. *Solanumrobinsonii* can be distinguished from *S.putii* in its more narrowly elliptic leaves that are more strongly discolorous when dry, and from *S.nienkui* in its slightly larger corollas (to 2 cm in diameter versus to 1.3 cm in diameter) that are less deeply stellate, and in its longer style (7.5–9 mm long versus 5.5–6 mm long).

The strongly discolorous leaves of *S.robinsonii* are reminiscent to those of *S.giganteum*, but that species has highly branched inflorescences and stout, prickly stems. Specimens of *S.robinsonii* have been considered to look like *S.elaeagnifolium* (annotation by D.E. Symon on the isolectotype specimen at Kew); this is due to the bulbous bases of the midpoints that make the trichomes look slightly lepidote. *Solanumelaeagnifolium*, however, has true lepidote trichomes that are more shield-like in structure (see Knapp et al. 2017).

Hul and Dy Phon (2014) erroneously cited the specimen in P as the holotype of *S.robinsonii*. Although Bonati worked in Paris, he did not cite any herbarium in the protologue or in the introductory part of his treatment (Bonati 1914), so lectotypification is necessary. We here select the Paris sheet (P00054128) annotated by Hul and Dy Phon as “holotype” as the lectotype for *S.robinsonii*.

#### Specimens examined.

See Suppl. materials 1–3.

### ﻿39. Solanum robustum

Taxon classificationPlantaeSolanalesSolanaceae

H.L.Wendl., Flora 27: 784. 1844.

6F245086-99CC-5EAD-A833-ACDFE44737AB

Fig. 41C–E No names based on tropical Asian types have been published; for complete synonymy see Vorontsova and Knapp 2016.

#### Type.

Cultivated in Hanover, Germany, from seeds sent from Brazil, *Anonymous s.n.* (no herbarium specimens cited, none found).

#### Description.

Vorontsova and Knapp (2016: 280–284); http://www.solanaceaesource.org/solanaceae/solanum-robustum.

#### Distribution.

*Solanumrobustum* has been collected in India (state of Tamil Nadu) and on the island of Réunion (two collections from the 1960s); it is native to southern South America. Outside of its native range it is apparently used as an ornamental (see Vorontsova and Knapp 2016).

#### Discussion.

*Solanumrobustum* is a distinctive species with strongly winged stems and small, pubescent berries. Even vegetatively it is not easily confused with any other species in the region. It has the potential to become a serious pest; in Hawai’i it is classified as a noxious weed (Wagner et al. 1999).

*Solanumrobustum* was described without reference to herbaria or specific collections; we, like Vorontsova and Knapp (2016), leave neotypification of this name for a monographer of the group to which this distinctive species belongs (Erythrotrichum clade).

#### Specimens examined.

See Suppl. materials 1–3.

### ﻿40. Solanum schefferi

Taxon classificationPlantaeSolanalesSolanaceae

F.Muell., Descr. Notes Papuan Pl. 1: 44. 1876.

05942791-6F92-551A-B986-99376D7924D5

Fig. 67

Solanum incanum Scheff., Ann. Jard. Bot. Buitenzorg 1: 39. 1876, nom. illeg., non Solanumincanum L., 1753. Type. Indonesia. West Papua: Andai (“Andaj”), sin. dat., *G. Teijsmann s.n.* (lectotype, designated here: BO [acc. # 1324395]; isolectotype: BO [acc. # 1323468]).
Solanum lianoides Elmer, Leaflets Philipp. Bot. 2: 733. 1910. Type. Philippines. MIMAROPA: Luzon, Romblon Province, Sibuyan Island, May 1910, *A.D.E. Elmer 10752* (lectotype, designated here: BM [BM000778113]; isolectotypes: A [00077845], BISH [BISH1005079], CAL [acc. # 316476], E [E00273864], F [v0073460F, acc. # 290900], G [G00442946], GH [GH00077844], HBG [HBG511454], K [K000196018], LE [LE00016968, LE00016969], MO [MO-503846, acc. # 3783069], NY [00172284], US [00027651, acc. # 779390]).
Solanum smilacocladum Bitter, Bot. Jahrb. Syst. 55: 79. 1919. Type. Indonesia. Sulawesi: [Sulawesi Utara] “Celebes (Minahassa): Bojong, N. Celebes”, 1888, *O. Warburg 15072* (lectotype, designated here: NY [00804057]).

#### Type.

Based on (replacement name for) *Solanumincanum* Scheff., non L.

#### Description.

Scrambling shrubs or woody lianas to 10 m long, armed. Stems flexuous, terete, moderately prickly and sparsely to densely stellate-pubescent; prickles 2–4 mm long, 2–2.5 mm wide at the base, strongly recurved (hooked), pale yellowish tan; pubescence of stalked porrect-stellate trichomes, the stalks to 0.5 mm long, multiseriate, the rays 4–8, 0.2–0.4 mm long, the midpoints absent or slightly longer than the rays; new growth moderately to densely stellate-pubescent, the trichomes sessile to short-stalked, the stalks 0.2–0.4 mm, the rays 4–8, 0.2–0.5 mm long, the midpoints ca. 0.4 mm long, equal to the rays; bark of older stems reddish brown and somewhat shiny. Sympodial units unifoliate, the leaves not geminate. Leaves simple, not lobed, the blades 2.5–15 cm long, 1.5–7.5 cm wide, ca. 2 times longer than wide, elliptic-ovate to ovate, widest in the middle or the lower third, chartaceous, somewhat discolorous, armed or unarmed; adaxial surface evenly and sparsely to densely pubescent with mixed sessile and short-stalked porrect-stellate trichomes, the multiseriate stalks to 0.2 mm long and conical, the rays 4–6, 0.1–0.2 mm long, sometimes ascending (pointing upwards from the stalk), the midpoints absent or very short; abaxial surface moderately to densely pubescent with mixed stalked and sessile porrect-stellate trichomes like the adaxial surface, but the pubescence denser, especially along the principal veins; major veins 4–6 pairs, impressed above, densely pubescent especially abaxially; base acute, usually strongly oblique; margins entire; apex acute; petioles 1–1.2 cm long, 1/10–1/5 as long as the leaf blades, densely pubescent with porrect-stellate trichomes like those of the stems, unarmed or armed with 0–5 hooked prickles. Inflorescences 3–10 cm long, internodal and lateral, unbranched or more commonly many times branched, with (2–)10–40 flowers, only a few flowers open at any one time, pubescent with porrect-stellate trichomes like those of the stems, occasionally with a few minute hooked prickles in larger inflorescences; peduncle 0.5–3 cm long; pedicels 0.8–1 cm long, ca. 1 mm in diameter at the base, ca. 1.5 mm in diameter at the apex, spreading, sparsely to moderately stellate-pubescent with porrect-stellate trichomes like the inflorescence axes, articulated above the base leaving a small peg on the inflorescence axis, the pegs 0.5–1 mm long, more glabrous than either the pedicels or the inflorescence axis, drying dark brown to black; pedicel scars irregularly spaced 1–3 mm apart, often raised as short pegs ca. 1 mm long. Buds stout and strongly tapering, strongly exserted from the calyx before anthesis. Flowers 5-merous, heterostylous with the distal flowers on each inflorescence branch short-styled and the plants weakly andromonoecious. Calyx with the tube 2.5–3 mm long, conical, in bud a hyaline cup with darker acumens from the rim, tearing irregularly at anthesis, the lobes 1–2 mm long including the acumens, ca. 1 mm wide, deltate with a subulate tip to 1 mm long, sparsely to moderately stellate-pubescent with porrect-stellate trichomes like those of the pedicels, unarmed. Corolla 2–3 cm in diameter, violet to dark purple, deeply stellate, lobed nearly to the base, interpetalar tissue scarce, but the petal margins thin and somewhat “ruffly”, the lobes 10–14 mm long, 3.5–5 mm wide, spreading at anthesis, mostly glabrous adaxially but in more pubescent individuals with porrect-stellate trichomes on the petal surface, densely stellate-pubescent abaxially with densely tangled stalked porrect-stellate trichomes where exposed in bud, the trichomes with 5–8 rays ca. 0.5 mm long and midpoints equal to the rays, sometimes tinged with purple, the petal margins and tips densely papillate, the tips cucullate. Stamens equal or very slightly unequal; anthers 6–8 mm long, ca. 1.5 mm wide in the lower third, strongly tapering, yellow, poricidal at the tips, the pores directed distally, not elongating to slits with drying; filament tube minute, glabrous; free portion of the filaments ca. 0.5 mm long, glabrous. Ovary conical, sparsely pubescent with simple glandular trichomes and papillae; style in long-styled flowers 9–10 mm long, strongly hooked at the tip, minutely glandular papillate in the lower 1/4, otherwise glabrous, in short-styled flowers ca. 1 mm long, straight, glabrous; stigma slightly lobed, borne on the inner surface of the hooked style tip, the surfaces minutely papillose. Fruit a globose or ovoid berry, 2–3 cm long, ca. 3 cm in diameter (fide Symon 1985: 145; 3–5 cm long fide Elmer 1910: 734), green, the pericarp leathery and shiny, glabrous; fruiting pedicels 1.8–3 cm long, ca. 1 mm in diameter at the base, tapering to an apex ca. 3 mm in diameter, woody, spreading or pendent from weight of berries; fruiting calyx slightly accrescent, the lobes ca. 5 mm long, ca. 2 mm wide, spreading. Seeds more than 100 per berry, ca. 2 mm long, ca. 1.5 mm wide, reddish brown or tan, the surfaces very minutely pitted or smooth, the testal cells not visible. Chromosome number: not known.

**Figure 67.**
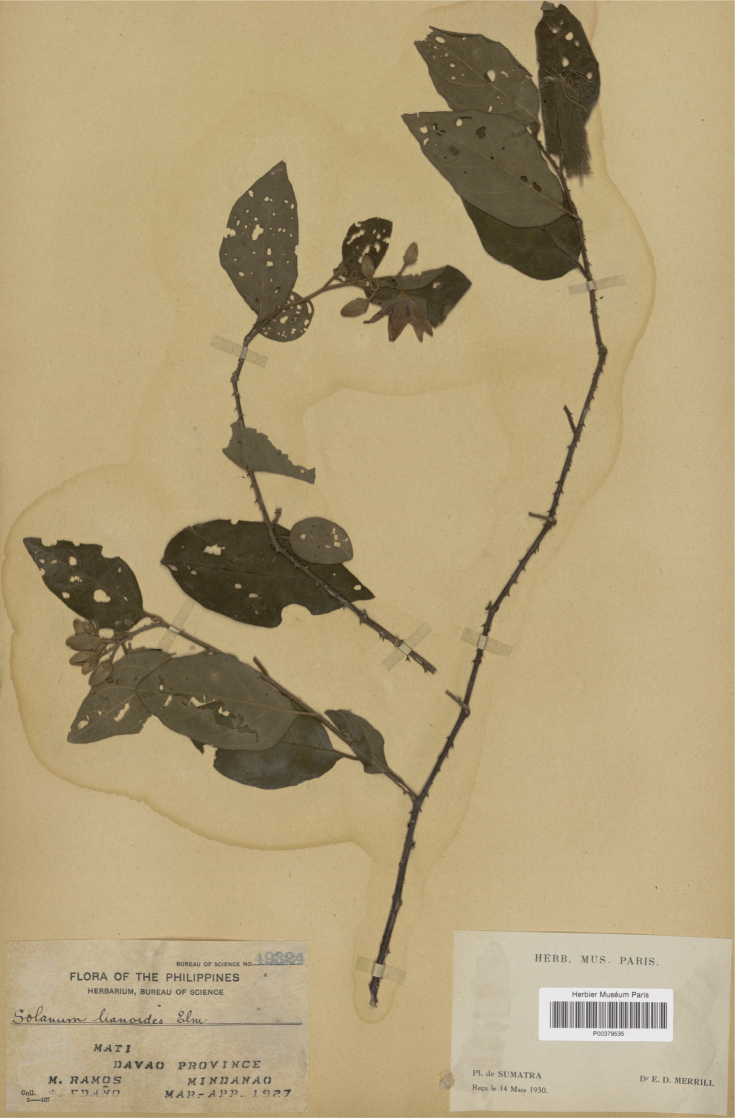
*Solanumschefferi* F.Muell. Herbarium specimen collected the Philippines in 1927 (*Ramos & Edaño 49324*, P00379535). Photograph credit: CC-BY, Muséum national d’Histoire naturelle, Paris.

**Figure 68.**
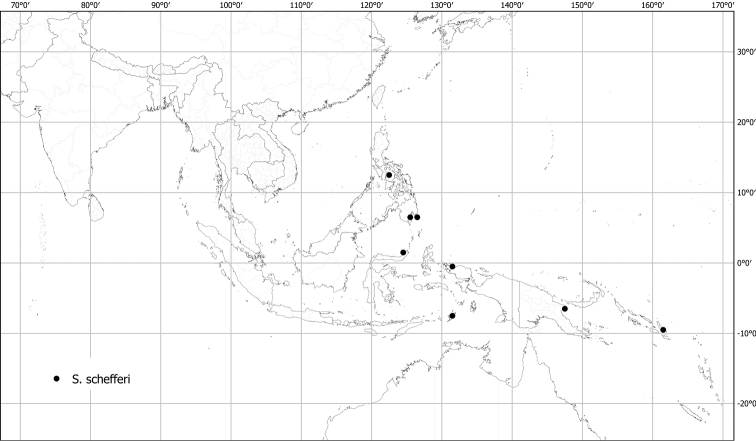
Distribution of *S.schefferi.*

#### Distribution

**(Fig. 68).***Solanumschefferi* occurs from the Philippines to northern New Guinea and the Solomon Islands (Malaita Island) to northern Sulawesi and the Malakus in Indonesia.

#### Ecology and habitat.

*Solanumschefferi* has been collected growing in open areas near abandoned fields or at forest edges from approximately sea level to 1,500 m elevation. It appears to grow on limestone, a soil type unusual for *Solanum*.

#### Common names and uses.

None recorded.

#### Preliminary conservation status

**(IUCN 2019).** Least Concern (LC). EOO (1,012,899 km^2^, LC); AOO (32 km^2^, EN). In the area of tropical Asia treated here *S.schefferi* is at the northwestern part of its range; it is more widely distributed on New Guinea. The small AOO is common in vines of forest canopies, these types of plants are notoriously under-collected but are also often rare where they occur.

#### Discussion.

*Solanumschefferi* is a species with most of its distribution on the island of New Guinea, but several collections are from the Malaku islands and northern Sulawesi. Symon (1985) suggested this distribution and its distinct differences from other New Guinea species suggested it was an introduction from the Americas. Molecular phylogenetic study, however, shows that *S.schefferi* is clearly a member of the Eastern Hemisphere clade of spiny solanums (Aubriot et al. 2016a) and is member of a small monophyletic group containing *S.athenae* Symon and *S.leptacanthum* Merr. & L.M.Perry, both New Guinea endemics (but see below).

*Solanumschefferi* is a striking species with large, showy (fide *Brass 24330*) flowers and large fruits. The plants climb with their hooked prickles (like rose thorns) and unlike many other species of New Guinean spiny solanums, the prickles on all parts are hooked. The berries are ovoid when young and grow to a considerable size (fide Elmer 1910: 734; Symon 1985: 145); smaller berries appear to be globose, elongating to ovoid with maturity.

*Solanumlianoides* has not previously be considered a synonym of *S.schefferi* (Symon 1985), but examination of the pubescence and flowers leads us to consider them as belonging to the same taxon. Molecular results of Aubriot et al. (2016a) place *S.lianoides* in the larger Sahul-Pacific clade and forming a group with *S.dunalianum* and *S.graciliflorum*, not closely related to *S.schefferi.* These results remain to be revisited to ascertain if contamination of one or other of these samples influenced the results. It is clear, however, that *S.schefferi* is native to tropical Asia, and is not an introduction from the Americas.

The protologue of *S.lianoides* cites a single collection (*Elmer 10752*) but does not mention a herbarium (Elmer 1910). We have selected the only one of the many duplicates of this collection we have seen that has both flowers and fruits (BM000778113) as the lectotype for this name.

In coining the replacement name *S.smilacocladum*Bitter (1919) cited the same sheets in BO as those used by Scheffer in his description of “S.incanum” (“Herb. Buitenzorg 9853! Sub nom. *S.incanum* Scheff.”), as well as additional syntypes from Berlin (*Schlechter 17627*, *Warburg 15072*, *Warburg 21246*) and Bogor (*Koorders 18033B*, *Koorders 18046B*). We have lectotypified *S.smilacocladum* with *Warburg 15072* from northern Sulawesi (NY 00804057), to not make the name homotypic with *S.schefferi.*

#### Specimens examined.

See Suppl. materials 1–3.

### ﻿41. Solanum sisymbriifolium

Taxon classificationPlantaeSolanalesSolanaceae

Lam., Tabl. Encycl. 2: 25. 1794, as “ sisymbrifolium”.

A2AAE003-0282-5FAE-BE7F-9774DDAA1667

Fig. 41F–H No names based on tropical Asian types have been published; for complete synonymy see Vorontsova and Knapp 2016.

#### Type.

Argentina. Buenos Aires: Buenos Aires, *P. Commerson s.n.* (lectotype, designated by Vorontsova and Knapp 2016, pg. 307: P-LA [P00357630, lower plant fragment, Morton neg. 8391]; isolectotypes: P [P00371604, P00371605, P00371606].

#### Description.

Vorontsova and Knapp (2016: 307–312); http://www.solanaceaesource.org/solanaceae/solanum-sisymbriifolium.

#### Distribution.

*Solanumsisymbriifolium* has been collected in tropical Asia in Bangladesh, China, and throughout India (see Saha and Datta 2013); it is native to South America but is widely adventive and somewhat invasive, so it is to be expected throughout the region in highly disturbed areas.

#### Common names.

China. suan jie qie (Zhang et al. 1994). India. mulathurivan [Mikir] (Jain and Borthakur 1986).

#### Discussion.

*Solanumsisymbriifolium* is the only spiny solanum species in tropical Asia with deeply pinnatifid to bipinnatifid leaves coupled with accrescent calyces. *Solanummultiflorum* of the Western Ghats sometimes has deeply pinnatifid leaves, but the calyces are not accrescent and it is a shrub with much smaller flowers (1.3–1.5 cm versus 2–3 cm in diameter). The prickly, accrescent calyx lobes turn back at fruit maturity to reveal the sticky red berries (often cultivated as fruit and called vila-vila in other parts of the world). Some specimens from the Americas have less deeply divided leaves, but all those we have seen from tropical Asia are deeply pinnatifid. Saha and Datta (2013) record it as very common in Tripura.

*Solanumsisymbriifolium* was introduced very early to European botanical gardens, from where it perhaps was introduced as colonisation expanded. It is a very weedy species, even in its native range; overgrazed pastures can become overgrown with *S.sisymbriifolium* very quickly. In some parts of Europe, it is planted in fallow potato fields as a trap crop for cyst nematodes; the nematodes lay eggs in the roots of *S.sisymbriifolium*, but the plants are destroyed before the eggs hatch (Timmermans et al. 2007 a, b).

#### Specimens examined.

See Suppl. materials 1–3.

### ﻿42. Solanum sulawesi

Taxon classificationPlantaeSolanalesSolanaceae

X.Aubriot & S.Knapp sp. nov.

327402FA-311A-504A-90A4-C24EB6DC1AEA

urn:lsid:ipni.org:names:77298805-1

Fig. 69

#### Diagnosis.

Like Solanum*involucratum* Blume, but differing in its lack of dense stellate pubescence on leaf blades and new growth, attenuate leaf bases, and fruiting calyces that are not accrescent.

#### Type.

Indonesia. Sulawesi: Sulawesi Utara, 220 km W. of Manado, 50 km inland from Pangi, on tributary of Sungai Ilanga, 350–750 m, 3 Mar 1990, *J.S. Burley et al. 3625* (holotype: L [L.2882218]; isotypes: A, BO [n.v.], K [K000014635]).

#### Description.

Shrubs to 1.5 m tall, strongly armed. Stems erect, terete, densely prickly, glabrous, purple (fide *Burley et al. 3625*); prickles 0.3–1 cm long, 0.1–0.25 cm in diameter at the base, straight, of many different sizes, pale yellowish tan; pubescence absent; new growth densely prickly and moderately stellate-pubescent with short-stalked porrect-stellate trichomes, the stalks ca. 0.2 mm long, the rays 4–6, ca. 0.5 mm long, the midpoints ca. 0.5 mm long, equal to the rays, the trichomes soon deciduous and the stems glabrate; bark of older stems pale tan. Sympodial units difoliate, the leaves not geminate. Leaves simple, shallowly lobed, the blades (5–)9–17 cm long, (4–)7.5–12 cm wide, 1–1.5 times longer than wide, broadly elliptic, chartaceous, concolorous, strongly armed along the midrib and major veins with straight prickles; adaxial surface glabrate, sparsely pubescent along the veins with scattered sessile porrect-stellate trichomes, the rays 4, 0.1–0.2 mm long, the midpoints 1–1.5 mm long, much longer than the rays; abaxial surface sparsely pubescent with multangulate stalked trichomes, the stalks to 0.5 mm long, the rays 8–10, to 0.5 mm long, irregular in size on a single trichome; major veins 5–6 pairs, densely prickly on both surfaces, the prickles ca. 40 per face, 0.3–1 cm long, straight; base attenuate; margins shallowly lobed, the lobes 5–6 on each side, 1–3 cm long, deltate, acute at the tips and occasionally somewhat bilobed, the sinuses ca. halfway to the midrib; apex acute to acuminate; petioles 2–4 cm long, 1/4–1/3 as long as the leaf blades, densely prickly and sparsely pubescent, the prickles like those of the stems and leaves, the pubescence of scattered stalked porrect-stellate-trichomes, the stalks to 0.5 mm, the rays 6–8, ca. 0.3 mm, the midpoints equalling the rays in length, soon deciduous and the petioles of older leaves glabrate. Inflorescences 0.5–3 cm long, internodal and lateral, unbranched, with 4–8 flowers, only 1 or 2 flowers open at any one time, glabrous except at the very tips, densely prickly, the prickles 2–3 mm long, straight; peduncle absent to 0.9 cm long, densely prickly; pedicels 0.5–0.6 cm long, ca. 0.5 mm in diameter at the base, ca. 1 mm in diameter at the apex, spreading and slightly nodding at anthesis, densely prickly and sparsely stellate-pubescent like the leaf adaxial surfaces, articulated at the base, the trichomes porrect-stellate, the rays 4–8, 0.2–0.5 mm long, midpoints not clearly differentiated from the rays; pedicel scars tightly packed and almost overlapping. Buds broadly ellipsoid and somewhat tapering, included in the calyx lobes until just before anthesis. Flowers 5-merous, apparently all perfect, but some distal flowers may be short-styled. Calyx with the tube 2–2.5 mm long, cup-shaped to slightly urceolate, abruptly narrowing to the pedicel and densely prickly, the lobes 4–6 mm long, ca. 2 mm wide, narrowly deltate, densely prickly on the midveins and sparsely stellate-pubescent abaxially with porrect-stellate trichomes like those of the pedicels. Corolla 1.4–1.8 cm in diameter, white, stellate, lobed 2/3 of the way to the base, interpetalar tissue minimal, the lobes 5–6 mm long, 2.5–3.5 mm wide, narrowly deltate, spreading at anthesis, mostly glabrous adaxially but with a few stellate trichomes at the tips, sparsely stellate-pubescent abaxially with densely tangled sessile trichomes where exposed in bud, these densest at the tips, a few tiny prickles along the petal midvein. Stamens equal; anthers 5–5.5 mm long, ca. 1.5 mm wide, tapering, yellow, densely papillate in the lower half, poricidal at the tips, the pores directed distally, not elongating to slits with drying; filament tube minute, glabrous; free portion of the filaments minute, glabrous, the anthers almost sessile. Ovary conical, densely pubescent with sessile porrect-stellate trichomes with tiny rays and elongate midpoints; style 6–6.5 mm long, glabrous; stigma strongly bilobed, the surfaces minutely papillose. Fruit a globose berry, several per infructescence, 1.5–2 cm in diameter, green (immature?), the pericarp thin and slightly shiny, evenly pubescent with porrect-stellate trichomes with 4–6 minute rays less than 0.1 mm long, the midpoints 3+-celled 1–2.5(–3) mm long; fruiting pedicels 0.8–1.1 cm long, ca. 2 mm in diameter at the base, abruptly enlarged at the apex to ca. 4 mm in diameter, woody, spreading to more or less deflexed from the weight of the berry; fruiting calyx accrescent, the lobes expanding to 7–8 mm long, reflexed and often breaking off. Seeds 50–100 per berry, ca. 2.5 mm long, ca. 2 mm wide, flattened reniform, yellowish tan or brown, the surfaces minutely pitted, the testal cells with straight or slightly sinuate margins. Chromosome number: not known.

**Figure 69.**
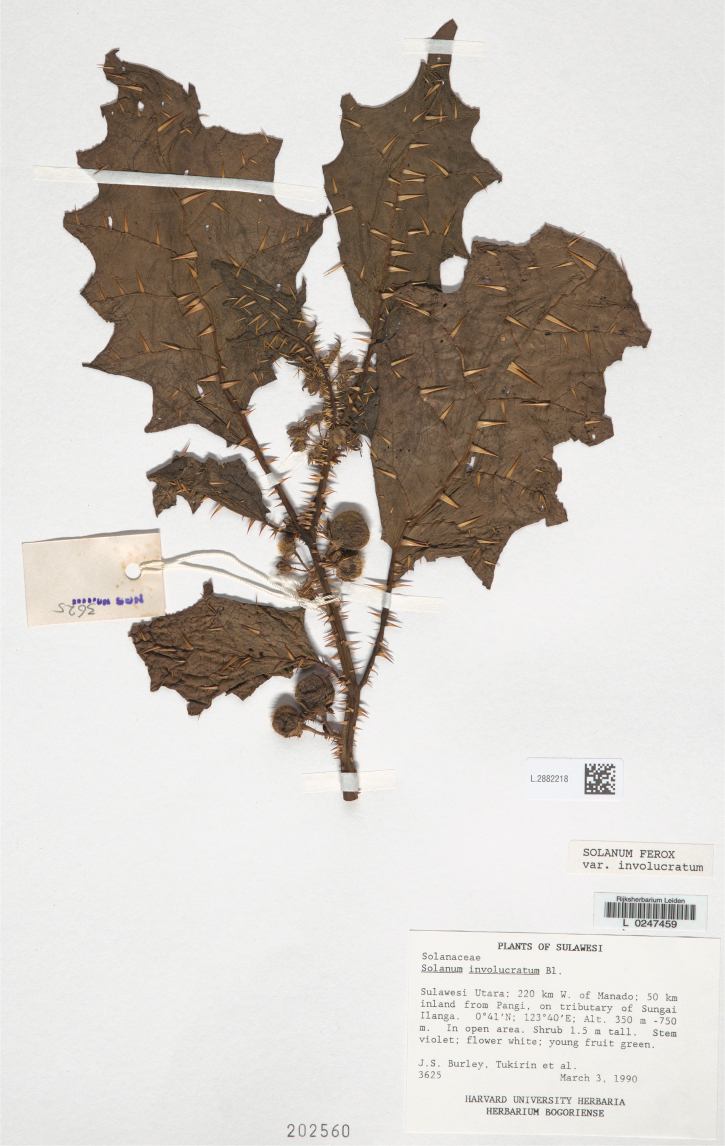
*Solanumsulawesi* X.Aubriot & S.Knapp – Herbarium specimen (holotype) collected in Indonesia in 1990 (*Burley et al. 3625*, L.2882218). Photograph credit: Naturalis Biodiversity Center.

#### Etymology.

*Solanumsulawesi* is named for the island on which it appears to be endemic, Sulawesi, which is of an extraordinary composite geologic nature at the collision zone of the Asian, Pacific, and Australian tectonic plates (Villeneuve et al. 2002).

**Figure 70.**
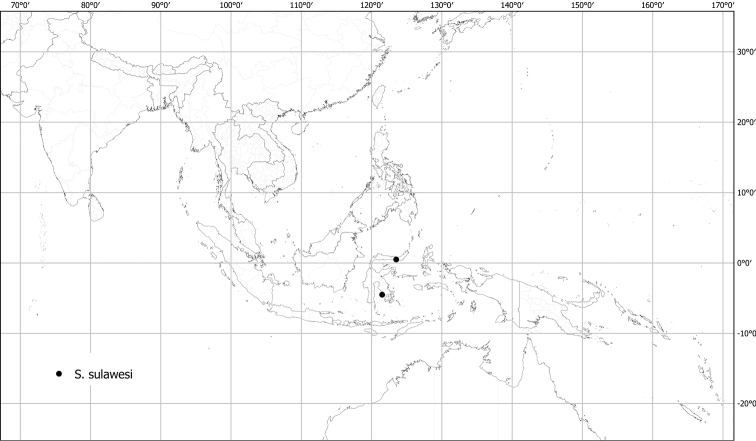
Distribution of *S.sulawesi.*

#### Distribution

**(Fig. 70).***Solanumsulawesi* is endemic to the Indonesian island of Sulawesi (previously known as Celebes under Portuguese colonial occupation); two collections are from the northern peninsula and the third from the southwestern peninsula of the island.

#### Ecology and habitat.

*Solanumsulawesi* has been collected in open areas in lowland tropical rainforest, from 350 to 750 elevation.

#### Common names and uses.

None recorded.

#### Preliminary conservation status

**(IUCN 2019).** Data Deficient (DD). The few collections, coupled with the possibility of more being uncovered as more specimens of *S.involucratum* are examined carefully, suggests that it is too early to assign a preliminary conservation status for this species.

#### Discussion.

*Solanumsulawesi* is morphologically similar to both *S.involucratum* and *S.lasiocarpum*, both of which have large, repand leaves and pubescent berries. It differs from *S.involucratum* in its calyx that is not accrescent in fruit, its glabrous stems and extremely sparsely pubescent leaves that are cuneate to attenuate and widest near the middle (versus truncate and widest near the base). *Solanumlasiocarpum* is a much more densely pubescent plant and is often completely unarmed. The leaves are more ovate in outline and more densely pubescent. The midpoints of the fruit trichomes in *S.sulawesi* are unusual in being 2–3-celled; those of both *S.lasiocarpum* and *S.involucratum* are single-celled. Fruits of *S.sulawesi* are smaller than those of S. *lasiocarpum*, and less densely pubescent.

We would have preferred to place the holotype for this species in a herbarium in Indonesia, but extensive searches in the herbarium at Bogor (BO; A. Kartonegoro, pers. comm.) failed to locate a duplicate of *Burley et al. 3625*, although one should be located there.

Material of *S.sulawesi* has not been included in molecular analyses but we suspect it will prove a member of a clade comprised of southeast Asian species and not a member of the largely American Lasiocarpa clade.

We have seen very few collections of *S.sulawesi*, but specimens of this species may have been previously identified as *S.involucratum* or *S.lasiocarpum.* These and unidentified collections in BO and other Indonesian herbaria are of particular interest and are a priority for examination.

#### Paratypes.

[also see Suppl. materials 1–3] **Indonesia. Sulawesi**: Sulawesi Tenggara, Southeast Celebes. Ladongi - Tirawuta - Kolaka [Kolaka Timur], 19 Oct 1978, *Prawiroatmodjo & Maskuri 1356* (K, L); Sulawesi Utara, “Ins. Celebes: (Minahassa): N Celebes”, 1888, *Warburg 15844* (NY).

### ﻿43. Solanum torvoideum

Taxon classificationPlantaeSolanalesSolanaceae

Merr. & L.M.Perry, J. Arnold Arb. 30: 47. 1949.

B9FE9FDE-15DE-52A0-8DB6-9DCF7E4F1CB1

Fig. 71

#### Type.

Papua New Guinea. Central: Mafulu, Sep-Nov 1933, *L.J. Brass 5411* (lectotype designated by Symon 1985, pg. 150, as “holotype”: A [00077838]; isolectotypes: BM [BM000886267], BRI [BRI-AQ0080383], L [L.0003661], NY [00172294]).

#### Description.

Shrubs to small trees, 1–5 m tall, unarmed or rarely sparsely armed. Stems erect, terete, usually unarmed or with a few broad-based straight prickles, stellate-pubescent; prickles, if present, 2–3 mm long, to 1.5 mm wide at the base, very sparse, straight, pale yellowish tan; pubescence of sessile to very short-stalked porrect-stellate trichomes, the stalks to 1 mm long, the rays 6–8, ca. 0.5 mm long, the midpoints absent or to 1 mm long (New Guinea only); new growth densely stellate-pubescent, the trichomes tangled, somewhat golden in dry material; bark of older stems grey to dark brown, glabrescent. Sympodial units difoliate, the leaves geminate, the leaves of pair equal in size and shape or one leaf slightly smaller. Leaves simple, shallowly lobed, the blades (7–)12–25 cm long, (2–)3–11 cm wide, 2.5–4 times longer than wide, elliptic to narrowly elliptic, chartaceous, discolorous, unarmed or sparsely armed along the midrib and major veins with small straight prickles; adaxial surface dark green, evenly and moderately to densely pubescent with golden short-stalked porrect-stellate trichomes, the stalks to 0.5 mm long, the rays 4–6, ca. 0.5 mm long, the midpoints absent or to 0.5 mm (in some New Guinea populations the midpoints glandular); abaxial surface with similar short-stalked porrect-stellate trichomes, but with more numerous rays (up to 10–12); major veins 4–7 pairs, densely pubescent especially abaxially; base acute, somewhat oblique; margins shallowly lobed, the lobes 4–7, 0.5–3 cm long, deltate to triangular, apically acute, the sinuses ca. one third of the way to the midrib; apex acute; petioles 1.5–3(–4+) cm long, ca. 1/4 as long as the leaf blades, sparsely prickly and densely to moderately pubescent with short-stalked stellate-trichomes like those of the stems, unarmed or prickly with 1–3 prickles like those of the stems. Inflorescences 4–9 cm long, internodal and lateral, forked to several times branched, with 50–70+ flowers, several flowers open at any one time, densely pubescent with short-stalked stellate-porrect trichomes like those of the stems, with 6–8 rays ca. 0.5 mm long and midpoints to 1 mm long, unarmed; peduncle absent and the first inflorescence branches appearing to arise directly from the stem, or 0.5 cm long, unarmed; pedicels 1–1.2 cm long, ca. 1 mm in diameter at the base and apex, spreading to erect at anthesis, densely stellate-pubescent like the inflorescence axes, articulated at the base, unarmed; pedicel scars irregularly spaced 1–2 mm apart. Buds elongate ellipsoid and somewhat tapering, strongly exserted from the calyx before anthesis. Flowers 5-merous, apparently all perfect, but some distal flowers may be short-styled. Calyx with the tube 2–3 mm long, conical, the lobes 2–5 mm long, 1–2 mm wide, deltate with a subulate acumen 1.5–2 mm long, densely stellate-pubescent with porrect-stellate trichomes like those of the pedicels, the acumen sometimes more glabrous and drying dark. Corolla 2–2.6 cm in diameter, white, shallowly stellate, lobed ca. halfway to the base, interpetalar tissue present and abundant, the lobes 8–10 mm long, 4–7 mm wide, spreading at anthesis, mostly glabrous adaxially but with a few stellate trichomes at the tips, densely stellate-pubescent abaxially with densely tangled sessile trichomes where exposed in bud, these densest at the tips, the interpetalar tissue glabrous. Stamens equal; anthers 5–6.5 mm long, ca. 1 mm wide, connivent or somewhat spreading, tapering, yellow, glabrous, poricidal at the tips, the pores directed distally, not elongating to slits with drying; filament tube minute, glabrous; free portion of the filaments 0.5–1 mm long, glabrous. Ovary conical, glabrous; style 6–9 mm long, glabrous; stigma capitate, the surfaces minutely papillose. Fruit a globose berry, several to many per infructescence, 1–1.4 cm in diameter, the pericarp thin and shiny, yellow to orange-yellow when ripe, aging to brown or almost black (fide Symon 1985), glabrous; fruiting pedicels 1.5–2 cm long, ca. 1.5 mm in diameter at the base, 3–3.5 mm in diameter at the apex, woody, erect or somewhat spreading from weight of berries; fruiting calyx not accrescent, the lobes often breaking off. Seeds 60–100 per berry, 2–3.5 mm long, ca. 2 mm wide, flattened reniform, pale tan or yellowish brown, the surfaces minutely pitted, the testal cells with sinuate margins. Chromosome number: n = 12 (Symon 1985; voucher *Symon 10669* from Papua New Guinea).

**Figure 71.**
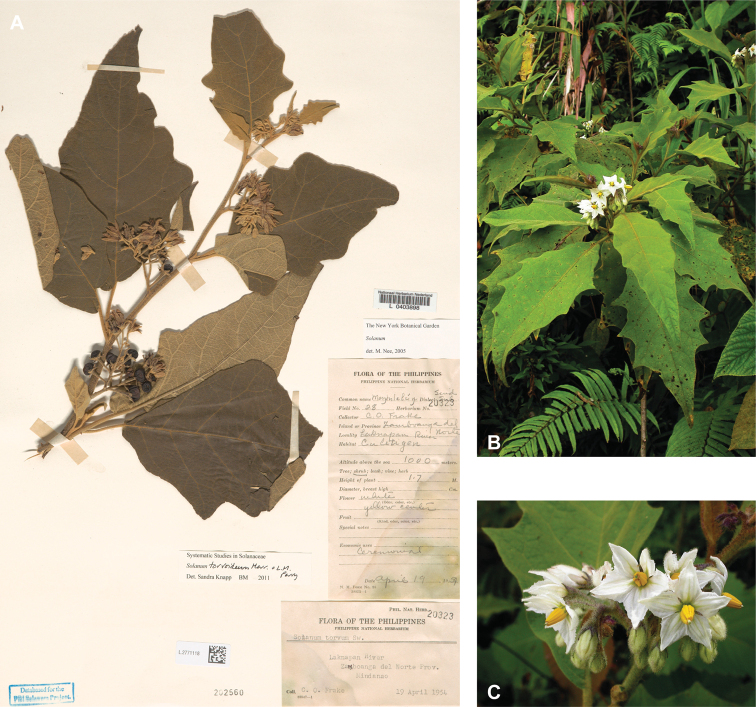
*Solanumtorvoideum* Merr. & L.M.Perry **A** herbarium specimen collected in the Philippines in 1954 (*Frake 20323*, L.2771110) **B** habit (field photograph, unvouchered, Philippines) **C** detail of the inflorescence (field photograph, unvouchered, Philippines). Photograph credits: **A** Naturalis Biodiversity Center **B, C** P.B. Pelser.

#### Distribution

**Figure 72.**
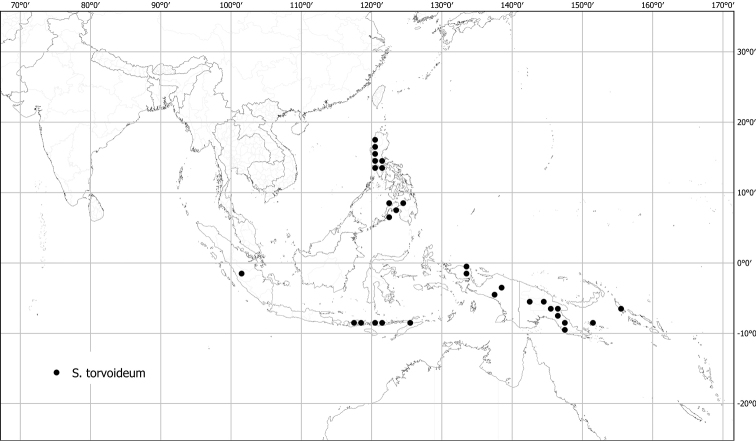
Distribution of *S.torvoideum*.

**(Fig. 72).***Solanumtorvoideum* occurs from the Sunda Islands in Indonesia and Timor Leste east to the Philippines and the island of New Guinea.

#### Ecology and habitat.

*Solanumtorvoideum* is a species of disturbed areas such as old garden sites, treefalls or roadsides; from 300 to 2,000 m elevation (higher elevations from New Guinea specimens). Symon (1985) records it as occurring in fagaceous forest and on gravelly stream beds on New Guinea.

#### Common names and uses.

Philippines. Mindanao: bokul [Bukid] (*Sulit 9868*), tangutaungan (*Zwickey 52*), gebubruca (*Frake 38012*); Luzon: tanutong (*Maliwanang 229*).

#### Preliminary conservation status

**(IUCN 2019).** Least Concern (LC). EOO (2,372,285 km^2^, LC); AOO (212 km^2^, EN). As a specialist of disturbed areas with a wide distribution when the island of New Guinea is taken into account, *S.torvoideum* is not of conservation concern.

#### Discussion.

*Solanumtorvoideum* is a member of the Torva clade (see Aubriot et al. 2016a) and like others in the group has difoliate sympodia with usually geminate leaves, highly branched inflorescences, straight or only slightly curved stem prickles, and sticky leathery berries. The Asian species of “torvoids” differ from their American relatives in having red, rather than green berries at maturity. In the area treated here *S.torvoideum* is most similar to the introduced and widespread *S.torvum*, from which it differs in its denser, more reddish gold pubescence, eglandular inflorescences, and red berries. In general, plants of *S.torvoideum* are more robust-looking than those of *S.torvum*. *Solanumtorvoideum* is superficially similar to another introduced species, S. *chrysotrichum* in its pubescence, but is not sympatric with it, and can be distinguished by fruit colour. It can be distinguished from *S.poka* and *S.pseudosaponaceum* in its more branched inflorescences with denser pubescence and larger flowers (to 2.6 cm in diameter versus to 2 cm or 1.5 cm in diameter).

On the island of New Guinea, *S.torvoideum* is most similar to the sympatric *S.dammerianum* Lauterb. & Schum. (Symon 1985). *Solanumtorvoideum* differs from *S.dammerianum* in is rusty (versus drab) leaf pubescence, its more compact and fewer branched inflorescence and its larger mature berries (to 2 cm in diameter versus to 1.5 cm in diameter). The type of *S.dammerianum* has been lost (Symon 1985) so further intensive study of New Guinea populations may reveal additional differences.

The Torva clade species occurring in tropical Asia are all each other’s closest relatives, suggesting a single dispersal event from the Americas (Aubriot et al. 2016a).

#### Specimens examined.

See Suppl. materials 1–3.

### ﻿44. Solanum torvum

Taxon classificationPlantaeSolanalesSolanaceae

Sw., Prodr. [O.P. Swartz] 47. 1788.

76599CF7-8079-58D5-84EC-FF05AD234DD0

**Figure 73.**
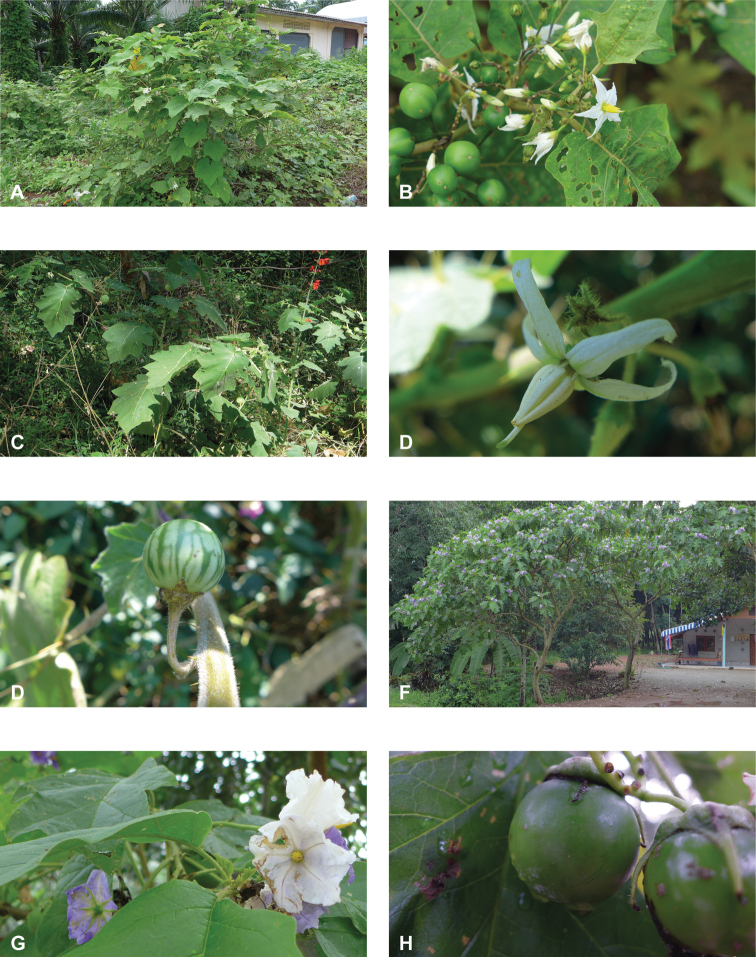
Introduced species of *Solanum.Solanumtorvum* Sw. **A** habit (*Meeboonya et al. RM 267*, Thailand) **B** detail view of an inflorescence with flowers and fruits (*Meeboonya et al. RM 267*, Thailand). *Solanumviarum* Dunal **C** habit (*Sampath Kumar et al. 126976*, India) **D** detail view of a flower (*Sampath Kumar et al. 126923*, India) **E** detail view of a fruit (*Sampath Kumar et al. 126923*, India). *Solanumwrightii* Benth. **F** habit (*Meeboonya et al. RM 271*, Thailand) **G** detail view of flowers (*Meeboonya et al. RM 271*, Thailand) **H** detail view of fruits (*Meeboonya et al. RM 271*, Thailand). Photograph credits: X. Aubriot.

Figs 3G , 73A, B Names based on tropical Asian types only; for complete synonymy see Vorontsova and Knapp 2016.

Solanum macaoense Dunal, Prodr. [A.P. de Candolle] 13(1): 264. 1852. Type. China. Guangdong: “Macao”, *S. Callery 28/29* (holotype: P [P00055495]).

#### Type.

“Indiae occidentalis” [“Provenit in sepibus Jamaicae, Hispaniolae, Insulis Bermudensibus” Swartz 1797: 456], *O. Swartz s.n.* (lectotype, designated by Vorontsova and Knapp 2016, pg. 351: S [S-R-5814]).

#### Description.

Vorontsova and Knapp (2016: 351–355); http://www.solanaceaesource.org/solanaceae/solanum-torvum-1.

#### Distribution.

*Solanumtorvum* is naturalised throughout tropical Asia; it is native to Central America and the Caribbean but has achieved a worldwide distribution in tropical and subtropical areas.

#### Common names.

Bangladesh. tith begum (*Rahman’s collector 1124*). Brunei Darussalam. terong pipit (*Norul Rozimah Pg Hj Seruji 18-b-3000*); tarong cit, tarong lowow (*Yati 412*). Cambodia. trâp put num nhong, trâp rom nhong (Hul and Dy Phon 2014). China. shui qie (Zhang et al. 1994). India. Assam: hati bhekur (*Marriot 86*); Bihar: jangali baigan (Varma 1981); Chhattisgarh: ban bhata, kutmi (*Mooney 120*); Kerala: anachunda (Mohanan and Henry 1994); Maharashtra: adhvi badnae [Kanara] (*Fernandes 1377*), ran vanghae (Marathi, *Fernandes 1377*); Odisha: tutugana (*Mooney 688*); Rajasthan: bhurat [Hindi] (Singh 1991); Tamil Nadu: sundai [Tamil] (Matthew 1983); sundaikai [Tamil] (Henry et al. 1987); sundaikkaai (*Sampath Kumar et al. 126940*). Indonesia. Borneo: terung pipit (*Wiriadianata 1196*); Sumatra: tĕrong pipit (Burkill 1935). Laos. kh’èngz do:n, kh’èngz hna:m hlwàng, kh’èngz khâm, kh’èngz saph’ao (Hul and Dy Phon 2014). Malaysia. Malacca: trong naya bang (*Anonymous 418*); terong manggor (*Anonymous s.n.* K001153289). Malaysia/Singapore. tĕrong pipit puteh, tĕrong rembang (Burkill 1935, but care to be taken with these names, they include other Torva clade taxa). Mauritius. Bringelle marron (*Drennen 24*). Sri Lanka. gona batu [Sinhala] (*Hepper & Silva 4675, Hepper 4411, Hepper 4415*); tibatu [Sinhala] (*Veldkamp 7852*). Thailand. weng (*387*); sambalan (*Hoed & Kostermans 962*). Vietnam. cà cõ, cà rùng, cà gai, cà pháo, trong phang, trong phet (Hul and Dy Phon 2014).

#### Discussion.

*Solanumtorvum* is by far the most commonly collected spiny solanum in tropical Asia; most recent collections sent to us for identification are this species. It may be becoming more common with anthropogenic change or with spread due to human use. *Solanumtorvum* is ubiquitous and fully naturalised in the region and has become culturally important in many countries (see multiplicity of common names above). The fruits are used in cooking in China and Indochina; they are exported to Europe under the name pea eggplant. Hul and Dy Phon (2014) record the use of seeds in traditional medicine in Indochina, and in India extracts of the plant are used for relief from bites of venomous insects or snakes. In Bhutan the roots and leaves are used medicinally and the fruits are eaten (Mill 2001).

Vorontsova and Knapp (2016) incorrectly placed *S.pseudosaponaceum* and its synonym *S.macaoense* in the synonymy of *S.torvum*, our examination of more extensive material from tropical Asia clearly shows that *S.pseudosaponaceum* is a distinct taxon. *Solanumtorvum* is a member of the Torvum clade and can be distinguished from all other members of that group in Asia (*S.chrysotrichum*, *S.comitis*, *S.kachinense*, *S.peikuoense*, *S.poka*, *S.pseudosaponaceum*, *S.torvoideum*) by its densely glandular inflorescences with tiny simple glandular trichomes, in addition to the stellate trichomes found in the rest of the plant; the rest of the plant is eglandular.

#### Specimens examined.

See Suppl. materials 1–3.

### ﻿45. Solanum trilobatum

Taxon classificationPlantaeSolanalesSolanaceae

L., Sp. Pl. 188. 1753.

EA6CD4AB-484C-5CD2-9962-3DFE2F8BEF68

**Figure 74.**
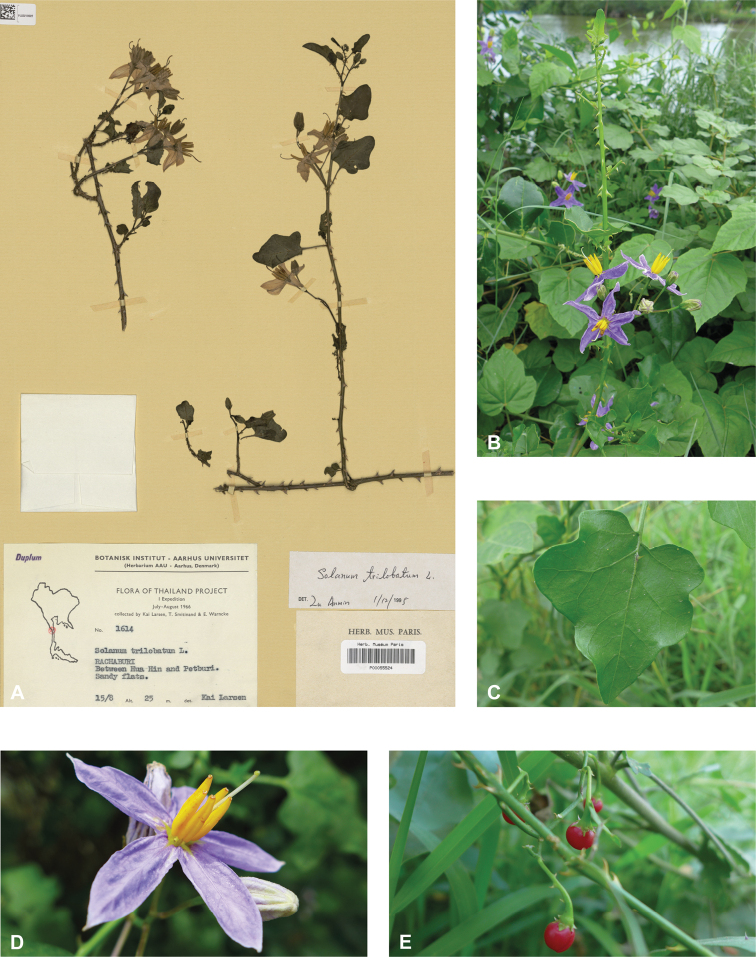
*Solanumtrilobatum* L. **A** herbarium specimen collected in Thailand in 1966 (*Larsen 1614*, P00055524) **B** habit (*Meeboonya et al. RM 243*, Thailand) **C** detail of the adaxial surface of a leaf blade (*Meeboonya et al. RM 301*, Thailand) **D** detail view of an open flower (*Meeboonya et al. RM 245*, Thailand) **E** detail view of an infructescence (field photograph, unvouchered, India). Photograph credits: **A** CC-BY, Muséum national d’Histoire naturelle, Paris **B–E** X. Aubriot.

Figs 1B , 2F , 4F , 74

Solanum acetosifolium Lam., Tabl. Encycl. 2: 24. 1794, as “*acetosaefolium*”. Type. India. Sin loc., *P. Sonnerat s.n.* (lectotype, designated here: P [P00055514]).
Solanum canaranum Miq., Hohen. Pl. Ind. Or. (Terr. Canara & Confin.) Exsicc. No. 740. 1847. Type. India. Karnakata: “prope Bettighery”, Feb., *R.F. Hohenacker 740* (lectotype, designated here: U [U.1740185]; isolectotypes: BM [BM000900314], K [K000014877]).
Solanum trilobatum L. var. griffithii C.B.Clarke, Fl. Brit. India [J. D. Hooker] 4: 237. 1883. Type. “Birma and Malay Peninsula”, *W. Griffith 5915* (lectotype, designated here: K [K000196631]).
Solanum griffithii (C.B.Clarke) Kuntze, Revis. Gen. Pl. 2: 454. 1891. Type. Based on SolanumtrilobatumL.var.griffithii C.B.Clarke

#### Type.

“Habitat in India”, *Anonymous s.n.* (lectotype, designated by Deb 1980, pg. 51: LINN [acc. # 248.44]).

#### Description.

Scandent herbs or shrubs, to 2.5 m tall, armed. Stems erect or spreading, terete, moderately to densely prickly, glabrous or with a few scattered stellate trichomes; prickles to 6 mm long, to 5 mm wide at the base, deltate, markedly hooked, laterally flattened, pale yellow to brown, glabrous; pubescence of sessile to very short-stalked porrect-stellate trichomes, the rays 2–5, 0.1–0.25 mm long, the midpoints absent or to 0.25 mm long; new growth sparsely stellate-pubescent, glabrescent, light yellowish green in dry material; bark of older stems brownish, glabrous. Sympodial units apparently difoliate, the leaves geminate, usually similar in size. Leaves simple, more or less deeply lobed, the blades 2.5–6.5 cm long, 2–7.5 cm wide, ca. 1–1.5 times longer than wide, ovate to broadly ovate, somewhat fleshy, slightly discolorous, unarmed or with a few hooked prickles like those of the stems, per face; adaxial surface green, glabrous or with a few scattered porrect-stellate trichomes, the trichomes sessile to sub sessile, the rays 4–8, 0.1–0.25 mm long, the midpoints to 0.1 mm long; abaxial surface light-green, glabrescent, sometimes with a few (i.e., < 10) porrect-stellate trichomes like those of the adaxial surface; major veins 3–5 pairs drying brown; base truncate to shortly attenuate; margins shallowly to deeply lobed, the lobes 1–2 on each side, 0.5–0.7 cm long, deltate, apically rounded, the sinuses extending up to 3/5 of the distance to the midvein; apex rounded; petioles 1.2–3 cm long, 1/2–2/3 of the leaf blade length, glabrescent, unarmed or with a few prickles like those of the stems. Inflorescences 1–6 cm long, apparently lateral, unbranched or forked, with ca. 3–11 flowers, only a few flowers open at any one time, glabrescent, unarmed; peduncle ca. 0.5 cm long, unarmed or a few prickles; pedicels 0.8–1.5 cm long, 0.8–1 mm in diameter at the base, 1.2–1.5 mm in diameter at the apex, erect, unarmed or with a few hooked prickles especially on the lower flowers, glabrescent, articulated at the base; pedicel scars spaced 0.4–1 cm apart. Buds ellipsoid, the corolla strongly exserted from the calyx before anthesis. Flowers 5-merous, apparently all perfect. Calyx with the tube 1.5–2 mm long, cup-shaped and somewhat keeled, the lobes 1–2 mm long, ca. 1 mm wide, narrowly deltate, apically acute, unarmed or with a few hooked to straight prickles, glabrous to sparsely stellate-pubescent with sessile porrect-stellate trichomes like those of the stems. Corolla 2–3 cm in diameter, purplish blue, stellate, lobed ca. 3/5–4/5 of the way to the base, the lobes 8–12 mm long, 2.5–7 mm wide, deltate to narrowly deltate, spreading at anthesis, sparsely to moderately stellate-pubescent abaxially on parts exposed in bud. Stamens equal; anthers 7–9 mm long, 1–1.5 mm wide, connivent, not tapering at anthesis, yellow, glabrous, poricidal at the tips, the pores directed distally, not elongating to slits with drying. Ovary globose, with minute glandular hairs; style 1.4–1.8 cm long, slender, curved at the apex, glabrous; stigma capitate, minutely papillate. Fruit a globose berry, 1 to several per infructescence, 0.7–1.2(–1.7) cm in diameter, red at maturity, the pericarp thin and shiny, glabrous; fruiting pedicels 1.2–2.6 cm long, ca. 1 mm in diameter at the base, 2–2.5 mm in diameter at the apex, erect to recurved, not markedly woody; fruiting calyx lobes slightly expanding to ca. 3 mm long, 1/5–1/3 the length of the mature fruit, deltate, not reflexed. Seeds ca. 16–47 per berry, 3–4 mm long, 2.5–3 mm wide, flattened-reniform, dull yellow, the surface minutely pitted, the testal cells pentagonal in outline. Chromosome number: n = 12, 2n = 24 (Madhavadian 1968).

#### Distribution

**Figure 75.**
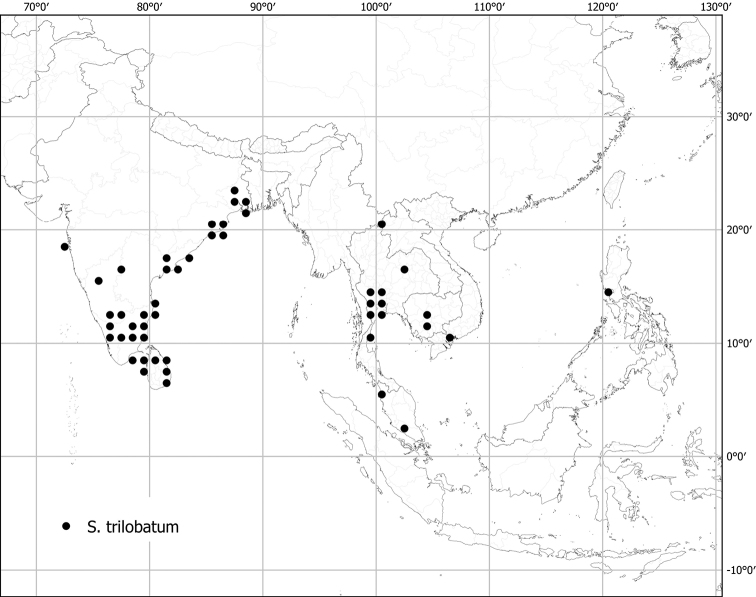
Distribution of *S.trilobatum*.

**(Fig. 75).***Solanumtrilobatum* is widely distributed in tropical Asia; from South India and Sri Lanka to Indochina, and Java. The single collection from the Philippines (*Loher s.n.*) is from the railway station in Manila and is probably cultivated.

#### Ecology and habitat.

*Solanumtrilobatum* grows in open areas or forest edges, on sand or limestone, close to coastal areas; often in open field or mangrove forest; from around sea level to 700 m elevation.

#### Common names and uses.

Cambodia. traob (Khmer, *Hul 4774*); India. Andhra Pradesh: uchinta (*Venkanna 5562*), usti (*Raji 291*); Karnataka: ullul (Singh 1988); Odisha: ankaranti (*Panigrahi EC-23792*); Tamil Nadu: toundou valay [Tamul] (*Commerson s.n.*), thoothuvalai [Tamul] (*Matthew 8323*), tudulai [Tamul] (*Mehen-Homji s.n.*), thoodhuvalai [Tamul] (*Narasimhan 624*, *Sampath Kumar et al. SC-126970*), thudhuvaelai [Tamul] (Matthew 1983), tuduvali [Tamul] (Henry et al. 1987); West Bengal: kantikuri (*Tribedi CNH-1435*); Malaysia. priapantie (*Curtis 858*).

It is reported that in Thailand the plant is boiled to serve as a decoction for ulcerated throat (*Collins 1380*). Indian collectors reported that the fruits are edible and used as medicinal (*Raji 291*, *Sengupta 145*). In Penang (Malaysia) the root is used medicinally (*Curtis 858*). For the many uses in traditional medicine in India see section on Uses (pg. 18–20).

#### Preliminary conservation status

**(IUCN 2019).** Least Concern (LC). EOO (2,326,480 km^2^, LC); AOO (528 km^2^, VU). Widely distributed and likely to be protected by people as a plant of considerable medicinal importance, *S.trilobatum* is not of immediate conservation concern. Methods of collection for medicinal use, however, have not been assessed and if these are destructive this could be cause for concern.

#### Discussion.

*Solanumtrilobatum* is widely distributed through India and Indochina. It was one of the first Asian spiny solanums to be described (Linnaeus 1753) and was brought into cultivation in European botanical gardens in the late 18^th^ century. It is a distinctive species with its rhomboidal to triangular leaves that are usually widest in the lower third, but has been confused with *S.procumbens*, a similarly scandent plant. Flowers of *S.trilobatum* are much larger than those of *S.procumbens* (2–3 cm in diameter versus 1–1.5 cm in diameter), with abundant interpetalar tissue; *S.procumbens* flowers are deeply stellate with narrow corolla lobes. The two species do not overlap in distribution.

No specimen labelled as *S.acetosifolium* has been found in the Lamarck herbarium (P-LAM), however in the French language column Lamarck (1794) cited “Indes Orient, Sonnerat” clearly indicating a collector. We have lectotypified *S.acetosifolium* with a specimen collected by Sonnerat labelled “Inde” in the general collection at Paris (P00055514).

In describing S.trilobatumvar.griffithii, Clarke (1883) cited only “Griffith 5915”; we have selected the single specimen thus labelled (K000196631) as the lectotype.

The name *S.canaranum* was effectively published by being printed on herbarium labels that were distributed to herbaria before 1953 (Turland et al. 2018, Art. 30.8); the name is also accompanied by a diagnosis attributed to Miquel. We have chosen the duplicate in Utrecht [U.1740185], where Friedrich Miquel worked (Stafleu 1966), as the lectotype for this name.

#### Specimens examined.

See Suppl. materials 1–3.

### ﻿46. Solanum vagum

Taxon classificationPlantaeSolanalesSolanaceae

Nees, Trans. Linn. Soc. London 17(1): 48 1834.

C7E74EC3-4875-57A6-92B2-27E55DF41CAF

Fig. 76

#### Type.

India. Sin. loc., “S. corymbosum Pers.?, hb. Wight”, *Herb. Wight s.n.* (Wallich cat. 2624b) (lectotype, designated here: GZU [GZU000255872]; isolectotypes: K [K001152841], K-W [K001116639]).

#### Description.

Erect shrubs 0.5–2.5 m tall, unarmed. Stems erect, terete, sparsely to moderately stellate-pubescent, soon glabrescent; pubescence of sessile to short-stalked porrect-stellate to somewhat multangulate trichomes, the stalks 0.1–0.2 mm long, the rays 4–12, 0.1–0.2 mm long, the midpoints shorter than or as long as the rays; new growth densely stellate-pubescent, the trichomes porrect-stellate, sessile or short-stalked, the stalks 0.1–0.2 mm long, the rays 6–8, 0.2–0.5 mm long, the midpoints equal to the rays; bark of older stems glabrescent, usually with only a few scattered stellate trichomes, greyish brown and somewhat shiny. Sympodial units plurifoliate, the leaves not geminate. Leaves simple, entire, the blades 5–15 cm long, 1.5–7 cm wide, lanceolate to narrowly elliptic to elliptic, membranous, discolorous; adaxial surface drying dark, glabrous or with a few scattered sessile porrect-stellate trichomes on the veins and lamina, the trichomes more delicate than those of the abaxial surfaces, with 4–8 rays 0.1–0.2 mm long and midpoints equal to the rays; abaxial surface paler, almost glabrous to moderately pubescent, the trichomes sessile or very short-stalked, the rays 4–8, 0.3–0.5 mm long, the midpoints equal to the rays; major veins 6–8 pairs, prominent and drying yellowish or darker than the lamina abaxially; base acute, somewhat oblique, but not markedly so; margins entire or slightly sinuate and irregularly lobed; apex acute to somewhat acuminate; petiole 2–6 cm long, 1/2 or more of the leaf blade length, glabrous to sparsely stellate-pubescent with porrect-stellate stellate trichomes like the stems. Inflorescences 3–8 cm long, apparently terminal or lateral, usually unbranched but occasional forked, with 4–10 flowers, 1–2 flowers open at any one time, glabrous to very sparsely pubescent with sessile porrect-stellate trichomes like those of the stems; peduncle 1–3 cm long; pedicels 1–1.2 cm long, 1–1.2 mm in diameter at the base, 1.5–1.8 mm in diameter at the apex, erect at anthesis, glabrous or sparsely stellate-pubescent like the inflorescence axes, articulated at the base or just above and leaving a minute peg on the inflorescence axis; pedicel scars spaced 1–3 mm apart. Buds ovoid, not markedly tapering, densely stellate-pubescent, strongly exerted from the calyx tube before anthesis, but the calyx acumens nearly to the tips of the buds. Flowers 5-merous, apparently all perfect. Calyx with the tube 1.5–2 mm long, conical, the lobes 1–2 mm long, 1–1.2 mm wide, narrowly deltate with a long subulate acumen to 2.5 mm long, glabrous or very sparsely pubescent abaxially with sessile porrect-stellate trichomes like those of the pedicels. Corolla 2–3 cm in diameter, white, stellate, lobed nearly to the base, the lobes 8–10 mm long, 2.5–3 mm wide, narrowly deltate, spreading at anthesis, glabrous adaxially, moderately pubescent abaxially with sessile stellate porrect trichomes, the rays 3–10, ca. 0.1 mm long, the midpoints equal to the rays, the tips somewhat cucullate. Stamens equal in bud, unequal at anthesis, with 4 short and one long and curved; long anther 7–9 mm long and strongly curved upwards, 1–1.5 mm wide, the short anthers 3–6 mm long, 1–1.5 mm wide, elliptic but the longer one somewhat tapering, all yellow, glabrous, poricidal at the tips, the pores directed distally and not elongating with age; filament tube minute, glabrous; free portion of all filaments ca. 0.5 mm long, glabrous; Ovary conical, glabrous or with a few porrect-stellate trichomes at the apex; style 9–11 mm long, slender, strongly curved in same direction as the long anther, glabrous or with a few porrect-stellate trichomes at the very base; stigma capitate, minutely papillate. Fruit a globose berry, 2–4 per infructescence, 0.8–1 cm in diameter, red at maturity, the pericarp thin and shiny, glabrous; fruiting pedicels 1.5–1.8 cm long, ca. 1 mm in diameter at the base, 2–2.5 mm in diameter at the apex, woody and erect; fruiting calyx lobes not markedly accrescent, splitting to the base and expanding to 4–6 mm long including the acumen which often breaks off in dry specimens. Seeds 10–20 per berry, 4–4.5 mm long, 3–4 mm wide, flattened-reniform, pale tan, the surfaces smooth in the seed centre, minutely pitted on the thickened margins, the testal cells somewhat sinuate in outline. Chromosome number; n = 12 (Madhavadian 1968).

**Figure 76.**
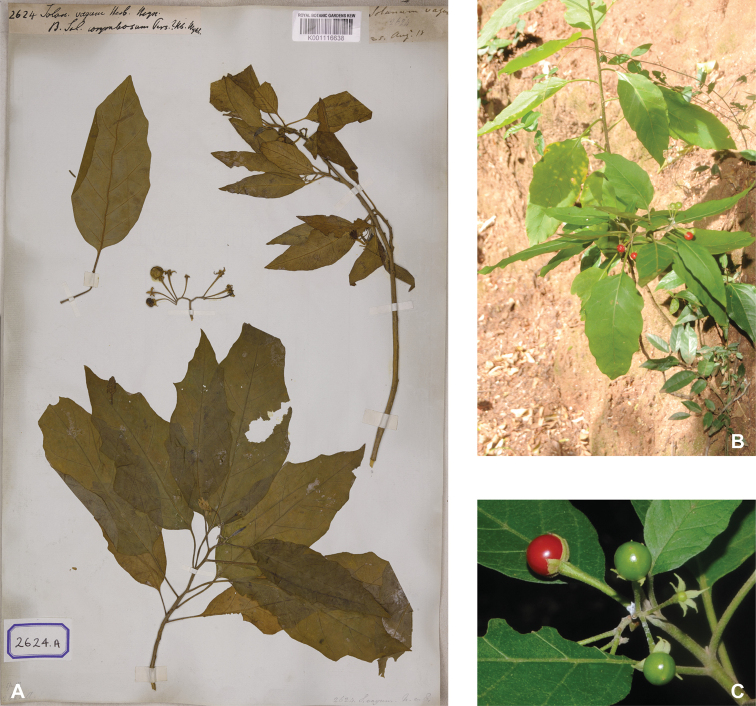
*Solanumvagum* B.Heyne ex Nees **A** herbarium specimen collected in India in 1818 (*Herb. Wight s.n.* (Wallich cat. 2624A), K001116638) **B** habit (field photograph, unvouchered, India) **C** detail view of an infructescence (field photograph, unvouchered, India). Photograph credits: **A** © copyright of the Board of Trustees of the Royal Botanic Gardens, Kew **B, C** R. Vellingiri.

**Figure 77.**
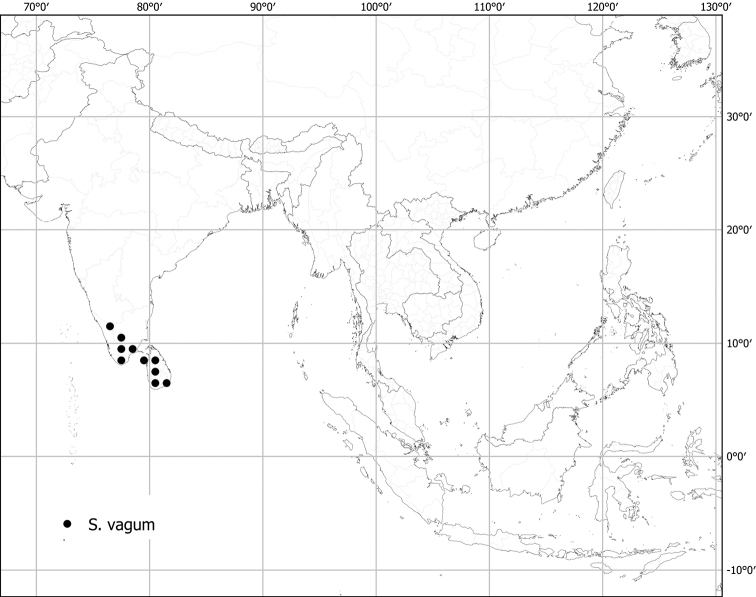
Distribution of *S.vagum*.

#### Distribution

**(Fig. 77).***Solanumvagum* occurs in southern India (states of Kerala and Tamil Nadu) and in Sri Lanka.

#### Ecology and habitat.

*Solanumvagum* grows in dry open woodland, degraded vegetation, grasslands and open areas; occurring from sea level to 750 m elevation.

#### Common names and uses.

None recorded.

#### Preliminary conservation status

**(IUCN 2019).** Least Concern (LC). EOO (75,338 km^2^, LC); AOO (76 km^2^, EN). *Solanumvagum* is found both in southern India and Sri Lanka, and where it occurs it is a plant of open, disturbed areas, suggesting that it is not of immediate conservation concern.

#### Discussion.

*Solanumvagum* is morphologically similar and probably closely related to *S.pubescens*, sharing with that species zygomorphic flowers at anthesis, heteromorphic anthers and shiny berries on erect or slightly pendulous pedicels. Hepper (1987) treated material of *S.vagum* from Sri Lanka as *S.pubescens* in the “Revised Handbook to the Flora of Ceylon”. It differs from *S.pubescens* in its complete lack of glandular pubescence, its narrowly elliptic leaves with attenuate bases and slightly larger, white (rather than violet) flowers. The pubescence of adaxial leaf surfaces of *S.vagum* is of very sparse sessile stellate trichomes with midpoints usually equal to the rays, and the lamina is clearly visible, while in *S.pubescens* the lamina of young leaves is obscured by the dense covering of glandular stellate to multangulate stalked trichomes with elongate midpoints. The two species apparently co-occur in southern India, while all specimens we have seen identified as *S.pubescens* from Sri Lanka are referable to *S.vagum*.

The long anther in the flowers of *S.vagum* appears to expand after anthesis; specimens with buds or very young flowers have the anthers all of equal size, while older flowers have one anther markedly longer than the rest. Post-anthesis anther expansion occurs in the unrelated *S.turneroides* Chodat (Brevantherum clade, see Stern et al. 2013) of southern South America. Buds of *S.pubescens* and the African species *S.somalense* Franch., which greatly resemble those of *S.vagum*, also have the anthers of more or less equal length and buds that are not markedly curved.

*Solanumvagum* is probably a member of the Giganteum clade (sensu Vorontsova et al. 2013; Aubriot et al. 2016a) based on similarity with *S.pubescens* and the African/Middle Eastern species *S.somalense*. These taxa can be distinguished from *S.giganteum*, the only other member of the group occurring in tropical Asia, by their zygomorphic flowers and calyx lobes with long subulate tips that are especially visible in bud.

The species has been indicated as endemic to the southern part of the Western Ghats biodiversity hotspot (https://indiabiodiversity.org/biodiv/species/show/264048) but also occurs in Sri Lanka.

Nees van Esenbeck (1834) validated the herbarium name previously listed as Wallich cat. 2624 citing two collections; “Solanumvagum. Herb. Heyn., Wall. l.c.” (Wallich cat. 2624a) and “Solanum corymbosum? Herb. Wight” (Wallich cat. 2624b). We have selected the specimen at GZU that comes from Nees van Esenbeck’s own herbarium (Stafleu and Cowan 1981) as the lectotype for *S.vagum* because it is clear that it was used by Nees, comes from one of the original collections in the Wallich herbarium and has both mature flowers and fruits. This collection from the Wight herbarium (Wallich cat. 2624b) also has more widely distributed duplicates than the other syntype from the Heyne herbarium (Wallich cat. 2624a). Although a specimen in BM (BM000900081) is labelled “Wall. cat. 2624b” it is also labelled as being from “herb. Heyne” and so is here treated as a syntype, not as an isolectotype.

#### Specimens examined.

See Suppl. materials 1–3.

### ﻿47. Solanum viarum

Taxon classificationPlantaeSolanalesSolanaceae

Dunal, Prodr. [A.P. de Candolle] 13(1): 240. 1852.

4C9F3FF2-D7D5-50A3-A318-9A48CA7672AB

Figs 3H , 73C–E Names based on tropical Asian types only; for complete synonymy see Nee 1979 ; Vorontsova and Knapp 2016.

Solanum khasianum C.B.Clarke var. chatterjeeanum Sengupta, Bull. Bot. Surv. India 3: 413. 1961. Type. India. Tamil Nadu: “Madras, Nilgiris district”, 1075 m, 17 Jul 1960, *K. Subramanyam 10413* (holotype: CAL [CAL-10413A]; isotypes: CAL [CAL-10413B, CAL-10413C]).

#### Type.

Brazil. São Paulo: sin. loc., *P. Lund 799* (holotype: G-DC [G00145818, F neg. 6816, IDC microfiche 800-61.2080:I.1]).

#### Description.

Vorontsova and Knapp (2016: 365–368); http://www.solanaceaesource.org/solanaceae/solanum-viarum.

#### Distribution.

*Solanumviarum* is widely distributed in tropical Asia, particularly in China and northern India; native to Brazil, this species is widely adventive and can be invasive (see below). *Solanumviarum* has been formally assessed as of Least Concern (LC) on the IUCN Red List (Knapp 2020).

#### Common names.

China. mao guo qie (Zhang et al. 1994). India. Andaman and Nicobar Islands: jangli baigun (*Basu ANC-6866*).

#### Discussion.

*Solanumviarum* is a member of the Acanthophora clade (sensu Stern et al. 2011; Gagnon et al. 2022), along with *S.aculeatissimum*, *S.capsicoides*, and *S.mammosum*. All members of the clade can be distinguished by their pubescence of simple trichomes (derived from stellate midpoints, see Whalen 1984) on upper leaf surfaces. It can be distinguished from the rest of the members of the clade in tropical Asia in its dense and fine glandular pubescence on all parts, relatively small fruits, unwinged seeds, and small, pale greenish cream flowers. It is most similar to *S.aculeatissimum*, which has similarly small berries, but differs from that species in its deltate, rather than long acuminate, calyx lobes and puberulent, rather than stipitate-glandular, ovary. The prickles of *S.viarum* are often slightly curved and of uniform length, while those of *S.aculeatissimum* are straight and of varying lengths.

*Solanumviarum* and *S.aculeatissimum* were both previously known as *S.khasianum* (see Babu and Hepper 1979), but subsequent monographic work across the native range of these taxa (Nee 1979) clarified that the two were distinct.

*Solanumviarum* has potent antioxidant compounds similar to those used medicinally from other species in tropical Asia (e.g., *S.insanum*, see Uses, pg. 18–20) and has potential for medicinal or other use in the area (Wu et al. 2012). In Bhutan it has been recorded as being grown as a source of the alkaloid solasodine (Mill 2001).

*Solanumviarum* (under the common name soda apple or tropical soda apple) has been classified as a noxious weed in the southern United States and Australia (https://www.ars.usda.gov/news-events/news/research-news/1999/digging-burning-thwart-soda-apple-weed/; https://weeds.dpi.nsw.gov.au/Weeds/Details/186). Where we have seen it in tropical Asia it is similarly aggressive and can take over fields and pastures.

#### Specimens examined.

See Suppl. materials 1–3.

### ﻿48. Solanum violaceum

Taxon classificationPlantaeSolanalesSolanaceae

Ortega, Nov. Pl. Descr. Dec. 56. 1798.

366CD9AD-3E00-5F5A-A80E-914687E6093A

Figs 3A , 78

Solanum chinense Dunal, Hist. Nat. Solanum 240. 1813. Type. China. Sin. loc. (lectotype, designated here: [illustration] Plukenet, Phytographia, pars altera, tab. 62, fig. 1. 1691).
Solanum heynei Roem. & Schult., Syst. Veg., ed. 15 bis [Roemer & Schultes] 4: 669. 1819, as “*Heynii*”. Type. “H. in India orientali. B. Heyne”, *B. Heyne s.n.* (lectotype, designated by Turner 2021, pg. 418: L [L 0403734]).
Solanum pinnatifidum Roth, Nov. Pl. Sp. 130. 1821, nom. illeg. non S.pinnatifidum Lam., 1794. Type. Based on same material and homotypic with S.heynei Roem. & Schult.
Solanum indicum Nees var. sinuato-lobatum Dunal, Prodr. [A. P. de Candolle] 13(1): 309. 1852. Type. Indonesia. “Malacca”, *H. Cuming 2261* (lectotype, designated here: G [G00442779]; isolectotypes: BM [BM000886164, BM000886188], E [E00526948], LE, K [K000014604, K000014606], P [P00055666]).
Solanum indicum Nees var. eroso-pinnatifidum Dunal, Prodr. [A. P. de Candolle] 13(1): 310. 1852. Type. India. Sin. loc., “Bengala inferior”, 1817, *N. Wallich s.n.* [Wallich Catal. 2626e] (lectotype, designated here: G-DC [G00130375]; isolectotypes: GZU [GZU000255428], K-W [K001116646 pro parte, bottom L hand fragment only]).
Solanum indicum Nees var. parvifolium Dunal, Prodr. [A. P. de Candolle] 13(1): 310. 1852. Type. Mauritius. “Isle Maurice”, 1839, *L. Bouton s.n.* (lectotype, designated here: G-DC [G00130374]).
Solanum junghuhnii Miq., Fl. Ned. Ind. 2: 649. 1857. Type. Indonesia. Java: Central Java, “op Tjilatjap” [Cilacap], *H. Gesker s.n.* [195] (lectotype, designated here: L [L0003638]).
Solanum indicum L. var. inerme Van Heurck & Müll.Arg., Observ. Bot. (Van Heurck) 2: 133. 1871. Type. India. Assam: Sin. loc., *W. Griffith 1000* (holotype: BR [AWH10071564]; isotype: BM [BM000900299]).
Solanum pubescens Willd. var. lobatum C.B.Clarke, Fl. Brit. India [J. D. Hooker] 4: 231. 1883. Type. India. Meghalaya: “Assam, Khasi Hills” [Khasia Hills], *S. Kurz s.n.* (lectotype, designated here: CAL [CAL0000018697]).
Solanum kurzii Brace ex Prain, J. Asiat. Soc. Bengal, Pt. 2, Nat. Hist. 65: 541. 1896. Type. India. Sikkim: Sin. loc., 8 May 1874, *G. King s.n.* (lectotype, designated here: K [K000441390]).
Solanum indicum L. var. mesarchon Bitter, Repert. Spec. Nov. Regni Veg. Beih. 16: 9. 1923. Type. Mauritius. Pamplemousses District: “Umgebung von Pamplemousse” [surroundings of Pamplemousse], May 1887, *S. Paulay s.n.* (holotype: W [acc # 1887-0010053]).
Solanum sanitwongsei Craib, Kew Bull. 1928: 246. 1928. Type. Thailand. Bangkok: “cultivated”, 20 Nov 1927, *A.F.G. Kerr s.n.* (lectotype, designated here: K [K000922035]).
Solanum nivalomontanum C.Y.Wu & S.C.Huang, Acta Phytotax. Sin. 16(2): 74. 1978. Type. China. Yunnan: Fengqing, “Shunning, Snowrange [transl. from the protologue]”, 25 May 1938, *T.T. Yu 15962* (lectotype, designated here: PE [PE00031398]; isolectotype: KUN [KUN183591], PE [PE00031399]).
Solanum indicum L. forma album C.Y.Wu & S.C.Huang, Fl. Yunnanica 2: 580. 1979. Type. China. Yunnan: Yanshan, “Yan-shan-hsien, Bar-garh [transl. from the protologue]”, 16 Nov 1939, *C.W. Wang 85018* (lectotype, designated here: PE [PE00031401]; isolectotype: KUN [KUN183578]).

#### Type.

India. “*Habitat* en Bahia Botanica. *Floret* Octobri et Novembri in Reg. Hort. Matrit. è seminibus Londino missis per *Exc. D. Marchionissam de Bute*” (neotype, designated by Knapp 2013b, pg. 59: MA [MA307449]).

**Figure 78.**
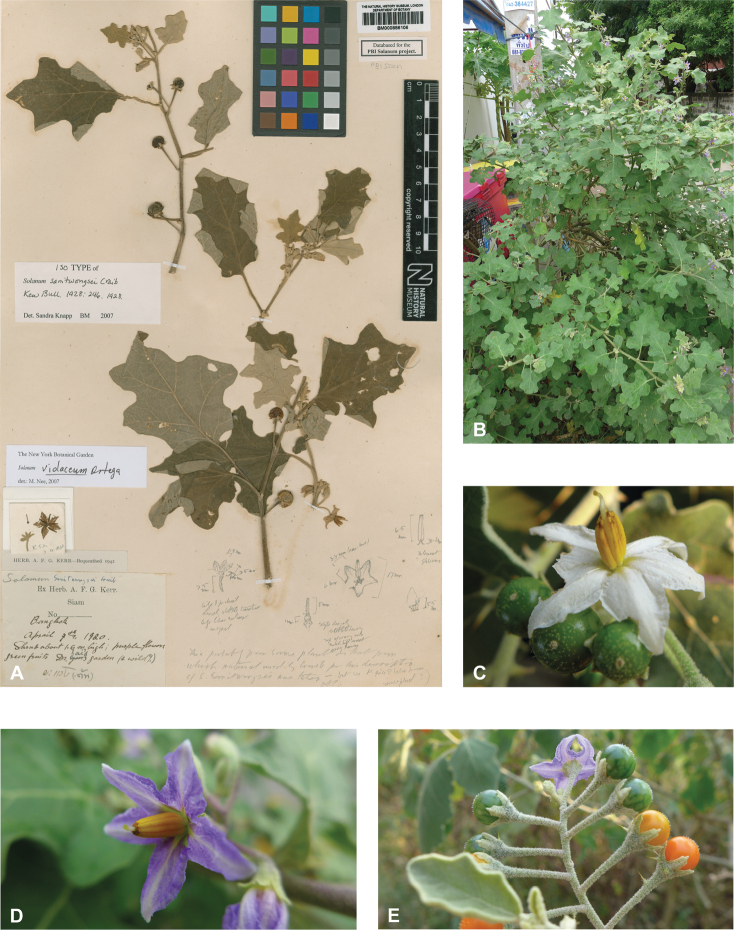
*Solanumviolaceum* Ortega **A** herbarium specimen collected in Thailand in 1920 (*Kerr s.n.*, BM000886106) **B** habit (field photograph, unvouchered, Thailand) **C** detailed view of a white flower with immature fruits (*Sampath Kumar et al. 126947*, India) **D** detailed view of a purple flower (field photograph, unvouchered, Thailand) **E** detail view of an infructescence (*Sampath Kumar et al. 126945*, India). Photograph credits: **A** CC-BY, Muséum national d’Histoire naturelle, Paris **B–E** X. Aubriot.

#### Description.

Erect shrub, to 3 m tall, armed or unarmed. Stems erect, flattened to terete, prickly or less often unarmed, moderately to densely stellate-pubescent; prickles to 10 mm long, to 7 mm wide at the base, usually curved, sometimes straight, rounded or flattened, straw-yellow to orange-brown, glabrous or sparsely pubescent in the lower 1/3; pubescence of sessile or shortly stalked porrect-stellate trichomes, the stalks less than 0.1 mm long, the rays 6–8, 0.1–0.2 mm long, the midpoints same length as the rays or to 1.5 mm long; new growth densely stellate-pubescent, the trichomes like those of the stems; bark of older stems brown, glabrescent. Sympodial units difoliate, the leaves geminate. Leaves simple, more or less deeply lobed, the blades 4–12 cm long, 2.5–11 cm wide, 1.5–2 times longer than wide, usually ovate in outline, sometimes elliptic, chartaceous, strongly discolorous but sometimes concolorous on dry material, unarmed or with several straight prickles to 10 mm long on veins of both surfaces; adaxial surface evenly and moderately to densely stellate-pubescent with sessile or short-stalked trichomes, the stalks to 0.5 mm, the rays 4–10, ca. 0.5 mm long, the midpoints shorter than the rays or to 2 mm long; abaxial surface densely stellate-pubescent with trichomes like those of the adaxial surface but with longer rays and midpoints, the lamina not usually visible; principal veins 3–5 pairs; base usually truncate, often oblique; margins lobed, the lobes (2-)3(-4) on each side, 1–4.5 cm long, broadly deltate to oblong or obovate, often with secondary lobes, apically obtuse, sometimes acute or rounded, the sinuses extending to 2/3 of the way to the midrib; apex acute; petiole 1–6 cm long, 1/5–3/5 of the leaf blade length, densely stellate-pubescent, unarmed or with a few straight or slightly curved prickles. Inflorescences, 2.5–8(–10) cm long, lateral or leaf-opposed, unbranched or sometimes forked, with 5–15(–30) flowers, 2–3 flowers open at any one time, moderately to densely pubescent with stellate-porrect trichomes like those of the stems, unarmed or with a few straight prickles; peduncle 0.1–1(–2.5) cm long, usually unarmed; pedicels 0.8–1.7 cm long, 0.7–1 mm in diameter at the base, 1.6–2 mm in diameter at the apex, straight and slender, unarmed, densely stellate-pubescent like the inflorescence axes, articulated at the base; pedicel scars spaced 2–10 mm apart. Buds ovoid, the corolla strongly exserted from the calyx before anthesis. Flowers 5-merous, apparently all perfect. Calyx with the tube 1.5–2 mm long, obconical to cup-shaped, the lobes 1.5–4 mm long, 1.5–2.5 mm wide, deltate, apically apiculate to acute, rarely long-acuminate, with no venation visible, unarmed or with up to 10 straight prickles, densely stellate-pubescent with sessile porrect-stellate trichomes like those of the rest of the inflorescence. Corolla 1.3–3 cm in diameter, pale blue to purple or more rarely white, stellate, lobed 1/2–2/3 of the way to the base, the lobes 4.5–8 mm long, 3–5 mm wide, deltate, spreading to slightly reflexed at anthesis, mostly glabrous adaxially, moderately to densely stellate-pubescent abaxially, the trichomes porrect, sessile to shortly stalked, the stalks up to 0.1 mm, the rays 6–8, 0.1–0.2 mm, the midpoints shorter than the rays, but lengthening towards corolla lobe apices. Stamens equal; filament tube minute; free portion of the filaments 0.5–1 mm long, glabrous; anthers 4.5–8.5 mm long, 1.2–1.5 mm wide, yellow-orange or yellow, connivent, tapering, glabrous but sometimes with a few stellate trichomes, poricidal at the tips, the pores directed distally and lengthening to slits with age. Ovary ovoid, glabrous but with a few stellate trichomes towards the apex; style 7–11(15) mm long, filiform, straight to gently curved, stellate-pubescent in the lower 1/3–2/3; stigma clavate to capitate, the surface minutely papillose. Fruit a globose berry, several per infructescence, 0.7–0.9 cm in diameter, yellow to orange when ripe, the pericarp thin and shiny, glabrous; fruiting pedicels 1.2–1.8 cm long, 0.5–1.2 mm wide at the base, 3–3.5 mm in diameter at the apex, somewhat woody, straight, spreading, unarmed or with a few straight prickles; fruiting calyx lobes elongating to 3.5–6(–8) mm long, 1/4–1/2 of the length of the mature fruit, spreading and usually not reflexed, unarmed or with up to 10 straight prickles. Seeds ca. 10–15 per berry, 2.2–3.5 mm long, 1.8–3 mm wide, flattened-reniform, yellow to orange-brown, the surfaces minutely pitted, the testal cells with thick, strongly sinuate margins. Chromosome number: n = 12 (Karihaloo 1991), 2n = 24 (Karihaloo 1991; Das and Borah 2015, as *S.kurzii*).

#### Distribution

**Figure 79.**
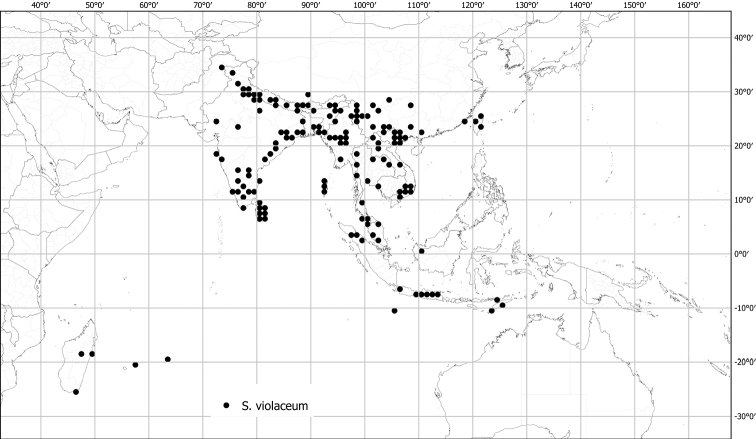
Distribution of *S.violaceum*.

**(Fig. 79).***Solanumviolaceum* occurs across Asia, from India to China, Vietnam and Malaysia, from the coasts to inland mountains between ca. 3–27° latitude. It is very common in India, southern China, and Thailand. The westernmost recorded occurrences are on the islands of Mauritius, Réunion and Rodrigues. Chinese populations are restricted to the more southern regions of Fujian, Guangdong, Guangxi, Hainan, Hong Kong, Sichuan Taiwan, and Yunnan (Zhang et al. 1994). There are occasional collections from Sumatra and Java, but it is not clear whether these are from the wild or cultivated. *Solanumviolaceum* does not occur wild in the Philippines, New Guinea, or Australia.

#### Ecology and habitat.

*Solanumviolaceum* is usually found growing in open places, abandoned cultivation, and roadsides, in a variety of forest types; between sea level and 2,000 m elevation.

#### Common names and uses.

China. ci tian qie (Zhang et al. 1994); Yunnan: xue shan qie (eggplant from the snow mountain; *Yu 15962*). India. Bihar: jangali baigan (Varma 1981, as *S.indicum*); Goa: ringani, inoti-ringani, badane, dorli (darli), motaring (Naithani et al. 1997); Karnataka: mullsunde, kad badne, ustikai (Singh 1988); Kerala: cheruchunda (Mohanan and Henry 1994, as *S.anguivi* Lam.); Tamil Nadu: naaimulli, mulluchundai [Tamil] (Matthew 1983); cherukinda, cheruvazhuthanai [Malayalam], ciruvaludalai, kondal, karimulli [Tamil] (Nair and Nayar 1987, as *S.indicum*), mulli, pappara-mulli, karimulli [Tamil] (Henry et al. 1987, as *S.anguivi*). Malaysia/Singapore: tĕrong pipit puteh, tĕrong pipit hijau (Burkill 1935), tèrong peuheur [Sundanese] (Burkill 1935).

The roots of *S.violaceum* (as *S.indicum*) are one of the ingredients of Dashamoola of Ayurvedic medicine used to treat inflammatory conditions of all kinds (Naithani et al. 1997; Jain et al. 2000). It is regarded as a diuretic, is used for treatment of dropsy, and as an asthma and catarrhal expectorant (Jain et al. 2000). Unripe fruits are used in the preparation of curries (Naithani et al. 1997).

#### Preliminary conservation status

**(IUCN 2019).** Least Concern (LC). EOO (10,078,363 km^2^, LC); AOO (988 km^2^, VU). *Solanumviolaceum* is one of the most widely distributed spiny solanums of tropical Asia and grows in a wide variety of disturbed habitats, often as large populations of individuals.

#### Discussion.

*Solanumviolaceum* is very widespread and is common where it occurs. In the older literature the name *Solanumindicum* was often used for this taxon, but considerable confusion over its application led to its suppression (Hepper 1978; see Doubtful and excluded names).

*Solanumviolaceum* is a plant of disturbed areas, often growing along roadsides and in stream beds. It has been confused with *S.anguivi* Lam., an African taxon, because of its small orange to red berries and small, hermaphroditic flowers (e.g., Hepper 1978; Singh 1991), but is not directly related to that species. It differs from *S.anguivi* in its orange, rather than red, berries, its usually violet or pale violet, rather than white flowers, and in its straight, spreading pedicels in fruit, rather than slightly curved pedicels.

In the Flora of Bhutan (Mill 2001) *S.violaceum* was treated as *S.kurzii* (specimens with few prickles) and *S.anguivi* (prickly plants).

*Solanumviolaceum* is sympatric with its close relatives *S.deflexicarpum* of southern China, *S.hovei*, and *S.multiflorum*, the latter two endemic to India. All of these species have strongly deflexed pedicels in fruit, while those of *S.violaceum* are broadly spreading and usually longer. See those species descriptions for more details. *Solanumviolaceum* is quite variable across its range in leaf shape and prickliness; populations from Sri Lanka and Thailand lacking prickles have been called *S.kurzii* and *S.sanitwongsei*, respectively. Similar unarmed plants, however, are found across the range of *S.violaceum* and even within populations.

*Solanumchinense* was described as a species of uncertain status related to *S.violaceum* (Dunal 1813) referring only to Tournefort (1700) and an illustration from Plukenet (1691); we have found no other authentic original material, and so lectotypify the name using the Plukenet illustration which matches the protologue.

The varieties of *S.indicum* sensu Nees described by Dunal (1852), were based on the polynomial varieties from Nees van Esenbeck (1834), which themselves were based entirely on specimens from Wallich’s herbarium that he saw in London (De Candolle and Radcliffe-Smith 1981). Dunal (1852) cited a number of specimens and concepts in synonymy, making typification difficult, but in lectotypifying these infraspecific names we have used specimens at G-DC that Dunal definitely would have seen during his preparation of the Prodromus treatment. For var. sinuatolobatum we have selected as the lectotype a collection specifically mentioned in the protologue “Cuming 2261, hb. Boiss.” (G00442779) that is widely duplicated. For var. parvifolium we have selected the *Bouton s.n.* specimen from Mauritius in G-DC cited in the protologue (G00130374) because it is an unambiguous, well-preserved specimen. Var. eroso-pinnatifidum cited “S.pinnatifidum*Roth*, S. heynii *R.&S.*, *Solanumindicum Wall. cat. 2626* D,E” as material; we have selected the G-DC specimen of “Wallich cat. 2626E” (G00130375) as the lectotype of this variety. The “duplicate” in the Wallich herbarium at Kew (K00116646) is a mixture of potentially three elements, only the lower L hand stem of which we regard as isolectotype material; this fragment has the letter “E” in pencil, as does the larger R hand fragment. This larger fragment appears to be a collection of *S.multiflorum*, and so we exclude it as type material for var. eroso-pinnatifidum. Duplicates of all these varieties in Nees van Esenbeck’s own herbarium at GZU all correspond to *S.violaceum* and are considered isolectotypes.

Miquel (1857) cited three specimens in the protologue of *S.junghuhnii*: *Gesker s.n.* from “Tjiltajap”, *Junghuhn s.n.* from “Awoe Awoe” and *Horsfield s.n.* from “Soerakarta”. We have selected the Leiden specimen (L 0003638) collected by F.W. Junghuhn as the lectotype, it is labelled unambiguously as to locality and matches the protologue.

Clarke (1883) indicated his uncertainty over the identity of his S.pubescensvar.lobatumby stating “Var.?lobata” and “it resembles the unarmed form of *S.Melongena* but the flowers are too small.” He cited a specimen “from Herb. Calcutta, named *S.pubescens* by Kurz”. Several specimens in CAL are so labelled, we have selected the best of these (CAL0000018697) as the lectotype.

The protologue of *S.kurzii* (Prain 1896) cites three specimens, two from Sikkim (*Thomson s.n.* and *King s.n.*) and one from “Khasia” (*Mann s.n.*). Specimens collected from the effort towards the flora of British India (Endersby 2008) are usually un-numbered and have minimal to no locality information. Tracing them can be challenging. A sheet at Kew (K000441390) collected by George King in Sikkim in 1874 matches the protologue in being nearly without prickles and is here selected as the lectotype for *S.kurzii*.

The protologue of *S.santiwongsei* cites only an un-numbered collection of A.F.G. Kerr from Bangkok, with no further details. Craib in his many papers of additions to “Siamese plants” never cited herbaria, but in the final compilation of these lists (Craib 1928) stated that the distributions of all species were taken from specimens at Kew. We have therefore selected a specimen collected by Kerr in Bangkok of a cultivated plant annotated with the name *S.sanitwongsei* (K000922035), as the lectotype of this name.

The type of *S.nivalomontanum* is indicated as being in PE (Wu and Huang 1978), but two duplicates of the type gathering are housed there; we here select the better preserved of these (PE00031398) as the lectotype. The protologue of S.indicumformaalbum cites a gathering (*Wang 85018*; Wu and Huang 1979), but no herbarium is mentioned. We have selected the duplicate preserved in PE (PE00031401) as the lectotype, because we have been able to access images of it and ascertain its identity.

#### Specimens examined.

See Suppl. materials 1–3.

### ﻿49. Solanum virginianum

Taxon classificationPlantaeSolanalesSolanaceae

L., Sp. Pl. 187. 1753.

1A25D1F6-4603-5177-8264-D6FA68DFE14C

Figs 2D , 80

Solanum surattense Burm.f., Fl. Ind. (N. L. Burman) 57. 1768. Type. India. Gujarat: “Surat”, *L. Garcin* s.n. (lectotype, designated by Hepper and Jaeger 1986, pg. 434, as “type”; second step designated here: G-PREL [G00811278]; isolectotype: G-PREL [G00811279]).
Solanum virginicum L., Mant. 2: 340. 1771. Type. Based on (orthographic error for) Solanumvirginianum L., not intended as a new name.
Solanum armatum Forssk., Fl. Aegypt.-Arab. 47. 1775. Type. Yemen. Al Hudaydah: Hays, “ad Haes” [from protologue], *P. Forsskål s.n.* (lectotype, designated here: KIEL [KIEL0005103]).
Solanum xanthocarpum Schrad. & J.C.Wendl., Sert. Hanov. 1: 8, tab. 2. 1795. Type. Based on Solanumvirginianum L. (see Hepper and Jaeger 1986).
Solanum jacquinii Willd., Sp. Pl., ed. 4 [Willdenow] 1(2): 1041. 1798, nom. illeg. superfl. Type. Based on Solanumvirginianum L. (cited indirectly in synonymy).
Solanum arabicum Dunal, Hist. Nat. Solanum 240. 1813, nom. illeg. superfl. Type. Based on Solanumarmatum Forssk. (cited in synonymy).
Solanum xanthocarpum Schrad. & J.C.Wendl. var. schraderi Dunal, Prodr. [A. P. de Candolle] 13(1): 302. 1852. Type. Cultivated in the Calcutta Botanical Garden, “Hort Calc”, *N. Wallich s.n.* [Wallich Catal. 2612b] (lectotype, designated here: G-DC [G00130292]; isolectotypes: BM [BM000900258], K [K001080635], K-W [K001116573]).
Solanum xanthocarpum Schrad. & J.C.Wendl. var. jacquinii (Willd.) Dunal, Prodr. [A. P. de Candolle] 13(1): 303. 1852. Type. Based on Solanumjacquinii Willd.
Solanum mairei H.Lév., Repert. Spec. Nov. Regni Veg. 12: 531. 1913. Type. China. Yunnan: “Plaine de Kiao-Kia”, May 1912, *E.E. Maire s.n.* (lectotype, designated here: E [E00284473]).
Solanum mccannii Santapau, J. Bombay Nat. Hist. Soc. 47: 654. 1948. Type. India. Maharashtra: Pune, “Khandala”, 18 Oct 1943, *H. Santapau 2972* (lectotype, designated here: BLAT [acc. # 89719]).

#### Type.

“America” (lectotype, designated by Hepper and Jaeger 1986, pg. 434: [illustration] “Solanum American. laciniatum spinossimum”, Dillenius, Hort. Eltham. 360, t. 267, f. 346. 1732).

#### Description.

Prostrate shrubs, to 60 cm tall, heavily armed. Stems sprawling to erect, terete, densely prickly and sparsely stellate-pubescent; prickles to 2 cm long, broad-based, straight, straw-yellow in dry material, pale whitish green in live plants; pubescence of sessile porrect-stellate trichomes, the rays 4–7, 0.2–0.5 mm long, the midpoints more or less equal in length to the rays; new growth densely stellate-pubescent with a dense covering of papillose glandular unicellular trichomes, yellowish green or purplish green; bark of older stems brown or purplish brown, glabrescent. Sympodial units difoliate, the leaves not geminate. Leaves simple, deeply lobed, the blades (1–)3–11 cm long, 2–4 cm wide, 1–2.5 times longer than wide, elliptic on outline, membranous to somewhat fleshy, concolorous or slightly discolorous, densely armed on both surfaces along the midrib and major veins with prickles like those of the stems; adaxial surface dark green and shiny, sparsely pubescent with sessile porrect-stellate trichomes, the rays 4–7, 0.2–0.5 mm long, the midpoints longer than the rays, moderately papillose with minute unicellular glandular papillae; abaxial surface lighter and usually more densely stellate-pubescent with trichomes like those of the adaxial surface; major veins 3–6 pairs, drying whitish yellow; base truncate, unevenly oblique with one side basiscopically extended along the petiole; margins deeply lobed, the lobes ca. 20 per side, 1.5–2 cm long, 1–1.5 cm wide, narrowly deltate to triangular, often with secondary lobing, apically acute, the sinuses extending up to halfway to the midrib; apex acute; petiole 1.5–5 cm long, 1/2–3/4 of the leaf blade length, very sparely stellate-pubescent with sessile porrect trichomes, densely prickly with more than 10 prickles like those of the stems. Inflorescences 1.5–7 cm long, internodal or occasionally almost opposite the leaves, usually unbranched, but occasionally forked, with 4–8(–10) flowers, only a few open at any one time, glabrous or with a few scattered porrect-stellate trichomes, moderately prickly with straight, straw-coloured prickles to 1.5 cm long; peduncle 1.4–2.5 cm long, moderately prickly with prickles to 1.5 cm long; pedicels 0.6–0.8(–1) cm long, ca. 1 mm in diameter at the base, 1–1.5 mm in diameter at the apex, erect, glabrous or sparsely pubescent with porrect-stellate trichomes like those of the stems articulated at the base; pedicel scars spaced 0.7–2 cm apart. Buds elongate-ovoid, pointed at the tip, strongly exserted from the calyx tube before anthesis. Flowers 5-merous, heterostylous and the plants weakly andromonoecious, with the lowermost flower(s) long-styled and hermaphrodite, the distal flowers short-styled and staminate. Calyx with the tube 2–3 mm long, cup-shaped, the lobes 2–3 mm long, 1.5–2 mm wide, broadly deltate abruptly narrowing to a slender subulate acumen 1–1.5 mm long, apically acute, densely prickly with prickles to 0.7 cm long, the prickles denser on long-styled flowers, glabrous or sparsely stellate pubescent with porrect trichomes like those of the pedicels. Corolla 2.5–3 cm in diameter, deep violet and fragrant, stellate to almost rotate with abundant interpetalar tissue, lobed 1/4–1/2 of the way to the base, the lobes 0.7–1 cm long, 0.5–0.9 cm wide, broad-deltate, spreading or slightly reflexed, glabrous or sparsely stellate-pubescent along the midveins adaxially, moderately stellate-pubescent abaxially especially on the parts of the corolla exposed in bud and on the tips. Stamens equal; anthers 7–9 mm long, 1.7–2 mm wide, tapering, more or less connivent, bright yellow, glabrous, poricidal at the tips, the pores directed distally, not elongating to slits with age; filament tube minute; free portion of the filaments 0.5–1 mm long, glabrous. Ovary conical, stellate-pubescent with white trichomes that are soon deciduous; style 12–15 mm long and strongly recurved in long-styled flowers, 2–2.5 mm long in short-styled flowers, glabrous or very sparsely pubescent with a few stellate trichomes near the base, the trichomes weak and deciduous; stigma capitate, minutely papillose. Fruit a globose berry, 1–3 per infructescence, 1.5–2.5 cm in diameter, marbled green and white when young, bright yellow when mature, the pericarp thick and leathery, smooth, glabrous; fruiting pedicels 1.2–1.5 cm long, 2–3 mm in diameter at the base, 3–5 mm in diameter at the apex, unarmed or with a few small straight prickles shorter than those of the calyx, thickened and slightly fleshy, strongly recurved, the apical quarter ridged; fruiting calyx lobes elongating to 4 mm long, ca. 1/4 the length of the mature fruit, thickened and slightly fleshy in live plants, appressed to the berry. Seeds 50–100 per berry, 2.5–4 mm long, 2–2.5 mm wide, reniform, not markedly flattened, pale yellowish tan, the surfaces minutely pitted, the testal cells sinuate in outline. Chromosome number: 2n = 24 (Rajasekaran 1971; Das and Borah 2015, both as *S.xanthocarpum*).

**Figure 80.**
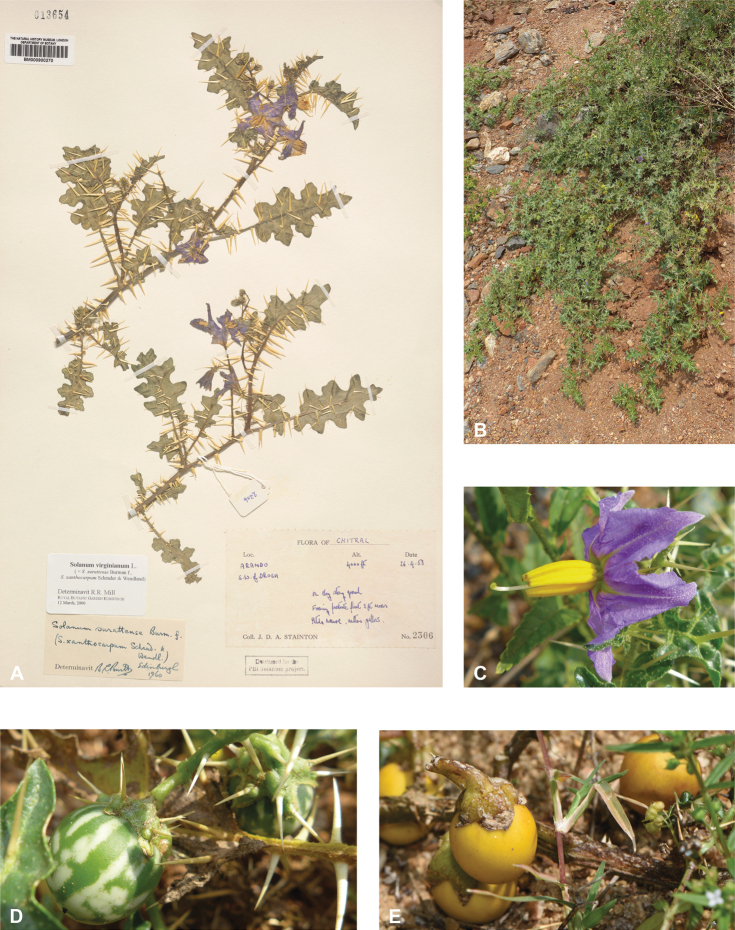
*Solanumvirginianum* L. **A** herbarium specimen collected in India in 1958 (*Stainton 2306*, BM000900270) **B** habit (*Sampath Kumar et al. 126968*, India) **C** detailed view of a flower (*Sampath Kumar et al. 126968*, India) **D** detailed view of immature fruits (*Sampath Kumar et al. 126968*, India) **E** detailed view of mature fruits (field photograph, unvouchered, India). Photograph credits: **A** CC-BY, © copyright The Trustees of the Natural History Museum, London **B–D** X. Aubriot **E** S. Knapp.

**Figure 81.**
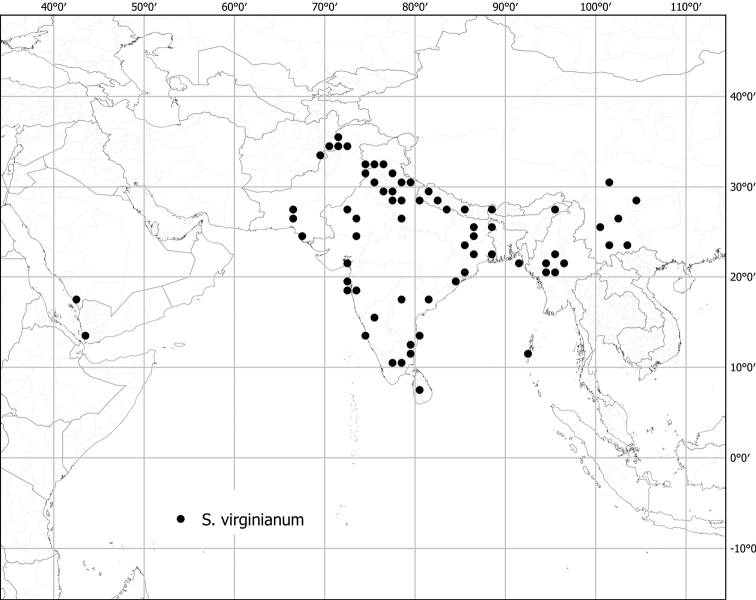
Distribution of *S.virginianum*.

#### Distribution

**(Fig. 81).***Solanumvirginianum* occurs from the Arabian Peninsula (Saudi Arabia, Yemen) to China and Myanmar.

#### Ecology and habitat.

*Solanumvirginianum* is a plant of disturbed places, such as roadsides, streambeds and edges of fields, and often occurs in large populations and forms impenetrable mats; from sea level to 3,100 m elevation (highest elevation from Afghanistan).

#### Common names and uses.

China. mao guo qie (Zhang et al. 1994). India. Bihar: rengani, kataila (Varma 1981), as *S.surattense*); Haryana: kateli (Jain et al. 2000, as *S.surattense*); Kerala: kandankathiri (Mohanan and Henry 1994, as *S.surattense*); Rajasthan: baiga-kateli, bhurangi, dhaturi [Hindi] (Singh 1991); Tamil Nadu: kandankathiri [Tamil] (Matthew 1983); kandangattiri [Tamil] (Henry et al. 1987).

*Solanumvirginianum* is widely used in medicine as one of the “dasa mulikas” of traditional Indian medicine; used in treatment of asthma, cough, fever etc. (Tadulingam and Venkatanarayana 1932; Jain et al. 2000). The whole plant is considered highly medicinal (Singh 1988).

#### Preliminary conservation status

**(IUCN 2019).** Least Concern (LC). EOO (7,899,801 km^2^, LC); AOO (336 km^2^, EN). *Solanumvirginianum* is a weedy species, forming dense patches in open disturbed areas and along dry riverbeds. Tropical Asia represents the easternmost part of its range.

#### Discussion.

*Solanumvirginianum* is a seriously mis-named species, Linnaeus (1753: 187) thought it originated in the then-English colony of Virginia (“Habitat in America” – taken from the polynomials cited in synonymy), while in fact it is not found there except as a recent introduction in ships ballast. With its almost glabrous leaves, prominent straw-coloured prickles, large, fragrant purple flowers and weedy habit it is easily recognised and unlikely to be confused with any other species in the region. In the field the flowers of *S.virginianum* are sweetly fragrant but little is known about the pollination of this, or any other spiny solanum in the region (except *S.insanum*, see Davidar et al. 2015).

The introduced *S.aculeatissimum* and *S.viarum* are similarly prickly, but the prickles are not as long and in those species the leaf pubescence is apparently simple, not stellate. The flowers of both these taxa are white or pale greenish white and the leaves are ovate to broadly elliptic rather than narrowly elliptic in outline.

*Solanumvirginianum* is sister to the main set of African clades of spiny solanums (Aubriot et al. 2016a), albeit this relationship is only weakly supported and is an isolated lineage; no other species clustered with it in the molecular analyses. Its affinities apparently lie with African rather than tropical Asian lineages such as the ‘Sahul-Pacific clade’.

Hepper and Jaeger (1986) inadvertently and effectively lectotypified *S.surattense* with citation of “Type.: Pakistan, Surat, [....] *Garcin* in Hb. *Burmann*” (G-Herb. Delessert!). The Burmann herbarium is held in G-PREL; pre-Linnaean collections were extracted from the general herbarium after 1992, and are no longer part of the Delessert herbarium. Two sheets of *Garcin s.n.* are housed in G-PREL necessitating a second lectotypification step. We have chosen the more complete of these sheets (G00811278) as the lectotype for *S.surattense.*

We have lectotypified *S.armatum* using the only authentic Forsskål material we have found in the Kiel University herbarium (KIEL0005103), although the specimen does not have an indication of the locality.

*Solanumjacquinii* is an illegitimate superfluous name because *S.virginianum* was cited in synonymy indirectly by citation of Jacquin’s (1786–1793) illustration, which itself cited Linnaeus. The specimen in the Willdenow herbarium at Berlin (B-W-04384-00 0) bears a copy of Willdenow’s short description on the cover and is certainly that used in the description.

In the description of S.xanthocarpumvar.schraderiDunal (1852) cited a number of names and elements, including *S.xanthocarpum* itself, suggesting he was considering this the typical variety. We have elected to typify the name however, since the citations are not clear, and choose as the lectotype the G-DC duplicate of *Wallich cat. 2618b* (G00130292) that is specifically cited in the protologue.

The herbarium of the French botanist and clergyman Augustin A.H. Léveillé was acquired by the Scottish botanist George Forrest from whence it passed to the Royal Botanic Garden Edinburgh. We have selected the specimen at E (E00284473) that corresponds to the description, collector and locality (Léveillé 1913), as the lectotype for *S.mairei*.

#### Specimens examined.

See Suppl. materials 1–3.

### ﻿50. Solanum wightii

Taxon classificationPlantaeSolanalesSolanaceae

Nees, Trans. Linn. Soc. London 17(1): 51. 1834.

DDB96F9B-2EF0-5BAE-9D2D-A675F74EB07D

Fig. 82

Solanum hohenackeri Van Heurck & Müll.Arg., Observ. Bot. (Van Heurck) 87. 1870. Type. India. Tamil Nadu: Nilgiris, “in montibus Nilagiri”, *R.F. Hohenacker 1076* (lectotype, designated here: BR [AWH10071212]; isolectotypes: BM [BM000778309], G [G00442609, G00442943, G00442944], HAL [HAL0010834], K [K000441384], L [0403690], LE [2 sheets], MEL [MEL2446521], P [P00055713, P00055714], W [acc. # 0000605]).
Solanum pulneyensis Soosairaj, Adansonia sér. 3, 43(21): 236. 2021. Type. India. Tamil Nadu: Dindigul district, Palani Hills National ark, Thonimalai, ca. 1300 m, 29 Jan 2018. *S. Soosairaj* 2514 (holotype: RHT [acc. # 076723]; isotype: MH).

#### Type.

India. Sin. loc., “Peninsula Ind. orientalis”, *R. Wight 1576/126* (lectotype, designated here: GZU [GZU000255932]; isolectotypes: BM [BM000900156], E [E00179475, E00179476, E00179477], G [G00442945], K [K000441376, K000441378, K000441379], LE [LE00017069]).

#### Description.

Scrambling herbs or shrubs to 2 m tall, armed or unarmed. Stems erect or spreading, terete, prickly and stellate-pubescent; prickles, if present, to 3 mm long, 1.5–2 mm wide at the base, slightly curved, pale yellowish tan; pubescence of sessile to very short-stalked porrect-stellate trichomes, the stalks, if present, to 0.2 mm, the rays 4–6, ca. 0.5 mm long, the midpoints 2–4 celled, to 2 mm long, glandular tipped, drying with violet tinge at cell junctions (trichomes described as black to reddish *Clarke 10793*); new growth densely stellate-pubescent, the trichomes tangled, soon deciduous and the stems glabrate; bark of older stems ashy white. Sympodial units difoliate, the leaves not geminate. Leaves simple, shallowly lobed, the blades 2–9 cm long, 1.7–6.8 cm wide, 1–1.3 times longer than wide, ovate to broadly triangular, widest in the lower third, chartaceous, somewhat discolorous, unarmed or very occasionally sparsely armed along the midrib and major veins with small prickles; adaxial surface evenly and densely pubescent with sessile and very short-stalked porrect-stellate trichomes, the rays 4–8, to 0.5 mm long, glandular at the tips, the midpoints 2–4-celled, to 2 mm long, glandular at the tips; abaxial surface with similar porrect-stellate trichomes, but these denser especially along the veins; major veins 3–4 pairs, densely pubescent; base abruptly truncate to cordate, somewhat oblique; margins shallowly lobed, the lobes 3–4 on each side, to 0.5 cm long, broadly deltate, apically rounded, the sinuses less than halfway to the midrib; apex acute to obtuse; petioles 1–4 cm long, ca. half as long as the leaf blades, unarmed or with a few prickles, densely stellate pubescent like the stems. Inflorescences 0.3–1 cm long, internodal and lateral, unbranched, with 1–3 flowers, only 1 or 2 flowers open at any one time, pubescent with mixed sessile and short-stalked stellate-porrect trichomes like those of the stems, with multicellular midpoints to 2 mm long, unarmed; peduncle absent to 0.2 cm long; pedicels 2.5–4 cm long, ca. 0.7 mm in diameter at the base, ca. 0.7 mm in diameter at the apex, spreading and slightly nodding at anthesis, unarmed or with a few prickles, more sparsely stellate-pubescent than the inflorescence axes, articulated at the base; pedicel scars tightly spaced ca. 1 mm apart. Buds elongate and tapering, curved, strongly exserted from the calyx before anthesis. Flowers 5-merous, heterostylous and the plants andromonoecious, with the distal flower(s) short-styled and smaller than the hermaphroditic flowers. Calyx with the tube 3.5–4 mm long, conical, sparsely prickly, the lobes 6–8 mm long, ca. 1 mm wide, long-triangular to lanceolate, unarmed, densely stellate-pubescent with mixed sessile and short-stalked porrect-stellate trichomes with multicellular glandular midpoints like those of the pedicels, the pubescence denser than that of the pedicels. Corolla (2–)3.5–5 cm in diameter, violet or deep purple, rotate-stellate, lobed to 1/3 of the way to the base, abundant interpetalar tissue present, the lobes (10–)14–17 mm long, (11–)14–18 mm wide, broad-deltate, spreading or somewhat campanulate at anthesis, mostly glabrous adaxially but with a few stellate trichomes along the petal midvein, densely stellate-pubescent abaxially with densely tangled sessile trichomes where exposed in bud, these densest at the tips, the interpetalar tissue glabrous. Stamens markedly unequal; anthers 3 long and 2 short, the long anthers 12–15 mm long, ca. 2 mm wide, strongly curved and tapering, the short anthers ca. 10 mm long, 0.6–0.7 mm wide, straight or slightly curved, all anthers yellow, glabrous, poricidal at the tips, the pores directed distally, not elongating to slits with drying; filament tube minute, glabrous; free portion of the filaments 1–1.5 mm long, glabrous. Ovary conical, glabrous; style ca. 5 mm long in short-styled flowers, 12–15 mm long, in long-styled flowers, strongly curved, glabrous; stigma capitate, the surfaces minutely papillose. Fruit a globose berry, 1–2 per infructescence, 1.1–1.5 cm in diameter, completely enclosed in the accrescent calyx, yellowish brown when ripe, drying and breaking into 4 irregular valves when ripe, the pericarp thin and shiny, glabrous; fruiting pedicels 3–3.5 cm long, 1–1.5 mm in diameter at the base, 3–4.5 mm in diameter at the apex, unarmed, somewhat woody, sharply deflexed and pendent; fruiting calyx strongly accrescent, the lobes often breaking off. Seeds 20–30 per berry, 4–5 mm long, 3–3.5 mm wide, flattened reniform, pale tan or yellowish brown, the surfaces minutely pitted, the testal cells with straight walls, pentagonal in outline, the margins incrassate. Chromosome number: not known.

**Figure 82.**
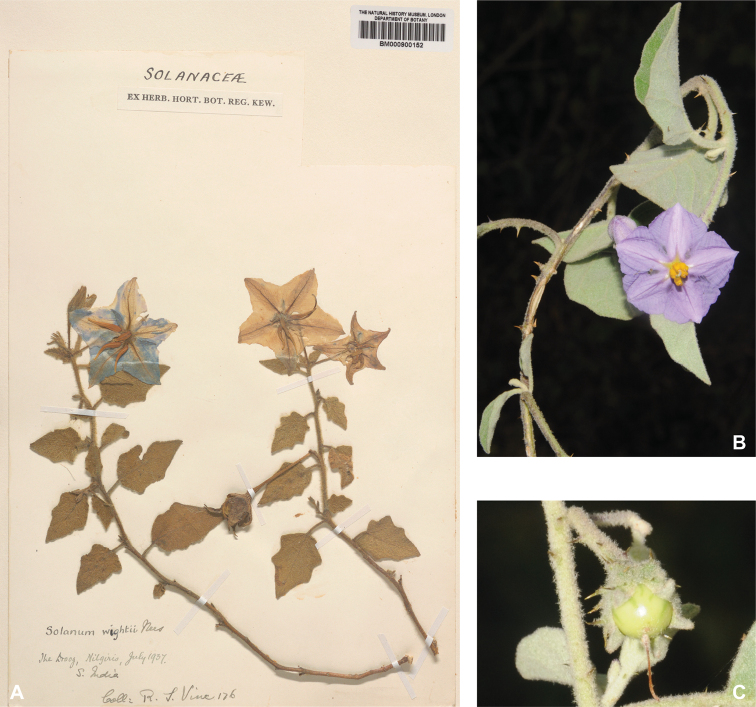
*Solanumwightii* Nees **A** herbarium specimen collected in India in 1937 (*Vine 176*, BM000900152) **B** detailed view of a flower (field photograph, unvouchered, India) **C** detailed view of a fruit (field photograph, unvouchered, India). Photograph credits: **A** CC-BY, © copyright The Trustees of the Natural History Museum, London **B, C** G. Gnanasekaran.

#### Distribution

**Figure 83.**
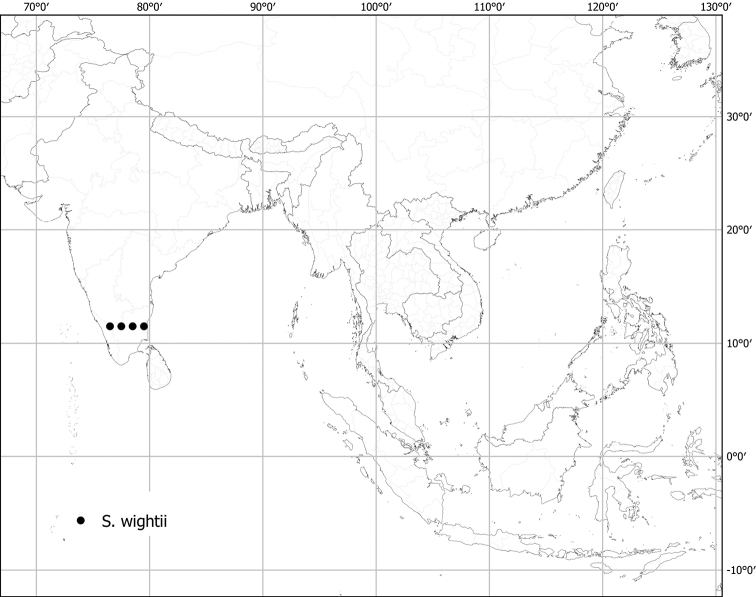
Distribution of *S.wightii*.

**(Fig. 83).***Solanumwightii* is endemic to the mountains of southeastern India in the states of Tamil Nadu and Andhra Pradesh.

#### Ecology and habitat.

*Solanumwightii* occurs in dry forests and forest margins, from 850 to 2,200 m elevation.

#### Common names and uses.

None recorded.

#### Preliminary conservation status

**(IUCN 2019).** Vulnerable (VU). EOO (32,261 km^2^, VU); AOO (44 km^2^, EN). *Solanumwightii* is rather narrowly distributed and occurs in open grassy areas subject to human disturbance; it occurs in the proposed Palani Hills National Park, so there is afforded some degree of protection.

#### Discussion.

*Solanumwightii* is a beautiful, distinctive Indian endemic with large, zygomorphic flowers and berries enclosed in accrescent calyces that are borne on long, strongly deflexed pedicels. The only other species in India with purple zygomorphic flowers is *S.pubescens* which has sticky pubescence, smaller flowers with a single long stamen rather than three, and more fruit on each infructescence borne on shorter pedicels. The berry of *S.wightii* has been recorded as dry and dehiscent (breaks into four valves, *Anon. s.n.*, in MH; described as “subcapsular” in Soosairaj et al. 2021) like members of the Androceras clade (Whalen 1979) and the Elaeagnifolium clade (see Knapp et al. 2017). Field studies of dispersal in *S.wightii* are needed; this type of dry berry is also found in members of the Leptostemonum Clade from Australia (Symon 1981; Martine et al. 2019) where dispersal can be either via a censer (shaking) mechanism or as trample burrs.

Aubriot et al. (2016a) suggest *S.wightii* is sister to *S.praetermissum*, but the relationship is poorly supported, and additional analyses are necessary.

Ramachandran and Viswanathan (2010) reported specimens of *S.wightii* from Sethukadai in the Namakkal District of Tamil Nadu as *S.cordatum*. The illustration in their paper is clearly of *S.wightii* although the description appears to be a mixture of information from published works and the specimens they used. The recently described *S.pulneyensis* (Soosairaj et al. 2021) clearly falls within the range of variation of *S.wightii*, and the holotype specimen at RHT corresponds to *S.wightii*. The character used to distinguish the two species is the dry pseudo-capsular berry, something that is known to occur in *S.wightii* (see above).

We have selected the sheet of *Wight 1576/126* from Nees van Esenbeck’s personal herbarium (GZU000255932) that is annotated in his handwriting as “S. Wightii n.sp.” as the lectotype of *S.wightii*; this collection is widely duplicated. After its initial description (Nees van Esenbeck 1834), Nees van Esenbeck (1836) published a more extensive treatment of *S.wightii*, in which he clarified his ideas about its relationships and illustrated the plant.

Two collections and two herbaria were cited in the protologue (Van Heurck 1870) of *S.hohenackeri* – “Hohenacker 1076, 1417! in hb. Van Heurck et hb. DC”. The van Heurck herbarium, previously held in Antwerp at AWH, has now been acquired by BR, but sheets are still barcoded with the AWH herbarium code. We have selected duplicate of *Hohenacker 1076* held in BR (AWH10071212) as the lectotype for *S.hohenackeri*; this specimen is well-preserved and duplicates of *Hohenacker 1076* are widely distributed.

#### Specimens examined.

See Suppl. materials 1–3.

### ﻿51. Solanum wrightii

Taxon classificationPlantaeSolanalesSolanaceae

Benth., Fl. Hongk. 243. 1861.

95B067FB-9642-5735-A04E-469B1C88BE3C

Fig. 73F–H Names based on tropical Asian types only; for complete synonymy see Vorontsova and Knapp 2016.

#### Type.

China. “Hongkong”, 1853–1856, *C. Wright 489* (lectotype, designated here: K [K000545757]; isotypes: GH [00077826], US [acc. # 79466, barcode 00731429]).

#### Description.

Vorontsova and Knapp (2016: 372–375); http://www.solanaceaesource.org/solanaceae/solanum-wrightii.

#### Distribution.

*Solanumwrightii* has been recorded from several areas in tropical Asia in cultivation as a street tree; it is native to Bolivia but widely cultivated for ornament and as a shade tree in coffee plantations. *Solanumwrightii* has been formally assessed as of Least Concern (LC) for the IUCN Red List (Botanical Gardens Conservation International (BGCI) and IUCN SSC Global Tree Specialist Group 2018).

#### Common names.

China. da hua qie (Zhang et al. 1994).

#### Discussion.

Although first described from Hong Kong, *S.wrightii* is a plant of the eastern slopes of the Andes in Bolivia. It is a member of the Crinitum clade (sensu Stern et al. 2011; Gagnon et al. 2022), characterised by large flowers and fruits, and usually large, repand leaves. In tropical Asia it is widely planted as a street and shade tree, and also sometimes as living fencing. It is not known to naturalise and is rarely collected out of cultivation.

Bentham (1861) did not state where he had examined herbarium material for his “Flora of Hong-Kong”, and subsequent authors have assumed the Kew sheet of *Wright 849* is the holotype for this name (e.g., Vorontsova and Knapp 2016). This is likely to have been the only material Bentham saw, as Wright’s collections were distributed from the Smithsonian, but since he did not cite a single collection in a single herbarium it is necessary to designate a lectotype for this widely used name (McNeill 2014); it is surprising it has not been done inadvertently previously. We lectotypify *S.wrightii* here in conformity with previous usage, selecting the Kew duplicate of *Wright 849* as the lectotype (K000545757).

**Table 3.** Preliminary threat status for native spiny solanums of tropical Asia (introduced species not assessed here, nor is the cultivated *S.melongena*). Also see individual species treatments for more details; *S.comitis*, *S.kachinense*, *S.putii* and *S.sulawesi* are known only from the types or too few collections to calculate a preliminary assessment and are therefore assessed as Data Deficient (DD). The highly fragmented island nature of these species’ distributions means more detailed assessments should be undertaken in the future once local herbaria are consulted (e.g., Delves 2021).

Species	EOO (km^2^)	AOO (km^2^)	Assessment
*Solanumarundo* Mattei	1,277,240	216	LC
*Solanumbarbisetum* Nees	1,244,077	248	LC
*Solanumcamranhense* Dy Phon & Hul	555	16	EN
*Solanumcomitis* Dunal	n/a	n/a	DD
*Solanumcordatum* Forssk.	Formally assessed for the IUCN Red List		LC (Knapp 2021)
*Solanumcyanocarphium* Blume	1,492,571	112	LC
*Solanumdeflexicarpum* C.Y.Wu & S.C.Huang	65,775	16	NT
*Solanumdunalianum* Gaudich.	887,043	100	LC
*Solanumforskalii* Dunal	Formally assessed for the IUCN Red List		LC (Knapp 2021)
*Solanumgiganteum* Jacq.	Formally assessed for the IUCN Red List		LC (BGCI and IUCN SSC 2020)
*Solanumgraciliflorum* Dunal	131,936	16	NT
*Solanumharmandii* Bonati	n/a	n/a	DD
*Solanumhovei* Dunal	173,911	564	LC
*Solanuminsanum* L.	10,914,652	1,052	LC
*Solanuminvolucratum* Blume	1,868,808	216	LC
*Solanumkachinense* X.Aubriot & S.Knapp	n/a	n/a	DD
*Solanumlasiocarpum* Dunal	6,950,615	1,032	LC
*Solanummelongena* L.	cultivated		Not assessed
*Solanummiyakojimense* T.Yamaz. & Takushi	641	40	EN
*Solanummultiflorum* Roth	669,122	324	LC
*Solanumnienkui* Merr. & Chun	64,999	72	NT
*Solanumpeikuoense* S.S.Ying	10,013	44	VU
*Solanumpoka* Dunal	1,202,780	68	LC
*Solanumpraetermissimum* Kerr ex Barnett	1,191,003	88	LC
*Solanumprocumbens* Lour.	1,575,888	164	LC
*Solaumpseudosaponaceum* Blume	2,387,194	160	LC
*Solanumpubescens* Willd.	996,977	272	LC
*Solanumputii* Kerr ex Barnett	n/a	n/a	DD
*Solanumretrorsum* Elmer	115,321	48	NT
*Solanumrobinsonii* Bonati	610	20	EN
*Solanumschefferi* F.Muell.	1,012,899	32	LC
*Solanumsulawesi* X.Aubriot & S.Knapp	n/a	n/a	DD
*Solanumtorvoideum* Merr. & L.M.Perry	2,372,285	212	LC
*Solanumtrilobatum* L.	2,326,480	528	LC
*Solanumvagum* Nees	75,338	76	LC
*Solanumviolaceum* Ortega	10,078,363	988	LC
*Solanumwightii* Nees	7,899,801	336	VU

#### Specimens examined.

See Suppl. materials 1–3.

##### ﻿Doubtful and suppressed names

*Solanumcanescens* Blume, Bijdr. Fl. Ned. Ind. 13: 701. 1826. No specimens or locality cited; Blume cited no specimens or localities, and it is unclear if his description is a re-description of *S.incanum* sensu Forssk. (= *S.incanum* L., see Vorontsova and Knapp 2016), or if he based it on Indonesian specimens. The protologue states “(aff. *S.sancto*; *S.incanum* Forssk, forsan eadem species)” perhaps indicating this is a replacement name for the unpublished *S.incanum* sensu Forssk., a bibliographic reference to *S.incanum* L. and not intended as a new name. No material in Leiden is annotated with this name, nor have we found any other material that Blume used annotated as *S.canescens*. We are treating this name as doubtful.

*Solanumferox* L., Sp. Pl. ed. 2: 267. 1762, nom. utique rej. (suppressed name; Knapp 2011, see Shenzhen Code Appendices at https://naturalhistory2.si.edu/botany/codes-proposals/index.cfm).

*Solanumindicum* L., Sp. Pl. 187. 1753, nom. utique rej. (suppressed name; Hepper 1978, see Shenzhen Code Appendices at https://naturalhistory2.si.edu/botany/codes-proposals/index.cfm).

*Solanumsodomeum* Dunal, Prodr. [A. P. de Candolle] 13(1): 365. 1852, nom. utique rej. (suppressed name; Hepper 1978, see Shenzhen Code Appendices at https://naturalhistory2.si.edu/botany/codes-proposals/index.cfm).

##### ﻿“Names” (designations) not validly published

*Solanumalbum* Noronha, Verh. Batav. Genootsch. Kunst. 5(Art. 4): 84. 1791, nomen nudum, name in list, no description or diagnosis (Art. 38.1). [Page 84 in 1827 exact reprint, read 1790, printed 1791] = *S.melongena* L.

*Solanumblattaroioides* Koen. ex Dunal, Prodr. [A. P. de Candolle] 13(1): 127. 1852, pro syn. *Solanumpubescens* Willd. = *S.pubescens* Willd. (herbarium name on *König s.n.* specimen of *S.pubescens* [BM000900094]).

*Solanumcanaranum* Miq. ex C.B.Clarke, Fl. Brit. India [J. D. Hooker] 4(10): 236. 1883, pro syn. *Solanumtrilobatum* L. = *S.trilobatum* L.

*Solanumcoccineum* Dunal, Prodr. [A. P. de Candolle] 13(1): 310. 1852, pro syn. SolanumindicumNeesvar.sinuato-lobatum Dunal = *S.violaceum* Ortega

*Solanumdiffusum* Roxb. ex Wall., Hort. Bengal. 17. 1814, nomen nudum, no description or diagnosis (Art. 38.1) = *Solanumvirginianum* L. (i.e., K001116578)

*Solanumferox* Jungh. ex Miq., Fl. Ned. Ind. 2: 650. 1857, pro syn. *Solanumjunghuhnii* Miq. = *S.violaceum* Ortega

*Solanumfuscum* B.Heyne ex Wall., Numer. List [Wallich] no. 2622b. 1831, nomen nudum [probably from Heyne’s herbarium], no description or diagnosis (Art. 38.1) = *S.trilobatum* L.

*Solanumindicum* Roxb., Hort. Bengal. 17. 1814, nomen nudum, no description or diagnosis (Art. 38.1); identity uncertain, could apply to any number of species from the region; probably a reference to *S indicum* L. (nom. utique rej.)

*Solanumintermedium* Dunal, Prodr. [A. P. de Candolle] 13(1): 311. 1852, pro syn. *Solanumhovei* Dunal (herbarium name on holotype specimen of *S.hovei*, *Hove s.n.* [BM000900293]) = *S.hovei* Dunal

*Solanuminvolucratum* Kurz, Forest Fl. Burma 2: 224. 1877, nomen nudum, no description or diagnosis (Art. 38.1), probably not intended as a new name; probably = *S.barbisetum* Nees

*Solanumlividum* Willd. ex Dunal, Prodr. [A. P. de Candolle] 13(1): 310. 1852, pro syn. SolanumindicumNeesvar.sinuato-lobatum Dunal = *S.violaceum* Ortega

*Solanumlongum* Roxb., Hort. Bengal. 16. 1814, nomen nudum, name in list, no description or diagnosis (Art. 38.1) = *Solanummelongena* L.

SolanummelongenaL.subsp.agrestis Filov, Kult. Fl. SSSR (Zhukovskii) 10: 318. 1958., not validly published, no description or diagnosis in Latin (Art. 39.1) = *S.melongena* L.

SolanummelongenaL.var.agrestis Dikii, Trudy Prikl. Bot. Genet. Selek. 88: 105. 1984. Not validly published, no description, diagnosis or type (seems to be homotypic with his subsp. agrestis) = *S.melongena* L.

SolanummelongenaL.var.americanum Filov, Kult. Fl. SSSR (Zhukovskii) 10: 317. 1958., not validly published, no description or diagnosis in Latin (Art. 39.1); as SolanummelongenaL.subsp.meridionaleFilovvar.americanum Filov = *S.melongena* L.

SolanummelongenaL.var.arabicum Filov, Kult. Fl. SSSR (Zhukovskii) 10: 318. 1958., not validly published, no description or diagnosis in Latin (Art. 39.1); as SolanummelongenaL.subsp.subspontaneumFilovvar.arabicum Filov = *S.melongena* L.

SolanummelongenaL.var.azerbaijanicum Filov, Kult. Fl. SSSR (Zhukovskii) 10: 313. 1958., not validly published, no description or diagnosis in Latin (Art. 39.1); as SolanummelongenaL.subsp.occidentaleHaz.var.azerbaijanicum Filov = *S.melongena* L.

SolanummelongenaL.var.bulgaricum Filov, Kult. Fl. SSSR (Zhukovskii) 10: 313. 1958., not validly published, no description or diagnosis in Latin (Art. 39.1); as SolanummelongenaL.subsp.occidentaleHaz.var.bulgaricum Filov = *S.melongena* L.

SolanummelongenaL.var.europaeum Filov, Kult. Fl. SSSR (Zhukovskii) 10: 314. 1958., not validly published, no description or diagnosis in Latin (Art. 39.1); as SolanummelongenaL.subsp.occidentaleHaz.var.europaeum Filov = *S.melongena* L.

SolanummelongenaL.var.giganteum (Alef.) Dikii, Trudy Prikl. Bot. Genet. Selek. 88: 105. 1984, as “*gigantea*”; not a new name, re-publication of Alefeld’s name at the same rank. = *S.melongena* L.

SolanummelongenaL.var.insanum (L.) Filov, Kult. Fl. SSSR (Zhukovskii) 10: 321. 1958, not validly published, no full and direct references to original place of publication (Art. 41.5); also nom. illeg., later homonym as SolanummelongenaL.subsp.agrestisFilovvar.insanum (L.) Filov = *S.insanum* L.

SolanummelongenaL.var.leucoum (Alef.) Dikii, Trudy Prikl. Bot. Genet. Selek. 88: 105. 1984; not a new name, re-publication of Alefeld’s name at the same rank. = *S.melongena* L.

SolanummelongenaL.subsp.meridionale Filov, Kult. Fl. SSSR (Zhukovskii) 10: 315. 1958., not validly published, no description or diagnosis in Latin (Art. 39.1) [cites as syn. var. insanum Nees, var. esculentum Bailey] = *S.melongena* L.

SolanummelongenaL.subsp.orientale Filov, Kult. Fl. SSSR (Zhukovskii) 10: 306. 1958., not validly published, no description or diagnosis in Latin (Art. 39.1) = *S.melongena* L.

SolanummelongenaL.var.ovigera Lam., Tabl. Encycl. 2: 19. 1794, the name that occurs in the agricultural and some other indices, is not presented as a binomial in Lamarck (1794). The protologue reads “β Idem fructu ovato, alb. Melongenaovigera. Ex Hort. Paris”. We do not consider this to be the coining of a new name (Art. 23). A specimen in the Lamarck herbarium at P (P00357638) labelled “Solanummelongenaovigera/Dict. No. 43” in Lamarck’s hand is probably original material associated with this polynomial. = *S.melongena* L.

SolanummelongenaL.var.ovigera Pers., Syn. Pl. [Persoon] 1: 221. 1805, this name that occurs in some indices was not published as a binomial, the protologue reads “β Melongena (ovigera)”, along with citations of Tournefort (1700), Miller (1768) and Lamarck (“encl.”) We do not consider this name to be validly published (Art. 23). = *S.melongena* L.

SolanummelongenaL.var.pekinense Filov, Kult. Fl. SSSR (Zhukovskii) 10: 310. 1958., not validly published, no description or diagnosis in Latin (Art. 39.1); as SolanummelongenaL.subsp.orientaleFilovvar.pekinense Filov= *S.melongena* L.

SolanummelongenaL.var.palestinicum Filov, Kult. Fl. SSSR (Zhukovskii) 10: 316. 1958., not validly published, no description or diagnosis in Latin (Art. 39.1); as SolanummelongenaL.subsp.meridionaleFilovvar.palestinicum Filov = *S.melongena* L.

SolanummelongenaL.var.racemosum Filov, Kult. Fl. SSSR (Zhukovskii) 10: 320. 1958., not validly published, no description or diagnosis in Latin (Art. 39.1); as SolanummelongenaL.subsp.agrestisFilovvar.racemosum Filov = *S.melongena* L.

SolanummelongenaL.var.stenoleucum (Alef.) Dikii, Trudy Prikl. Bot. Genet. Selek. 88: 104. 1984, as “*stenoleuca*”; not a new name, re-publication of Alefeld’s name at the same rank.

SolanummelongenaL.subsp.subspontaneum Filov, Kult. Fl. SSSR (Zhukovskii) 10: 318. 1958., not validly published, no description or diagnosis in Latin (Art. 39.1) [cites in synonymy subsp. italo-arabicum Filov, perhaps a herbarium name?] = *S.melongena* L.

SolanummelongenaL.var.variegatum (Alef.) Dikii, Trudy Prikl. Bot. Genet. Selek. 88: 105. 1984, as “*variegata*”; not a new name, re-publication of Alefeld’s name with spelling correction. = *S.melongena* L.

SolanummelongenaL.var.violaceum (Alef.) Dikii, Trudy Prikl. Bot. Genet. Selek. 88: 105. 1984, as “*violacea*”; not a new name, re-publication of Alefeld’s name at the same rank. = *S.melongena* L.

*Solanumnelsonii* Zipp. ex Span., Linnaea 15: 337. 1841, pro syn. *Solanumindicum* L. = *S.violaceum* Ortega.

SolanumovigerumDunalvar.album Sweet, Hort. Brit., ed. 2: 387. 1830, nomen nudum, listed in table, no description or diagnosis (Art. 38.1) = *S.melongena* L.

SolanumovigerumDunalvar.luteum Sweet, Hort. Brit., ed. 2: 387. 1830, nomen nudum, listed in table, no description or diagnosis (Art. 38.1) = *S.melongena* L.

SolanumovigerumDunalvar.ruber Sweet, Hort. Brit., ed. 2: 387. 1830, nomen nudum, listed in table, no description or diagnosis (Art. 38.1) probably = *S.aethiopicum* L.

SolanumovigerumDunalvar.violaceum Sweet, Hort. Brit., ed. 2: 387. 1830, nomen nudum, listed in table, no description or diagnosis (Art. 38.1) = *S.melongena* L.

*Solanumovoideum* Zipp. ex Miq., Fl. Ned. Ind. 2: 647. 1857, pro syn. SolanumferoxL.var.involucratum (Blume) Miq. = *S.involucratum* Blume

*Solanumpeikuoense* S.S.Ying, Quarterly J. Exp. For. Natl. Taiwan Univ. 11(1): 14. 1997, as “peikuoensis”, name not validly published; the words holotype or the equivalent were not used contrary to Art. 40.6. = *S.peikuoense* S.S.Ying in. X.Aubriot & S.Knapp

*Solanumprostratum* Raeusch., Nomencl. Bot. 60. 1797, nomen nudum, no description or diagnosis (Art. 38.1); identity uncertain.

*Solanumpubescens* B.Heyne ex Walp., Repert. Bot. Syst. (Walpers) 3: 83. 1844, pro syn. *Solanumindicum* L. var. δ = *S.pubescens* Willd.

*Solanumquercifolium* Banks ex Dunal, Prodr. [A. P. de Candolle] 13(1): 345. 1852, pro syn. *Solanumpoka* Dunal = *S.pseudosaponaceum* Blume (herbarium name on *Banks & Solander s.n.* specimen of *S.pseudosaponaceum* [BM000886238]).

*Solanumracemosum* Noronha, Verh. Batav. Genootsch. Kunst. 5(Art. 4): 26. 1790, nomen nudum, no description or diagnosis (Art. 38.1); identity uncertain, probably misuse of *S.racemosum* Jacq., a Caribbean species that is a synonym of *S.bahamense* L.

*Solanumreflexispinosum* Merr., Fl. Arfak Mts. [Gibbs] 178. 1917, pro syn. *Solanumretrorsum* Elmer (herbarium name on a specimen of *S.retrorsum*, *Williams 1074* [K000195907]) = *S.retrorsum* Elmer

*Solanumserpentinum* Noronha, Verh. Batav. Genootsch. Kunst. 5(Art. 4): 84. 1791, nomen nudum, name in list, no description or diagnosis (Art. 38.1). = *S.melongena* L.

*Solanumserpentinum* Desf., Cat. Pl. Horti Paris 397. 1829, nomen nudum; on page 115 in list as “serpentinum H.”; on page 397 as “Solanumserpentinum/ Fructus elongatus serpentinus. An varietas S. Melongenae?”; refers to a horticultural name and not with the intention of coining a new name, this description is much less detailed than those in which Desfontaines did intend new names in this work. = *S.melongena* L.

SolanumsurattenseBurm.f.var.awanicum Yousef, Mir A.Khan & Shinwari, Pakistan J. Bot 41(5) 2099. 2009, not validly published, no description or diagnosis in Latin (Art. 39.1). = *S.virginianum* L.

*Solanumtomentosum* Herb. Hasselt. ex Miq., Fl. Ned. Ind. 2: 654. 1857, pro syn. *Solanumundatum* Lam. = *S.insanum* L.

*Solanumundatum* Poir., Encycl. (Lamarck) 4: 301. 1797, not a new name, Poiret makes clear reference to Lamarck’s protologue.

*Solanumvagum* B.Heyne ex Wall., Numer. List [Wallich] n. 2624. 1831, nomen nudum, no description or diagnosis (Art. 38.1) = *Solanumvagum* Nees.

*Solanumviolaceum* DC. ex Dunal, Prodr. [A. P. de Candolle] 13(1): 359. 1852, pro syn. SolanumundatumLam.var.violaceum Dunal = *S.insanum* L.

*Solanumvincentii* Delile ex Dunal, Prodr. [A. P. de Candolle] 13(1): 310. 1852, pro syn. SolanumindicumNeesvar.sinuato-lobatum Dunal = *S.violaceum* Ortega

*Solanumvirginianum* Jacq., Icon. Pl. Rar. [Jacquin] 2: 11, t. 332. 1792, not a new name, Jacquin (1792) makes clear reference to the Linnaean protologue.

## Supplementary Material

XML Treatment for Solanum subgenus Leptostemonum

XML Treatment for Solanum aculeatissimum

XML Treatment for Solanum aethiopicum

XML Treatment for Solanum arundo

XML Treatment for Solanum barbisetum

XML Treatment for Solanum camranhense

XML Treatment for Solanum capsicoides

XML Treatment for Solanum chrysotrichum

XML Treatment for Solanum comitis

XML Treatment for Solanum cordatum

XML Treatment for Solanum cyanocarphium

XML Treatment for Solanum deflexicarpum

XML Treatment for Solanum dunalianum

XML Treatment for Solanum elaeagnifolium

XML Treatment for Solanum forskalii

XML Treatment for Solanum giganteum

XML Treatment for Solanum graciliflorum

XML Treatment for Solanum harmandii

XML Treatment for Solanum hovei

XML Treatment for Solanum insanum

XML Treatment for Solanum involucratum

XML Treatment for Solanum jamaicense

XML Treatment for Solanum kachinense

XML Treatment for Solanum lasiocarpum

XML Treatment for Solanum macrocarpon

XML Treatment for Solanum mammosum

XML Treatment for Solanum melongena

XML Treatment for Solanum miyakojimense

XML Treatment for Solanum multiflorum

XML Treatment for Solanum nienkui

XML Treatment for Solanum peikuoense

XML Treatment for Solanum poka

XML Treatment for Solanum praetermissum

XML Treatment for Solanum procumbens

XML Treatment for Solanum pseudosaponaceum

XML Treatment for Solanum pubescens

XML Treatment for Solanum putii

XML Treatment for Solanum retrorsum

XML Treatment for Solanum robinsonii

XML Treatment for Solanum robustum

XML Treatment for Solanum schefferi

XML Treatment for Solanum sisymbriifolium

XML Treatment for Solanum sulawesi

XML Treatment for Solanum torvoideum

XML Treatment for Solanum torvum

XML Treatment for Solanum trilobatum

XML Treatment for Solanum vagum

XML Treatment for Solanum viarum

XML Treatment for Solanum violaceum

XML Treatment for Solanum virginianum

XML Treatment for Solanum wightii

XML Treatment for Solanum wrightii

## ﻿Index to Numbered Collections (tropical Asia only)

Only first collectors in collections from the area treated in this revision (tropical Asia) made by two or more collectors are listed here. Collections by anonymous collectors without date or other identifying features are not listed here. All collections seen are listed in this format in Suppl. material 1, and full collector strings can be found in the Suppl. materials 2, 3 searchable files. These files are also available on the NHM Data Portal (https://doi.org/10.5519/0rqfzvgd).

Aban Gibot SAN-94555 (lasiocarpum); SAN-99310 (lasiocarpum); SAN-99536 (lasiocarpum).

Abdul, K. bin A. 2854 (lasiocarpum).

Abedin, S. 9419 (virginianum).

Abraham, V. 2 (insanum); 176 (trilobatum); 239 (trilobatum); 279 (insanum).

Abulaila, K. JOR 3 -1 (elaeagnifolium).

Aclaud, R.D. ACK 793 (hovei).

Adduru, M. 62 (retrorsum).

Aët 581 (lasiocarpum).

Ahamed, S.M. KFP-282 (violaceum).

Ahern’s collector 2161 (torvoideum); 2436 (lasiocarpum).

Ahmad, S. SA-483 (lasiocarpum).

Ahmed, H. 9842 (melongena).

Ahuja, B.S. 19607 (multiflorum); 47366 (hovei).

Ahuja, K.K. 87297 (hovei).

Aitchison, J.E.T. 482 (virginianum); 521 (cordatum).

Alcasid, G.L. 1885 (pseudosaponaceum).

Ali, S.I. 1183 (cordatum).

Alleizette, C. d’ 5 (violaceum).

Alston, A.H.G. 448 (violaceum); 14609 (lasiocarpum); 16158 (retrorsum).

Ambriansyah, A.A. 193 (jamaicense); 657 (lasiocarpum); 687 (melongena); 1656 (jamaicense); 3068 (jamaicense).

Amin, A. SAN-111132 (lasiocarpum); SAN 117381 (lasiocarpum).

Amin, G. SAN-96560 (lasiocarpum).

Anang 88 (lasiocarpum).

Anders, O. 2978 (virginianum); 3560 (virginianum); 4850 (virginianum).

Anderson, E.F. 5233 (torvum).

Anderson, T. 1021 (insanum).

Andrews, C. 660 (sisymbriifolium).

Andrews, C.W. 97 (involucratum); 99 (involucratum).

Andrews, F.W. A 328 (trilobatum); A 355 (sisymbriifolium).

Anglade, L. 824 (pubescens).

Anilkumarin, Y. 951 (multiflorum).

Annandale, N. 1288 (trilobatum).

Annari, R. SC-67984 (insanum).

Ansari, M.Y. WC-63735 (insanum); 67512 (hovei); 87373 (hovei); 87766 (hovei); 94279 (hovei); 99990 (hovei); 104715 (hovei); 122027 (hovei); 124133 A (hovei); 127073 (hovei); 127202 (hovei); 146866 (multiflorum).

Ansari, R. ANC-1353 (torvum).

Anstead, R.D. 2 (torvum); 120 (trilobatum); 146 (torvum).

Antony, V.T. 78 (capsicoides); 221 (multiflorum).

Anupama, K. AP 179 (trilobatum); AP 299 (virginianum); 1P 729 (trilobatum).

Argent, G.C.G. 108279 (lasiocarpum).

Aron, C.M. CC-2702 (insanum).

Arora, C.M. NC-37130 (violaceum); UK-37131 (chrysotrichum); NC-38865 (insanum).

Aroy, R.K. 11677 (multiflorum); 12130 (multiflorum); 12168 (multiflorum); 12229 (hovei).

Artyushenko, Z.T. 238 (virginianum).

Arulappan, C. 67 (trilobatum).

Aryes, P.B. t 132 (violaceum).

Asrana, R. RA 2887 (hovei); RA 3171 (hovei).

Aswal, B.S. NC-40178 (insanum).

Auglade, L. 2175 (violaceum).

Aung, M.M. 92183 (torvum); 92372 (viarum).

Averyanov, L. AL-482 (robinsonii); VH 1090 (insanum); VH 2739 (violaceum).

Aye, T.T. 20611 (melongena); 21276 (violaceum).

B S 1102 (trilobatum).

Babu, C.R. NC-34959 (chrysotrichum); NC-35284 (violaceum).

Backer, C.A. 6910 (violaceum); 17065 (melongena); 36562 (involucratum); 37259 (involucratum).

Badami, P.S.K. 114 (cordatum).

Bai, B. 417 (deflexicarpum).

Bakhuizen van den Brink Jr, R.C. 3274 (involucratum); 4940 (involucratum); 7111 (involucratum).

Bakia, K. 468 (lasiocarpum).

Baladh, S. NC-67100 (violaceum).

Balakrishnan, N.P. ANC-150 (torvum); 614 (insanum); ANC-738 (torvum); ANC-985 (virginianum); ANC-985 (virginianum); ANC-1308 (torvum); ANC-1312 (violaceum); ANC-1347 (torvum); ANC-3589 (torvum); ANC-5503 (violaceum); ANC-5504 (violaceum); SC-10094 (elaeagnifolium); SC-11388 (insanum); 40288 (violaceum).

Balamani, B. BB 449 (hovei).

Balansa, B. 943 (procumbens); 945 (violaceum); 948 (insanum); 3744 (procumbens); 4571 (violaceum).

Balfour, I.B. 1007 (insanum); 1252 (violaceum); 1324 (melongena).

Banarjee, L.K. 604 (insanum).

Banerjee, B.C. CNH-11328 (capsicoides); CNH-11516 (capsicoides).

Banerjee, R.N. 85 (trilobatum); 89 (trilobatum); CNH-18621 (torvum); CNH-18697 (torvum).

Banerjee, S.P. 64 (violaceum).

Bansjee, L.K. CNH-600 (virginianum).

Bara, T. 37 (dunalianum).

Barber, C.A. 132 (trilobatum); 2188 (multiflorum); 3203 (vagum); 4909 (trilobatum); 6490 (pubescens); 7114 (trilobatum); 8079 (insanum).

Barbon, E.B. PPI 1760 (torvum); 1976 (torvum); 2178 (jamaicense); 5935 (torvum); PPI 8208 (torvum); 8271 (torvum); 8590 (torvum); 18814 (torvum).

Barclay, C. 1311 (robustum).

Barnard, D.E. SoKUB 4 (virginianum).

Barnes, E. 92 (multiflorum); 281 (giganteum); 301 (torvum); 352 (multiflorum); 431 (torvum); 439 (multiflorum); 452 (multiflorum); 589 (multiflorum); 1062 (insanum).

Barry, J.P. 62 (camranhense); 63 (camranhense).

Bartholomew, B. 3955 (torvum); 3965 (viarum).

Bartlett, H.H. 437 (violaceum); 440 (violaceum); 8407 (violaceum); 14717 (torvum); 15140 (miyakojimense); 15144 (insanum); 15385 (miyakojimense).

Basu, P. ANC-6618 (torvum); ANC-6866 (viarum); ANC-6869 (lasiocarpum); CNH-13235 (melongena); CNH-13324 (melongena).

Bateson, G. 146 (lasiocarpum).

Batianoff, G.N. 505100 (melongena).

Beauvais, J. 20 (violaceum).

Beddome, R.H. 5477 (torvum); 5478 (pubescens); 5489 (giganteum); 5490 (giganteum); 5493 (lasiocarpum); 5494 (wrightii); 5495 (multiflorum); 5496 (melongena); 5498 (insanum); 5499 (melongena); 5500 (violaceum); 5941 (lasiocarpum); 5942 (lasiocarpum).

Béguin, D. 1037 (lasiocarpum).

Bélanger, C.P. 380 (hovei).

Belcher, C. 20 (torvum); 99 (virginianum).

Belcher, R.O. 390 (torvum); 561 (torvum); 748 (viarum); 848 (violaceum).

Bell, A.S. Mrs 40 (melongena); 119 -B (insanum); 863 (insanum).

Bennet, S.S.R. 482 (trilobatum); 1064 (trilobatum).

Bernardi, L. 14298 (trilobatum); 15327 (trilobatum).

Bernstein, J.H. 133 (lasiocarpum).

Beusekom, C.F. van 3772 (lasiocarpum).

Bhargava, N. ANC-1711 (lasiocarpum); ANC-1930 (violaceum); ANC-2309 (torvum); ANC-2456 (viarum); ANC-3325 (torvum); ANC-4103 (torvum); ANC-5639 (melongena); ANC-5895 (lasiocarpum); ANC-6302 (lasiocarpum); ANC-6318 (torvum); ANC-6539 (torvum); NC-51506 (insanum); NC-63716 (chrysotrichum).

Bhargavan, N.P. ANC-1784 (torvum).

Bhargavan, P. 90001 (multiflorum).

Bhattacharyya, U.C. NC-13625 (insanum); 15236 (insanum); NC-17688 (insanum); NC-37674 (violaceum).

Bhide, R.K. 877 (hovei).

Billore, K.V. 110573 (hovei); 110948 (hovei); 111709 (hovei); 111928 (hovei); 112933 (hovei); 113081 (hovei); 113582 (hovei); 115473 (hovei); 115538 (hovei); 116745 (hovei).

Binh, B.T. 746 (torvum).

Bis Ram 229 (violaceum); 274 (violaceum).

Biswas, K. 559 (barbisetum); 1380 (mammosum); 3731 (sisymbriifolium); 5756 (torvum); 5784 (viarum); 6550 (viarum); 6781 (torvum).

Biswas, S. 560 (lasiocarpum); 893 (lasiocarpum).

Blasco, F. 45 (violaceum); 2089 (trilobatum); 2113 (insanum); 2573 (pubescens).

Blatter, E. 89 (multiflorum); 2115 (hovei).

Bloembergen, S. 3439 (lasiocarpum).

Bodinier, E.M. 1098 (lasiocarpum).

Bohs, L. 3519 (chrysotrichum).

Bois, D.G.J.M. 93 (violaceum); 226 (procumbens); 475 (torvum); 531 (violaceum).

Bole, P.V. 1628 (hovei); 2388 (hovei).

Bombay Natural History Society 2016 (pubescens).

Bon, R.P. 96 (violaceum); 120 (procumbens); 196 (insanum); 350 (procumbens); 506 (lasiocarpum); 598 (procumbens); 642 (procumbens); 680 (lasiocarpum); 1741 (insanum); 1742 (lasiocarpum); 2193 (procumbens); 3873 (praetermissum); 4017 (violaceum); 4442 (lasiocarpum); 5071 (insanum).

Bor, N.L. 18478 (barbisetum).

Borden, T.E. 1948 (insanum); 2336 (torvoideum).

Borgmann, E. 322 (torvoideum).

Borssum Waalkes, J. van 2695 (lasiocarpum).

Botanical Expedition to Himalaya 1990 90-60015 (violaceum).

Botanical Expedition to Far West Nepal 1991 91-94137 (violaceum).

Bouamanivong, S. 6 (torvum).

Bourell, M. 2372 (torvum); 2390 (torvum).

Bourne, A.G. 456 (giganteum); 1643 (violaceum); 2809 (giganteum); 2810 (violaceum); 3308 (virginianum); 5055 (pubescens); 5146 (pubescens); 6218 (torvum); 6293 (torvum).

Bourne, E.T. 3168 (trilobatum).

Bouton, M. 5 (violaceum).

Bowes-Lyon, S.A. 6010 (torvum).

Bradtke, W. 62 (dunalianum); 184 (dunalianum).

Bragg, K. 81 (torvum); 85 (melongena).

Brass, L.J. 3815 (dunalianum); 5411 (torvoideum); 7467 (lasiocarpum); 8514 (dunalianum); 11590 (torvoideum); 22030 (lasiocarpum); 23950 (schefferi); 23962 (lasiocarpum); 24330 (schefferi); 27452 (dunalianum); 27637 (schefferi); 28146 (lasiocarpum); 28809 (lasiocarpum).

Britton, B.B. 396 (lasiocarpum).

Britton, M.E. 62 (dunalianum).

Buchanan-Hamilton, F. 570 -2 (insanum); 570 -1 (insanum); 571 (insanum); 573 (violaceum).

Buesekom, C.F. van 3772 (lasiocarpum).

Buller, R.H. REP-19845 (cordatum).

Bullock, ? 13816 (procumbens).

Bullock, A.A. 646 (torvum).

Bulmer, R.N.H. 139 (torvoideum).

Bult, M. van de 174 (barbisetum).

Bunnemeyer, H.A.B. 2786 (poka); 4882 (lasiocarpum); 7261 (lasiocarpum); 7975 (torvoideum); 11592 (involucratum); 11732 (poka); 11813 A (poka); 11980 (lasiocarpum); 12117 (poka).

Bunpheng, D. 224 (violaceum).

Burkill, H.M. 2253 (torvum); 2854 (torvum).

Burkill, I.H. 33280 (hovei); 36313 (violaceum).

Burley, J.S. 123 (lasiocarpum); 1999 (jamaicense); 3625 (sulawesi).

Burmann, J. 41 (virginianum).

Burnemeyer, ? 12117 (pseudosaponaceum).

Burtt, B.L. 710 (violaceum); B-1084 (cordatum).

Buwalda, P. 4147 (schefferi); 4309 (lasiocarpum); 4633 (lasiocarpum).

Buysman, M. 147 (involucratum).

Cadet, T. 1493 (robustum); 2386 (violaceum); 4684 (torvum).

Calléry, J.M.M. 28 29 (torvum); 812 (violaceum).

Campbell, J. 79 (violaceum).

Carles, W.R. 157 (insanum).

Carr, C.E. 12747 (lasiocarpum); 12748 (torvoideum); 13198 (torvoideum); 15417 (torvoideum); 15613 (lasiocarpum).

Carrick, J. 1463 (torvum).

Carter, H.G. EB-301 (melongena).

Castle-Smith, P.M. 80 (torvum).

Castro, P. 1360 (torvum); 1504 (cyanocarphium).

Cavalerie, P.J. 2722 (aculeatissimum); 4092 (violaceum); 4344 (violaceum).

Chakraborty, G. ANC-12336 (lasiocarpum).

Chakraborty, P. ANC-1232 (insanum); BSHC-1941 (virginianum); ANC-2142 (insanum).

Chandra, S. 5 (trilobatum).

Chandrabose, M. SC-28500 (insanum); SC-28526 (insanum); 28533 (trilobatum); SC-28977 (elaeagnifolium); 30514 (trilobatum); 30631 (trilobatum); 31106 (trilobatum); 51683 (pubescens).

Chang, C.C. 99063 (miyakojimense).

Chang, C.E. 9059 (miyakojimense).

Chantaranothai, P. 90 185 (lasiocarpum); 90 448 (barbisetum); 90 449 (involucratum); 1161 (torvum).

Charnpareet 9430 (insanum).

Chassagne, F.C. FC 138 (cyanocarphium).

Chatterjee, A.C. 3 (melongena); 38 (melongena); 48 (insanum); 273 (melongena).

Cheng Shaoqin 13537 (praetermissum); 17170 (praetermissum).

Cheng, K.C. CL 929 (cyanocarphium).

Cheng, S.K. CL-1122 (torvum); CL-1186 (lasiocarpum); CL-1367 (trilobatum).

Cheo, H.C. 96 (melongena).

Cheo, T.Y. 109 (melongena).

Cherian, P.J. 106290 (hovei); 109394 (hovei); 111106 (hovei); 111397 (hovei); 111574 (hovei); 112767 (hovei).

Chevalier, A.J.B. 29028 (violaceum); 29170 (procumbens); 29547 (procumbens); 29563 (violaceum); 30724 (violaceum); 38932 (camranhense); 39251 (involucratum); 39391 (violaceum); 39417 (violaceum); 39496 (procumbens); 39497 (procumbens); 40321 (violaceum); 40449 (insanum); 40551 (violaceum); 40562 (nienkui).

Chew, W.L. 1667 (lasiocarpum); 7087 (lasiocarpum).

Chiao, C.Y. 2972 (melongena); 14490 (melongena).

Chin, S.C. 2553 (lasiocarpum); 3559 (lasiocarpum).

Chinese collector 4500 (procumbens).

Ching, R.C. 5271 (violaceum); 7640 (pseudosaponaceum).

Chiu, S.F. H-4 (violaceum).

Chongko, S. 114 (lasiocarpum).

Chorian, J. WC-106704 (insanum).

Chorley, M. 3 (insanum).

Chow, H.-.C. 1335 (melongena).

Chow, K.S. 78074 (torvum); 78313 (nienkui); 78465 (capsicoides); 78889 (nienkui); 78904 (nienkui).

Chowdhery, H.J. NC-75822 (violaceum); NC-75825 (violaceum).

Chowdhury, A.M. 13 (cordatum).

Chuang, T.I. 2420 (miyakojimense).

Chun, N.K. 40175 (lasiocarpum); 43319 (nienkui); 43473 (insanum); 44522 (nienkui); 44631 (procumbens).

Chun, S.H. 11876 (praetermissum); 13537 (praetermissum).

Chun, W.Y. 6713 (melongena).

Chun, Z.C. 50421 (pseudosaponaceum).

Chung, H.H. 7532 (violaceum).

Cid, F. 81 (lasiocarpum).

Clarke, C.B. 37 (insanum); 4626 (lasiocarpum); 4864 A (barbisetum); 6611 (lasiocarpum); 6681 (lasiocarpum); 6694 (violaceum); 6695 (torvum); 6902 (melongena); 9057 (barbisetum); 10728 A (giganteum); 10772 (wrightii); 10793 (wightii); 11701 A (virginianum); 11701 B (virginianum); 11764 (violaceum); 11772 (barbisetum); 11923 (barbisetum); 11935 A (barbisetum); 17369 (lasiocarpum); 26478 B (violaceum); 37071 e (trilobatum); 37071 F (trilobatum); 37071 D (trilobatum); 37071 A (trilobatum); 37414 (aculeatissimum); 38107 C (barbisetum); 38359 A (aculeatissimum); 38418 B (sisymbriifolium); 38707 (barbisetum); 44089 B (aculeatissimum).

Clemens, J. 3311 (insanum); 3422 (torvum); 3453 (procumbens); 4004 (procumbens); 21518 (lasiocarpum).

Clemens, M.S. 1699 (schefferi); 17487 (torvoideum); 41609 (lasiocarpum); 51887 (retrorsum).

Clunie, N.M.U. LAE-63523 (lasiocarpum).

Cole, ? 35 (multiflorum).

Collard, D.J. 32 (insanum); 56 (insanum).

Collett, H. 312 (virginianum).

Collins, D.J. 66 (torvum); 904 (involucratum); 1380 (trilobatum).

Comanor, P.L. 781 (insanum); 785 (trilobatum); 922 (torvum); 1082 (torvum).

Condon, W. 1 (torvum).

Conklin, H.C. 217 (pseudosaponaceum); I-935 (melongena); 2563 (melongena); 17576 (lasiocarpum); 18710 (lasiocarpum); 18780 (melongena); 19187 (melongena); 19367 (melongena); 19401 (melongena).

Conn, B.J. 87 (lasiocarpum); 1672 (torvoideum); 5072 (torvum).

Coode, M.J.E. 5457 (lasiocarpum); NGF-29876 (lasiocarpum); NGF-32569 (dunalianum).

Cooper, R.E. 2435 (insanum); 3174 (violaceum).

Cooray, R.G. 68-102305 R (vagum).

Corner, E.J.H. SFN. 37775 (melongena); SF-37775 [a] (melongena); 38070 (violaceum).

Covile, D.P.M. 0237 (torvum); 2318 (capsicoides); 3669 (insanum); 4449 (capsicoides); 5444 (violaceum); 6199 (insanum).

Cowan, J.M. 114 (violaceum); 1018 (violaceum).

Cowper, S.G. 6 (torvum).

Cox, ? 532 (torvum).

Cramer, L.H. 3872 (violaceum); 4430 (torvum).

Craven, L.A. 89 (torvoideum).

Crawford, S. 1234 (torvum).

Croat, T.B. 52975 (torvum).

Crookshank, H. 549 (virginianum).

Cuadra, A. A 1010 (cyanocarphium); A 1169 (lasiocarpum); A 1171 (melongena); A-2160 (lasiocarpum).

Cubitt, G.E.S. 580 (melongena); 581 (lasiocarpum).

Cui Jinyun 14666 (praetermissum).

Cuming, H. 443 (insanum); 690 (lasiocarpum); 1529 (lasiocarpum); 2261 (violaceum); 2342 (trilobatum); 2422 (torvum).

Cuong, N.M. 48 (torvum); NMC 1439 (violaceum); NMC 1440 (procumbens).

Curran, H.M. 4874 (retrorsum); 4985 (torvoideum).

Curtis, C. 9 (torvum); 858 (trilobatum).

Dahlström, E. 467 (insanum).

Dakshum, K.M.M. NC-5554 (violaceum).

Daliph, N.E. 77 (cordatum).

Dalzell, N.A. 603 (insanum).

Dalziel, J.M. (insanum).

Damas, D. SAJ-1188 (torvum).

Daniel, P. NC-67592 (insanum).

Darnton, S. 72 (torvum).

Das, N.Y. NYD 190 (hovei); NYD 191 (hovei); NYD 709 (hovei); NYD 710 (hovei); NYD 1491 (hovei); NYD 2040 (hovei); NYD 5102 (hovei).

Das, R.K. 388 (praetermissum).

Datsum, K.M.M. NC-5509 (chrysotrichum).

Davidse, G. 7257 (insanum); 7374 (torvum).

Dawson, G. 541 (viarum).

Deb, D.B. 212 (insanum); 262 (viarum); 307 (violaceum); 1807 (melongena); CNH-3470 (insanum); BG-3471 (insanum); BG-3472 (insanum); BG-3474 (insanum); BG-3475 (insanum); BG-3476 (insanum); BG-3477 (insanum); BG-3478 (insanum); BG-3480 (insanum); BG-3481 (insanum); BG-3482 (insanum); BG-3483 (insanum); BG-3484 (insanum); BG-3485 (insanum); BG-3487 (insanum); BG-3489 (insanum); BG-3490 (insanum); 3491 (melongena); EC-26919 (melongena); 30146 (giganteum); SC-30163 (capsicoides); 30314 (multiflorum); 30725 (multiflorum).

Debbarman, P.M. 86 (insanum).

Debeaux, G. 258 (trilobatum).

Debeaux, J.O. 355 (violaceum).

Deguchi, K. 6317 35573 (torvum).

Delavay, J.M. 711 (violaceum); 1899 (violaceum).

Delavay, R.P. 898 (virginianum); 1025 (virginianum); 3147 (virginianum); 4400 (virginianum).

Dells, A. 3247 (elaeagnifolium).

Denker, C.J.F. 2 (involucratum).

Deshpande, S.D. 165729 (hovei); 165857 (hovei).

Dhothalri, K. BSI-9107 (torvum).

Dickason, F.G. 5932 (mammosum); 6719 (torvum).

Dickoré, B. 13338 (virginianum).

Diraviam, ? RHT-20553 (insanum).

Divakar, P. PD 2804 (hovei).

Diwakar, P.G. 184346 (hovei); 191880 (hovei).

Dobremez, J.F. 904 (violaceum); 2731 (virginianum).

Docters van Leeuwen, W.M. 10586 (lasiocarpum).

Dodar, M.N. 179172 (hovei).

Dorgelo, J.D. S 268 (involucratum).

Drennen, D.S.H. 24 (torvum).

Drummond, J.R. 13856 (cordatum); 14441 (insanum); 14448 (cordatum); 14703 (cordatum); 15259 (insanum); Kew-20549 (virginianum); 23619 (virginianum); 25858 (virginianum); 25859 (insanum); 25860 (virginianum); 25862 (insanum); 25863 (insanum); 25864 (insanum).

Du Puy, D.J. CI 94 (insanum).

Du, N.V. HNK-2470 (torvum); HNK-2799 (torvum).

Du, Y. SCSB-00007 (torvum).

Ducloux, F. 3910 (violaceum); 3911 (virginianum); 4772 (virginianum); 4773 (violaceum); 5209 (virginianum); 5888 (virginianum); 6235 (violaceum).

Dulong Jiang Investigation Team 6 (violaceum).

Dumont d’Urville, J.S.C. 211 (lasiocarpum).

Dunlop, C.R. 9619 (lasiocarpum).

Duport, ? 11 (procumbens); 63 (violaceum).

Dupuy, C. 92 (involucratum); 93 (violaceum).

Duthie, J.F. 10481 (insanum); 10482 (insanum).

Dutt, A.K. 664 (melongena).

Ebalo, L.E. 934 (lasiocarpum); 1344 (pseudosaponaceum).

Eberhardt, P.A. 1347 (procumbens); 2162 (procumbens); 3811 (insanum); 4265 (praetermissum).

Eckert, E.J. 69 (insanum).

Edaño, G.E. 247 (lasiocarpum); 11872 (torvum); 11990 (torvum); 36789 (torvoideum); 38321 (torvoideum); 48815 (torvoideum); BS-76376 (retrorsum); 77832 (lasiocarpum); 78556 (mammosum).

Eder, M.P. 28563 (torvoideum).

Edgeworth, M.P. 116 (insanum).

Edinburgh Nepal Expedition 21 (torvum).

Elbert, J. 2401 (lasiocarpum); 3224 (poka); 3575 (torvoideum).

Ellen, R.F. 836 (torvum).

Ellis, J.L. SC-11769 (trilobatum); SC-14242 (insanum); ANC-18793 (torvum); 20499 (multiflorum); SC-22154 (insanum); 29496 (multiflorum); SC-32673 (insanum).

Elmer, A.D.E. 2233 (lasiocarpum); 5528 (insanum); 5610 (insanum); 5819 (lasiocarpum); 5895 (torvoideum); 6073 (retrorsum); 6204 (pseudosaponaceum); 6732 (lasiocarpum); 8214 (insanum); 8328 (lasiocarpum); 8629 (torvoideum); 8719 (retrorsum); 10277 (lasiocarpum); 10752 (schefferi); 12879 (pseudosaponaceum); 13157 (cyanocarphium); 13879 (pseudosaponaceum); 17934 (lasiocarpum); 18123 (retrorsum); 20283 (torvum); 21869 (lasiocarpum).

Endert, F.H. 3477 (lasiocarpum); 3745 (lasiocarpum).

Erlanson, E.W. 5300 (torvum); 5602 (giganteum).

Escritor, L. 21415 (torvoideum); 21470 (pseudosaponaceum).

Esquirol, J.H. 931 p.p. (pseudosaponaceum).

Evrard, C.M. 2443 (lasiocarpum).

Evrard, F. 174 (violaceum); 176 (insanum); 426 (insanum); 819 (insanum); 1231 (camranhense); 1331 (violaceum); 1530 (nienkui).

Expeditio Biologica Sino-Rossica 1955 567/ 74 (violaceum).

Expedition Novara 134 (melongena).

Expedition Team 10159 (barbisetum); 32117 (lasiocarpum); 34605 (lasiocarpum).

Eyma, P.J. 2166 (poka).

F.F.B. 236 (multiflorum).

Faber, E. 190 (pseudosaponaceum); 612 (virginianum).

Falconer, H. 13 (violaceum); 35 (barbisetum); 38 (lasiocarpum); S 106 (violaceum).

Fallen, M. 328 (lasiocarpum).

Fan, C.S. 688 (capsicoides).

Fang, L.F. LHF-06 (torvum).

Faurie, U. 319 (capsicoides); 323 (violaceum); 646 (violaceum); 647 (lasiocarpum); 648 (pseudosaponaceum); 1158 (capsicoides); 1193 (violaceum); 1194 (violaceum); 1484 (capsicoides).

Feng, G. 11429 (deflexicarpum).

Fénix, E. 106 (violaceum); 3565 (insanum); 12934 (retrorsum); 15801 (pseudosaponaceum); 26103 (lasiocarpum).

Fernandes, J. 172 (torvum); 1055 (giganteum); 1098 (torvum); 1100 (giganteum); 1134 (torvum); 1265 (multiflorum); 1332 (torvum); 1377 (torvum); 1735 (torvum); 1748 (hovei); 1865 (hovei); 1924 (torvum); 2197 (lasiocarpum); 2333 (torvum); 2335 (giganteum).

Fernández, J. JF 1223 (multiflorum).

Fischer, C.E.C. 444 (trilobatum); 733 (wightii); 577 (torvum); 1552 (robustum); 2955 (multiflorum).

Fleury, F. 29910 (involucratum); 32439 (praetermissum).

Floto, F. 7617 (melongena); 7821 (trilobatum).

Floyd, A.G. 3506 (torvum).

Flügel, H.J. 1046 (hovei).

Forbes, F.B. 601 (wrightii); 613 (torvum).

Forbes, H.O. 10 (torvoideum); 438 (lasiocarpum); 949 b (torvum); 994 (torvum); 1253 (torvum); 1270 (lasiocarpum); 2085 (torvum); 2316 (lasiocarpum); 3634 (involucratum); 3786 (melongena); 3806 (poka); 3841 (violaceum); 4008 (involucratum); 4091 (insanum); 4096 (involucratum).

Ford, C.A. 123 (violaceum); 536 (torvum).

Foreman, D.B. LAE-60091 (torvoideum).

Forest District Officer 28 (insanum).

Forrest, G. 4483 (melongena); 6553 (viarum); 9103 (viarum); 9239 (torvum); 29576 (violaceum).

Fosberg, F.R. 36811 (hovei); 50081 (violaceum); 52845 (insanum); 53483 (violaceum); 56928 (insanum); 58158 (violaceum).

Foster, R. 14909 (torvum).

Foulkes, G.F.F. 83 (insanum).

Fox, R.B. 4793 (insanum); 13390 (lasiocarpum).

Frake, C.M. PNH 35973 (cyanocarphium).

Frake, C.O. 20323, 38012 (torvoideum).

Franck, C.W. 413 (violaceum).

Fraser, J. 120 (torvum).

Freitag, H. 51 (virginianum).

Frodin, D.G. 2413 (lasiocarpum).

Fujikawa, K. 35960 (torvum); 50117 (violaceum); 53092 (violaceum); 53095 (torvum); 53218 (viarum); 53219 (violaceum); 86161 (torvum); 86627 (torvum); 86711 (torvum); 86766 (torvum); 86914 (torvum); 90739 (torvum); 95433 (violaceum).

Fukuoka, N. T 7083 (trilobatum); T-36247 (trilobatum).

Furet, L. 200 (insanum).

Gachalian, F.S. 14937 (violaceum).

Gadd, G. 74 (virginianum).

Gaerlan 4887 (torvum); 5287 (schefferi).

Gage, A.T. 4 (lasiocarpum); 5 (torvum); 6 (violaceum); 145 (lasiocarpum).

Gale, U.M.G. 12310 (virginianum).

Gambating, A. SAN 107137 (cyanocarphium).

Gamble, J.S. 2460 A (torvum); 8966 (virginianum); 10490 (barbisetum); 10716 (trilobatum); 10987 (pubescens); 11265 (multiflorum); 11419 (giganteum); 11601 (wightii); 12189 (trilobatum); 12414 (wightii); 14294 (giganteum); 14490 (wightii); 14498 (wightii); 15071 (violaceum); 15173 (pubescens); 15787 (violaceum); 15829 (insanum); 15872 (trilobatum); 16045 (insanum); 16273 (violaceum); 16274 (torvum); 16275 (multiflorum); 16326 (melongena); 16874 (wightii); 16879 (wightii); 17077 (virginianum); 17376 (multiflorum); 17721 (insanum); 18677 (pubescens); 20879 (pubescens); 21410 (wightii); 21597 (insanum); 21672 (virginianum).

Gammie, G.A. 287 (insanum); 301 (sisymbriifolium); 638 (wightii); 15191 (hovei); 15593 (hovei).

Gandhi, K.N. HFP-2075 (violaceum).

Gaoligong Shan Expedition 7981 (viarum); 8570 (torvum); 8572 (violaceum); 9920 (torvum); 10444 (violaceum); 10529 (torvum); 10998 (aethiopicum).

Gaoligong Shan Biodiversity Survey 17207 (violaceum); 17239 (torvum); 17539 (torvum); 18145 (torvum); 18147 (violaceum); 18499 (viarum); 19935 (violaceum); 20957 (viarum); 23448 (torvum); 23513 (violaceum); 23859 (torvum); 24730 (aculeatissimum); 26298 (violaceum); 28945 (aculeatissimum); 28947 (violaceum); 29058 (viarum).

Gardner, G. 629 (torvum); 630 (violaceum); 631 (lasiocarpum).

Gardner, P.C. 1633 (viarum); 1638 (torvum).

Garrett, H.B.G. 1093 (lasiocarpum); 1253 (barbisetum).

Gaudichaud, C. 94 (insanum); 138 (lasiocarpum); 145 (insanum).

Geno, J.M. 77 (dunalianum).

Geoffray, C. 69 (lasiocarpum); 79 (insanum).

George, P. SAN 117639 (cyanocarphium).

Ghafoor, A. 4953 (virginianum).

Ghose, M.K. CNH-2637 (trilobatum).

Ghosh, M.K. CNH-133 (trilobatum); CNH-10944 (hovei); CSH-10968 (hovei).

Gibbs, L.S. 2639 (torvum).

Gideon, O. LAE 77094 (lasiocarpum).

Gilbert, M.G. 57 (procumbens); 341 (procumbens); 449 (capsicoides).

Gilli, A. 102 (torvoideum); 287 (torvoideum); 3245 (melongena); 3246 (melongena).

Gillison, A.N. NGF 22120 (lasiocarpum).

Ging, T.S. 6735 (violaceum).

Gjellerup, K. 651 (lasiocarpum).

Godefroy, A. 16 (insanum).

Godefroy-Lebeuf, M. 112 (lasiocarpum).

Goel, A.K. NC-65880 (violaceum).

Gopalan, V.J. 67255 (multiflorum).

Goring, V. 11 (torvum).

Gray, E.C. NGF-8094 (torvoideum).

Greijmans, M. 57-98 (lasiocarpum).

Gressitt, J.L. 907 (nienkui).

Grey-Wilson, C. 245 (virginianum).

Grierson, A.J.C. 1393 (torvum); 2164 (torvum); 2214 (torvum); 2675 (violaceum); 3378 (torvum).

Griffith, W. 614 (violaceum); 998 (lasiocarpum); 1000 (violaceum); 2352 (violaceum); 5906 (virginianum); 5906 [a] (virginianum); 5907 (trilobatum); 5910 (lasiocarpum); 5911 /1 (praetermissum); 5912 [a] (violaceum); 5912 [b] (violaceum); 5912 [c] (violaceum); 5912 [d] (melongena); 5913 (melongena).

Gueho, J. 15121 (torvum).

Gutiérrez, H.G. 78007 (lasiocarpum).

Haegens, R.M.A.P. 340 (lasiocarpum).

Hahn, L. 10 (lasiocarpum).

Haines, H.H. 146 (torvum); 462 (violaceum); 674 [a] (torvum); 674 [b] (violaceum); 2113 (virginianum); 3891 (trilobatum); 4180 (giganteum); 4918 (trilobatum).

Hajra, P.K. EC-37373 (aethiopicum); NC-82586 (insanum).

Hallier, H. 416 (torvoideum); 4185 (lasiocarpum); 4308 (torvoideum); 4476 (torvoideum).

Hallier, J.G. 220 (involucratum).

Hance, H.F. 860 (insanum); 860 [b] (insanum); 860 [a] (violaceum); 1285 (wrightii); 2483 (lasiocarpum); 5440 (pseudosaponaceum); 7565 (violaceum); 13816 (procumbens).

Hancock, A.D. 39 (torvum).

Haniff, M. 3852 (violaceum); 3873 (involucratum); 4351 (trilobatum).

Hansen, B. 10994 (torvum); 10995 (violaceum).

Hara, H. 1536 (viarum); 63-03526 (viarum); 63-03530 (violaceum); 63-03534 (torvum); 63-03540 (torvum); 24963 (violaceum).

Hardeveld, C. van 12 (violaceum); 153 (violaceum).

Hardial 239 (torvum); 497 (torvum).

Hartley, T.G. 10078 (dunalianum); 10405 (lasiocarpum); TGH-11579 (torvoideum).

Hasskarl, J. 1018 (involucratum).

Hayakawa’s collector S-86 (capsicoides).

Hayata, B. 150 (violaceum); 349 (nienkui); 800 (nienkui); 891 A (insanum).

Heinig, R.L. 44 (torvum); 45 (torvum).

Helfer, J.W. 24 (violaceum); 104 (torvum); 5908 (torvum); 5910 (lasiocarpum); 5911 (barbisetum).

Hemadri, K. 94357 (hovei); 99573 (hovei); 102360 (hovei); 107521 (hovei).

Hendrian, R. 964 (poka).

Henry, A. 259 (pseudosaponaceum); 358 (lasiocarpum); 746 (pseudosaponaceum); 795 (insanum); 795 A (insanum); 2792 (melongena); 8056 (procumbens); 8068 (insanum); 8140 (lasiocarpum); 8654 (lasiocarpum); 8856 (virginianum); 12158 (deflexicarpum); 12552 (torvum); 13592 (virginianum).

Henry, A.N. 2 (trilobatum); CNH-6 (insanum); 13 (elaeagnifolium); 20 (robustum); 65 (capsicoides); 17392 (multiflorum); 19811 (multiflorum); 19900 (vagum); 45403 (trilobatum); 49661 (multiflorum); SC-53308 (insanum).

Henty, E.E. NGF-11553 (torvoideum); NGF-11646 (torvoideum); NGF-49450 (lasiocarpum).

Hepper, F.N. 4402 (melongena); 4411 (torvum); 4415 (torvum); 4482 (torvum); 4497 (insanum); 4500 (torvum); 4506 (torvum); 4518 (violaceum); 4519 (torvum); 4548 (melongena); 4575 (violaceum); 4576 (torvum); 4581 (giganteum); 4582 (violaceum); 4585 (giganteum); 4599 (violaceum); 4617 (violaceum); 4633 (violaceum); 4641 (insanum); 4642 a (insanum); 4642 b (melongena); 4643 (insanum); 4646 (insanum); 4649 (torvum); 4665 (violaceum); 4675 (torvum); 4704 (insanum); 4713 (torvum); 4718 (violaceum); 4719 (insanum); 4769 (violaceum); 4770 (melongena).

Herb. East India Company 5910 (lasiocarpum).

Herb. Griffith 5912 (multiflorum).

Herb. Pallas 80 (violaceum).

Herbarium Boschproefstation 5413 (involucratum).

Herbert, P.S. S.H. 1047 (hovei).

Hermengild, S. 4965 (hovei); 5609 (hovei); 6947 (hovei); 9693 (hovei); 9694 (hovei); 12702 (hovei); 15831 (hovei); 16096 (hovei); 17518 (hovei); 17519 (hovei); 17573 (hovei); 18879 (hovei); 19384 (hovei); 20946 (hovei); 20947 (hovei); 21111 (hovei); 21112 (hovei); 21113 (hovei); 21192 (hovei); 22454 (hovei); 22459 (hovei); 22461 (hovei); 22463 (hovei); 22465 (hovei); 22754 (hovei); 23153 (hovei); 28419 (hovei); 28850 (hovei).

Herre, A.W. 333 (dunalianum).

Hewer, T.F. 1344 (virginianum).

Heyne, B. Wall. cat. 2624 a (vagum); Wall.cat. 2629 a (pubescens); Wall.cat. 2629 b (pubescens).

Ho, P.H. 5296 (robinsonii).

Hoan, D.T. HN-NY 1022 (torvum).

Hoare, A. 13 (lasiocarpum).

Hobby, B.M. 2574 (lasiocarpum).

Hoed, G. den 962 (torvum); 977 (barbisetum).

Hoffmannsegg, J.C. von 119 (insanum); 137 (pseudosaponaceum).

Hofmann, U. 1763 (torvum).

Hohenacker, R.F. 36 (torvum); 36 A (torvum); 36 B (insanum); 672 a (torvum); 740 (trilobatum); 780 (virginianum); 1074 (multiflorum); 1075 (giganteum); 1076 (wightii); 1416 (violaceum); 1417 (wightii).

Holman, R.M. 184 (melongena).

Holttum, R.E. 24715 (lasiocarpum).

Hoogland, R.D. 3334 (schefferi); 4349 (schefferi).

Hooker, J.D. 14 (aculeatissimum); 24 (melongena).

Hooper, D. REP-25810 (torvum); 39555 (trilobatum); 39606 (melongena).

Hoover, W.S. 30027 (torvum); 30778 (lasiocarpum); 30804 (torvum).

Hore, D.K. ANC-8823 (torvum).

Horreüs de Haas, R.H. 512 (virginianum).

Horsfield, T. 14 (cyanocarphium); 15 (graciliflorum); 16 (violaceum); 18 (violaceum); 19 (violaceum); 20 (involucratum); 114 (insanum); 429 (violaceum); 786 (poka).

Hosagoudar, V.B. 96871 (multiflorum).

Hosseus, C.C. 475 (torvum).

How, F.C. 70256 (pseudosaponaceum); 71437 (insanum).

Hsu, C.C. 4880 (miyakojimense); 13689 (peikuoense).

Htwe, K.M. 23984 (torvum); 24059 (violaceum); 32526 (torvum); 32622 (virginianum); 32757 (torvum); 32777 (violaceum).

Hu, S.Y. 5710 (lasiocarpum); 5711 (torvum); 5728 (torvum); 9436 (melongena); 12358 (torvum); 12785 (torvum); 12901 (torvum); 23553 (violaceum).

Huang, T.C. 15850 (peikuoense).

Hügel, A. 1655 (hovei); 2119 (hovei); 2290 (hovei); 2979 (hovei).

Huk, A. 98 (virginianum).

Huklajn, A.K. CNH-2229 (arundo).

Hul, S. 3184 (robinsonii); 3211 (camranhense); 3552 (violaceum); 4774 (trilobatum).

Huq, A.M. 10456 (torvum); 10569 (virginianum); 10618 (torvum).

Hutchinson, W.I. 3969 (torvoideum).

Illegible 279 (insanum); 22438 a (insanum).

Ináyat 22438 (insanum); NC-22439 (violaceum).

Incarville, P.N. d’ 133 (insanum).

Irani, N.A. NI 2559 (hovei); NI 2619 (hovei); NI 4588 (hovei); NI 5073 (hovei).

Isles, S. NGF-34491 (lasiocarpum).

Islewas, M.C. CNH-19460 (trilobatum).

Ismail, S. 8 (violaceum); 60 (barbisetum).

Iwatsuki, K. 250 (trilobatum); T-8512 (torvum); T-10866 (lasiocarpum).

Jaag, O. 452 (violaceum); 1209 (lasiocarpum).

Jackson, J.K. 6111 (violaceum); 6151 (barbisetum).

Jackson, S. 4 (virginianum).

Jacob, K.C. 16190 (multiflorum); 17314 (pubescens).

Jacobs, M. 7056 (pseudosaponaceum); 7797 (pseudosaponaceum).

Jacquemont, V.V. 193 (violaceum); 197 (virginianum); 258 (violaceum); 304 (insanum); 408 (violaceum); 563 (hovei); 1110 (insanum); 1489 (insanum); 2315 (insanum); 2318 (virginianum); 9109 (insanum).

Jafri, S.M.H. 806 (virginianum); 903 (cordatum); 3737 (elaeagnifolium).

Jain, S.K. 939 (hovei); 2277 (hovei); 2405 (hovei); 8207 (hovei).

James, S.A. SAJ 72 (lasiocarpum).

Jamieson 333 (insanum); 488 (virginianum).

Janaki Ammal, E.K. 1445 (torvum).

Janardhanan, K.P. 69106 (hovei); 69289 (hovei); 70102 (hovei); 72239 (hovei); 72408 (hovei); 75885 (hovei); 76061 (hovei); 76126 (hovei); 76278 (hovei); 76645 (hovei).

Jaru, S.K. 29183 (hovei).

Jayasuriya, A.H.M. 2039 (trilobatum); 6987 (vagum); 17104 (melongena); 17179 (insanum).

Jayaweera, D.M.A. 923 (lasiocarpum); 1045 (violaceum).

Jenkins, F. 249 (barbisetum); 250 (praetermissum); 252 (violaceum); 253 (praetermissum).

Jensen, H. 332 (dunalianum).

Jeshoda, K. 413 (pubescens); 414 (torvum).

Jeswiet, J. 1155 (involucratum).

Johansson, J.T. 144 (involucratum); 419 (poka).

Johns, R.J. 8232 (lasiocarpum).

Jones, B.L. 152 (melongena).

Joseph SAN-117010 (lasiocarpum).

Joseph, J. 10598 (wightii); 13574 (pubescens); SC-14701 (insanum); SC-17065 (insanum).

Juan, G.E. 343 (melongena).

K’tung 78 5951 (violaceum).

Kadamban, D. 259 (trilobatum); 269 (torvum).

Kadhum, H. 37772 (elaeagnifolium).

Kadim, T. KN. 270 (lasiocarpum).

Kadir bin Abdul A-2834 (lasiocarpum).

Kairo, A. 43 (dunalianum); 283 (lasiocarpum); 443 (torvoideum); 10650 (lasiocarpum); NGF-30692 (lasiocarpum).

Kalkman, C. BW-3720 (dunalianum).

Kalu, A. 4172 (hovei).

Kamble, M.Y. ANC-29117 (melongena).

Kameswara Rao, N. 74 (torvum).

Kanai, H. 63 03535 (torvum); 72601 (viarum); 72709 (torvum); 6303525 (virginianum); 6303533 (violaceum).

Kanazawa University Japan East Nepal 95-52915 (torvum).

Kanodia, K.C. 88299 (hovei); 89338 (hovei); 89795 (hovei).

Kao, M.T. 10722 (miyakojimense).

Kapadia, Z.F. 302 (hovei); ZK 1238 (hovei); ZK 2030 c (multiflorum); ZK 2030 b (multiflorum); ZK 2030 d (multiflorum); ZK 2030 e (multiflorum); ZK 2030 a (multiflorum); ZK 2100 (hovei); ZK 2474 (hovei).

Kartonegro, A. 300 (jamaicense).

Kasin 160 (barbisetum).

Katik, P. LAE 70804 (lasiocarpum); LAE-70809 (dunalianum).

Kato, M. C-5431 (poka); C-7942 (poka).

Kau, M.G. 245 (wrightii).

Kaudern, W.A. 57 (dunalianum).

Kaulback, R. 180 (torvum).

Kaur, U. UK-2 (chrysotrichum).

Kayal, R.N. Ph-597 (elaeagnifolium).

Keenan, J. 946 (lasiocarpum); 1090 (torvum); 1276 (barbisetum); 1281 (torvum); 1524 (barbisetum); 1662 (barbisetum); 1723 (barbisetum); 1741 (torvum); 3828 (viarum).

Keith, H.G. 9282 (lasiocarpum).

Kerenga, K. LAE 56923 (dunalianum); LAE-73982 (lasiocarpum).

Kern, J.H. 8491 (involucratum).

Kerr, A.F.G. 1236 (lasiocarpum); 1237 (violaceum); 1801 (torvum); 1862 (barbisetum); 2168 (trilobatum); 2201 (insanum); 2320 (mammosum); 2370 (virginianum); 3675 (trilobatum); 3956 (torvum); 4019 (insanum); 4020 (melongena); 4064 (violaceum); 4220 (lasiocarpum); 4707 (capsicoides); 5115 (mammosum); 9048 (violaceum); 10780 (involucratum); 11039 (violaceum); 14751 (violaceum); 15151 (wrightii); 15820 (torvum); 16141 (insanum); 16210 (procumbens); 18226 (violaceum); 18641 (capsicoides); 19844 (involucratum); 20903 (praetermissum).

Kerr, F.H.W. 2051 (melongena); 2520 (torvum).

Kessler, P.J.A. 1559 (lasiocarpum); 2952 (lasiocarpum).

Khalil, A. 189 (insanum).

Khan, B. 539 (lasiocarpum).

Khanna, V. 513 (virginianum).

Khirti, G.H. 124419 (hovei); 125235 (hovei).

King’s collector 18 (violaceum); 82 (lasiocarpum); 105 (torvum); 111 (torvum); 363 (violaceum); 398 (violaceum); 454 (violaceum); 539 (lasiocarpum); 1018 (virginianum); 1022 (lasiocarpum).

Kingdon-Ward, F. 18635 (sisymbriifolium); 18879 (violaceum); 21211 (kachinense).

Kjellberg, G. 399 (lasiocarpum); 745 (lasiocarpum); 1707 (lasiocarpum); 2525 (lasiocarpum).

Klackenberg, J. 1047 (praetermissum).

Klappa, S. 83 (lasiocarpum); 85 (torvum).

Kloss, C.B. 6990 (torvum).

Knapp, S. IM-10106 a (violaceum); IM-10107 (aculeatissimum); IM-10109 (torvum); 10110 (violaceum); 10113 (melongena); IM-10114 (melongena); IM-10115 (melongena); IM-10116 (melongena); IM-10117 (melongena); IM-10118 (melongena); IM-10119 (melongena); IM-10121 (aethiopicum); IM-10122 (melongena); 10123 (torvum); 10124 (melongena); IM-10125 (barbisetum); IM-10126 (barbisetum); 10127 (barbisetum); 10128 (viarum); 10129 (barbisetum); 10130 (deflexicarpum); 10131 (melongena); IM-10132 (melongena); BHSC-37709 (torvum); BHSC-37711 (aethiopicum); BHSC-37713 (torvum).

Kobayashi, K. 31426 (virginianum).

Kochhar, R.K. 152547 (hovei); 153565 (hovei); 153573 (hovei); 157988 (hovei).

Koelz, W. 1633 (virginianum); 4210 (insanum); 10727 (violaceum); 10842 (insanum); 11040 (multiflorum); 19751 (multiflorum); 19899 (insanum); 19970 (violaceum).

Kondo, Y. 38863 (cyanocarphium).

König, J.G. H iv 235 (torvum).

Koorders, S.H. 18035 (poka); 18037 (poka); 22525 B (involucratum); 22827 B (involucratum); 23333 (involucratum); 26280 (lasiocarpum); 28410 (involucratum); 28822 (involucratum); 31988 (lasiocarpum); 43213 (involucratum).

Kooy, C.W. 532 (lasiocarpum).

Koster, C. BW-13829 (torvoideum); BW-13976 (lasiocarpum).

Kostermans, A.J.G.H. 32 (lasiocarpum); 435 (melongena); 609 (lasiocarpum); 962 (torvum); 1369 (torvum); 1433 (barbisetum); 1447 (lasiocarpum); 4814 (cyanocarphium); 7886 (lasiocarpum); 19055 (torvoideum); 21707 (lasiocarpum); 23178 (torvum); 26207 (giganteum).

Koyama, H. 49061 (trilobatum).

Koyama, T. 13357 (vagum); 15698 (insanum); 15973 (torvum); 16012 (violaceum); 16034 (chrysotrichum); 16038 (torvum); 16043 (chrysotrichum); 16059 (insanum); 16060 (torvum).

Kray, C.W. 156 (lasiocarpum).

Krempf, M. 1415 (insanum).

Krishna, B. SKS 1627 (torvum).

Krishnappa, D.G. 20 (insanum); 63 (trilobatum); 68 (insanum); 70 (hovei); 80 (pubescens); 109 (violaceum); 121 (violaceum); 158 (multiflorum); 162 (elaeagnifolium); 165 (virginianum); 371 (hovei); 397 (giganteum).

Ku, S.-.M. 1752 (peikuoense).

Kulkarni, B.G. 105670 (hovei); 106383 (hovei); 106481 (hovei); 108582 (hovei); 118911 (hovei); 119422 (hovei); 119542 (hovei); 120490 (hovei); 121005 (hovei); 121319 (hovei); 121456 (hovei); 128798 (hovei); 128883 (hovei); 131746 (hovei).

Kumar, A. CNH-15565 (insanum).

Kumar, S. ANC-26305 (torvum); ANC-26356 (torvum); ANC-26924 (virginianum); ANC-26953 (virginianum); ANC-26967 (violaceum); ANC-27623 (melongena); NC-92333 (insanum); NC-93273 (chrysotrichum).

Kumarilingam, ? 5218 (hovei).

Kundu, B.C. 564 (trilobatum).

Kuntze, C.E.O. 3578 (insanum); 3766 (procumbens); 7481 (trilobatum); 7620 (hovei).

Kuroiwa, N. 28755 (torvum); 28797 (virginianum).

Kurz, S. 202 (lasiocarpum); 204 (lasiocarpum); 2267 (lasiocarpum); 2268 (barbisetum); 2648 (virginianum).

Lacaita, C.C. H IV 234 (violaceum).

Lace, J.H. 1710 (virginianum); 2808 (violaceum); 2809 (torvum); 3242 (torvum); 3278 (violaceum); 3672 (cordatum); 3969 (virginianum); 3972 (cordatum).

Ladell, W.R.S. 213 (torvum); 260 (torvum).

Lahaie, F. de 1765 (violaceum); 2384 (pseudosaponaceum); 2400 (trilobatum).

Lai, C.L. 392 (insanum).

Lai, P. NC-78916 (violaceum).

Lakra, G.S. ANC-12677 (viarum); ANC-15574 (torvum); ANC-28053 (torvum).

Lal, P. NC-63254 (insanum).

Lam, H.J. 636 (lasiocarpum); 2772 (poka).

Lambach, P.M. 1268 (lasiocarpum).

Lamei, P. LAE-89467 (torvum).

Lamond, J.

Lamond, J.M. 1353 (cordatum); 1399 (elaeagnifolium).

Lamont, J. 494 (violaceum); 495 (insanum); 496 (lasiocarpum).

Larsen, K. 1614 (trilobatum); 10520 (barbisetum); 33899 (barbisetum); 45465 (procumbens).

Lau, S.K. 17 (procumbens); 114 (insanum); 157 (pseudosaponaceum); 163 (lasiocarpum); 685 (violaceum); 1029 (insanum); 1154 (nienkui); 1397 (nienkui); 2828 (insanum); 2970 (nienkui); 3446 (procumbens); 3468 (nienkui); 3646 (insanum); 3659 (lasiocarpum); 4392 (capsicoides); 4770 (capsicoides); 5024 (nienkui); 5095 (insanum); 5138 (procumbens); 5339 (pseudosaponaceum); 6011 (insanum); 6214 (pseudosaponaceum); 6285 (nienkui); 25547 (pseudosaponaceum); 25849 (lasiocarpum); 27093 (nienkui); 27479 (nienkui); 27683 (nienkui); 28189 (melongena); 28327 (melongena).

Laukkonen, P. 333 (elaeagnifolium).

Le Cong Kiet 94 (camranhense).

Le Guillou, E.J.F. 37 (insanum).

Lecomte, H. 78 (violaceum); 1426 (insanum); 1640 (lasiocarpum); 2092 (insanum).

Ledermann, C.L. 6898 (lasiocarpum).

Lee, Y.K. 01142 (praetermissum).

Lefèvre, E. 13 (insanum).

Lehmann, F.C. 4729 (mammosum).

Lei, C.I. 277 (procumbens); 392 (insanum); 413 (violaceum); 1259 (nienkui); 1299 (nienkui); 1390 (nienkui).

Lelean, Y. LAE 52534 (lasiocarpum).

Leschenault de la Tour, J.B.L. 163 (multiflorum); 189 (pubescens); 822 (insanum); 855 (virginianum).

Leu, W.-.P. 1026 (violaceum); 2118 (retrorsum); 2164 (miyakojimense).

Levine, C.O. 375 (torvum); 609 (torvum); 3257 (lasiocarpum).

Li Yanhui 1079 (lasiocarpum); 1699 (lasiocarpum); 2578 (praetermissum); 2668 (praetermissum); 3860 (barbisetum); 4232 (barbisetum); 4835 (lasiocarpum); 12316 (praetermissum); 12594 (barbisetum).

Li, H. 13382 (aculeatissimum); 13383 (violaceum); 14067 (aethiopicum).

Liang, H.Y. 61510 (procumbens); 61698 (pseudosaponaceum); 62209 (nienkui); 62400 (pseudosaponaceum); 62477 (procumbens); 63040 (nienkui); 63797 (pseudosaponaceum); 64482 (procumbens); 64626 (procumbens); 66186 (insanum); 66539 (nienkui); 66558 (procumbens).

Lichy, R. 18 (cyanocarphium); 45 (insanum).

Lin, C.-.H. 405 (peikuoense).

Lin, H.-.W. 938 (peikuoense).

Lin, Y.-.R. 134 (peikuoense).

Lister, J.L. 194 (lasiocarpum); 203 (torvum).

Liu, T.-.Y. 427 (peikuoense); 1369 (peikuoense).

Liu, Y.C. 540 (pseudosaponaceum).

Llanos 259 (lasiocarpum).

Loher, A. (torvoideum); 4366 (torvoideum); 4367 (torvoideum); 4368 (insanum); 4369 (lasiocarpum); 4370 (lasiocarpum); 4371 (lasiocarpum); 4372 (torvoideum); 4373 (trilobatum); 4378 (retrorsum); 4379 (retrorsum); 6644 (retrorsum).

Londhe, A.N. 170566 (hovei).

Lorence, D.H. 508 (melongena).

Lörzing, J.A. 4425 (lasiocarpum); 5494 (lasiocarpum); 11630 (violaceum); 11878 (lasiocarpum); 13254 (melongena); 15014 (lasiocarpum).

Lu, F.Y. 679 (insanum).

Lu, P.-.F. 12541 (peikuoense); 16536 (peikuoense).

Ludlow, F. 7244 (torvum).

Lugas, L. 367 (lasiocarpum); 546 (lasiocarpum); 682 (lasiocarpum); 1185 (lasiocarpum); 2646 (lasiocarpum).

Luke, A. 87 (viarum).

Ly, V. 953 (involucratum).

MacGregor, R.W. 580 (melongena); 581 (violaceum); 585 (violaceum); 587 (violaceum); 1063 (violaceum).

Maclagan, G.J. 113 (insanum).

Macrae, J. 107 (trilobatum); 634 (insanum).

Madani, L. SAN 90973 (lasiocarpum); SAN 91102 (lasiocarpum).

Madras Herbarium 9604 (multiflorum); 10350 (trilobatum); 12247 (vagum); 13207 (vagum); 13338 (pubescens); 13609 (multiflorum); 14331 (pubescens); 16772 (vagum).

Madulid, D.A. 3115 (pseudosaponaceum); 8146 (torvum).

Maesen, L.J.G. van der 672 (pubescens); 737 (violaceum); 922 (chrysotrichum); 1981 (violaceum); 2693 [a] (insanum); 2693 [b] (melongena); 2702 (torvum); 2705 (violaceum); 3131 (virginianum); 3235 (pubescens); 3276 (violaceum); 3289 (insanum); 4035 (violaceum).

Mahajan, S.D. 6933 (hovei); 13222 (hovei); 24723 (hovei); 27196 (hovei).

Mahyar, U.W. 950 (lasiocarpum).

Maikin, ? SAN 101847 (lasiocarpum).

Maingay, A.C. 398 (melongena); 1158 (cyanocarphium); 1776 (torvum).

Maire, E.E. 7416 (violaceum).

Majumdar, S.C. 192892 (hovei).

Maknoi, C. 889 (barbisetum).

Malhotra, B.K. NC-18518 (chrysotrichum); NC-27684 (violaceum).

Malhotra, C.L. NC-12454 (chrysotrichum); NC-12457 (chrysotrichum); NC-36843 (chrysotrichum); NC-37197 (chrysotrichum); NC-42787 (violaceum); 50949 (insanum); 50959 (insanum); NC-77727 (violaceum).

Malhotra, S.K. NC-15410 (chrysotrichum); NC-17227 (chrysotrichum); NC-19132 (chrysotrichum); NC-22736 (chrysotrichum); NC-22764 (violaceum); NC-23458 (chrysotrichum); NC-23726 (violaceum); NC-23856 (violaceum); NC-23986 (chrysotrichum); NC-25671 (violaceum); NC-27101 (insanum); NC-31207 (chrysotrichum).

Malik, K.A. 970 (virginianum).

Maliwanang, E. 229 (torvoideum).

Man, H. 85157 (torvum).

Man, L.S. 52114 (torvum); 55002 (torvum); 55208 (torvum); 87170 (torvum); 87438 (viarum); 87540 (violaceum); 87983 (viarum); 87986 (torvum); 88072 (torvum); 92848 (torvum); 92883 (violaceum); 96161 (viarum); 96248 (violaceum).

Mandal, N.R. BSHC-13356 (violaceum).

Mandel, N.R. 9345 (trilobatum).

Mann, G. 1110 (capsicoides).

Marcan, A. 31 (torvum); 67 (melongena); 68 (lasiocarpum); 73 (trilobatum); 192 (involucratum); 284 (violaceum); 702 (wrightii); 2125 (melongena).

Marche, A. 187 (insanum).

Maries, C. 34 (virginianum); 277 (torvum).

Marriot, M.V. 86 (torvum).

Martin, M. 1383 (pseudosaponaceum).

Maser, R. 6 (torvum).

Matthew, K.M. 8323 (trilobatum); RHT-12652 (trilobatum); 16383 (violaceum); 16401 (vagum); RHT 19640 (pubescens); RHT-20367 (violaceum); RHT 24704 (trilobatum); 28443 (pubescens); 40094 (multiflorum); 40301 (multiflorum); RHT-41189 (torvum); RHT-41805 (violaceum); RHT 43220 (trilobatum); RHT 51614 (pubescens); 51696 (giganteum); RHT-53878 (virginianum).

Maung, L.S. 24373 (torvum).

Maxwell, J.F. 95-353 (lasiocarpum); 02-360 (torvum); 06-373 (barbisetum); 05-378 (barbisetum); 91-385 (violaceum); 92-446 (barbisetum); 98-564 (barbisetum); 91-631 (barbisetum); 74-702 (involucratum); 89-729 (barbisetum); 95-773 (barbisetum); 89-784 (torvum); 90-813 (barbisetum); 97-886 (barbisetum); 89-946 (barbisetum).

Maxwell, R.H. 988 (macrocarpon); 995 (insanum).

McCann, C. 2002 (hovei); 2006 (hovei); 22451 (hovei); 22453 (hovei); CS 50504 (multiflorum).

McClatchey, W. WCM 3145 (violaceum); WCM 3151 (lasiocarpum).

McClure, F.A. 7641 (insanum); 8383 (lasiocarpum); 8833 (procumbens); 9518 (pseudosaponaceum).

McDonald, ? 4122 (lasiocarpum); 4479 (insanum).

McGregor, R.C. 8525 (pseudosaponaceum); 19741 (pseudosaponaceum); 20128 (retrorsum).

Meebold, A. 7661 (mammosum); 7664 (lasiocarpum); 7743 (violaceum); 10029 (trilobatum); 15084 (mammosum); 16683 (barbisetum).

Meeboonya, R. RM 242 (trilobatum); RM 243 (trilobatum); RM 244 (trilobatum); RM 245 (trilobatum); RM 248 (melongena); RM 249 (torvum); RM 250 (melongena); RM 251 (melongena); RM 257 (melongena); RM 266 (melongena); RM 267 (torvum); RM 271 (wrightii); RM 272 (lasiocarpum); RM 274 (torvum); RM 277 (melongena); RM 287 (lasiocarpum); RM 294 (melongena); RM 295 (melongena); RM 296 (torvum); RM 301 (trilobatum).

Mendoza, D. 10718 (torvoideum).

Mendum, M. 99-264 (retrorsum).

Merchant, Y.A. 635 (hovei); 1179 (hovei).

Merrill, E.D. 345 (insanum); 465 (lasiocarpum); 623 (melongena); 856 (torvoideum); 938 (insanum); 1736 (torvoideum); 1736 (torvoideum); 3406 (torvum); 4237 (retrorsum); 4807 (pseudosaponaceum); 7754 (retrorsum); 9665 (retrorsum).

Merritt, M.L. 12038 (lasiocarpum).

Metcalf, F.P. 7423 (capsicoides); 7647 (chrysotrichum).

Meyer, A.B. 43 (pseudosaponaceum).

Meyer, R. 2233 (lasiocarpum); 2285 (torvoideum).

Mikage, M. 95-52940 (violaceum).

Millar, A.N. NGF-15605 (lasiocarpum).

Milliken, W. 1058 (lasiocarpum).

Millspaugh, C.F. 3506 (torvum).

Minaki, M. 90-80025 (violaceum).

Misra, O.P. NC-38288 (violaceum); CNH-41463 (insanum); 41608 (insanum); NC-44313 (insanum); 44661 (insanum); NC-47295 (insanum).

Mitchell, B.A. 17 (involucratum).

Mobbs, R.H. p 182 (violaceum).

Mohan, A. 13431 (viarum).

Mohanan, C.N. 55516 (multiflorum); SC-72093 (capsicoides); SC-77917 (capsicoides).

Moi, W. 188 (torvoideum).

Mokim, S. 1 (trilobatum); 623 (capsicoides); 1239 (virginianum); 1594 (melongena).

Monro, A.K. 6413 (viarum); 6454 (torvum); 6497 (torvum).

Moon, A. 465 (giganteum).

Mooney, H.F. 120 (torvum); 463 (violaceum); 688 (torvum); 976 (virginianum); 2482 (violaceum); 2715 (giganteum); 3066 (trilobatum); 3231 (violaceum); 3331 (violaceum); 3332 (torvum); 3934 (torvum).

Morrison, A. 13 (sisymbriifolium).

Morrison, K. 5 (insanum).

Morse, H.B. 281 [a] (violaceum); 281 [b] (insanum); 282 (insanum).

Motley, J. 148 (cyanocarphium); 187 (involucratum).

Mouret, M. 203 (violaceum); 205 (procumbens); 206 (procumbens).

Mousset, J.P. 93 (involucratum).

Mueller-Dombois, D. 68-10210 R (insanum).

Mukerjee, S.K. 1918 (insanum); 3247 (aethiopicum).

Mukherjee, A. 150 (insanum).

Mukherjee, A.K. E-4301 (trilobatum); E-4307 (trilobatum); 5358 (trilobatum); 5934 (trilobatum).

Mukherjee, B.K. 6114 (violaceum); 6144 (viarum).

Mukhopadhyay, C.R. 428 A (insanum).

Murata, G. B-19 (lasiocarpum); 3722 (torvum); T-15456 (insanum); T-16792 (violaceum); T-16862 (barbisetum); T-17303 (trilobatum); T-17786 (torvum); T-17787 (involucratum).

Murty, U.R. 1559 (chrysotrichum); 5631 (insanum).

Murugan, C. 117673 (multiflorum).

Naattacharyya, U.C. CNH-39177 (insanum).

Nadil Khan 123 (aculeatissimum).

Naeh, M. 19403 A (virginianum).

Nafday, K.U.R. 46 (virginianum); 158 (melongena).

Nagamasu, H. 6917 (torvum); 35557 (torvum).

Naganathan, T.R. 19407 (trilobatum).

Nair, N.C. ANC-810 (viarum); ANC-811 (violaceum); ANC-1599 (insanum); ANC-6248 (torvum); 50743 (multiflorum); 52937 (trilobatum); 52939 (pubescens); 60827 (pubescens); SC-60904 (insanum); 64365 (multiflorum); 70195 (multiflorum).

Nair, V.J. NC-23156 (insanum); 56538 (trilobatum); 67255 (multiflorum); 67370 (multiflorum).

Naithani, B.D. 23184 (trilobatum); 24666 (multiflorum).

Nanakorn, W. 88-0192 (trilobatum).

Narasimhan, D. 624 (trilobatum).

Narasimhan, P.L. 163984 (hovei); 165976 (hovei).

Narayanaswami, V. 131 (insanum); 131 (insanum); 168 (melongena); 451 (melongena); 849 (multiflorum); 3507 (multiflorum); 3586 (insanum); 4131 (trilobatum); 4739 (multiflorum); 19021 (pubescens); 19996 (pubescens); 80789 (trilobatum).

Nasir, E. 16303 (cordatum).

Native Collector CD-209 (lasiocarpum); 1056 (violaceum).

Nautujal, S. 41 (violaceum).

Nawi, R. SAN-55905 (torvum).

Nayar, M.P. 153128 (hovei).

Nedi 5 (jamaicense); 494 (dunalianum); 514 (lasiocarpum); 639 (melongena).

Nepal Bajhang Expedition 209-13060 (virginianum); 209-13087 (viarum).

Neubauer, J.H.F. 1775 (virginianum).

Newman, M.F. LAO-43 (torvum); 2222 (torvum).

Nguyen, V.D. HNK 1332 (torvum).

Nicholson, C.J. Godwin 49 (torvum).

Nicolson, D.H. 1868 (torvum).

Noor, M. MN. 57 (insanum).

Nooteboom, H.P. 5837 (lasiocarpum).

Norul Rozimah Pg Hj Seruji 18b 3000 (torvum).

Noshiro, S. 9760025 (virginianum); 9760490 (virginianum).

Nuraliev, M.S. NUR-101 o (viarum); NUR-1863 a (putii).

Nuskar, J.D. 11 (trilobatum).

Oates, J.F. 159 (giganteum).

Ober 300 (insanum).

Ohashi, H. 77 4330 (violaceum).

Oldham, R. 338 (violaceum); 339 (insanum); 339 b (insanum); 342 (pseudosaponaceum).

Olsen, S. 424 (lasiocarpum); 773 (cyanocarphium); 1057 (cyanocarphium).

Oswald B, A. 146 (violaceum).

Oswald, P.H.O. 15 (elaeagnifolium).

Otanes, F. 2071 (lasiocarpum); 17710 (retrorsum); 17776 (insanum).

Palee, P. 988 (barbisetum).

Pallithanam, J. G 459 (hovei); 58-3436 (pubescens).

Pandey, R.P. AC-7972 (insanum).

Panigrahi, G. CC-3057 (insanum); 3754 (melongena); CC-4283 (insanum); 5495 (viarum); CC-5881 (insanum); CC-6353 (insanum); 11240 (melongena); 12048 (melongena); EC-21602 (melongena); EC-23645 (insanum); EC-23792 (trilobatum); EC-23832 (trilobatum).

Panst, P.C. 43446 (insanum).

Pant, P.C. NC-37543 (chrysotrichum); NC-38551 (violaceum); NC-43404 (insanum); NC-43446 (insanum); NC-43795 (violaceum).

Parker, C. 7254 (violaceum).

Parker, R.N. 3195 (viarum); 3326 (cordatum).

Parkinson, C.E. 15699 (violaceum).

Parkinson, C.L. 13980 (torvum); 14657 (torvum).

Parkinson, R. 10 (dunalianum).

Parmar, P.J. AC-2458 (virginianum).

Parthasarathy, N. 708 (vagum).

Pataskan, R.D. 102138 (hovei); WC-105834 (insanum); WC-105889 (insanum); 108875 (hovei); WC-110313 (insanum).

Patel, V.M. VP 721 (hovei); V.P. 730 (hovei); V.P. 733 (hovei); V.P. 739 (hovei); VP 769 (hovei); VP 770 (hovei); VP 771 (hovei); VP 776 (hovei); V.P. 782 (hovei); VP 796 (hovei); V.P. 797 (hovei); V.P. 798 (hovei); V.P. 799 (hovei); VP 800 (hovei); V.P. 804 (hovei); V.P. 814 (hovei); V.P. 815 (hovei); V.P. 816 (hovei); VP 817 (hovei); V.P. 818 (hovei); VP 935 (hovei); VP 936 (hovei); V.P. 1080 (hovei); V.P. 1119 (hovei); V.P. 1139 (hovei); V.P. 1140 (hovei); V.P. 1437 (hovei); V.P. 1438 (hovei); VP 1894 (multiflorum); VP 1895 (multiflorum); VP 1896 (multiflorum); VP 1897 (multiflorum); VP 1898 (multiflorum); VP 1899 (multiflorum); VP 1900 (multiflorum); VP 1901 (multiflorum); VP 1902 (multiflorum); V.P. 1951 (hovei); V.P. 1982 (hovei).

Paul, T.K. CNH-28122 (virginianum).

Pearson, A. p 43 (insanum).

Pedrono, ? 6 (insanum).

Pei Sheng-ji 9247 (barbisetum); 59-9253 (barbisetum); 59-10078 (praetermissum).

Pei, C. 2996 (violaceum).

Peng, C.-.I. 6576 (peikuoense); 16833 (peikuoense).

Peng, T.C. 2136 (pseudosaponaceum).

Pereira, J.T. 498 (capsicoides).

Perianayagam, S. RHT 12022 (virginianum).

Periaswamy, K. 19791 (wightii).

Perrottet, G.S. 289 (trilobatum); 905 (multiflorum).

Perumal, P. RHT-17881 (trilobatum); 21473 (violaceum); 21714 (torvum); RHT-22680 (trilobatum).

Pételot, A. 777 (violaceum); 1279 (procumbens); 1356 (praetermissum).

Phengkhlai, C. 4082 (torvum).

Phengklai, C. 12724 (involucratum); 12854 (involucratum).

Philcox, D. 10403 (trilobatum).

Phuong, V.X. HNK 314 (torvum).

Phusomsaeng, S. 36 (barbisetum).

Pierre, L. 3 (trilobatum); 6 (lasiocarpum); 9 (trilobatum); 92 (involucratum); 706 (melongena); 710 (lasiocarpum).

Pitopang, R. 1079 (torvum).

Pius, G. SAN-143601 (lasiocarpum).

Playfair, G.M.H. 75 (insanum); 310 (violaceum).

Pleyte, D.R. 613 (schefferi); 671 (lasiocarpum).

Podlech, D. 17424 (virginianum).

Poilane, E. 17 (lasiocarpum); 69 (insanum); ch 85 (insanum); 99 (insanum); 184 (lasiocarpum); 2777 (robinsonii); 3245 (involucratum); 3916 (violaceum); 4993 (procumbens); 4995 (procumbens); 5320 (insanum); 5770 (lasiocarpum); 5781 (nienkui); 7280 (cyanocarphium); 8151 (insanum); 8933 (nienkui); 9082 (nienkui); 9529 (lasiocarpum); 9887 (nienkui); 11816 (lasiocarpum); 12277 (cyanocarphium); 13390 (procumbens); 13599 (violaceum); 14481 (involucratum); 14578 (trilobatum); 15590 (lasiocarpum); 16337 (procumbens); 16415 (praetermissum); 16836 (pseudosaponaceum); 16845 (pseudosaponaceum); 17959 (procumbens); 18866 (insanum); 18869 (insanum); 19554 (lasiocarpum); 19702 (involucratum); 20213 (violaceum); 20487 (barbisetum); 20877 (nienkui); 20943 (insanum); 20990 (violaceum); 21010 (insanum); 21657 (involucratum); 22494 (lasiocarpum); 22521 (lasiocarpum); 25787 (praetermissum); 26089 (insanum); 27167 (violaceum); 27848 (procumbens); 27887 (procumbens); 28113 (insanum); 28172 (praetermissum); 28332 (lasiocarpum); 28533 (insanum); 28854 (insanum); 29290 (procumbens); 32478 (violaceum).

Polunin, O. 815 (violaceum); 3607 (virginianum); 5705 (violaceum).

Pooma, R. 5485 (trilobatum); 6715 (trilobatum).

Pottier, ? 263 (insanum); 506 A (insanum); 576 (violaceum).

Powell, D.A. 62 (involucratum).

Powell, J.M. UPNG 1705 (torvoideum).

Prain’s collector 72 (torvum); 250 (torvum); 3855 (barbisetum).

Prasad, R. CC-31647 (insanum).

Praveen, K.K. PR 91 (virginianum); PR 92 (torvum); PR 93 (torvum).

Prawiroatmodjo, S. 1356 (sulawesi).

Premanath, R.K. ANC-5986 (torvum); ANC-8442 (torvum).

Prescott, T.A.K. 28 (torvum).

Project Soma GM-6 (lasiocarpum); XM-33 (lasiocarpum).

Pullaiah, T. 333 (pubescens).

Pulle, A. 3151 (lasiocarpum).

Pullen, R. 8060 (torvoideum).

Puri, G.S. 339 (hovei); 2939 (hovei); 6001 (hovei); 8481 (hovei); 9145 (hovei); 12599 (hovei); 14047 (hovei); 20056 (hovei).

Puri, V. 71 (violaceum); 478 (violaceum).

Purseglove, J.W. 4120 (torvum); 4260 (torvum).

Put Phraisurind 28 a (barbisetum); 1773 (barbisetum); 3227 (putii); 3683 (cyanocarphium); 4031 (barbisetum).

Quang, B.H. HN-NY 1213 (torvum).

Quisumbing, E. 45 x9 2066 (torvum); 82295 (torvoideum).

Rabil, N. 204 (barbisetum).

Raghavan, R.S. 62536 (multiflorum); 68102 (hovei); 74029 (multiflorum); 74160 (capsicoides); 74210 (multiflorum); 79628 (multiflorum); 90267 (multiflorum); WC-97232 (capsicoides); 103287 (hovei); 103974 (multiflorum).

Ragupathy, S. 1085 (trilobatum).

Rahayu, M. 318 (lasiocarpum).

Rahman’s collector 1124 (torvum).

Rahman, M.A. 98-108 (torvum); 98-165 (torvum); 4604 (torvum).

Raizada’s collectors 26031 (pubescens).

Raizada, M.B. 21109 (violaceum); 23149 (virginianum).

Rajamani, C. 76 (virginianum); 1992 (giganteum).

Rajan, R. 86240 (virginianum); 86353 (trilobatum).

Rajendran, A. RHT 12625 (torvum); 86731 (vagum).

Raji, D.C.S. 291 (trilobatum).

Raju, R.R.V. 3107 (melongena).

Raju, S.R. 6161 (pubescens); 20258 (multiflorum).

Ram, J.V.S. NC-39940 (violaceum).

Ram, S.B. SFS-2018 (virginianum).

Rama Rao, M. 571 (capsicoides); 2225 (capsicoides); 2312 (capsicoides).

Ramachandran, A. 0846 (wightii).

Ramachandran, V.S. 52352 (multiflorum); 54193 (multiflorum); 62156 (multiflorum); 62747 (multiflorum); 65364 (multiflorum).

Ramamurthy, K. 18177 (multiflorum); 20241 (pubescens); 22807 (trilobatum); 23370 (multiflorum); 25845 (trilobatum); SC-25846 (insanum); 49373 (multiflorum); SC-49388 (insanum); 50651 (pubescens); 51175 (trilobatum); 51218 (trilobatum); 52899 (trilobatum); SC-53624 (insanum); 53658 (trilobatum); SC-53660 (insanum); 60121 (trilobatum); 60369 (trilobatum); 64171 (trilobatum); 77363 (pubescens); SC-77438 (insanum); 77579 (pubescens); 79615 (insanum); SC-83835 (insanum); 84628 (pubescens); 84629 (pubescens); 86490 (trilobatum); 88834 (trilobatum).

Ramanandan, P. 4847 (torvum).

Ramanujam, M.P. MPR-106 (trilobatum); MPR-232 (insanum); MPR-233 (insanum); MPR-234 (trilobatum).

Ramaswami, M.S. 1688 (violaceum).

Ramesh, B.R. 501 (pubescens); 726 (pubescens); 1056 (pubescens); 1512 (lasiocarpum).

Ramesh, S.R. KFP-1879 (lasiocarpum).

Ramlanto, ? 60 (jamaicense); 863 (lasiocarpum); 865 (lasiocarpum).

Ramos, M. 623 (melongena); 1049 (torvoideum); 1820 (torvum); 1857 (lasiocarpum); 4792 (retrorsum); 4955 (insanum); 8091 (retrorsum); 15341 (aethiopicum); 20544 (torvoideum); 22426 (torvoideum); 22586 (lasiocarpum); 24376 (lasiocarpum); 26487 (retrorsum); 27004 (pseudosaponaceum); 33186 (retrorsum); 33235 (retrorsum); 34812 (lasiocarpum); 36583 (cyanocarphium); 36789 (torvoideum); 37058 (torvoideum); 37835 (torvoideum); 38020 (torvoideum); 39466 (lasiocarpum); 40197 (pseudosaponaceum); 43154 (cyanocarphium); 43228 (lasiocarpum); 43939 (lasiocarpum); 44333 (cyanocarphium); 45077 (retrorsum); 46482 (torvoideum); 46590 (retrorsum); 47243 (lasiocarpum); 49081 (lasiocarpum); 49141 (melongena); 49324 (schefferi); 79839 (miyakojimense); 85001 (lasiocarpum); 85142 (cyanocarphium).

Randeria, A.F. AR 18 (hovei); AR 113 (hovei); A.R. 385 (hovei).

Randeria, G.J. AR 50 (hovei); AR 282 (hovei); 29003 (hovei).

Rani, N. AHT-28282 (giganteum).

Rao Rolls, S. WC-92454 (insanum).

Rao, A.S. 7760 (hovei); 61707 (multiflorum); 74417 (multiflorum); 74452 (multiflorum); 77922 (hovei); 79761 (multiflorum); 79815 (multiflorum); 80001 (multiflorum); 80163 (multiflorum); WC-80447 (trilobatum); 81575 (hovei); 85335 (multiflorum); 85425 (multiflorum); 85784 (multiflorum); 85948 (multiflorum); 95633 (multiflorum); 131545 (hovei).

Rao, J.V.S. NC-39878 (insanum).

Rao, K.K. 74 (torvum).

Rao, P.S.N. ANC-15598 (torvum).

Rao, R.S. 66175 (hovei); 83455 (hovei); 84337 (hovei); 85243 (hovei).

Rao, T.A. CNH-561 (arundo); CNH-1453 (insanum); NC-1597 (violaceum); CNH-1949 (arundo); CNH-2086 (insanum); NC-3214 (violaceum); NC-3214 (violaceum); 4209 (trilobatum); 5614 (trilobatum); CNH-7234 (trilobatum); CNH-7441 (trilobatum); NC-10755 (insanum).

Rao, Y.V.S. 39878 (insanum).

Rashid, A. 3692 (elaeagnifolium).

Rau, M.A. 2414 (chrysotrichum); NC-14597 (chrysotrichum).

Razali, H. HR-1 (torvum).

Read, B.E. R 1340 (sisymbriifolium).

Ream, R.R. 440 (pseudosaponaceum).

Rechinger, K. 4821 (dunalianum).

Rechinger, K.H. 29735 (elaeagnifolium); 29752 (elaeagnifolium).

Reksodikardjo, S. 199 (dunalianum).

Remaudiere, G. 1797 (virginianum).

Rensch, I. 1501 (torvoideum).

Reodi, B. 84730 (hovei).

Reporter on Economic Products to the Govt. of India 11787 (barbisetum).

Revel-Macdonald, N. 49 (melongena).

Revisión de Plantas Vasculares Filipinas 1625 (torvoideum).

Reynoso 4379 (torvum); 24173 (torvum); 118706 (lasiocarpum).

Rice, C. 44 (virginianum).

Rich, H.H. 341 (virginianum); 496 (insanum).

Ridley, H.N. 7159 (chrysotrichum); 12383 (torvum); 14250 (involucratum).

Ridsdale, C.E. 5457 (lasiocarpum).

Rihu, ? 752 (violaceum).

Ritchie, C. 509 [a] (hovei); 509 [b] (violaceum).

Robbins, R.G. 79 (torvoideum); 808 (torvoideum).

Robinson, C.B. 285 (melongena); 286 (lasiocarpum); 287 (lasiocarpum); 1082 (robinsonii); 1153 (insanum).

Robinson, H.C. 38 (torvum).

Rock, J.F. 2339 (torvum).

Rolla, S.R. 66519 (violaceum); 69711 (hovei); 71716 (hovei); 73614 (multiflorum); 77625 (hovei); 86542 (hovei); 87491 (hovei); 88664 (hovei).

Rothé, ? 18 (lasiocarpum); 78 (procumbens).

Roy, A. CNH-11965 (insanum); CNH-14914 (trilobatum).

Roy, G.F. CC-44994 (insanum).

Roy, G.P. 2747 (melongena); AC-3950 (melongena); CC-43624 (melongena).

Royen, P. van 3136 (lasiocarpum); 4553 (dunalianum); 8103 (torvoideum).

Ruse, L.F. 142 (torvum).

Ryan, G.A. 313 (hovei).

Ryan, G.M. 1475 (hovei); 1488 (giganteum).

Safui, B. CNH-2488 (arundo).

Sahira 35825 (melongena).

Sahni, K.C. 563 (violaceum); 3418 (hovei); 6154 (hovei).

Saito, S. 6965 (peikuoense).

Saldanha, C.J. CS 5129 (multiflorum); CS 5129 A (multiflorum); CS 5130 (multiflorum); CS 6256 (multiflorum).

Sampath Kumar, V. SC-126916 (virginianum); SC-126918 (insanum); SC-126919 (torvum); SC-126920 (robustum); SC-126922 (sisymbriifolium); SC-126923 (viarum); SC-126932 (multiflorum); SC-126933 (multiflorum); SC-126937 (multiflorum); SC-126938 (sisymbriifolium); SC-126940 (torvum); SC-126941 (insanum); SC-126942 (elaeagnifolium); SC-126943 (torvum); SC-126944 (viarum); SC-126945 (violaceum); SC-126946 (torvum); SC-126947 (violaceum); SC-126949 (multiflorum); SC-126950 (multiflorum); SC-126951 (viarum); SC-126953 (multiflorum); SC-126958 (multiflorum); SC-126960 (multiflorum); SC-126962 (viarum); SC-126963 (multiflorum); SC-126964 (torvum); SC-126965 (violaceum); SC-126966 (violaceum); SC-126968 (virginianum); SC-126969 (torvum); SC-126970 (trilobatum); SC-126971 (insanum); SC-126972 (elaeagnifolium); SC-126973 (insanum); SC-126974 (torvum); SC-126975 (pubescens); SC-126976 (viarum); SC-126977 (torvum).

Sampson 13816 (procumbens).

Sanarthanam, K.P. NC-51468 (violaceum).

Sands, L.J.S. 6330 (lasiocarpum).

Sands, M.J.S. 477 (poka); 1384 (dunalianum).

Santapau, H. HS 589 (hovei); HS 746 (hovei); HS 1443 (hovei); HS 2035 (hovei); HS 2092 (hovei); HS 2093 (hovei); HS 2094 (hovei); 2972 (virginianum); 2973 (virginianum); HS 10212 (hovei); 11574 (hovei); HS 14461 (hovei); HS 14462 (hovei); HS 15830 (hovei); HS 16095 (hovei); HS 17291 (hovei); HS 17365 (hovei); HS 17516 (hovei); HS 17517 (hovei); HS 17579 (hovei); HS 18855 (hovei); HS 18880 (hovei); HS 18963 (hovei); HS 20767 (multiflorum); HS 22226 (hovei); HS 22458 (hovei); HS 23085 (multiflorum); HS 27337 (hovei); HS 27423 (hovei).

Santisuk, T. 468 (virginianum).

Santos, J.K. 4 (lasiocarpum); 30 26 (retrorsum).

Santos, J.V. 4147 (cyanocarphium); 5470 (pseudosaponaceum).

Santos, K. 37 (torvoideum).

Sanyal, M.N. 879 (melongena); 880 (melongena).

Saran, R. 306 (violaceum); 5367 (viarum).

Sarin, Y.K. NC-1491 (chrysotrichum); NC-5079 (insanum).

Sasaki, S. 275 [a] (pseudosaponaceum).

Sastry, A.R.K. CNH-9264 (insanum); BSI-40896 (violaceum).

Satyanarayana, P. 122410 (pubescens).

Saulière, A. 52 bis (multiflorum); 370 bis (multiflorum).

Savinierre, E. de la 343 (poka); 1171 (torvum).

Saxton, W.T. 499 (cordatum).

Sayers, C.D. NGF-21505 (torvoideum).

Schierbrand, W.C. von 137 (pseudosaponaceum).

Schiffner, V. 2504 (involucratum); 2517 (jamaicense); 2518 (involucratum).

Schlagintweit, H.A.R. von 3220 (virginianum); 4409 (melongena); 10480 (virginianum).

Schlechter, F.R.R. 13749 (dunalianum); 17627 (schefferi).

Schmid, M. 1084 (violaceum); 1087 (insanum); 1608 (insanum).

Schmutz, E. 702 (lasiocarpum).

Schneider, C. 2545 (torvum).

Schodde, R. 2141 (torvoideum); 4244 (dunalianum).

Schomburgk, R. 217 (insanum).

Scott, D.r. 32 (hovei).

Sebastine, K.M. 540 (pubescens); 1195 (trilobatum); SC-2038 (robustum); 3726 (vagum); 4539 (vagum); 5181 (trilobatum); 5898 (vagum); 8523 (vagum); 9640 (vagum); 9729 (pubescens); SC-11666 (insanum); 12510 (pubescens); SC-14577 (insanum); 14589 (pubescens); 18433 (multiflorum).

Second Darwin Nepal Fieldwork Training Expedition B-38 (torvum).

Sedgwick, L.J. 3939 (virginianum); 4189 (giganteum); 4745 (giganteum); 5654 (giganteum); 6085 (torvum); 7281 (torvum).

Sengupta, G. 35 (violaceum); 145 (trilobatum); 924 (violaceum); 974 (viarum); 1205 (viarum); 1326 (violaceum); 14592 (melongena).

Shah, G.L. 812 (hovei); 9041 (hovei); 9122 (hovei).

Sharifi, M.R. 313 (virginianum); 508 (virginianum).

Sharma, B.D. 35638 (multiflorum); 35953 (multiflorum); 36100 (wightii); 42430 (multiflorum); 167305 (hovei); 167857 (hovei).

Sharma, J.P. NC-79451 (chrysotrichum).

Sharma, S. SS-93 (insanum).

Shea, G. SAN-75994 (lasiocarpum).

Shein, L. 88370 (torvum).

Shenoy, K.V. K.S.V. 11 (hovei); K.S.V. 94 (hovei); K.S.V. 168 (hovei); K.S.V. 553 (hovei); K.S.V. 1960 (hovei); K.S.V. 1997 (hovei); K.S.V. 2124 (hovei); K.S.V. 3822 (hovei); K.S.V. 5613 (hovei).

Shetty, B.V. AC-2272 (forskalii); 10349 (multiflorum); 11915 (multiflorum); 26461 (multiflorum); 27932 (pubescens); 27980 (vagum); 37663 (multiflorum).

Shimizu, T. T 8257 (torvum).

Shine, L. 96319 (torvum); 96349 (violaceum).

Shukla, A.N. NC-122192 (insanum).

Shukle, U. NC-70031 (violaceum); NC-70264 (violaceum).

Shuter, J. 1580 (trilobatum).

Si Boeea, R. 6286 (lasiocarpum); 6904 (melongena); 7097 (violaceum); 7740 (lasiocarpum); 8015 (torvum); 8741 (lasiocarpum); 8919 (melongena); 8922 (lasiocarpum); 9035 (lasiocarpum); 9225 (melongena).

Sibil, J. 286 (melongena).

Siddiqi, M.A. 6104 (insanum).

Sidiyasa, K. 1293 (lasiocarpum); BRF 1773 (lasiocarpum).

Sieber, F.W. 227 (violaceum).

Sikdar, J.K. CNH-36 (torvum); 709 (melongena).

Sillitoe, P. 50 (torvoideum).

Silva, F. de Wall. Cat. 2628 e (barbisetum).

Simond, ? 77 (violaceum).

Simpson, N.D. 8050 (insanum); 8052 (torvum); 8755 (violaceum); 8890 (giganteum); 9196 (capsicoides); 9323 (trilobatum); 9344 (vagum).

Sinclair, J. 186 (melongena); 5153 (torvum); 9778 (lasiocarpum); 10889 (melongena).

Singh, A.N. AC-5742 (insanum); AC-7051 (melongena).

Singh, H. 497 (torvum).

Singh, N.P. 124144 (hovei); 124560 (hovei); 124704 (hovei); 124800 (hovei); 124875 (hovei); 125338 (hovei).

Singh, P. BSHC-15539 (violaceum).

Singh, R. 83 (violaceum).

Singh, U. 11 (violaceum); 99 (violaceum); 363 (violaceum); 365 (insanum).

Singh, V. AC-2889 (insanum); AC-2978 (virginianum); AC-5503 (insanum); AC-7263 (insanum); 80328 (elaeagnifolium).

Sino-American Botanical Expedition 15 (violaceum); 338 (violaceum).

Sitinand, T. 4307 (praetermissum).

Smith, E. 046 (procumbens); 478 (lasiocarpum); 4018 (lasiocarpum); 15398 (capsicoides).

Smith, J.F. 2 (torvum).

Smitinand, T. 3377 (violaceum); 4807 (praetermissum); 7421 (barbisetum).

Soenarko, S. 298 (lasiocarpum).

Sohmer, S.H. 8333 (giganteum); 8555 (giganteum); 8756 (torvum).

Soibeh, D. 773 (melongena).

Soll, S. 252 (cordatum).

Song, H.M. 93 (miyakojimense).

Soosairaj, S. 2514 (wightii); 2514 (wightii).

South Vietnam First Darwin Expedition 85 (involucratum).

Sowastava, G. F.R.I. 116 (violaceum).

Sparrow, C.E.H. 46 (elaeagnifolium).

Spire, C.J. 126 (lasiocarpum); 914 (insanum); 1080 (lasiocarpum); 1081 (insanum); 1426 (violaceum).

Sprague, T.A. 13 (virginianum); 57 (virginianum).

Squires, R.W. 27 (procumbens); 105 (torvum); 282 (insanum).

Sreemadhavan, C.P. CPS 173 (pubescens).

Sreenath, K.P. KFP-10225 (trilobatum).

Srinivaran, S.R. SC-51515 (robustum).

Srinivasan, S.R. 89592 (pubescens).

Srisanga, P. 3832 (barbisetum); 97239 (torvum); 97280 (viarum); 97698 (torvum); 97769 (viarum).

Staff (Northern Centre) NC-1039 (chrysotrichum).

Stainton, J.D.A. 21 (torvum); 25 (virginianum); 552 (viarum); 2306 (virginianum); 2413 (viarum); 4830 (violaceum); 5126 (viarum); 5310 (violaceum); 6753 (violaceum); 6797 (torvum); 7615 (viarum); 8223 (torvum); 8749 (violaceum).

Stearn, W.T. 24 (virginianum); 32 (cordatum).

Steenis, C.G.G.J. van 18309 (procumbens).

Steiner, M.L. 864 (torvoideum).

Stephenson, T.A. 531 (torvum).

Sterly, J. 80-215 (torvoideum).

Stevens, P.F. LAE-58631 (lasiocarpum).

Steward, A.N. 1144 (capsicoides).

Stewart, J.L. 334 (cordatum); 2404 (violaceum).

Stewart, R.R. 230 (insanum); 397 (virginianum); 1071 (insanum); 13612 (cordatum); 15052 (torvum); 19349 A (virginianum).

Stocks, J.E. 252 (cordatum).

Stone, B.C. PPI 688 (torvoideum); 14063 (torvum).

Strachey, R. 2 (virginianum); 5 (violaceum); 7 (insanum).

Streimann, H. 8498 (lasiocarpum); NGF-27523 (dunalianum); LAE-52624 (dunalianum).

Subba Rao, G.V. 2442 b (virginianum); SC-20166 (insanum); SC-22279 (insanum); SC-24413 (insanum); 31844 (pubescens); 36571 (multiflorum); 37340 (pubescens); 37470 (pubescens); 45801 (pubescens); 46813 (pubescens); SC-46980 (insanum).

Subramanian, K.N. 149 (trilobatum); 554 (pubescens); 736 (pubescens); 1608 (pubescens); 1807 (pubescens); 2330 (pubescens); 3348 (pubescens); 5113 (trilobatum); 5309 (trilobatum); 5594 (vagum); 6386 (pubescens); 10413 D (viarum); 14065 (pubescens).

Sudheresan, C. 429 (multiflorum).

Sugau, J.B. 337 (lasiocarpum).

Sulit, M.D. 1302 7079 (wrightii); 7450 (pseudosaponaceum); 7599 (torvoideum); 8502 (lasiocarpum); 9868 (torvoideum); 13802 (lasiocarpum); 16790 (pseudosaponaceum); 17133 (lasiocarpum).

Sumithraarachchi, D.B. 08S 536 (virginianum); DBS-912 (giganteum).

Suppiah, T. FRI-14773 (lasiocarpum).

Susami, A. 1151 (violaceum).

Suzana, S. SAN-151080 (lasiocarpum).

Suzuki, M. 88-20084 (viarum); 88-20133 (violaceum); 88-60006 (viarum); 88-80032 (viarum).

Symon, D.E. 10657 (torvum); 10669 (torvoideum); 13856 (torvoideum); 14318 (elaeagnifolium); 15128 (elaeagnifolium).

Sørensen, T.J. 77 (trilobatum); 1569 (torvum); 1982 (trilobatum).

Taam, Y.W. 1759 (torvum); 1831 (insanum).

Tadong, D. 102 (lasiocarpum); 382 (lasiocarpum).

Tagawa, M. T-3160 (torvum).

Tai, L.Y. 11447 (pseudosaponaceum).

Takahashi, H. T 62798 (torvum); T 63068 (torvum).

Takeuchi, W.N. 9299 (dunalianum); 9323 (dunalianum); 12549 (torvoideum); 16958 (torvum); 17830 (lasiocarpum); 23333 (lasiocarpum).

Takushi, A. 6400 (miyakojimense).

Tanaka, N. 20306 (torvum); 20352 (virginianum); 23309 (viarum); 23310 (torvum); 30877 (melongena).

Tanaka, T. 5044 (capsicoides).

Tang Siu Ging, ? 15213 (pseudosaponaceum); 16169 (pseudosaponaceum); 16346 (pseudosaponaceum).

Tao Guoda 14666 (barbisetum); 16747 (lasiocarpum); 17505 (barbisetum); 17509 (praetermissum); 43763 (lasiocarpum); 44995 (barbisetum); 47460 (praetermissum); 47466 (praetermissum); 930327 (praetermissum).

Taquet, E.J. 1155 (melongena).

Tateishi, Y. 15125 (miyakojimense).

Tavakari, S.C. T 151 (hovei); T 1268 (hovei).

Taylor, P.M. P 112 (melongena); P 344 (melongena).

Tehan Uji 2793 (lasiocarpum).

Teijsmann, J.E. 7853 (schefferi); 7854 (dunalianum).

Telado, ? 1920 (melongena).

Thanikaimoni, G. G.T. 1231 (trilobatum).

Thesiger, W. 1667 (virginianum).

Thomson, G. 10 (multiflorum); 15 (insanum); 16 (insanum); 178 (melongena).

Thomson, T. 59 (violaceum); 304 138 (violaceum); 518 (virginianum); 578 (virginianum).

Thorel, C. 23 (insanum); 84 (lasiocarpum); 1419 (cyanocarphium); 3410 b (procumbens); 3410 a (procumbens); 9074 (torvum).

Thothathri, K. 10331 (viarum).

Thwaites, G.H.K. 1503 (giganteum); 1901 (vagum); 1903 (virginianum); 1903 [b] (giganteum); 1903 [c] (giganteum); 1904 (violaceum); 1905 (virginianum); 1906 (trilobatum); 1907 (insanum); 2868 (lasiocarpum).

Tirvengadum, D.D. 190 (violaceum).

Tiwari, G.H. AC-941 (forskalii).

To Kang Peng 1146 (procumbens); 2738 (procumbens).

Tomardhamam, K.P. 51506 (insanum).

Tong Shaoquan 32872 (praetermissum).

Toppin, S. 2105 (insanum).

Toroes, R. si 1639 (cyanocarphium); 1642 (lasiocarpum); 2848 (lasiocarpum); 2926 (cyanocarphium); 5510 (lasiocarpum).

Townsend, C.C. 128 (wightii).

Treutler, W.J. 20 (violaceum); 1257 (virginianum).

Tribedi, G.N. 514 (insanum); Pharm-594 (robustum); CNH-1409 (trilobatum); CNH-1435 (trilobatum).

Tsai, H.T. 53448 (violaceum).

Tsam, Y.W. 1831 (insanum).

Tsang, W.T. 30 (procumbens); 137 (procumbens); 161 (procumbens); 207 (insanum); 409 (pseudosaponaceum); 703 (melongena); 811 (pseudosaponaceum); 874 (pseudosaponaceum); 16102 (lasiocarpum); 17668 (procumbens); 21896 (violaceum); 23892 (violaceum); 23902 (pseudosaponaceum); 30055 (insanum); 30369 (violaceum).

Tsiang Ying (=Tsiang, Y.) 847 (torvum); 877 (procumbens); 2232 (procumbens); 2733 (violaceum); 2984 (lasiocarpum).

Tsiang, Y. 459 (torvum); 877 (procumbens); 914 (procumbens); 2733 (violaceum); 7217 (violaceum).

Tso, C.L. 23004 (insanum).

Tsugaru, S. 30162 (melongena); 61752 (torvum).

Tsvelev, N.N. 1302 (virginianum).

Tuerk, M. 5 (viarum).

Uniyal, B.P. NC-37151 (insanum); NC-77338 (insanum); NC-80081 (violaceum); NC-81753 (insanum); NC-92623 (insanum).

USA Typhus Commission 284 (torvum).

Usha Nanda, ? USHA 142 (hovei).

Vajravelu, E. SC-19071 (insanum); SC-20505 (insanum); 20582 (pubescens); 20767 (trilobatum); 24392 (pubescens); 26109 (multiflorum); SC-27842 (insanum); 33831 (vagum); 33926 (vagum); 33966 (pubescens); 35170 (multiflorum); 36726 (pubescens); 36827 (multiflorum); 38755 (pubescens); 39423 (pubescens); 39431 (vagum); 39748 (multiflorum); 39793 (multiflorum); 43559 (wightii); 44312 (pubescens); 44891 (multiflorum); 49813 (multiflorum); 51801 (pubescens); 57958 (pubescens); 62898 (multiflorum); 62910 (multiflorum); SC-77822 (capsicoides).

Vanoverbergh, M. 30 (torvoideum); 611 (torvoideum); 1979 (insanum); 2020 (torvoideum); 2027 (lasiocarpum).

Vartak, V.D. 5556 (hovei).

Vasavada, J.A. 4844 (hovei).

Veldkamp, J.F. 7032 (torvoideum); 7852 (torvum).

Venkanna, P. 5562 (trilobatum).

Venkata Reddi, B. 93139 (hovei); 93237 (hovei); 93499 (hovei); 96093 (hovei); 97969 (hovei).

Venugopal, N. RHT 15880 (pubescens); RHT-21446 (virginianum).

Verheijen, J.A.J. 2307 (procumbens).

Versteegh, C. BW-8330 (lasiocarpum).

Vidal, J.E. 1802 (insanum); 1803 (insanum); 1804 (lasiocarpum); 5438 (lasiocarpum); 5855 (praetermissum).

Vidal, S. 1623 (torvoideum); 1624 (retrorsum); 1625 (torvoideum); 3363 bis (torvoideum); 3363 (torvoideum); 3365 (lasiocarpum); 3366 (retrorsum); 3368 (retrorsum).

Vinas, A. 302 (lasiocarpum).

Vine, R.S. 176 (wightii); 177 (violaceum); 178 (sisymbriifolium); 180 (robustum).

Viswanathan, M.V. MVV 92 (pubescens); NC-61592 (violaceum).

Vivekananthan, K. SC-20388 (capsicoides); SC-22974 (capsicoides); SC-29328 (capsicoides); 42938 (multiflorum); 45300 (multiflorum); SC-46694 (capsicoides).

Vogel, E.F. de 2504 (poka).

Vohra, J.N. NC-60310 (insanum).

Voigt, J.O. 281 (violaceum).

Voogd, C.N.A. de 2177 (graciliflorum).

Vratak, V.D. 8564 (elaeagnifolium).

Waas, S. 439 (torvum); 467 (giganteum).

Wadhwa, B.M. 5219 (forskalii); 5314 (forskalii); CC-8283 (insanum); NC-37197 (chrysotrichum); NC-57713 (violaceum); NC-62734 (insanum); NC-62909 (violaceum); 64027 (hovei); 67378 (hovei); 109821 (hovei); 127608 (hovei); 128061 (hovei); 128221 (hovei).

Wadwha, R.M. CC-5437 (insanum).

Wagh, S.K. 3681 (pubescens); 3683 (pubescens); 5950 (pubescens); 5951 (pubescens); 5952 (pubescens); 6001 (pubescens); SKW 7665 (multiflorum).

Walker, G.W. 267 (violaceum).

Wallich, N. cat.Burm. 9 (lasiocarpum); cat.Burm. 9 (lasiocarpum); cat.Burm. 135 (insanum); cat.Burm. 176 (violaceum); cat.Burm. 210 (lasiocarpum); cat.Burm. 255 (violaceum); Cat. 262 (torvum); cat.Burm. 465 (virginianum); Cat. 1041 (virginianum); cat.Burm. 1328 [a] (barbisetum); cat.Burm. 1328 [b] (barbisetum); Cat. 2610 b (giganteum); Cat. 2610 a (giganteum); Cat. 2612 B (virginianum); Cat. 2622 b (trilobatum); Cat. 2623 c (lasiocarpum); Cat. 2623 b (lasiocarpum); Cat. 2623 d (lasiocarpum); Cat. 2626 a (violaceum); Cat. 2626 d (violaceum); Cat. 2626 f (insanum); Cat. 2626 e (multiflorum); Cat. 2627 b (torvum); Cat. 2627 a (torvum); Cat. 2627 e (torvum); 2627 (torvum); 2627 (torvum); Cat. 2628 b (melongena); Cat. 2628 f (cyanocarphium); cat. 2628 d (melongena); cat. 2628 K (violaceum); cat. 2628 K [2] (melongena); Cat. 2629 b (pubescens); Cat. 2629 [a] (pubescens).

Wallkes, J.V.B. 2695 (lasiocarpum).

Walsh, M.E. 367 (procumbens); 440 (procumbens); 455 (torvoideum).

Wang, C. 32726 (nienkui); 32860 (procumbens); 32863 (melongena); 32965 (pseudosaponaceum); 32999 (lasiocarpum); 33733 (pseudosaponaceum); 35416 (procumbens); 36176 (pseudosaponaceum); 36464 (procumbens); 38866 (pseudosaponaceum).

Wang, C.-.M. 4317 (peikuoense); 4376 (peikuoense); 4720 (peikuoense); 5514 (peikuoense).

Wang, C.W. 76607 (barbisetum); 85018 (violaceum).

Wang, J.-.C. 11628 (peikuoense).

Wang, Y.-.M. 190 (viarum).

Wanghong 2622 (barbisetum).

Warburg, O. 15069 (lasiocarpum); 15072 (schefferi); 15844 (sulawesi); 21251 (dunalianum).

Warwick, M. MW-15 (lasiocarpum).

Waterhouse, J.H.L. 298 (torvum).

Watt, G. 7454 (violaceum); 7608 (violaceum); 9778 (insanum); 9819 (violaceum).

Watters, T. 7565 (violaceum).

Weinland, K. 133 (dunalianum).

Wen, J. 10190 (lasiocarpum).

Wendelbo, P. W 7412 (virginianum).

Wheeler, J.M. ANU 5987 (torvoideum); ANU 6451 (torvoideum).

White, C.T. 369 (torvoideum).

Whitford, H.N. 980 (retrorsum).

Whiting, M.M. 103 (procumbens).

Wiakabu, J. LAE-70377 (wrightii); LAE-70450 (dunalianum); LAE 73480 (lasiocarpum).

Widjaja, E.A. 1877 (lasiocarpum).

Widmer, M. 41 (violaceum); 106 (lasiocarpum).

Wight, R. 345 (torvum); 585 (lasiocarpum); 586 (multiflorum); 587 (violaceum); 588 (violaceum); 694 (hovei); 1571 (giganteum); 1573 Aa (melongena); 1573 AB (virginianum); 1573 A (melongena); 1573 Ab (insanum); 1573 Ab (insanum); 1573 c (insanum); 1574 (lasiocarpum); 1575 (multiflorum); 1576 /126 (wightii); 1577 (torvum); 1578 (violaceum); 1579 (virginianum); 1580 (trilobatum); 1581 (pubescens); 2007 (trilobatum); 2011 (pubescens); 2013 (virginianum); 2015 (violaceum); 2016 (wightii); 2017 (trilobatum); 2018 (torvum); 2019 (lasiocarpum); 2019 [a] (multiflorum); 2020 (insanum); 2022 (giganteum); Wall. cat. 2624 b (vagum); Wall.cat. 2629 (pubescens); 5906 (virginianum).

Wijewickrama, T. MO 41 (vagum).

Wilde, W.J.J.O. de 12693 (lasiocarpum); 12799 (violaceum); 19835 (lasiocarpum); 21306 (lasiocarpum).

Wilfords, ? 83 (insanum).

Williams, L.H.J. 61 (violaceum); 62 (torvum); 8102 (viarum); 8223 (torvum); 8268 (virginianum); 8628 (virginianum).

Williams, R.S. 1074 (retrorsum); 1149 (torvoideum).

Wilson, E.H. 2001 (virginianum); 4197 (violaceum); 4198 (virginianum); 9657 (peikuoense).

Winit, P. 1424 (barbisetum).

Winkler, H.J.P. 163 (cyanocarphium).

Wiriadinata, H. 20 (jamaicense); 428 (lasiocarpum); 590 (torvum); 1198 (torvum).

Womersley, J.S. 3408 (torvum); NGF-11021 (lasiocarpum); NGF-37340 (torvoideum).

Wongprasert, T. 997-129 (barbisetum).

Woo, J.P.W. 391 (insanum).

Wood, J.J. C 11 (virginianum); 121 (violaceum).

Woods, P.J.B. 176 (dunalianum).

Wright, C. 7 (virginianum); 348 (lasiocarpum); 349 (insanum); 489 (wrightii).

Wu, M.J. 976 (violaceum).

Wu, S.H. 1273 (peikuoense).

Wuang, C.T. 451 (violaceum).

Wykeham Perry, W. 503 (insanum).

Xiam Expedition 32 (violaceum); 032 (violaceum); 54 (insanum); 56 (capsicoides).

Xuyen, D. HN-NY 357 (torvum).

Yagermani 777 (torvum).

Yang, S.-.Z. 23750 (peikuoense).

Yang, T.Y.A. 1574 (lasiocarpum); 3932 (miyakojimense); 8701 (miyakojimense); 8701 (miyakojimense); 10063 (lasiocarpum); 11473 (miyakojimense); 15044 (miyakojimense).

Yapp, R.H. 148 (torvum).

Yates, H.S. 1175 (violaceum); 1361 (lasiocarpum); 1405 (poka).

Yati, H.A. 412 (torvum).

Yeshoda, K. 172 (violaceum); 413 (pubescens).

Yesoda, N. 703 (pubescens); 1088 (pubescens).

Yi Zhong 3978 (lasiocarpum).

Yip, H.G. 142 (violaceum); 452 (torvum).

Yoshida, S. 2432 (lasiocarpum).

Yu, T.T. 1352 (virginianum); 14891 (violaceum); 15962 (violaceum).

Yunnan expedition 5005 (violaceum).

Zeng Pei 13316 (nienkui).

Zhang Jianhou 13639 (lasiocarpum).

Zimmerman, R. 55 (torvum).

Zimmermann, R. 17 (insanum); 25 (melongena); 35 (torvum); 56 (trilobatum).

Zollinger, H. II-129 (involucratum); 139 (involucratum); 212 [a] (torvum); 369 (pseudosaponaceum); 529 (capsicoides); 698 (insanum); 702 (melongena); 1018 (cyanocarphium); 1125 (torvoideum); II-2648 [b] (violaceum); II 2648 [a] (insanum); 2660 (insanum); 2907 (graciliflorum); 3369 z (torvum); II 3773 7c (melongena); 3773 (melongena); III-3825 (cyanocarphium); 26482 (insanum).

Zwickey, A.L. 52 (torvoideum); 159 (mammosum); 405 (pseudosaponaceum).

## Supplementary materials

Supplementary material 1 Index to all numbered collections Data type Occurrences (pdf. file) Explanation note Index of all herbarium collections seen for this mongraph. https://binary.pensoft.net/file/689777This dataset is made available under the Open Database License (http://opendatacommons.org/licenses/odbl/1.0/). The Open Database License (ODbL) is a license agreement intended to allow users to freely share, modify, and use this Dataset while maintaining this same freedom for others, provided that the original source and author(s) are credited.Xavier Aubriot, Sandra Knapp

Supplementary material 2 Tropical Asian specimens examined Data type Specimens (csv. file) Explanation note A searchable csv file of all herbarium specimens collected in tropical Asia seen for this monograph. https://binary.pensoft.net/file/689778This dataset is made available under the Open Database License (http://opendatacommons.org/licenses/odbl/1.0/). The Open Database License (ODbL) is a license agreement intended to allow users to freely share, modify, and use this Dataset while maintaining this same freedom for others, provided that the original source and author(s) are credited.Xavier Aubriot, Sandra Knapp

Supplementary material 3 All specimens examined Data type Specimens (csv. file) Explanation note A searchable csv file of all specimens examined (worldwide) for this monograph. https://binary.pensoft.net/file/689779This dataset is made available under the Open Database License (http://opendatacommons.org/licenses/odbl/1.0/). The Open Database License (ODbL) is a license agreement intended to allow users to freely share, modify, and use this Dataset while maintaining this same freedom for others, provided that the original source and author(s) are credited.Xavier Aubriot, Sandra Knapp
