# Supplementary material for: ﻿A revision of the “spiny solanums” of Tropical Asia (Solanum, the Leptostemonum Clade, Solanaceae)
Source: PhytoKeys. 2022 Jun 1;198:1–270. doi: 10.3897/phytokeys.198.79514 (PMC9849010; doi:10.3897/phytokeys.198.79514)
Supplement: Supplementary material 1 — Index to all numbered collections [file phytokeys-198-001_article-79514__-s001.pdf]

# Supplementary Material 1

### Index to Numbered Collections

Only first collectors in collections made by two or more collectors are listed here. Collections by anonymous collectors without date or other identifying features are not listed. These and full collector strings can be found in the Supplementary Material searchable files (Suppl. Mat. 2 and 3). These files are also available on the NHM Data Portal (https://doi.org/10.5519/0rqfzvgd).

Aban Gibot SAN-94555 (lasiocarpum); SAN-94555 (lasiocarpum); SAN-99310 (lasiocarpum); SAN-99536 (lasiocarpum).
Abd El Ghani, M. 6908 (elaeagnifolium).
Abdallah, R. 377 (robustum).
Abdul, K. bin A. 2854 (lasiocarpum).
Abedin, S. 9419 (virginianum).
Abraham, V. 2 (insanum); 176 (trilobatum); 239 (trilobatum); 279 (insanum).
Abrams, L. 6607 (elaeagnifolium).
Abulaila, K. JOR 3 -1 (elaeagnifolium).
Acevedo Rodríguez, P. 16215 (torvum).
Acevedo Rosas, R. 32 (elaeagnifolium).
Achten, L. 533 (giganteum).
Ackermann, M. 218 (elaeagnifolium).
Aclaud, R.D. ACK 793 (hovei).
Acocks, J.P.H. 11519 (giganteum); 15926 (sisymbriifolium).
Acosta, L. 448 (chrysotrichum).
Acosta, L.E. 448 (chrysotrichum).
Acosta-Solís, M. 7795 (sisymbriifolium); 11327 (sisymbriifolium); 12941 (sisymbriifolium).
Adam, J.G. 20453 (torvum).
Adames, P. 527 (torvum).
Adams, C.D. 6783 (capsicoides); 7322 (jamaicense); 10339 (capsicoides); 10340 (jamaicense); 10371 (mammosum).
Adamson, T.G. 6 (giganteum).
Adan, H. 107 (arundo).
Adduru, M. 62 (retrorsum).
Adeka, R. 15 KA (arundo); 158 (arundo).
Adetunji, M.O. 34038 (wrightii).
Adeyanju, M.A. 7 (torvum).

Aedo, C. 10789 (melongena).

Aët 581 (lasiocarpum).

Agra, M.F. 648 (capsicoides); 662 (capsicoides); 684 (capsicoides); 697 (capsicoides); 745 (capsicoides); 1119 (capsicoides); 1247 (torvum); 1248 (torvum); 1267 (capsicoides); 1295 (torvum); 1329 (capsicoides); 1477 (torvum); 1524 (capsicoides); 2236 (torvum); 2258 (capsicoides); 2403 (capsicoides); 2817 (capsicoides); 5355 (robustum); 5358 (robustum); 6756 (sisymbriifolium); 6758 (torvum); 7006 (torvum); 7228 (robustum); 7237 (viarum); 7347 (robustum).

Aguayo, A. 444 (robustum).

Aguilar C, A. 164 (elaeagnifolium).

Águilar M, G. 9189 (torvum).

Aguilar, L.M. 912 (elaeagnifolium).

Aguilar, R. 1571 (torvum); 3821 (chrysotrichum); 4792 (jamaicense); 4796 (torvum).

Aguilar, R.M. 425 (elaeagnifolium); 555 (elaeagnifolium).

Aguirre T, S. 45 (elaeagnifolium); 116 (elaeagnifolium); 151 (elaeagnifolium); 259 (elaeagnifolium).

Aguirre, ? 173 (chrysotrichum).

Ahamed, S.M. KFP-282 (violaceum).

Ahern's collector 2161 (torvoideum); 2436 (lasiocarpum).

Ahles, H.E. 56834 (elaeagnifolium).

Ahmad, S. SA-483 (lasiocarpum).

Ahmed, H. 9842 (melongena).

Ahuja, B.S. 19607 (multiflorum); 47366 (hovei).

Ahuja, K.K. 87297 (hovei).

Aitchison, J.E.T. 482 (virginianum); 521 (cordatum).

Akogo, M. 49 (torvum).

Akpabla, G. 180 (torvum).

Akpabla, G.K. 1860 (wrightii).

Alba, A. de 45 (elaeagnifolium).

Albert de Escobar, L. 310 (jamaicense); 633 (torvum); 1468 (virginianum).

Albuquerque, B.W.P. de 699 (jamaicense).

Alcasid, G.L. 1885 (pseudosaponaceum).

Alfaro V, E. 768 (chrysotrichum).

Alfaro, E. 202 (jamaicense); 768 (chrysotrichum).

Ali, S.I. 1183 (cordatum).

Allard, H.A. 14501 (torvum).

Alleizette, C. d' 5 (violaceum).

Allen, J.B. 260 (arundo); 366 (cordatum).

Almeda, F. 2113 (chrysotrichum); 5170 (chrysotrichum); 5289 (capsicoides); 8147 (torvum).

Almeyda L, H. 23 (chrysotrichum).

Alston, A.H.G. 448 (violaceum); 14609 (lasiocarpum); 16158 (retrorsum).

Alstrup, V. 41 (forskalii).

Altamirano, F. 1736 (elaeagnifolium).

Álvarez M, D. 5990 (torvum).

Álvarez, R. 401 (elaeagnifolium); 499 (elaeagnifolium); 573 (elaeagnifolium); 631 (elaeagnifolium); 989 (elaeagnifolium).

Alverson, W.S. 189 (jamaicense); 427 (jamaicense).

Ambriansyah, A.A. 193 (jamaicense); 657 (lasiocarpum); 687 (melongena); 1656 (jamaicense); 3068 (jamaicense).

Ambrosetti, J.A. 1418 (elaeagnifolium); 1472 (elaeagnifolium); 1480 (elaeagnifolium); 1481 (elaeagnifolium); 1483 (elaeagnifolium).

Amin, A. SAN-111132 (lasiocarpum); SAN 117381 (lasiocarpum).

Amin, G. SAN-96560 (lasiocarpum).

Amith, J.D. 2542 (capsicoides); 2581 (chrysotrichum); 2635 (jamaicense); 2637 (capsicoides).

Anang 88 (lasiocarpum).

Anders, O. 2978 (virginianum); 3560 (virginianum); 4850 (virginianum).

Anderson, E.F. 5233 (torvum).

Anderson, J. 92 (sisymbriifolium).

Anderson, R.C. 51 (jamaicense).

Anderson, T. 1021 (insanum).

Anderson, W.R. 3504 (elaeagnifolium); 36268 (robustum).

Andoh, J.E. 4419 (torvum); 5267 (torvum).

Andrade C, M.G. 210 (jamaicense).

Andrade, B.O. 113 (aculeatissimum).

Andrews, C. 660 (sisymbriifolium).

Andrews, C.W. 97 (involucratum); 99 (involucratum).

Andrews, F.W. A 328 (trilobatum); A 355 (sisymbriifolium).

Andru, J. 4938 (torvum).

Anglade, L. 824 (pubescens).

Angus, A. 2878 (torvum); 2880 (chrysotrichum); 3681 (chrysotrichum).

Anilkumarin, Y. 951 (multiflorum).

Annandale, N. 1288 (trilobatum).

Annari, R. SC-67984 (insanum).

Ansari, M.Y. WC-63735 (insanum); 67512 (hovei); 87373 (hovei); 87766 (hovei); 94279 (hovei); 99990 (hovei); 104715 (hovei); 122027 (hovei); 124133 A (hovei); 127073 (hovei); 127202 (hovei); 146866 (multiflorum).

Ansari, R. ANC-1353 (torvum).

Anstead, R.D. 2 (torvum); 120 (trilobatum); 146 (torvum).

Antony, V.T. 78 (capsicoides); 221 (multiflorum).

Anupama, K. AP 179 (trilobatum); AP 299 (virginianum); 1P 729 (trilobatum).

Apolinaire-Maria 47 (jamaicense).

Arakaki, M. 83 (chrysotrichum).

Aranha, C. 25 (mammosum).

Araque M, J. 19Ar 341 (elaeagnifolium).

Araújo-M, A. 2244 (sisymbriifolium); 2296 (wrightii).

Araya, F. 109 (torvum).

Arbo, M.M. 900 (sisymbriifolium); 1978 (robustum).

Archer, W.A. 2047 (jamaicense); 2903 (mammosum); 2904 (capsicoides); 8127 (jamaicense).

Arenas, P. 2360 (elaeagnifolium).

Argañaras, J.L. 51 (elaeagnifolium).

Argent, G.C.G. 108279 (lasiocarpum).

Argüelles, E. 23 (elaeagnifolium); 2468 (elaeagnifolium).

Arias, L.A. 39 (jamaicense).

Ariza Espinar, L. 1575 (elaeagnifolium); 2393 (elaeagnifolium); 3587 (elaeagnifolium); 3588 b (elaeagnifolium).

Arnoldo-Broeders, M. 3621 (elaeagnifolium).

Aron, C.M. CC-2702 (insanum).

Arora, C.M. NC-37130 (violaceum); UK-37131 (chrysotrichum); NC-38865 (insanum).

Aroy, R.K. 11677 (multiflorum); 12130 (multiflorum); 12168 (multiflorum); 12229 (hovei).

Arroyo P, L. 1818 (sisymbriifolium); 3029 (sisymbriifolium); 5561 (sisymbriifolium).

Arsène, G. 10632 a (elaeagnifolium).

Arteaga Saucedo, M.C. 840 (elaeagnifolium).

Articó, L. 241 (elaeagnifolium); 244 (elaeagnifolium); 250 (elaeagnifolium).

Artyushenko, Z.T. 238 (virginianum).

Arulappan, C. 67 (trilobatum).

Aryes, P.B. t 132 (violaceum).

Arzolla, F.A.R.D. 530 (robustum); 580 (torvum).

Ashall, C. DA 45 (cordatum); DA 45 (cordatum).

Ashe, C. 34 (jamaicense).

Asrana, R. RA 2887 (hovei); RA 3171 (hovei).

Aswal, B.S. NC-40178 (insanum).

Atwood, N.D. 11023 (elaeagnifolium); 16071 (elaeagnifolium); 28852 (elaeagnifolium).

Auglade, L. 2175 (violaceum).

Aulestia, C. 1080 (jamaicense).

Aung, M.M. 92183 (torvum); 92372 (viarum).

Austin, S.B. 277 (elaeagnifolium).

Avellaneda, M. 8 (jamaicense).

Avery, C.N. 779 A (wrightii).

Averyanov, L. AL-482 (robinsonii); VH 1090 (insanum); VH 2739 (violaceum).

Awando, E.S. B 9409 (arundo).

Aye, T.T. 20611 (melongena); 21276 (violaceum).

B S 1102 (trilobatum).

Babu, C.R. NC-34959 (chrysotrichum); NC-35284 (violaceum).

Bacigalupi, R. 2663 (elaeagnifolium).

Backer, C.A. 6910 (violaceum); 17065 (melongena); 36562 (involucratum); 37259 (involucratum).

Badami, P.S.K. 114 (cordatum).

Bagshawe, A.G. 1091 (giganteum); 1120 (aethiopicum).

Bai, B. 417 (deflexicarpum).

Baines, R. 255 (elaeagnifolium).

Baird, G.I. 1818 (elaeagnifolium).

Baker, M.S. 11244 (elaeagnifolium); 11574 (elaeagnifolium).

Bakhuizen van den Brink Jr, R.C. 3274 (involucratum); 4940 (involucratum); 7111 (involucratum).

Bakia, K. 468 (lasiocarpum).

Baladh, S. NC-67100 (violaceum).

Balaka, J.L. 777 (torvum).

Balakrishnan, N.P. ANC-150 (torvum); 614 (insanum); ANC-738 (torvum); ANC-985 (virginianum); ANC-1347 (torvum); ANC-1308 (torvum); ANC-1312 (violaceum).ANC-3589 (torvum); ANC-5503 (violaceum); ANC-5504 (violaceum); SC-10094 (elaeagnifolium); SC-11388 (insanum); 40288 (violaceum).

Balamani, B. BB 449 (hovei).

Balansa, B. 943 (procumbens); 945 (violaceum); 948 (insanum); 2096 (robustum); 3744 (procumbens); 4571 (violaceum).

Baldwin Jr, J.T. 12101 (aculeatissimum).

Baldwin, J.T. 9853 (wrightii).

Balegno, B. 249 (elaeagnifolium).

Balfour Gourlay, W. 270 (elaeagnifolium).

Balfour, I.B. 1007 (insanum); 1252 (violaceum); 1324 (melongena).

Balls, E.K. 5910 (sisymbriifolium); 10857 (elaeagnifolium).

Bally, J. B 3620 (aculeatissimum).

Bally, P.R.O. B 1034 (arundo); 2350 (aculeatissimum); B 3522 (giganteum); 3620 (aculeatissimum); B 3622 (giganteum); K 4127 (arundo); 4849 (aculeatissimum); B 7468 (giganteum); 7784 (aculeatissimum); 8296 (aculeatissimum); B 9409 (arundo); B 11693 (arundo); K 12496 (arundo).

Balsinhas, A. 3445 (sisymbriifolium).

Bamps, P. 55 (aculeatissimum).

Banarjee, L.K. 604 (insanum).

Banerjee, B.C. CNH-11328 (capsicoides); CNH-11516 (capsicoides).

Banerjee, R.N. 85 (trilobatum); 89 (trilobatum); CNH-18621 (torvum); CNH-18697 (torvum).

Banerjee, S.P. 64 (violaceum).

Bang, M. 614 (sisymbriifolium); 1608 (wrightii).

Bansjee, L.K. CNH-600 (virginianum).

Bara, T. 37 (dunalianum).

Barber, C.A. 132 (trilobatum); 2188 (multiflorum); 3203 (vagum); 4909 (trilobatum); 6490 (pubescens); 7114 (trilobatum); 8079 (insanum).

Barbon, E.B. PPI 1760 (torvum); 1976 (torvum); 2178 (jamaicense); 5935 (torvum); PPI 8208 (torvum); 8271 (torvum); 8590 (torvum); 18814 (torvum).

Barbour, M. 119 (elaeagnifolium).

Barboza, G.E. 76 (elaeagnifolium); 220 (elaeagnifolium); 379 (robustum); 382 (robustum); 394 (capsicoides); 483 (robustum); 819 (viarum); 826 (sisymbriifolium); 900 (aculeatissimum); 908 (aculeatissimum); 921 (viarum); 1006 (aculeatissimum); 1617 (aculeatissimum); 1623 (capsicoides); 1641 (capsicoides); 1923 (elaeagnifolium); 1944 (elaeagnifolium); 2010 (elaeagnifolium); 2038 (capsicoides); 2049 (viarum); 2051 (sisymbriifolium); 2079 (robustum); 2089 (viarum); 2276 (viarum); 2308 (elaeagnifolium); 2319 (elaeagnifolium); 2321 (elaeagnifolium); 2334 (elaeagnifolium); 2340 (elaeagnifolium); 3084 (elaeagnifolium); 3131 (elaeagnifolium); 3159 (elaeagnifolium); 3160 (elaeagnifolium); 3246 (elaeagnifolium); 3434 (elaeagnifolium); 3435 (elaeagnifolium); 3449 (elaeagnifolium); 4120 (elaeagnifolium); 4337 (elaeagnifolium); 4450 (elaeagnifolium); 4778 (elaeagnifolium); 5155 (elaeagnifolium).

Barclay, C. 1311 (robustum).

Barclay, G.W. 1132 (torvum); 1314 (capsicoides).

Barclay, H.G. 8419 (sisymbriifolium).

Barfod, A. 48017 (jamaicense).

Barker, J. 506 (macrocarpon).

Barkhadele, A.M.I. 280 (arundo).

Barkham, J. J 24 (arundo).

Barkley, F.A. A015 (sisymbriifolium); 18C 015 (sisymbriifolium); 38C 409 (jamaicense); 14A 539 (elaeagnifolium); 783 (elaeagnifolium); 17 C 996 (torvum); 18657 C (torvum); 35355 (jamaicense); 38495 (torvum).

Barnard, D.E. SoKUB 4 (virginianum).

Barnes, E. 92 (multiflorum); 281 (giganteum); 301 (torvum); 352 (multiflorum); 431 (torvum); 439 (multiflorum); 452 (multiflorum); 589 (multiflorum); 1062 (insanum).

Baro, D. 495 (elaeagnifolium).

Baron, R. 1584 (insanum); 4405 (insanum); 4405 (melongena); 4574 (melongena); 5001 (aethiopicum).

Barringer, K. 1827 (chrysotrichum); 2646 (jamaicense); 4063 (jamaicense).

Barringer, K.A. 1827 (chrysotrichum).

Barry, J.P. 62 (camranhense); 63 (camranhense).

Barthelat, F. 243 (torvum).

Bartholomew, B. 2430 (elaeagnifolium); 3955 (torvum); 3965 (viarum).

Bartlett, H.H. 437 (violaceum); 440 (violaceum); 8407 (violaceum); 10991 (elaeagnifolium); 14717 (torvum); 15140 (miyakojimense); 15144 (insanum); 15385 (miyakojimense); 19760 (elaeagnifolium); 19871 (elaeagnifolium); 19886 (elaeagnifolium); 19905 (elaeagnifolium).

Basu, P. ANC-6618 (torvum); ANC-6866 (viarum); ANC-6869 (lasiocarpum); CNH-13235 (melongena); CNH-13324 (melongena).

Basudde, I. 385 (aethiopicum).

Basunde, I. 892 (aculeatissimum).

Bateson, G. 146 (lasiocarpum).

Batianoff, G.N. 505100 (melongena).

Bauer, P.J. 58 (torvum); 185 (torvum).

Baum, H. 725 (aethiopicum).

Bayliss, R.D.A. 8220 (sisymbriifolium).

Beard, P. 1159 (torvum).

Beatley, J. 9028 (elaeagnifolium).

Beauvais, J. 20 (violaceum).

Beccari, N. 194 (melongena).

Beck, H.T. 75 (jamaicense).

Beck, S.G. 3812 (wrightii); 5159 (jamaicense).

Beddome, R.H. 5477 (torvum); 5478 (pubescens); 5489 (giganteum); 5490 (giganteum); 5493 (lasiocarpum); 5494 (wrightii); 5495 (multiflorum); 5496 (melongena); 5498 (insanum); 5499 (melongena); 5500 (violaceum); 5941 (lasiocarpum); 5942 (lasiocarpum).

Bedi, R. 225 (torvum).

Beentje, H.J. 413 (aculeatissimum).

Beer's collectors BSIP-7305 (lasiocarpum).

Beetle, A.A. 26132 (elaeagnifolium).

Béguin, D. 1037 (lasiocarpum).

Bélanger, C.P. 380 (hovei).

Belcher, C. 20 (torvum); 99 (virginianum).

Belcher, R.O. 390 (torvum); 561 (torvum); 748 (viarum); 848 (violaceum).

Belém, R.P. 1404 (robustum).

Bell, A.S. Mrs 40 (melongena); 119 -B (insanum); 863 (insanum).

Bell, W.M. 48 (elaeagnifolium).

Bello, M.A. 585 (torvum).

Bels, L. 60 (torvum).

Beltrán, G.D. 23 (capsicoides); 30 (mammosum); 35 (wrightii); 41 (torvum); 80 (viarum).

Benedeto, ? 235 (elaeagnifolium).

Benedetto, P. 137 (giganteum); 449 (capsicoides).

Benedictis, A. de 496 (forskalii).

Benítez de Rojas, C.E. 462 (jamaicense); 471 (jamaicense); 1031 (mammosum); 1045 (wrightii).

Benítez P, A. 1814 (elaeagnifolium).

Benke, H.C. 5209 (elaeagnifolium).

Benko-Iseppon, A.M. 419 (sisymbriifolium).

Bennet, H.R. 8659 (elaeagnifolium).

Bennet, S.S.R. 482 (trilobatum); 1064 (trilobatum).

Bennett, H.R. 8486 (elaeagnifolium).

Bequaert, J.C.C. 188 (torvum); 219 (giganteum); 1521 (giganteum); 2371 (macrocarpon).

Berendsohn, W.G. WB 36 (wrightii).

Berg, C.C. 153 (elaeagnifolium).

Bergen, M.A. van 99 (torvum).

Berhaut, R.P. 1376 (forskalii); 2326 (forskalii).

Berlandier, J.L. 665 (elaeagnifolium); 1726 (elaeagnifolium); 2075 (elaeagnifolium).

Bernacci, L.C. 2129 (sisymbriifolium); 33909 (sisymbriifolium).

Bernard, R. 1309 (torvum).

Bernardello, G.L. 449 (elaeagnifolium); 503 (elaeagnifolium); 522 (elaeagnifolium); 534 (elaeagnifolium); 746 (elaeagnifolium); 754 (elaeagnifolium); 788 (elaeagnifolium).

Bernardi, A.L. 18126 (sisymbriifolium); 18420 (sisymbriifolium); 20378 (sisymbriifolium); 20502 (sisymbriifolium).

Bernardi, L. 8244 (torvum); 11864 (torvum); 14298 (trilobatum); 15327 (trilobatum); 16802 (wrightii); 18126 (sisymbriifolium); 18278 (sisymbriifolium); 18489 (robustum); 18595 (sisymbriifolium); 18883 (robustum).

Bernardi, M. 18883 (robustum).

Bernoulli, K.G. 2345 (mammosum); 2368 (chrysotrichum).

Bernstein, J.H. 133 (lasiocarpum).

Berro, M.B. 4411 (elaeagnifolium); 7942 (elaeagnifolium).

Best, E.B. 143 (chrysotrichum).

Betancourt A, C. 7 (mammosum); 21 (jamaicense).

Betancur, J.C. 1401 (jamaicense); 15164 (jamaicense); 18609 (jamaicense).

Beusekom, C.F. van 3772 (lasiocarpum).

Beuson, J.M. 273 (torvum).

Bhargava, N. ANC-1711 (lasiocarpum); ANC-1930 (violaceum); ANC-1930 (violaceum); ANC-2309 (torvum); ANC-2456 (viarum); ANC-3325 (torvum); ANC-4103 (torvum); ANC-5639 (melongena); ANC-5895 (lasiocarpum); ANC-6302 (lasiocarpum); ANC-6318 (torvum); ANC-6539 (torvum); NC-51506 (insanum); NC-63716 (chrysotrichum).

Bhargavan, N.P. ANC-1784 (torvum).

Bhargavan, P. 90001 (multiflorum).

Bhattacharyya, U.C. NC-13625 (insanum); 15236 (insanum); NC-17688 (insanum); NC-37674 (violaceum).

Bhide, R.K. 877 (hovei).

Bianor 962 (melongena).

Bidgood, S.; Keeley, P.A. 317 (aculeatissimum).

Biegel, H.M. 2429 (aculeatissimum); 3463 (wrightii).

Bigger, M. 1179 (aculeatissimum).

Bihar, ? 473 (insanum).

Bililong, M. 389 (torvum).

Billiet, F. 3329 (sisymbriifolium).

Billore, K.V. 110573 (hovei); 110948 (hovei); 111709 (hovei); 111928 (hovei); 112933 (hovei); 113081 (hovei); 113582 (hovei); 115473 (hovei); 115538 (hovei); 116745 (hovei).

Biloni, J.S. 175 M (elaeagnifolium).

Biltmore Herbarium 3438 (elaeagnifolium).

Binh, B.T. 746 (torvum).

Binuyo, A. FHI 35550 (torvum); FHI 41356 (torvum).

Biolley, P. 7041 (torvum).

Birch, ? 59 139 (arundo).

Bis Ram 229 (violaceum); 274 (violaceum).

Bisschoff, J. 1 (elaeagnifolium).

Biswas, K. 559 (barbisetum); 1380 (mammosum); 3731 (sisymbriifolium); 5756 (torvum); 5784 (viarum); 6550 (viarum); 6781 (torvum).

Biswas, S. 560 (lasiocarpum); 893 (lasiocarpum).

Bittencourt, F. 114 (torvum); 223 (torvum).

Bittner, J. 1698 (torvum).

Biurrun, F. 2103 (elaeagnifolium); 2729 (elaeagnifolium); 7225 (elaeagnifolium).

Biye, E. 35 (aethiopicum); 58 (melongena); 63 (aethiopicum).

Blackmore, S. 978 (aculeatissimum); 1417 (chrysotrichum); 1703 (torvum).

Blanchet, J.S. 81 (capsicoides); 236 (melongena); 368 (melongena); 947 (capsicoides); 6410 (elaeagnifolium).

Blasco, F. 45 (violaceum); 2089 (trilobatum); 2113 (insanum); 2573 (pubescens).

Blatter, E. 89 (multiflorum); 2115 (hovei).

Blicher-Mathieson, U. 62551 (jamaicense).

Bloembergen, S. 3439 (lasiocarpum).

Blume, E. 399 (sisymbriifolium).

Blumer, J.C. 1732 (elaeagnifolium).

Boabaid, L.F. 010 (sisymbriifolium).

Boddingius, J. 17 (torvum).

Bodenbender, W. 55 (elaeagnifolium).

Bodinier, E.M. 1098 (lasiocarpum).

Boege, W. 2793 (elaeagnifolium).

Boelcke, O. 1412 (elaeagnifolium); 5070 (sisymbriifolium); 11691 (elaeagnifolium).

Bogdan, A. 452 (aculeatissimum); 4271 (aculeatissimum).

Bohs, L. 1525 (jamaicense); 1801 (chrysotrichum); 3307 (sisymbriifolium); 3519 (chrysotrichum); 3603 (torvum); 3934 (chrysotrichum); 3965 (torvum).

Bois, D.G.J.M. 93 (violaceum); 226 (procumbens); 475 (torvum); 531 (violaceum).

Boivin, B. 13795 (elaeagnifolium).

Bole, P.V. 1628 (hovei); 2388 (hovei).

Bolinger, A. 10 (arundo).

Bolt, ? 78 (forskalii).

Bombay Natural History Society 2016 (pubescens).

Bon, R.P. 96 (violaceum); 120 (procumbens); 196 (insanum); 350 (procumbens); 506 (lasiocarpum); 598 (procumbens); 642 (procumbens); 680 (lasiocarpum); 1741 (insanum); 1742 (lasiocarpum); 2193 (procumbens); 3873 (praetermissum); 4017 (violaceum); 4442 (lasiocarpum); 5071 (insanum).

Bonnin, L. 408 (torvum).

Bono, G. 101 (aculeatissimum).

Boom, B.M. 2516 (jamaicense); 2516 (jamaicense); 7195 (jamaicense); 8055 (jamaicense).

Bor, N.L. 18478 (barbisetum).

Borden, T.E. 1948 (insanum); 2336 (torvoideum).

Borgmann, E. 322 (torvoideum).

Borsini, O. 1013 (elaeagnifolium).

Borsini, O.H. 1201 (elaeagnifolium); 1333 (elaeagnifolium).

Borssum Waalkes, J. van 2695 (lasiocarpum).

Bortoletto, C.A. 3 (sisymbriifolium).

Bos, J.J. 1047 (aculeatissimum); 1927 (torvum); 1967 (torvum); 2031 (torvum); 2385 (torvum); 2633 (torvum); 3262 (torvum).

Bosser, J.M. 5632 (torvum); 11510 (torvum).

Botanical Expedition to Himalaya 1990 90-60015 (violaceum).

Botanical Expedition to Far West Nepal 1991 91-94137 (violaceum).

Botany students Yrs 2 & 3 98 (robustum).

Botello, L. 14 (elaeagnifolium).

Botter, K.R. 24226 (aculeatissimum).

Botteri, M. 854 (chrysotrichum).

Bouamanivong, S. 6 (torvum).

Boughey, A.S. 107 (torvum).

Boulos, L. 13826 (forskalii); 16689 (forskalii); 16711 (cordatum); 16992 (pubescens); 18198 (elaeagnifolium).

Bourell, M. 2372 (torvum); 2390 (torvum); 2622 (sisymbriifolium).

Bourne, A.G. 456 (giganteum); 1643 (violaceum); 2809 (giganteum); 2810 (violaceum); 3308 (virginianum); 5055 (pubescens); 5146 (pubescens); 6218 (torvum); 6293 (torvum).

Bourne, E.T. 3168 (trilobatum).

Bourobou, H.P. 255 (torvum).

Bouton, M. 5 (violaceum).

Bowen, J. 27 (elaeagnifolium).

Bowes-Lyon, S.A. 6010 (torvum).

Bowie, C.B.J. 1 (aculeatissimum); 131 (giganteum); 160 (sisymbriifolium).

Box, H.E. 960 (capsicoides); 984 (torvum); 1567 (capsicoides).

Bradtke, W. 62 (dunalianum); 184 (dunalianum).

Bragg, K. 81 (torvum); 85 (melongena).

Brande, J. van den 313 (giganteum).

Brant, A.E. 1204 (jamaicense).

Brass, L.J. 3815 (dunalianum); 5411 (torvoideum); 7467 (lasiocarpum); 8514 (dunalianum); 11590 (torvoideum); 22030 (lasiocarpum); 23950 (schefferi); 23962 (lasiocarpum); 24330 (schefferi); 27452 (dunalianum); 27637 (schefferi); 28146 (lasiocarpum); 28809 (lasiocarpum).

Braun, K. 995 (sisymbriifolium).

Braun, K.P. 1824 (sisymbriifolium).

Braun, K.P.J.G. 2217 (robustum); 2843 (aculeatissimum).

Braunton, E. 697 (elaeagnifolium).

Brédo, H.J. 1455 (giganteum).

Breedlove, D.E. 1150 (elaeagnifolium); 6595 (torvum); 11389 (torvum); 13150 (torvum).

Brenan, J.P.M. 8443 (torvum); 14811 (forskalii).

Breteler, F.J. 183 (torvum); 5730 (torvum); 6362 (torvum); 7081 (torvum); 8229 (torvum); 8667 (torvum); 8802 (aethiopicum); 9683 (torvum); 9973 (torvum); 15336 (torvum).

Bridges, T. 399 (elaeagnifolium).

Bridson, D.M. 28 (aculeatissimum).

Brigada Zacatecas 86 (elaeagnifolium).

Brink, E. 276 (chrysotrichum); 294 (giganteum).

Bristol, M.L. 1455 (sisymbriifolium).

Britton, B.B. 396 (lasiocarpum).

Britton, M.E. 62 (dunalianum).

Brizuela, J. 847 (elaeagnifolium); 1159 (elaeagnifolium).

Brother Anect 115 (elaeagnifolium).

Broadway, W.E. 270 (torvum); 583 (mammosum); 858 (jamaicense); 4594 (torvum).

Brodhurst-Hill, E. 381 (aculeatissimum).

Broome, C.R. 313 (elaeagnifolium).

Brown, E.S. 876 c (torvum).

Brown, F. 52 (cordatum).

Bruce, E.M. 188 (giganteum); 527 (robustum).

Brummitt, R.K. 8590 (aculeatissimum); 9391 (giganteum); 11543 (chrysotrichum); 15093 A (aculeatissimum); 15241 (wrightii).

Brunner, D. 1942 (robustum).

Bruno, R. 4 (elaeagnifolium).

Brunt, M. 2235 (torvum).

Brunt, M.A. 287 (torvum).

Buch, ? 61 116 (arundo).

Buchanan, J. 65 (aculeatissimum); 110 (aethiopicum); 140 (aethiopicum); 155 (aethiopicum).

Buchanan-Hamilton, F. 570 -2 (insanum); 570 -1 (insanum); 571 (insanum); 573 (violaceum).

Buchtien, O. 1431 (sisymbriifolium).

Buesekom, C.F. van 3772 (lasiocarpum).

Bufo, L.V.B. 141 (robustum).

Buller, R.H. REP-19845 (cordatum).

Bullock, ? 13816 (procumbens).

Bullock, A.A. 646 (torvum).

Bullock, W. 3 (sisymbriifolium).

Bulmer, R.N.H. 139 (torvoideum).

Bult, M. van de 174 (barbisetum).

Bunnemeyer, H.A.B. 2786 (poka); 4882 (lasiocarpum); 7261 (lasiocarpum); 7975 (torvoideum); 11592 (involucratum); 11732 (poka); 11813 A (poka); 11980 (lasiocarpum); 12117 (poka).

Bunpheng, D. 224 (violaceum).

Burch, D. 2302 (torvum); 2334 (torvum); 3444 (mammosum).

Burchell, W.J. A-739 (robustum); 3239 (aculeatissimum); 4495 (giganteum); 4739 (robustum).

Burge, A. 25 (elaeagnifolium).

Burger, W.C. 3652 (aculeatissimum); 7959 (chrysotrichum).

Burhoni, ? 27 (robustum).

Burkart, A. 3364 (elaeagnifolium); 15989 (elaeagnifolium); 21399 (elaeagnifolium); 23491 (elaeagnifolium); 24918 (elaeagnifolium); 24927 (elaeagnifolium).

Burkill, H.M. 2253 (torvum); 2854 (torvum).

Burkill, I.H. 432 (elaeagnifolium); 33280 (hovei); 36313 (violaceum).

Burley, J.S. 123 (lasiocarpum); 1999 (jamaicense); 3625 (sulawesi).

Burmann, J. 41 (virginianum).

Burnemeyer, ? 12117 (pseudosaponaceum).

Burnham, R. 35 (torvum).

Burtt, B.D. 3665 (arundo); 4276 (arundo); 5258 (arundo).

Burtt, B.L. 710 (violaceum); B-1084 (cordatum).

Bush, B.F. 266 (elaeagnifolium); 392 (elaeagnifolium); 1588 (elaeagnifolium).

Busse, W. 3097 (giganteum).

Busyman, M. 92 (torvum).

Butterwick, M. 7782 (elaeagnifolium); 8217 (elaeagnifolium).

Büttner, O.A.R. 341 (aethiopicum).

Buwalda, P. 4147 (schefferi); 4309 (lasiocarpum); 4633 (lasiocarpum).

Buysman, M. 147 (involucratum).

Buzato, S. 20693 (robustum).

Bye, R.A. 28334 (elaeagnifolium).

Bytebier, B. 65 (aculeatissimum).

Cabezas, F. 111 (torvum); 209 (torvum).

Cable, S. 322 (torvum); 907 (torvum); 1526 (torvum); 2552 (torvum); 2780 (torvum).

Cabrera R, I. 3055 (jamaicense).

Cabrera, A.L. 7007 (elaeagnifolium); 30038 (elaeagnifolium); 30771 (elaeagnifolium); 31065 (elaeagnifolium).

Cabrera, I. 3536 (jamaicense).

Cadena, B. 23 (elaeagnifolium).

Cadet, T. 1493 (robustum); 2386 (violaceum); 4684 (torvum).

Cafferty, S. 66 (jamaicense).

Calderón, S. 16 (torvum); 2494 (torvum).

Calhoun, D. 129 (elaeagnifolium).

Callejas, R. 736 (sisymbriifolium); 3436 (jamaicense); 4970 (jamaicense).

Calléry, J.M.M. 28 29 (torvum); 812 (violaceum).

Calónico-Soto, J. 21238 (torvum).

Calzada, J.I. 2020 (chrysotrichum); 24391 (elaeagnifolium); 25305 (elaeagnifolium).

Camargo, A. 66 (jamaicense).

Camilo, H.A. 65 (jamaicense).

Campbell, J. 79 (violaceum).

Campo, J.M. 2170 (jamaicense).

Campos V, A. 2333 (capsicoides).

Campos, J. 2229 (capsicoides); 2391 (sisymbriifolium); 5424 (sisymbriifolium).

Campos, M.T.V.A. CFSC 13326 (viarum).

Canedo, F. 9075 (elaeagnifolium).

Cano R, P. 21 (elaeagnifolium).

Cano, A. 867 (chrysotrichum).

Cano, C. 185 (torvum).

Cantero, J.J. 6183 (elaeagnifolium).

Cantino, P.D. 505 (elaeagnifolium).

Carauta, J.P. 3172 (torvum).

Carbonó, E. 363 (torvum); 468 (jamaicense).

Cárdenas, M. 7614 (sisymbriifolium).

Cardoso, D. 640 (robustum).

Carette, E. 22 (elaeagnifolium).

Carles, W.R. 157 (insanum).

Carleton, M.A. 171 (elaeagnifolium); 560 (torvum).

Carnegie, F.G. 40 (elaeagnifolium).

Carnevali, G. 6111 (torvum).

Caro R, A.M. 1416 (jamaicense); 2191 (jamaicense).

Carpio, A. 16 (wrightii).

Carr, C.E. 12747 (lasiocarpum); 12748 (torvoideum); 13198 (torvoideum); 15417 (torvoideum); 15613 (lasiocarpum).

Carretero, A. 1002 (sisymbriifolium).

Carrick, J. 1463 (torvum).

Carrington, J.F. 66 (torvum).

Carter, G.F. 26 (sisymbriifolium).

Carter, H.G. EB-301 (melongena).

Carter, S. 2004 (forskalii).

Carvalheira, R.P.C. JPB 3532 (capsicoides).

Carvalho, A.M. de 6683 (torvum).

Carvalho, F.A.F. 6683 (torvum).

Carvalho, M.F. do 2022 (torvum).

Cascante, A. 759 (chrysotrichum).

Case, P. 154 (wrightii).

Castañeda Vega, ? 64 (elaeagnifolium); 68 (elaeagnifolium).

Castellanos, A. 90 (elaeagnifolium); 389 (elaeagnifolium); 806 (elaeagnifolium).

Castello, L.V. 232 (elaeagnifolium).

Castillón, L. 1779 (elaeagnifolium).

Castle-Smith, P.M. 80 (torvum).

Castro, D. 2634 (wrightii).

Castro, E. 53 (torvum); 137 (torvum).

Castro, J. 1 (mammosum).

Castro, P. 1360 (torvum); 1504 (cyanocarphium).

Castro, R.M. 1234 (robustum).

Castro, S. 118 (jamaicense); 348 (jamaicense); 349 (jamaicense); 593 (jamaicense).

Castroviejo, S. 9110 (torvum).

Cavalerie, P.J. 2722 (aculeatissimum); 4092 (violaceum); 4344 (violaceum).

Caxambú, M.G. 1906 (sisymbriifolium).

Cerón, C.E. 15487 (sisymbriifolium); 15774 (sisymbriifolium); 16047 (sisymbriifolium); 20851 (mammosum); 24424 (sisymbriifolium); 42944 (sisymbriifolium).

Cerribile, M. 146 (sisymbriifolium).

Cervantes M, H. 58 (elaeagnifolium).

Cervi, A.C. 2897 (sisymbriifolium).

Chakraborty, G. ANC-12336 (lasiocarpum).

Chakraborty, P. ANC-1232 (insanum); BSHC-1941 (virginianum); ANC-2142 (insanum).

Chan, C. 599 (torvum); 3018 (torvum); 3596 (torvum); 4699 (torvum).

Chandler, P. 203 (mammosum).

Chandra, S. 5 (trilobatum).

Chandrabose, M. SC-28500 (insanum); SC-28526 (insanum); 28533 (trilobatum); SC-28977 (elaeagnifolium); 30514 (trilobatum); 30631 (trilobatum); 31106 (trilobatum); 51683 (pubescens).

Chang, C.C. 99063 (miyakojimense).

Chang, C.E. 9059 (miyakojimense).

Chantaranothai, P. 90 185 (lasiocarpum); 90 448 (barbisetum); 90 449 (involucratum); 1161 (torvum).

Chapama, H.T. 429 (aculeatissimum).

Chapman, G.D. 1373 (chrysotrichum).

Chapman, J.D. 2832 (aculeatissimum); 4004 (giganteum); 5161 (torvum); 8878 (giganteum).

Charlton, D. 3259 (elaeagnifolium).

Charnpareet 9430 (insanum).

Charpin, A. 20190 (robustum).

Chase, N.C. 5164 (giganteum); 7445 (giganteum).

Chassagne, F.C. FC 138 (cyanocarphium).

Chatterjee, A.C. 3 (melongena); 38 (melongena); 48 (insanum); 273 (melongena).

Chaudhary, S.A. E 455 (elaeagnifolium).

Chavarría, U. 339 (torvum); 761 (torvum).

Cheek, M.R. 7059 (torvum); 7903 (torvum); 17838 (viarum).

Cheng Shaoqin 13537 (praetermissum); 17170 (praetermissum).

Cheng, K.C. CL 929 (cyanocarphium).

Cheng, S.K. CL-1122 (torvum); CL-1186 (lasiocarpum); CL-1367 (trilobatum).

Cheo, H.C. 96 (melongena).

Cheo, T.Y. 109 (melongena).

Cherian, P.J. 106290 (hovei); 109394 (hovei); 111106 (hovei); 111397 (hovei); 111574 (hovei); 112767 (hovei).

Chevalier, A.J.B. 589 (aethiopicum); 5180 (aethiopicum); 5198 (giganteum); 5198 (giganteum); 29028 (violaceum); 29170 (procumbens); 29547 (procumbens); 29563 (violaceum); 30724 (violaceum); 38932 (camranhense); 39251 (involucratum); 39391 (violaceum); 39417 (violaceum); 39496 (procumbens); 39497 (procumbens); 40321 (violaceum); 40449 (insanum); 40551 (violaceum); 40562 (nienkui).

Chew, W.L. 1667 (lasiocarpum); 7087 (lasiocarpum).

Chiao, C.Y. 2972 (melongena); 14490 (melongena).

Chiapella, J. 1804 (elaeagnifolium); 2175 (elaeagnifolium); 2186 (elaeagnifolium).

Chiarini, F. 27 (sisymbriifolium); 40 (elaeagnifolium); 462 (elaeagnifolium); 507 (elaeagnifolium); 533 (viarum); 537 (viarum); 558 (elaeagnifolium); 559 (elaeagnifolium); 565 (elaeagnifolium); 566 (elaeagnifolium); 644 (viarum); 736 (elaeagnifolium); 781

(elaeagnifolium); 824 (elaeagnifolium); 828 (elaeagnifolium); 838 (viarum); 879 (elaeagnifolium); 884 (sisymbriifolium); 890 (elaeagnifolium); 892 (elaeagnifolium); 899 (elaeagnifolium); 917 (elaeagnifolium); 928 (elaeagnifolium); 1026 (elaeagnifolium); 1028 (elaeagnifolium); 1030 (elaeagnifolium); 1033 (elaeagnifolium); 1034 (elaeagnifolium); 1039 (elaeagnifolium); 1270 (elaeagnifolium); 1346 (viarum); 1384 (elaeagnifolium); 1394 (elaeagnifolium); 1403 (elaeagnifolium); 1466 (elaeagnifolium).

Chibes, J.E. 7 (melongena).

Child, H. 7 (torvum).

Chin, S.C. 2553 (lasiocarpum); 3559 (lasiocarpum).

Chinchilla, M. 126 (torvum); 130 (jamaicense).

Chinese collector 4500 (procumbens).

Ching, R.C. 5271 (violaceum); 7640 (pseudosaponaceum).

Chiovenda, E. 1072 (giganteum); 1202 (giganteum).

Chiu, S.F. H-4 (violaceum).

Chongko, S. 114 (lasiocarpum).

Chorian, J. WC-106704 (insanum).

Chorley, M. 3 (insanum); 163 (torvum); 288 (jamaicense).

Chow, H.-.C. 1335 (melongena).

Chow, K.S. 78074 (torvum); 78313 (nienkui); 78465 (capsicoides); 78889 (nienkui); 78904 (nienkui).

Chowdhery, H.J. NC-75822 (violaceum); NC-75825 (violaceum).

Chowdhury, A.M. 13 (cordatum).

Christ, J. 991 (elaeagnifolium).

Christenhusz, M.J.M. 5361 (chrysotrichum); 5460 (torvum); 5554 (torvum).

Chuang, T.I. 2420 (miyakojimense).

Chun, N.K. 40175 (lasiocarpum); 43319 (nienkui); 43473 (insanum); 44522 (nienkui); 44631 (procumbens).

Chun, S.H. 11876 (praetermissum); 13537 (praetermissum).

Chun, W.Y. 6713 (melongena).

Chun, Z.C. 50421 (pseudosaponaceum).

Chung, H.H. 7532 (violaceum).

Cid Ferreira, C.A. 1903 (mammosum); 3823 (jamaicense).

Cid, F. 81 (lasiocarpum).

Cirujano, S. R-10431 (elaeagnifolium).

Claessens, J. 621 (giganteum).

Claraz Schenkung, A. 81 (elaeagnifolium).

Clark, J.L. 9611 (jamaicense).

Clarke, C.B. 37 (insanum); 4626 (lasiocarpum); 4864 A (barbisetum); 6611 (lasiocarpum); 6681 (lasiocarpum); 6694 (violaceum); 6695 (torvum); 6902 (melongena); 9057 (barbisetum); 10728 A (giganteum); 10772 (wrightii); 10793 (wightii); 11701 A (virginianum); 11701 B (virginianum); 11764 (violaceum); 11772 (barbisetum); 11923 (barbisetum); 11935 A (barbisetum); 17369 (lasiocarpum); 26478 B (violaceum); 37071 e (trilobatum); 37071 F (trilobatum); 37071 D (trilobatum); 37071 A (trilobatum); 37414 (aculeatissimum); 38107 C (barbisetum); 38359 A (aculeatissimum); 38418 B (sisymbriifolium); 38707 (barbisetum); 44089 B (aculeatissimum).

Clausen, J. 595 (elaeagnifolium).

Clausen, R.T. 4597 (elaeagnifolium).

Claussen, P. 204 (sisymbriifolium); 16851 (robustum).

Clayton, J.W. 4 (torvum).

Clemens, J. 3311 (insanum); 3422 (torvum); 3453 (procumbens); 4004 (procumbens); 21518 (lasiocarpum).

Clemens, M.S. 1699 (schefferi); 17487 (torvoideum); 41609 (lasiocarpum); 51887 (retrorsum).

Clement, E.J. TB93 97 (torvum).

Clokey, I.W. 5937 (elaeagnifolium).

Clover, E.U. 165 (elaeagnifolium).

Clunie, N.M.U. LAE-63523 (lasiocarpum).

Clute, W.N. 32 (torvum).

Cochrane, T.S. 8630 (chrysotrichum).

Cocucci, A.A. 14 (elaeagnifolium); 220 (elaeagnifolium); 369 (elaeagnifolium); 439 (elaeagnifolium); 440 (elaeagnifolium); 977 (elaeagnifolium); 1011 (elaeagnifolium); 1014 (elaeagnifolium); 1364 (elaeagnifolium); 3848 (elaeagnifolium); 5014 (elaeagnifolium); 5041 (elaeagnifolium); 5760 (elaeagnifolium).

Codd, L.E. 2548 (sisymbriifolium).

Coêlho, D.F. 663 (jamaicense).

Coêlho, L.S. 63 (sisymbriifolium).

Cole, ? 35 (multiflorum).

Collard, D.J. 32 (insanum); 56 (insanum).

Collazos, H. 175 (jamaicense); 174 288 (jamaicense).

Collenette, I.S. 246 (forskalii); 939 (forskalii); 1537 (forskalii); 3731 (virginianum); 5503 (cordatum); 5790 (cordatum); 5986 (cordatum).

Collett, H. 312 (virginianum).

Collins, D.J. 66 (torvum); 904 (involucratum); 1380 (trilobatum).

Comanor, P.L. 781 (insanum); 785 (trilobatum); 922 (torvum); 1082 (torvum).

Combs, R. 185 (torvum); 190 (jamaicense); 209 (mammosum); 665 (capsicoides).

Comins, R.B. 250 (schefferi).

Compère, P. 222 (torvum).

Compling, M. 9 (elaeagnifolium).

Compton, R.H. 17 (torvum).

Conceição, S.F. 952 (robustum).

Condon, W. 1 (torvum).

Conklin, H.C. 217 (pseudosaponaceum); I-935 (melongena); 2563 (melongena); 17576 (lasiocarpum); 18710 (lasiocarpum); 18780 (melongena); 19187 (melongena); 19367 (melongena); 19401 (melongena).

Conn, B.J. 87 (lasiocarpum); 1672 (torvoideum); 5072 (torvum).

Conrad, J. 2107 (capsicoides); 2206 (sisymbriifolium).

Contreras, E. 2878 (jamaicense); 5690 (jamaicense); 6470 (jamaicense); 9664 (jamaicense); 11120 (jamaicense); 11181 (jamaicense).

Coode, M.J.E. 5457 (lasiocarpum); NGF-29876 (lasiocarpum); NGF-32569 (dunalianum).

Cooke, B.K. 127 (forskalii).

Cooper, J.J. 5870 (chrysotrichum).

Cooper, R.E. 2435 (insanum); 3174 (violaceum).

Cooper, T. 1026 (aculeatissimum); 1126 (aculeatissimum); 1147 (aculeatissimum).

Cooray, R.G. 68-102305 R (vagum).

Cordeiro, J. 796 (sisymbriifolium); 1558 (torvum); 3108 (elaeagnifolium); 5472 (robustum).

Cordeiro, M. 631 (jamaicense).

Cordero P, Z. 546 (torvum).

Cordero, G. 236 (torvum).

Córdoba, C. 43 (jamaicense).

Córdoba, W.A. 151 (jamaicense); 231 (jamaicense); 478 (jamaicense).

Cornejo V, F. 1137 (wrightii).

Corner, E.J.H. SFN. 37775 (melongena); SF-37775 [a] (melongena); 38070 (violaceum).

Coronado G, I. 802 (wrightii).

Coronado, I. 802 (wrightii).

Correa M, M. 3 (melongena).

Correa, A.L. 17 (wrightii).

Correll, D.S. 47007 (torvum).

Cortés Castillo, D. 236 (jamaicense).

Cory, V.L. 52468 (elaeagnifolium).

Cosa, M.T. 44 (elaeagnifolium); 108 (elaeagnifolium); 115 (elaeagnifolium); 124 (elaeagnifolium); 162 (elaeagnifolium); 182 (elaeagnifolium); 186 (elaeagnifolium).

Costich, D.E. 1012 (viarum); 1013 (viarum).

Coulter, T. 1246 (elaeagnifolium); 1247 (elaeagnifolium); 1248 (elaeagnifolium).

Covile, D.P.M. 0237 (torvum); 2318 (capsicoides); 3669 (insanum); 4449 (capsicoides); 5444 (violaceum); 6199 (insanum).

Cowan, J.M. 114 (violaceum); 1018 (violaceum).

Cowper, S.G. 6 (torvum).

Cox, ? 532 (torvum).

Cramer, L.H. 3872 (violaceum); 4430 (torvum).

Crampton, B. 7136 (elaeagnifolium).

Craven, L.A. 89 (torvoideum).

Crawford, S. 1234 (torvum).

Criollo, G. 2101 (mammosum).

Croat, T.B. 74 (torvum); 11987 (jamaicense); 17476 (jamaicense); 17907 (wrightii); 18138 (jamaicense); 18291 (jamaicense); 19083 A (jamaicense); 19129 (jamaicense); 21723 (jamaicense); 22619 (torvum); 32379 (torvum); 32863 (chrysotrichum); 35499 (chrysotrichum); 36141 (chrysotrichum); 41941 (torvum); 42731 (jamaicense); 51138 (capsicoides); 52266 (jamaicense); 52975 (torvum); 62350 (sisymbriifolium); 68526 (torvum); 68531 (jamaicense); 68541 (capsicoides); 68579 (torvum); 68590 (capsicoides); 74871 (chrysotrichum); 78523 (torvum); 84056 (jamaicense); 99850 (jamaicense).

Crookshank, H. 549 (virginianum).

Crosby, M.R. 2860 (torvum); 2908 (torvum).

Crosswhite, F.S. 720 (elaeagnifolium).

Cruz, J.M. 21 (torvum); 97 (capsicoides).

Cuadra, A. A 1010 (cyanocarphium); A 1169 (lasiocarpum); A 1171 (melongena); A-2160 (lasiocarpum).

Cuadrado, ? 14 (sisymbriifolium).

Cuatrecasas, J. 1991 (mammosum); 3501 (jamaicense); 3565 (jamaicense); 3594 (jamaicense); 15255 (jamaicense); 15887 (jamaicense); 16959 (jamaicense); 21387 (jamaicense).

Cubitt, G.E.S. 580 (melongena); 581 (lasiocarpum).

Cuesta, L.R. 57 (elaeagnifolium).

Cueva, M. 636 (viarum).

Cuezzo, A.R. 872 (elaeagnifolium); 898 (elaeagnifolium); 1058 (elaeagnifolium); 1302 (elaeagnifolium); 2227 (elaeagnifolium); 2254 (elaeagnifolium).

Cui Jinyun 14666 (praetermissum).

Cuming, H. 169 (elaeagnifolium); 443 (insanum); 690 (lasiocarpum); 1090 (elaeagnifolium); 1529 (lasiocarpum); 2261 (violaceum); 2342 (trilobatum); 2422 (torvum).

Cuong, N.M. 48 (torvum); NMC 1439 (violaceum); NMC 1440 (procumbens).

Curran, H.M. 4874 (retrorsum); 4985 (torvoideum).

Curtis, C. 9 (torvum); 858 (trilobatum).

Curtiss, A.H. 106 (torvum); 258 (jamaicense); 2201 (sisymbriifolium); 2202 (capsicoides); 5913 (elaeagnifolium).

D'Arcy, W.G. 44 A (elaeagnifolium); 1709 (jamaicense); 1738 (capsicoides); 1974 (elaeagnifolium); 2584 (capsicoides); 2598 (capsicoides); 4719 (torvum); 4728 (torvum); 4861 (elaeagnifolium); 5000 (torvum); 8857 (aculeatissimum); 11689 (elaeagnifolium); 12027 (elaeagnifolium); 12131 (wrightii); 12132 (wrightii); 13380 (capsicoides); 15236 (violaceum); 15250 (torvum); 15257 (torvum); 15303 (aethiopicum); 15318 (insanum); 15333 (insanum); 15343 (insanum); 15386 (violaceum); 15488 (insanum); 15773 (sisymbriifolium); 16702 (melongena); 17676 (torvum); 17696 (insanum); 17699 (insanum); 17700 (insanum); 17701 (insanum); 17704 (melongena); 17738 (sisymbriifolium); 17863 (chrysotrichum).

D'Orbigny, A. 223 (elaeagnifolium).

D´Angelo, C. 285 (elaeagnifolium).

Dahlström, E. 467 (insanum).

Dakshum, K.M.M. NC-5554 (violaceum).

Dale, I.R. K 3272 (giganteum).

Daliph, N.E. 77 (cordatum).

Dalton, G. 6 (arundo).

Daly, D.C. 31 (sisymbriifolium); 44 (sisymbriifolium).

Dalzell, N.A. 603 (insanum).

Dalziel, J.M. (insanum); 1189 (macrocarpon); 1190 (torvum).

Damarzco, L. 1645 (robustum).

Damas, D. SAJ-1188 (torvum).

Daniel 2068 (jamaicense).

Daniel, P. NC-67592 (insanum).

Daniel, P.M. 31 (torvum).

Daramola, B.D. 96 (torvum).

Daramola, B.O. FHI 62453 (giganteum).

Darnton, S. 72 (torvum).

Darwin, S.P. 2519 (torvum).

Das, N.Y. NYD 190 (hovei); NYD 191 (hovei); NYD 709 (hovei); NYD 710 (hovei); NYD 1491 (hovei); NYD 2040 (hovei); NYD 5102 (hovei).

Das, R.K. 388 (praetermissum).

Datsum, K.M.M. NC-5509 (chrysotrichum).

Daveau, J. 2260 (elaeagnifolium).

Davidse, G. 3987 (torvum); 5461 (jamaicense); 5559 (sisymbriifolium); 5652 (jamaicense); 5711 (jamaicense); 6814 (giganteum); 7257 (insanum); 7374 (torvum); 9252 (elaeagnifolium);

23157 (chrysotrichum); 24087 (chrysotrichum); 24921 (chrysotrichum); 25554 (chrysotrichum); 26191 (chrysotrichum); 30743 (jamaicense); 34455 (jamaicense); 34590 (chrysotrichum); 37113 (torvum); 37140 (capsicoides).

Davidson, C. 7231 (chrysotrichum).

Davis, T. 677 (elaeagnifolium); 1000 (elaeagnifolium).

Dawe, M.T. 763 (aculeatissimum).

Dawson, G. 541 (viarum).

Deanna, R. 150 (sisymbriifolium); 185 (sisymbriifolium); 396 (elaeagnifolium).

Deaver, C.F. 6439 (elaeagnifolium).

Deb, D.B. 212 (insanum); 262 (viarum); 307 (violaceum); 1807 (melongena); CNH-3470 (insanum); BG-3471 (insanum); BG-3472 (insanum); BG-3474 (insanum); BG-3475 (insanum); BG-3476 (insanum); BG-3477 (insanum); BG-3478 (insanum); BG-3480 (insanum); BG-3481 (insanum); BG-3482 (insanum); BG-3483 (insanum); BG-3484 (insanum); BG-3485 (insanum); BG-3487 (insanum); BG-3489 (insanum); BG-3490 (insanum); 3491 (melongena); EC-26919 (melongena); 30146 (giganteum); SC-30163 (capsicoides); 30314 (multiflorum); 30725 (multiflorum).

Debbarman, P.M. 86 (insanum).

Debeaux, G. 130 (torvum); 258 (trilobatum).

Debeaux, J.O. 355 (violaceum).

Decary, R. 6308 (macrocarpon); 7581 (insanum).

Dechamps, R. 12366 (torvum); 13023 (torvum).

Déda, R.M. 143 (capsicoides); 178 (viarum).

Deflers, M. 377 (forskalii); 488 (cordatum).

Deguchi, K. 6317 35573 (torvum).

Deighton, F.C. 229 (torvum); 597 (torvum); 1782 (aculeatissimum); 1803 (macrocarpon); 2635 (aculeatissimum); 5470 (torvum); 5516 (wrightii).

Del Vitto, L.A. 850 (elaeagnifolium); 3558 (elaeagnifolium).

Delavay, J.M. 711 (violaceum); 1899 (violaceum).

Delavay, R.P. 898 (virginianum); 1025 (virginianum); 3147 (virginianum); 4400 (virginianum).

Dells, A. 3247 (elaeagnifolium).

Delnatte, C. 1045 (mammosum); 1049 (jamaicense); 1166 (torvum); 3099 (torvum); 3463 (torvum); 3485 (torvum).

Demange, R. 1495 (forskalii).

Demaree, D. 7674 (elaeagnifolium); 10857 (elaeagnifolium); 17916 (elaeagnifolium).

Denker, C.J.F. 2 (involucratum).

Denslow, J. 1 (jamaicense); 2393 (jamaicense).

Dent, H.C. 22 (sisymbriifolium); 97 (sisymbriifolium).

Deru, ? MIB 329 (giganteum).
Deshmukh, P.G. JESS 250 (cordatum).
Deshpande, S.D. 165729 (hovei); 165857 (hovei).
Desissa, D. DD 455 (giganteum).
Devia A, W. 141 (wrightii).
Devia, W. 141 (wrightii).
Deville, A. 285 (giganteum).
Dhetchuvi Matchu-Mandje, J.-.B. 1267 (torvum).
Dhothalri, K. BSI-9107 (torvum).
Di Fulvio, T.E. 799 (elaeagnifolium); 823 (elaeagnifolium); 825 (elaeagnifolium); 831 (elaeagnifolium); 941 (elaeagnifolium); 942 (elaeagnifolium); 949 (elaeagnifolium); 1110 (elaeagnifolium); 1112 (elaeagnifolium); 1126 (elaeagnifolium).
Díaz Vilchis, I. 124 (elaeagnifolium); 438 (elaeagnifolium).
Díaz, C. 534 (jamaicense); 534 (jamaicense); 1666 (wrightii).
Dibata, J.J. 296 (torvum).
Dickason, F.G. 5932 (mammosum); 6719 (torvum).
Dickie, J. 9 1 (arundo).
Dickoré, B. 13338 (virginianum).
Dieterlen, A. 887 (aculeatissimum).
Diggs, G.M. 2746 (wrightii); 2746 (wrightii).
Dilbert, V.C. 920 (arundo).
Dile, I.R. H 768 (arundo).
Dinelli, E. 654 (elaeagnifolium).
Dinklage, M. 1509 (aethiopicum); 3307 (torvum).
Diraviam, ? RHT-20553 (insanum).
Divakar, P. PD 2804 (hovei).
Diwakar, P.G. 184346 (hovei); 191880 (hovei).
Dixon, D. 3636 (elaeagnifolium).
Döbbeler, P. 1674 (chrysotrichum).
Dobremez, J.F. 904 (violaceum); 2731 (virginianum).
Docters van Leeuwen, W.M. 10586 (lasiocarpum).
Dodar, M.N. 179172 (hovei).
Dodson, C.H. 642 (sisymbriifolium).
Domingo, M.V.Z. 524 (elaeagnifolium).
Domínguez C, R. 1237 (elaeagnifolium).
Donnell Smith, J. 12894 (jamaicense).
Dorgelo, J.D. S 268 (involucratum).

Dorr, L.J. 4305 (torvum); 6660 (sisymbriifolium).

Dottori, N.M. 138 (elaeagnifolium); 139 (elaeagnifolium); 145 (elaeagnifolium); 146 (elaeagnifolium); 148 (elaeagnifolium); 169 (elaeagnifolium); 179 (elaeagnifolium); 180 (elaeagnifolium); 186 (elaeagnifolium).

Dowsett-Lemaire, F. 1110 (torvum).

Drennen, D.S.H. 24 (torvum).

Dreveck, S. 500 (sisymbriifolium).

Dreyfus, G. 362 (chrysotrichum).

Drummond, J.R. 13856 (cordatum); 14441 (insanum); 14448 (cordatum); 14703 (cordatum); 15259 (insanum); Kew-20549 (virginianum); 23619 (virginianum); 25858 (virginianum); 25859 (insanum); 25860 (virginianum); 25862 (insanum); 25863 (insanum); 25864 (insanum).

Drummond, R.B. 1239 (arundo); 2414 (aculeatissimum); 2422 (giganteum); 2874 (robustum).

Drummond, T. 93 (elaeagnifolium); 266 (elaeagnifolium).

Du Puy, D.J. CI 94 (insanum).

Du, N.V. HNK-2470 (torvum); HNK-2799 (torvum).

Du, Y. SCSB-00007 (torvum).

Duarte, A.P. 6579 (mammosum); 6580 (jamaicense).

Ducloux, F. 3910 (violaceum); 3911 (virginianum); 4772 (virginianum); 4773 (violaceum); 5209 (virginianum); 5888 (virginianum); 6235 (violaceum).

Dueñas G, H.C. 11 (jamaicense).

Dugand G, A. 5320 (melongena).

Duke, J.A. 7344 (torvum).

Dullas, W. 135 (elaeagnifolium).

Dulong Jiang Investigation Team 6 (violaceum).

Dümmer, R.A. 671 (giganteum); 1098 (macrocarpon); 2477 (aethiopicum); 4003 (aculeatissimum).

Dumont d'Urville, J.S.C. 211 (lasiocarpum).

Dunlap, D. 104 (torvum); 144 (torvum).

Dunlap, V.C. 262 (mammosum); 267 (jamaicense).

Dunlop, C.R. 9619 (lasiocarpum).

Dunn, D.B. 23177 (elaeagnifolium); 23026 (wrightii).

Dunnett, D. 13 (elaeagnifolium).

Duport, ? 11 (procumbens); 63 (violaceum).

Dupuy, C. 92 (involucratum); 93 (violaceum).

Duque Jaramillo, J.M. 2173 (jamaicense); 3949 (mammosum); 4035 A (torvum); 4094 (torvum); 4096 (mammosum); 4104 (sisymbriifolium); 4106 (melongena).

Duron, C. 63 (jamaicense).

Dusén, P. 5217 (elaeagnifolium); 17162 (sisymbriifolium).

Duss, A. 2881 (macrocarpon); 3832 (capsicoides); 4685 (capsicoides).

Duthie, J.F. 10481 (insanum); 10482 (insanum); 22438 (melongena).

Dutt, A.K. 664 (melongena).

Dwyer, J.D. 1525 (jamaicense); 4420 (jamaicense); 11414 (jamaicense); 11493 (jamaicense); 12679 (torvum); 14151 (elaeagnifolium); 15253 (wrightii).

Dybowski, J. 568 (giganteum).

Eastwood, A. 1040 (elaeagnifolium); 5958 (elaeagnifolium); 8550 (elaeagnifolium); 8627 (elaeagnifolium); 9301 A (elaeagnifolium); 11763 (elaeagnifolium); 13946 (elaeagnifolium); 15419 (elaeagnifolium); 15646 (elaeagnifolium); 17097 (elaeagnifolium); 18099 (elaeagnifolium).

Eaton 57 (elaeagnifolium).

Ebalo, L.E. 934 (lasiocarpum); 1344 (pseudosaponaceum).

Ebba, T. 547 (aculeatissimum).

Ebbe, J. 598 (macrocarpon).

Eberhardt, P.A. 1347 (procumbens); 2162 (procumbens); 3811 (insanum); 4265 (praetermissum).

Ebinger, J.E. 332 (jamaicense); 897 (jamaicense).

Echeverry E, R. 2100 (jamaicense).

Eckert, E.J. 69 (insanum).

Edaño, G.E. 247 (lasiocarpum); 11872 (torvum); 11990 (torvum); 36789 (torvoideum); 38321 (torvoideum); 48815 (torvoideum); BS-76376 (retrorsum); 77832 (lasiocarpum); 78556 (mammosum).

Eder, M.P. 28563 (torvoideum).

Edgeworth, M.P. 116 (insanum).

Edinburgh Nepal Expedition 21 (torvum).

Edwall, G. CGG 3965 (viarum).

Edwards, T. 2196 (aculeatissimum); 2914 (chrysotrichum).

EEAAFRO Staff 60 (giganteum).

Egea, J. de 82 (sisymbriifolium); 396 (sisymbriifolium); 535 (sisymbriifolium); 799 (robustum); 808 (sisymbriifolium); 894 (sisymbriifolium).

Eggeling, W.J. E 6690 (arundo).

Eggers, H.F.A. von 101 (torvum); 105 (torvum); 1978 (torvum); 4397 (capsicoides).

Eggleston, W.W. 7424 (elaeagnifolium).

Ehlers, J.H. 6410 (elaeagnifolium).

Eiten, G. 1926 (capsicoides); 3232 (viarum); 9432 (sisymbriifolium).

Ekman, E.L. 13942 (elaeagnifolium).

El Ghani, M.A. 6908 (elaeagnifolium).

Elbert, J. 189 (giganteum); 2401 (lasiocarpum); 3224 (poka); 3575 (torvoideum).

Elia, J. 11 (aculeatissimum); 127 (aculeatissimum).

Elisetch, ? 342 (elaeagnifolium).

Ellen, R.F. 836 (torvum).

Ellenbeck, H. 2024 (cordatum).

Elliot, B. 11929 c (elaeagnifolium).

Elliot, C. 62 (elaeagnifolium).

Ellis, J.L. SC-11769 (trilobatum); SC-14242 (insanum); ANC-18793 (torvum); 20499 (multiflorum); SC-22154 (insanum); 29496 (multiflorum); SC-32673 (insanum).

Ellis, P.E. 151 (cordatum); 268 (cordatum).

Elmer, A.D.E. 2233 (lasiocarpum); 5528 (insanum); 5610 (insanum); 5819 (lasiocarpum); 5895 (torvoideum); 6073 (retrorsum); 6204 (pseudosaponaceum); 6732 (lasiocarpum); 8214 (insanum); 8328 (lasiocarpum); 8629 (torvoideum); 8719 (retrorsum); 10277 (lasiocarpum); 10752 (schefferi); 12879 (pseudosaponaceum); 13157 (cyanocarphium); 13879 (pseudosaponaceum); 17934 (lasiocarpum); 18123 (retrorsum); 20283 (torvum); 21869 (lasiocarpum).

Elmi, A.A. 4100 (forskalii).

Els, P. 3118 (elaeagnifolium).

Endert, F.H. 3477 (lasiocarpum); 3745 (lasiocarpum).

Ensermu, K. 753 (giganteum); 818 (giganteum); 3893 (giganteum).

Enti, A.A. sp 146 (torvum).

Epenhuijsen, C.W. van 37 (macrocarpon).

Equipo 5 (elaeagnifolium).

Erickson, H.T. 23 (sisymbriifolium); 26 (viarum); 27 (viarum).

Erilles, R.P. 29 (aethiopicum).

Erlanson, E.W. 5300 (torvum); 5602 (giganteum).

Ern, H. 2131 (torvum).

Ernst, W.R. 1453 (torvum).

Ertter, B. 2939 (elaeagnifolium).

Escobedo, J.M. 928 (elaeagnifolium); 1594 (elaeagnifolium).

Escritor, L. 21415 (torvoideum); 21470 (pseudosaponaceum).

Espejo, A. 1023 (elaeagnifolium).

Espina Z, J. 1189 (jamaicense); 1924 (jamaicense); 2214 (jamaicense).

Espinal T, S. 564 (torvum); 1552 (sisymbriifolium); 2264 (sisymbriifolium).

Espinal, S. 2531 (jamaicense); 2748 (jamaicense).

Espinosa, R. E-40 (sisymbriifolium).

Espíritu, R.A. 740 (torvum).

Esquirol, J.H. 931 p.p. (pseudosaponaceum).

Etuge, M. 1644 (torvum).

Eugênio, C. 19 (capsicoides).

Evans, J.W. 93 (sisymbriifolium).

Evrard, C. 1193 (giganteum).

Evrard, C.M. 2443 (lasiocarpum).

Evrard, F. 174 (violaceum); 176 (insanum); 426 (insanum); 819 (insanum); 1231 (camranhense); 1331 (violaceum); 1530 (nienkui).

Ewan, J.A. 15 (elaeagnifolium); 680 (elaeagnifolium).

Exell, A.W. 541 (capsicoides); 840 (torvum).

Expeditio Biologica Sino-Rossica 1955 567/ 74 (violaceum).

Expedition Novara 134 (melongena).

Expedition Team 10159 (barbisetum); 32117 (lasiocarpum); 34605 (lasiocarpum).

Eyerdam, W.J. 23093 (elaeagnifolium); 23735 (elaeagnifolium).

Eyma, P.J. 2166 (poka).

F.F.B. 236 (multiflorum).

Faber, E. 190 (pseudosaponaceum); 612 (virginianum).

Fabris, H.A. 8357 (elaeagnifolium).

Faden, R.B. 376 (giganteum); 71/ 394 (arundo); 413 (aculeatissimum); 68/ 534 (giganteum); 68/ 565 (aculeatissimum).

Fairbairn, W.A. 4 (torvum); 41 (torvum); 42 (torvum).

Falcao, J.I. 954 (jamaicense).

Falconer, H. 13 (violaceum); 35 (barbisetum); 38 (lasiocarpum); S 106 (violaceum).

Fallen, M. 328 (lasiocarpum).

Fan, C.S. 688 (capsicoides).

Fang, L.F. LHF-06 (torvum).

Fanshawe, D.B. 6871 (chrysotrichum); 6882 (chrysotrichum); 7304 (aculeatissimum).

Farnsworth, E.L. 310 (elaeagnifolium).

Faurie, U. 319 (capsicoides); 323 (violaceum); 646 (violaceum); 647 (lasiocarpum); 648 (pseudosaponaceum); 860 (capsicoides); 1158 (capsicoides); 1193 (violaceum); 1194 (violaceum); 1484 (capsicoides).

Fay, J.M. 5424 (macrocarpon).

Felger, R.S. 86-400 (elaeagnifolium); 85-795 (elaeagnifolium).

Felicite, N. 82 (torvum).

Felippone, F. 5092 (elaeagnifolium); 6166 (elaeagnifolium); 6200 (sisymbriifolium).

Félix, L.P. 3425 (capsicoides); HST 5526 (jamaicense).

Fendler, A. 610 (mammosum); 611 (jamaicense); 672 (elaeagnifolium); 993 (jamaicense).

Feng, G. 11429 (deflexicarpum).

Fénix, E. 106 (violaceum); 3565 (insanum); 12934 (retrorsum); 15801 (pseudosaponaceum); 26103 (lasiocarpum).

Fernandes, J. 172 (torvum); 1055 (giganteum); 1098 (torvum); 1100 (giganteum); 1134 (torvum); 1265 (multiflorum); 1332 (torvum); 1377 (torvum); 1735 (torvum); 1748 (hovei); 1865 (hovei); 1924 (torvum); 2197 (lasiocarpum); 2333 (torvum); 2335 (giganteum).

Fernández Alonso, J.L. 5669 (jamaicense); 7311 A (jamaicense); 11460 (torvum); 16018 (jamaicense); 16033 (jamaicense); 16196 (jamaicense); 22978 (jamaicense).

Fernández P, A. 289 (jamaicense); 307 (jamaicense); 2732 (sisymbriifolium).

Fernández, A. 1318 (jamaicense).

Fernández, Á. 13858 (sisymbriifolium).

Fernández, A. 17925 (jamaicense).

Fernández, J. JF 1223 (multiflorum).

Fernández, J.R. 156 (elaeagnifolium).

Fernández-Casas, J. 3640 (robustum); 3743 (sisymbriifolium); 3743 (sisymbriifolium); 3912 (sisymbriifolium); 3912 (sisymbriifolium); 5689 (sisymbriifolium); 7284 (sisymbriifolium); 8433 (sisymbriifolium); 10059 (torvum); 10195 (torvum); 10743 (torvum); 11029 (elaeagnifolium); 11162 (elaeagnifolium); 11355 (torvum); 11540 (torvum); 11619 (torvum); 11713 (torvum); 11991 (aethiopicum).

Ferrari, G. 1662 (jamaicense).

Ferrari, R.V. 313 (elaeagnifolium).

Ferreira, L.V. 54 (viarum).

Ferreyra, R. 3697 (mammosum); 4752 (sisymbriifolium); 4863 (sisymbriifolium); 7945 (sisymbriifolium); 10111 (sisymbriifolium); 12768 (capsicoides); 15221 (sisymbriifolium); 16217 (mammosum); 19367 (chrysotrichum); 20464 (sisymbriifolium).

Ferris, R.S. 1153 (elaeagnifolium); 2302 (elaeagnifolium); 2720 (elaeagnifolium); 3133 (elaeagnifolium).

Ferrucci, M.S. 2953 (elaeagnifolium); 2979 (elaeagnifolium).

Fertig, W. 22568 (elaeagnifolium).

Festo, L. 554 (aculeatissimum); 1527 (aculeatissimum).

Fiebrig, K. 814 (robustum); 5752 (robustum); 6291 (robustum).

Field, C.R. ALP 122 (forskalii); ALP 268 (forskalii).

Fiennes, R.T.W. 50 (cordatum).

Figueroa, L. 169 (torvum).

Filippa, E.M. 79 (elaeagnifolium).

Fiori, A. 685 (forskalii); 686 (forskalii).

Fischer, C.E.C. 444 (trilobatum); 577 (torvum); 733 (wightii); 1552 (robustum); 2955 (multiflorum).

Fischer, W. 238 (elaeagnifolium).

Fisher, G.L. 5089 (elaeagnifolium).

Fisher, J.D. 91 (melongena).

Fleury, F. 29910 (involucratum); 32439 (praetermissum).

Fliervoet, E.L.S. 803 A (arundo).

Flint, L. 13 (torvum).

Florêncio, F.J. 66 (torvum).

Flores, J. JF-51 (wrightii); JF-105 (wrightii).

Flores, R.M. 2 (elaeagnifolium).

Flores, S. 167 (mammosum).

Floto, F. 7617 (melongena); 7821 (trilobatum).

Floyd, A.G. 3506 (torvum).

Floyer 1 (elaeagnifolium).

Flügel, H.J. 1046 (hovei).

Folsom, J. 8941 (jamaicense).

Fonnegra, R. 954 (jamaicense); 2513 (wrightii); 4851 (jamaicense); 4870 (jamaicense); 5023 (jamaicense); 6301 (jamaicense); 7276 (jamaicense).

Forbes, F.B. 601 (wrightii); 613 (torvum).

Forbes, H.O. 10 (torvoideum); 438 (lasiocarpum); 949 b (torvum); 994 (torvum); 1253 (torvum); 1270 (lasiocarpum); 2085 (torvum); 2316 (lasiocarpum); 3634 (involucratum); 3786 (melongena); 3806 (poka); 3841 (violaceum); 4008 (involucratum); 4091 (insanum); 4096 (involucratum).

Ford, C.A. 123 (violaceum); 536 (torvum).

Ford, J. 303 (wrightii).

Foreman, D.B. LAE-60091 (torvoideum).

Forero P, L.E. 588 (mammosum); 1548 (wrightii).

Forero, E. 805 (jamaicense); 1581 (jamaicense); 1824 (jamaicense); 2523 (jamaicense); 3059 (mammosum); 4589 (torvum); 5242 (jamaicense); 5249 (jamaicense); 5467 (jamaicense); 6694 (jamaicense); 8964 (jamaicense).

Forest District Officer 28 (insanum).

Forke 1 (jamaicense).

Foroughi, H. 10812 (virginianum).

Forrest, G. 4483 (melongena); 6553 (viarum); 9103 (viarum); 9239 (torvum); 29576 (violaceum).

Förther, H. 10865 (torvum); 11080 (chrysotrichum).

Fortuna, J. 22 (elaeagnifolium).

Fortunato, R.H. 1242 (sisymbriifolium); 2122 (robustum); 2401 (robustum); 2424 (elaeagnifolium); 2951 (sisymbriifolium); 3095 (elaeagnifolium); 3987 (sisymbriifolium); 9916 (elaeagnifolium); 9942 (elaeagnifolium).

Fosberg, F.R. S-3399 (elaeagnifolium); 36811 (hovei); 50081 (violaceum); 52845 (insanum);53483 (violaceum); 56928 (insanum); 58158 (violaceum); 59353 (torvum).

Foster, R. 3935 (elaeagnifolium); 14909 (torvum).

Foulkes, G.F.F. 83 (insanum).

Fourcade, G.H. 643 (aculeatissimum).

Fox, R.B. 4793 (insanum); 13390 (lasiocarpum).

Frake, C.M. PNH 35973 (cyanocarphium).

Frake, C.O. 20323 (torvoideum); 38012 (torvoideum).

Franc, I. 508 (torvum); 841 (insanum).

Francisco, E.M. 128 (robustum).

Franck, C.W. 413 (violaceum).

Fraser, J. 120 (torvum).

Fraume, M. de 1104 A (jamaicense).

Freije, N. 352 (jamaicense).

Freitag, H. 51 (virginianum).

Freitas, L. 742 (viarum); 840 (sisymbriifolium).

Frenkel, R.E. 841 A (elaeagnifolium).

Frey, R. 474 (sisymbriifolium); 576 (sisymbriifolium).

Friedrichsthal, E. von 1788 (mammosum).

Fries, K.R.E. 1676 (sisymbriifolium).

Friis, I. 2247 (aculeatissimum); 2927 (cordatum); 4564 (arundo); 4766 (cordatum); 8494 (wrightii); 9783 (giganteum); 10378 (forskalii); 10581 (cordatum); 15208 (cordatum).

Frodin, D.G. 2413 (lasiocarpum).

Frye, T.C. 2383 (elaeagnifolium).

Fryxell, P.A. 5131 (elaeagnifolium).

Fuentes, A.F. 6183 (sisymbriifolium).

Fuertes, J. 260 (jamaicense).

Fujikawa, K. 35960 (torvum); 50117 (violaceum); 53092 (violaceum); 53095 (torvum); 53218 (viarum); 53219 (violaceum); 86161 (torvum); 86627 (torvum); 86711 (torvum); 86766 (torvum); 86914 (torvum); 90739 (torvum); 95433 (violaceum).

Fujimoto, T. 610 (macrocarpon).

Fukuoka, N. T 7083 (trilobatum); T-36247 (trilobatum).

Fuller, T.C. 8567 (elaeagnifolium); 9706 (elaeagnifolium); 9796 (elaeagnifolium); 11226 (elaeagnifolium); 19758 (elaeagnifolium).

Funez, L.A. 249 (sisymbriifolium); 403 (torvum); 683 (capsicoides); 858 (torvum); 1370 (aculeatissimum).

Funk, V.A. 10414 (jamaicense); 10743 (torvum).

Furet, L. 200 (insanum).

Gachalian, F.S. 14937 (violaceum).

Gadd, G. 74 (virginianum).

Gadelha Neto, P.C. 1134 (capsicoides).

Gaerlan 4887 (torvum); 5287 (schefferi).

Gaetan, ? 84 (aculeatissimum).

Gafui, I. BSIP-10800 (schefferi).

Gagah, C.N. H 460 (arundo).

Gage, A.T. 4 (lasiocarpum); 5 (torvum); 6 (violaceum); 145 (lasiocarpum).

Galán, P. 1583 (torvum); 2069 (torvum); 2299 (torvum); 2870 (torvum); 3579 (chrysotrichum).

Galassi, S.C. CFSC 13084 (viarum).

Gale, U.M.G. 12310 (virginianum).

Galeano, G. 9258 (mammosum).

Galeotti, H.G. 1225 Q (chrysotrichum).

Galetto, L. 24 (elaeagnifolium); 214 (elaeagnifolium); 232 (elaeagnifolium); 237 (elaeagnifolium); 239 (elaeagnifolium); 260 (elaeagnifolium).

Gallegos Harking, F. 81 (elaeagnifolium).

Galpin, E.E. 11868 (torvum); 12986 (sisymbriifolium); 14039 (aculeatissimum).

Galván, M. 618 (elaeagnifolium).

Galván, R. 2761 (elaeagnifolium); 3614 (elaeagnifolium).

Galvão, L.S. 1 (torvum).

Gambating, A. SAN 107137 (cyanocarphium).

Gamble, J.S. 2460 A (torvum); 8966 (virginianum); 10490 (barbisetum); 10716 (trilobatum); 10987 (pubescens); 11265 (multiflorum); 11419 (giganteum); 11601 (wightii); 12189 (trilobatum); 12414 (wightii); 14294 (giganteum); 14490 (wightii); 14498 (wightii); 15071 (violaceum); 15173 (pubescens); 15787 (violaceum); 15829 (insanum); 15872 (trilobatum); 16045 (insanum); 16273 (violaceum); 16274 (torvum); 16275 (multiflorum); 16326 (melongena); 16874 (wightii); 16879 (wightii); 17077 (virginianum); 17376 (multiflorum); 17721 (insanum); 18677 (pubescens); 20879 (pubescens); 21410 (wightii); 21597 (insanum); 21672 (virginianum).

Gammie, G.A. 287 (insanum); 301 (sisymbriifolium); 638 (wightii); 15191 (hovei); 15593 (hovei).

Gandhi, K.N. HFP-2075 (violaceum).

Gaoligong Shan Expedition 7981 (viarum); 8570 (torvum); 8572 (violaceum); 9920 (torvum); 10444 (violaceum); 10529 (torvum); 10998 (aethiopicum).

Gaoligong Shan Biodiversity Survey 17207 (violaceum); 17239 (torvum); 17539 (torvum); 18145 (torvum); 18147 (violaceum); 18499 (viarum); 19935 (violaceum); 20957 (viarum); 23448

(torvum); 23513 (violaceum); 23859 (torvum); 24730 (aculeatissimum); 26298 (violaceum); 28945 (aculeatissimum); 28947 (violaceum); 29058 (viarum).

García M, E. 322 (elaeagnifolium).

García P, J. 670 (elaeagnifolium).

García R, L.A. 1181 (elaeagnifolium).

García, A. 40 (elaeagnifolium).

García, A.L. 1033 (sisymbriifolium).

García, D. 101 (jamaicense).

Garcia, E.M. 413 (elaeagnifolium).

Garcia, J.D. 828 (jamaicense).

García, L. 59 (jamaicense).

García, M.C. 494 (jamaicense).

García, R.J. 23 (elaeagnifolium).

Garcia, R.J.F. 2310 (robustum).

García-Barriga, H. 4369 (sisymbriifolium); 6390 (torvum); 7133 (mammosum); 12411 (jamaicense); 12987 (mammosum); 12995 (jamaicense); 18683 (mammosum); 20148 (jamaicense); 21219 (mammosum).

Gardner, G. 552 (sisymbriifolium); 629 (torvum); 630 (violaceum); 631 (lasiocarpum); 837 (sisymbriifolium); 3914 (viarum); 5043 (sisymbriifolium).

Gardner, H.M. K 1149 (giganteum).

Gardner, M.F. 58 (elaeagnifolium); 4346 (elaeagnifolium).

Gardner, P.C. 1633 (viarum); 1638 (torvum).

Gardner, T.S. 35 (torvum); 86 (torvum).

Garrett, H.B.G. 1093 (lasiocarpum); 1253 (barbisetum).

Garrette, G.H. 22 (melongena).

Gartner N, A. 23 (torvum).

Garwood, N.C. 582 (torvum); 651 (torvum); 664 (torvum); 1503 A (jamaicense); 1896 A (jamaicense).

Gaston, A. 1162 (forskalii).

Gaudichaud, C. 94 (insanum); 138 (lasiocarpum); 145 (insanum); 475 (aethiopicum).

Gaumer, G.F. 1167 (torvum).

Gautier, L. LG 2422 (torvum); 4665 (torvum); LG 52265 (torvum).

Gay, C. 834 (elaeagnifolium).

Gbile, E.C. 62860 (aculeatissimum).

Gbile, Z.O. FHI 20550 (torvum); FHI 73662 (torvum).

Gedye, ? 7359 (arundo).

Geilinger, G. 1578 (wrightii).

Genelle, P. 819 (elaeagnifolium).

Geno, J.M. 77 (dunalianum).

Gentle, P.H. 21 (jamaicense); 3976 (torvum); 8809 (jamaicense).

Gentry, A.H. 1255 (torvum); 8453 (jamaicense); 15253 (jamaicense); 15475 (jamaicense); 15756 (jamaicense); 17539 (jamaicense); 17734 (jamaicense); 18223 (jamaicense); 20062 (jamaicense); 20248 (jamaicense); 21216 (jamaicense); 23222 (sisymbriifolium); 50597 (jamaicense).

Gentry, H.S. 51-393 (elaeagnifolium).

Gentry, J.L. 2779 (torvum); 2838 (torvum); 2865 (chrysotrichum); 2880 (chrysotrichum); 3033 (jamaicense).

Geoffray, C. 69 (lasiocarpum); 79 (insanum).

George, P. SAN 117639 (cyanocarphium).

Gerard, P. 2461 (giganteum); 3698 (giganteum).

Gereau, R.E. 2973 (giganteum); 4053 (aculeatissimum); 6430 (aculeatissimum).

Gereau, R.E.; Aduake, ?; Esulanji, ? 5409 (torvum).

Gereau, R.E.; Lovett, J.; Kayombo, C.J. 3494 (aculeatissimum).

Germain, R.G.A. 5187 (giganteum).

Getahun, A. 371 (aculeatissimum).

Ghafoor, A. 4953 (virginianum).

Ghogue, J.-.P. 67 (torvum).

Ghose, M.K. CNH-2637 (trilobatum).

Ghosh, M.K. CNH-133 (trilobatum); CNH-10944 (hovei); CSH-10968 (hovei).

Giacomelli, A. 88900 (elaeagnifolium).

Giacomin, L.L. 204 (robustum); 348 (viarum); 1093 (sisymbriifolium); 1236 (capsicoides); 1610 (robustum); 1726 (aculeatissimum).

Gibbs, L.S. 840 (torvum); 2639 (torvum).

Gibbs, P.E. 5328 (sisymbriifolium).

Gibert, E.J. 145 (elaeagnifolium).

Gibson, G.D. 18 (aethiopicum).

Gideon, O. LAE 77094 (lasiocarpum).

Giffen, M.F. 1437 (giganteum).

Gilbert, ? 2216 (giganteum).

Gilbert, G. 2146 (giganteum).

Gilbert, M.G. 57 (procumbens); 130 (cordatum); 341 (procumbens); 449 (capsicoides); 3993 (cordatum); 4855 (aculeatissimum); 4855 (aculeatissimum); 5530 (arundo).

Gilbert, V.C. E 8 (aculeatissimum); 2272 (aculeatissimum).

Gillespie, J.W. 5222 (elaeagnifolium).

Gillespie, L.J. 701 (jamaicense).

Gillett, J.B. 3203 (aculeatissimum); 4097 (cordatum); 4877 (cordatum); 4983 (melongena); 12790 (cordatum); 15284 (torvum); 16596 (wrightii); 19174 (arundo); 19248 (arundo); 20453 (wrightii); 21581 (arundo); 22357 (forskalii); 22390 b (forskalii); 22406 (cordatum); 24160 (forskalii); 24477 (arundo).

Gillett, J.M. 17013 (elaeagnifolium).

Gilli, A. 102 (torvoideum); 287 (torvoideum); 497 (robustum); 498 (robustum); 3245 (melongena); 3246 (melongena).

Gillies, J. Solanum 2 (elaeagnifolium); 28 (elaeagnifolium); 669 (elaeagnifolium).

Gilliland, H.B. 1037 (aculeatissimum).

Gillison, A.N. NGF 22120 (lasiocarpum).

Gillman, H. 1313 (giganteum).

Ging, T.S. 6735 (violaceum).

Giorgi, S. de 1563 (giganteum).

Giorgis, M. 693 (elaeagnifolium).

Giulietti, A.M. CFSC 12482 (viarum).

Gjellerup, K. 651 (lasiocarpum).

Glass, V. 10674 (elaeagnifolium).

Glaziou, A.F.M. 8842 (robustum); 8842 (robustum).

Gledhill, D. 40 (torvum); 771 (torvum); 860 (torvum).

Glocker, E.F. von 120 (capsicoides); 535 (melongena); 594 (jamaicense).

Glover, P.E. 1258 (forskalii); 3495 (arundo).

Glover, P.E.; Gwynne, M.D.; Samuel, S.P.; Tucker, H. 2074 (aculeatissimum).

Goddard, D.R. 836 (elaeagnifolium).

Godefroy, A. 16 (insanum).

Godefroy-Lebeuf, M. 112 (lasiocarpum).

Goel, A.K. NC-65880 (violaceum).

Goldblatt, P. 8399 (giganteum).

Gomes, F.S. 968 (robustum).

Gómez Lorence, F. 3 (elaeagnifolium).

Gómez Marín, E. 11 (torvum).

Gómez, A. 3 (elaeagnifolium); 13 (elaeagnifolium).

Gómez, J.A. 1 (wrightii).

Gómez, M.G. 66 (wrightii).

Gómez, N. 9071 (barbisetum).

Gonzales, A. 491 (jamaicense).

Gonzáles, P. 1993 (chrysotrichum); 2127 (sisymbriifolium).

González Elizondo, M.S. 140 (jamaicense).

González Medrano, F. 8003 (elaeagnifolium); 8013 (elaeagnifolium).

González, F. 2588 (torvum); 4148 (sisymbriifolium).

González, J. 1544 (jamaicense).

González, M. 10 (elaeagnifolium).

González, M.F. 76 (wrightii).

González, R. 12 (jamaicense).

González-Medrano, F. 39 (elaeagnifolium); 115 (elaeagnifolium); 8131 (elaeagnifolium); 12993 (elaeagnifolium).

Gooding, E.G.B. 194 (torvum); 196 (capsicoides).

Goodman, G.J. 1142 (elaeagnifolium).

Gopalan, V.J. 67255 (multiflorum).

Goring, V. 11 (torvum).

Gossweiler, J. 7060 (aethiopicum); 7160 (aethiopicum); 7445 (aethiopicum); 7446 (macrocarpon); 7966 (aethiopicum); 8371 (macrocarpon); 8914 (aculeatissimum); 8960 b (mammosum); 8960 (aethiopicum); 9296 (aethiopicum); 9400 (aethiopicum).

Gouvêa, Y.F. 139 (robustum); 162 (robustum); 172 (capsicoides).

Goyder, D.J. 3564 (aculeatissimum).

Graham, E.H. 3021 (elaeagnifolium); 3619 (elaeagnifolium).

Graham, J.G. 1091 (mammosum).

Granados T, J.C. 901 (jamaicense).

Grant, G.B. 5550 (elaeagnifolium).

Grant, J.R. 91 01521 (chrysotrichum); 92 01649 (chrysotrichum).

Gray, E.C. NGF-8094 (torvoideum).

Grayum, M.H. 3799 (chrysotrichum); 8136 (torvum); 10358 (chrysotrichum).

Greensword, J. H 2 (aethiopicum).

Greenway, P.J. 2277 (wrightii); 3074 (giganteum); 4183 (giganteum); 6061 (aethiopicum); 6751 (arundo); 12167 (arundo).

Greenway, P.J.; Kanuri, K. 12435 (aculeatissimum).

Gregory, L.E. 1853 (chrysotrichum).

Greijmans, M. 57-98 (lasiocarpum).

Gressitt, J.L. 907 (nienkui).

Greuter, W. 25099 (jamaicense); 26051 (jamaicense); 26615 (torvum); 26627 (torvum); 26913 (torvum).

Grey-Wilson, C. 245 (virginianum).

Grierson, A.J.C. 1393 (torvum); 2164 (torvum); 2214 (torvum); 2675 (violaceum); 3378 (torvum).

Griffith, R. 99 (forskalii).

Griffith, W. 614 (violaceum); 998 (lasiocarpum); 1000 (violaceum); 2352 (violaceum); 5906 (virginianum); 5906 [a] (virginianum); 5907 (trilobatum); 5909 (barbisetum); 5910 (lasiocarpum); 5911 /1 (praetermissum); 5912 [a] (violaceum); 5912 [b] (violaceum); 5912 [c] (violaceum); 5912 [d] (melongena); 5913 (melongena); 5914 (cyanocarphium); 5915 (trilobatum).

Griffiths, G. 216 (elaeagnifolium); 267 (elaeagnifolium).

Griffon du Bellay, M.-.T. 298 (torvum).

Grifo, F.T. 51 (capsicoides); 816 (robustum).

Grijalva Pineda, A. 167 (chrysotrichum); 2523 (wrightii); 2702 (wrightii); 2911 (wrightii).

Grimshaw, J.M. 94-192 (aculeatissimum); 94-570 (aculeatissimum).

Gross, C.A. 82 (elaeagnifolium).

Grote, M. 8073 (giganteum).

Groves, M. 51 (torvum).

Grumbler, L.A.S. 16601 (aethiopicum).

Guaglianone, E.R. 1555 (elaeagnifolium); 1571 (elaeagnifolium).

Guedes, M.L. 6324 (robustum); 7654 (viarum); 19257 (robustum).

Gueho, J. 15121 (torvum).

Guerra, O. 601 (elaeagnifolium).

Guerra, T.P. 76 (robustum).

Guerreo, O. 1 (chrysotrichum).

Guerrero, I. 24 (mammosum).

Guichard, K. KG 33 (pubescens).

Guilding, L. 40 (torvum).

Guillén, R. 3703 (jamaicense).

Guimarães, E.A. 5 (torvum).

Guinea, E. 1156 (torvum).

Guízar N, E. 4031 (elaeagnifolium).

Gullion, G.W. 383 (elaeagnifolium).

Gust, G. 2206 (elaeagnifolium).

Gustafson, R. 1955 (jamaicense).

Gutiérrez V, G. 17 C 160 A (jamaicense); 320 (sisymbriifolium).

Gutiérrez, G. 1025 (jamaicense).

Gutiérrez, H.G. 78007 (lasiocarpum).

Gutierrez, L. 10 (jamaicense).

Gutiérrez, M. 548 (sisymbriifolium).

Gutzwiller, R. 3124 (giganteum).

Guzmán C, U. 2947 (elaeagnifolium); 3013 (elaeagnifolium).

Guzmán, C.A. 11 (elaeagnifolium); 44 (elaeagnifolium).

Guzmán, R. 999 (elaeagnifolium).

Guzmán-Teare, M. 1630 (wrightii).

Haarer, A.E. 936 (aculeatissimum); 1037 (aculeatissimum); 1868 (robustum); 2441 (giganteum).

Haber, W.A. 5 (chrysotrichum); 376 (torvum); 11777 (torvum).

Haegens, R.M.A.P. 81 (torvum); 340 (lasiocarpum).

Haene, E. 61 (elaeagnifolium).

Haerdi, F. 52 92 (macrocarpon).

Hage, J.L. 1981 (torvum).

Hahn, L. 10 (lasiocarpum); 410 (torvum); 625 (jamaicense).

Hahn, W. 685 (robustum); 1420 (elaeagnifolium); 1494 (robustum); 3814 (jamaicense); 4828 (jamaicense).

Haines, H.H. 146 (torvum); 462 (violaceum); 674 [a] (torvum); 674 [b] (violaceum); 2113 (virginianum); 3891 (trilobatum); 4180 (giganteum); 4918 (trilobatum).

Hajra, P.K. EC-37373 (aethiopicum); NC-82586 (insanum).

Hall, E. 498 (elaeagnifolium).

Hall, J.B. 47102 (torvum); 47103 (macrocarpon); 47106 (aethiopicum); 47108 (aethiopicum); 47123 (macrocarpon); 47124 (macrocarpon); 47128 (macrocarpon); GC 47129 (aethiopicum); 47132 (macrocarpon); 47133 (macrocarpon); 47135 (melongena); 47136 (melongena); 47137 (macrocarpon); 47138 (macrocarpon).

Hall, J.S. 7891 (wrightii).

Hallé, N. 3633 (torvum); 3854 (torvum); 4922 (aethiopicum).

Hallier, H. 416 (torvoideum); 4185 (lasiocarpum); 4308 (torvoideum); 4476 (torvoideum).

Hallier, J.G. 220 (involucratum).

Halse, R.R. 568 (elaeagnifolium).

Hammel, B. 12027 (jamaicense); 19789 (wrightii); 20629 (mammosum).

Hampshire, R.J. 1225 (chrysotrichum).

Hance, H.F. 860 (insanum); 860 [b] (insanum); 860 [a] (violaceum); 1285 (wrightii); 2483 (lasiocarpum); 5440 (pseudosaponaceum); 7565 (violaceum); 13816 (procumbens).

Hancock, A.D. 39 (torvum).

Hanekom, W.J. 554 (elaeagnifolium).

Haniff, M. 3852 (violaceum); 3873 (involucratum); 4351 (trilobatum).

Hannington, C. 23 (elaeagnifolium).

Hansen, B. 1392 (elaeagnifolium); 1791 (elaeagnifolium); 9321 (capsicoides); 10994 (torvum); 10995 (violaceum).

Hansen, C.J. 4421 (elaeagnifolium).

Hansen, G. 1174 (elaeagnifolium).

Hansen, O.J. 119 (robustum).

Hanson, C.G. S.L.-98 -91 (torvum); ARG-99-16* A (sisymbriifolium); ARG-99-16* A (sisymbriifolium); 144 (sisymbriifolium); 323 (sisymbriifolium).

Hara, H. 1536 (viarum); 63-03526 (viarum); 63-03530 (violaceum); 63-03534 (torvum); 63-03540 (torvum); 24963 (violaceum).

Hardeveld, C. van 12 (violaceum); 153 (violaceum).

Hardham, C.B. 2628 (elaeagnifolium); 2804 (elaeagnifolium).

Hardial 239 (torvum); 497 (torvum).

Harding, J.J. 819 (aculeatissimum).

Harley, R.M. 21567 (sisymbriifolium).

Harling, G.W. 6039 (sisymbriifolium).

Harmon, W.E. 5944 (wrightii); 6225 (chrysotrichum).

Harper, R.M. 1518 (sisymbriifolium).

Harrington, H. 7966 (elaeagnifolium).

Harris, B.J. 4734 (arundo).

Harris, C.M. 1123 (giganteum).

Harris, T. 72 (giganteum); 410 (aculeatissimum).

Harris, W. 6356 (mammosum).

Hart, T.B. 338 (torvum).

Hartley, T.G. 10078 (dunalianum); 10405 (lasiocarpum); TGH-11579 (torvoideum).

Hartman, R.L. 65234 (elaeagnifolium).

Hartweg, K.T. 203 (elaeagnifolium).

Harvey, D.R. 585 (elaeagnifolium).

Harvey, Y.B. 74 (torvum).

Hasskarl, J. 1018 (involucratum).

Hassler, É. 473 (sisymbriifolium); 477 (robustum); 813 (sisymbriifolium); 987 (robustum); 1606 (viarum); 2385 (robustum); 2386 (viarum); 3079 (robustum); 3081 (sisymbriifolium); 3112 (viarum); 7557 (robustum); 11728 (robustum); 11773 (viarum); 12234 (sisymbriifolium).

Haswell, D.R. 97 (torvum).

Hatschbach, G.G. 24255 (sisymbriifolium); 39931 (sisymbriifolium); 40741 (sisymbriifolium); 42229 (sisymbriifolium); 43487 (aculeatissimum); 44522 (capsicoides); 45583 (sisymbriifolium); 49264 (sisymbriifolium); 50592 (robustum); 51331 (robustum); 52860 (robustum); 57107 (robustum); 59396 (sisymbriifolium); 61015 (sisymbriifolium); 63460 (sisymbriifolium); 65229 (robustum); 65667 (sisymbriifolium); 68290 (robustum); 78050 (sisymbriifolium).

Haught, O. 1975 (jamaicense); 2168 (torvum); 6297 (sisymbriifolium).

Hawkes, J.G. 1166 (elaeagnifolium); 1463 (elaeagnifolium).

Hawkins, T. 1285 (torvum).

Hayakawa's collector S-86 (capsicoides).

Hayata, B. 150 (violaceum); 349 (nienkui); 800 (nienkui); 891 A (insanum).

Hayes, S. 394 (mammosum).

Head, D. 81 (torvum).

Hedberg, K.O. 280 (aculeatissimum).

Heels 255 (melongena).

Heiner, A. 344 (viarum).

Heinig, R.L. 44 (torvum); 45 (torvum).

Heiser, C.B. 4978 [b] (sisymbriifolium); 9517 (lasiocarpum).

Helberg, J.J. 2528 (elaeagnifolium).

Helfer, J.W. 24 (violaceum); 104 (torvum); 5908 (torvum); 5910 (lasiocarpum); 5911 (barbisetum).

Heller, A.A. 1511 (elaeagnifolium); 3733 (elaeagnifolium); 4185 (elaeagnifolium); 6089 (torvum).

Hemadri, K. 94357 (hovei); 99573 (hovei); 102360 (hovei); 107521 (hovei).

Hemming, C.F. 85 (arundo); 152 (forskalii); 186 (cordatum); 521 (forskalii).

Henderson, D.M. 6848 (elaeagnifolium).

Hendrian, R. 964 (poka).

Hendrickx, F.L. 4332 (aculeatissimum).

Henkel, T.W. 2670 (jamaicense); 2670 (jamaicense).

Hennings, C.F. 1331 (arundo).

Henrickson, J. 5708 (elaeagnifolium); 6673 a (elaeagnifolium); 7444 (elaeagnifolium); 7917 (elaeagnifolium).

Henry, A. 259 (pseudosaponaceum); 358 (lasiocarpum); 746 (pseudosaponaceum); 795 (insanum); 795 A (insanum); 2792 (melongena); 8056 (procumbens); 8068 (insanum); 8140 (lasiocarpum); 8654 (lasiocarpum); 8856 (virginianum); 12158 (deflexicarpum); 12552 (torvum); 13592 (virginianum).

Henry, A.N. 2 (trilobatum); CNH-6 (insanum); 13 (elaeagnifolium); 20 (robustum); 65 (capsicoides); 17392 (multiflorum); 19811 (multiflorum); 19900 (vagum); 45403 (trilobatum); 49661 (multiflorum); SC-53308 (insanum).

Henty, E.E. NGF-11553 (torvoideum); NGF-11646 (torvoideum); NGF-49450 (lasiocarpum).

Henz, E. 26472 (sisymbriifolium).

Hepper, F.N. 1949 (aculeatissimum); 2209 (torvum); 2511 (torvum); 4402 (melongena); 4411 (torvum); 4415 (torvum); 4482 (torvum); 4497 (insanum); 4500 (torvum); 4506 (torvum); 4518 (violaceum); 4519 (torvum); 4548 (melongena); 4575 (violaceum); 4576 (torvum); 4581 (giganteum); 4582 (violaceum); 4585 (giganteum); 4599 (violaceum); 4617 (violaceum); 4633 (violaceum); 4641 (insanum); 4642 a (insanum); 4642 b (melongena); 4643 (insanum); 4646 (insanum); 4649 (torvum); 4665 (violaceum); 4675 (torvum); 4704 (insanum); 4713 (torvum); 4718 (violaceum); 4719 (insanum); 4769 (violaceum); 4770 (melongena); 4913

(aculeatissimum); 4985 (aculeatissimum); 7262 (arundo); 7273 (forskalii); 7338 (aculeatissimum); 7353 (aculeatissimum); 7586 (torvum); 7773 (torvum).

Herb. East India Company 5910 (lasiocarpum).

Herb. Gadeceau 5106 (mammosum).

Herb. Griffith 5912 (multiflorum).

Herb. Miers 1416 (sisymbriifolium).

Herb. Pallas 80 (violaceum); 2097 (melongena).

Herbarium Boschproefstation 5413 (involucratum).

Herbert, P.S. S.H. 1047 (hovei).

Herbst, D. 7170 (torvum).

Heringer, E.P. 1292 (sisymbriifolium); 1336 (viarum); 2944 (sisymbriifolium); 15949 (sisymbriifolium); 16884 (sisymbriifolium); 17683 -a (sisymbriifolium).

Herlocker, D. H 159 (arundo).

Hermann, M. 309 (wrightii); 323 (mammosum).

Hermengild, S. 4965 (hovei); 5609 (hovei); 6947 (hovei); 9693 (hovei); 9694 (hovei); 12702 (hovei); 15831 (hovei); 16096 (hovei); 17518 (hovei); 17519 (hovei); 17573 (hovei); 18879 (hovei); 19384 (hovei); 20946 (hovei); 20947 (hovei); 21111 (hovei); 21112 (hovei); 21113 (hovei); 21192 (hovei); 22454 (hovei); 22459 (hovei); 22461 (hovei); 22463 (hovei); 22465 (hovei); 22754 (hovei); 23153 (hovei); 28419 (hovei); 28850 (hovei).

Hernández Magaña, R. 4642 (elaeagnifolium); 7714 (elaeagnifolium); 8185 (elaeagnifolium); 8352 (elaeagnifolium); 8415 (elaeagnifolium); 9752 (elaeagnifolium).

Hernández X, E. X-2362 (elaeagnifolium); X-2493 (elaeagnifolium).

Hernández, J.J. 175 (jamaicense).

Hernani, L. 548 (mammosum).

Herre, A.W. 333 (dunalianum).

Herter, W.G. 7290 (elaeagnifolium).

Herter, W.G.F. 51 (sisymbriifolium); 87290 (elaeagnifolium).

Hespenheide, H.A. 8 (elaeagnifolium).

Heudelot, J. 417 (forskalii).

Hewer, T.F. 1344 (virginianum).

Heyne, B. Wall. cat. 2624 a (vagum); Wall.cat. 2629 a (pubescens); Wall.cat. 2629 b (pubescens).

Hick, P.M.L. 22 (elaeagnifolium); 30 (elaeagnifolium); 33 (elaeagnifolium).

Hicks, J.R. 88 (jamaicense).

Hieronymus, G. 288 (elaeagnifolium); 915 (sisymbriifolium).

Higgins, L.C. 14434 (elaeagnifolium); 20971 (elaeagnifolium); 26756 (elaeagnifolium).

Hill, M. 225 (elaeagnifolium).

Hill, R.I. 1406 (mammosum).

Hill, S.R. 21333 (torvum).

Hincher, P. AL 100 (forskalii).

Hinds, J.H. 306 (chrysotrichum); 306 b (chrysotrichum).

Hinton, G.B. 2824 (chrysotrichum).

Hitchcock, A.S. 16624 (jamaicense); 16786 (capsicoides); 21498 (sisymbriifolium).

Hladik, A. 1373 (torvum).

Hmama, H. 890 (elaeagnifolium).

Ho, P.H. 5296 (robinsonii).

Hoan, D.T. HN-NY 1022 (torvum).

Hoare, A. 13 (lasiocarpum).

Hobby, B.M. 2574 (lasiocarpum).

Hoed, G. den 962 (torvum); 977 (barbisetum).

Hoehne, W. 5564 (torvum); 5932 (torvum); JPB 17126 (capsicoides); JPB 24261 (viarum).

Hoffman, B. 2426 (jamaicense).

Hoffmannsegg, J.C. von 119 (insanum); 137 (pseudosaponaceum).

Hofmann, U. 1763 (torvum).

Hohenacker, R.F. 36 (torvum); 36 A (torvum); 36 B (insanum); 672 a (torvum); 740 (trilobatum); 780 (virginianum); 1074 (multiflorum); 1075 (giganteum); 1076 (wightii); 1416 (violaceum); 1417 (wightii).

Holdridge, L.R. 1103 (torvum); 1835 (torvum).

Holm, R. 201 (jamaicense).

Holm, R.W. 201 (jamaicense).

Holman, R.M. 184 (melongena).

Holst, C. 382 (aethiopicum); 2031 (giganteum); 9031 (giganteum).

Holton, I.F. 556 (sisymbriifolium).

Holttum, R.E. 24715 (lasiocarpum).

Home, J.E. 1 (arundo).

Hoogland, R.D. 3334 (schefferi); 4349 (schefferi).

Hooker, J.D. 14 (aculeatissimum); 24 (melongena).

Hooker, W.J. H 2 (macrocarpon).

Hooper, D. REP-25810 (torvum); 39555 (trilobatum); 39606 (melongena).

Hoover, W.S. 30027 (torvum); 30778 (lasiocarpum); 30804 (torvum).

Hopkins, M. 897 (elaeagnifolium).

Hore, D.K. ANC-8823 (torvum).

Hormia, K. 2209 (sisymbriifolium).

Hornby, A.J. 2138 (arundo).

Horr, W.H. 3521 (elaeagnifolium).

Horreüs de Haas, R.H. 512 (virginianum).

Horsfield, T. 14 (cyanocarphium); 15 (graciliflorum); 16 (violaceum); 18 (violaceum); 19 (violaceum); 20 (involucratum); 114 (insanum); 429 (violaceum); 786 (poka).

Hortman, ? 220 (jamaicense).

Hosagoudar, V.B. 96871 (multiflorum).

Hossain, ? GC 37723 (torvum).

Hosseus, C.C. 65 (elaeagnifolium); 210 (elaeagnifolium); 233 (elaeagnifolium); 475 (torvum); 500 (elaeagnifolium); 1259 (elaeagnifolium); 2007 (elaeagnifolium); 2050 (elaeagnifolium); 2097 (elaeagnifolium); 2638 (elaeagnifolium).

Hostmann, F.W.R. 220 (jamaicense).

House, P.R. 32 -1 (mammosum); 4668 (mammosum).

Houseman, F. 60 A (sisymbriifolium).

How, F.C. 70256 (pseudosaponaceum); 71437 (insanum).

Howard, R.A. 5678 (capsicoides); 8612 (torvum); 9748 (capsicoides).

Howell, J.T. 12238 (elaeagnifolium); 26563 (elaeagnifolium); 39457 (elaeagnifolium); 47231 (elaeagnifolium).

Hoyos G, S.E. 244 (jamaicense).

Hsu, C.C. 4880 (miyakojimense); 13689 (peikuoense).

Htwe, K.M. 23984 (torvum); 24059 (violaceum); 32526 (torvum); 32622 (virginianum); 32757 (torvum); 32777 (violaceum).

Hu, S.Y. 5710 (lasiocarpum); 5711 (torvum); 5728 (torvum); 9436 (melongena); 12358 (torvum); 12785 (torvum); 12901 (torvum); 19892 (melongena); 23553 (violaceum).

Huang, T.C. 15850 (peikuoense).

Hubrecht, R.C. 17 (torvum).

Huertas, M. 16 (chrysotrichum).

Huertas, M.L. 21 (jamaicense).

Hügel, A. 1655 (hovei); 2119 (hovei); 2290 (hovei); 2979 (hovei).

Huidobro, A.M.R. 324 (elaeagnifolium); 482 (sisymbriifolium); 1496 (sisymbriifolium); 1791 (sisymbriifolium); 3514 (elaeagnifolium); 3572 (sisymbriifolium).

Huk, A. 98 (virginianum).

Huklajn, A.K. CNH-2229 (arundo).

Hul, S. 3184 (robinsonii); 3211 (camranhense); 3552 (violaceum); 4774 (trilobatum).

Humbert, H. 8356 (giganteum); 18848 (insanum); 23252 (torvum); 32124 (torvum); 32125 (torvum); 32153 (torvum); 32679 (insanum).

Humbles, J.E. 6002 (sisymbriifolium).

Hunter, C. CH 13 (aculeatissimum).

Hunter, R. 104 (mammosum).

Hunziker, A.T. 20 (elaeagnifolium); 1030 (elaeagnifolium); 3252 (elaeagnifolium); 10353 (sisymbriifolium); 15924 (elaeagnifolium); 16035 (elaeagnifolium); 17347 (elaeagnifolium); 23318 (elaeagnifolium); 24860 (elaeagnifolium); 24868 (elaeagnifolium); 25089 (elaeagnifolium); 25228 (elaeagnifolium); 25356 (elaeagnifolium); 25385 (elaeagnifolium); 25395 (elaeagnifolium); 25396 (elaeagnifolium); 25403 (elaeagnifolium); 25554 (elaeagnifolium); 25936 (elaeagnifolium).

Hunziker, J.H. 66 (elaeagnifolium); 280 (elaeagnifolium); 347 (elaeagnifolium); 2534 (viarum); 4097 (elaeagnifolium).

Huq, A.M. 10456 (torvum); 10569 (virginianum); 10618 (torvum).

Hutchinson, J. 138 (torvum); 2227 (aculeatissimum); 4552 (aculeatissimum).

Hutchinson, W.I. 3969 (torvoideum).

Hutchison, P.C. 3544 (capsicoides); 3867 (sisymbriifolium).

Ibáñez, A. 7753 (torvum).

Ibarrola, T.S. 151 (elaeagnifolium); 2016 (sisymbriifolium); 2955 (elaeagnifolium).

Ichikawa, M. 722 (arundo).

Idrobo, J.M. 122 (jamaicense); 6327 (torvum); 6808 (jamaicense).

Ikabanga, D. 113 (torvum).

Illanes, A. 104 (elaeagnifolium).

Illegible 16 (insanum); 279 (insanum); 22438 a (insanum).

Imaguire, N. 662 (robustum).

Imburt, A.T. H 64 58 (mammosum).

Ináyat 22438 (insanum); NC-22439 (violaceum).

Incarville, P.N. d' 133 (insanum).

Induni, G. 11 (torvum).

Instituto de Investigaciónes Ecológicas Chiloé 502 (elaeagnifolium).

Irani, N.A. NI 2559 (hovei); NI 2619 (hovei); NI 4588 (hovei); NI 5073 (hovei).

Iriah, H.B. FHI 23085 (torvum).

Irvine, F.R. 276 (elaeagnifolium); 768 (aethiopicum); 1356 (torvum); 1754 (torvum); 2030 (wrightii); 3364 (aculeatissimum); 3605 (macrocarpon); 4686 (macrocarpon); 4854 (torvum); 5188 (torvum); 5494 (macrocarpon); 5495 (macrocarpon).

Irwin, H.S. 534 (mammosum); 10711 (sisymbriifolium); 20396 (sisymbriifolium); 20604 (viarum); 23857 (viarum); 25111 (viarum).

Isern, J. 6468 (sisymbriifolium); 8096 (elaeagnifolium).

Isles, S. NGF-34491 (lasiocarpum).

Islewas, M.C. CNH-19460 (trilobatum).

Ismail, S. 8 (violaceum); 60 (barbisetum).

Ivens, G.W. 692 (aculeatissimum); 946 (aculeatissimum).

Iwatsuki, K. 250 (trilobatum); T-8512 (torvum); T-10866 (lasiocarpum).

Iwen, F.A. 355 (elaeagnifolium).

Jaag, O. 452 (violaceum); 1209 (lasiocarpum).

Jack Mrs 1258 (giganteum).

Jackson, J.K. 1265 (giganteum); 4011 (forskalii); 4035 (forskalii); 6111 (violaceum); 6151 (barbisetum).

Jackson, S. 4 (virginianum).

Jacob, K.C. 16190 (multiflorum); 17314 (pubescens).

Jacobs, M. 7056 (pseudosaponaceum); 7797 (pseudosaponaceum).

Jacot Guillarmod, A. 7904 (chrysotrichum).

Jacquemont, V.V. 193 (violaceum); 197 (virginianum); 258 (violaceum); 304 (insanum); 408 (violaceum); 563 (hovei); 1110 (insanum); 1489 (insanum); 2315 (insanum); 2318 (virginianum); 9109 (insanum).

Jaeger, P. 8407 (aculeatissimum).

Jaeger, P.M.L. 2 (aculeatissimum).

Jafri, S.M.H. 806 (virginianum); 903 (cordatum); 3737 (elaeagnifolium).

Jain, S.K. 939 (hovei); 2277 (hovei); 2405 (hovei); 8207 (hovei).

James, S.A. SAJ 72 (lasiocarpum).

Jameson, W. 201 (sisymbriifolium); 616 (sisymbriifolium).

Jamieson 333 (insanum); 488 (virginianum).

Janaki Ammal, E.K. 1445 (torvum).

Janardhanan, K.P. 69106 (hovei); 69289 (hovei); 70102 (hovei); 72239 (hovei); 72408 (hovei); 75885 (hovei); 76061 (hovei); 76126 (hovei); 76278 (hovei); 76645 (hovei).

Jansen, J.W.A. 784 (torvum).

Jansen, P.C.M. 6036 (arundo).

Jaramillo M, R. 350 (mammosum); 384 (jamaicense); 1145 (jamaicense).

Jard Bot Tananarive 171 (insanum).

Jardim, J.G. 335 (capsicoides); 591 (torvum); 1050 (robustum); 1788 (robustum).

Jardin, D.E.S.A. 88 (aculeatissimum).

Jaru, S.K. 29183 (hovei).

Jayasuriya, A.H.M. 2039 (trilobatum); 6987 (vagum); 17104 (melongena); 17179 (insanum).

Jayaweera, D.M.A. 923 (lasiocarpum); 1045 (violaceum).

Jefford, T.G. 1937 (giganteum).

Jeffrey, C. 46 (torvum).

Jenkins, F. 249 (barbisetum); 250 (praetermissum); 252 (violaceum); 253 (praetermissum).

Jenman, G.S. 4575 (jamaicense); 5372 (jamaicense); 5373 (torvum); 5376 (torvum); 5474 (capsicoides); 8727 (aethiopicum).

Jensen, H. 332 (dunalianum).

Jensen, R. 1 (jamaicense).

Jeshoda, K. 413 (pubescens); 414 (torvum).

Jeswiet, J. 1155 (involucratum).

Jiménez B, L.C. 325 (jamaicense).

Jiménez Chimil, M. JDA-30165 (chrysotrichum); 30804 (capsicoides); 30813 (jamaicense).

Jiménez E, N.D. 1283 (jamaicense).

Jiménez Muñoz, A. 938 (jamaicense); 2824 (torvum); 2937 (chrysotrichum).

Jiménez, J. 426 (elaeagnifolium).

Jiménez, M. 648 (sisymbriifolium).

Johansson, J.T. 144 (involucratum); 419 (poka).

Johns, R.J. 8232 (lasiocarpum).

Johns, T. 86 504 (macrocarpon).

Johnson, R.L. 12-029 (elaeagnifolium).

Johnston, I.M. 2158 (elaeagnifolium).

Johnston, M.C. 256 (elaeagnifolium); 10443 (elaeagnifolium).

Jones, B.L. 152 (melongena).

Jones, G.C. 3194 (jamaicense).

Jones, M.E. 4119 (elaeagnifolium).

Jordan, H.D. 2114 (capsicoides).

Jörgensen, P. 1070 (elaeagnifolium); 2817 (sisymbriifolium); 3678 (sisymbriifolium); 11860 (robustum).

Jorgensen, P.M. 56347 (sisymbriifolium).

Joseph SAN-117010 (lasiocarpum).

Joseph, J. 10598 (wightii); 13574 (pubescens); SC-14701 (insanum); SC-17065 (insanum).

Jost, T. 236 (jamaicense); 314 (jamaicense).

Juan, G.E. 343 (melongena).

Juliani, H.R. 34 (elaeagnifolium); 35 (elaeagnifolium); 36 (elaeagnifolium); 37 (elaeagnifolium); 39 (elaeagnifolium).

Junk, W.J. 1068 (jamaicense).

Junod, H.A. 4291 (giganteum).

Juracy, A.R.M. 07 (sisymbriifolium).

Jury, S.L. 8776 (elaeagnifolium); 20869 (elaeagnifolium).

Jussel, M.S. 69 (elaeagnifolium).

K'tung 78 5951 (violaceum).

Kadamban, D. 259 (trilobatum); 269 (torvum).

Kadhum, H. 37772 (elaeagnifolium).

Kadim, T. KN. 270 (lasiocarpum).

Kadir bin Abdul A-2834 (lasiocarpum).

Kairo, A. 43 (dunalianum); 283 (lasiocarpum); 443 (torvoideum); 10650 (lasiocarpum); NGF-30692 (lasiocarpum).

Kalkman, C. BW-3720 (dunalianum).

Kalu, A. 4172 (hovei).

Kamble, M.Y. ANC-29117 (melongena).

Kameswara Rao, N. 74 (torvum).

Kami, E. 4288 (torvum).

Kamino, L.H.Y. 814 (robustum).

Kanai, H. 63 03535 (torvum); 72601 (viarum); 72709 (torvum); 6303525 (virginianum); 6303533 (violaceum).

Kanazawa University Japan East Nepal 95-52915 (torvum).

Kandt, R. 70 (aculeatissimum).

Kanodia, K.C. 88299 (hovei); 89338 (hovei); 89795 (hovei).

Kanuri, K. 103 (arundo).

Kanywa, P.P. 30 (wrightii).

Kao, M.T. 10722 (miyakojimense).

Kapadia, Z.F. 302 (hovei); ZK 1238 (hovei); ZK 2030 c (multiflorum); ZK 2030 b (multiflorum); ZK 2030 d (multiflorum); ZK 2030 e (multiflorum); ZK 2030 a (multiflorum); ZK 2100 (hovei); ZK 2474 (hovei).

Kappler, A. 1348 (jamaicense).

Kartonegro, A. 300 (jamaicense).

Karwinski, W.H. 583 bis (elaeagnifolium); 583 (elaeagnifolium).

Kasin 160 (barbisetum).

Kassam, A. 105 (arundo).

Katende, A.B. K 66 (aethiopicum); K 97 (macrocarpon); K 1313 (giganteum); K 1603 (aethiopicum).

Katik, P. LAE 70804 (lasiocarpum); LAE-70809 (dunalianum).

Kato, M. C-5431 (poka); C-7942 (poka).

Kau, M.G. 245 (wrightii).

Kaudern, W.A. 57 (dunalianum).

Kaulback, R. 180 (torvum).

Kaur, U. UK-2 (chrysotrichum).

Kayal, R.N. Ph-597 (elaeagnifolium).

Keck, D.D. 2507 (elaeagnifolium).

Keenan, J. 946 (lasiocarpum); 1090 (torvum); 1276 (barbisetum); 1281 (torvum); 1524 (barbisetum); 1662 (barbisetum); 1723 (barbisetum); 1741 (torvum); 3828 (viarum).

Keith, H.G. 9282 (lasiocarpum).

Kellerman, W.A. 7327 (jamaicense).

Kelley, H. 21 (elaeagnifolium).

Kenfack, D. 324 (torvum).

Kennedy, J.D. 1766 (torvum); 2417 (torvum).

Kenywa, P. 30 (wrightii).

Kerber, E. 236 (chrysotrichum).

Kercher, P. AL 100 (forskalii).

Kerenga, K. LAE 56923 (dunalianum); LAE-73982 (lasiocarpum).

Kerfoot, O. 716 (aculeatissimum); 1987 (aculeatissimum); 1987 (aculeatissimum).

Kern, J.H. 8491 (involucratum).

Kernan, C. 193 (torvum).

Kerr, A.F.G. 1236 (lasiocarpum); 1237 (violaceum); 1801 (torvum); 1862 (barbisetum); 2168 (trilobatum); 2201 (insanum); 2320 (mammosum); 2370 (virginianum); 3675 (trilobatum); 3956 (torvum); 4019 (insanum); 4020 (melongena); 4064 (violaceum); 4220 (lasiocarpum); 4707 (capsicoides); 5115 (mammosum); 9048 (violaceum); 10780 (involucratum); 11039 (violaceum); 14751 (violaceum); 15151 (wrightii); 15820 (torvum); 16141 (insanum); 16210 (procumbens); 18226 (violaceum); 18641 (capsicoides); 19844 (involucratum); 20903 (praetermissum).

Kerr, F.H.W. 2051 (melongena); 2520 (torvum).

Kerr, J.G. BP-6 (robustum).

Kerr, W.S. 2346 (macrocarpon).

Kersting, O. 131 (aethiopicum).

Kessler, P.J.A. 1559 (lasiocarpum); 2952 (lasiocarpum).

Kewart, J.S. 632 (arundo).

Khalil, A. 189 (insanum).

Khan, B. 539 (lasiocarpum).

Khan, R. 736 (capsicoides); 880 (torvum); 1111 (capsicoides).

Khanna, V. 513 (virginianum).

Khattab, A. 6303 (forskalii).

Khayota, B. 71 (aculeatissimum); 570 (torvum).

Khirti, G.H. 124419 (hovei); 125235 (hovei).

Kibue, K. 103 (arundo).

Kibuwa, S.P. 2476 (arundo).

Kiesling, R. 1049 (elaeagnifolium); 3006 (elaeagnifolium); 3959 (sisymbriifolium); 4241 (elaeagnifolium); 4652 (elaeagnifolium); 4827 (elaeagnifolium).

Killip, E.P. 5076 (jamaicense); 14854 (jamaicense); 14854 (jamaicense); 26887 (jamaicense); 33303 (jamaicense); 33732 (sisymbriifolium); 35057 (jamaicense); 35203 (jamaicense).

Kimani, Z.J. 234 (arundo).

Kimeu, J. KARI 23/ 02 (aculeatissimum).

Kindeketa, W. 1264 (arundo).

King's collector 18 (violaceum); 82 (lasiocarpum); 105 (torvum); 111 (torvum); 363 (violaceum); 398 (violaceum); 454 (violaceum); 539 (lasiocarpum); 1018 (virginianum); 1022 (lasiocarpum).

King, D.O. 148 (elaeagnifolium).

King, R.A. 145 (forskalii).

King, R.M. 9161 (sisymbriifolium).

Kingdon-Ward, F. 18635 (sisymbriifolium); 18879 (violaceum); 21211 (kachinense).

Kirika, P. P.K 83 (aculeatissimum).

Kirkbride Jr, J.H. 165 (jamaicense).

Kirui, A. 29 (arundo).

Kishler, J. 145 (elaeagnifolium); 238 (elaeagnifolium); 579 (elaeagnifolium).

Kjellberg, G. 399 (lasiocarpum); 745 (lasiocarpum); 1707 (lasiocarpum); 2525 (lasiocarpum).

Klackenberg, J. 1047 (praetermissum).

Klaine, T.J. 2351 (aethiopicum).

Klappa, S. 83 (lasiocarpum); 85 (torvum).

Klein, R.M. 4025 (capsicoides).

Klingen, A.I. 1304 118 (virginianum).

Klitgaard, B.B. 1154 (robustum).

Kloss, C.B. 6990 (torvum).

Klug, G. 335 (jamaicense); 1204 (jamaicense).

Knapp, S. 9057 (sisymbriifolium); 9814 (sisymbriifolium); 9831 (sisymbriifolium); 9837 (robustum); IM-10078 (elaeagnifolium); IM-10106 a (violaceum); IM-10107 (aculeatissimum); IM-10109 (torvum); 10110 (violaceum); 10113 (melongena); IM-10114 (melongena); IM-10115 (melongena); IM-10116 (melongena); IM-10117 (melongena); IM-10118 (melongena); IM-10119 (melongena); IM-10121 (aethiopicum); IM-10122 (melongena); 10123 (torvum); 10124 (melongena); IM-10125 (barbisetum); IM-10126 (barbisetum); 10127 (barbisetum); 10128 (viarum); 10129 (barbisetum); 10130 (deflexicarpum); 10131 (melongena); IM-10132 (melongena); 10354 (sisymbriifolium); IM-10466 (elaeagnifolium); 10470 (elaeagnifolium); 10724 (sisymbriifolium); BSHC-37709(torvum); BSHC-37711 (aethiopicum); BSHC-37713.

Knees, S.G. 2731 (torvum).

Knight, D.H. 221 (sisymbriifolium).

Knobloch, I.W. 70 (elaeagnifolium).

Kobayashi, K. 31426 (virginianum).

Kochhar, R.K. 152547 (hovei); 153565 (hovei); 153573 (hovei); 157988 (hovei).

Koechlin, B. 11 (insanum).

Koelz, W. 1633 (virginianum); 4210 (insanum); 10727 (violaceum); 10842 (insanum); 11040 (multiflorum); 19751 (multiflorum); 19899 (insanum); 19970 (violaceum).

Kokwaro, J.O. 202 (arundo); 3445 (arundo); 4086 (arundo).

Kokwaro, J.O.; Mathenge, S.G. 2995 (aculeatissimum).

Kolberg, H. 925 (elaeagnifolium).

Kollmann, L. 3308 (capsicoides).

Komaromi, R. 20 (torvum).

Kondo, Y. 38863 (cyanocarphium).

Kones, D. 140 (aculeatissimum).

König, J.G. H iv 235 (torvum).

Koorders, S.H. 18035 (poka); 18037 (poka); 22525 B (involucratum); 22827 B (involucratum); 23333 (involucratum); 26280 (lasiocarpum); 28410 (involucratum); 28822 (involucratum); 31988 (lasiocarpum); 43213 (involucratum).

Kooy, C.W. 532 (lasiocarpum).

Koritschoner, H. 802 (aculeatissimum).

Korte, A. 5073 (viarum); 5994 (torvum).

Koster, C. BW-13829 (torvoideum); BW-13976 (lasiocarpum).

Kostermans, A.J.G.H. 32 (lasiocarpum); 435 (melongena); 609 (lasiocarpum); 962 (torvum); 1369 (torvum); 1433 (barbisetum); 1447 (lasiocarpum); 4814 (cyanocarphium); 7886 (lasiocarpum); 19055 (torvoideum); 21707 (lasiocarpum); 23178 (torvum); 26207 (giganteum).

Kotschy, K.G.T. 309 (forskalii).

Koyama, H. 49061 (trilobatum).

Koyama, T. 13357 (vagum); 15698 (insanum); 15973 (torvum); 16012 (violaceum); 16034 (chrysotrichum); 16038 (torvum); 16043 (chrysotrichum); 16059 (insanum); 16060 (torvum).

Kozera, C. 488 (aculeatissimum).

Kral, R. 43070 (sisymbriifolium).

Krapovickas, A. 2574 (sisymbriifolium); 5950 (elaeagnifolium); 25543 (robustum); 35054 (wrightii).

Kray, C.W. 156 (lasiocarpum).

Krempf, M. 1415 (insanum).

Kress, W.J. 94-3811 (torvum); 94-4434 (chrysotrichum).

Kriebel, R. 8208 (elaeagnifolium).

Krieger, L. 12042 (jamaicense).

Krishna, B. SKS 1627 (torvum).

Krishnappa, D.G. 20 (insanum); 63 (trilobatum); 68 (insanum); 70 (hovei); 80 (pubescens); 109 (violaceum); 121 (violaceum); 158 (multiflorum); 162 (elaeagnifolium); 165 (virginianum); 371 (hovei); 397 (giganteum).

Krukoff, B.A. 8044 (jamaicense).

Ku, S.-.M. 1752 (peikuoense).

Kuchar, P. 7962 (arundo); 10260 (aculeatissimum); 16860 (forskalii); 17094 (forskalii); 20268 (cordatum); 23276 C (forskalii).

Kufer, J. 33 (torvum); 77 (mammosum).

Kugel, A.R. 2080 (elaeagnifolium).

Kuhlmann, J.G. 907 (jamaicense).

Kuhn, B. 2605 (elaeagnifolium).

Kulkarni, B.G. 105670 (hovei); 106383 (hovei); 106481 (hovei); 108582 (hovei); 118911 (hovei); 119422 (hovei); 119542 (hovei); 120490 (hovei); 121005 (hovei); 121319 (hovei); 121456 (hovei); 128798 (hovei); 128883 (hovei); 131746 (hovei).

Kumar, A. CNH-15565 (insanum).

Kumar, S. ANC-26305 (torvum); ANC-26356 (torvum); ANC-26924 (virginianum); ANC-26953 (virginianum); ANC-26967 (violaceum); ANC-27623 (melongena); NC-92333 (insanum); NC-93273 (chrysotrichum).

Kumarilingam, ? 5218 (hovei).

Kummrow, R. 1163 (sisymbriifolium).

Kundu, B.C. 564 (trilobatum).

Kuntze, C.E.O. 256 (sisymbriifolium); 3578 (insanum); 3766 (procumbens); 7481 (trilobatum); 7620 (hovei).

Kuroiwa, N. 28755 (torvum); 28797 (virginianum).

Kurtz, F. 209 (elaeagnifolium); 911 (elaeagnifolium); 2604 (elaeagnifolium); 4222 (elaeagnifolium); 4224 (elaeagnifolium); 4744 (elaeagnifolium); 5117 (elaeagnifolium); 5209 (elaeagnifolium); 7901 (elaeagnifolium); 7993 (elaeagnifolium); 8034 (elaeagnifolium); 8086 (elaeagnifolium); 8945 (elaeagnifolium); 9503 (elaeagnifolium); 13117 (elaeagnifolium); 14206 (elaeagnifolium); 14406 (elaeagnifolium); 15732 (elaeagnifolium); 16036 (elaeagnifolium); 16090 (elaeagnifolium); 16090 a (elaeagnifolium).

Kurz, S. 202 (lasiocarpum); 204 (lasiocarpum); 2267 (lasiocarpum); 2268 (barbisetum); 2648 (virginianum).

Kusserow, M. K 67 (arundo).

Kvist, L.P. 379 (jamaicense); 379 (jamaicense); 40469 (jamaicense); 40487 (mammosum).

Kwangue, A. 5 (torvum).

la Barrera, J.M. de 18 (elaeagnifolium); 20 (elaeagnifolium).

La Croix, J.F. 2380 (torvum).

la Seta, A.V. de 255 (elaeagnifolium).

la Torre, C. de 47 (elaeagnifolium).

Lacaita, C.C. H IV 234 (violaceum).

Lace, J.H. 1710 (virginianum); 2808 (violaceum); 2809 (torvum); 3242 (torvum); 3278 (violaceum); 3672 (cordatum); 3969 (virginianum); 3972 (cordatum).

Ladell, W.R.S. 213 (torvum); 260 (torvum).

Lahaie, F. de 1765 (violaceum); 2384 (pseudosaponaceum); 2400 (trilobatum).

Lai, C.L. 392 (insanum).

Lai, P. NC-78916 (violaceum).

Lajtha, K. 123 (elaeagnifolium).

Lakra, G.S. ANC-12677 (viarum); ANC-15574 (torvum); ANC-28053 (torvum).

Lal, P. NC-63254 (insanum).

Lam, H.J. 636 (lasiocarpum); 2772 (poka).

Lambach, P.M. 1268 (lasiocarpum).

Lamei, P. LAE-89467 (torvum).

Lamond, J.M. 1353 (cordatum); 1399 (elaeagnifolium).

Lamont, J. 494 (violaceum); 495 (insanum); 496 (lasiocarpum).

Landrum, L.R. 9537 (elaeagnifolium).

Lanfranchi, A.E. 35 (elaeagnifolium); 1315 (elaeagnifolium).

Langlassé, E. 69 (jamaicense).

Langman, I.K. 2915 (elaeagnifolium).

Lanna, J.P. 1848 (torvum).

Lansdell, K.A. 854 (aculeatissimum).

Larsen, K. 1614 (trilobatum); 10520 (barbisetum); 33899 (barbisetum); 45465 (procumbens).

Latilo, M.G. FHI-43388 (aethiopicum).

Lau, S.K. 17 (procumbens); 114 (insanum); 157 (pseudosaponaceum); 163 (lasiocarpum); 685 (violaceum); 1029 (insanum); 1029 (insanum); 1154 (nienkui); 1397 (nienkui); 2828 (insanum); 2970 (nienkui); 3446 (procumbens); 3468 (nienkui); 3646 (insanum); 3659 (lasiocarpum); 4392 (capsicoides); 4770 (capsicoides); 5024 (nienkui); 5095 (insanum); 5138 (procumbens); 5339 (pseudosaponaceum); 6011 (insanum); 6214 (pseudosaponaceum); 6285 (nienkui); 25547 (pseudosaponaceum); 25849 (lasiocarpum); 27093 (nienkui); 27479 (nienkui); 27683 (nienkui); 28189 (melongena); 28327 (melongena).

Laughlin, R.M. 208 (torvum).

Laukkonen, P. 333 (elaeagnifolium).

Lavranos, J.J. 23199 (forskalii); 23276 Ca (forskalii).

Lawrence, E. 100 (aculeatissimum).

Laws, S. 34 (torvum); 61 (torvum).

Lawton, R.M. 2402 (cordatum).

LB 11864 (torvum).

Le Cong Kiet 94 (camranhense).

Le Guillou, E.J.F. 37 (insanum).

Le Testu, G. 3863 (aculeatissimum); 8021 (giganteum).

Leakey, D.G.B. 1223 (arundo).

Lebrun, J.P. 4746 (aculeatissimum).

Lecomte, H. 78 (violaceum); 1426 (insanum); 1640 (lasiocarpum); 2092 (insanum).

Ledermann, C.L. 6898 (lasiocarpum).

Ledesma Corral, C. JDA-20166 (chrysotrichum).

Lee, Y.K. 01142 (praetermissum).

Leeuwenberg, A.J.M. 1718 (torvum); 2170 (aculeatissimum); 4601 (torvum); 5104 (torvum); 5727 (wrightii); 6608 (torvum); 7409 (torvum); 8532 (torvum); 9176 (torvum); 10235 (aculeatissimum); 11400 (torvum); 12547 (torvum).

Lefèvre, E. 13 (insanum).

Legler, B. 10779 (elaeagnifolium).

Legname, V. 221 (elaeagnifolium).

Lehmann, B.T. 486 (jamaicense).

Lehmann, F.C. 226 (mammosum); 1924 (jamaicense); 4729 (mammosum); 4942 (jamaicense).

Lei, C.I. 277 (procumbens); 392 (insanum); 413 (violaceum); 1259 (nienkui); 1299 (nienkui); 1390 (nienkui).

Leippert, H. 5010 (arundo).

Leitão Filho, H.F. 11707 (robustum); 33091 (capsicoides); 34550 (torvum); 34556 (torvum).

Leiva G, S. 1287 (sisymbriifolium).

Lelean, Y. LAE 52534 (lasiocarpum).

Lemos Fróes, R. 19929 (aethiopicum); 21066 (jamaicense); 21068 (mammosum).

Lemos, J.R. 16 (jamaicense).

Lent, R. 3259 (jamaicense).

León 8782 (elaeagnifolium).

León, H. 87 (jamaicense); 211 (jamaicense).

Leonard, A. 2661 (aculeatissimum).

Leonard, E.C. 9642 (torvum).

Leonard, J. 88 (viarum).

Leonard, S.W. 3394 (elaeagnifolium).

Lépiz, E. 436 (jamaicense).

Leschenault de la Tour, J.B.L. 163 (multiflorum); 189 (pubescens); 822 (insanum); 855 (virginianum).

Lester, R.N. 39 (torvum); 46 (aethiopicum).

Letty, C. 318 (sisymbriifolium).

Leu, W.-.P. 1026 (violaceum); 2118 (retrorsum); 2164 (miyakojimense).

Leuenberger, B.E. 4459 (elaeagnifolium).

Levine, C.O. 375 (torvum); 609 (torvum); 3257 (lasiocarpum).

Lewis, M.A. 35089 (sisymbriifolium); 35090 (sisymbriifolium); 37248 (sisymbriifolium); 40501 (sisymbriifolium); 88102 (sisymbriifolium).

Lewis, W.H. 10098 (mammosum); 10747 (mammosum).

Lewis, W.L. 7674 (torvum).

Li Yanhui 1079 (lasiocarpum); 1699 (lasiocarpum); 2578 (praetermissum); 2668 (praetermissum); 3860 (barbisetum); 4232 (barbisetum); 4835 (lasiocarpum); 12316 (praetermissum); 12594 (barbisetum).

Li, H. 13382 (aculeatissimum); 13383 (violaceum); 14067 (aethiopicum).

Liane, ? 3601 (torvum).

Liang, H.Y. 61510 (procumbens); 61698 (pseudosaponaceum); 62209 (nienkui); 62400 (pseudosaponaceum); 62477 (procumbens); 63040 (nienkui); 63797 (pseudosaponaceum); 64482 (procumbens); 64626 (procumbens); 66186 (insanum); 66539 (nienkui); 66558 (procumbens).

Lichy, R. 18 (cyanocarphium); 45 (insanum).

Liebenberg, L.C.C. 3446 (sisymbriifolium); 8394 (sisymbriifolium).

Liede, S. 2834 (insanum).

Liesner, R.L. 2021 (torvum); 13615 (wrightii).

Lilljekvist, R. 204 (torvum).

Lin, C.-H. 405 (peikuoense).

Lin, H.-W. 938 (peikuoense).

Lin, Y.-R. 134 (peikuoense).

Lindeman, J.C. 2533 (sisymbriifolium).

Lindheimer, F.J. fasc. I 135 (elaeagnifolium); fasc. III 135 (elaeagnifolium); 667 (elaeagnifolium); 1041 (elaeagnifolium).

Liogier, A.H. 9024-60 (capsicoides); 9024-77 (torvum); 9732 (elaeagnifolium).

Lisbôa, M.S. 283 (torvum).

Lister, J.L. 194 (lasiocarpum); 203 (torvum).

Lister, J.R.A. 137 (mammosum).

Little, E.L. 9883 (chrysotrichum); 25731 (torvum); 26101 (torvum).

Liu, T.-.Y. 427 (peikuoense); 1369 (peikuoense).

Liu, Y.C. 540 (pseudosaponaceum).

Llanos 259 (lasiocarpum).

Llanos, A.C. 14 (chrysotrichum).

Llatas Quiroz, S. 827 (chrysotrichum); 2269 (capsicoides); 3827 (chrysotrichum).

Lleras, E. P 17032 (jamaicense).

Lloyd, R.N. 1080 (torvum).

Loher, A. (torvoideum); 4366 (torvoideum); 4367 (torvoideum); 4368 (insanum); 4369 (lasiocarpum); 4370 (lasiocarpum); 4371 (lasiocarpum); 4372 (torvoideum); 4373 (trilobatum); 4378 (retrorsum); 4379 (retrorsum); 6644 (retrorsum).

Lombardi, J.A. 938 (sisymbriifolium); 5023 (viarum); 5066 (robustum); 5287 (robustum); 5707 (robustum).

Londhe, A.N. 170566 (hovei).

Long, S. 1100 (elaeagnifolium).

López F, R.M. 6335 (elaeagnifolium).

López Francisco, O. 74166 (capsicoides).

López J, V. 104 (mammosum).

López, ? 713 (elaeagnifolium).

López, A.C. 583 (jamaicense).

López, J.O. 164 (torvum).

López, M. 10 (mammosum).

Lord, J.K. 2 (forskalii); 37 (forskalii).

Lorence, D.H. 508 (melongena); 3246 (chrysotrichum).

Lorentz, P.G. 9 (elaeagnifolium); 73 (sisymbriifolium); 98 (elaeagnifolium); 99 (elaeagnifolium); 106 (elaeagnifolium); 320 (elaeagnifolium); R 321 (sisymbriifolium); 322 (sisymbriifolium); 362 (sisymbriifolium); 533 (elaeagnifolium); 905 (elaeagnifolium); 908 (elaeagnifolium); 1072 (sisymbriifolium); 1243 (elaeagnifolium).

Lörzing, J.A. 4425 (lasiocarpum); 5494 (lasiocarpum); 11630 (violaceum); 11878 (lasiocarpum); 13254 (melongena); 15014 (lasiocarpum).

Lossen, W. 12 (elaeagnifolium).

Lott, E.J. 73 (elaeagnifolium); 5121 (elaeagnifolium); 5573 (elaeagnifolium).

Louis, A.M. 129 (torvum); 364 (torvum); 4092 (wrightii).

Louis, J.L.P. 143 (viarum); 173 (viarum); 1280 (giganteum).

Lourteig, A. 1019 (elaeagnifolium).

Lousley, J.E. W/ 819 (chrysotrichum); W/ 3382 (sisymbriifolium).

Louw, W.J. 1618 (sisymbriifolium).

Lovett, J.C. 1318 (melongena); 1727 (aculeatissimum); 2322 (giganteum).

Lowe, J. 3241 (torvum).

Lowrie, S.R. 382 (viarum); 622 (mammosum).

Lowy C, P.D. 417 (torvum).

Lozano C, G. 474 (jamaicense); 532 (jamaicense); 1776 (jamaicense); 5166 (jamaicense); 5315 (jamaicense); 5637 (jamaicense).

Lozano, R. 2051 (sisymbriifolium); 3169 (sisymbriifolium).

Lu, F.Y. 679 (insanum).

Lu, P.-.F. 12541 (peikuoense); 16536 (peikuoense).

Luchetti, A.M. 4 (elaeagnifolium); 5 (elaeagnifolium); 6 (elaeagnifolium).

Luchman, A. 7252 (wrightii).

Ludanga, R.I. R 86 (giganteum).

Ludlow, F. 7244 (torvum).

Lugas, L. 367 (lasiocarpum); 546 (lasiocarpum); 682 (lasiocarpum); 1185 (lasiocarpum); 2646 (lasiocarpum).

Luke, A. 87 (viarum).

Luke, Q..

Luke, W.R.Q. 676 (aculeatissimum); 3892 (aculeatissimum); 6859 (aculeatissimum); 7708 (giganteum); 7959 (giganteum); 9041 (sisymbriifolium); 10411 Z (torvum).

Luna, F.E. 1021 (elaeagnifolium).

Lundell, C.L. 5145 (elaeagnifolium); 6672 (jamaicense); 8784 (elaeagnifolium); 16400 (jamaicense).

Lundstrom, G. 24 (aculeatissimum).

Lunt, W. 129 (pubescens); 164 (melongena); 220 (pubescens).

Lurvey, E. 227 (sisymbriifolium).

Luteyn, J.L. 728 (chrysotrichum); 11251 (sisymbriifolium).

Luteyn, J.L.; Foster, R. 1535 (jamaicense).

Ly, V. 953 (involucratum).

Lye, K.A. 1953 (giganteum).

Maas Geesternanus, R.A. 5608 (aculeatissimum).

MacDaniels, L.H. 703 (elaeagnifolium).

MacDonald, J.R. 7551 (elaeagnifolium).

Macedo, A. 3038 (sisymbriifolium).

Macêdo, M. 1828 (sisymbriifolium).

MacGregor, R.W. 580 (melongena); 581 (violaceum); 585 (violaceum); 587 (violaceum); 1063 (violaceum).

MacGregor, W.D. 571 (torvum).

Macha, E.S. 376 (wrightii).

Machado Gomes, B. 536 (sisymbriifolium).

Maciel, J.R. 111 (capsicoides).

MacKee, H.S. 24079 (melongena); 31708 (mammosum).

Maclagan, G.J. 113 (insanum).

MacOwan, P. 1424 (aculeatissimum); 1460 (giganteum).

Macrae, J. 107 (trilobatum); 634 (insanum).

Macuacua, L. 1481 (aculeatissimum).

Madani, L. SAN 90973 (lasiocarpum); SAN 91102 (lasiocarpum).

Madras Herbarium 9604 (multiflorum); 10350 (trilobatum); 12247 (vagum); 13207 (vagum); 13338 (pubescens); 13609 (multiflorum); 14331 (pubescens); 16772 (vagum).

Madrigal, B. 582 (jamaicense); 816 (jamaicense).

Madsen, J.E. 50240 (sisymbriifolium).

Madulid, D.A. 3115 (pseudosaponaceum); 8146 (torvum).

Maesen, L.J.G. van der 672 (pubescens); 737 (violaceum); 922 (chrysotrichum); 1981 (violaceum); 2693 [a] (insanum); 2693 [b] (melongena); 2702 (torvum); 2705 (violaceum); 3131 (virginianum); 3235 (pubescens); 3276 (violaceum); 3289 (insanum); 4035 (violaceum); 5556 (torvum); 5944 (torvum).

Magalhães, M. 1129 (robustum); 2393 (robustum).

Magenta, M.A.G. 35 (robustum); 123 (torvum).

Magogo, F.C. 367 (aculeatissimum).

Mahajan, S.D. 6933 (hovei); 13222 (hovei); 24723 (hovei); 27196 (hovei).

Mahyar, U.W. 950 (lasiocarpum).

Maikin, ? SAN 101847 (lasiocarpum).

Maingay, A.C. 398 (melongena); 1158 (cyanocarphium); 1776 (torvum).

Maire, E.E. 7416 (violaceum).

Maitland, T.D. 88 (torvum); 183 (torvum); 281 (torvum); 681 (aculeatissimum); 1745 (aculeatissimum).

Majumdar, S.C. 192892 (hovei).

Makin, M.J. 25 (arundo).

Makings, E. 1102 (elaeagnifolium).

Maknoi, C. 889 (barbisetum).

Malcomber, S.T. 1722 (torvum).

Malhotra, B.K. NC-18518 (chrysotrichum); NC-27684 (violaceum).

Malhotra, C.L. NC-12454 (chrysotrichum); NC-12457 (chrysotrichum); NC-36843 (chrysotrichum); NC-37197 (chrysotrichum); NC-42787 (violaceum); 50949 (insanum); 50959 (insanum); NC-77727 (violaceum).

Malhotra, S.K. NC-15410 (chrysotrichum); NC-17227 (chrysotrichum); NC-19132 (chrysotrichum); NC-22736 (chrysotrichum); NC-22764 (violaceum); NC-23458 (chrysotrichum); NC-23726 (violaceum); NC-23856 (violaceum); NC-23986 (chrysotrichum); NC-25671 (violaceum); NC-27101 (insanum); NC-31207 (chrysotrichum).

Malik, K.A. 970 (virginianum).

Maliwanang, E. 229 (torvoideum).

Malvarez, M.R. 75 (sisymbriifolium).

Man, H. 85157 (torvum).

Man, L.S. 52114 (torvum); 55002 (torvum); 55208 (torvum); 87170 (torvum); 87438 (viarum); 87540 (violaceum); 87983 (viarum); 87986 (torvum); 88072 (torvum); 92848 (torvum); 92883 (violaceum); 96161 (viarum); 96248 (violaceum).

Mandal, N.R. BSHC-13356 (violaceum).

Mandel, N.R. 9345 (trilobatum).

Mandon, E. 1010 (elaeagnifolium).

Mann, G. 55 (torvum); 1110 (capsicoides).

Manning, S.D. 155 (torvum); 1170 (torvum).

Manning, W.E. 531244 (elaeagnifolium).

Manríque, ? 1255 (elaeagnifolium).

Marais, W. 28703 (elaeagnifolium).

Marcan, A. 31 (torvum); 67 (melongena); 68 (lasiocarpum); 73 (trilobatum); 192 (involucratum); 284 (violaceum); 702 (wrightii); 2125 (melongena).

Marche, A. 187 (insanum).

Marchetti, D. 13 (aculeatissimum).

Marcus, E.J. 26245 (elaeagnifolium).

Margwe, B. 16 (aculeatissimum).

Maries, C. 34 (virginianum); 277 (torvum).

Marín, C. 2137 (jamaicense).

Marín, R. 150 (elaeagnifolium).

Marin, R.A. MH64-16 (torvum).

Marineros, L. 127 (torvum).

Marinho, J.S. 14562 (robustum).

Marino, G. 201 (elaeagnifolium); 1914 (elaeagnifolium); 1942 (elaeagnifolium); 2047 (elaeagnifolium).

Marino, L. 17 (viarum).

Marriot, M.V. 86 (torvum).

Marsh, E.G. 895 (elaeagnifolium).

Marshall, S.A. 6359 (torvum); 6521 (torvum).

Martin, H.C. 272 (elaeagnifolium).

Martín, J. 67 (torvum).

Martin, M. 1383 (pseudosaponaceum).

Martin, R.T. 1010 (jamaicense).

Martínez Calderón, G. 1542 (wrightii); 1783 (mammosum).

Martínez Marín, J.L. 416 (elaeagnifolium); 464 (elaeagnifolium).

Martínez O, E. 413 (elaeagnifolium).

Martínez R, C. 104 (elaeagnifolium).

Martínez S, E.M. 11573 (torvum); 20379 (chrysotrichum); 20453 (chrysotrichum); 39643 (elaeagnifolium); 39673 (elaeagnifolium); 39711 (elaeagnifolium); 40416 (elaeagnifolium); 40719 (elaeagnifolium).

Martínez, C. 1796 (elaeagnifolium).

Martínez, G.J. 314 (elaeagnifolium); 486 (elaeagnifolium); 524 (elaeagnifolium); 1321 (elaeagnifolium).

Martínez, J.L. 683 (elaeagnifolium).

Martínez, M. 6827 (elaeagnifolium).

Martius, C.F.P. 251 (capsicoides); 1249 (sisymbriifolium).

Maser, R. 6 (torvum).

Mason, H.L. 8256 (elaeagnifolium); 13097 (elaeagnifolium).

Matesevach, A.M. 3 (elaeagnifolium).

Mathenge, S.G. 185 (arundo); 189 (arundo).

Mathew, B. 6553 (forskalii); 6796 (forskalii).

Mathews, A. 238 (elaeagnifolium); 3250 (sisymbriifolium).

Mathey, A. 172 (torvum); 184 (torvum).

Matthew, K.M. 8323 (trilobatum); RHT-12652 (trilobatum); 16383 (violaceum); 16401 (vagum); RHT 19640 (pubescens); RHT-20367 (violaceum); RHT 24704 (trilobatum); 28443 (pubescens); 40094 (multiflorum); 40301 (multiflorum); RHT-41189 (torvum); RHT-41805 (violaceum); RHT 43220 (trilobatum); RHT 51614 (pubescens); 51696 (giganteum); RHT-53878 (virginianum).

Mattos Silva, L.A. 4038 (torvum).

Matuda, E. 3633 (jamaicense); 18953 (elaeagnifolium); 19545 (elaeagnifolium); 28754 (elaeagnifolium).

Maturo, H. 2 (robustum).

Maung, L.S. 24373 (torvum).

Mawi, F. 204 (arundo).

Maxon, W.R. 6737 (jamaicense); 7271 (torvum).

Maxwell, J.F. 95-353 (lasiocarpum); 02-360 (torvum); 06-373 (barbisetum); 05-378 (barbisetum); 91-385 (violaceum); 92-446 (barbisetum); 98-564 (barbisetum); 91-631 (barbisetum); 74-702 (involucratum); 89-729 (barbisetum); 95-773 (barbisetum); 89-784 (torvum); 90-813 (barbisetum); 97-886 (barbisetum); 89-946 (barbisetum).

Maxwell, R.H. 988 (macrocarpon); 995 (insanum).

Mbailwa, ? 82 (aethiopicum).

Mbani, J.M. 41 (torvum); 51 (torvum).

McCann, C. 2002 (hovei); 2006 (hovei); 22451 (hovei); 22453 (hovei); CS 50504 (multiflorum).

McClain, W. 537 (torvum).

McClatchey, W. WCM 3145 (violaceum); WCM 3151 (lasiocarpum).

McClatchie, A.J. 1282 (elaeagnifolium).

McClean, A.P.D. 55 (aculeatissimum).

McClelland, D. 562 (torvum).

McClure, F.A. 7641 (insanum); 8383 (lasiocarpum); 8833 (procumbens); 9518 (pseudosaponaceum).

McDaniel, S. 16535 (mammosum); 17693 (jamaicense).

McDaniel, S.T. 11278 (sisymbriifolium).

McDonagh, J. 80 (jamaicense); 404 (jamaicense).

McDonald, ? 4122 (lasiocarpum); 4479 (insanum).

McDowell, T. 121 (jamaicense); 350 (jamaicense); 3788 (jamaicense).

McGregor, E.A. 857 (elaeagnifolium).

McGregor, R.C. 8525 (pseudosaponaceum); 19741 (pseudosaponaceum); 20128 (retrorsum).

McLeish, I.M. 578 (cordatum); 737 (cordatum); 1498 (cordatum); 2284 (cordatum); 2315 (cordatum).

McMillen, H.L. 2 (robustum).

McVaugh, R. 10111 (chrysotrichum).

Mearns, E.A. 122 (elaeagnifolium); 135 (elaeagnifolium); 619 (elaeagnifolium); 1698 (elaeagnifolium); 2496 (giganteum).

Medina C, M. 2036 (elaeagnifolium).

Medina, V. 241 (elaeagnifolium).

Medina, W.A. 57 (elaeagnifolium).

Medley Wood, J. 4515 (aculeatissimum); 5644 (aculeatissimum).

Meebold, A. 7661 (mammosum); 7664 (lasiocarpum); 7743 (violaceum); 10029 (trilobatum); 15084 (mammosum); 16683 (barbisetum).

Meeboonya, R. RM 242 (trilobatum); RM 243 (trilobatum); RM 244 (trilobatum); RM 245 (trilobatum); RM 248 (melongena); RM 249 (torvum); RM 250 (melongena); RM 251 (melongena); RM 257 (melongena); RM 266 (melongena); RM 267 (torvum); RM 271 (wrightii); RM 272 (lasiocarpum); RM 274 (torvum); RM 277 (melongena); RM 287 (lasiocarpum); RM 294 (melongena); RM 295 (melongena); RM 296 (torvum); RM 301 (trilobatum).

Meglioli, S. 33 (elaeagnifolium).

Meikle, R.D. 1272 (torvum).

Mejía, M. I0286 (torvum); 103 ll (capsicoides); 7521 (torvum); 7957 (jamaicense); 8104 (torvum); 8293 (capsicoides); 10008 (torvum); 11056 (torvum).

Melo, E. 3047 (torvum); 4793 (mammosum); 7748 (sisymbriifolium); 7838 (sisymbriifolium).

Melville, F.A. 24 (torvum).

Méndez, R. 26 (jamaicense).

Mendonça, R.C. 35 (sisymbriifolium).

Mendoza, D. 10718 (torvoideum).

Mendum, M. 99-264 (retrorsum).

Mentz, L.A. 219 (aculeatissimum).

Merchant, Y.A. 635 (hovei); 1179 (hovei).

Mercier, J. VII.51 (giganteum).

Mereles, F. 2363 (robustum); 2481 (sisymbriifolium); 4010 (elaeagnifolium); 5109 (elaeagnifolium); 9095 (elaeagnifolium).

Mericle, L.W. 500 (elaeagnifolium).

Merrill, E.D. 345 (insanum); 465 (lasiocarpum); 623 (melongena); 856 (torvoideum); 938 (insanum); 1736 (torvoideum); 1736 (torvoideum); 3406 (torvum); 4237 (retrorsum); 4807 (pseudosaponaceum); 7754 (retrorsum); 9665 (retrorsum).

Merritt, M.L. 12038 (lasiocarpum).

Mertz, S.M. 15 (elaeagnifolium).

Mesfin Tadese 116 (giganteum); 452 (giganteum); 5295 (giganteum); 6827 (giganteum).

Metcalf, F.P. 7423 (capsicoides); 7647 (chrysotrichum).

Metcalf, R.D. 30806 (sisymbriifolium).

Metz, M.C. 3164 (elaeagnifolium).

Mexia, Y. 1757 (chrysotrichum); 4157 (viarum); 4375 (sisymbriifolium); 4466 (viarum).

Meyer, A.B. 43 (pseudosaponaceum).

Meyer, F.G. 7766 (giganteum); 7790 (macrocarpon); 8633 (giganteum); 8762 (macrocarpon).

Meyer, R. 2233 (lasiocarpum); 2285 (torvoideum).

Meyer, T. 565 (elaeagnifolium); 3506 (elaeagnifolium); 4046 (elaeagnifolium); 4067 (elaeagnifolium); 9863 (elaeagnifolium).

Mgaza, C.D. 27 (giganteum).

Mhoro, B. 5694 (wrightii).

Miehe, G. 277 (elaeagnifolium).

Miers, J. 12581 (elaeagnifolium).

Migeod, F.W.H. 241 (torvum); 394 (aculeatissimum).

Mikage, M. 95-52940 (violaceum).

Mildbraed, G.W.J. 9507 (giganteum); 9517 (torvum).

Millar, A.N. NGF-15605 (lasiocarpum).

Mille, L. 974 (jamaicense).

Miller, A.G. 3146 (cordatum); 3494 (forskalii); 6341 (cordatum); 7157 (cordatum).

Miller, J.S. 3371 (torvum); 3371 (torvum); 8638 (arundo).

Milliken, W. 1058 (lasiocarpum).

Millspaugh, C.F. 3506 (torvum).

Milne-Redhead, E.W. 7153 (arundo); 7598 (giganteum).

Minaki, M. 90-80025 (violaceum).

Miranda, F. 1418 (elaeagnifolium).

Misra, O.P. NC-38288 (violaceum); CNH-41463 (insanum); 41608 (insanum); NC-44313 (insanum); 44661 (insanum); NC-47295 (insanum).

Mitchell, B.A. 17 (involucratum).

Mitchell, E.R. 84 (torvum).

Mizoguchi, K. 952 (sisymbriifolium).

Mlangwa, J.A. 1606 (giganteum); 1769 (giganteum).

Mobbs, R.H. p 182 (violaceum).

Mogg, A.O.D. 12455 (giganteum).

Mohamedi, Y. 9105 (robustum).

Mohan, A. 13431 (viarum).

Mohanan, C.N. 55516 (multiflorum); SC-72093 (capsicoides); SC-77917 (capsicoides).

Mohr, C. 7 (torvum); 1094 (torvum).

Moi, W. 188 (torvoideum).

Mokim, S. 1 (trilobatum); 623 (capsicoides); 1239 (virginianum); 1594 (melongena).

Molina R, A. 551 (torvum); 1979 (jamaicense); 2846 (jamaicense); 5537 (jamaicense); 5686 (jamaicense); 6235 (torvum); 6539 (chrysotrichum); 6761 (torvum); 6902 (jamaicense); 7070 (torvum); 7087 (jamaicense); 7320 (jamaicense); 8155 (jamaicense); 8317 (jamaicense); 8364 (jamaicense); 8457 (jamaicense); 10297 (jamaicense); 10959 (jamaicense); 11706 (jamaicense); 11813 (jamaicense); 13011 (jamaicense); 13376 (torvum); 17383 (jamaicense); 17729 (jamaicense); 34413 (wrightii).

Molina, A.M. 505 (elaeagnifolium); 993 (elaeagnifolium); 1108 (elaeagnifolium); 1265 (elaeagnifolium); 1305 (elaeagnifolium); 1352 (elaeagnifolium); 2220 (elaeagnifolium); 2644 (elaeagnifolium); 2723 (elaeagnifolium); 4048 (elaeagnifolium).

Molina, D. 18 (torvum).

Molinar, R. 47 (elaeagnifolium).

Moll, E.J. 3452 (aculeatissimum).

Moncada, F. 4741 (elaeagnifolium).

Mondragón, M.E. 142 (wrightii).

Monod, T. 302 (forskalii); 1608 (forskalii); 1609 (forskalii); 17095 (pubescens).

Monro, A.K. 2317 (wrightii); 2776 (torvum); 2777 (wrightii); 3999 (sisymbriifolium); 6413 (viarum); 6454 (torvum); 6497 (torvum).

Monteiro, O.P. INPA 53382 (jamaicense).

Montenegro, M.A. 380 (elaeagnifolium).

Montero Castro, J.C. 136 (elaeagnifolium).

Monterrosa, J. 1371 (chrysotrichum).

Mooers, B.H.M. 854 (elaeagnifolium).

Moon, A. 465 (giganteum).

Moon, J.K. 11 (arundo).

Mooney, H.F. 120 (torvum); 463 (violaceum); 688 (torvum); 976 (virginianum); 2482 (violaceum); 2715 (giganteum); 3066 (trilobatum); 3231 (violaceum); 3331 (violaceum); 3332 (torvum); 3934 (torvum); 5730 (giganteum); 6765 (giganteum); 8412 (giganteum).

Moore, H.E. 1640 (elaeagnifolium).

Moraes, P.L.R. 23597 (robustum).

Morales, J.F. 1718 (chrysotrichum); 3658 (torvum); 5362 (jamaicense); 6345 (mammosum).

Moran, R. 27703 (elaeagnifolium).

Morat, P. 1257 (torvum); 1393 (torvum).

Moreira, R.S. 119 (sisymbriifolium).

Morel, I. 527 (sisymbriifolium); 1786 (sisymbriifolium); 5294 (sisymbriifolium); 6147 (sisymbriifolium).

Morely, B.D. 843 (torvum).

Moreno, P.P. 92 (wrightii); 15588 (wrightii).

Mori, S. 19222 (torvum).

Mori, S.A. 51 (jamaicense); 351 (chrysotrichum); 14428 (robustum).

Morley, B.D. 843 (torvum).

Morong, T. 91 (sisymbriifolium); 102 (viarum); 1190 (elaeagnifolium).

Morrison, A. 13 (sisymbriifolium).

Morrison, K. 5 (insanum).

Morse, H.B. 281 [a] (violaceum); 281 [b] (insanum); 282 (insanum).

Morton, C.V. 3371 (torvum); 4672 (torvum); 7003 (torvum).

Morton, J.K. K 931 (torvum); SL-2691 (aculeatissimum); SL-3145 (aculeatissimum); SL-3667 (aculeatissimum); A 4193 (melongena); A 5008 (capsicoides); 6331 (torvum).

Morton, M. 12 (melongena).

Moscone, E.A. 34 (elaeagnifolium); 70 (elaeagnifolium); 71 (elaeagnifolium); 109 (elaeagnifolium); 111 (elaeagnifolium); 161 (elaeagnifolium); 180 (elaeagnifolium); 183 (elaeagnifolium); 191 (elaeagnifolium); 245 (elaeagnifolium).

Mosén, C.W.H. 1555 (sisymbriifolium).

Mosha, C.J. 1901 (arundo).

Mosheti, ? 5 (arundo).

Mosnier, M. 3786 (virginianum).

Moss, C.E. 2062 (sisymbriifolium); 8550 (sisymbriifolium).

Mota, C.D.A. 348 (jamaicense).

Mota, R.C. 326 (robustum).

Motley, J. 148 (cyanocarphium); 187 (involucratum).

Mouandza Mbembo, J.-.C. 105 (torvum).

Moura, C. 354 (torvum).

Mouret, M. 203 (violaceum); 205 (procumbens); 206 (procumbens).

Mousset, J.P. 93 (involucratum).

Mshana, H. 49 (robustum).

Muasya, T. 119 (arundo).

Muchiri, J. 592 (aculeatissimum).

Mueller-Dombois, D. 68-10210 R (insanum).

Mugambi, G.K. 776 (wrightii).

Mukerjee, S.K. 1918 (insanum); 3247 (aethiopicum).

Mukete, W. 24 (torvum).

Mukherjee, A. 150 (insanum).

Mukherjee, A.K. E-4301 (trilobatum); E-4307 (trilobatum); 5358 (trilobatum); 5934 (trilobatum).

Mukherjee, B.K. 6114 (violaceum); 6144 (viarum).

Mukhopadhyay, C.R. 428 A (insanum).

Mulford, A.I. 684 (elaeagnifolium).

Müller, F. 551 (mammosum).

Múller, P.J. 223 (aculeatissimum).

Muller, P.J. 225 (giganteum).

Muller, P.J.; Scheepers, J.C. 223 (aculeatissimum).

Müller-Hohenstein, K. 1258 (forskalii).

Mungai, W.T.F. 5 (arundo).

Muñoz E, M. 292 (sisymbriifolium).

Munroe, ? 20 (arundo).

Murata, G. B-19 (lasiocarpum); 3722 (torvum); T-15456 (insanum); T-16792 (violaceum); T-16862 (barbisetum); T-17303 (trilobatum); T-17786 (torvum); T-17787 (involucratum).

Murillo A, J.C. 1718 (jamaicense).

Murphy, H. 473 (mammosum).

Murray, G.W. 3744 (forskalii).

Murty, U.R. 1559 (chrysotrichum); 5631 (insanum).

Murugan, C. 117673 (multiflorum).

Muthuri, J. 225 (arundo).

Mutis, J.C. 3561 (mammosum); 3571 (torvum).

Mwangangi, O.M. 54 (arundo); 1553 (arundo).

Mwangoka, M.A. 128 (giganteum); 199 (giganteum); 271 (giganteum); 433 (melongena); 4486 (aculeatissimum).

Mwiga, G.T. 5 (aculeatissimum).

Mwinyjuma, I.G.S. 405 (arundo).

Myers, J.G. 11101 (aculeatissimum).

N'Kounkou, J.S. 230 (torvum).

Naattacharyya, U.C. CNH-39177 (insanum).

Nadil Khan 123 (aculeatissimum).

Naeh, M. 19403 A (virginianum).

Nafday, K.U.R. 46 (virginianum); 158 (melongena).

Nagahama, N. 129 (elaeagnifolium); 130 (elaeagnifolium); 131 (elaeagnifolium); 132 (elaeagnifolium).

Nagamasu, H. 6917 (torvum); 35557 (torvum).

Naganathan, T.R. 19407 (trilobatum).

Nair, N.C. ANC-810 (viarum); ANC-811 (violaceum); ANC-1599 (insanum); ANC-6248 (torvum); 50743 (multiflorum); 52937 (trilobatum); 52939 (pubescens); 60827 (pubescens); SC-60904 (insanum); 64365 (multiflorum); 70195 (multiflorum).

Nair, V.J. NC-23156 (insanum); 56538 (trilobatum); 67255 (multiflorum); 67370 (multiflorum).

Naithani, B.D. 23184 (trilobatum); 24666 (multiflorum).

Nanakorn, W. 88-0192 (trilobatum).

Nannan, A.C. 96 (aethiopicum).

Napier, E.R. 381 (aculeatissimum); 598 (aculeatissimum); 1950 (aculeatissimum); 2651 (arundo); 3119 (aculeatissimum); 5898 (aculeatissimum).

Napper, D.M. 510 (arundo); 748 (arundo).

Naranjo, C. 937 (elaeagnifolium).

Narasimhan, D. 624 (trilobatum).

Narasimhan, P.L. 163984 (hovei); 165976 (hovei).

Narayanaswami, V. 131 (insanum); 131 (insanum); 168 (melongena); 451 (melongena); 849 (multiflorum); 3507 (multiflorum); 3586 (insanum); 4131 (trilobatum); 4739 (multiflorum); 19021 (pubescens); 19996 (pubescens); 80789 (trilobatum).

Narvaez C, J. 2 (elaeagnifolium).

Narváez, J.M. 4 (elaeagnifolium).

Narváez, M. 157 (mammosum); 465 (torvum).

Nasir, E. 16303 (cordatum).

Native Collector CD-209 (lasiocarpum); 1056 (violaceum).

Nautujal, S. 41 (violaceum).

Nawi, R. SAN-55905 (torvum).

Nayar, M.P. 153128 (hovei).

Ndive, E. 172 (torvum).

Nedi 5 (jamaicense); 494 (dunalianum); 514 (lasiocarpum); 639 (melongena).

Nee, M. 3406 (capsicoides); 3426 (viarum); 3456 (torvum); 3520 (jamaicense); 3521 (jamaicense); 3777 (jamaicense); 3786 (jamaicense); 4173 (wrightii); 4179 (mammosum); 4295 (jamaicense); 4296 (jamaicense); 4301 (jamaicense); 11787 (elaeagnifolium); 11815 (elaeagnifolium); 15007 b (sisymbriifolium); 16097 a (sisymbriifolium); 17408 (jamaicense); 17448 (jamaicense); 17662 (jamaicense); 18771 (torvum); 18972 (elaeagnifolium); 24064 (elaeagnifolium); 25104 (jamaicense); 25319 (elaeagnifolium); 27690 (wrightii); 27691 (wrightii); 27770 (jamaicense); 27841 (mammosum); 27843 (jamaicense); 31572 (sisymbriifolium); 31621 (sisymbriifolium); 31712 (sisymbriifolium); 31761 (sisymbriifolium); 31804 (sisymbriifolium); 33904 (sisymbriifolium); 34106 (sisymbriifolium); 34373 (viarum); 34931 (jamaicense); 35563 (sisymbriifolium); 35766 (sisymbriifolium); 36280 (wrightii); 36669 (sisymbriifolium); 38513 (sisymbriifolium); 42063 (sisymbriifolium); 42368 (jamaicense); 42370 (viarum); 44209 (torvum); 44373 (sisymbriifolium); 46290 (viarum); 46313 (chrysotrichum); 46548 (sisymbriifolium); 50521 (sisymbriifolium); 51845 (sisymbriifolium); 51942 (sisymbriifolium); 54599 (aethiopicum); 54913 (sisymbriifolium); 57070 (elaeagnifolium); 57083 (elaeagnifolium).

Neill, D.A. 3024 (chrysotrichum).

Nelson, A. 1651 (elaeagnifolium); 11558 (elaeagnifolium).

Nelson, C.H. 3815 (chrysotrichum).

Nelson, E.B. 4123 (jamaicense).

Nelson, E.W. 2904 (torvum); 3029 (torvum); 6429 (elaeagnifolium).

Nepal Bajhang Expedition 209-13060 (virginianum); 209-13087 (viarum).

Nere, ? de 1174 (torvum); 1753 (torvum); 1846 (wrightii).

Neubauer, J.H.F. 1775 (virginianum).

Newberry, P.E. 205 (forskalii); 206 (forskalii); 389 (forskalii).

Newbould, J.G.B. 4596 (aculeatissimum); 6477 (aculeatissimum); 7164 (aculeatissimum); 19174 (arundo).

Newbury, R.J. 13 (torvum).

Newman, M. 315 (elaeagnifolium).

Newman, M.F. LAO-43 (torvum); 2222 (torvum).

Nezu, C.Y. V 12 (torvum).

Ngomba, J.N. 5 (torvum).

Nguyen, V.D. HNK 1332 (torvum).

Ngwang, R. 58 (torvum).

Nicholas, A. 2033 (aculeatissimum).

Nichols, G.E. 81 (torvum).

Nicholson, C.J. Godwin 49 (torvum).

Nicolson, D.H. 1868 (torvum); 2068 (capsicoides).

Nicora, E.G. 1646 (elaeagnifolium); 2101 (elaeagnifolium); 4162 (elaeagnifolium); 4189 (elaeagnifolium); 4282 (elaeagnifolium); 8114 (elaeagnifolium).

Njau, E.; Kayombo, C.J.; Festo, L. 1331 (aculeatissimum).

Nkouka, S.O.C. 764 (aethiopicum).

Nning, J. 190 (torvum).

Nobick, L.R. 2523 (jamaicense).

Noblick, L.R. 2523 (jamaicense); 2564 (capsicoides).

Noor, M. MN. 57 (insanum).

Nooteboom, H.P. 5837 (lasiocarpum).

Nordlindh, T. 4563 (giganteum).

Norul Rozimah Pg Hj Seruji 18b 3000 (torvum).

Noshiro, S. 9760025 (virginianum); 9760490 (virginianum).

Novara, L.J. 8248 (elaeagnifolium); 8504 (elaeagnifolium).

Novoa L, C.P. 919 (elaeagnifolium).

Nugent, R. 18 (forskalii).

Nunes, T.S. 1431 (torvum).

Núñez V, P. 8251 (mammosum).

Nuraliev, M.S. NUR-101 o (viarum); NUR-1863 a (putii).

Nusbaumer, L. 1021 (torvum); 1084 (torvum); 4036 (capsicoides).

Nuskar, J.D. 11 (trilobatum).

Nyakundi, D. 195 (aculeatissimum).

O'Donell, C.A. 224 (elaeagnifolium); 1712 (elaeagnifolium); 1815 (elaeagnifolium); 1910 (elaeagnifolium).

Oates, J.F. 159 (giganteum).

Obeng-Darko, K. 5027 (torvum).

Ober 300 (insanum).

Obunyali, C. EFRI 253 (arundo).

Ocampo, R. 39 (elaeagnifolium).

Odewo, T.K. FHI 67123 (torvum).

Odonne, G. 427 (mammosum).

Ohashi, H. 77 4330 (violaceum).

Oldeman, R.A.A. 23 (aculeatissimum); 90 (torvum).

Oldham, R. 338 (violaceum); 339 (insanum); 339 b (insanum); 342 (pseudosaponaceum).

Olsen, S. 424 (lasiocarpum); 773 (cyanocarphium); 1057 (cyanocarphium).

Omondi, W. KEFRI 45 (arundo).

Onana, J.-.M. 1924 (giganteum); 1925 (torvum).

Orcutt, C.R. 680 (elaeagnifolium); 840 (torvum); 1270 (elaeagnifolium); 2158 (torvum); 3158 (elaeagnifolium); 3422 (elaeagnifolium).

Orejuela, A. 153 (jamaicense); 2820 (sisymbriifolium).

Orlandi, R.P. 581 (capsicoides).

Ormonde, J. 509 (chrysotrichum).

Orozco, C.I. 609 (jamaicense); 3323 (jamaicense).

Ortega, L.C.S. 1927 (sisymbriifolium).

Ortiz, F. 456 (jamaicense).

Ortiz, R.T. 2029 (jamaicense); 2372 (jamaicense).

Osten, C. 8895 (robustum).

Osterhout, G.E. 2064 (elaeagnifolium).

Oswald B, A. 146 (violaceum).

Oswald, P.H.O. 15 (elaeagnifolium).

Otanes, F. 2071 (lasiocarpum); 17710 (retrorsum); 17776 (insanum).

Otero, J.I. 21 (melongena); 164 (torvum); 171 (capsicoides); 206 (wrightii).

Paciornik, E.F. 196 (sisymbriifolium).

Páez V, J.A. 12 (sisymbriifolium).

Paião, F.A. 303 (sisymbriifolium).

Palacios, M.A. 1695 (elaeagnifolium).

Palee, P. 988 (barbisetum).

Pallithanam, J. G 459 (hovei); 58-3436 (pubescens).

Palmer, E. 88 (torvum); 101 [b] (elaeagnifolium); 233 (elaeagnifolium); 235 (elaeagnifolium); 388 (elaeagnifolium); 935 (elaeagnifolium); 936 (elaeagnifolium); 941 (elaeagnifolium); 942 (elaeagnifolium); 2101 (elaeagnifolium).

Palmer, E.J. 7678 (elaeagnifolium).

Palms, C.S. 96 (forskalii).

Pandey, R.P. AC-7972 (insanum).

Panigrahi, G. CC-3057 (insanum); 3754 (melongena); CC-4283 (insanum); 5495 (viarum); CC-5881 (insanum); CC-6353 (insanum); 11240 (melongena); 12048 (melongena); EC-21602 (melongena); EC-23645 (insanum); EC-23792 (trilobatum); EC-23832 (trilobatum).

Panst, P.C. 43446 (insanum).

Pant, P.C. NC-37543 (chrysotrichum); NC-38551 (violaceum); NC-43404 (insanum); NC-43446 (insanum); NC-43795 (violaceum).

Panti, M.A. 841 (elaeagnifolium).

Paoli, G. 137 (arundo); 1083 (cordatum).

Pappi, A. 2953 (forskalii); 3996 (forskalii).

Parada-Gutierrez, G.A. 3228 (sisymbriifolium).

Parham, H.B.R. 1 (torvum); 24 (torvum).

Parish, S.B. 8104 (elaeagnifolium).

Parish, W.F. 172 (elaeagnifolium).

Parker, C. 7254 (violaceum).

Parker, H.M. 379 (elaeagnifolium).

Parker, R.N. 3195 (viarum); 3326 (cordatum).

Parkinson, C.E. 13980 (torvum); 14657 (torvum); 15699 (violaceum).

Parkinson, R. 10 (dunalianum).

Parmar, P.J. AC-2458 (virginianum).

Paroisse, G. 263 (melongena).

Parry, C.C. 536 (elaeagnifolium); 636 (elaeagnifolium).

Parthasarathy, N. 708 (vagum).

Paskin, M.W.J. H 13 (forskalii); H 43 (forskalii).

Pastório, F.F. 29 (torvum); 73 (torvum).

Pataskan, R.D. 102138 (hovei); WC-105834 (insanum); WC-105889 (insanum); 108875 (hovei); WC-110313 (insanum).

Patel, V.M. VP 721 (hovei); V.P. 730 (hovei); V.P. 733 (hovei); V.P. 739 (hovei); VP 769 (hovei); VP 770 (hovei); VP 771 (hovei); VP 776 (hovei); V.P. 782 (hovei); VP 796 (hovei); V.P. 797 (hovei); V.P. 798 (hovei); V.P. 799 (hovei); VP 800 (hovei); V.P. 804 (hovei); V.P. 814 (hovei); V.P. 815 (hovei); V.P. 816 (hovei); VP 817 (hovei); V.P. 818 (hovei); VP 935 (hovei); VP 936 (hovei); V.P. 1080 (hovei); V.P. 1119 (hovei); V.P. 1139 (hovei); V.P. 1140 (hovei); V.P. 1437 (hovei); V.P. 1438 (hovei); VP 1894 (multiflorum); VP 1895 (multiflorum); VP 1896 (multiflorum); VP 1897 (multiflorum); VP 1898 (multiflorum); VP 1899 (multiflorum); VP 1900 (multiflorum); VP 1901 (multiflorum); VP 1902 (multiflorum); V.P. 1951 (hovei); V.P. 1982 (hovei).

Paul, T.K. CNH-28122 (virginianum).

Paula-Souza, J. 7856 (elaeagnifolium); 8046 (elaeagnifolium).

Paulo, S. 488 (arundo).

Pawek, J. 7374 (aculeatissimum); 9949 (aculeatissimum).

Pearson, A. p 43 (insanum).

Peck, E.F. 366 (cordatum).

Pedersen, T.M. 372 (robustum); 5789 (elaeagnifolium); 5790 (elaeagnifolium); 6017 (robustum); 8235 (elaeagnifolium).

Pedraza, P. 772 (jamaicense).

Pedro, J.G. 3725 (aculeatissimum).
Pedrono, ? 6 (insanum).
Peebles, R.H. 7410 (elaeagnifolium).
Pegler, A. 142 (aculeatissimum); 709 (giganteum).
Pei Sheng-ji 9247 (barbisetum); 59-9253 (barbisetum); 59-10078 (praetermissum).
Pei, C. 2996 (violaceum).
Peláez, C. 206 (sisymbriifolium).
Peña-Chocarro, M.C. 1483 (elaeagnifolium); 1494 (robustum); 1500 (sisymbriifolium); 1873 (robustum); 1934 (sisymbriifolium).
Peng, C.-.I. 6576 (peikuoense); 16833 (peikuoense).
Peng, T.C. 2136 (pseudosaponaceum).
Penland, C.W.T. 1131 (sisymbriifolium).
Pennell, F.W. 1576 (jamaicense); 4218 (jamaicense).
Pennington, T.D. 17682 (wrightii).
Pensiero, J.F. 2424 (elaeagnifolium); 2736 (sisymbriifolium); 6063 (elaeagnifolium); 7209 (elaeagnifolium); 7374 (elaeagnifolium).
Perdue, R.E. 9474 (giganteum).
Perdue, R.E.; Kibuwa, S.P. 8332 (aculeatissimum).
Pereira, J.T. 498 (capsicoides).
Pereira-Silva, G. 2080 (sisymbriifolium); 15482 (jamaicense); 15715 (sisymbriifolium).
Pérez Arbeláez, E. 615 (sisymbriifolium); 701 (mammosum); 755 (jamaicense); 5860 (sisymbriifolium); 6410 (mammosum); 6447 (torvum); 8129 (torvum).
Pérez C, E. 3359 (elaeagnifolium).
Pérez H, S. 86 (elaeagnifolium).
Pérez M, B.R. 1197 (sisymbriifolium); 1310 (sisymbriifolium).
Pérez Moreau, R.L. 3976 (elaeagnifolium).
Pérez Viso, R. 59 (torvum); 408 (torvum); 662 (torvum); 1840 (torvum); 2064 (torvum); 3021 (aethiopicum); 4347 (torvum).
Perez, ? 439 (sisymbriifolium).
Pérez, B. 451 (robustum).
Pérez, L. 71 (sisymbriifolium); 284 (sisymbriifolium); 1394 (sisymbriifolium).
Pérez, V. 174 (sisymbriifolium).
Perianayagam, S. RHT 12022 (virginianum).
Periaswamy, K. 19791 (wightii).
Periquet, L. 203 (giganteum).
Perkins, A.E. 3377 (elaeagnifolium).
Perrier de la Bâthie, J.M.H.A. 263 (melongena).

Perrottet, G.S. 289 (trilobatum); 340 (forskalii); 558 (forskalii); 563 (forskalii); 905 (multiflorum).

Perumal, P. RHT-17881 (trilobatum); 21473 (violaceum); 21714 (torvum); RHT-22680 (trilobatum).

Pervillé, A. 266 (aethiopicum); 510 (aethiopicum).

Pételot, A. 777 (violaceum); 1279 (procumbens); 1356 (praetermissum).

Peterson, J. 075 (elaeagnifolium).

Petetín, C.A. 1054 (sisymbriifolium); 1706 (elaeagnifolium); 1798 (sisymbriifolium).

Peyre de Fabrègues, B. 1911 (forskalii).

Pfeifer, H.W. 1953 (chrysotrichum).

Pfund, J. 646 (forskalii).

Phengklai, C. 4082 (torvum); 12724 (involucratum); 12854 (involucratum).

Philcox, D. 3976 (sisymbriifolium); 10403 (trilobatum).

Philipson, W.R. 551 (torvum); 555 (torvum).

Phillips, E. 1267 (aculeatissimum); 4292 (aculeatissimum).

Phillipson, P.B. 1198 (aculeatissimum); 2805 (insanum); 3411 (insanum); 5212 (giganteum).

Phuong, V.X. HNK 314 (torvum).

Phusomsaeng, S. 36 (barbisetum).

Piccinini, B.G. 2046 (elaeagnifolium); 3115 (elaeagnifolium); 3117 (elaeagnifolium); 4144 (sisymbriifolium).

Pichi-Sermolli, R.E.G. 2576 (giganteum); 2577 (giganteum); 2578 (giganteum).

Pickel, D.B. 2253 (jamaicense).

Pierotti, S.A. 4258 (sisymbriifolium).

Pierre, L. 3 (trilobatum); 6 (lasiocarpum); 9 (trilobatum); 92 (involucratum); 200 (aethiopicum); 706 (melongena); 710 (lasiocarpum).

Pilg, I. 852 (wrightii).

Pilz, G.E. 1865 (torvum).

Pinheiro, G.S. 672 (viarum).

Pinto, D. 11 (jamaicense); 17 (mammosum).

Pinto, G.C.P. 346 /83 (jamaicense); 356 /83 (jamaicense).

Pipoly, J.J. 9114 (jamaicense); 9128 (jamaicense); 9399 (jamaicense); 9399 (jamaicense); 11314 (jamaicense).

Pires, J.M. 9486 (sisymbriifolium); 52573 (jamaicense).

Pitopang, R. 1079 (torvum).

Pittier, H.F. 62 (chrysotrichum); 3979 (jamaicense).

Pius, G. SAN-143601 (lasiocarpum).

Playfair, G.M.H. 75 (insanum); 310 (violaceum).

Pleyte, D.R. 613 (schefferi); 671 (lasiocarpum).

Plowman, T.C. 127 (jamaicense); 2085 (jamaicense); 2085 (jamaicense); 2320 (jamaicense); 2711 (elaeagnifolium); 3188 (mammosum); 4163 (jamaicense); 4236 (mammosum); 7264 (jamaicense); 7265 (jamaicense); 9060 (aethiopicum); 11043 (chrysotrichum); 12147 (jamaicense).

Plowman, T.C.; Kennedy, H. 2241 (jamaicense).

Pócs, T. 89-025 /D (aculeatissimum); 6106 G (robustum); 6461 A (robustum); 6500 A (giganteum).

Podlech, D. 17424 (virginianum).

Podzorski, A.C. 1014 (forskalii).

Poeppig, E.F. 26 (elaeagnifolium); 140 (elaeagnifolium); Diar 540 (elaeagnifolium).

Pogge, P. 1156 (aethiopicum).

Pohl, J.B.E. 3453 (robustum).

Poilane, E. 17 (lasiocarpum); 69 (insanum); ch 85 (insanum); 99 (insanum); 184 (lasiocarpum); 2777 (robinsonii); 3245 (involucratum); 3916 (violaceum); 4993 (procumbens); 4995 (procumbens); 5320 (insanum); 5770 (lasiocarpum); 5781 (nienkui); 7280 (cyanocarphium); 8151 (insanum); 8933 (nienkui); 9082 (nienkui); 9529 (lasiocarpum); 9887 (nienkui); 11816 (lasiocarpum); 12277 (cyanocarphium); 13390 (procumbens); 13599 (violaceum); 14481 (involucratum); 14578 (trilobatum); 15590 (lasiocarpum); 16337 (procumbens); 16415 (praetermissum); 16836 (pseudosaponaceum); 16845 (pseudosaponaceum); 17959 (procumbens); 18866 (insanum); 18869 (insanum); 19554 (lasiocarpum); 19702 (involucratum); 20213 (violaceum); 20487 (barbisetum); 20877 (nienkui); 20943 (insanum); 20990 (violaceum); 21010 (insanum); 21657 (involucratum); 22494 (lasiocarpum); 22521 (lasiocarpum); 25787 (praetermissum); 26089 (insanum); 27167 (violaceum); 27848 (procumbens); 27887 (procumbens); 28113 (insanum); 28172 (praetermissum); 28332 (lasiocarpum); 28533 (insanum); 28854 (insanum); 29290 (procumbens); 32478 (violaceum).

Polhill, R.M. 181 (aculeatissimum); 267 (giganteum); 1505 (aculeatissimum); 2283 (arundo).

Pollard, B.J. 1035 (giganteum).

Pollard, C.L. 324 (torvum).

Polunin, O. 815 (violaceum); 3607 (virginianum); 5705 (violaceum).

Pooma, R. 5485 (trilobatum); 6715 (trilobatum).

Popov, G.B. P4 11 (cordatum); GP 69 266 (elaeagnifolium); GP/H 606 (cordatum).

Porras, N. 1 (mammosum); 2 (mammosum); 3 (jamaicense).

Portal, E. 602 (sisymbriifolium); 811 (sisymbriifolium).

Pottier, ? 263 (insanum); 506 A (insanum); 576 (violaceum).

Powell, D.A. 62 (involucratum).

Powell, J.M. UPNG 1705 (torvoideum).

Powys, G. 390 (forskalii).

Prain's collector 72 (torvum); 250 (torvum); 3855 (barbisetum).

Prance, G.T. 2894 (sisymbriifolium); 8822 (sisymbriifolium); 30347 (jamaicense).

Prasad, R. CC-31647 (insanum).

Pratt, C.J. 196 (arundo).

Praveen, K.K. PR 91 (virginianum); PR 92 (torvum); PR 93 (torvum).

Prawiroatmodjo, S. 1356 (sulawesi).

Premanath, R.K. ANC-5986 (torvum); ANC-8442 (torvum).

Prescott, T.A.K. 28 (torvum).

Price, D. WK 167 (aculeatissimum).

Price, T. 338 (elaeagnifolium).

Pringle, C.G. 1566 (elaeagnifolium); 9370 (elaeagnifolium).

Procter, J.E.A. 313 (giganteum); 4473 (torvum).

Proctor, G.R. 10625 (mammosum); 11246 (mammosum); 16834 (torvum); 16862 (capsicoides); 16864 (jamaicense); 17570 (torvum); 17604 (jamaicense); 17805 (capsicoides); 18980 (capsicoides); 19170 (torvum); 19762 (jamaicense); 19853 (torvum); 20635 (jamaicense); 20782 (jamaicense); 22802 (jamaicense); 23539 (jamaicense); 23625 (capsicoides); 23753 (capsicoides); 27394 (jamaicense); 30279 (torvum); 30960 (elaeagnifolium); 31737 (capsicoides); 33728 (capsicoides); 38955 (jamaicense); 48250 (torvum); 49664 (elaeagnifolium).

Project Soma GM-6 (lasiocarpum); XM-33 (lasiocarpum).

Projeto Pedregal de San Angel 354 (sisymbriifolium).

Puccioni, N. 111 (115) (arundo); 551 [605] (cordatum); 1061 [605] (cordatum).

Pujalte, J.C. 210 (elaeagnifolium); 259 (elaeagnifolium).

Pullaiah, T. 333 (pubescens).

Pulle, A. 3151 (lasiocarpum).

Pullen, R. 8060 (torvoideum).

Puri, G.S. 339 (hovei); 2939 (hovei); 6001 (hovei); 8481 (hovei); 9145 (hovei); 12599 (hovei); 14047 (hovei); 20056 (hovei).

Puri, V. 71 (violaceum); 478 (violaceum).

Purpus, C.A. 4548 (elaeagnifolium).

Purseglove, J.W. P 1201 a (aethiopicum); P 1202 (aethiopicum); P 1203 (aethiopicum); 1413 (aculeatissimum); P 1757 (macrocarpon); P 1861 (macrocarpon); P 1893 (macrocarpon); 3155 (aculeatissimum); P 3562 (aethiopicum); 4120 (torvum); 4260 (torvum).

Put Phraisurind 28 a (barbisetum); 1773 (barbisetum); 3227 (putii); 3683 (cyanocarphium); 4031 (barbisetum).

Putman, P.T.L. 130 (giganteum).

Pynaert, L. 302 (chrysotrichum).

Quang, B.H. HN-NY 1213 (torvum).

Quevedo G, E. 134 (jamaicense).

Quick, C.R. 69-15 (elaeagnifolium).

Quiñones M, L.M. 27 (mammosum).

Quintas, F.J.D. 43 (aethiopicum).

Quiroz-Villarreal, D.K. 1116 (macrocarpon); 1325 (torvum); 1792 (torvum); 1848 (torvum).

Quisumbing, E. 45 x9 2066 (torvum); 82295 (torvoideum).

Rabelo, B.V. 1856 (jamaicense); 3754 (jamaicense).

Rabil, N. 204 (barbisetum).

Radcliffe-Smith, A. 4740 (cordatum).

Raffaeli, M. 1194 (cordatum).

Raghavan, R.S. 62536 (multiflorum); 68102 (hovei); 74029 (multiflorum); 74160 (capsicoides); 74210 (multiflorum); 79628 (multiflorum); 90267 (multiflorum); WC-97232 (capsicoides); 103287 (hovei); 103974 (multiflorum).

Ragonese, A. 7948 (sisymbriifolium).

Ragonese, A.M. 6390 (elaeagnifolium).

Ragupathy, S. 1085 (trilobatum).

Rahayu, M. 318 (lasiocarpum).

Rahman's collector 1124 (torvum).

Rahman, M.A. 98-108 (torvum); 98-165 (torvum); 4604 (torvum).

Raizada's collectors 26031 (pubescens).

Raizada, M.B. 21109 (violaceum); 23149 (virginianum).

Rajamani, C. 76 (virginianum); 1992 (giganteum).

Rajan, R. 86240 (virginianum); 86353 (trilobatum).

Rajendran, A. RHT 12625 (torvum); 86731 (vagum).

Raji, D.C.S. 291 (trilobatum).

Raju, R.R.V. 3107 (melongena).

Raju, S.R. 6161 (pubescens); 20258 (multiflorum).

Rakotomalala, L. 25 (torvum).

Rakotondrafara, A. 321 (torvum).

Rakotozafy, A. 2070 (insanum); 2078 (insanum).

Ralimanana, H. RLI-871 (insanum).

Ram, J.V.S. NC-39940 (violaceum).

Ram, S.B. SFS-2018 (virginianum).

Rama Rao, M. 571 (capsicoides); 2225 (capsicoides); 2312 (capsicoides).

Ramachandran, A. 0846 (wightii).

Ramachandran, V.S. 52352 (multiflorum); 54193 (multiflorum); 62156 (multiflorum); 62747 (multiflorum); 65364 (multiflorum).

Ramahlo Campêlo, C. 2235 (jamaicense).

Ramalho, R. 624 (robustum).

Ramamurthy, K. 18177 (multiflorum); 20241 (pubescens); 22807 (trilobatum); 23370 (multiflorum); 25845 (trilobatum); SC-25846 (insanum); 49373 (multiflorum); SC-49388 (insanum); 50651 (pubescens); 51175 (trilobatum); 51218 (trilobatum); 52899 (trilobatum); SC-53624 (insanum); 53658 (trilobatum); SC-53660 (insanum); 60121 (trilobatum); 60369 (trilobatum); 64171 (trilobatum); 77363 (pubescens); SC-77438 (insanum); 77579 (pubescens); 79615 (insanum); SC-83835 (insanum); 84628 (pubescens); 84629 (pubescens); 86490 (trilobatum); 88834 (trilobatum).

Ramanandan, P. 4847 (torvum).

Ramanujam, M.P. MPR-106 (trilobatum); MPR-232 (insanum); MPR-233 (insanum); MPR-234 (trilobatum).

Ramaswami, M.S. 1688 (violaceum).

Rambo, B. 31514 (sisymbriifolium).

Ramesh, B.R. 501 (pubescens); 726 (pubescens); 1056 (pubescens); 1512 (lasiocarpum).

Ramesh, S. .R. KFP-1879 (lasiocarpum).

Ramírez G, J. 221 (jamaicense).

Ramírez, J.G. 1209 (jamaicense); 1220 (jamaicense); 1457 (jamaicense); 1750 (jamaicense); 1915 (jamaicense); 7720 (jamaicense).

Ramlanto, ? 60 (jamaicense); 863 (lasiocarpum); 865 (lasiocarpum).

Ramos, M. 623 (melongena); 1049 (torvoideum); 1820 (torvum); 1857 (lasiocarpum); 4792 (retrorsum); 4955 (insanum); 8091 (retrorsum); 15341 (aethiopicum); 20544 (torvoideum); 22426 (torvoideum); 22586 (lasiocarpum); 24376 (lasiocarpum); 26487 (retrorsum); 27004 (pseudosaponaceum); 33186 (retrorsum); 33235 (retrorsum); 34812 (lasiocarpum); 36583 (cyanocarphium); 36789 (torvoideum); 37058 (torvoideum); 37835 (torvoideum); 38020 (torvoideum); 39466 (lasiocarpum); 40197 (pseudosaponaceum); 43154 (cyanocarphium); 43228 (lasiocarpum); 43939 (lasiocarpum); 44333 (cyanocarphium); 45077 (retrorsum); 46482 (torvoideum); 46590 (retrorsum); 47243 (lasiocarpum); 49081 (lasiocarpum); 49141 (melongena); 49324 (schefferi); 79839 (miyakojimense); 85001 (lasiocarpum); 85142 (cyanocarphium).

Randeria, A.F. AR 18 (hovei); AR 113 (hovei); A.R. 385 (hovei).

Randeria, G.J. AR 50 (hovei); AR 282 (hovei); 29003 (hovei).

Randolph, D. 173 (elaeagnifolium); 394 (elaeagnifolium).

Rangel G, A. 98 (sisymbriifolium).

Rangel Ramírez, R. 130 (elaeagnifolium).

Rangel, O. 1442 (jamaicense); 2968 (sisymbriifolium); 10162 (jamaicense).

Rangel, R. 330 (elaeagnifolium).

Rani, N. AHT-28282 (giganteum).

Rao Rolls, S. WC-92454 (insanum).

Rao, A.S. 7760 (hovei); 61707 (multiflorum); 74417 (multiflorum); 74452 (multiflorum); 77922 (hovei); 79761 (multiflorum); 79815 (multiflorum); 80001 (multiflorum); 80163 (multiflorum); WC-80447 (trilobatum); 81575 (hovei); 85335 (multiflorum); 85425 (multiflorum); 85784 (multiflorum); 85948 (multiflorum); 95633 (multiflorum); 131545 (hovei).

Rao, J.V.S. NC-39878 (insanum).

Rao, K.K. 74 (torvum).

Rao, P.S.N. ANC-15598 (torvum).

Rao, R.S. 66175 (hovei); 83455 (hovei); 84337 (hovei); 85243 (hovei).

Rao, T.A. CNH-561 (arundo); CNH-1453 (insanum); NC-1597 (violaceum); CNH-1949 (arundo); CNH-2086 (insanum); NC-3214 (violaceum); NC-3214 (violaceum); 4209 (trilobatum); 5614 (trilobatum); CNH-7234 (trilobatum); CNH-7441 (trilobatum); NC-10755 (insanum).

Rao, Y.V.S. 39878 (insanum).

Rashid, A. 3692 (elaeagnifolium).

Rasp, A.E. 68 (elaeagnifolium).

Rau, M.A. 2414 (chrysotrichum); NC-14597 (chrysotrichum).

Raulerson, L. 27617 (torvum).

Raven, P.H. 16690 (elaeagnifolium).

Raynal, A. 15949 (torvum).

Raynal, J. 20752 (giganteum).

Razali, H. HR-1 (torvum).

Read, B.E. R 1340 (sisymbriifolium).

Reales, A. 952 (elaeagnifolium); 968 (elaeagnifolium); 1615 (elaeagnifolium); 1678 (elaeagnifolium); 2015 (elaeagnifolium).

Ream, R.R. 440 (pseudosaponaceum).

Rechinger, K. 4821 (dunalianum).

Rechinger, K.H. 29735 (elaeagnifolium); 29752 (elaeagnifolium); 63645 (elaeagnifolium).

Redden, K.M. 2279 (jamaicense).

Reddick, D. 434 (elaeagnifolium).

Redmogue, A. EA 13514 (aculeatissimum).

Reed, C.F. 44349 (virginianum).

Reekmans, M. 7727 (mammosum).

Regnell, A.F. III 982 [a] (viarum); III-985 (sisymbriifolium).

Reidel, L. 135 (aculeatissimum); 392 (capsicoides); 764 (capsicoides).

Reina, M. 410 (sisymbriifolium).

Reineck, E.M. 86 (sisymbriifolium).

Reitsma, J.M. 2056 (torvum).

Reitz, R. 14300 (sisymbriifolium); 16898 (sisymbriifolium).

Reksodikardjo, S. 199 (dunalianum).

Remaudiere, G. 1797 (virginianum).

Renderos, M.A. 547 (mammosum).

Rendle, A.B. 102 (torvum).

Rensch, I. 1501 (torvoideum).

Rentería, E. 1581 (jamaicense).

Renvoize, S.A. 1925 (aculeatissimum).

Reodi, B. 84730 (hovei).

Reporter on Economic Products to the Govt. of India 11787 (barbisetum).

Repton, J.E. 621 (giganteum).

Revel-Macdonald, N. 49 (melongena).

Revilla, J. 1233 (jamaicense); 8396 (viarum).

Revisión de Plantas Vasculares Filipinas 1625 (torvoideum).

Reyes García, A. 2067 (torvum); 2243 (jamaicense); 2246 (torvum); 2342 (mammosum).

Reynoso 4379 (torvum); 24173 (torvum); 118706 (lasiocarpum).

Ribas, O.S. 2106 (torvum); 3181 (viarum); 3182 (aculeatissimum); 3219 (robustum); 5594 (sisymbriifolium); 5856 (sisymbriifolium); 7320 (sisymbriifolium); 7457 (sisymbriifolium).

Rice, C. 44 (virginianum).

Rich, H.H. 341 (virginianum); 496 (insanum).

Richards, H.M. Mrs 7550 (aculeatissimum); 8683 (aculeatissimum); 13844 (aculeatissimum); 20351 (aculeatissimum); 20354 (giganteum); 21632 (giganteum); 23316 (aculeatissimum); 23830 (aculeatissimum); 23897 (giganteum); 24970 (arundo); 26566 (aculeatissimum); 28835 (arundo).

Ricksecker, A.E. 123 (torvum).

Ricksecker, J.J. 71 (torvum).

Ridley, H.N. 76 (mammosum); 7159 (chrysotrichum); 12383 (torvum); 14250 (involucratum).

Ridsdale, C.E. 5457 (lasiocarpum).

Riedel, J. 54 (elaeagnifolium).

Riedel, L. 392 (capsicoides); 396 (robustum); 408 (capsicoides).

Rihu, ? 752 (violaceum).

Rimachi Y, M. 304 (jamaicense); 699 (sisymbriifolium); 822 (jamaicense); 5954 (jamaicense); 10622 (sisymbriifolium).

Ringuelet, E.J. 255 (elaeagnifolium).

Ritchie, C. 509 [a] (hovei); 509 [b] (violaceum).

Rivera, D. DANA 31 (sisymbriifolium); DANA 44 (sisymbriifolium).

Rob, E.; Fries, C.E. 917 (aculeatissimum).

Robbins, R.G. 79 (torvoideum); 808 (torvoideum).

Robbins, S.B. 5551 (torvum).

Robertson, S.A. 439 (aculeatissimum); 770 (aculeatissimum).

Robinette, W.L. 1010 (arundo).

Robinson, C.B. 285 (melongena); 286 (lasiocarpum); 287 (lasiocarpum); 288 (melongena); 1082 (robinsonii); 1153 (insanum).

Robinson, H.C. 38 (torvum).

Robleto, W. 746 (wrightii).

Robson, N.K.B. 1067 (chrysotrichum); 1674 (giganteum).

Robson, T.O. 222 (arundo).

Rock, J.F. 2339 (torvum).

Rodin, R.J. 3959 (giganteum).

Rodrigues, I.M.C. 427 (robustum).

Rodrigues, W.A. 5483 (jamaicense).

Rodriguez A, C. 30 (sisymbriifolium).

Rodríguez C, A. 6100 (elaeagnifolium).

Rodríguez R, E. 1605 (capsicoides).

Rodríguez, ? 200 (viarum); (V) 869 (elaeagnifolium).

Rodríguez, A. 35 (torvum); 11714 (torvum).

Rodríguez, D. 17 (chrysotrichum); 1891 (melongena); 2163 (mammosum); 2800 (torvum); 2969 (torvum); 3944 (chrysotrichum); 5671 (chrysotrichum).

Rodríguez, E. 42 (elaeagnifolium); 1605 (capsicoides).

Rodríguez, J.P. 43 (elaeagnifolium).

Rodríguez, J.V. 2923 (jamaicense).

Rodríquez, H. 121 (wrightii).

Roe, K.E. 1406 (torvum).

Rogers, F.A. 10008 (aculeatissimum); 18078 (aculeatissimum); 20291 (sisymbriifolium).

Rojas, T. 458 (robustum); 1992 (elaeagnifolium); 8342 (sisymbriifolium); 13678 (elaeagnifolium); 14310 (sisymbriifolium).

Roldan, F.J. 605 (jamaicense).

Rolfe, R.A. 1269 (torvum).

Rolla, S.R. 66519 (violaceum); 69711 (hovei); 71716 (hovei); 73614 (multiflorum); 77625 (hovei); 86542 (hovei); 87491 (hovei); 88664 (hovei).

Rollins, R.C. 2749 (elaeagnifolium).

Román Miranda, M.L. C-742 (elaeagnifolium); 1337 (elaeagnifolium).

Román V, C. 55 (jamaicense).

Romero, M. de 103 (melongena).

Romero, S. 1405 (elaeagnifolium); 1406 (elaeagnifolium).

Romero-Castañeda, R. 3778 (jamaicense); 6262 (jamaicense); 6583 (torvum); 8371 (jamaicense).

Rosales, J.M. 1939 (torvum); 2413 (torvum); 2507 (torvum).

Rosas R, M. 1423 (chrysotrichum).

Rose, J.N. 23054 (sisymbriifolium).

Rose, L.S. 65121 (elaeagnifolium).

Ross, A.F. 135 /10/8 (torvum).

Ross, R. 80 (torvum); SAN 190 (torvum); 273 (torvum).

Rossato, M. 4575 (sisymbriifolium).

Rothé, ? 18 (lasiocarpum); 78 (procumbens).

Rottland, T. D 270 (aculeatissimum).

Rouhan, G. 27 (torvum).

Roy, A. CNH-11965 (insanum); CNH-14914 (trilobatum).

Roy, G.F. CC-44994 (insanum).

Roy, G.P. 2747 (melongena); AC-3950 (melongena); CC-43624 (melongena).

Roybal, J.J. 904 (elaeagnifolium).

Royen, P. van 3136 (lasiocarpum); 4553 (dunalianum); 8103 (torvoideum).

Rubio, D. 1492 (mammosum).

Rubtzoff, P. 1321 (elaeagnifolium).

Rueda, R. 1382 (jamaicense).

Rueda, R.M. 891 (sisymbriifolium); 16783 (wrightii).

Ruffo, C.K. 159 (robustum).

Ruffo, F.R. 2739 (aculeatissimum).

Rugel, F. 239 (torvum); 240 (torvum); 293 (torvum); 294 (capsicoides).

Ruiz, L. 53398 (elaeagnifolium).

Runyinya, B. 872 (giganteum).

Runyon, R. 4194 (elaeagnifolium).

Rusby, H.H. 451 (wrightii).

Ruse, L.F. 142 (torvum).

Russell, G.F. 530 (jamaicense).

Russell, P.G. 51541 (wrightii).

Rwaburindore, P.K. 3861 (aculeatissimum).

Ryan, G.A. 313 (hovei).

Ryan, G.M. 1475 (hovei); 1488 (giganteum).

Ryan, P. 336 (torvum).

Ryder, V.P. 248 (elaeagnifolium).

Rzedowski, J. 16116 (elaeagnifolium); 29179 (elaeagnifolium).

Sacco, J.C. 167 (sisymbriifolium).

Safui, B. CNH-2488 (arundo).

Sagalyn, J.L. 69 (elaeagnifolium).

Sagástegui, A. 16014 (sisymbriifolium).

Sahira 35825 (melongena).

Sahni, K.C. 563 (violaceum); 3418 (hovei); 6154 (hovei).

Saito, S. 6965 (peikuoense).

Saldanha, C.J. CS 5129 (multiflorum); CS 5129 A (multiflorum); CS 5130 (multiflorum); CS 6256 (multiflorum).

Saldías, M. 4996 (sisymbriifolium).

Salimena, F.R.G. 2833 (sisymbriifolium); 3293 (viarum).

Salinas T, A. 7104 (elaeagnifolium).

Salino, A. 4705 (robustum); 9001 (robustum).

Sallee, E.H. ES-54 (elaeagnifolium).

Sallu, A.N. 412 (giganteum); 457 (robustum).

Salubeni, A.J. 512 (aculeatissimum).

Salzmann, P. 384 (jamaicense); 392 (melongena).

Sampath Kumar, V. SC-126916 (virginianum); SC-126918 (insanum); SC-126919 (torvum); SC-126920 (robustum); SC-126922 (sisymbriifolium); SC-126923 (viarum); SC-126932, SC-126933, SC-126937 (multiflorum); SC-126938 (sisymbriifolium); SC-126940 (torvum); SC-126941 (insanum); SC-126942 (elaeagnifolium); SC-126943 (torvum); SC-126944 (viarum); SC-126945 (violaceum); SC-51 (viarum); SC-126918 126946 (torvum); SC-126947 (violaceum); SC-126949, SC-126950 (multiflorum); SC-126953, SC-126958, SC-126960 (multiflorum); SC-126962 (viarum); SC-126963 (multiflorum); SC-126964 (torvum); SC-126965, SC-126966 (violaceum); SC-126968 (virginianum); SC-126969 (torvum); SC-126970 (trilobatum); SC-126971 (insanum); SC-126972 (elaeagnifolium); SC-126973 (insanum); SC-126974 (torvum); SC-126975 (pubescens); SC-126976 (viarum); SC-126977 (torvum).

Sampson 13816 (procumbens).

Samvura, A. 115 (torvum).

Sanabia, O.L. 333 (mammosum).

Sanabria G, A. 386 (jamaicense).

Sanadiki, N. 56 (pubescens).

Sanane, M. 1398 (aculeatissimum).

Sanarthanam, K.P. NC-51468 (violaceum).

Sánchez Mejorada R, H. 80 (elaeagnifolium).

Sanders, A.C. 19612 (elaeagnifolium); 25026 (elaeagnifolium).

Sandino, J.C. 5076 (wrightii); 5118 (jamaicense).

Sands, L.J.S. 6330 (lasiocarpum).

Sands, M.J.S. 477 (poka); 1384 (dunalianum).

Santa S, J.I. 249 (jamaicense).

Santapau, H. HS 589 (hovei); HS 746 (hovei); HS 1443 (hovei); HS 2035 (hovei); HS 2092 (hovei); HS 2093 (hovei); HS 2094 (hovei); 2972 (virginianum); 2973 (virginianum); HS 10212 (hovei); 11574 (hovei); HS 14461 (hovei); HS 14462 (hovei); HS 15830 (hovei); HS 16095 (hovei); HS 17291 (hovei); HS 17365 (hovei); HS 17516 (hovei); HS 17517 (hovei); HS 17579 (hovei); HS 18855 (hovei); HS 18880 (hovei); HS 18963 (hovei); HS 20767 (multiflorum); HS 22226 (hovei); HS 22458 (hovei); HS 23085 (multiflorum); HS 27337 (hovei); HS 27423 (hovei).

Santiago, D.S. 150 (sisymbriifolium); 187 (sisymbriifolium); 478 (viarum); 530 (viarum).

Santisuk, T. 468 (virginianum).

Santos, E.B. 331 (macrocarpon).

Santos, E.B. dos 331 (macrocarpon).

Santos, J.K. 4 (lasiocarpum); 30 26 (retrorsum).

Santos, J.L. 776 (jamaicense).

Santos, J.V. 2720 (torvum); 4147 (cyanocarphium); 5470 (pseudosaponaceum).

Santos, K. 37 (torvoideum); 224 (robustum).

Sanyal, M.N. 879 (melongena); 880 (melongena).

Saran, R. 306 (violaceum); 5367 (viarum).

Saravia Toledo, C. 1675 (sisymbriifolium); 1897 (sisymbriifolium); 2336 a (elaeagnifolium); 4066 (sisymbriifolium).

Sargent, F.H. 81 (torvum); 253 (mammosum); 319 (capsicoides); 513 (elaeagnifolium).

Sarin, Y.K. NC-1491 (chrysotrichum); NC-5079 (insanum).

Särkinen, T. 4079 (chrysotrichum); 4536 (sisymbriifolium); 4547 (capsicoides); 4562 (sisymbriifolium); 4631 (sisymbriifolium).

Sarmiento L, H. 523 (jamaicense).

Sarson, C. 42 (aculeatissimum).

Sasaki, S. 275 [a] (pseudosaponaceum).

Sastre, C. 2182 (jamaicense); 2805 (capsicoides); 3092 (jamaicense).

Sastry, A.R.K. CNH-9264 (insanum); BSI-40896 (violaceum).

Sato, R. 31 (giganteum).

Sato, S. 119 (arundo); 173 (cordatum); 456 (forskalii); 457 (forskalii); 600 (forskalii).

Satyanarayana, P. 122410 (pubescens).

Sauleda, R. 2649 (elaeagnifolium).

Saulière, A. 52 bis (multiflorum); 370 bis (multiflorum).

Saunders, J. 262 (torvum).

Savinierre, E. de la 343 (poka); 1171 (torvum).

Saxton, W.T. 499 (cordatum).

Sayago, M. 570 (elaeagnifolium).

Sayers, C.D. NGF-21505 (torvoideum).

Scaldaferro, M. 21 (elaeagnifolium); 25 (elaeagnifolium).

Schaffner, J.G. 695 (elaeagnifolium).

Schallert, P.O. 540 (elaeagnifolium); 8212 (capsicoides); 8212 a (capsicoides).

Scheepers, J.C. 538 (aculeatissimum).

Schickendantz, F. 80 (elaeagnifolium); 82 (elaeagnifolium).

Schiede, C.J.W. 140 (aculeatissimum).

Schierbrand, W.C. von 137 (pseudosaponaceum).

Schiffner, V. 2504 (involucratum); 2517 (jamaicense); 2518 (involucratum).

Schimper, G.H.W. 1338 (giganteum).

Schinini, A. 3494 (robustum); 4492 (robustum); 5021 (robustum); 8740 (sisymbriifolium); 37059 (elaeagnifolium).

Schipp, W.A. 175 (torvum); S-664 (mammosum).

Schlagintweit, H.A.R. von 3220 (virginianum); 4409 (melongena); 10480 (virginianum).

Schlechter, F.R.R. 13749 (dunalianum); 15022 (torvum); 17627 (schefferi).

Schlieben, H.J. 2708 (giganteum); 3376 (aculeatissimum); 4494 (aculeatissimum); 11299 (torvum); 11794 (torvum).

Schlottmann, A. 269 (elaeagnifolium).

Schmeda, G. 433 (robustum).

Schmid, M. 1084 (violaceum); 1087 (insanum); 1608 (insanum).

Schmitz, A. 166 [a] (elaeagnifolium); 166 [b] (elaeagnifolium).

Schmutz, E. 702 (lasiocarpum).

Schneider, C. 2545 (torvum).

Schnell, R. 11705 (torvum).

Schodde, R. 2141 (torvoideum); 4244 (dunalianum).

Scholl, M.R. 26 (elaeagnifolium).

Scholz, U. 418 (torvum); 533 (torvum).

Schomburgk, R. 217 (insanum).

Schreiter, R. 12408 (elaeagnifolium).

Schroeder, F. 94 (aethiopicum).

Schubert, B.G. 650 (chrysotrichum).

Schultes, R.E. 3450 (mammosum); 3651 (mammosum); 3808 (mammosum); 3893 (jamaicense); 8203 (jamaicense); 24098 (jamaicense).

Schulz, C.L. 1363 (elaeagnifolium); 5590 (elaeagnifolium).

Schulz, E.D. 97 (elaeagnifolium).

Schumann, W. 970 (elaeagnifolium).

Schunke Vigo, J. 1788 (sisymbriifolium); 7355 (sisymbriifolium); 10509 (mammosum); 14674 (sisymbriifolium).

Schwarz, G.J. 241 (robustum); 1142 (sisymbriifolium); 1481 (sisymbriifolium); 6938 (robustum); 8354 (robustum); 9758 (elaeagnifolium).

Schweinfurth, G.A. 82 (forskalii); 296 (forskalii); 1362 (forskalii); 1364 (forskalii); 1380 (melongena).

Sciuva, ? 10 (arundo).

Scolnik, R. 19S 020 (jamaicense).

Scora, R.W. 2285 (elaeagnifolium).

Scott, D.r. 32 (hovei).

Scott-Elliot, G.F. 442 (elaeagnifolium); 7693 (aculeatissimum).

Sebastine, K.M. 540 (pubescens); 1195 (trilobatum); SC-2038 (robustum); 3726 (vagum); 4539 (vagum); 5181 (trilobatum); 5898 (vagum); 8523 (vagum); 9640 (vagum); 9729 (pubescens); SC-11666 (insanum); 12510 (pubescens); SC-14577 (insanum); 14589 (pubescens); 18433 (multiflorum).

Sebsebe Demissew 1780 (cordatum).

Second Darwin Nepal Fieldwork Training Expedition B-38 (torvum).

Sedgwick, L.J. 3939 (virginianum); 4189 (giganteum); 4745 (giganteum); 5654 (giganteum); 6085 (torvum); 7281 (torvum).

Seemann, B.C. 164 (mammosum); 197 (melongena).

Sehnem, A. 3773 (sisymbriifolium).

Seidel, R. 7419 (wrightii).

Seidenschwarz, F.G. 364 /1 (sisymbriifolium).

Selander, R.B. 4 -53 (elaeagnifolium); 6 -53 (elaeagnifolium); 21 (elaeagnifolium).

Selassie, L.G. 611 (giganteum).

Semir, J. 31622 (viarum).

Semsei, S. 2787 (robustum); 3953 (aculeatissimum); 4128 (robustum).

Semsei, S.R. 2182 (giganteum); 2830 (giganteum); 3953 (aculeatissimum); 4126 (giganteum).

Sendulsky, T. 526 (capsicoides).

Sengupta, G. 35 (violaceum); 145 (trilobatum); 924 (violaceum); 974 (viarum); 1205 (viarum); 1326 (violaceum); 14592 (melongena).

Senn, H.A. 226 (torvum).

Senni, L. 11 (arundo); 799 (cordatum).

Seret, F. 166 (giganteum); 1094 (aculeatissimum).

Serna I, R.A. 600 (jamaicense).

Serrano, A. 128 (elaeagnifolium).

Serrano, M. 6976 (sisymbriifolium).

Sessé, M. 1415 (chrysotrichum); 1532 (elaeagnifolium); 1535 (mammosum); 5382 (elaeagnifolium).

Seyrig, A. 264 (insanum).

Shabani, S. 333 (aculeatissimum).

Shabetai, J.R. F. 1753 bis (forskalii).

Shafer, J.A. 10376 (jamaicense); 12358 (torvum).

Shah, G.L. 812 (hovei); 9041 (hovei); 9122 (hovei).

Sharifi, M.R. 313 (virginianum); 508 (virginianum).

Sharland, R.E. 1856 (torvum); 2041 (torvum).

Sharma, B.D. 35638 (multiflorum); 35953 (multiflorum); 36100 (wightii); 42430 (multiflorum); 167305 (hovei); 167857 (hovei).

Sharma, J.P. NC-79451 (chrysotrichum).

Sharma, S. SS-93 (insanum).

Sharman, A. 44 (macrocarpon).

Shea, G. SAN-75994 (lasiocarpum).

Shein, L. 88370 (torvum).

Shenoy, K.V. K.S.V. 11 (hovei); K.S.V. 94 (hovei); K.S.V. 168 (hovei); K.S.V. 553 (hovei); K.S.V. 1960 (hovei); K.S.V. 1997 (hovei); K.S.V. 2124 (hovei); K.S.V. 3822 (hovei); K.S.V. 5613 (hovei).

Shepherd, G.J. 4082 (sisymbriifolium); 19141 (robustum).

Shepherd, J.D. 460 (jamaicense).

Shetty, B.V. AC-2272 (forskalii); 10349 (multiflorum); 11915 (multiflorum); 26461 (multiflorum); 27932 (pubescens); 27980 (vagum); 37663 (multiflorum).

Shevock, J. 502 (elaeagnifolium); 12209 (elaeagnifolium).

Shimizu, T. T 8257 (torvum).

Shine, L. 96319 (torvum); 96349 (violaceum).

Shirasuna, R.T. 821 (robustum).

Short, M.J. 35 (jamaicense).

Shukla, A.N. NC-122192 (insanum).

Shukle, U. NC-70031 (violaceum); NC-70264 (violaceum).

Shuter, J. 1580 (trilobatum).

Si Boeea, R. 6286 (lasiocarpum); 6904 (melongena); 7097 (violaceum); 7740 (lasiocarpum); 8015 (torvum); 8741 (lasiocarpum); 8919 (melongena); 8922 (lasiocarpum); 9035 (lasiocarpum); 9225 (melongena).

Sibil, J. 286 (melongena).

Siddiqi, M.A. 6104 (insanum).

Sidiyasa, K. 1293 (lasiocarpum); BRF 1773 (lasiocarpum).

Sieber, F.W. 67 (torvum); 68 (mammosum); 227 (violaceum); 308 (melongena).

Siegenthaler, I.E. 1534 (giganteum).

Sikdar, J.K. CNH-36 (torvum); 709 (melongena).

Sillitoe, P. 50 (torvoideum).

Silva R, L. 184 (elaeagnifolium); 187 (elaeagnifolium).

Silva, D.A. 4611 (torvum).

Silva, E.M.G.B. 144 (torvum).

Silva, F. de Wall. Cat. 2628 e (barbisetum).

Silva, H.G.V. 67 (sisymbriifolium).

Silva, J.A.C. 115 (viarum).

Silva, J.M. 2608 (aculeatissimum); 2832 (capsicoides).

Silva, M. 1027 (jamaicense).

Silva, M.A. 1917 (mammosum); 2752 (sisymbriifolium).

Silva, M.G. 1027 (jamaicense).

Silverstone-Sopkin, P.A. 2037 (sisymbriifolium).

Simão Bianchini, R. CFCR 8809 (viarum).

Simmons, J.W. 39 (cordatum); JS 39 (cordatum).

Simon, G. 69 (aculeatissimum); 913 (aculeatissimum).

Simond, ? 77 (violaceum).

Simonds 40 (forskalii).

Simpson, N.D. 8050 (insanum); 8052 (torvum); 8755 (violaceum); 8890 (giganteum); 9196 (capsicoides); 9323 (trilobatum); 9344 (vagum).

Sinclair, J. 186 (melongena); 5153 (torvum); 9778 (lasiocarpum); 10889 (melongena).

Singh, A.N. AC-5742 (insanum); AC-7051 (melongena).

Singh, H. 497 (torvum).

Singh, N.P. 124144 (hovei); 124560 (hovei); 124704 (hovei); 124800 (hovei); 124875 (hovei); 125338 (hovei).

Singh, P. BSHC-15539 (violaceum).

Singh, R. 83 (violaceum).

Singh, U. 11 (violaceum); 99 (violaceum); 363 (violaceum); 365 (insanum).

Singh, V. AC-2889 (insanum); AC-2978 (virginianum); AC-5503 (insanum); AC-7263 (insanum); 80328 (elaeagnifolium).

Sino-American Botanical Expedition 15 (violaceum); 338 (violaceum).

Sintenis, P. 83 (torvum).

Sintenis, P.E.E. 1680 (jamaicense); 6059 (torvum).

Siqueiros, M. 515 (elaeagnifolium).

Sitinand, T. 4307 (praetermissum).

Sitoni, D. 962 (macrocarpon).

Six, I.L. 3409 -84 (elaeagnifolium).

Skehan, J. 16 (elaeagnifolium).

Skorupa, ? 384 (sisymbriifolium).

Skutch, A.F. 1994 (torvum); 3222 (chrysotrichum); 3926 (jamaicense); 4227 (jamaicense).

Small, J.K. 4841 (elaeagnifolium).

Smeyers, F. 184 (wrightii).

Smith, A.P. H-1628 (jamaicense); F-1757 (jamaicense).

Smith, E. 046 (procumbens); 478 (lasiocarpum); 4018 (lasiocarpum); 15398 (capsicoides).

Smith, G.L. 1030 (elaeagnifolium).

Smith, H.H. 329 (mammosum); 1146 (jamaicense).

Smith, J.F. 2 (torvum).

Smith, L.B. 3128 (torvum); 11184 (sisymbriifolium); 12263 (sisymbriifolium); 12648 (viarum); 13350 (aculeatissimum); 13724 (sisymbriifolium); 14638 (aculeatissimum).

Smith, S.D. 13 (torvum); 37 (torvum).

Smitinand, T. 3377 (violaceum); 4807 (praetermissum); 7421 (barbisetum).

Sneidern, K. von 1054 (jamaicense); 1230 (jamaicense); 2579 (jamaicense).

Snethlage, E.H. 31 (jamaicense).

Snowden, J.D. 375 (wrightii).

Soakai, E. 892 (torvum); 1048 (dunalianum).

Sobral, M. 14618 (robustum).

Sodiro, A. 755 (sisymbriifolium).

Soejarto, D.D. 2614 (mammosum); 4347 (jamaicense).

Soenarko, S. 298 (lasiocarpum).

Sohmer, S.H. 8333 (giganteum); 8555 (giganteum); 8756 (torvum).

Soibeh, D. 773 (melongena).

Solano, D. 1963 (capsicoides).

Solbrig, O.T. 140 (elaeagnifolium).

Soll, S. 252 (cordatum).

Solomon, J.C. 10318 (sisymbriifolium).

Song, H.M. 93 (miyakojimense).

Soosairaj, S. 2514 (wightii); 2514 (wightii).

Soria, N. 1881 (robustum); 2268 (robustum).

Soriano M, A.M. 144 (elaeagnifolium).

Soriano, A. 548 (elaeagnifolium); 1143 (elaeagnifolium).

Soriano, S. 13 (robustum).

Sosef, M.S.M. 1923 (aculeatissimum); 1989 (torvum).

Sota, A.V. de la 132 (elaeagnifolium).

Soto Núñez, J.C. 9508 (elaeagnifolium).

Soto, A. 983 (wrightii).

Soto, D.A. 1222 (chrysotrichum).

Soukup, J. 3557 (chrysotrichum).

South Vietnam First Darwin Expedition 85 (involucratum).

Souza, N.L. 172 (sisymbriifolium).

Souza, R.S. 124 (viarum).

Souza, V.C. 474 (torvum).

Sowastava, G. F.R.I. 116 (violaceum).

Soyaux, H. 329 (torvum).

Soza, D. 51 (wrightii).

Sparrow, C.E.H. 46 (elaeagnifolium).

Spegazzini, C.L. 28174 (elaeagnifolium); 33629 (elaeagnifolium).

Spencer, M.F. 1417 (elaeagnifolium).

Spetzman, L. 1366 (elaeagnifolium).

Spichiger, R. 5256 (robustum).

Spire, C.J. 126 (lasiocarpum); 914 (insanum); 1080 (lasiocarpum); 1081 (insanum); 1426 (violaceum).

Sprague, T.A. 13 (virginianum); 57 (virginianum).

Sprankle, J.A. 96 (elaeagnifolium).

Spruce, R. 2104 (jamaicense); 3887 (mammosum); 4144 (sisymbriifolium).

Squires, R.W. 27 (procumbens); 105 (torvum); 282 (insanum).

Sreemadhavan, C.P. CPS 173 (pubescens).

Sreenath, K.P. KFP-10225 (trilobatum).

Srinivasan, S.R. SC-51515 (robustum); 89592 (pubescens).

Srisanga, P. 3832 (barbisetum); 97239 (torvum); 97280 (viarum); 97698 (torvum); 97769 (viarum).

St Pierre, M. 2601 (elaeagnifolium).

Staff (Northern Centre) NC-1039 (chrysotrichum).

Stainton, J.D.A. 21 (torvum); 25 (virginianum); 552 (viarum); 2306 (virginianum); 2413 (viarum); 4830 (violaceum); 5126 (viarum); 5310 (violaceum); 6753 (violaceum); 6797 (torvum); 7615 (viarum); 8223 (torvum); 8749 (violaceum).

Stamatiadou, E. 13340 (elaeagnifolium); 15571 (elaeagnifolium).

Standley, P.C. 4823 (chrysotrichum); 8833 (jamaicense); 12562 (jamaicense); 18568 (jamaicense); 18839 (jamaicense); 19238 (jamaicense); 19661 (torvum); 19949 (torvum); 20767 (torvum); 21126 (torvum); 22011 (torvum); 22249 (torvum); 22588 (torvum); 24720 (jamaicense); 33353 (chrysotrichum); 44131 (chrysotrichum); 45216 (mammosum); 46778 (capsicoides); 46798 (jamaicense); 49340 (capsicoides); 50058 (chrysotrichum); 53232 (torvum); 53615 (jamaicense); 54533 (jamaicense); 55085 (torvum); 55476 (torvum); 55527 (torvum); 55958 (torvum); 72140 (jamaicense); 72614 (jamaicense); 85084 (chrysotrichum); 91849 (jamaicense).

Stanford, L.R. 273 (elaeagnifolium); 586 (elaeagnifolium); 802 (elaeagnifolium).

Starlinger, F. 22 -93 (elaeagnifolium).

Starr, R. 113 (mammosum).

Stauffer, H.U. 265 (aculeatissimum).

Stearn, W.T. 24 (virginianum); 32 (cordatum); 151 (capsicoides); 154 (torvum); 290 (torvum); 333 (torvum); 423 (capsicoides); 425 (torvum); 468 (torvum); 470 (capsicoides); 471 (jamaicense); 484 (capsicoides); 522 (jamaicense); 524 (capsicoides); 525 (mammosum); 682 (jamaicense); 683 (torvum); 913 (jamaicense); 997 (jamaicense).

Steenis, C.G.G.J. van 18309 (procumbens).

Stehmann, J.R. 101 (aculeatissimum); 205 (viarum); 206 (capsicoides); 366 (aculeatissimum); 428 (aculeatissimum); 552 (capsicoides); 621 (aculeatissimum); 654 (aculeatissimum); 695 (viarum); 754 (capsicoides); 792 (aculeatissimum); 1204 (sisymbriifolium); 1398 (sisymbriifolium); 1593 (viarum); 1600 (robustum); 1610 (aculeatissimum); 1638 (torvum); 2105 (sisymbriifolium); 2179 (aculeatissimum); 2185 (capsicoides); 2250 (aculeatissimum); 2265 (viarum); 4277 (aculeatissimum); 4320 (aculeatissimum); 6356 (viarum).

Steibel, P.E. 4092 (elaeagnifolium).

Steinbach, J. 8636 (sisymbriifolium); 8751 (sisymbriifolium); 9713 (sisymbriifolium).

Steinbach, R.F. 128 (sisymbriifolium); 684 (sisymbriifolium).

Steiner, M.L. 864 (torvoideum).

Stephens, S. 10953 (elaeagnifolium).

Stephenson, T.A. 531 (torvum).

Sterly, J. 80-215 (torvoideum).

Stern, S. 142 (sisymbriifolium); 155 (sisymbriifolium); 265 (jamaicense); 363 (torvum); 389 (jamaicense); 401 (jamaicense).

Stern, W.L. 219 (jamaicense).

Stevens, G.W. 802 (elaeagnifolium); 889 (elaeagnifolium).

Stevens, P.F. LAE-58631 (lasiocarpum).

Stevens, W.D. 2825 (torvum); 3380 (torvum); 4862 a (torvum); 4929 (torvum); 5012 (torvum); 5098 (mammosum); 6462 (torvum); 8003 (jamaicense); 8101 (torvum); 13451 (chrysotrichum); 14038 (chrysotrichum); 20045 (jamaicense); 25877 (torvum).

Steward, A.N. 1144 (capsicoides).

Stewart, J.L. 334 (cordatum); 2404 (violaceum).

Stewart, R. 41218 (virginianum).

Stewart, R.R. 230 (insanum); 397 (virginianum); 1071 (insanum); 13612 (cordatum); 15052 (torvum); 19349 A (virginianum).

Steyermark, J.A. 37432 (torvum); 44240 (jamaicense); 54843 (sisymbriifolium); 61233 (jamaicense); 83071 (elaeagnifolium).

Stiefkens, L.B. 4 (elaeagnifolium).

Stimson, W.R. 1403 (elaeagnifolium); 1605 (torvum).

Stirton, C.H. 6306 (chrysotrichum).

Stival-Santos, A. 987 (torvum); 3066 (capsicoides).

Stocks, J.E. 252 (cordatum).

Stofella, ? 328 (elaeagnifolium).

Stolz, A. 1552 (aculeatissimum).

Stone, B.C. PPI 688 (torvoideum); 14063 (torvum).

Strachey, R. 2 (virginianum); 5 (violaceum); 7 (insanum).

Streimann, H. 8498 (lasiocarpum); NGF-27523 (dunalianum); LAE-52624 (dunalianum).

Strey, R.G. 4537 (sisymbriifolium).

Strijk, J.S. 2 (torvum).

Stroh, A.L. 69. 63 .02 (elaeagnifolium).

Strother, J.L. 258 (elaeagnifolium).

Stubbings, H.G. 84 (torvum).

Stuckert, T. 1239 (elaeagnifolium); 1720 (elaeagnifolium); 2153 (elaeagnifolium); 2409 (elaeagnifolium); 2981 (elaeagnifolium); 3347 (elaeagnifolium); 3510 (elaeagnifolium); 4027 (elaeagnifolium); 4157 (elaeagnifolium); 4300 (elaeagnifolium); 4696 (elaeagnifolium); 6152 (elaeagnifolium); 6517 (elaeagnifolium); 7049 (elaeagnifolium); 8103 (elaeagnifolium); 8335 (elaeagnifolium); 8591 (elaeagnifolium); 8592 (elaeagnifolium); 8689 (elaeagnifolium); 9286 (elaeagnifolium); 9337 (elaeagnifolium); 10520 (elaeagnifolium); 11278 (elaeagnifolium); 11590 (elaeagnifolium); 12070 (elaeagnifolium); 15263 (elaeagnifolium); 15264 (elaeagnifolium); 15745 (elaeagnifolium); 16858 (elaeagnifolium); 16968 (elaeagnifolium); 19448 (elaeagnifolium); 19468 (elaeagnifolium); 19933 (elaeagnifolium); 19933 (elaeagnifolium); 23189 (elaeagnifolium); 23783 (elaeagnifolium).

Stuhlmann, F. 3662 (aethiopicum); 6432 (aethiopicum).

Suárez S, L.S. 986 (jamaicense).

Subba Rao, G.V. 2442 b (virginianum); SC-20166 (insanum); SC-22279 (insanum); SC-24413 (insanum); 31844 (pubescens); 36571 (multiflorum); 37340 (pubescens); 37470 (pubescens); 45801 (pubescens); 46813 (pubescens); SC-46980 (insanum).

Subils, R. 2319 (elaeagnifolium); 2909 (elaeagnifolium); 3427 (elaeagnifolium); 3584 (sisymbriifolium); 3589 b (elaeagnifolium); 3699 (elaeagnifolium); 3865 (sisymbriifolium); 3881 (elaeagnifolium); 4129 (robustum); 4131 (viarum); 4135 (sisymbriifolium); 4147 (viarum); 4173 (sisymbriifolium); 4174 (viarum); 4258 (robustum); 4262 (viarum); 4414 (elaeagnifolium); 4463 (elaeagnifolium); 4586 (sisymbriifolium); 4694 (sisymbriifolium); CORD-23068 (melongena).

Subramanian, K.N. 149 (trilobatum); 554 (pubescens); 736 (pubescens); 1608 (pubescens); 1807 (pubescens); 2330 (pubescens); 3348 (pubescens); 5113 (trilobatum); 5309 (trilobatum); 5594 (vagum); 6386 (pubescens); 10413 D (viarum); 14065 (pubescens).

Suclli, E. 1955 (sisymbriifolium).

Sucre, D. 3323 (torvum).

Sudheresan, C. 429 (multiflorum).

Sueltenfuss, C. 193 (elaeagnifolium).

Sugau, J.B. 337 (lasiocarpum).

Sugiyama, M. 554 (aculeatissimum).

Sulit, M.D. 1302 7079 (wrightii); 7450 (pseudosaponaceum); 7599 (torvoideum); 8502 (lasiocarpum); 9868 (torvoideum); 13802 (lasiocarpum); 16790 (pseudosaponaceum); 17133 (lasiocarpum).

Sumithraarachchi, D.B. 08S 536 (virginianum); DBS-912 (giganteum).

Sunderland, T.C.H. 1308 (torvum); 1485 (torvum).

Suppiah, T. FRI-14773 (lasiocarpum).

Susami, A. 1151 (violaceum).

Suzana, S. SAN-151080 (lasiocarpum).

Suzuki, M. 88-20084 (viarum); 88-20133 (violaceum); 88-60006 (viarum); 88-80032 (viarum).

Svolenski, A.C. 177 (sisymbriifolium).

Swarbrick, J.T. 2521 (aethiopicum).

Symes, Y.E. 521 (aculeatissimum).

Symon, D.E. 10657 (torvum); 10669 (torvoideum); 13856 (torvoideum); 14318 (elaeagnifolium); 15128 (elaeagnifolium).

Synnott, T.J. 746 (giganteum); 1269 (aculeatissimum); 1517 (aculeatissimum).

Sørensen, T. 77 (trilobatum); 1569 (torvum).

Sørenson, T. 1982 (trilobatum).

Taam, Y.W. 1759 (torvum); 1831 (insanum).

Täckholm, V. 2031 c (forskalii); 2031 b (forskalii).

Tadesse, E. 547 (aculeatissimum); 598 (macrocarpon).

Tadjouteu, F. 409 (torvum); 629 (torvum).

Tadong, D. 102 (lasiocarpum); 382 (lasiocarpum).

Tagawa, M. T-3160 (torvum).

Tai, L.Y. 11447 (pseudosaponaceum).

Takahashi, H. T 62798 (torvum); T 63068 (torvum).

Takaki, F. 326 (elaeagnifolium).

Takeuchi, W.N. 9299 (dunalianum); 9323 (dunalianum); 12549 (torvoideum); 16958 (torvum); 17830 (lasiocarpum); 23333 (lasiocarpum).

Takushi, A. 6400 (miyakojimense).

Talbot, P.A. 1062 (torvum).

Tanaka, N. 20306 (torvum); 20352 (virginianum); 23309 (viarum); 23310 (torvum); 30877 (melongena).

Tanaka, T. 5044 (capsicoides).

Tang Siu Ging, ? 15213 (pseudosaponaceum); 16169 (pseudosaponaceum); 16346 (pseudosaponaceum).

Tanner, R.E.S. 2256 (robustum).

Tao Guoda 4666 (barbisetum); 16747 (lasiocarpum); 17505 (barbisetum); 17509 (praetermissum); 43763 (lasiocarpum); 44995 (barbisetum); 47460 (praetermissum); 47466 (praetermissum); 930327 (praetermissum).

Tapia, J.L. 996 (torvum).

Taquet, E.J. 1155 (melongena).

Taroda, N. 18537 (robustum); 18538 (robustum).

Tate, R. 263 (205) (mammosum); 268 (jamaicense).

Tateishi, Y. 15125 (miyakojimense).

Tavakari, S.C. T 151 (hovei); T 1268 (hovei).

Tawakali, E.J. 296 (torvum).

Taylor, A.A. 223 (torvum).

Taylor, C.M. 1898 (elaeagnifolium); 2196 (chrysotrichum); 6546 (torvum); 6904 (torvum); 7482 (torvum); 11816 (torvum).

Taylor, G. 2505 (giganteum).

Taylor, P.M. P 112 (melongena); P 344 (melongena).

Taylor, R.J. 17463 (capsicoides).

Tchiengue, B. 2728 (aculeatissimum); 3140 (aculeatissimum).

Tchouto, P. 11 (torvum); 164 (torvum).

Teague, G.W. 351 (sisymbriifolium).

Tehan Uji 2793 (lasiocarpum).

Teijsmann, J.E. 7853 (schefferi); 7854 (dunalianum).

Tejada, R. 322 (chrysotrichum).

Tejero-Díez, D. 6256 (elaeagnifolium); 6517 (elaeagnifolium).

Telado, ? 1920 (melongena).

Téllez V, O. 6303 (torvum).

Temple, L.C. 5513 (elaeagnifolium); 12151 (elaeagnifolium).

Templeton, B. 8644 (elaeagnifolium).

Tenorio L, P. 1122 (elaeagnifolium); 13676 (elaeagnifolium).

Tepe, E.J. 2770 (macrocarpon); 2782 (robustum); 2786 (arundo); 2790 (wrightii).

Terracciano, A. 776/293 (forskalii); 783/2224 (forskalii); 784/2190 (forskalii); 797/2192 (forskalii); 2422 (forskalii); 2424 (forskalii); 2425 (forskalii); 2825 (forskalii).

Terribile, M. 808 (elaeagnifolium).

Tessmann, G. 459 (torvum); 513 (giganteum); 648 (aculeatissimum); 2095 a (aethiopicum).

Thackery, F.A. 226 (elaeagnifolium).

Thanikaimoni, G. G.T. 1231 (trilobatum).

Tharp, B.C. 52-78 (elaeagnifolium); 51-261 (elaeagnifolium).

Thesiger, W. 1667 (virginianum).

Thomas, D.W. 4234 (torvum); 4264 (torvum); 4506 (torvum).

Thomas, J.H. 9978 a (elaeagnifolium).

Thomas, N.W. 146 (aethiopicum); 456 (melongena); 991 (macrocarpon); 1162 (macrocarpon); 1635 (macrocarpon); 2063 (macrocarpon); 2946 (aculeatissimum); 4213 (aculeatissimum); 4357 (melongena).

Thomas, R.D. 96112 (elaeagnifolium).

Thomas, W.W. 4609 (sisymbriifolium); 11002 (torvum).

Thompson, S.A. 1402 (torvum); 1686 (wrightii); 1756 (macrocarpon).

Thompson, W.J. 8070 (capsicoides).

Thoms, M. 30 (jamaicense).

Thomson, G. 10 (multiflorum); 15 (insanum); 16 (insanum); 178 (melongena).

Thomson, T. 25 (melongena); 59 (violaceum); 304 138 (violaceum); 518 (virginianum); 578 (virginianum).

Thorel, C. 23 (insanum); 84 (lasiocarpum); 1419 (cyanocarphium); 3410 b (procumbens); 3410 a (procumbens); 9074 (torvum).

Thornber, J.J. 482 (elaeagnifolium).

Thornewill, A.S. 95 (macrocarpon).

Thothathri, K. 10331 (viarum).

Thulin, M. 4713 (forskalii); 11244 (forskalii).

Thurn, E.F. im 273 (torvum).

Thwaites, G.H.K. 1503 (giganteum); 1901 (vagum); 1903 (virginianum); 1903 [b] (giganteum); 1903 [c] (giganteum); 1904 (violaceum); 1905 (virginianum); 1906 (trilobatum); 1907 (insanum); 2868 (lasiocarpum).

Tilton, D. 218 (elaeagnifolium); 229 (elaeagnifolium); 236 (elaeagnifolium); 237 (elaeagnifolium); 241 (elaeagnifolium); 266 (elaeagnifolium); 267 (elaeagnifolium); 321 (elaeagnifolium); 330 (elaeagnifolium); 337 (elaeagnifolium); 353 (elaeagnifolium); 369 (elaeagnifolium); 374 (elaeagnifolium); 399 (elaeagnifolium).

Timaná, M. 3733 (mammosum).

Timberlake, J.R. 1032 (arundo).

Tirado, N. 2 (elaeagnifolium); 51 (elaeagnifolium).

Tirvengadum, D.D. 190 (violaceum).

Tisserant, C. 122 (giganteum); 2323 (torvum); 3543 (torvum).

Tiwari, G.H. AC-941 (forskalii).

To Kang Peng 1146 (procumbens); 2738 (procumbens).

Tolbutt, S.A. 27 (viarum).

Tolstead, W.L. 5859 (elaeagnifolium).

Tomardhamam, K.P. 51506 (insanum).

Tonduz, A. 467 (torvum); 2096 (chrysotrichum); 4817 (mammosum); 8511 (chrysotrichum); 9563 (mammosum); 11833 B (chrysotrichum); 12640 (chrysotrichum); 12786 (mammosum); 12894 (jamaicense); 12894 (jamaicense); 18050 (capsicoides).

Tong Shaoquan 32872 (praetermissum).

Toppin, S. 2105 (insanum).

Toroes, R. si 1639 (cyanocarphium); 1642 (lasiocarpum); 2848 (lasiocarpum); 2926 (cyanocarphium); 5510 (lasiocarpum).

Torre, A.R. 10475 (giganteum).

Torrecillas, E. 208 (elaeagnifolium).

Torres Colín, R. 15684 (elaeagnifolium); 17481 (elaeagnifolium).

Torres R, J.H. 445 (torvum).

Torres, R.B. 678 (sisymbriifolium); 807 (sisymbriifolium).

Torriglia, M. 327 (elaeagnifolium).

Tostain, O. 825 (jamaicense).

Touit, J. 3345 (wrightii).

Toumey, J.W. 398 (elaeagnifolium).

Tovar, O. 7827 (chrysotrichum).

Townsend, C.C. 128 (wightii).

Townsend, C.H.T. 937 (sisymbriifolium).

Toy, B. 4 (wrightii).

Tracey, S.M. 6894 (capsicoides).

Tracy, S.M. 55 (sisymbriifolium); 7576 (elaeagnifolium).

Traut, H. H 129 55 (aethiopicum); H 184 55 (aethiopicum).

Traverse, A. 802 (elaeagnifolium).

Trench, M. EA 13024 (mammosum).

Treutler, W.J. 20 (violaceum); 1257 (virginianum).

Triana, J.J. 192 (sisymbriifolium); 3855 (mammosum).

Tribedi, G.N. 514 (insanum); Pharm-594 (robustum); CNH-1409 (trilobatum); CNH-1435 (trilobatum).

Trigui, S.M. 225 (torvum); 292 (torvum).

Trinta, Z.A. 811 (sisymbriifolium).

Trochain, J.L. 2070 (forskalii).

Troncoso, N.S. 6030 (elaeagnifolium).

Troupin, G. 11254 (aculeatissimum).

Trujillo, B. 12963 (jamaicense); 14590 (jamaicense).

Tsai, H.T. 53448 (violaceum).

Tsam, Y.W. 1831 (insanum).

Tsang, W.T. 30 (procumbens); 137 (procumbens); 161 (procumbens); 207 (insanum); 409 (pseudosaponaceum); 703 (melongena); 811 (pseudosaponaceum); 874 (pseudosaponaceum); 16102 (lasiocarpum); 17668 (procumbens); 21896 (violaceum); 23892 (violaceum); 23902 (pseudosaponaceum); 30055 (insanum); 30369 (violaceum).

Tsiang Ying (=Tsiang, Y.) 847 (torvum); 877 (procumbens); 2232 (procumbens); 2733 (violaceum); 2984 (lasiocarpum).

Tsiang, Y. 459 (torvum); 877 (procumbens); 914 (procumbens); 2733 (violaceum); 7217 (violaceum).

Tso, C.L. 23004 (insanum).

Tsugaru, S. B-775 (jamaicense); B-793 (jamaicense); B-1793 (viarum); 30162 (melongena); 61752 (torvum).

Tsuji, R. 1406 (robustum).

Tsvelev, N.N. 610 (torvum); 1302 (virginianum).

Tucker, J.M. 481 (torvum); 812 (torvum).

Tuerk, M. 5 (viarum).

Tuley, P. 627 (torvum); 628 (aculeatissimum).

Tun Ortíz, R. 1600 (torvum); 2346 (torvum); 2372 (jamaicense); 2670 (torvum).

Türckheim, H. von 2156 (chrysotrichum); 3293 (torvum); 4028 (jamaicense); 8551 (jamaicense); 8557 (torvum).

Turland, N.J. 671 (elaeagnifolium).

Tweedie, J. 29 (elaeagnifolium); 70 (elaeagnifolium); 425 (elaeagnifolium).

Tweedie, M. Mrs 715 (aculeatissimum); 1848 (arundo).

Twisselmann, E.C. 1266 (elaeagnifolium); 2248 (elaeagnifolium); 4791 (elaeagnifolium); 4918 (elaeagnifolium); 9088 (elaeagnifolium); 10232 (elaeagnifolium); 14887 (elaeagnifolium); 15599 (elaeagnifolium).

Ucán Ek, E. 225 (mammosum); 337 (torvum); 703 (mammosum); 4067 (torvum).

Uieda, W. 9357 (robustum); 9365 (robustum).

Ujor, E.U. FHI 30333 (aculeatissimum); FHI 30356 (giganteum).

Uller, H.F. 225 (torvum).

Umana, O.A. FHI 29110 (torvum).

Underhill, P.W.D. B 8946 (arundo).

Uniyal, B.P. NC-37151 (insanum); NC-77338 (insanum); NC-80081 (violaceum); NC-81753 (insanum); NC-92623 (insanum).

Unsicker, J. 134 (elaeagnifolium).

Urdampilleta, J.D. 583 (elaeagnifolium).

Uribe Uribe, L. 1112 (sisymbriifolium); 1113 (torvum); 1204 (mammosum); 3083 (jamaicense); 4211 (jamaicense).

Urrea, G. 41 (melongena).

Urueta, E. 6 (mammosum).

USA Typhus Commission 284 (torvum).

Usha Nanda, ? USHA 142 (hovei).

Utley, J.F. 1154 (torvum); 2451 (chrysotrichum).

Vajravelu, E. SC-19071 (insanum); SC-20505 (insanum); 20582 (pubescens); 20767 (trilobatum); 24392 (pubescens); 26109 (multiflorum); SC-27842 (insanum); 33831 (vagum); 33926 (vagum); 33966 (pubescens); 35170 (multiflorum); 36726 (pubescens); 36827 (multiflorum); 38755 (pubescens); 39423 (pubescens); 39431 (vagum); 39748 (multiflorum); 39793 (multiflorum); 43559 (wightii); 44312 (pubescens); 44891 (multiflorum); 49813 (multiflorum); 51801 (pubescens); 57958 (pubescens); 62898 (multiflorum); 62910 (multiflorum); SC-77822 (capsicoides).

Valdés, J. 55-2 (elaeagnifolium); 15 -1978 (elaeagnifolium).

Vale, G.D. 202 (sisymbriifolium).

Valenzuela, L. 6752 (mammosum).

Valeur, E.J. 1015 (capsicoides).

van der Linden, L. 321 (aculeatissimum).

Van Devender, T.R. 99-379 (elaeagnifolium).

van Hermann, H.A. 655 (jamaicense).

Van Nek, F.I. 1776 (torvum).

Van Someren, G.R.C. 7337 (giganteum).

Vanni, R. 2620 (elaeagnifolium).

Vanoverbergh, M. 30 (torvoideum); 611 (torvoideum); 1979 (insanum); 2020 (torvoideum); 2027 (lasiocarpum).

Varela, F. 713 (elaeagnifolium).

Varela, F.J. de 585 (elaeagnifolium).

Vargas C, I.G. 819 (sisymbriifolium); 2000 (sisymbriifolium); 2331 (sisymbriifolium).

Vargas, C. 23637 (chrysotrichum).

Vargas, L.D. 18 (jamaicense).

Vargas, O. 165 (jamaicense).

Vargas, W. 9534 (jamaicense).

Vartak, V.D. 5556 (hovei).

Vasavada, J.A. 4844 (hovei).

Vasconcellos Neto, J. 6705 (robustum); 9266 (torvum).

Vasconcellos, D. INPA 11076 (jamaicense).

Vasey, G.R. 35 1 (elaeagnifolium); 351 (elaeagnifolium).

Vásquez, R. 10252 (jamaicense); 10270 (mammosum); 10271 (mammosum); 19101 (jamaicense); 20785 (jamaicense); 25193 (capsicoides); 25896 (sisymbriifolium); 27228 (sisymbriifolium); 34668 (jamaicense).

Vatova, A. 479 (aculeatissimum); 939 (aculeatissimum); 1022 (giganteum).

Vaughan, J.H. 3069 (arundo).

Vavrek, M. 216 (sisymbriifolium).

Vázquez, J. 2164 (elaeagnifolium).

Veillon, J.M. 45 (torvum).

Velasco Gutiérrez, K. 40055 (torvum); 40137 (torvum).

Velázquez L, C. 58 (chrysotrichum).

Veldkamp, J.F. 6086 (torvum); 7032 (torvoideum); 7852 (torvum).

Vélez, C. 5836 (jamaicense).

Venkanna, P. 5562 (trilobatum).

Venkata Reddi, B. 93139 (hovei); 93237 (hovei); 93499 (hovei); 96093 (hovei); 97969 (hovei).

Ventura A, A. 1361 (elaeagnifolium); 1509 (elaeagnifolium); 1595 (elaeagnifolium); 1595 (elaeagnifolium).

Ventura A, F. 11982 (jamaicense); 16841 (jamaicense); 17959 (jamaicense).

Ventura, E. 1121 (torvum); 6112 (elaeagnifolium); 6812 (elaeagnifolium); 8022 (elaeagnifolium); 8329 (elaeagnifolium).

Venturi, S. 63 (elaeagnifolium); 1091 (elaeagnifolium); 5450 (sisymbriifolium); 7132 (elaeagnifolium); 7678 (elaeagnifolium); 9320 (sisymbriifolium); 9882 (elaeagnifolium).

Venugopal, N. RHT 15880 (pubescens); RHT-21446 (virginianum).

Verdcourt, B. 72 (robustum); 1780 (wrightii); 2356 (arundo); 3050 (melongena); EA 12303 (melongena).

Vergara, C. 38 (jamaicense).

Verheijen, J.A.J. 2307 (procumbens).

Versteegh, C. BW-8330 (lasiocarpum).

Verugo, A. 340 (sisymbriifolium).

Vesey-Fitzgerald, L.D.E.F. 6517 (aculeatissimum); 16532 /2 (forskalii).

Vidal, J.E. 1802 (insanum); 1803 (insanum); 1804 (lasiocarpum); 5438 (lasiocarpum); 5855 (praetermissum).

Vidal, J.O. 20 (jamaicense).

Vidal, S. 1623 (torvoideum); 1624 (retrorsum); 1625 (torvoideum); 3363 bis (torvoideum); 3363 (torvoideum); 3365 (lasiocarpum); 3366 (retrorsum); 3368 (retrorsum).

Vieira, A.O.S. 153 (sisymbriifolium).

Vieira, L. 10 (viarum).

Vignoli-Silva, M. 292 (torvum).

Viguez, M. 36 (jamaicense).

Vilcapoma, G. 375 (chrysotrichum); 378 (capsicoides); 395 (capsicoides); 520 (elaeagnifolium); 540 (elaeagnifolium).

Villafañe, A. 937 (elaeagnifolium); 1028 (elaeagnifolium); 1265 (elaeagnifolium).

Villafañe, M. 36 (elaeagnifolium); 903 (elaeagnifolium); 993 (elaeagnifolium); 1234 (elaeagnifolium); 1271 (elaeagnifolium).

Vimercat, J.M. 171 (viarum).

Vinas, A. 302 (lasiocarpum).

Vine, R.S. 81 (elaeagnifolium); 176 (wightii); 177 (violaceum); 178 (sisymbriifolium); 180 (robustum).

Viswanathan, M.V. MVV 92 (pubescens); NC-61592 (violaceum).

Vivekananthan, K. SC-20388 (capsicoides); SC-22974 (capsicoides); SC-29328 (capsicoides); 42938 (multiflorum); 45300 (multiflorum); SC-46694 (capsicoides).

Vogel, E.F. de 2504 (poka).

Vogel, T. 52 (melongena).

Vohra, J.N. NC-60310 (insanum).

Voigt, J.O. 281 (violaceum).

Volponi, C.R. 240 (elaeagnifolium); 241 (elaeagnifolium).

Voogd, C.N.A. de 2177 (graciliflorum).

Vorontsova, M.S. 80 (arundo); 156 (aethiopicum); 161 (giganteum); 578 (insanum); 644 (insanum); 1709 (aculeatissimum).

Votava, F. 72 (torvum).

Vratak, V.D. 8564 (elaeagnifolium).

Vuyk, D.H. 275 (giganteum).

Waaijenbrg, H. 24 (macrocarpon).

Waas, S. 439 (torvum); 467 (giganteum).

Wadhwa, B.M. 5219 (forskalii); 5314 (forskalii); CC-8283 (insanum); NC-37197 (chrysotrichum); NC-57713 (violaceum); NC-62734 (insanum); NC-62909 (violaceum); 64027 (hovei); 67378 (hovei); 109821 (hovei); 127608 (hovei); 128061 (hovei); 128221 (hovei).

Wadwha, R.M. CC-5437 (insanum).

Wager, V.A. 39285 (wrightii).

Wagh, S.K. 3681 (pubescens); 3683 (pubescens); 5950 (pubescens); 5951 (pubescens); 5952 (pubescens); 6001 (pubescens); SKW 7665 (multiflorum).

Wagner, R.J. 1228 (jamaicense); 1559 (elaeagnifolium); 1646 (elaeagnifolium); 1880 (elaeagnifolium).

Wakabi, J. W 20 (giganteum).

Walker, G.W. 267 (violaceum).

Wall, E. 2256 (giganteum).

Wallich, N. cat.Burm. 9 (lasiocarpum); cat.Burm. 9 (lasiocarpum); cat.Burm. 135 (insanum); cat.Burm. 176 (violaceum); cat.Burm. 210 (lasiocarpum); cat.Burm. 255 (violaceum); Cat. 262 (torvum); cat.Burm. 465 (virginianum); Cat. 1041 (virginianum); cat.Burm. 1328 [a] (barbisetum); cat.Burm. 1328 [b] (barbisetum); Cat. 2610 b (giganteum); Cat. 2610 a (giganteum); Cat. 2612 B (virginianum); Cat. 2612 (virginianum); Cat. 2622 b (trilobatum); Cat. 2623 c (lasiocarpum); Cat. 2623 b (lasiocarpum); Cat. 2623 d (lasiocarpum); Cat. 2626 a (violaceum); Cat. 2626 d (violaceum); Cat. 2626 (insanum); Cat. 2626 f (insanum); Cat. 2626 e (multiflorum); Cat. 2626 g (insanum); Cat. 2627 b (torvum); Cat. 2627 a (torvum); Cat. 2627 e (torvum); 2627 (torvum); 2627 (torvum); Cat. 2628 b (melongena); H.I. 2628 c (insanum); H.I. 2628 L.b. (insanum); H.I. 2628 L.a. (melongena); Cat. 2628 f (cyanocarphium); cat. 2628 d (melongena); cat. 2628 K (violaceum); cat. 2628 K [2] (melongena); Cat. 2629 b (pubescens); Cat. 2629 [a] (pubescens).

Wallkes, J.V.B. 2695 (lasiocarpum).

Walsh, M.E. 367 (procumbens); 440 (procumbens); 455 (torvoideum).

Wang, C. 32726 (nienkui); 32860 (procumbens); 32863 (melongena); 32965 (pseudosaponaceum); 32999 (lasiocarpum); 33733 (pseudosaponaceum); 35416 (procumbens); 36176 (pseudosaponaceum); 36464 (procumbens); 38866 (pseudosaponaceum).

Wang, C.-.M. 4317 (peikuoense); 4376 (peikuoense); 4720 (peikuoense); 5514 (peikuoense).

Wang, C.W. 76607 (barbisetum); 85018 (violaceum).

Wang, J.-.C. 11628 (peikuoense).

Wang, Y.-.M. 190 (viarum).

Wanghong 2622 (barbisetum).

Warburg, O. 15069 (lasiocarpum); 15072 (schefferi); 15844 (sulawesi); 21251 (dunalianum).

Ward, C.J. 608 (giganteum); 11176 (torvum).

Ward, D.B. 5742 (elaeagnifolium); 5771 (elaeagnifolium).

Warfa, A.M. 100 (arundo).

Warnecke, O. 351 (melongena); 357 (melongena).

Warwick, M. MW-15 (lasiocarpum).

Wasum, R. 2002 (sisymbriifolium).

Waterhouse, J.H.L. 298 (torvum).

Watkins, ? 590 (giganteum).

Watt, G. 7454 (violaceum); 7608 (violaceum); 9778 (insanum); 9819 (violaceum).

Watters, T. 7565 (violaceum).

Wawra, H. 2565 (robustum).

Way, M.J. WSSB-3 (elaeagnifolium).

Weaver Jr, R.E. 2044 (elaeagnifolium).

Webb, J. 328 (torvum).

Webster, G.L. 4825 (torvum); 5039 (jamaicense); 5281 (torvum); 25397 (robustum).

Weinland, K. 133 (dunalianum).

Weitzman, A. 252 (jamaicense).

Welch, J.R. 380 (robustum).

Welsh, S.L. 25320 (elaeagnifolium).

Welwitsch, F.M.J. 3855 (sisymbriifolium); 6076 (macrocarpon); 6077 b (aethiopicum); 6085 (capsicoides); 6091 (aethiopicum); 6092 (aethiopicum); 6093 (aethiopicum); 6094 (aethiopicum).

Wen, J. 10190 (lasiocarpum).

Wendelbo, P. W 7412 (virginianum).

Werdermann, E. 142 (elaeagnifolium).

West, J. 8366 (sisymbriifolium).

West, O. 1148 (aculeatissimum).

Westphal, E. 2734 (macrocarpon).

Whalen, M.D. 850 (chrysotrichum).

Wheeler, H.-.M. 5859 (elaeagnifolium).

Wheeler, J.M. ANU 5987 (torvoideum); ANU 6451 (torvoideum).

White, C.T. 369 (torvoideum).

White, F. 2199 (wrightii); 3812 (aculeatissimum).

White, L.D. 6 (elaeagnifolium).

White, S. 754 (sisymbriifolium); 777 (sisymbriifolium).

White, S.D. 4526 (elaeagnifolium).

White, S.S. 183 (elaeagnifolium); 1180 (elaeagnifolium); 1712 (elaeagnifolium); 2052 (elaeagnifolium); 2526 (elaeagnifolium); 2574 (elaeagnifolium).

Whitefoord, C. 68 (jamaicense); 1092 (jamaicense); 1198 (torvum); 1466 (torvum); 1818 (torvum); 2021 (torvum); 3519 (torvum); 3691 (capsicoides); 5425 (torvum); 6072 (capsicoides); 7286 (capsicoides); 10300 (capsicoides).

Whitford, H.N. 980 (retrorsum).

Whiting, A.F. 905 (elaeagnifolium).

Whiting, A.G.S. 16 (torvum).

Whiting, M.M. 103 (procumbens).

Whitson, M. 803 (elaeagnifolium); 817 (elaeagnifolium).

Whyte, A. 183 (aculeatissimum).

Wiakabu, J. LAE-70377 (wrightii); LAE-70450 (dunalianum); LAE 73480 (lasiocarpum).

Wickens, G.E. 344 (forskalii); 424 (forskalii); 828 (elaeagnifolium).

Widjaja, E.A. 1877 (lasiocarpum).

Widmer, M. 41 (violaceum); 106 (lasiocarpum).

Wieland, R.G. 4322 (forskalii); 4659 [b] (cordatum).

Wieringa, J.J. 254 (torvum).

Wigg, L.T. 1370 (robustum).

Wiggins, I.L. 444 (elaeagnifolium); 11750 (elaeagnifolium); 11778 (elaeagnifolium); 11911 (elaeagnifolium).

Wight, R. 345 (torvum); 585 (lasiocarpum); 586 (multiflorum); 587 (violaceum); 588 (violaceum); 694 (hovei); 1571 (giganteum); 1573 Aa (melongena); 1573 AB (virginianum); 1573 A (melongena); 1573 Ab (insanum); 1573 b (insanum); 1573 Ab (insanum); 1573 c (insanum); 1574 (lasiocarpum); 1575 (multiflorum); 1576 /126 (wightii); 1577 (torvum); 1578 (violaceum); 1579 (virginianum); 1580 (trilobatum); 1581 (pubescens); 2007 (trilobatum); 2011 (pubescens); 2013 (virginianum); 2015 (violaceum); 2016 (wightii); 2017 (trilobatum); 2018 (torvum); 2019 (lasiocarpum); 2019 [a] (multiflorum); 2020 (insanum); 2022 (giganteum); Wall. cat. 2624 b (vagum); Wall.cat. 2629 (pubescens); 5906 (virginianum).

Wijewickrama, T. MO 41 (vagum).

Wilbur, R.L. 7617 (torvum); 7982 (torvum); 14828 (chrysotrichum); 35155 (elaeagnifolium); 35189 (elaeagnifolium); 36096 (elaeagnifolium).

Wilczek, C. 239 (elaeagnifolium).

Wild, H. 4431 (aculeatissimum).

Wilde, J.J.F.E. de 274 (torvum); 9902 (torvum); 11162 (torvum).

Wilde, W.J.J.O. de 7314 (forskalii); 12693 (lasiocarpum); 12799 (violaceum); 19835 (lasiocarpum); 21306 (lasiocarpum).

Wiley, J.R. 32 (jamaicense); 54 A (torvum).

Wilfords, ? 83 (insanum).

Wilks, C.M. 2635 (torvum).

Williams, D.E. 666 (sisymbriifolium).

Williams, G.R. 372 (wrightii).

Williams, L. 129 (jamaicense); 1313 (jamaicense); 1345 (jamaicense); 1482 (jamaicense); 1484 (jamaicense); 1970 (jamaicense); 2180 (jamaicense); 2599 (jamaicense); 3225 (jamaicense); 7978 (jamaicense).

Williams, L.H.J. 61 (violaceum); 62 (torvum); 8102 (viarum); 8223 (torvum); 8268 (virginianum); 8628 (virginianum).

Williams, L.O. 17111 (torvum); 23984 (torvum); 28745 (jamaicense); 40174 (chrysotrichum).

Williams, M.J. 82-26 -1 (elaeagnifolium); 79-176 -1 (elaeagnifolium).

Williams, R.S. 422 (mammosum); 647 (wrightii); 706 (sisymbriifolium); 1074 (retrorsum); 1149 (torvoideum).

Wilson, E.H. 2001 (virginianum); 4197 (violaceum); 4198 (virginianum); 9657 (peikuoense).

Winit, P. 1424 (barbisetum).

Winkler, H.J.P. 163 (cyanocarphium).

Wiriadinata, H. 20 (jamaicense); 428 (lasiocarpum); 590 (torvum); 1198 (torvum).

Wissman, H. von 2493 (pubescens); 2597 (cordatum); 2778 (cordatum); 2973 (pubescens); 3110 (cordatum).

Wit, P. 132 (torvum); 164 (macrocarpon); 2241 (torvum).

Witte, G.F. de 3495 (aculeatissimum); 9723 (giganteum).

Wittman, R.C. 18438 (elaeagnifolium).

Wolf, C.B. 2485 (elaeagnifolium); 2558 (elaeagnifolium); 3086 (elaeagnifolium).

Womersley, J.S. 3408 (torvum); NGF-11021 (lasiocarpum); NGF-37340 (torvoideum).

Wongprasert, T. 997-129 (barbisetum).

Woo, J.P.W. 391 (insanum).

Wood, J.J. C 11 (virginianum); 121 (violaceum).

Wood, J.R.I. Y/75-271 (cordatum); Y/74-419 (virginianum); 2327 (cordatum); 2727 (cordatum); 2765 (cordatum); 3260 (forskalii); 7590 (sisymbriifolium); 8288 (sisymbriifolium); 14339 (viarum); 17872 (sisymbriifolium); 19344 (sisymbriifolium); 22127 (sisymbriifolium).

Woodford, M.H. 2 (melongena).

Woods, P.J.B. 176 (dunalianum).

Woodson, R.E. 1391 (jamaicense).

Woolston, A.L. 285 (robustum); 1512 (robustum).

Wooton, E.O. 59 (elaeagnifolium); 3127 (elaeagnifolium).

Worth, C.R. 16215 (elaeagnifolium).

Woytkowski, F. 5066 (sisymbriifolium); 8122 (sisymbriifolium).

Wrangham, R.W. 41 (aethiopicum); 81 42 (aethiopicum).

Wright, C. 7 (virginianum); 300 (jamaicense); 348 (lasiocarpum); 349 (insanum); 380 (jamaicense); 489 (wrightii); 621 (jamaicense); 1350 [b] (torvum); 1590 (elaeagnifolium).

Wu, M.J. 976 (violaceum).

Wu, S.H. 1273 (peikuoense).

Wuang, C.T. 451 (violaceum).

Wullschlägel, H.R. 392 (torvum); 945 (jamaicense).

Wurdack, J.J. 1039 (sisymbriifolium).

Wykeham Perry, W. 503 (insanum).

Wyld, J.W.G. 809 (macrocarpon); 836 (aethiopicum).

Wynd, F.L. 76 (elaeagnifolium); 780 (elaeagnifolium).

Xiam Expedition 32 (violaceum); 032 (violaceum); 54 (insanum); 56 (capsicoides).

Xuyen, D. HN-NY 357 (torvum).

Yagermani 777 (torvum).

Yang, S.-.Z. 23750 (peikuoense).

Yang, T.Y.A. 1574 (lasiocarpum); 3932 (miyakojimense); 8701 (miyakojimense); 10063 (lasiocarpum); 11473 (miyakojimense); 15044 (miyakojimense).

Yapp, R.H. 148 (torvum).

Yates, H.S. 1175 (violaceum); 1361 (lasiocarpum); 1405 (poka).

Yati, H.A. 412 (torvum).

Yeshoda, K. 172 (violaceum); 413 (pubescens).

Yesoda, N. 703 (pubescens); 1088 (pubescens).

Yi Zhong 3978 (lasiocarpum).

Yip, H.G. 142 (violaceum); 452 (torvum).

York, C.L. 52-300 (elaeagnifolium).

York, H.H. 49 (elaeagnifolium).

Yoshida, S. 2432 (lasiocarpum).

Young, K. 775 A (wrightii).

Yu, T.T. 1352 (virginianum); 14891 (violaceum); 15962 (violaceum).

Yuncker, T.G. 8081 (torvum); 17082 (torvum); 18112 (torvum).

Yunnan expedition 5005 (violaceum).

Zamudio, S. 2305 (elaeagnifolium).

Zanoni, T.A. 11591 (torvum); 20737 (mammosum); 24201 (jamaicense); 29273 (torvum); 30044 (jamaicense); 30419 (capsicoides); 31191 (jamaicense); 33168 (elaeagnifolium); 39584 (torvum); 41215 (mammosum); 41293 (jamaicense); 42719 (torvum).

Zardini, E.M. 5378 (robustum); 5828 (robustum); 5903 (robustum); 13124 (robustum); 17735 (sisymbriifolium); 20130 (robustum); 21372 (robustum); 28216 (sisymbriifolium); 28255 (sisymbriifolium); 28318 (sisymbriifolium); 29294 (sisymbriifolium); 29520 (sisymbriifolium); 31618 (sisymbriifolium); 32271 (robustum); 32281 (sisymbriifolium); 33697 (sisymbriifolium); 33744 (sisymbriifolium); 33865 (sisymbriifolium); 34174 (sisymbriifolium); 34382 (sisymbriifolium); 35656 (sisymbriifolium); 35943 (sisymbriifolium); 36391 (elaeagnifolium); 36894 (sisymbriifolium); 37722 (sisymbriifolium); 39339 (sisymbriifolium); 39521 (sisymbriifolium); 40129 (sisymbriifolium); 40382 (sisymbriifolium); 41810 (sisymbriifolium); 51885 (sisymbriifolium); 56674 (sisymbriifolium); 58064 (sisymbriifolium).

Zarucchi, J.L. 4206 (jamaicense).

Zeng Pei 13316 (nienkui).

Zeyher, C.L.P. 908 (giganteum).

Zhang Jianhou 13639 (lasiocarpum).

Zimmerman, R. 55 (torvum).

Zimmermann, A.W.P. 8072 (giganteum).

Zimmermann, R. 17 (insanum); 25 (melongena); 35 (torvum); 56 (trilobatum).

Zohner, O. 34 (elaeagnifolium).

Zollinger, H. II-129 (involucratum); 139 (involucratum); 212 [a] (torvum); 369 (pseudosaponaceum); 529 (capsicoides); 698 (insanum); 702 (melongena); 1018 (cyanocarphium); 1125 (torvoideum); II-2648 [b] (violaceum); II 2648 [a] (insanum); 2660 (insanum); 2907 (graciliflorum); 3369 z (torvum); II 3773 7c (melongena); 3773 (melongena); III-3825 (cyanocarphium); 26482 (insanum).

Zorello, D. 17 (jamaicense).

Zuloaga, F.O. 9373 (elaeagnifolium); 12096 (elaeagnifolium).

Zuluaga R, S. 1208 (jamaicense); 1230 (jamaicense).

Zwickey, A.L. 52 (torvoideum); 159 (mammosum); 405 (pseudosaponaceum).

Zygadlo, J.A. 4 (elaeagnifolium); 18 (elaeagnifolium); 39 (elaeagnifolium).
